# Supplementary material for: Associations between vision impairment and vision-related interventions on crash risk and driving cessation: systematic review and meta-analysis
Source: BMJ Open. 2023 Aug 11;13(8):e065210. doi: 10.1136/bmjopen-2022-065210 (PMC10423787; doi:10.1136/bmjopen-2022-065210)
Supplement: Supplementary data [file bmjopen-2022-065210supp002.pdf]

Appendix 2 Complete search strategy for review. While search terms were included for driving performance, driving scores and errors, the studies with outcome measures of driving performance was outside of the scope of this current manuscript and are reported elsewhere.

MEDLINE (OVID) search strategy

1. exp Eye Diseases/
2. exp Cataract Extraction/
3. Lens Implantation, Intraocular/
4. Lenses, Intraocular/
5. cataract$.tw.
6. ((intraocular or intra ocular) adj3 lens$).tw.
7. (IOL or IOLs).tw.
8. Vision Tests/
9. Visual Acuity/
10. exp Refractive Errors/
11. Visual Fields/
12. Visual Field Tests/
13. Contrast Sensitivity/
14. Depth Perception/
15. (visual adj2 (acuit$ or field$)).tw.
16. contrast sensitivity.tw.
17. (depth perception or stereopsis).tw.
18. ((impair$ or decreas$ or declin$) adj3 (vision or visual$ or sight$)).tw.
19. (improv$ adj3 (vision or visual$ or sight$)).tw.
20. ((visual or vision) adj2 function$).tw.
21. exp Vision, Ocular/
22. Vision Screening/
23. or/1-22
24. Mass Screening/
25. ((eye$ or sight or vision or visual$) adj2 (test$ or screen$ or exam$ or diagnos$ or assess$)).tw
26. 24 and 25
27. 23 or 26
28. exp Motor Vehicles/
29. exp Automobile Driving/
30. Accidents, Traffic/
31. (driver$ or driving).tw.
32. (automobile$ or car or cars or vehicle$).tw.
33. (motoring or motorcar or "motor car" or "motor cars").tw.
34. crash$.tw.
35. ((road or traffic) adj2 injur$).tw.
36. ((road or traffic or motor) adj2 (accident$ or incident$)).tw.
37. ((road or traffic or motor) adj2 collision$).tw.
38. or/28-37
39. epidemiologic studies/ or case-control studies/ or cohort studies/ or observational study/ or follow-up studies/ or longitudinal studies/ or prospective studies/ or retrospective studies/ or controlled before-after studies/ or cross-sectional studies/ or historically controlled study/ or interrupted time series analysis/
40. epidemiologic methods/ or focus groups/ or interviews as topic/ or exp "surveys and questionnaires"/

41. epidemiologic research design/ or control groups/ or cross-over studies/ or double-blind method/ or meta-analysis as topic/ or network meta-analysis/ or random allocation/ or single-blind method/
42. epidemiologic methods/ or clinical trials as topic/ or feasibility studies/ or multicenter studies as topic/ or pilot projects/ or sampling studies/ or twin studies as topic/
43. randomized controlled trial/ or controlled clinical trials as topic/ or randomized controlled trials as topic/
44. comparative study/ or evaluation studies/ or meta-analysis/ or review/ or multicenter study/ or "systematic review"/ or validation studies/
45. health surveys/
46. outcome assessment, health care/
47. risk factors/
48. self report/
49. (population or cohort or observation$ or intervention$ or prospective or retrospective or comparative).tw.
50. (questionnaire$ or survey$).tw.
51. (randomized or randomised or randomly or RCT).tw.
52. (systematic review or meta-analysis).tw.
53. (before adj2 after).tw.
54. (case$ adj2 control$).tw.
55. (cross adj1 section$).tw.
56. or/39-55
57. 27 and 38
58. 56 and 57
59. vehicle-controlled.tw.
60. (vehicle adj3 inject$).tw.
61. 59 or 60
62. 58 not 61
63. (animal$ or mouse or mice$ or dog or canine or rat or rats or primate$).ti.
64. (dry eye or cell$ or mutation$ or genes or genome or sequencing).ti.
65. or/63-64
66. 62 not 65
67. limit 66 to english language
68. exp case reports/
69. (case adj2 report$).tw.
70. 68 or 69
71. 67 not 70
72. limit 71 to (editorial or letter)
73. 71 not 72

EMBASE Search Strategy

1. exp eye disease/
2. exp cataract extraction/
3. lens implantation/
4. lens implant/
5. cataract$.tw.
6. ((intraocular or intra ocular) adj3 lens$).tw.
7. (IOL or IOLs).tw.
8. vision test/
9. visual acuity/

10. refractive error/
11. visual field/
12. perimetry/
13. contrast sensitivity/
14. depth perception/
15. (visual adj2 (acuit$ or field$)).tw.
16. contrast sensitivity.tw.
17. (depth perception or stereopsis).tw.
18. ((impair$ or decreas$ or declin$) adj3 (vision or visual$ or sight$)).tw.
19. (improv$ adj3 (vision or visual$ or sight$)).tw.
20. ((visual or vision) adj2 function$).tw.
21. vision/
22. or/1-21
23. mass screening/
24. ((eye$ or sight or vision or visual$) adj2 (test$ or screen$ or exam$ or diagnos$ or assess$)).tw.
25. 23 and 24
26. 22 or 25
27. exp car driving/
28. exp motor vehicle/
29. traffic accident/
30. (driver$ or driving).tw.
31. (automobile$ or car or cars or vehicle$).tw.
32. (motoring or motorcar or "motor car" or "motor cars").tw.
33. crash$.tw.
34. ((road or traffic) adj2 injur$).tw.
35. ((road or traffic or motor) adj2 (accident$ or incident$)).tw.
36. ((road or traffic or motor) adj2 collision$).tw.
37. or/27-36
38. study design/
39. controlled clinical trial/
40. case control study/
41. cohort analysis/
42. observational study/
43. follow up/
44. longitudinal study/
45. prospective study/
46. retrospective study/
47. epidemiology/
48. cross-sectional study/
49. control group/
50. crossover procedure/
51. "meta analysis (topic)"/
52. network meta-analysis/
53. randomization/
54. single blind procedure/
55. double blind procedure/
56. "clinical trial (topic)"/
57. "controlled clinical trial (topic)"/
58. "randomized controlled trial (topic)"/
59. "multicenter study (topic)"/
60. feasibility study/

61. pilot study/
62. comparative study/
63. evaluation study/
64. multicenter study/
65. randomized controlled trial/
66. meta analysis/
67. "systematic review"/
68. validation study/
69. interview/
70. questionnaire/
71. outcome assessment/
72. "systematic review (topic)"/
73. health survey/
74. risk factor/
75. self report/
76. evidence based practice/
77. (population or cohort or observation$ or intervention$ or prospective or retrospective or comparative).tw.
78. (questionnaire$ or survey$).tw.
79. (randomized or randomised or randomly or RCT).tw.
80. (systematic review or meta-analysis).tw.
81. (before adj2 after).tw.
82. (case$ adj2 control$).tw.
83. (cross adj1 section$).tw.
84. or/38-83
85. 26 and 37
86. 84 and 85
87. vehicle-controlled.tw.
88. (vehicle adj3 inject$).tw.
89. or/87-88
90. 86 not 89
91. (animal$ or mouse or mice$ or dog or canine or rat or rats or primate$).ti.
92. (dry eye or cell$ or mutation$ or genes or genome or sequencing).ti.
93. or/91-92
94. 90 not 93
95. limit 94 to conference abstract status
96. 94 not 95
97. limit 96 to english language
98. exp case report/
99. (case adj2 report$).tw.
100. or/98-99
101. 97 not 100
102. limit 101 to (conference paper or "conference review" or editorial or letter or note)
103. 101 not 102

GLOBAL HEALTH Search Strategy

1. exp eye diseases/
2. exp vision disorders/
3. cataract$.tw.
4. ((intraocular or intra ocular) adj3 lens$).tw.
5. (IOL or IOLs).tw.

6. (visual adj2 (acuit$ or field$)).tw.
7. contrast sensitivity.tw.
8. (depth perception or stereopsis).tw.
9. ((impair$ or decreas$ or declin$) adj3 (vision or visual$ or sight$)).tw.
10. (improv$ adj3 (vision or visual$ or sight$)).tw.
11. ((visual or vision) adj2 function$).tw.
12. ((eye$ or sight or vision or visual$) adj2 (test$ or screen$ or exam$ or diagnos$ or assess$)).tw.
13. or/1-12
14. drivers/
15. vehicles/
16. motor cars/
17. traffic/
18. traffic accidents/
19. (driver$ or driving).tw.
20. (automobile$ or car or cars or vehicle$).tw.
21. (motoring or motorcar or "motor car" or "motor cars").tw.
22. crash$.tw.
23. ((road or traffic) adj2 injur$).tw.
24. ((road or traffic or motor) adj2 (accident$ or incident$)).tw.
25. ((road or traffic or motor) adj2 collision$).tw.
26. or/14-25
27. cohort studies/
28. case-control studies/
29. longitudinal studies/
30. retrospective studies/
31. epidemiology/
32. exp clinical trials/
33. randomized controlled trials/
34. feasibility studies/
35. pilot projects/
36. meta-analysis/
37. systematic reviews/
38. reviews/
39. questionnaires/
40. surveys/
41. epidemiological surveys/
42. risk factors/
43. (population or cohort or observation$ or intervention$ or prospective or retrospective or comparative).tw.
44. (questionnaire$ or survey$).tw.
45. (randomized or randomised or randomly or RCT).tw.
46. (systematic review or meta-analysis).tw.
47. (before adj2 after).tw.
48. (case$ adj2 control$).tw.
49. (cross adj1 section$).tw.
50. or/27-49
51. 13 and 26
52. 50 and 51
53. (animal$ or mouse or mice$ or dog or canine or rat or rats or primate$).ti.
54. (dry eye or cell$ or mutation$ or genes or genome or sequencing).ti.
55. 53 or 54

56. 52 not 55
57. limit 56 to english language
58. case reports/
59. (case adj2 report$).tw.
60. 58 or 59
61. 57 not 60
62. limit 61 to (conference or conference paper or conference proceedings or correspondence or editorial or thesis)
63. 61 not 62
