# Supplementary material for: Associations between vision impairment and vision-related interventions on crash risk and driving cessation: systematic review and meta-analysis
Source: BMJ Open. 2023 Aug 11;13(8):e065210. doi: 10.1136/bmjopen-2022-065210 (PMC10423787; doi:10.1136/bmjopen-2022-065210)
Supplement: Supplementary data [file bmjopen-2022-065210supp003.pdf]

Appendix 3 Risk of Bias Assessment for all Included Studies

Analytical Cross-Sectional Study

Citation	Q1 (SB)	Q2 (SB)	Q3 (DB)	Q4 (C)	Q5 (C)	Q6(C)	Q7 (DB)	Q8 (V)	Risk of Bias*
**High Income Countries**									
Adler G, et al. 2005.	Y	Y	N	Y	Y	Y	Y	Y	L
Alvarez-Peregrina C et al., 2021	N	N	N	N	N	N	Y	N	H
Ball K, et al. 1993.	Y	N	U	U	N	N	U	Y	H
Cohen Y, et al. 2007.	Y	Y	Y	Y	N	N	Y	Y	M
Crizzle AM et al., 2020	Y	Y	U	U	N	N	Y	Y	M
Cross JM, et al. 2009.	U	U	Y	N	N	U	Y	Y	H
DeCarlo DK, et al. 2003.	Y	Y	N	U	U	U	Y	Y	M
Edwards JD, et al. 2008.	Y	Y	Y	N	Y	Y	Y	Y	L
Garre-Olmo J, et al. 2009.	Y	Y	Y	U	Y	Y	Y	Y	M
Gilhotra JS, et al. 2001.	Y	Y	Y	Y	Y	Y	Y	Y	L
Hajek A, et al. 2019.	Y	Y	N	N	Y	Y	Y	Y	M

Huisingh C, et al. 2015.	Y	Y	Y	Y	Y	Y	Y	Y	L
Ivers RQ, et al. 1999.	N	N	Y	N	Y	Y	N	Y	M
Kaleem MA et al., 2021	Y	Y	Y	Y	N	N	Y	N	M
Keay L, et al. 2016.	Y	Y	Y	Y	Y	Y	Y	Y	L
Kwon M, et al. 2016.	Y	Y	Y	Y	Y	Y	Y	Y	L
Levecq L, et al. 2013.	Y	Y	Y	Y	N	N	Y	Y	M
MacLeod KE, et al. 2014.	Y	Y	Y	Y	Y	Y	Y	Y	L
Marottoli RA, et al. 1993.	Y	Y	N	Y	U	Y	Y	Y	M
Moon SH & Park K et al., 2020	Y	Y	N	N	N	N	Y	Y	M
Ono T, et al. 2015.	Y	Y	Y	Y	Y	Y	N	Y	L
Owsley C, et al. 2001.	Y	Y	Y	Y	Y	Y	Y	Y	L
Owsley C, et al. 2020.	Y	Y	Y	Y	Y	Y	Y	Y	L
Ramulu PY, et al. 2009.	Y	Y	Y	Y	Y	Y	Y	Y	L

Robinson JL et al., 2021	Y	Y	Y	Y	N	N	Y	N	M
Ross LA, et al. 2009.	Y	Y	Y	Y	N	N	Y	Y	M
Runge JW. 2000.	N	N	U	U	U	U	Y	Y	H
Segal-Gidan F, et al. 2010.	Y	Y	Y	Y	Y	Y	Y	Y	L
Sengupta S, et al. 2014.	Y	Y	Y	Y	Y	Y	Y	Y	L
Stafford WR. 1981.	N	N	Y	U	N	N	Y	Y	H
Stewart RB, et al. 1993.	Y	Y	N	N	Y	Y	Y	Y	M
Swain TA et al., 2021	Y	Y	Y	Y	Y	Y	Y	Y	L
Tam ALC, et al. 2018.	Y	Y	Y	Y	N	N/A	Y	Y	M
Tanabe S, et al. 2011.	Y	Y	Y	Y	Y	Y	N	Y	L
van Landingham SW, et al. 2013.	Y	Y	Y	Y	U	U	Y	Y	M
Wedenoja J et al., 2021	Y	Y	U	U	N	N	Y	N	H
Yuki K, et al. 2014.	Y	Y	Y	Y	N	N	N	Y	M

Zebardast N, et al. 2015.	Y	Y	Y	Y	Y	Y	Y	Y	L
**Low Middle Income Countries**									
Abebe Y, et al. 2002.	Y	Y	Y	N	N	N	N	Y	M
Abraham EG, et al. 2010.	Y	N	N	N	N	N	N	Y	H
Adekoya BJ, et al. 2009.	Y	Y	Y	Y	N	N	N	Y	M
Ahmed M et al., 2021	N	N	Y	Y	Y	Y	Y	Y	M
Bekibele CO, et al. 2007.	N	Y	Y	Y	N	N	N	Y	M
Biza M, et al. 2013.	Y	Y	Y	Y	N	N	N	Y	M
Boadi-Kusi SB, et al. 2016.	Y	Y	N	Y	N	N	N	U	H
Emerole CG, et al. 2013.	N	N	Y	Y	N	N	N	N	H
Humphriss D. 1987.	Y	N	N	N	N	N/A	U	U	H
Isawumi MA, et al. 2011.	Y	Y	U	Y	N	N	N	U	H
Ogbonnaya CE, et al. 2018.	Y	Y	Y	Y	N	N	N	Y	M

Oladehinde MK, et al. 2007.	N	N	Y	Y	N	N	N	Y	H
Ovenseri-Ogomo G, et al. 2011.	Y	Y	Y	Y	N	N/A	N	Y	M
Pepple G, et al. 2014.	Y	Y	Y	N	N	N	N	Y	M
Vofo BN et al., 2021	Y	Y	Y	Y	N	N	Y	N	M

*Risk of bias scores: high (1-3), medium (4-6), and low (7-8)

SB= selection bias, DB= detection bias, C= confounding, V= validity

Case Control Study

**Citation**	**Q1 (SB)**	**Q2 (SB)**	**Q3 (SB)**	**Q4 (DB)**	**Q5 (PB)**	**Q6 (C)**	**Q7 (C)**	**Q8 (PB)**	**Q9 (V)**	**Q10 (V)**	**Risk of Bias***
**High Income Countries**											
Campbell MK, et al. 1993.	Y	U	Y	N	U	Y	Y	Y	U	U	M
Gallo JJ, et al. 1999.	Y	N	Y	U	U	Y	Y	Y	N	Y	M
Gresset JA, et al. 1994.	N	Y	Y	Y	Y	Y	Y	Y	Y	Y	L
Gresset JA, et al. 1994.	U	Y	Y	U	U	N	N	Y	Y	Y	M
McCloskey LW, et al. 1994.	Y	Y	Y	Y	Y	U	U	Y	Y	Y	M

McGwin G, et al. 2000.	Y	Y	Y	N	Y	Y	Y	Y	Y	Y	L
McGwin G, et al. 2004.	Y	U	Y	Y	Y	Y	Y	Y	Y	Y	L
McGwin G, et al. 2005.	Y	Y	Y	Y	Y	Y	Y	Y	Y	Y	L
Owsley C, et al. 1998.	Y	Y	Y	Y	Y	N	N	Y	Y	Y	M
Sims RV, et al. 1998.	Y	U	Y	Y	Y	N	N	N	Y	Y	M
Szlyk JP, et al. 1995.	Y	Y	Y	Y	Y	N	N	Y	Y	Y	M
Wood JM, et al. 2018.	Y	Y	N	Y	Y	Y	Y	Y	U	Y	M
Wood JM, et al. 2016.	Y	Y	Y	Y	Y	Y	Y	Y	U	Y	L
Owsley C, et al. 1999.	Y	Y	Y	Y	Y	Y	Y	Y	Y	Y	L
**Low Middle Income Countries**											
Deshmukh AV, et al. 2019.	Y	N	Y	Y	Y	N	N	Y	U	N	M

*Risk of bias scores: high (1-4), medium (5-8), and low (9-10)

SB= selection bias, DB= detection bias, PB = performance bias, C= confounding, V= validity

Case Series

Citation	Q1 (SB)	Q2 (DB)	Q3 (DB)	Q4 (SB)	Q5 (SB)	Q6 (SB)	Q7 (SB)	Q8 (AtB)	Q9 (SB)	Q10 (V)	Risk of Bias*
**High Income Countries**											
Goh YW, et al. 2011	Y	Y	Y	Y	Y	Y	Y	Y	N	Y	L

*Risk of bias scores: high (1-4), medium (5-8), and low (9-10)
SB= selection bias, DB= detection bias, AtB= attrition bias, V= validity

Cohort Study

Citation	Q1 (SB)	Q2 (PB)	Q3 (DB)	Q4 (C)	Q5 (C)	Q6 (SB)	Q7 (DB)	Q8 (V)	Q9 (AtB)	Q10 (AtB)	Q11 (V)	Risk of Bias*
**High Income Countries**												
Anstey KJ, et al. 2006.	Y	Y	Y	Y	Y	Y	Y	Y	Y	Y	Y	L
Baker JM, et al. 2019.	N	Y	N	Y	Y	Y	Y	Y	Y	N/A	Y	M
Fishman GA et al., 1981	Y	U	Y	Y	Y	N	N	U	N	N	N	H
Freeman EE, et al. 2005.	Y	Y	Y	Y	Y	Y	Y	Y	Y	N	Y	L
Green KA, et al. 2013.	Y	Y	Y	Y	Y	U	Y	Y	Y	N	Y	M

Haymes SA, et al. 2007.	Y	Y	Y	Y	Y	N/A	Y	Y	Y	N/A	Y	M
Huisingh C, et al. 2017.	Y	Y	Y	Y	Y	Y	Y	Y	Y	U	Y	L
Huisingh C, et al. 2016.	Y	Y	Y	Y	Y	Y	Y	Y	N	N	Y	M
Janz NK, et al. 2009.	Y	Y	Y	Y	Y	U	Y	Y	N	N	Y	M
Keay L, et al. 2009.	Y	Y	Y	Y	Y	Y	Y	Y	Y	Y	Y	L
Keeffe JE, et al. 2002.	Y	Y	Y	N	N/A	Y	N	Y	Y	U	U	M
Kristalovich L, et al. 2019.	U	Y	Y	N	N	Y	U	Y	N/A	N/A	Y	M
Maag U, et al. 1997.	Y	N	U	Y	Y	N	Y	Y	Y	N/A	Y	M
Margolis KL, et al. 2002.	Y	Y	Y	Y	Y	Y	Y	Y	Y	U	Y	L
McGwin G, et al. 2015.	Y	Y	Y	Y	Y	Y	Y	Y	Y	U	Y	L
McGwin G, et al. 2013.	Y	Y	Y	N	N/A	Y	Y	Y	U	U	Y	M
Meuleners LB,et al. 2019.	Y	Y	Y	Y	Y	Y	Y	Y	U	Y	Y	L

Meuleners LB, et al. 2012.	Y	Y	Y	Y	Y	Y	Y	Y	Y	N/A	Y	L
Meuleners LB, et al. 2012.	Y	Y	Y	Y	Y	Y	Y	Y	U	U	Y	M
Monestam E, et al. 2005.	Y	Y	Y	N	N	Y	Y	Y	N	N	U	M
Monestam E, et al. 1997.	Y	U	Y	N	N	Y	Y	Y	Y	N	Y	M
Naredo Turrado J, et al. 2020.	Y	Y	Y	Y	Y	U	Y	Y	Y	U	Y	M
Owsley C, et al. 2002.	Y	Y	Y	Y	Y	Y	Y	Y	Y	U	Y	L
Rahi JS, et al. 2006.	Y	Y	U	Y	Y	Y	N	Y	N	N	Y	M
Rubin GS, et al. 2007.	Y	Y	Y	Y	Y	Y	Y	Y	Y	N	Y	L
Schlenker MB, et al. 2018.	Y	Y	Y	Y	Y	Y	Y	Y	Y	U	Y	L
Swain TA et al., 2021	Y	Y	Y	Y	Y	Y	Y	Y	Y	U	Y	L
Takahashi A, et al. 2018.	U	Y	Y	Y	Y	Y	Y	Y	N	N	Y	M
Yuki K, et al. 2016.	Y	Y	Y	N	N/A	Y	N	N	Y	U	N	M

*Risk of bias scores: high (1-4), medium (5-9), and low (10-11)

SB= selection bias, PB= performance bias, DB= detection bias, C= confounding, AtB= attrition bias, V= validity

Systematic Reviews

Citation	Q1 (SB)	Q2 (SB)	Q3 (SB)	Q4 (SB)	Q5 (InB)	Q6 (InB)	Q7 (InB)	Q8 (C)	Q9 (PubB)	Q10 (V)	Q11 (V)	Risk of Bias
**High Income Countries**												
Piyasena P et al., 2021	Y	Y	Y	Y	Y	U	Y	Y	Y	Y	Y	L

Risk of bias scores: high (1-4), medium (5-9), and low (10-11)
SB= selection bias, InB= information bias, C= confounding, PubB= publication bias, V= validity

Randomised Controlled Trials

Citation	Q1 (AlB)	Q2 (PB)	Q3 (SB)	Q4 (PB)	Q5 (PB)	Q6 (AlB)	Q7 (C)	Q8 (AtB)	Q9 (V)	Q10 (DB)	Q11 (DB)	Q12 (V)	Q13 (V)	Risk of Bias
**High Income Countries**														
Bressler NM, et al. 2013.	Y	Y	Y	Y	Y	U	Y	Y	Y	Y	Y	Y	Y	L
Bressler NM, et al. 2016.	Y	Y	Y	Y	Y	Y	Y	Y	Y	Y	Y	Y	Y	L

*Risk of bias scores: high (1-6), medium (7-11), and low (12-13)
AlB = allocation bias, SB= selection bias, PB= performance bias, C= confounding, DB= detection bias, AtB= attrition bias, V= validity

Nguyen H, *et al. BMJ Open* 2023; 13:e065210. doi: 10.1136/bmjopen-2022-065210
