# Supplementary material for: Associations between vision impairment and vision-related interventions on crash risk and driving cessation: systematic review and meta-analysis
Source: BMJ Open. 2023 Aug 11;13(8):e065210. doi: 10.1136/bmjopen-2022-065210 (PMC10423787; doi:10.1136/bmjopen-2022-065210)
Supplement: Supplementary data [file bmjopen-2022-065210supp004.pdf]

## **Appendix 4a** Raw data tables and additional narrative summaries of papers on motor vehicle crashes

**Table 4a(i)** All studies (n=16) on glaucoma and Motor Vehicle Crashes (MVC). Of the 16 studies, 5 studies were suitable for meta-analysis on associations with any MVC involvement and 2 studies on associations with at-fault MVC involvement

**Additional Narrative Summary:**
Associations between glaucoma and MVCs were mixed in the studies identified. Even though seven controlled studies found glaucoma to increase the odds of any, injurious, and at-fault MVC involvement, two studies found crash involvement to halve in drivers with glaucoma. Only one study looked at not-at-fault crashes, but found no associations (OR 1 (95% CI 0.4-2.5)). Drivers with more severe glaucoma, irrespective of whether it was in the better or worse eye, were involved in more MVCs and also had greater odds of any crash and at-fault crash involvement compared to drivers without glaucoma and drivers with mild glaucoma.

| **Author and Year** | **Study Design** | **Participants/ Sample Size** | **Mean Age** | **Country** | **VI Definition** | **Comparator** | **Outcome Measure (OR, RR, HR, etc?)** | **Effect Measure (with 95% CI) + any description of results (if appropriate** |
|---|---|---|---|---|---|---|---|---|
| **Included in Meta-analysis (any MVC involvement)** | | | | | | | | |
| Cross JM et al., 2009 | Cross-sectional | 3158 (249/2909) | 71.9 | USA | Self-reported physician diagnosed | Drivers without glaucoma | RR (rate ratio) | **Any MVC**: 1.18 (0.81, 1.72) |
| Haymes S et al., 2007 | Retrospective Cohort | 95 (48/47) | 69 | Canada | Diagnosis from glaucoma specialist, glaucomatous optic disc damage and corresponding visual field damage | Drivers without glaucoma | OR (logistic regression) | **Any MVC**: 6.62 (1.4, 31.23) |
| Kwon M et al., 2016 | Cross-sectional | 1899 (206/1693) | age, no.: 70-79 years = | USA | Physician diagnosed | Drivers without glaucoma | RR (rate ratio) | **Any MVC involvement**: 1.65 (1.2, 2.28) |

| | | | | | | | | |
|---|---|---|---|---|---|---|---|---|
| | | | 1358, 80-89 years = 502, 90-98 years = 39 | | | | | |
| Naredo Turrado J et al., 2020 | Prospective Cohort | 11670 (525/11145) | 62.4 | France | Self-reported physician diagnosed | Drivers without glaucoma | OR | **Any MVC**: 0.93 (0.72, 1.22) |
| McGwin G Jr et al., 2004 | Case Control | 691 (576/115) | 69.2 | USA | ICD-9 codes 365.1 and 265.3 | Drivers without glaucoma | RR (relative risk) | **Any MVC**: 0.58 (0.4, 0.83) |
| **Included in Meta-analysis (at-fault MVC involvement)** | | | | | | | | |
| Cross JM et al., 2009 | Cross-sectional | 3158 (249/2909) | 71.9 | USA | Self-reported physician diagnosed | Drivers without glaucoma | RR (rate ratio) | **At-fault MVC**: 0.91 (0.48, 1.72) |
| Haymes S et al., 2007 | Retrospective Cohort | 95 (48/47) | 69 | Canada | Diagnosis from glaucoma specialist, glaucomatous optic disc damage and corresponding visual field damage | Drivers without glaucoma | OR (logistic regression) | **At-fault MVC**: 12.44 (1.08, 143.99) |
| McGwin G Jr et al., 2004 | Case Control | 691 (576/115) | 69.2 | USA | ICD-9 codes 365.1 and 265.3 | Drivers without glaucoma | RR (relative risk) | **At-fault MVC**: 0.99 (0.54, 1.8) |
| **Included in Narrative Summaries Only – High Income Countries** | | | | | | | | |
| Adler G et al., 2004 | Cross-sectional | 199 (52/147) | 71.3 | USA | Open-or closed-angle glaucoma | Drivers without glaucoma | Prevalence (%) | 25% (13/52) of drivers with glaucoma had been in an MVC compared to |

| | | | | | | | | |
|---|---|---|---|---|---|---|---|---|
| | | | | | | | | 25.9% (38/147) of drivers without glaucoma (p= 0.86). |
| Cross JM et al., 2009 | Cross-sectional | 3158 (249/2909) | 71.9 | USA | Self-reported physician diagnosed | Drivers without glaucoma | RR (rate ratio) | **Injurious MVC**: 0.63 (0.19, 2.06) |
| Haymes S et al., 2007 | Retrospective Cohort | 95 (48/47) | 69 | Canada | Diagnosis from glaucoma specialist, glaucomatous optic disc damage and corresponding visual field damage | Drivers without glaucoma | OR (logistic regression) | **Any MVC (state-reported)**: 3.21 (0.72, 14.27) |
| | | | | | | | | **At-fault MVC (state-reported)**: 7.21 (0.46, 113.4) |
| | | | | | | | Prevalence (%) | 27% (11/400 of drivers with glaucoma had been involved in an MVC compared to 7% (3/44) in drivers without glaucoma.<br><br>20% (8/40) of drivers with glaucoma were at-fault in an MVC compared to 2% (1/44) in drivers without glaucoma. |

| | | | | | | | | |
|---|---|---|---|---|---|---|---|---|
| Kwon M et al., 2016 | Cross-sectional | 1899 (206/1693) | age, no.: 70-79 years = 1358, 80-89 years = 502, 90-98 years = 39 | USA | Physician diagnosed | Drivers without glaucoma | Prevalence (%) | 18% (37/206) of drivers with glaucoma were at-fault in a crash compared to 13% (219/1693) of drivers without glaucoma. |
| McCloskey L et al., 1994 | Case Control | 683 (42/641) | age, no.: 65-69 years = 264, 70-74 years = 195, 75-79 years = 138, 80+ years = 86 | USA | Physician diagnosed (hospital data) | Age-matched drivers with glaucoma who have not been injured in a police-reported MVC in the same calendar year as their matched case. | RR (relative risk) | **Injurious MVC**: 1.5 (0.8, 2.9)* |
| | | | | | | | Prevalence (%) | 7.7% (18/234) of all drivers who had an injurious crash also had glaucoma. |
| McGwin G Jr et al., 2000 | Case Control | 901 (447/454) | N/A | USA | Self-reported physician diagnosed | Not-at-fault drivers involved in crashes, without glaucoma | OR | **Not at-fault MVC**: 1 (0.4, 2.5) |
| | | | | | | | Prevalence (%) | 5.2% (10/198) of not-at-fault crashes involved drivers with |

| | | | | | | | | |
|---|---|---|---|---|---|---|---|---|
| | | | | | | | | glaucoma. 6.9% (17/249) 0f at-fault crashes involved drivers with glaucoma. |
| McGwin G Jr et al., 2004 | Case Control | 691 (576/115) | 69.2 | USA | ICD-9 codes 365.1 and 265.3 | Drivers without glaucoma | RR (relative risk) | **All MVC per person-time**: 0.57 (0.39, 0.83) |
| | | | | | | | | **At-fault MVC per person-time**: 1.02 (0.56, 1.87) |
| | | | | | | | Prevalence (%) | 27% (153/576) of drivers with glaucoma were involved in an MVC compared to 37% (42/115) of drivers without glaucoma.<br><br>15% (87/576) of drivers with glaucoma were at-fault in a crash compared to 12% (14/115) of drivers without glaucoma. |
| McGwin G Jr et al., 2005 | Case Control | 240 (120/120) | 72.9 | USA | ICD-9 codes 365.1 and 265.3, given an AGIS score | Drivers with glaucoma who have not had | OR | **At-fault MVC**: 1.7(0.7, 3.7) |

| | | | | | | | | |
|---|---|---|---|---|---|---|---|---|
| | | | | | from visual fields examinations – **mild defect in better eye** | an MVC between 1994 and 2000. | | |
| | | | | | ICD-9 codes 365.1 and 265.3, given an AGIS score from visual fields examinations **– moderate defect in better eye** | | OR | **At-fault MVC:** 2 (0.7, 5.4) |
| | | | | | ICD-9 codes 365.1 and 265.3, given an AGIS score from visual fields examinations – **severe defect in better eye** | | OR | **At-fault MVC:** 4.2 (0.9, 15.3) |
| | | | | | ICD-9 codes 365.1 and 265.3, given an AGIS score from visual fields examinations – **mild defect in worse eye** | | OR | **At-fault MVC:** 1.9 (0.6, 6.1) |
| | | | | | ICD-9 codes 365.1 and 265.3, given an AGIS score from visual fields examinations – **moderate defect in worse eye** | | OR | **At-fault MVC:** 4.2 (1.2, 15) |

| | | | | | | | | |
|---|---|---|---|---|---|---|---|---|
| | | | | | ICD-9 codes 365.1 and 265.3, given an AGIS score from visual fields examinations – **severe defect in worse eye** | | OR | **At-fault MVC:** 9 (2.4, 33.2) |
| | | | | | ICD-9 codes 365.1 and 265.3, given an AGIS score from visual fields examination – **moderate bilateral defect** | | OR | **Any MVC:** 3.6 (1.4, 9.4) |
| | | | | | ICD-9 codes 365.1 and 265.3, given an AGIS score from visual fields examination – **severe bilateral defect** | | OR | **Any MVC:** 4.4 (1.6, 12.4) |
| Owsley C et al., 1998 | Case Control | 294 (179/155) | 71 | USA | Physician diagnosed | Drivers without glaucoma | OR | **Injurious MVC**: 3.6 (1.2, 10.9)* |
| | | | | | | | | **At-fault MVC**: 1.5 (0.5, 4.8)* |
| | | | | | | | Prevalence (%) | 14.1% (11/78) of all injurious crash drivers had glaucoma. 6.3% (6/101) of all non-injurious crash drivers had glaucoma. |

| | | | | | | | | |
|---|---|---|---|---|---|---|---|---|
| Ono T et al., 2015 | Cross-sectional | 386 (199/187) | 64.7 | Japan | **Mild POAG in the worse eye** as a visual field defect corresponding to a mean deviation (MD) of −6 dB or better | Drivers without glaucoma | OR (logistic regression) | **Any MVC**: 1.07 (0.55, 2.1)* |
| | | | | | **Moderate POAG in the worse eye** as an MD between −6 and −12 dB | | OR (logistic regression) | **Any MVC**: 1.44 (0.68, 3.08)* |
| | | | | | **Severe POAG in the worse eye** as an MD of −12 dB or worse | | OR (logistic regression) | **Any MVC**: 2.28 (1.07, 4.88)* |
| | | | | | **Mild POAG in the better eye** as a visual field defect corresponding to a mean deviation (MD) of −6 dB or better | | OR (logistic regression) | **Any MVC**: 1.36 (0.78, 2.37)* |
| | | | | | **Moderate POAG in the better eye** as an MD between −6 and −12 dB | | OR (logistic regression) | **Any MVC**: 1.82 (0.65, 5.11)* |
| | | | | | **Severe POAG in the better eye** as an MD of −12 dB or worse | | OR (logistic regression) | **Any MVC**: 1.65 (0.39, 6.87)* |

| | | | | | | | | |
|---|---|---|---|---|---|---|---|---|
| | | | | | Physician diagnosis of POAG in any eye | | Prevalence (%) | 22.6% (45/199) pf drivers with glaucoma have been in an MVC compared to 16% (30/187) of drivers without glaucoma. |
| Tanabe S et al., 2011 | Cross-sectional | 265 (121/144) | 61.6 | Japan | Mild POAG as a visual field defect corresponding to a mean deviation (MD) of -5 dB or better in both eyes, moderate POAG as corresponding to an MD of -5 to -10 dB in the worse eye, severe POAG as an MD of -10 dB or worse in the worse eye | Drivers free of ocular disease | OR | **Any MVC (severe glaucoma)**: 9.9 (2.1, 47.8) |
| | | | | | | | Prevalence (%) | 6% (7/121) of drivers with glaucoma have been involved in an MVC compared to 3.5% (5/144) of drivers without glaucoma.<br><br>When dividing by glaucoma severity, 3.9% (2/51) or moderate and 25% (5/20) of severe glaucoma drivers have been involved in a crash. |

| | | | | | | | | |
|---|---|---|---|---|---|---|---|---|
| Wood J et al., 2016 | Case Control | 145 (75/70) | 72.9 | Australia | Visual acuity better than 20/40 with one or both eyes and binocular visual fields with a horizontal extent of at least 110° within 10° above and below the horizontal midline | Age-matched controls without glaucoma | Prevalence (%) | 4% (3/75) of glaucoma drivers had an MVC in the past 12 months compared to 6% (4/70) of drivers without glaucoma; difference was not significant (p= 0.64) |
| | | | | | | | | 19% (14/75) of drivers with glaucoma had an MVC in the past 5 years compared to 23% (16/70) of drivers without glaucoma; difference was not significant (p=0.56) |
| Yuki K et al., 2014 | Cross-sectional | 247 (147/0) | 63.7 | Japan | Severity categorised using Mills Glaucoma Staging system – **better eye** | Drivers without history of MVC | Prevalence (%) | Amongst drivers with a history of MVCs, 11.8% (6/51), 72.5% (37/51), 9.8% (5/51), and 5.9% (3/51) had a better eye glaucoma |

| | | | | | | | | |
|---|---|---|---|---|---|---|---|---|
| | | | | | | | | severity score of 0, 1, 2, 3 or more, respectively. Amongst drivers without a history of MVC, this glaucoma score were: 20.4% (40/196), 65.8% (129/196), 9.2% (18/196), and 4.6% (9/196). The differences between proportion of people assigned these scores in the two MVC groups was not significant (p= 0.86). |
| | | | | | Severity categorised using Mills Glaucoma Staging system – **worse eye** | | | Amongst drivers with a history of MVCs, 2% (1/51), 47.1% (24/51), 23.5% (5/51) and 5.9% (3/51) had a worse eye glaucoma severity score of 0, 1, 2, 3 or more, |

| | | | | | | | | |
|---|---|---|---|---|---|---|---|---|
| | | | | | | | | respectively. Amongst drivers without a history of MVCs, the glaucoma scores were: 2.6% (5/196), 54.6% (107/196), 24.5% (48/196), and 18.3% (36/196), respectively. The differences between proportion of people assigned these scores in the two MVC groups was not significant (p= 0.86). |
| Yuki K et al., 2016 | Prospective Cohort | 191 (191/0) | 63.7 | Japan | Primary open angle glaucoma (POAG) | Drivers with POAG but no history of MVCs. | Prevalence (%) | 15% (28/191) of drivers with glaucoma have been involved in an MVC. Of these, 64.3% (18/28) had mild, 14.3% (4/28) has moderate, and 22.4% (6/27) had severe glaucoma. |
| **Included in Narrative Summaries Only – Low Middle Income Countries** | | | | | | | | |

| | | | | | | | | |
|---|---|---|---|---|---|---|---|---|
| Deshmukh AV et al., 2019 | Case Control | 150 (100/50) | 64.5 | India | Diagnosed glaucomatous optic nerve head changes and corresponding visual field defects, which satisfied Anderson criterion | Aged-matched (older than 40 years) non-glaucoma controls | Prevalence (%) | 12.9% (11/85) of drivers with glaucoma had an MVC in the past 12 months compared to 70% (35/50) of drivers without glaucoma. This significance was significant (p<0.001). |

*unadjusted results

**Table 4a(ii)** All studies (n=8) on cataract and Motor Vehicle Crashes (MVC) with meta-analyses suitable for 2 studies on associations with any MVC involvement

| **Author and Year** | **Study Design** | **Total Participants (exposed/control)** | **Mean Age/Age Range** | **Country** | **VI Definition** | **Comparator** | **Outcome Measure (OR, RR, HR, etc.?)** | **Effect Measure (with 95% Cl) + any description of results (if appropriate)** |
|---|---|---|---|---|---|---|---|---|
| **Included in Meta-analysis (any MVC involvement)** | | | | | | | | |
| Cross JM et al., 2009 | Cross-sectional | 3158 (1165/1993) | 71.9 | USA | Self-reported physician diagnosed | Drivers without cataract | RR (rate ratio) | **Any MVC**: 1.21 (0.95, 1.55) |
| Margolis KL et al., 2002 | Prospective Cohort | 1416 (370/1046) | 71.3 | USA | Self-reported physician diagnosed | Drivers without cataracts. | HR | **Any MVC:** 1.1 (0.88, 1.38) |
| **Included in Narrative Summaries Only – High Income Countries** | | | | | | | | |
| Cross JM et al., 2009 | Cross-sectional | 3158 (1165/1993) | 71.9 | USA | Self-reported physician diagnosed | Drivers without cataract | RR (rate ratio) | **Injurious MVC**: 1.5 (0.85, 2.64) |
| | | | | | | | | **At-fault MVC**: 1.01 (0.69, 1.49) |
| McCloskey L et al., 1994 | Case Control | 683 (118/672) | age, no.: 65- 69 years = 264, 70-74 years = 195, 75-79 years = 138, 80+ years = 86 | USA | Physician diagnosed (hospital data) | Age-matched drivers with cataracts who have not been injured in a police reported MVC in the same calendar year as their matched case. | RR (relative risk) | **Injurious MVC**: 1 (0.7, 1.16)* |
| | | | | | | | Prevalence (%) | 17.9% (42/234) of all injurious MVCs involved drivers with cataract. |

| | | | | | | | | |
|---|---|---|---|---|---|---|---|---|
| McGwin G Jr et al., 2000 | Case Control | 901 (447/454) | N/A | USA | Self-reported physician diagnosed | Not-at-fault drivers without cataract were involved in crashes | OR | **Not-at-fault MVC**: 1.1 (0.7, 1.8) |
| | | | | | | | Prevalence (%) | 35.1% (69/198) of all not-at-fault crashes involved drives with cataract**.**<br>44.6% of all at-fault crashes involved drivers with cataract. |
| Naredo Turrado J et al., 2020 | Prospective Cohort | 11670 (525/11145) | 62.4 | France | Self-reported physician diagnosed | Drivers without cataract | OR | **Any MVC**: 1.27 (0.91, 1.76)* |
| Owsley C et al., 1998 | Case Control | 294 (179/155) | 71 | USA | Physician diagnosed | Drivers without cataract | OR | **Injurious MVC**: 1 (0.6, 1.8)* |
| | | | | | | | | **Non-injurious MVC**: 1.1 (0.6, 1.8)* |
| | | | | | | | Prevalence (%) | 47.4% (37/78) of injurious MVCs involved drivers with cataracts. |
| Owsley C et al., 1999 | Case Control | 384 (279/105) | 69.9 | USA | Cataract in one or both eyes from clinic notes with VA in one eye of 20/40 or worse and no previous | Drivers without cataract | RR (relative risk) | **At-fault MVC**: 2.46 (1, 6.16) |
| | | | | | | | X^2 (Chi Square) | The difference between the number of accidents between drivers with cataract and those |

| | | | | | | | | |
|---|---|---|---|---|---|---|---|---|
| | | | | | cataract surgery in either eye | | | without cataract was non-significant (p= 0.19) |
| Owsley C et al., 2001 | Cross-sectional | 377 (274/103) | 69.9 | USA | Best-corrected VA of 20/40 or worse in worse eye eyes | Crash-free drivers | OR (logistic regression) | **Any MVC:** 1.26 (0.28, 5.59) |
| | | | | | Best-corrected VA of 20/40 or worse in better eyes | | | **Any MVC:** 1.39 (0.42, 4.62) |

*unadjusted results

**Table 4a(iii)** All studies (n=3) on Age-Related Macular Degeneration (AMD) and Motor Vehicle Crashes (MVC) all suitable to only be summarised narratively

| Author and Year | Study Design | Total Participants (exposed/ control) | Mean Age/ Age Range | Country | VI Definition | Comparator | Outcome Measure (OR, RR, HR etc.?) | Effect Measure (with 95% Cl) + any description of results (if appropriate) |
|---|---|---|---|---|---|---|---|---|
| **Included in Narrative Summaries Only – High Income Countries** | | | | | | | | |
| Cross JM et al., 2009 | Cross-sectional | 3158 (88/2070) | 71.9 | USA | Self-reported physician diagnosed | Participants without AMD | RR (rate ratio) | **Any MVC**: 0.57 (0.23, 1.39) |
| | | | | | | | | **Injurious MVC**: 0.9 (0.11, 7.44) |
| | | | | | | | | **At-fault MVC**: 0.95 (0.35, 2.56) |
| McCloskey L et al., 1994 | Case Control | 683 (25/658) | age, no.: 65-69 years = 264, 70-74 years = 195, 75-79 years = 138, 80+ years = 86 | USA | Physician diagnosed (hospital data) | Age-matched drivers with AMD who had not been injured in a police-reported MVC in the same calendar year as their matched case. | RR (relative risk) | **Injurious MVC**: 0.9 (0.4, 2)* |
| | | | | | | | Prevalence (%) | 3.8% (9/234) of drivers with AMD had a history of an MVC. |
| McGwin G Jr et al., 2013 | Retrospective Cohort | 205 (142/63) | 72.7 | USA | AREDS definition for early AMD | Participants without AMD | RR (rate ratio) | **Any MVC**: 0.48 (0.2, 1.18)* **Any MVC per 100 person-** |

| | | | | | | | | | |
|---|---|---|---|---|---|---|---|---|---|
| | | | | | | | | | **years**: 0.67 (0.32, 1.39)* **Any MVC per 1, 000, 000 person-miles**: 0.73 (0.36, 1.5)* |
| | | | | | AREDS definition for intermediate AMD | | | | **Any MVC**: 0.22 (0.08, 0.64)* **Any MVC per 100 person-years**: 0.34 (0.13, 0.89)* **Any MVC per 1, 000, 000 person-miles**: 0.35 (0.13, 0.91)* |
| | | | | | AREDS definition for severe AMD | | | | **Any MVC**: 0.46 (0.14, 1.54)* **Any MVC per 100 person-years**: 0.93 (0.31, 2.77)* **Any MVC per 1, 000, 000 person-miles**: 1.11 (0.38, 3.19)* |
| Szlyk et al., 1995 | Case Control | 21 (10/11) | 73.2 | USA | Physician diagnosed | Age-similar subjects with normal vision | X^2 (Chi Square) | | X2= 4.68 (p<0.03); Age similar controls had more self- |

| | | | | | | | | |
|---|---|---|---|---|---|---|---|---|
| | | | | | | | | reported accidents than those with ARMD. The difference between the groups for the numbers of individuals involved in self-reported accidents was significant |
| | | | | | | Younger control subjects with normal vision | X^2 (Chi Square) | X2= 8.06 (p=0.01); The number of self-reported accidents was significantly different between the younger control group and the ARMD group with the younger control group having more self-reported accidents. |

| | | | | | | | | |
|---|---|---|---|---|---|---|---|---|
| Wood JM et al., 2018 | Case Control | 83 (33/50) | 75.4 | Australia | AREDS definition for late AMD | Aged-matched controls with no AMD | Prevalence with X^2 (Chi Square) | 9% (3/33) of drivers with AMD had a crash in the past 12 months compared to 2% (1/50) of control drivers (p=0.28). 30% (10/33) of drivers with AMD had a history of 1 or more crashes in the past 5 years compared to 16% (8/50) of controls drivers (p=0.23). |

*unadjusted results

**Table 4a(iv)** All studies (n=3) on diabetic retinopathy (DR) and Motor Vehicle Crashes (MVC) all suitable to only be summarised narratively

| Author and Year | Study Design | Total Participants (Exposed/Control) | Mean Age | Country | VI Definition | Comparator | Outcome Measure (OR, RR, HR, etc.?) | Effect Measure (with 95% Cl) + any description of results (if appropriate |
|---|---|---|---|---|---|---|---|---|
| **Included in Narrative Summaries Only – High Income Countries** | | | | | | | | |
| Cross JM et al., 2009 | Cross-sectional | 3158 (98/3060) | 71.9 | USA | Self-reported physician diagnosed | Drivers without DR | RR (rate ratio) | **Any MVC**: 0.6 (0.26, 1.38) |
| | | | | | | | | **Injurious MVC**: 0.95 (0.18, 4.92) |
| | | | | | | | | **At-fault MVC**: 0.32 (0.08, 1.17) |
| McGwin G Jr et al., 2000 | Case Control | 901 (447/454) | N/A | USA | Self-reported physician diagnosed | Drivers without DR involved in not-at-fault crashes | OR | **Not at-fault MVC**: 1.9 (0.3, 10.9) |
| | | | | | | | | **At-fault MVC**: 1.1 (0.3, 3.8) |
| | | | | | | | Prevalence (%) | 1.1% (2/198) of not at-fault crash drivers had DR. 1.6% (3/249) of all at-fault crash drivers had DR. |
| Owsley C et al., 1998 | Case Control | 294 (179/155) | 71 | USA | Physician diagnosed | Drivers without DR | OR | **Non-injurious MVCs**:1 (0.1, 7.5)* |
| | | | | | | | | **Injurious MVCs:** 0.7 (0.1, 8.2)* |

*unadjusted results

**Table 4a(v)** All studies (n=5) on stereopsis impairment and Motor Vehicle Crashes (MVC) with meta-analysis suitable for 3 studies on associations with any MVC involvement

| Author and Year | Study Design | Participants/ Sample Size | Mean Age | Country | VI Definition | Comparator | Outcome Measure (OR, RR, HR?) | Effect Measure (with 95% Cl) + any description of results (if appropriate) |
|---|---|---|---|---|---|---|---|---|
| **Included in Meta-analysis (any MVC involvement)** | | | | | | | | |
| Boadi-Kusi SB et al., 2016 | Cross-sectional | 520 (80/440) | 39.2 | Ghana | Physician diagnosed as abnormal. | Drivers with normal stereopsis | OR | **Any MVC:** 0.89 (0.44, 1.8)* |
| Margolis KL et al., 2002 | Prospective Cohort | 1416 (N/A) | 71.3 | USA | Physician diagnosed - distance depth perception per standard deviation change | Drivers with normal stereopsis. | HR | **Any MVC:** 1.01 (0.92, 1.11) |
| Oladehinde MK et al., 2007 | Cross-sectional | 215 (11/204) | 41.5 | Nigeria | Physician diagnosed - Visual acuity of 6/6 - 6/18 was normal, < 6/18 - 6/60 was classified as visual impairment and < 6/60 - 3/60 was classified as severe visual impairment while visual acuity less than 3/60 was classified as blindness. | Drivers with normal stereopsis | RR (risk ratio) | **Any MVC:** 1.45 (0.42, 5.3)* |

| | | | | | | | | |
|---|---|---|---|---|---|---|---|---|
| **Included in Narrative Summaries Only – High Income Countries** | | | | | | | | |
| Alvarez-Peregrina C et al., 2022 | Cross-sectional | 736 (55/681) | 46.4 | Spain | Physician diagnosed | Drivers with normal stereopsis. | X^2 (Chi Square) | Stereopsis was not linked with history of MVCs (p> 0.05). |
| **Included in Narrative Summaries Only – Low Middle Income Countries** | | | | | | | | |
| Boadi-Kusi SB et al., 2016 | Cross-sectional | 520 (80/440) | 39.2 | Ghana | Physician diagnosed as abnormal. | Drivers with normal stereopsis | Prevalence (%) | 25% (20/30) of drivers with abnormal stereopsis were involved in an MVC. |
| Humphriss D, 1987 | Cross-sectional | 366 (N/A) | N/A | South Africa | Visual acuity of at least 6/12 in each eye separately, or if one eye is below 6/12 then the second eye must be 616 or, wearing glasses and seeing binocularly the acuity must be 6/12. A lateral field of vision of 45 degrees is required. | Drivers with better stereopsis | Mean (SD) | Mean vision test score for stereopsis drivers without MVC involvement: 4.128<br><br>Mean vision test score for stereopsis drivers with MVC involvement: 5 |
| Oladehinde MK et al., 2007 | Cross-sectional | 215 (11/204) | 41.5 | Nigeria | Physician diagnosed - Visual acuity of 6/6 - 6/18 was normal, < 6/18 - 6/60 was classified as visual | Drivers with normal stereopsis | Prevalence (%) | 18.2% (2/11) of all drivers with abnormal stereopsis have been involved in an MVC |

| | | | | | | | | |
|---|---|---|---|---|---|---|---|---|
| | | | | | impairment and < 6/60 - 3/60 was classified as severe visual impairment while visual acuity less than 3/60 was classified as blindness. | | | |

*unadjusted results

**Table 4a(vi)** All studies (n=4) on myopia and Motor Vehicle Crashes (MVC), with 2 studies for meta-analysis

| **Author and Year** | **Study Design** | **Total Participants (exposed/control)** | **Mean Age** | **Country** | **VI Definition** | **Comparator** | **Outcome Measure (OR, RR, HR?)** | **Effect Measure (with 95% Cl) + any description of results (if appropriate)** |
|---|---|---|---|---|---|---|---|---|
| **Included in Meta-analysis (any MVC involvement)** | | | | | | | | |
| Ahmed M et al., 2021 | Cross-sectional | 700 (62/638) | 42.3 | Bangladesh | Physician diagnosed | Drivers without myopia but with a history of MVCs | OR | **Any MVC:** 0.5 (0.15, 1.65)* |
| Boadi-Kusi SB et al., 2016 | Cross-sectional | 520 (10/510) | 39.2 | Ghana | Physician diagnosed – spherical power in the better eye of -0.50D or worse | Drivers without myopia but with a history of MVCs | OR | **Any MVC**: 0.99 (0.41, 2.4)* |
| **Included in Narrative Summaries Only – High Income Countries** | | | | | | | | |
| Cohen Y et al., 2007 | Cross-sectional | 136 (34/102) | 21 | Israel | Night myopia: refraction in illumination and in total darkness in both eyes changed by 0.75 D or more | Drivers without night myopia | Fischer's Exact Test | No statistically significant difference in day time crashes between night myopia and normal subjects (p= 0.22). |
| | | | | | | | | Night myopia drivers had higher night-time crashes than non-night myopia drivers (p=0.044). |

| | | | | | | | | |
|---|---|---|---|---|---|---|---|---|
| McCloskey L et al., 1994 | Case Control | 683 (235/448) | age, no.: 65- 69 years = 264, 70-74 years = 195, 75-79 years = 138, 80+ years = 86 | USA | Physician diagnosed (hospital data) | Age-matched drivers with myopia who have not been injured in a police-reported MVC in the same calendar year as their matched case. | RR (relative risk) | **Injurious MVC**: 0.6 (0.1, 1)* |

*unadjusted results

**Table 4a(vii)** All studies (n=8) on colour vision deficiency (CVD) and Motor Vehicle Crashes (MVC), all suitable to only be summarised narratively due to methodological limitations in non-standardised diagnosis of colour vision deficiencies

**Additional Narrative Summary:**
One study looking at the different types of colour deficiency found individuals with protan colour deficiency, measured by Hardy-Rand Rittler (HRR) pseudo-isochromatic plates, to report significantly more MVCs than those with deutan colour deficiency (p= 0.034).

| Author and Year | Study Design | Total Participants (exposed/control) | Mean Age/Age Range | Country | VI Definition | Comparator | Outcome Measure (OR, RR, HR, etc.?) | Effect Measure (with 95% CI) + any description of results (if appropriate) |
|---|---|---|---|---|---|---|---|---|
| **Included in Narrative Summaries Only – High Income Countries** | | | | | | | | |
| Piyasena P et al., 2021 | Systematic review | 15394 (254/15140) | 39.3 | N/A | Physician diagnosed | Drivers without colour deficiencies. | RR | **Any MVC:** 1.36 (1.01, 1.82)* |
| **Included in Narrative Summaries Only – Low Middle Income Countries** | | | | | | | | |
| Abebe Y et al., 2002 | Cross-sectional | 1878 (85/1794) | 33.5 | Ethiopia | Physician diagnosed - Ishihara plates | Drivers without colour deficiencies. | OR | **Any MVC:** 1.94 (1.18, 3.17) |
| | | | | | | | Prevalence (%) | 32% (27/85) of all drivers with colour blindness were involved in an MVC. |
| Biza M et al., 2013 | Cross-sectional | 249 (4/245) | 33.6 | Ethiopia | Physician diagnosed - Ishihara plates | Drivers without colour deficiencies. | OR | **Any MVC:** 2.34 (0.19, 28.58) |
| | | | | | | | Prevalence (%) | 25% (1/4) of all drivers with colour blindness were involved in an MVC. |
| Boadi-Kusi SB et al., 2016 | Cross-sectional | 520 (37/483) | 39.2 | Ghana | Protan colour deficient – Hardy-Rand | Deutan colour deficient | Prevalence (%) | 52.9% (9/17) of proton colour blindness drivers |

| | | | | | | | | |
|---|---|---|---|---|---|---|---|---|
| | | | | | Rittler (HRR) pseudo-isochromatic plate | | | were involved in an MVC compared to 30.8% (4/13) of deutan colour blindness drivers; X2= 6.194 (p=0.034) |
| | | | | | | | | 35% (13/37) of all colour blind drivers were involved in an MVC. |
| | | | | | | | X^2 (Chi Square) | Protan colour blind drivers were more likely to report MVCs than deutan colour blind drivers: 6.194 (p= 0.034) |
| Isawumi MA et al., 2011 | Cross-sectional | 99 (6/93) | 45.9 | Nigeria | Physician diagnosed - Ishihara plates | Drivers with an MVC history without colour deficiencies. | X^2 (Chi Square) | X^2= 0.09, p=0.76 No significance between the number of MVC involvement in those with colour blindness and those without. |
| | | | | | | | Prevalence (%) | 33% (2/6) of all drivers with colour blindness |

| | | | | | | | | |
|---|---|---|---|---|---|---|---|---|
| | | | | | | | | were involved in an MVC. |
| Oladehinde MK et al., 2007 | Cross-sectional | 215 (7/208) | 41.5 | Nigeria | Physician diagnosed - Ishihara plates | Drivers without a history of MVCs | RR (risk ratio) | **Any MVC:** 1.12 (10.3, 11.5) |
| | | | | | | | Prevalence (%) | 2% (1/57) of all recorded MVCs involved colour blind drivers. |
| | | | | | | | X^2 (Chi Square) | There were no statistically significant associations between colour vision impairment and RTA: 2.3 (p= 0.1) |
| Ovenseri-Ogomo G et al., 2011 | Cross-sectional | 206 (7/199) | 39.2 | Ghana | VA < 6/18 in the better eye) | Drivers without a history of MVCs | X^2 (Chi Square) | No significance found for MVC involvement in drivers with colour blindness: X^2= 2.142, p=0.344 |
| Pepple G et al., 2014 | Cross-sectional | 400 (262/138) | 37.8 | Nigeria | Physician diagnosed - Ishihara plates | Drivers without colour blindness. | RR (did not state test used) | **Any MVC**: 1.23 (p=0.4)* |
| | | | | | | | Prevalence (%) | 56% (10/18) of drivers with colour blindness were involved in an MVC. |

*unadjusted results

**Table 4a(viii)** All studies (n=28) on visual acuity (VA) impairment and Motor Vehicle Crashes (MVC) with meta-analysis suitable for 5 studies on any crash involvement and 2 on at-fault crashes

**Additional Narrative Summary:**
Results for injurious crashes were mixed; all non-significant. Similarly, there were no significant risks found for non-injurious and at-fault crash involvement, irrespective of worsening VA. One study looking at visual acuity in normal and low luminance also found poor acuity to not be a significant predictor of crash risk in both lighting conditions. A Japanese study, however, found the odds of crashing to increase by 20% in drivers with primary open angle glaucoma (POAG) experiencing worse eye declines of 0.01 LogMAR increments compared to those without VA changes.

| Author and Year | Study Design | Total Participants (exposed/control) | Mean Age | Country | VI Definition | Comparator | Outcome Measure (OR, RR, HR?) | Effect Measure (with 95% CI) + any description of results (if appropriate) |
|---|---|---|---|---|---|---|---|---|
| **Included in Meta-analysis (any MVC involvement)** | | | | | | | | |
| Green K et al., 2013 | Retrospective Cohort | 2000 (N/A) | Age, no.: 70-79 years = 1432, 80-89 years = 526, 90-99 years = 40 | USA | VA worse than 20/40 | Drivers with VA 20/40 or better | RR (rate ratio) | **Any MVC**: 1.04 (0.74, 1.48) |
| Huisingh C et a., 2017 | Prospective Cohort | 659 (35/624) | N/A | USA | Distance VA > 0.3logMAR | Drivers with VA 20/40 or better | RR (rate ratio) | **Any MVC:** 0.98 (0.52, 1.84) |
| Margolis KL et al., 2002 | Prospective Cohort | 1416 (N/A) | N/A | USA | 20/40 or worse | Drivers with VA 20/40 or better | HR | **Any MVC:** 1.14 (0.73, 1.8) |
| Piyasena P et al., 2021 | Systematic Review | 15394 (710/14684) | 39.3 | N/A | Physician diagnosed | Drivers without a vision impairment | RR | **Any MVC:** 1.46 (1.2, 1.78) |

| | | | | | | | | |
|---|---|---|---|---|---|---|---|---|
| Rubin G et al., 2007 | Prospective Cohort | 2520 (N/A) | age, no.: 65-69 years = 780, 70-74 years = 829, 77-79 years = 553, 80-85 years = 350 | USA | Physician diagnosed – 15-letter loss of visual acuity (0.3 logMAR i.e. VA 20/40) | Drivers with a VA better than 20/40. | HR | **Any MVC:** 1.06 (0.77, 1.68) |
| **Included in Meta-analysis (at-fault MVC involvement)** | | | | | | | | |
| Green K et al., 2013 | Retrospective Cohort | 2000 (N/A) | Age, no.: 70-79 years = 1432, 80-89 years = 526, 90-99 years = 40 | USA | VA worse than 20/40 = impairment | Drivers with VA better than 20/40 | RR (rate ratio) | **At-fault MVC**: 1.08 (0.71, 1.4) |
| Huisingh C et a., 2017 | Prospective Cohort | 659 (35/624) | N/A | USA | Distance VA > 0.3logMAR | Drivers with VA 20/40 or better | RR (rate ratio) | **At-fault MVC:** 1.09 (0.58, 2.05 |
| **Included in Narrative Summary Only – High Income Countries** | | | | | | | | |
| Alvarez-Peregrina C et al., 2021 | Cross-sectional | 736 (548/188) | 46.4 | Spain | Physician diagnosed - cut-off not defined in study | Drivers without a VA impairment | X^2 (Chi Square) | Poor VA was linked with increased risk of MVCs (p< 0.001). |
| Cross JM et al., 2009 | Cross-sectional | 3158 (1323/1835) | 71.9 | USA | VA worse 20/20 and better 20/40 | Those with binocular acuity of 20/20 or better in any MVC | RR (rate ratio) | **Any MVC:** 1 (0.78, 1.29) |

| | | | | | | | | |
|---|---|---|---|---|---|---|---|---|
| | | | | | VA worse 20/20 and better 20/40 | Those with binocular acuity of 20/20 or better in injurious MVC | | **Injurious MVC:** 0.54 (0.28, 1.01) |
| | | | | | VA worse 20/20 and better 20/40 | Those with binocular acuity of 20/20 or better in at-fault MVC | | **At-fault MVC:** 1.08 (0.72, 1.62) |
| | | | | | VA 20/40 or worse | Those with binocular acuity of 20/20 or better in any MVC | | **Any MVC:** 1.24 (0.74, 2.09) |
| | | | | | VA 20/40 or worse | Those with binocular acuity of 20/20 or better in injurious MVC | | **Injurious MVC:** 0.55 (0.11, 2.8) |
| | | | | | VA 20/40 or worse | Those with binocular acuity of 20/20 or better in at-fault MVC | | **At-fault MVC:** 1.37 (0.66, 2.82) |
| Gresset J et al., 1994 | Case Control | 4036 (151/3885) | N/A | Canada | Physician diagnosed poor VA | Those with better VA | OR | **Any MVC:** 0.99 (0.71, 1.4) |
| | | | | | | | Prevalence (%) | 8.4% (118/1400) of those involved in an MVC had poor VA. |

| | | | | | | | | |
|---|---|---|---|---|---|---|---|---|
| Gresset J et al., 1994 | Case Control | 4021 (N/A) | N/A | Canada | VA equal to 6/12 or 6/15 and normal binocularity | Drivers with VA 20/40 or better | OR | **Any MVC**: 0.97 (0.68, 1.38)* |
| | | | | | VA equal to 6/12 or 6/15 and lack of binocular vision | | | **Any MVC:** 1.23 (0.88, 1.72)* |
| Huisingh C et a., 2017 | Prospective Cohort | 659 (35/624) | N/A | USA | Distance VA > 0.3logMAR | Drivers with VA 20/40 or better | RR (rate ratio) | **Major MVC:** 0.81 (0.29, 2.26) |
| | | | | | | | | **Any MVC:** 1.29 (0.87, 1.93) |
| | | 659 (74/585) | | | Near VA > 0.3 logMAR | | | **Major MVC:** 1.54 (0.9, 2.63) |
| | | | | | | | | **At-fault MVC:** 1.19 (0.77, 1.85) |
| Ivers R et al., 1999 | Cross-sectional | 3654 (N/A) | N/A | Australia | Best eye VA <20/40-20/60 | drivers with Best eye VA >/=20/40 | Prevalence ratio (PR) | **Any MVC:** 1.3 (0.6, 2.8) |
| | | | | | Best eye VA <20/60 | drivers with Best eye VA >/=20/40 | | **Any MVC:** 1.2 (0.3, 5) |
| | | | | | Right eye VA <20/40-20/60 | drivers with Right eye VA >/=20/40 | | **Any MVC:** 0.7 (0.3, 1.6) |
| | | | | | right eye VA<20/60 | drivers with right eye VA >/=20/40 | | **Any MVC:** 2 (1.2, 3.5) |
| | | | | | left eye VA <20/40-20/60 | drivers with left eye VA >/=20/40 | | **Any MVC:** 1.1 (0.5, 2) |

| | | | | | | | | |
|---|---|---|---|---|---|---|---|---|
| | | | | | left eye VA<20/60 | drivers with left eye VA >/=20/40 | | **Any MVC:** 1.1 (0.5, 24) |
| Keeffe JE et al., 2002 | Retrospective Cohort | 2594 (N/A) | 62.5 | Australia | Visual acuity <6/12 | Drivers with better vision (>6/12) | X^2 (Chi Square) | People with impaired vision (<6/12) were no more likely to have an accident or to attribute that the accident was the result of impaired vision; X2= 0.175 (p>0.9) |
| | | | | | | | Prevalence (%) | 9.5% (32/339) of participant involved in an MVC had poor VA. |
| Kwon M et al., 2016 | Cross-sectional | 1899 (145/1754) | age, no.: 70-79 years = 1358, 80-89 years = 502, 90-98 years = 39 | USA | Low VA classified as <20/40 (0.3 logMAR) | Drivers with glaucoma and binocular VA ≥ 20/20 | RR (rate ratio) | **Any MVC:** 1.51 (0.55, 4.16) |
| McCloskey L et al., 1994 | Case Control | 683 | age, no.: 65- 69 years = 264, 70-74 | USA | Uncorrected VA of 20/25 or 20/30 | Drivers with VA 20/15 or 20/20 | RR (relative risk) | **Injurious MVC**: 2.5 (0.8, 7.2)* |

| | | | | | | | | |
|---|---|---|---|---|---|---|---|---|
| | | | years = 195, 75-79 years = 138, 80+ years = 86 | | Uncorrected VA of 20/40 | | | **Injurious MVC:** 1.7 (0.6, 5.3)* |
| | | | | | Uncorrected VA 20/50 or 20/60 | | | **Injurious MVC:** 2.4 (0.8, 7.2)* |
| | | | | | Uncorrected VA 20/70 of greater | | | **Injurious MVC:** 2.1 (0.7, 5.8)* |
| | | | | | Corrected VA 20/25 or 20/30 | | | **Injurious MVC:** 0.7 (0.5, 1.1)* |
| | | | | | Corrected VA 20/40 | | | **Injurious MVC:** 0.6 (0.3, 1.2)* |
| | | | | | Uncorrected VA 20/50 or 20/60 | | | **Injurious MVC:** 0.3 (0.1, 0.9)* |
| | | | | | Uncorrected VA 20/70 of greater | | | **Injurious MVC:** 4.3 (0.5, 40.3)* |
| McGwin G Jr et al., 2000 | Case Control | 901 (104/797) | N/A | USA | Near vision impairment | Not-at-fault drivers involved in crashes without poor near vision | OR | **Not-at-fault MVC:** 1.6 (0.8, 3.3) |
| | | | | | | | Prevalence (%) | 8% (16/198) of not-at-fault MVCs involved drivers with near vision impairment.<br><br>13.2% (33/249) of at-fault MVCs involved drivers |

| | | | | | | | | |
|---|---|---|---|---|---|---|---|---|
| | | | | | | | | with near vision impairment. |
| | | 901 (339/562) | | | Far vision impairment | Not-at-fault drivers involved in crashes without poor far vision | OR | **Not-at-fault MVC:** 1.1 (0.7, 1.7) |
| | | | | | | | Prevalence (%) | 36% (71/198) of not-at-fault crashes involved drivers with far vision impairment.<br><br>41% (102/249) of at-fault crashes involved drivers with far vision impairment. |
| | | 901 (57/844) | | | Peripheral vision impairment | Not-at-fault drivers involved in crashes without poor peripheral vision | OR | **Not-at-fault MVC:** 1.6 (0.7, 3.9) |
| | | | | | | | Prevalence (%) | 4.7% (9/198) of not-at-fault crashes involved drivers with peripheral vision impairment.<br><br>8.5% of at-fault crashes |

| | | | | | | | | |
|---|---|---|---|---|---|---|---|---|
| | | | | | | | | involved drivers with peripheral vision impairment. |
| Ono T et al., 2015 | Cross-sectional | 386 (N/A) | 64.7 | Japan | BCVA in the **better eye** LogMAR per 0.1 increment | POAG drivers with BCVA in both eyes of 0.7 or more | OR | **Any MVC:** 0.94 (0.87, 1.01) |
| Owsley C et al., 2001 | Cross-sectional | 377 (136/241) | 69.9 | USA | VA 20/25 - 20/30 in better eye | Drivers with VA 20/25 or better in the **better eye** | OR | **At-fault MVC:** 1.88 (0.72, 4.88) |
| | | 377 (118/259) | | | VA 20/35 - 20/50 in better eye | | | **At-fault MVC:** 2.54 (0.87, 7.47) |
| | | 377 (77/300) | | | worse than VA 20/50 in better eye | | | **At-fault MVC:** 1.75 (0.45, 6.85) |
| | | 377 (51/326) | | | VA 20/25 - 20/30 in worse eye | Drivers with VA 20/25 or better in the **worse eye** | | **At-fault MVC:** 0.19 (0.03, 1.27) |
| | | 377 (67/310) | | | VA 20/35 - 20/50 in worse eye | | | **At-fault MVC:** 0.82 (0.19, 3.61) |
| | | 377 (110/267) | | | worse than VA 20/50 in worse eye | | | **At-fault MVC:** 0.74 (0.16, 3.52) |
| | | 377 (N/A) | | | VA impairment defined as worse than 20/50 in only 1 eye | Drivers with no VA impairment (better than VA 20/50) | | **At-fault MVC:** 1.35 (0.58, 3.15) |

| | | | | | | | | |
|---|---|---|---|---|---|---|---|---|
| | | 377 (N/A) | | | VA impairment defined as worse than 20/50 in both eyes | Drivers with no VA impairment (better than VA 20/50) | | **At-fault MVC:** 1.01 (0.29, 3.45) |
| Owsley C et al., 1998 | Case Control | 294 (36/258) | 71 | USA | VA worse than 20/40 | Drivers with VA 20/40 or better | OR | **Injurious MVC:** 1.6 (0.6, 3.8)* |
| | | | | | | | | **Non-injurious MVC:** 1.6 (0.7, 3.6)* |
| Rubin G et al., 2007 | Prospective Cohort | 2520 (N/A) | age, no.: 65-69 years = 780, 70-74 years = 829, 77-79 years = 553, 80-85 years = 350 | USA | Physician diagnosed – 15-letter loss of visual acuity (0.3 logMAR i.e. VA 20/40) | Drivers with a VA better than 20/40. | HR | **Any MVC (at low luminance):** 1.06 (0.75, 1.47) |
| Sims RV et al., 1998 | Case Control | 174 (N/A) | 71.1 | USA | Physician Diagnosed | Older drivers without crashes in 6 years preceding 1991 | Univariate analysis using student t-tests | Mean (SD) VA of those with a history of MVCs was 0.09 (0.31), compared to 0.03 (0.19) in those without a history of MVCs (p= 0.001). |
| Yuki K et al., 2014 | Cross-sectional | 247 (N/A) | 63.7 | Japan | Physician diagnosed as better VA (LogMar) | Drivers with POAG but without a | Unpaired t-test with Benjamini's correction | Differences between the VA of those who had a history of |

| | | | | | | | | |
|---|---|---|---|---|---|---|---|---|
| | | | | | | history of MVCs | | an MVC and those who did not was significant, p= 0.036 |
| | | | | | Physician diagnosed as worse VA (logMar) | | | Differences between the VA of those who had a history of an MVC and those who did not was not significant, p= 0.6 |
| Yuki K et al., 2016 | Prospective Cohort | 191 (N/A) | 63.7 | Japan | POAG with 0.01 logMAR increase in **worse eye** | Drivers with POAG but without a history of MVCs | OR | **Any MVC:** 1.2 (1.1, 1.4)* |
| | | | | | POAG with 0.001 increase logMAR in **the better eye** | | | **Any MVC:** 0.68 (0, 221)* |
| **Included in Narrative Summaries only – Low Income Countries** | | | | | | | | |
| Adekoya BJ et al., 2009 | Cross-sectional | 399 (N/A) | 44.7 | Nigeria | VA 6/9 in the better eye | N/A – looked at all participants | X^2 (Chi Square) | Inadequate VA in the better eye is not associated with MVC involvement in the last 10 years; X2= 0.035 (p= 0.851) |

| | | | | | | | | |
|---|---|---|---|---|---|---|---|---|
| | | | | | VA 6/24 in the better eye | | X^2 (Chi Square) | Inadequate VA in the second eye is not associated with involvement in RTA in the last 10 years; X2= 0.372 (p= 0.542) |
| Bekibele CO et al., 2007 | Cross-sectional | 99 (16/83) | 50.1 | Nigeria | Presenting vision less than 6/9 and improved with the aid of a minimum of 0.5 Diopter lenses, with VA <6/18 | Drivers without refractive error | OR | **Any MVC:** 1.2 (0.4, 3.7)* |
| Boadi-Kusi SB et al., 2016 | Cross-sectional | 520 (38/482) | 39.2 | Ghana | Visual acuity of less than 0.2, either monocularly or binocularly, was classified as poor vision | N/A | X^2 (Chi Square) | No statistically significantly associations between poor vision due to refractive error and MVC involvement: X2= 3.090 (p= 0.388) |
| Humphriss D, 1987 | Cross-sectional | 366 (N/A) | N/A | South Africa | Binocular | Better mean vision test scores for binocular VA | Mean (SD) | Drivers involved in accidents were more likely to have worse mean vision test |

| | | | | | | | | |
|---|---|---|---|---|---|---|---|---|
| | | | | | | | | scores (10.031) for binocular VA compared to accident-free drivers (10.847), p<0.001 |
| | | | | | right eye monocular VA | Better mean vision test scores for right eye monocular VA | Mean (SD) | Drivers involved in accidents more likely to have worse mean vision test scores (9.219) for right eye monocular VA compared to accident-free drivers (10.100), p<0.001 |
| | | | | | left eye monocular VA | Better mean vision test scores for left eye monocular VA | Mean (SD) | Drivers involved in accidents more likely to have worse mean vision test scores (9.031) for left eye monocular VA compared to accident-free drivers (10.024), p<0.001 |

| | | | | | | | | |
|---|---|---|---|---|---|---|---|---|
| | | | | | Worse eye monocular acuity | Better mean vision test scores for worse eye monocular VA | Mean (SD) | Drivers involved in accidents more likely to have worse mean vision test scores for depth perception (4.128) compared to accident-free drivers (5.000), p<0.001 |
| Isawumi MA et al., 2011 | Cross-sectional | 99 (5/94) | 45.9 | Nigeria | Poor driving vision if VA <6/12 in either eye | Drivers with an MVC but with normal vision. | X^2 (Chi Square) | MVCs were not directly related with VA and vice versa; X2= 1.6 (p= 0.65) |
| Oladehinde MK et al., 2007 | Cross-sectional | 215 | 41.5 | Nigeria | Visual acuity < 6/18 - 6/60 was classified as visual impairment and < 6/60 - 3/60 was classified as severe visual impairment. VA < 3/60 was classified as blindness. | Drivers with VA 20/20 - 20/40 | RR (did not state test used) | **Any MVC:** 3.5 (2.38, 5.14)* |
| Ogbonnaya CE et al., 2018 | Cross-sectional | 103 (7/96) | 43.2 | Nigeria | Minimum VA of 6/9 in the | Drivers without vision | X^2 (Chi Square) | The relationship between visual |

| | | | | | | | | |
|---|---|---|---|---|---|---|---|---|
| | | | | | better eye and 6/12 in the worse eye of commercial motor vehicle drivers. Visually unfit to drive if VA <6/12 in the poorer eye. | impairment and no MVC history. | | acuity fitness for driving and self-reported history of MVC was not statistically significant; X2= 0.05 (p= 0.82). |
| Ovenseri-Ogomo G et al., 2011 | Cross-sectional | 206 (14/192) | 39.2 | Ghana | VA < 6/18 in the better eye | Drivers without a history of MVC | X^2 (Chi Square) | VA not associated with history of MVC involvement; X^2= 5.982 (p=0.05) |
| Vofo BN et al. 2021 | Cross-sectional | 207 (51/156) | 41.8 | Cameroon | VA < 0.5 | Drivers with VA > 0.5 | Mean (SD) | Drivers with VA < 0.5 had a higher than average number of MVCs (2.91 +/- 1.72) compared to drives with VA > 0.5 (1.01 +/- 1.33). |

*unadjusted results

**Table 4a(ix)** All studies (n=13) on contrast sensitivity (CS) impairment and Motor Vehicle Crashes (MVC), with only two studies suitable for meta-analysis due to different CS cut-off points, type of crash outcome explored and comparators used for each study.

| Author and Year | Study Design | Total Participants (exposure/control) | Mean Age | Country | VI Definition | Comparator | Outcome Measure (OR, RR, HR, etc. ?) | Effect Measure (with 95% Cl) + any description of results (if appropriate) |
|---|---|---|---|---|---|---|---|---|
| **Included in Meta-analysis (any MVC involvement)** | | | | | | | | |
| Huisingh C et al., 2017 | Prospective Cohort | 659 (291/368) | N/A | USA | CS in better eye (< 1.5) | Drivers with CS ≥ 1.5 in better eye | RR (rate ratio) | **Any MVC**: 1.22 (0.82, 1.81) |
| Swain TA et al., 2021 | Cross-sectional | 159 (17/142) | 79.3 | USA | CS of <1.5 log sensitivity in the worse eye | Drivers with CS of >1.5 log sensitivity in the worse eye | RR | **Any MVC:** 1.5 (0.8, 3.2) |
| **Included in Narrative Summaries Only – High Income Countries** | | | | | | | | |
| Cross JM et al., 2009 | Cross-sectional | 3158 (1323/1835) | 71.9 | USA | CS is >=1.575 and <1.675 | Drivers without binocular CS impairments | RR (rate ratio) | **Any MVC:** 0.91 (0.68, 1.23) |
| | | | | | | | | **Injurious MVC:** 0.94 (0.56, 1.58) |
| | | | | | | | | **At-fault MVC**: 0.72 (0.49, 1.05) |
| | | | | | CS is >=1.450 and <1.575 | Drivers without binocular CS | RR (cox proportional hazards) | **Any MVC:** 0.72 (0.49, 1.05) |
| | | | | | | | | **Injurious MVC:** 0.71 (0.32, 1.56) |
| | | | | | | | | **At-fault MVC**: 0.87 (0.49, 1.56) |
| | | | | | CS is <1.450 | Drivers without binocular CS | RR (cox proportional hazards) | **Any MVC:** 1.01 (0.66, 1.55) |
| | | | | | | | | **Injurious MVC:** 0.49 (0.16, 2.37) |

| | | | | | | | | |
|---|---|---|---|---|---|---|---|---|
| | | | | | | | | **At-fault MVC:** 1.27 (0.68, 2.37) |
| Green K et al., 2013 | Retrospective Cohort | 2000 (N/A) | Age, no.: 70-79 years = 1432, 80-89 years = 526, 90-99 years = 40 | USA | Impairment defined as <1.5 on Pelli-Robson chart. | Drivers without binocular CS impairments | RR (rate ratio) | **Any MVC**; 1.42 (1, 2.02) |
| | | | | | | | | **At-fault MVC**: 1.52 (0.93, 2.68) |
| Huisingh C et al., 2017 | Prospective Cohort | 659 (291/368) | N/A | USA | CS in worse eye (< 1.5) | Drivers with CS ≥ 1.5 in worse eye | | **Any MVC:** 1.38 (1.05, 1.81) |
| | | | | | CS in better eye (< 1.5) | Drivers with CS ≥ 1.5 in better eye | | **Major crash involvement**: 1.29 (0.77, 2.18) |
| | | | | | CS in worse eye (< 1.5) | Drivers with CS ≥ 1.5 in worse eye | | **Major crash involvement:** 1.54 (1.07, 2.23) |
| | | | | | CS in better eye (< 1.5) | Drivers with CS ≥ 1.5 in better eye | | **At-fault MVC:** 1.28 (0.84, 1.94) |
| | | | | | CS in worse eye (< 1.5) | Drivers with CS ≥ 1.5 in worse eye | | **At-fault MVC: 1.44** (1.08, 1.93) |
| Ivers R et al., 1999 | Cross-sectional | 3654 (N/A) | N/A | Australia | Vectorvision CSV-1000 chart: 3 cycle per degree in best eye CS | Reference group ≤ 2 units compared with >2 on a scale of 1-8 | PR (Prevalence Ratio) | **Any MVC:** 1.3 (0.7, 2.2) |

| | | | | | | | | |
|---|---|---|---|---|---|---|---|---|
| | | | | | Vectorvision CSV-1000 chart: 6 cycle per degree in best eye CS eye CS | | | **Any MVC:** 1.2 (0.7, 2.1) |
| | | | | | Vectorvision CSV-1000 chart: 12 cycle per degree in best eye CS | | | **Any MVC:** 1.4 (0.8, 2.3) |
| | | | | | Vectorvision CSV-1000 chart: 18 cycle per degree in best eye CS | | | **Any MVC:** 1.4 (0.9, 2.3) |
| | | | | | Vectorvision CSV-1000 chart: 3 cycle per degree in right eye CS | | | **Any MVC:** 1.2 (0.8, 1.9) |
| | | | | | Vectorvision CSV-1000 chart: 6 cycle per degree in right eye CS eye CS | | | **Any MVC:** 1 (0.6, 1.5) |
| | | | | | Vectorvision CSV-1000 chart: 12 cycle per degree in right eye CS | | | **Any MVC:** 2 (1.2, 3.1) |

| | | | | | | | | |
|---|---|---|---|---|---|---|---|---|
| | | | | | Vectorvision CSV-1000 chart: 18 cycle per degree in right eye CS | | | **Any MVC:** 1.3 (0.8, 2.2) |
| | | | | | Vectorvision CSV-1000 chart: 3 cycle per degree in left eye CS | | | **Any MVC:** 1 (0.6, 1.6) |
| | | | | | Vectorvision CSV-1000 chart: 6 cycle per degree in left eye CS eye CS | | | **Any MVC:** 1.1 (0.6, 1.7) |
| | | | | | Vectorvision CSV-1000 chart: 12 cycle per degree in left eye CS | | | **Any MVC:** 1.3 (0.8, 2.2) |
| | | | | | Vectorvision CSV-1000 chart: 18 cycle per degree in left eye CS | | | **Any MVC:** 1.3 (0.8, 2.1) |
| Kwon M et al., 2016 | Cross-sectional | 1899 (432/1467) | age, no.: 70-79 years = 1358, 80-89 years = | USA | Pelli-Robson chart measure of ≤ 1.6 log sensitivity was defined as an impairment. | Older drivers with glaucoma, without CS impairment | RR (rate ratio) | **Any MVC**: 0.72 (0.36, 1.42) |

| | | | | | | | | |
|---|---|---|---|---|---|---|---|---|
| | | | 502, 90-98 years = 39 | | | | | |
| Margolis KL et al., 2002 | Prospective Cohort | 1416 (N/A) | 71.3 | USA | low spatial frequencies per standard deviation change | N/A – looked at MVC information from all participants from 1986-1995 | HR | **Any MVC:** 0.99 (0.89, 1.1) |
| | | | | | high spatial frequencies per standard deviation change | | | **Any MVC:** 0.94 (0.85, 1.04) |
| Owsley C et al., 1998 | Case Control | 294 (56/238) | 71 | USA | Pelli-Robson chart measure of <= 1.5 log sensitivity was defined as an impairment. | Older drivers with log(CS) >1.5 | OR | **Injurious MVC:** 0.9 (0.4, 1.8)* |
| | | | | | | | | **Non-injurious MVC:** 0.7 (0.3, 1.3)* |
| Owsley C et al., 2001 | Cross-sectional | 377 (274/103) | 69.9 | USA | CS impairment defined as ≤1.25 | Participants with no CS impairment (CS >=1.50) | OR | **At-fault MVC (better eye CS >1.35 – 2.50):** 1.18 (0.41, 3.36) |
| | | | | | | | | **At-fault MVC (better eye CS >1.25-1.35):** 1.21 (0.4, 3.68) |
| | | | | | | | | **At-fault MVC (better eye CS ≤1.25):** 3.78 (1.15, 12.48) |

| | | | | | | | | |
|---|---|---|---|---|---|---|---|---|
| | | | | | | | | **At-fault MVC (worse eye CS >1.35 – 2.50):** 3.28 (0.71, 14.17) |
| | | | | | | | | **At-fault MVC (worse eye CS >1.25-1.35):** 4.36 (0.84, 22.7) |
| | | | | | | | | **At-fault MVC (worse eye CS ≤1.25):** 7.86 (1.55, 39.79) |
| | | | | | | | | **At-fault MVC (unilateral CS ≤1.25):** 2.7 (1.16, 6.51) |
| | | | | | | | | **At-fault MVC (bilateral CS ≤1.25):** 5.78 (1.87, 18.86) |
| Owsley C et al., 2020 | Cross-sectional | 915 (179/155) | age, no.: 60-69 years = 310, 70-79 years = 396, 80-90 years = | USA | Low photopic area under the log CS function | Drivers with higher photopic peak log sensitivity | RR (rate ratio) | **All MVC:** 0.8 (0.61, 1.04) |
| | | | | | | | | **At-fault MVC:** 0.77 (0.57, 1.03) |
| | | | | | Low photopic peak log sensitivity | | | **All MVC:** 0.8 (0.61, 1.04) |
| | | | | | | | | **At-fault MVC:** 0.77 (0.58, 1.03) |

| | | | | | | | | |
|---|---|---|---|---|---|---|---|---|
| | | | 200, 90-99 years = 9 | | Low mesopic area under the log CS function | Drivers with higher mesopic peak log sensitivity | | **All MVC:** 1.36 (1.06, 1.72) |
| | | | | | | | | **At-fault MVC:** 1.28 (1.01, 1.63) |
| | | | | | Low mesopic peak log sensitivity | | | **All MVC**: 1.5 (1.16, 1.93) |
| | | | | | | | | **At-fault MVC:** 1.38 (1.07, 1.78) |
| Rubin G et al., 2007 | Prospective Cohort | 2520 (N/A) | age, no.: 65-69 years = 780, 70-74 years = 829, 77-79 years = 553, 80-85 years = 350 | USA | Pelli-Robson Chart: 6 letter worsening (worsening of 0.3 logCS units) | N/A – looked at driver with and without MVC in whole population. | HR | **Any MVC when CS < 1.7**: 0.75 (0.49, 1.21) |
| | | | | | | | | **Any MVC when CS ≥ 1.7**: 1.25 (0.44, 5.65) |
| Swain TA et al., 2021 | Prospective Cohort | 154 (17/137) | 79.3 | USA | CS of <1.5 log sensitivity in the worse eye | Drivers with CS of >1.5 log sensitivity in the worse eye | RR | **At-fault or near crash involvement:** 2.7 (1.3, 5.5) |

**Table 4a(x)** All studies (n=20) on visual field (VF) impairment and Motor Vehicle Crashes (MVC) with meta-analysis suitable for only four studies on associations with any MVC involvement

**Additional Narrative Summary:**
When comparing impairments in the upper, lower, left, right, vertical and horizontal visual fields, impairments on the left side were found to be the most significant predictor of crash involvement (data from US, driving on right side of road).

| **Author and Year** | **Study Design** | **Total Participants (exposure/control)** | **Mean Age** | **Country** | **VI Definition** | **Comparator** | **Outcome Measure (OR, RR, HR, etc.?)** | **Effect Measure (with 95% CI) + any description of results (if appropriate)** |
|---|---|---|---|---|---|---|---|---|
| **Included in meta-analysis (any MVC involvement)** | | | | | | | | |
| Huisingh C et al., 2015 | Cross-sectional | 2000 (N/A) | N/A | USA | Bilateral VF impairment | Drivers without visual field impairments | RR (rate ratio) | **Any MVC:** 1.4 (1.07, 1.83) |
| Oladehinde MK et al., 2007 | Cross-sectional | 215 (22/193) | 41.5 | Nigeria | Bilateral VF impairment | Drivers without a MVC history | RR (risk ratio) | **Any MVC:** 1.07 (0.98, 6.73) |
| Piyasena P et al., 2021 | Systematic Review | 15394 (337/15057) | 39.3 | N/A | Physician Diagnosed | Drivers without VF impairment | RR | **Any MVC:** 1.36 (1.25, 1.48) |
| Swain TA et al., 2021 | Cross-sectional | 159 (40/119) | 79.3 | USA | Overall VF loss of ≤ 22.4dB in the worse eye | Drivers with no overall VF loss in the worse eye | RR | **Any MVC**: 1.6 (0.8, 3.1) |
| **Included in Narrative Summaries Only – High Income Countries** | | | | | | | | |
| Ball K et al., 1993 | Cross-sectional | 294 (N/A) | 71 | USA | Sensitivity loss in the 30 to 60 degree region of the visual field | N/A | Spearman's Correlation (r) | VF loss was significantly related to crash frequency however the LISREL model shows that it only has indirect effects |

| | | | | | | | | |
|---|---|---|---|---|---|---|---|---|
| | | | | | | | | on crash frequency but direct effects on UFOV which is the most significantly associated variable with crash frequency; 0.26 |
| Huisingh C et al., 2015 | Cross-sectional | 2000 (N/A) | N/A | USA | Upper field impairments | Drivers without visual field impairments | RR (rate ratio) | **Any MVC**: 1.1 (0.83, 1.44) |
| | | | | | Lower Field Impairments | | | **Any MVC:** 1.4 (1.07, 1.82) |
| | | | | | Horizontal Meridian Impairments | | | **Any MVC:** 1.31 (1, 1.72) |
| | | | | | Vertical Meridian Impairments | | | **Any MVC:** 1.26 (0.97, 1.65) |
| | | | | | Left Side impairments | | | **Any MVC:** 1.49 (1.15, 1.92) |
| | | | | | Right side impairments | | | **Any MVC:** 1.16 (0.88, 1.53) |
| Huisingh C et al., 2017 | Prospective Cohort | 659 (406/253) | N/A | USA | Peripheral visual field loss at 70 or 85 degrees temporally in **either eye** | Drivers with no visual field loss in either eye | RR (rate ratio) | **Any MVC:** 1.08 (0.8, 1.47) |
| | | | | | | | | **Major MVC:** 1.53 (1.02, 2.29) |
| | | | | | | | | **At-fault MVC:** 0.98 (0.71, 1.37) |

| | | | | | | | | |
|---|---|---|---|---|---|---|---|---|
| | | 659 (186/473) | | | Peripheral visual field loss at 70 or 85 degrees temporally in **both eye** | Drivers with no visual field loss in both eyes | | **Any MVC:** 1.74 (1.18, 2.56) |
| | | | | | | | | **Major MVC:** 2.32 (1.4, 3.83) |
| | | | | | | | | **At-fault MVC:** 0.73 (1.14, 2.61) |
| Kwon M et al., 2016 | Cross-sectional | 1899 (N/A) | age, no.: 70-79 years = 1358, 80-89 years = 502, 90-98 years = 39 | USA | Overall visual field loss ≤ 22.5 dB | Drivers (with glaucoma) without severe visual field loss. | RR (rate ratio) | **Any MVC:** 2.11 (1.09, 4.09) |
| | | | | | Upper visual field loss ≤ 22.5 dB | | | **Any MVC:** 2.37 (1.19, 4.74) |
| | | | | | Lower visual field loss ≤ 22.5 dB | | | **Any MVC:** 2.32 (1.13, 4.75) |
| | | | | | Left visual field loss ≤ 22.5 dB | | | **Any MVC:** 3.16 (1.55, 6.46) |
| | | | | | Right visual field loss ≤ 22.5 dB | | | **Any MVC:** 1.63 (0.84, 3.14) |
| | | | | | Horizontal meridian loss ≤ 22.5 dB | | | **Any MVC:** 1.78 (0.92, 3.44) |
| | | | | | Vertical meridian loss ≤ 22.5 dB | | | **Any MVC:** 1.09 (0.56, 2.11) |
| Kristalovich L et al., 2019 | Retrospective cohort | 445 (286/159) | N/A | Canada | Loss of at least 120 continuous degrees along the horizontal meridian and 15 continuous | Drivers with either no VFI or with VFI but meeting licensing standards | X^2 (Chi Square) | No significant difference in rate of crash between VFI/not meet licensing |

| | | | | | | | | |
|---|---|---|---|---|---|---|---|---|
| | | | | | degrees above and below fixation with both eyes open and examined together. | | | standards and no VFI and VFI/meet licensing standards (p=0.402) |
| McGwin G Jr et al., 2015 | Retrospective Cohort | 438 (N/A) | 72.8 | USA | Binocular visual field **total deviations <7.25** | Drivers (with glaucoma) without severe visual field impairments | RR (rate ratio) | **At-fault MVC:** 1.5 (0.82, 2.74) |
| | | | | | Binocular visual impairment severely **impaired threshold** <20.4 | | | **At-fault MVC:** 1.49 (0.81, 2.74) |
| | | | | | Binocular visual impairment severely impaired **pattern deviation <3.97** | | | **At-fault MVC:** 2.13 (1.21, 3.75) |
| Owsley C et al., 1998 | Case Control | 294 (36/258) | 71 | USA | Central 30 degree VF sensitivity: >10 | Older drivers with central 30 degree VF sensitivity of 0-10 | OR | **Injurious MVC:** 2.6 (1.1, 6.3)* |
| | | | | | | | | **Non-injurious MVC:** 1.8 (0.8, 4.4)* |
| | | 294 (108/186) | | | Peripheral 20-60 degree VF sensitivity: >10 | Older drivers with peripheral 30-60 degree VF sensitivity of 0-10 | | **Injurious MVC:** 2.4 (1.3, 4.5)* |
| | | | | | | | | **Non-injurious MVC:** 1.8 (1, 3.1)* |
| Rubin G et al., 2007 | Prospective Cohort | 2520 (N/A) | age, no.: 65-69 | USA | Binocular visual field <20 (loss of 15 points) | N/A – looked at drivers with and without MVC in | HR | **Any MVC:** 0.59 (0.34, 1) |

| | | | | | | | | |
|---|---|---|---|---|---|---|---|---|
| | | | years = 780, 70-74 years = 829, 77-79 years = 553, 80-85 years = 350 | | Binocular visual field >=20 (loss of 15 points) | whole population. | | **Any MVC:** 1.31 (1.31, 4.27) |
| Swain TA et al., 2021 | Cross-sectional | 159 (41/118) | | | Peripheral VF loss of ≤ 19.2dB in the worse eye | Drivers with no peripheral VF loss in the worse eye | | **Any MVC:** 2.4 (1.3, 4.4) |
| | | 159 (41/118) | | | Superior VL loss of ≤ 22.0dB in the worse eye | Drivers with no superior VF loss in the worse eye | | **Any MVC:** 0.7 (0.4, 1.5) |
| | | 159 (41/118) | | | Inferior VL loss of ≤ 22.1dB in the worse eye | Drivers with no inferior VF loss in the worse eye | | **Any MVC:** 1.7 (0.4, 1.5) |
| | | 159 (40/119) | | | Left VL loss of ≤ 21.6dB in the worse eye | Drivers with no left VF loss in the worse eye | | **Any MVC:** 1.7 (0.9, 3.2) |
| | | 159 (41/118) | | | Right VF loss of ≤ 21.8dB in the worse eye | Drivers with no right VF loss in the worse eye | | **Any MVC:** 1.6 (0.9, 3) |
| Swain TA et al., 2021 | Prospective Cohort | 154 (38/116) | 79.3 | USA | Overall VF loss of ≤ 22.4dB in the worse eye | Drivers with no overall VF loss in the worse eye | RR | **At-fault or near crash:** 1.4 (0.8, 2.8) |

| | | | | | | | | |
|---|---|---|---|---|---|---|---|---|
| | | 154 (40/114) | | | Peripheral VF loss of ≤ 19.2dB in the worse eye | Drivers with no peripheral VF loss in the worse eye | | **At-fault or near crash:** 1.8 (1, 3.3) |
| | | 154 (43/111) | | | Superior VL loss of ≤ 22.0dB in the worse eye | Drivers with no superior VF loss in the worse eye | | **At-fault or near crash:** (1.3 (0.7, 2.5) |
| | | 154 (41/113) | | | Inferior VL loss of ≤ 22.1dB in the worse eye | Drivers with no inferior VF loss in the worse eye | | **At-fault or near crash:** (1.4, 0.8, 2.5) |
| | | 154 (42/112) | | | Left VL loss of ≤ 21.6dB in the worse eye | Drivers with no left VF loss in the worse eye | | **At-fault or near crash:** 1.3 (0.7, 2.5) |
| | | 154 (36/118) | | | Right VF loss of ≤ 21.8dB in the worse eye | Drivers with no right VF loss in the worse eye | | **At-fault or near crash:** 0.9 (0.5, 1.8) |
| Yuki K et al., 2014 | Cross-sectional | 247 (N/A) | 63.7 | Japan | N/A | POAG drivers without a MVC history | Mean (SD) | The mean IVF-MD (db) of glaucoma drivers with a history of MVCS was -0.6 (3.4) compared to -0.8 (3.7) in glaucoma drivers without a history of MVCs. |
| Yuki K et al., 2016 | Prospective Cohort | 191 (N/A) | 63.7 | Japan | POAG with **1dB increase in visual field** | POAG drivers without a MVC history | OR | **Any MVC:** 0.95 (0.8, 1.1)* |
| | | | | | | | Mean (SD) | Mean (SD) IVF-MD (dB) of glaucoma |

| | | | | | | | | |
|---|---|---|---|---|---|---|---|---|
| | | | | | | | | drivers with a history of MVC was -2.1 (3.9) compared to -1.6 (3.7) in glaucoma drivers without a history of MVCs. |
| **Included in Narrative Summaries Only – Low Middle Income Countries** | | | | | | | | |
| Abraham EG et al., 2010 | Cross-sectional | 291 (13/278) | 41.5 | Nigeria | Cup-disc ratio >0.5 cup-disc disparity between the two eyes of up to 0.2 or more, abnormal disc pallor (localised or generalised) | Drivers without visual field impairments. | RR (relative risk) | **Any MVC:** 0.628* |
| Adekoya BJ et al., 2009 | Cross-sectional | 399 (21/378) | 44.7 | Nigeria | Presence of 1 or more abnormal quadrants on confrontation perimetry | N/A | X^2 (Chi Square) | Abnormal visual fields was not associated with MVC involvement in the last 10 years; X2= 1.715 (p= 0.19). |
| Humphriss D, 1987 | Cross-sectional | 366 (N/A) | N/A | South Africa | N/A | Drivers with no MVC history | Mean (SD) | Data not reported |
| Isawumi et al., 2011 | Cross-sectional | 99 (N/A) | 45.9 | Nigeria | N/A | Drivers with a MVC but | Prevalence (%) | 21.1% (8/38) of drivers with an MVC also had |

| | | | | | | | | |
|---|---|---|---|---|---|---|---|---|
| | | | | | | without visual field loss | | horizontal visual field loss. |
| Ovenseri-Ogomo G et al., 2011 | Cross-sectional | 206 (14/192) | 39.2 | Ghana | VA < 6/18 in the better eye | Drivers without a history of MVC | OR | Any MVC: 0.54 (0.016, 18.45)* |
| Pepple G et al., 2014 | Cross-sectional | 400 (16/384) | 37.8 | Nigeria | Physician diagnosed | Drivers without visual field impairments | RR (did not state test used) | **Any MVC:** 1.25* |
| | | | | | | | Prevalence (%) | 56% (9/16) of those with visual field impairment were have been involved in an MVC. |

*unadjusted results

**Table 4a(xi)** All studies (n=3) on glare sensitivity (GS) impairment and Motor Vehicle Crashes (MVC), all suitable to only be summarised narratively due to their different GS cut-off points, type of crash outcome explored and comparators

| Author and Year | Study Design | Total Participants (exposed/control) | Mean Age/ Age Range | Country | VI Definition | Comparator | Outcome Measure (OR, RR, HR, etc.?) | Effect Measure (with 95% CI) + any description of results (if appropriate) |
|---|---|---|---|---|---|---|---|---|
| **Included in Narrative Summaries Only – High Income Countries** | | | | | | | | |
| Owsley C et al., 1998 | Case Control | 294 (71/179) | 71 | USA | Measured using MCT-8000 (Vis Tech), defined as disability glare >0 | Older drivers with disability glare <= 0 | OR | **Injurious MVC**: 1.4 (0.8, 2.5)* |
| | | | | | | | | **Non-injurious MVC:** 1.3 (0.9, 2.2)* |
| Owsley C et al., 2001 | Cross-sectional | 377 (274/103) | 69.9 | USA | Glare impairment defined as >=0.25, measured with Pelli-robson chart with BAT: | Those with disability glare <0.25 in the better/worse eye | OR | **At-fault MVC in the better eye:** 0.68 (0.22, 2.12) |
| | | | | | Glare impairment defined as >=0.25 in both eyes | those with no disability glare impairment (<0.25 score) | OR (logistic regression) | **At-fault MVC in the worse eye**: 0.62 (0.29, 1.33) |
| | | | | | | | | **At-fault MVC in both eyes:** 0.46 (0.14, 1.53) |
| Rubin G et al., 2007 | Prospective Cohort | 2520 (N/A) | age, no.: 65-69 years = 780, 70-74 years = 829, 77-79 years = | USA | 6 letter worsening (worsening of 0.3 logCS units) - measured using Pelli-Robson chart with BAT | N/A – looked at driver with and without MVC in whole population. | HR | **Any MVC (glare <3 letters)**: 0.46 (0.26, 0.89) |
| | | | | | | | | **Any MVC (glare ≥ 3 letters)**: 2.3 (1.14, 16.78) |

| | | | | | | | | |
|---|---|---|---|---|---|---|---|---|
| | | | 553, 80-85 years = 350 | | | | | |

**Table 4a(xii)** All studies (n=19) on other types of vision impairment and Motor Vehicle Crashes (MVC), all suitable to only be summarised narratively

| Author and Year | Study Design | Total Participants (exposure/control) | Mean Age/ Age Range | Country | Type of VI | VI definition | Comparator | Outcome Measure (OR, RR, HR, etc.?) | Effect Measure (with 95% Cl) + any description of results (if appropriate) |
|---|---|---|---|---|---|---|---|---|---|
| **Included in Narrative Summaries only – High Income Countries** | | | | | | | | | |
| Baker JM et al., 2019 | Retrospective Cohort | 66253 (62/66191) | 20.8 | USA | Unilateral vision impairment | ICD-9 diagnostic codes (369.6-369.8) | Young adult drivers without unilateral vision impairment | HR | **Any MVC**: 1.08 (0.6, 1.95) |
| | | 66253 (352/65901) | | | Amblyopia | Using the ICD-9 diagnostic codes (368.00 - 368.03) in the HER with diganosis noted in medical record from age 6 | Young adult drivers without amblyopia | HR | **Any MVC**: 1.08 (0.85, 1.38) |
| Crizzle AM et al., 2020 | Cross-sectional | 3346 (513/2833) | 61.5 | Canada | Vision impairment | Physician diagnosed | Drivers without vision impairment | Univariate log rank test | Vision impairment was not associated with history of MVCs (p=0.9178). |

| | | | | | | | | | |
|---|---|---|---|---|---|---|---|---|---|
| Fishman GA et al., 1981 | Retrospective Cohort | 129 (42/87) | 37.3 | USA | Retinitis Pigmentosa | Physician diagnosed | Drivers free from ophthalmic or general defects | X^2 (Chi Square) | Statistical significant difference in accidents recorded over 5 years between retinitis pigmentosa patients (50%; 21/42)) and controls (29%; 25/62); p= 0.02 |
| Gresset J et al., 1994 | Case Control | 4036 (15/4021) | N/A | Canada | Monocularity | Physician diagnosed | Male drivers who had no accident during their 70th year in 1988 and 1989 | OR | **Any MVC**: 0.95 (0.32, 2.77) |
| | | 4036 (327/3709) | | | Visual impairment | | | OR | **Any MVC:** 1.07 (0.84, 1.36) |
| Maag U et al., 1997 | Retrospective Cohort | 116 (N/A) | N/A | Canada | Vision impairment | Non stereoscopic vision (> 160 seconds); an acuity of 20/40 for the better eye and zero in the other. | Drivers in good health | Mean (SD) | Average total number of crashes in people with good health with a taxi per year (SD): 0.218 (0.501)<br>Average total number of crashes in people with binocular vision problems with |

| | | | | | | | | | |
|---|---|---|---|---|---|---|---|---|---|
| | | | | | | | | | taxi per year (SD): 0.369 (0.595); the difference was statistically significant (p= 0.01) |
| McCloskey L et al., 1994 | Case Control | 683 (10/673) | age, no.: 65-69 years = 264, 70-74 years = 195, 75-79 years = 138, 80+ years = 86 | USA | Retinopathy | Physician diagnosed (hospital data) | Age-matched drivers with retinopathy who have not been injured in a police reported MVC in the same calendar year as their matched case. | RR (relative risk) | **Injurious MVC**: 0.6 (0.1, 2.6)* |
| | | 683 (37/646) | | | Retinal disorders | Physician diagnosed (hospital data) | Age-matched drivers with other retinal disorders who have not been injured in a police | RR (relative risk) | **Injurious MVC**: 0.8 (0.4, 1.6)* |

| | | | | | | | | | |
|---|---|---|---|---|---|---|---|---|---|
| | | | | | | | reported MVC in the same calendar year as their matched case. | | |
| | | 683 (394/289) | | | Hypermetropia | Physician diagnosed (hospital data) | Age-matched drivers with hypermetropia who have not been injured in a police reported MVC in the same calendar year as their matched case. | RR (relative risk) | **Injurious MVC**: 0.9 (0.7, 1.4)* |
| | | 683 (544/139) | | | Presbyopia | Physician diagnosed (hospital data) | Age-matched drivers with presbyopia who have not been injured in a police reported MVC in the same calendar year as their | RR (relative risk) | **Injurious MVC**: 1 (0.6, 1.8)* |

| | | | | | | | | | |
|---|---|---|---|---|---|---|---|---|---|
| | | | | | | | matched case. | | |
| | | 683 (339/344) | | | Astigmatism | Physician diagnosed (hospital data) | Age-matched drivers with astigmatism who have not been injured in a police reported MVC in the same calendar year as their matched case | RR (relative risk) | **Injurious MVC**: 0.9 (0.7, 1.4)* |
| | | 638 (597/41) | | | Refractive disorder | Physician diagnosed (hospital data) | Age-matched drivers with refractive disorders who have not been injured in a police reported MVC in the same calendar year as their matched case | RR (Mantel-Haenszel) | **Injurious MVC**: 0.3 (0.1, 0.8)* |
| | | 638 (6/632) | | | Monocular vision | Physician diagnosed (hospital data) | Age-matched drivers with monocular vision who have not | RR (relative risk) | **Injurious MVC**: 0.7 (0.1, 4.1)* |

| | | | | | | | | | |
|---|---|---|---|---|---|---|---|---|---|
| | | | | | | | been injured in a police reported MVC in the same calendar year as their matched case | | |
| | | 638 (10/628) | | | Diplopia | Physician diagnosed (hospital data) | Age-matched drivers with diplopia who have not been injured in a police reported MVC in the same calendar year as their matched case | RR (relative risk) | **Injurious MVC**: 1.2 (0.4, 4.2)* |
| | | 638 (13/625) | | | Vision/ophthalmic conditions | Physician diagnosed (hospital data) | Age-matched drivers with other vision and opthalmic conditions who have not been injured in a police reported MVC in the same | RR (relative risk) | **Injurious MVC**: 0.6 (0.2, 1.6)* |

| | | | | | | | | | |
|---|---|---|---|---|---|---|---|---|---|
| | | | | | | | calendar year as their matched case | | |
| Naredo Turrado J et al., 2020 | Prospective Cohort | 11670 (11/11659) | 62.4 | France | Retinal detachment | Self-reported physician diagnosed | Drivers without retinal detachment | OR | **Any MVC**: 0.99 (0.37, 2.7) |
| Owsley C et al., 1998 | Case Control | 294 (N/A) | 71 | USA | Stereoacuity | Scores ≥ 500 arcseconds on TNO test | Older drivers with stereoacuity <500 arcseconds | OR (logistic regression) | **Injurious MVC**: 2.2 (1.1, 1.4)* |
| | | | | | | | | | **Non-injurious MVC**: 1.2 (0.7, 2.3)* |
| Pepple G et al., 2014 | Cross-sectional | 400 (32/368) | 37.8 | Nigeri a | Vision impairment | Physician diagnosed | Drivers without a vision impairment | RR (did not state test used) | **Any MVC**: 0.62 (p= 0.46) |
| Rubin G et al., 2007 | Prospective Cohort | 2520 (545/2066) | age, no.: 65-69 years = 780, 70-74 years = 829, 77-79 years = 553, 80-85 years = 350 | USA | Stereoacuity | Stereodeficient was defined at failing the test at 457 arc seconds. | Drivers who were not sterodeficient | HR (cox proportion al hazard) | **Any MVC**: 1.44 (0,88, 2.27) |

| | | | | | | | | | |
|---|---|---|---|---|---|---|---|---|---|
| Runge JW, 2000 | Cross-sectional | N/A | N/A | USA | Vision Impairment | Physician diagnosed | Drivers without vision impairment s | RR (relative risk) | **At-fault MVC**: 1.51*<br><br>The at-fault crash rate of those with a vision impairment was 1.14 compared to those without an impairment (0.75). |
| Rahi J et al., 2006 | Retrospective Cohort | 8661 (429/8432) | N/A | UK | Amblyopia | Mild = acuity 6/6 in one eye and 6/9 or 6/12 in the other and unilateral visual loss | People with normal vision in each eye | OR | **Any MVC**: 1.28 (0.87, 1.89) |
| | | | | | | Moderate/severe = acuity of 6/6 in one eye and 6/18 or worse in the other and unilateral visual loss, with or without strabismus, earlier in childhood. | People with normal vision in each eye | OR (ordinal regression) | **Any MVC**: 2.33 (1.29, 4.2) |
| Wedenoja J et al., 2021 | Cross-sectional | N/A | N/A | Finland | Vision impairment | Physician diagnosed | Drivers without | Prevalence | Only 1.3% (13/968) of all fatal MVCs were |

| | | | | | | | | | |
|---|---|---|---|---|---|---|---|---|---|
| | | | | | | | vision impairment. | | caused by vision-related problems. |
| **Included in Narrative Summaries Only – Low Middle Income Countries** | | | | | | | | | |
| Ahmed M et al., 2021 | Cross-sectional | 700 (492/208) | 42.3 | Bangla desh | Near or distance visual impairment | Presenting VA ≥ 6/7.5 in the better eye and or presence of presbyopia. | Drivers without near or distance visual impairment but with a history of MVCs. | OR | **Any MVC:** 2.45 (1.09, 5.49) |
| | | 700 (125/575) | 42.3 | Bangla desh | Hyperopia | Physician diagnosed | Drivers without hyperopia but with a history of MVCs. | OR | **Any MVC:** 1.1 (0.56, 2.23)* |
| | | 700 (11/689) | 42.3 | Bangla desh | Presbyopia | Physician diagnosed | Drivers without presbyopia but with a history of MVCs. | OR | **Any MVC:** 1.7 (0.96, 3.01)* |
| | | 700 (N/A) | 42.3 | Bangla desh | Any distance refractive error | Physician diagnosed | Drivers without any distance refractive error but with a history of MVCs. | OR | **Any MVC:** 1.66 (0.88, 3.12)* |
| Biza M et al., 2013 | Cross-sectional | 249 (13/236) | 33.6 | Ethiop ia | Visual impairment | VA <6/18-6/60 was classified as | Drivers with a MVC but no | OR | **Any MVC (both eyes** |

| | | | | | | | | | |
|---|---|---|---|---|---|---|---|---|---|
| | | | | | | moderate visual impairment and <6/60-3/60 was classified as severe VI while VA less than 3/60 was classified as blindness. | VA impairment | | **impairment)**:42.82 (2.53, 724.03) |
| | | | | | | | | | **Any MVC (right eye impairment)**: 0.03 (0.004, 0.28)* |
| | | | | | | | | | **Any MVC (left eye impairment)**: 0.09 (0.01, 0.97)* |
| Boadi-Kusi SB et al., 2016 | Cross-sectional | 520 (66/454) | 39.2 | Ghana | Hyperopia | Hyperopia defined as the spherical power in the better eye of +1.00D or more | Drivers with a history of MVC but no hyperopia | OR | **Any MVC**: 0 (0, 0); |
| | | 520 (30/490) | | | Astigmatism | Astigmatism was defined as −0.50D cylinder or worse in the better eye | Drivers with a history of MVC but no astigmatism | OR | **Any MVC**: 0.885 (0.32, 2.5)* |
| Emerole C et al., 2013 | Cross-sectional | 280 (102/178) | N/A | Nigeria | Vision impairment causing poor visibility. | Physician diagnosed with VA of 6/30 classified as abnormal. | N/A – compared with a “control” group but paper never explained what/who | Prevalence (%) | 119 (79.3%) participants in the study group had an MVC history.<br><br>40.3% (448/119) participants in the study group |

| | | | | | | | | | |
|---|---|---|---|---|---|---|---|---|---|
| | | | | | | | the control group was. | | and 70.6% (36/51) in the control group listed poor visibility as the cause of their MVC involvement (p < 0.05). |
| Ogbonnaya CE et al., 2018 | Cross-sectional | 103 (9/94) | 43.2 | Nigeria | Monocular vision impairment | Physician diagnosed | Drivers with monocular impairment but with no MVC history | X^2 (Chi Square) | The relationship between monocular visual impairment and self-reported history of RTA was not statistically significant; X2 =0.045, (p= 0.85) |
| | | 103 (7/96) | | | Monocular blindness | Physician diagnosed | Drivers with monocular blindness but with no MVC history | X^2 (Chi Square) | The relationship between monocular blindness and self-reported history of RTA was not statistically significant; X2 =0.358 (p= 0.55) |
| Vofo BN et al., 2021 | Cross-sectional | 207 (51/156) | 41.8 | Cameroon | Self-reported vision impairment. | Self-reported | Drivers without self-reported | X^2 (Chi Square) | Drivers with self-reported VI were involved in significantly |

| | | | | | | | | | |
|---|---|---|---|---|---|---|---|---|---|
| | | | | | | | vision impairment | | higher number of MVCs (72.5%) than those with self-reported good vision (55.8%) (p< 0.05) |
| | | | | | | | | | Drivers with self-reported VI had higher average number of MVCs over previous 10 years (1.75 +/- 1.64) than drivers with self-reported good vision (1.03 +/- 1.40 (p< 0.05). |

*unadjusted results

**Table 4a(xiii)** All studies (n=6) evaluating cataract surgery and Motor Vehicle Crashes (MVC) with meta-analysis suitable for 3 studies on the associations with any MVC involvement

| **Author and Year** | **Study Design** | **Total Participants (exposure/control)** | **Mean Age** | **Country** | **VI Definition** | **Comparator** | **Outcome Measure (OR, RR, HR?)** | **Effect Measure (with 95% CI)** |
|---|---|---|---|---|---|---|---|---|
| **Included in Meta-Analysis (any MVC involvement)** | | | | | | | | |
| Meuleners L et al., 2012 | Retrospective Cohort | 27827 (N/A) | age, no.: 60-69 years = 6609, 70-79 years = 14506, 80+ years = 6712 | Australia | Physician diagnosed | Crashes before surgery | RR (risk ratio) | 0.87 (0.76, 0.99) |
| Meuleners L et al., 2019 | Retrospective Cohort | 2849 (N/A) | age, no.: 60-64 years = 347, 65-69 years = 482, 70-74 years = 720, 75-79 years = 719, 80-84 years = 454, 85 + | Australia | Physician diagnosed cataract | Crashes before surgery | RR (risk ratio) | 0.39 (0.37, 0.41) |

| | | | | | | | | |
|---|---|---|---|---|---|---|---|---|
| | | | years = 127 | | | | | |
| Owsley C et al., 2002 | Prospective Cohort | 277 (174/103) | 71.3 | USA | Cataract in 1 or both eyes with best-corrected VA of 20/40 or worse | Crashes before surgery | RR (rate ratio) | 0.47 (0.23, 0.94) |
| **Included in Narrative Summaries Only – High Income Countries** | | | | | | | | |
| McCloskey L et al., 1994 | Case Control | 683 (235/448) | age, no.: 65- 69 years = 264, 70-74 years = 195, 75-79 years = 138, 80+ years = 88 | USA | Self-reported physician diagnosed cataracts | Drivers who experienced no injuries in a crash. | RR (relative risk) | **Post-surgery with lens implant:**1 (0.5, 2.3)* |
| Meuleners L et al., 2012 | Retrospective Cohort | Males: 1091 (513/611) | age, no.: 60-69 years = 447, 70-79 years = 823, 80 + years = 445 | Australia | Physician diagnosed | No. of pre cataract surgery police reported crashes in all participants. | RR (risk ratio) | **Males:** 0.84 (0.72, 0.99) |
| | | Females: 624 (308/330) | | | | | | **Females**: 0.99 (0.75, 1.16) |
| Meuleners L et al., 2019 | Retrospective Cohort | 2849 (N/A) | age, no.: 60-64 years = 347, 65-69 years | Australia | Physician diagnosed cataract | Crashes before surgery | RR (risk ratio) | **After 2nd eye cataract surgery:** 0.77 (0.75, 0.78) |

| | | | | | | | | |
|---|---|---|---|---|---|---|---|---|
| | | | = 482, 70-74 years = 720, 75-79 years = 719, 80-84 years = 454, 85 + years = 127 | | | | | |
| Schlenker M et al., 2018 | Prospective cohort | 559546 (N/A) | 76 | Canada | Physician diagnosed | No. of pre cataract surgery crashes in all participants | OR | 0.91 (0.84, 0.97)* |

*unadjusted results

**Table 4a(xiv)** All studies (n=1) evaluating corrective lens wear to improve refractive error and Motor Vehicle Crashes (MVC)

| **Author and Year** | **Study Design** | **Total Participants (exposure/control)** | **Mean Age** | **Country** | **Vision impairment** | **VI Definition** | **Comparator** | **Outcome Measure (OR, RR, HR?)** | **Effect Measure (with 95% CI)** |
|---|---|---|---|---|---|---|---|---|---|
| McCloskey L et al., 1994 | Case Control | 683 (235/448) | age, no.: 65- 69 years = 264, 70-74 years = 195, 75-79 years = 138, 80+ years = 94 | USA | Refractive Error | Use of corrective lenses for any reason (far or near vision) | Drivers who experienced no crash-related injuries | RR (risk ratio) | 0.6 (0.3, 1.1)* |
| | | | | | | | | Prevalence (%) | % with condition, cases: 91% (214/235) % with condition, controls: 94.6% (424/448) |

* unadjusted results

## **Appendix 4b** Raw data tables and additional narrative summaries of papers on driving cessation

**Table 4b(i)** All studies (n=13) on glaucoma and driving cessation with meta-analysis suitable for 2 studies

**Additional Narrative Summaries:**
Persons with bilateral glaucoma (OR 2.6 (95% Cl 1.4-4.8); p= 0.002) were more likely to stop driving but those with unilateral glaucoma were not (OR 1.5 (95% Cl 0.7-2.9); p= 0.3) with one Japanese study reporting individuals with severe POAG in the better eye to have an approximately 11.5 times greater odds of driving cessation than persons without POAG.

| Author and Year | Study Design | Total Participants (exposure/control) | Mean Age | Country | VI Definition | Comparator | Outcome Measure (OR, RR, HR?) | Effect Measure (with 95% Cl) |
|---|---|---|---|---|---|---|---|---|
| **Included in Meta-analysis** | | | | | | | | |
| Edwards J et al., 2008 | Cross-sectional | 1656 (152/1504) | 72.95 | USA | Self-reported physician diagnosed | Participants without glaucoma | HR | 1.47 (0.98, 2.19); p=0.06 |
| Gilhotra JS et al., 2001 | Cross-sectional | 3654 (61/3593) | 65.9 | Australia | Self-reported and physician diagnosed | Participants without glaucoma | OR | 2.2 (1.3, 3.9) |
| **Included in Narrative Summaries Only – High Income Countries** | | | | | | | | |
| Adler G et al., 2004 | Cross-sectional | 199 (52/147) | 71.3 | USA | Open-or closed-angle glaucoma | Participants without glaucoma | X^2 (Chi Square) | Drivers with glaucoma were no more likely than controls to have made plans for driving cessation; p=0.49 |
| Edwards J et al., 2008 | Cross-sectional | 1656 (152/1504) | 72.95 | USA | Self-reported physician diagnosed | Participants without glaucoma | Prevalence (%) | 8.6% (125/1450) of current drivers had glaucoma compared to 13.9% (28/199) of non-drivers with glaucoma. |
| Gilhotra JS et al., 2001 | Cross-sectional | 3654 (61/3593) | 65.9 | Australia | Open-angled | Participants without glaucoma | Prevalence (%) | 2% (37/2379) of current drivers had glaucoma compared to 5% (24/451) of non-drivers with glaucoma. |

| | | | | | | | | |
|---|---|---|---|---|---|---|---|---|
| Goh Y et al., 2011 | Case Series | 77 (77/0) | 71.8 | UK | Physician diagnosed | Participants with glaucoma and other ocular pathologies | OR | At clinic presentation: 4.99 (1.2, 20.6)* Glaucoma patients with other ocular pathologies were more likely to fail the driving criteria and give up driving than patients with only glaucoma. |
| | | | | | | | | At last clinic visit: 4.37 (1.6, 11.8) Glaucoma patients with other ocular pathologies were more likely to fail the driving criteria and give up driving than patients with only glaucoma. |
| Kaleem MA et al., 2021 | Cross-sectional | 191 (191/0) | 77 | USA | Physician diagnosed | Drivers with glaucoma but with either better VA or CS. | Prevalence (%) and X^2 (Chi Square) | 78% of participants reported that they had stopped driving. |
| | | | | | | | | Participants with worse VA were more likely to stop driving ($p< 0.05$) |
| | | | | | | | | Participants with worse CS were more likely to stop driving ($p< 0.01$). |
| MacLeod K et al., 2014 | Cross-sectional | 1279 (67/1211) | age, no.: 55-64 years = 233, 65- | USA | Self-reported physician diagnosed | Ex-drivers without glaucoma | RR (risk ratio) | 1.3 |
| | | | | | | | Attributable Risk | 1.6 |

| | | | | | | | | |
|---|---|---|---|---|---|---|---|---|
| | | | 74 years = 499, 75+ years = 547 | | | | Prevalence (%) | 7.4% (6/79) of non-driving participants had glaucoma compared to 5.7% (5/79) who did not have glaucoma. |
| Marottoli RA et al., 1993 | Cross-sectional | 1331 (28/1303) | age, no.: 65-74 years = 484, 75-84 years = 105, 85+ years = 6 | USA | Self-reported physician diagnosed | Participants without glaucoma | Prevalence (%) | From the 28 participants who reported glaucoma at baseline (1983), 42.9% (12/28) stopped driving by 198 compared to 22.2% (125/564) of people who did not have glaucoma and who also stopped driving. |
| Naredo Turrado J et al., 2020 | Prospective cohort | 11670 (525/11144) | 62.4 | France | Self-reported physician diagnosed | Participants without glaucoma | HR | 1.6, p>0.05 |
| Ramulu P et al., 2009 | Cross-sectional | 1135 (138/997) | 79.7 | USA | Bilateral or unilateral | Participants without glaucoma | OR | Bilateral: 2.6 (1.4, 4.8)<br><br>Stopped driving for over 8 years (bilateral): 3 (1.4, 6.4)*<br><br>Stopped driving less than 2 years ago (bilateral): 3.6 (1.5, 5.8) |
| | | | | | | | | Unilateral: 1.5 (0.7, 2.9)<br><br>Stopped driving less than 2 years ago (unilateral): 2.4 (1, 6) |
| | | | | | | | Prevalence (%) | 40.6% (28/68) of all participants with |

| | | | | | | | | |
|---|---|---|---|---|---|---|---|---|
| | | | | | | | | bilateral glaucoma were not driving. 21.4% (15/70) of all with unilateral glaucoma were not driving. 15% (150/997) of all without glaucoma were not driving. |
| Takahashi A et al., 2018 | Prospective cohort | 359 (211/148) | 54 | Japan | Mild POAG at baseline | Participants without glaucoma | OR | No association found (data not shown) |
| | | | | | Moderate POAG in the better eye at the 3 year follow-up | | | 37.7 (3.7, 383.8) |
| | | | | | Severe POAG in the better eye at baseline | | | 11.52 (2.87, 46.35) |
| | | | | | Severe POAG in the better eye at 3-year follow-up | | | 52.8 (3.5, 797) |
| | | | | | | | Prevalence (%) | 5.3% (8/152) of those with mild glaucoma were no longer driving.<br><br>21% (7/33) of those with moderate/severe glaucoma were no longer driving.<br><br>A total of 8.1% (15/185) of all participants with glaucoma were not driving compared to 1/3% (1/80) of drivers without glaucoma who |

| | | | | | | | | |
|---|---|---|---|---|---|---|---|---|
| | | | | | | | | were also no longer driving. |
| Tam A et al., 2018 | Cross-sectional | 99 (99/0) | 71.5 | Canada | Glaucoma severity was defined by the visual field mean deviation (MD) in the better eye and classified into 2 groups: mild (MD >−6 dB) and moderate/severe (MD ≤−6 dB), corrected visual acuity in the better eye ≥20/50 | Mild/moderate glaucoma patients | Prevalence (%) | 33% (15/46) of mild/moderate glaucoma reported driving cessation compared to 8% (4/53) of mild glaucoma patients; p= 0.002 |
| vanLandingham et al., 2013 | Cross-sectional | 139 (81/58) | 70.1 | USA | Physician diagnosed | Glaucoma suspect controls | OR | 4 (1.1, 4.7); p=0.03 |
| | | | | | | | Prevalence (%) | 22.5% (18/81) of participants with glaucoma were no longer driving. |
| **Included in Narrative Summaries Only – Low Middle Income Countries** | | | | | | | | |
| Deshmukh AV et al., 2019 | Case Control | 150 (100/50) | 64.5 | India | Anderson criterion | Drivers without glaucoma | Prevalence (%) | 16% (16/100) of those with glaucoma has stopped driving. |

*unadjusted results

**Table 4b(ii)** All studies (n=4) on cataract and driving cessation, all suitable to be summarised narratively only

**Additional Narrative Summaries:**
One study with sex disaggregated analysis found male drivers to be 7.01 times more likely to stop driving compared to female drivers who only had a 3.67 odds of driving cessation. Only one study examined the impact of a diagnosis of wet AMD but did not find any significant associations.

| **Author and Year** | **Study Design** | **Total Participants (exposure/control)** | **Mean Age** | **Country** | **VI Definition** | **Comparator** | **Outcome Measure (OR, RR, HR?)** | **Effect Measure (with 95% CI)** |
|---|---|---|---|---|---|---|---|---|
| **Included in Narrative Summaries Only – High Income Countries** | | | | | | | | |
| MacLeod K et al., 2014 | Cross-sectional | 1279 (278/1001) | age, no.: 55-64 years = 233, 65-74 years = 499, 75+ years = 547 | USA | Self-reported physician diagnosed | Ex-drivers without cataract. | RR (risk ratio) | 1.5 |
| | | | | | | | Attributable risk | 10.5, p<0.1 |
| | | | | | | | Prevalence (%) | 8% (6/79) of participants with cataracts no longer drove compared to 5.2% (4/79) with no cataracts. |
| Marottoli RA et al., 1993 | Cross-sectional | 1331 (105/1226) | age, no.: 65-74 years = 484, 75-84 years = 105, 85+ years = 6 | USA | Self-reported physician diagnosed | Current Drivers | OR | 2.29 (1.28, 4.1) |
| | | | | | | | Prevalence (%) | 45.7% (48/105) of participant with cataracts were no longer driving compared to 18.4% (90/488) of those who were no longer driving and did not have cataracts. |
| Naredo Turrado J et al., 2020 | Prospective cohort | 11670 (291/11379) | 62.4 | France | Self-reported physician diagnosed | Current drivers | HR | 1.79, p>0.05 |
| Sengupta S et al., 2014 | Cross-sectional | 122 (N/A) | 72.4 | USA | Physician diagnosed | Participants without cataract/PCSO in better eye. | PR (Prevalence Ratio) | Presence of cataract/PCO in the better seeing eye did not show any significant association |

| | | | | | | | | |
|---|---|---|---|---|---|---|---|---|
| | | | | | | | | with driving cessation; p>0.5 |

*unadjusted results

**Table 4b(iii)** All studies (n=5) on AMD and driving cessation with meta-analysis suitable for 3 studies

| Author and Year | Study Design | Total Participants (exposure/control) | Mean Age | Country | VI Definition | Comparator | Outcome Measure (OR, RR, HR?) | Effect Measure (with 95% CI) |
|---|---|---|---|---|---|---|---|---|
| **Included in Meta-analysis** | | | | | | | | |
| Campbell MK et al., 1993 | Case Control | 1656 (276/1380) | N/A | USA | Self-reported physician diagnosed | Current drivers | OR | 4.25 (2.6, 7); p<0.001 |
| Edwards J et al., 2008 | Cross-sectional | 1656 (89/1567) | 72.95 | USA | Self-reported physician diagnosed | Current drivers | HR | 1.46 (0.91, 2.36); p=0.12 |
| Stewart RB et al., 1993 | Cross-sectional | 1470 (N/A) | 78.1 | USA | Self-reported physician diagnosed | Current drivers | OR | 3.32 (1.91, 5.77); p=0.0001 |
| **Included in Narrative Summaries Only – High Income Countries** | | | | | | | | |
| Campbell MK et al., 1993 | Case Control | 1656 (276/1380) | N/A | USA | Self-reported physician diagnosed | Current drivers | OR | **Male**: 7.01 (3.1, 15.9); p<0.001)* |
| | | | | | | | | **Female**: 3.67 (2.0, 6.8), p<0.001* |
| | | | | | | | Prevalence (%) | 5.06% (70/1379) of participants still driving had AMD compared to 17.88% (50/277) of non-drivers with AMD. |
| Edwards J et al., 2008 | Cross-sectional | 1656 (89/1567) | 72.95 | USA | Self-reported physician diagnosed | Current drivers | Prevalence (%) | 4.9% (71/1457) of participant still driving had AMD compared to 9.5% (19/198) of non-driving participant with AMD. |

| | | | | | | | | |
|---|---|---|---|---|---|---|---|---|
| MacLeod K et al., 2014 | Cross-sectional | 1279 (48/1231) | age, no.: 55-64 years = 233, 65-74 years = 499, 75+ years = 547 | USA | Self-reported physician diagnosed | Ex-drivers without AMD. | RR (risk ratio) | 2.3 |
| | | | | | | | Attributable risk | 4.5, p<0.01 |
| | | | | | | | Prevalence (%) | 12.7% (10/79) of ex-drivers had AMD compared to 5.6% (4/79) of ex-drivers without AMD. |
| Sengupta S et al., 2014 | Cross-sectional | 122 (64/58) | 72.4 | USA | Physician reported wet AMD | Participants without AMD | OR | **Any eye:** 1.9 (0.5, 7.3) |
| | | | | | | | | **Worse eye:** 0.6 (0.1, 3.3) |
| | | | | | | | | **Better eye:** 2.7 (0.6, 11.5) |
| | | | | | | | Prevalence (%) | 74.6% (48/64) of participant with AMD were still driving compared. |
| | | | | | | | | More participants in the AMD group (25.4%) had stopped driving compared to those without AMD (6.9%); p= 0.006 |
| Stewart RB et al., 1993 | Cross-sectional | 1470 (N/A) | 78.1 | USA | Self-reported physician diagnosed | Current drivers | Prevalence (%) | 59.8% (35/58) of participant with AMD were still driving. |

*unadjusted results

**Table 4b(iv)** All studies (n=18) on visual acuity (VA) impairment and driving cessation, all suitable to only be summarised narratively due to their different VA cut-off points and comparators

| Author and Year | Study Design | Total Participants (exposure/control) | Mean Age | Country | VI Definition | Comparator | Outcome Measure (OR, RR, HR?) | Effect Measure (with 95% CI) |
|---|---|---|---|---|---|---|---|---|
| **Included in Narrative Summaries Only – High Income Countries** | | | | | | | | |
| Anstey K et al., 2006 | Prospective Cohort | 1466 (446/1020) | age no.: 70-74 years = 378, 75-79 years = 353, 80-84 years = 339, 85+ years = 396 | Australia | Corrected distance VA at 3 metres in best eye at 6/12 or worse | Participants with VA better than 6/12 (i.e. better than 20/40) | OR | Visit 2: 1.91 (0.51, 7.13) |
| | | | | | | | | Visit 3: 1.84 (0.68, 4.99) |
| | | | | | | | | Visit 4: 1.15 (0.55, 2.41) |
| DeCarlo D et al., 2003 | Cross-sectional | 126 (N/A) | 79 | USA | Better eye | Current drivers | Mean (SD) | VA in the better eye was worse in non-drivers (1.03 +/- 0.39) than drivers (0.74 +/- 0.34). |
| | | | | | Worse eye | | | VA in the worse eye was worse in non-drivers (1.58 +/- 0.43) than drivers (1.18 +/- 0.42). |

| | | | | | | | | |
|---|---|---|---|---|---|---|---|---|
| Edwards J et al., 2008 | Cross-sectional | 1656 (N/A) | 72.95 | USA | ETDRS chart with scores assigned from 0 to 90 (e.g. score of 0 = Snellen score of 20/125, score of 90 = Snellen score of 20/16) | Current drivers | HR (multivariate model) | 0.91 (0.791, 1.046); p=0.184 |
| | | | | | | | HR (cox regression) | 0.69 (0.61, 0.78); p<0.001 |
| Freeman E et al., 2005 | Prospective cohort | 1824 (263/1561) | 73.4 | USA | ≥ 0.1 and <0.3 logMAR at baseline | <0.1 logMAR as baseline | HR | 1.27 (0.96, 1.69) |
| | | 1824 (63/1498) | | | ≥0.3 logMAR VA at baseline | <0.1 logMAR as baseline | | 1.23 (0.69, 2.18) |
| | | 1824 (329/1495) | | | 1-2 lines VA loss | <1 line loss in VA | | 1.25 (0.96, 1.65) |
| | | 1824 (134/1690) | | | >2 lines VA loss | <1 line loss in VA | | 1.26 (0.87, 1.84) |
| Garre-Olmo J et al., 2009 | Cross-sectional | 875 (N/A) | 81.7 | Spain | Self-reported | Drivers without impaired VA | OR | 0.379 (0.201, 0.714); p=0.003* |
| Gilhotra JS et al., 2001 | Cross-sectional | 3654 (80/3574) | 65.9 | Australia | BCVA worse than 6/12 in the better eye | Current drivers | OR | 4 (2.5, 3.9) |
| | | 3654 (283/3371) | | | Presenting VA worse than 6/12 in the better eye | | | 2.5 (1.9, 3.4) |
| | | | | | | | Prevalence (%) | 11% (49/452) of participants have stopped driving have VA >20/40 compared to the 1% (21/2379) who are still driving with VA >20/40. |
| Huisingh C et al., 2016 | Prospective Cohort | 1995 (161/1834) | 77.2 | USA | logMar <0.3 | Drivers without VA impairment | HR | 0.83 (0.49, 1.42) |
| | | | | | | | Mean (SD) | VA of those who stopped driving (0.097 [0.15]) compared to those still driving (0.051 [0.13]). |

| | | | | | | | | |
|---|---|---|---|---|---|---|---|---|
| | | | | | | | Prevalence (%) | 90.9% (149/164) of those not driving had a VA of ≤20/40 compared to 9.2% (15/164) who stopped driving with a VA of >20/40. |
| Janz N et al., 2009 | Prospective Cohort | 607 (N/A) | age, no.: 25-49 years = 131, 50-64 years = 240, 65-74 years = 177 | USA | Better eye at 6 months | Driving vs. non-drivers | 2-sample t-test | Mean (SD) of VA in drivers (87.7 [4.9]) vs. non-drivers (85.1 [5.4]); p<0.001 |
| | | | | | | | Linear regression | Mean (SD) of VA in drivers (87.7 [4.9]) vs. non-drivers (85.1 [5.4]); p=0.012 |
| | | | | | Better eye at 54 months | | 2-sample t-test | Mean (SD) of VA in drivers (86.9 [5.7]) vs. Non-drivers (83.2 [6.9]); p= 0.025 |
| | | | | | | | Linear regression | Mean (SD) of VA in drivers (86.9 [5.7]) vs. Non-drivers (83.2 [6.9]); p=0.458 |
| | | | | | Mean (SD) difference in VA in better eye from 6 months to 54 months | Remained drivers vs. became non-drivers | Linear regression | Changes in Mean (SD) in VA of drivers (-0.4[0.6]) vs. became non-drivers (3.9[0.7]); p=0.001 |
| | | | | | Worse eye at 6 months | Driving vs. non-drivers | 2-sample t-test | Mean (SD) of VA in drivers (83.2 [7.5]) vs. non-drivers (79.7 [11.0]); p= 0.007 |
| | | | | | | | Linear regression | Mean (SD) of VA in drivers (83.2 [7.5]) vs. non-drivers (79.7 [11.0]); |

| | | | | | | | | |
|---|---|---|---|---|---|---|---|---|
| | | | | | | | | p= 0.095 |
| | | | | | Worse eye at 54 months | | 2-sample t-test | Mean (SD) of VA in drivers (81.5 [10.6]) vs. non-drivers (75.3 [14.4]); p=0.001 |
| | | | | | | | Linear regression | Mean (SD) of VA in driver (81.5 [10.6]) vs. non-driver: 75.3 (14.4); p=0.003 |
| | | | | | Mean (SD) difference in VA in worse eye from 6 months to 54 months | Remained drivers vs. became non-drivers | Linear regression | Mean (SD) of VA in drivers (1.4[1.3]) vs. became non-drivers: -5.5(2.1); p=0.054 |
| Keay L et al., 2009 | Prospective Study | 1425 (N/A) | 75 | USA | LogMAR scale | Whole population | Mean (SD) | mean(SD) of VA statistically significant different between those who stopped driving 0.08 (0.014) and those who continued driving -0.01(0.11); p=0.0006 |
| Keay et al., 2016 | Cross-sectional | 442 (N/A) | 73 | Australia | High contrast vision | Current drivers with cataracts NOTE: all participants had cataracts | OR | 1.21 (1.07, 1.37) |
| | | | | | Binocular | | X^2 (Chi Square) | p<0.001 |
| | | | | | Better eye | | | p<0.001 |
| | | | | | Worse Eye | | | p<0.001 |
| Levecq L et al., 2013 | Cross-sectional | 1000 (N/A) | 71.3 | Belgium | Physician diagnosed binocular VA worse than 20/40 | Current drivers | X^2 (Chi Square) | Right eye: Mean VA of current drivers (0.31) was significantly better than those who gave up driving due to vision (0.25); p=0.016 |
| | | | | | | | | Left eye: Mean VA in current drivers (0.31) was significantly |

| | | | | | | | | |
|---|---|---|---|---|---|---|---|---|
| | | | | | | | | better than those who gave up driving due to vision (0.24); p=0.004 |
| | | | | | | | | Both eyes:<br>Mean VA in current drivers (0.36)<br>Was significantly better than those who gave up driving due to vision (0.31); p=0.031 |
| Ramulu P et al., 2009 | Cross-sectional | 1135 (N/A) | 79.7 | USA | Binocular acuity 0.1 logMAR or worse in better eye. | Drivers without 0.1 logMAR binocular. | OR | 1.5, p<0.001 |
| Ross L et al., 2009 | Cross-sectional | 5206 (1062/4144) | 76.3 | Australia | Physician diagnosed with participants categorised into having a VA LogMAR 0.3 or better, or worse than LogMar 0.3. | Participants with normal vision (logMAR of or better than 0.3). | OR | 2.08 (2.56, 1.69)* |
| Rubin G et al., 2007 | Prospective Cohort | 2520 (N/A) | age, no.: 65-69 years = 780, 70-74 years = 829, 77-79 years = 553, 80-85 years = 350 | USA | 15 letter loss (logMAR 0.3) | Current Drivers | Prevalence (%) | Of those no longer driving: 84% (604/719) had VA ≤ 20/40 whilst 16% (115/719) had VA > 20/40. |

| | | | | | | | | |
|---|---|---|---|---|---|---|---|---|
| Segal-Gidan F et al., 2010 | Cross-sectional | 421 (44/377) | 72 | USA | Mild vision impairment was defined at the BCVA in the better eye (20/40-20/63) | Current drivers | OR | 5.53 (1.45, 20.98) |
| | | 421 (23/377) | | | Moderate/severe vision impairment was defined as BCVA in the better eye (20/80 or worse) | | | 13.23 (1.45, 120.3) |
| Sengupta S et al., 2014 | Cross-sectional | 122 (N/A) | 72.4 | USA | Worse VA in the better eye (1 line loss of vision) | 1 line worse in better eye acuity (logMAR) in all participants | OR | Low VA in either eye:<br>1.4 (1.1, 1.9); p<0.001<br>Low VA in better eye: 1.5 (1.2, 1.9); p<0.001 |
| | | | | | | | Mean (SD) | Participants who had stopped<br>driving (logMAR VA 0.77) had significantly worse vision in the better seeing eye than those still driving<br>(LogMAR VA 0.08); p=0.001 |
| Tam A et al., 2018 | Cross-sectional | 99 (N/A) | 71.5 | Canada | Physician diagnosed | N/A – looked at VA in whole population (all glaucoma patients) | X^2 (Chi Square) | Best corrected VA not associated with cessation; p=0.18 |
| | | | | | | | | Declines in central vision was significantly associated with driving cessation; p= 0.001 |
| | | | | | | | | Declines in near vision was significantly associated with driving cessation; p= 0.001 |
| | | | | | | | | Declines in peripheral vision was significantly associated |

| | | | | | | | | |
|---|---|---|---|---|---|---|---|---|
| | | | | | | | | with driving cessation; p= 0.001 |
| vanLandingham S et al., 2013 | Cross-sectional | 139 (N/A) | 70.1 | USA | 1 line worse in the better eye | Glaucoma suspect controls | OR | 1.3 (1, 1.8); p<0.05 |
| | | | | | | Moderate VA loss in glaucoma cases | | Severe VA loss: 1.5 (1.2, 1.8); p< 0.05 |

*unadjusted results

**Table 4b(v)** All studies (n=8) on contrast sensitivity (CS) impairment and driving cessation with 3 studies suitable for meta-analysis

**Additional Narrative Summaries:**
CS was measured either as a continuous measure, or categorised as "poor" according to normative cut-points, with one study using both measures.

| Author and Year | Study Design | Total Participants (exposure/control) | Mean Age | Country | VI Definition | Comparator | Outcome Measure (OR, RR, HR?) | Effect Measure (with 95% CI) |
|---|---|---|---|---|---|---|---|---|
| **Included in Meta-analysis** | | | | | | | | |
| Huisingh C et al., 2016 | Prospective cohort | 1995 (130/1865) | 77.2 | USA | <1.5 score on Pelli-Robson chart | Drivers with no bilateral CS impairment | HR | 1.73 (1.1, 2.72) |
| | | | | | | | Mean (SD) | The mean log CS of current drivers was 1.68 (0.13) compared to 1.61 (0.16) in non-drivers. |
| | | | | | | | Prevalence (%) | 5.8% (106/1831) of current drivers had a log CS <1.5, compared to 14.6% (24/164) who stopped driving. |
| Keay L et al., 2009 | Prospective cohort | 1425 (N/A) | 75 | USA | Per letter lost | Drivers with no bilateral CS impairment | OR | 1.15 (1.03, 1.28)* |
| | | | | | Better eye CS | | Mean (SD) | CS in better eye of those who stopped driving 32.4(4.1) significantly different between those who continued driving 35.3(2.2); p<0.001 |
| | | 122 (N/A) | 72.4 | USA | | | OR | 1.36 (1.1, 1.7); p<0.05 |

| | | | | | | | | |
|---|---|---|---|---|---|---|---|---|
| Sengupta S et al., 2014 | Cross-sectional | | | | Binocular CS 1 letter worse | Drivers with no bilateral CS impairment | | |
| | | | | | | | X^2 (Chi Square) | Those who stopped driving had significantly worse CS (log CS 1.8) compared to those still driving (log CS 1.2); p=0.03 |
| **Included in Narrative Summaries Only – High Income Countries** | | | | | | | | |
| Freeman E et al., 2005 | Prospective cohort | 1824 (725/1099) | 73.4 | USA | >=32 and <36 letters CS at baseline | Baseline CS equal to or more than 36 letters. | HR | 1.26 (0.97, 1.63) |
| | | 1824 (158/1666) | | | <32 letters at baseline | | | 1.46 (0.98, 2.17) |
| | | 1824 (79/1725) | | | 5 letter CS loss in 2 years | Less than 5 letter CS loss | | 1.33 (0.8, 2.22) |
| | | 1824 (86/1738) | | | >= 6 letter CS loss in 2 years | | | 1.71 (1.01, 2.9) |
| Keay L et al., 2016 | Cross-sectional | 442 (N/A) | 73 | Australia | 0.12 log units drop in CS. | Cataract patients who are still driving | OR | 1.29 (1.11, 1.49) |
| | | | | | | | Prevalence (%) | 17% (45/263) of current drivers and 35% (37/110) of former drivers had a CS <1 log decrease by at follow-up; p< 0.001 |
| | | | | | | | Mean (SD) | The worse eye CS in current drivers was 1.27 (+/- 0.36) compared to 1.11 (+/- 0.41) in former drivers; p< 0.001 |
| Ramulu P et al., 2009 | Cross-sectional | 1135 (N/A) | 79.7 | USA | 5 letters worse in better eye | Current drivers without 5 letters worse in better eye CS. | OR | 3, p<0.001 |
| Rubin G et al., 2007 | Prospective Cohort | 2520 (N/A) | Age, no,: 65-69 years = | USA | Log CS ≥ 1.65 | Current drivers | Prevalence (%) | 49.1% (884/1801) participants had stopped driving. |

| | | | | | | | | |
|---|---|---|---|---|---|---|---|---|
| | | | 780, 70-74 years = 829, 77-79 years = 553, 80-85 years = 350 | | Log CS 1.35-1.65 | | | 54% (973/1801) had stopped driving. |
| | | | | | Log CS <1.35 | | | 96.9% (1745/1801) had stopped driving. |
| vanLandingham S et al., 2013 | Cross-sectional | 139 (N/A) | 70.1 | USA | Binocular CS 1 letter worse | Glaucoma suspect controls | OR | 1.3 (1.2, 1.4); p<0.05 |

*unadjusted results

**Table 4b(vi)** All studies (n=8) on visual field (VF) impairment and driving cessation, all suitable to only be summarised narratively due to their different VF cut-off points and comparators

| **Author and Year** | **Study Design** | **Total Participants (exposure/control)** | **Mean Age** | **Country** | **VI Definition** | **Comparator** | **Outcome Measure (OR, RR, HR?)** | **Effect Measure (with 95% CI)** |
|---|---|---|---|---|---|---|---|---|
| **Included in Narrative Summaries Only – High Income Countries** | | | | | | | | |
| Freeman E et al., 2005 | Prospective Cohort | 1824 (659/1165) | 73.4 | USA | >1 and <= 8 points of central visual field at baseline | Equal to or greater than 1 points missed at baseline central VF | HR | 1.34 (1.02, 1.76) |
| | | 1824 (174/1650) | | | >9 points of central visual field at baseline | Equal to or greater than 1 points missed at baseline central VF | | 1.81 (1.23, 2.66) |
| | | 1824 (65/1759) | | | 5-7 points of central visual field loss in 2 years | <5 central VF loss | | 1.01 (0.6, 1.72) |

| | | | | | | | | |
|---|---|---|---|---|---|---|---|---|
| | | 1824 (92/1732) | | | >=8 points of central visual field loss in 2 years | <5 central VF loss | | 0.83 (0.53, 1.29) |
| | | 1824 (632/1192) | | | >9 and <=18 points of peripheral visual field at baseline | Less than or equal to 9 points missed at baseline peripheral VF | | 1.51 (1.14, 1.98) |
| | | 1824 (180/1644) | | | >18 points of peripheral visual field at baseline | Less than or equal to 9 points missed at baseline peripheral VF | | 1.73 (1.14, 1.98) |
| | | 1824 (106/1718) | | | 6-7 points of peripheral visual field loss in 2 years | <6 points loss of peripheral VF | | 1.04 (0.65, 1.65) |
| | | 1824 (88/1736) | | | >= 8 points of peripheral visual field loss in 2 years | <6 points loss of peripheral VF | | 1.91 (1.23, 2.96) |
| Huisingh C et al., 2016 | Prospective cohort | 1995 (493/1502) | 77.2 | USA | sensitivity <=22.5 dB | Participants without VF impairment | HR | 1.78 (1.29, 2.46) |
| Janz N et al., 2009 | Prospective cohort | 607 (N/A) | age, no.: 25-49 years = 131, 50-64 years = 240, 65-74 years = 187 | USA | Better eye at 6 months | Drivers vs. non-drivers | 2-sample t-test | Mean (SD) MD of drivers (-2.1 [2.7]), vs. non-drivers (-2.9 [3.0)]; p=0.014 |
| | | | | | | | Liner regression | Mean (SD) MD of drivers (-2.1 [2.7]) vs. non-drivers (-2.9 [3.0]); p=0.966 |
| | | | | | Better eye at 54 months | | 2-sample t-test | Mean (SD) MD of drivers (-1.9[3.1]) vs. non-drivers (-3.5 [3.7]); p<0.001 |

| | | | | | | | | |
|---|---|---|---|---|---|---|---|---|
| | | | | | | | Linear regression | Mean (SD) MD of drivers (-1.9[3.1]) vs. non-driver: -(3.5[3.7]); p= 0.007 |
| | | | | | Mean (SD) difference in VA in better eye from 6 months to 54 months | Remain drivers vs. became non-drivers | Linear regression | Difference in mean (SD) MD of drivers (0.2 [2.1]) vs. became non-drivers (-0.7 [2.7]); p=0.008 |
| | | | | | Worse eye at 6 months | Drivers vs. non-drivers | 2-sample t-test | Mean (SD) MD of drivers (-5.7 [4.9]) vs. non-drivers (-5.9 [4.0]); p=0.014 |
| | | | | | | | Liner regression | Mean (SD) MD of drivers (-5.7 [4.9]) vs. non-drivers (-5.9 [4.0]); p=0.429 |
| | | | | | Worse eye at 54 months | | 2-sample t-test | Mean (SD) MD of drivers (-5.4 [5.2]) vs. non-drivers (-7.0 [4.9]); p=0.012 |
| | | | | | | | Linear regression | Mean (SD) MD of drivers (-5.4 [5.2]) vs. non-drivers (-7.0 [4.9]); p=0.080 |
| | | | | | Mean (SD) difference in VA in worse eye from 6 months to 54 months | Remain drivers vs. became non-drivers | Linear regression | Difference in mean (SD) MD of drivers (0.3 [0.4]), vs. became non-drivers (-1.3 [0.7]); p= 0.013 |
| Keay L et al., 2009 | Prospective Cohort | 1425 (N/A) | 75 | USA | Bilateral VF points missing | Whole population | Mean (SD) | Mean(SD) of bilateral VF points missing was statistically |

| | | | | | | | | |
|---|---|---|---|---|---|---|---|---|
| | | | | | | | | significant different between those who stopped driving 9.8(17.1) and those who continued driving 1.98(5.1); p=0.001 |
| Keay L et al., 2016 | Cross-sectional | 442 (N/A) | 73 | Australia | Points missed on bilateral VF. | Current drivers with cataracts NOTE: all participants in this study had cataracts. | X^2 (Chi Square) | Median (IQR) of current drivers: 3 (0-10) vs. Median (IQR) of former drivers: 8 (1-19); p= 0.02 |
| Ramulu P et al., 2009 | Cross-sectional | 1135 (N/A) | 79.7 | USA | Bilateral VF damage in glaucoma participants | Participants without glaucoma | OR | 2 (1.6, 2.5) |
| | | | | | | | Prevalence (%) | 21% (14/68) of participants with bilateral VF loss in the lowest tertile (less than 3 dB of VF loss in better-eye) had stopped driving.<br><br>36% (24/68) of participants with VF loss in the middle tertile (better-eye VF mean deviation between −3 and −9 dB) had stopped driving.<br><br>52% (35/68) of participants with VF loss in the highest tertile (better eye VF mean deviation <-9 dB) had stopped driving. |
| Segal-Gidan F et al., 2010 | Cross-sectional | 421 (30/391) | 72 | USA | Unilateral | Current drivers | OR | 1.91 (0.63, 5.76) |
| | | 421 (108/318) | | | Bilateral, mild | | | 2.05 (0.74, 5.66) |

| | | | | | | | | |
|---|---|---|---|---|---|---|---|---|
| | | 421 (93/328) | | | Bilateral, moderate/severe | | | 2.84 (0.92, 8.78) |
| vanLandingham S et al., 2013 | Cross-sectional | 139 (N/A) | 70.1 | USA | 5 dB worse in the better eye | Glaucoma suspect controls | OR | 1.7 (1.1, 2.5); p= 0.008 |
| **NOTE:** There are a range of different study designs as well as cut-off points and areas of VF investigated in the identified studies. Due to methodological differences between each study, meta-analysis was limited and narrative reviews have been used instead to synthesise data. | | | | | | | | |

*unadjusted results

**Table 4b(vii)** All studies (n=3) on glare sensitivity (GS) impairment and driving cessation, all suitable to only be summarised narratively due to their different GS cut-off points and comparators

| Author and Year | Study Design | Total Participants (exposure/control) | Mean Age | Country | VI Definition | Comparator | Outcome Measure (OR, RR, HR?) | Effect Measure (with 95% CI) |
|---|---|---|---|---|---|---|---|---|
| **Included in Narrative Summaries Only – High Income Countries** | | | | | | | | |
| Freeman E et al., 2005 | Prospective Cohort | 1824 (702/1122) | 73.4 | USA | 3-4 points of glare sensitivity at baseline | ≤2 points difference with baseline glare GS | HR | 0.78 (0.61, 0.99) |
| | | 1824 (206/1618) | | | ≥5 points of glare sensitivity at baseline | | | 0.9 (0.63, 1.28) |
| | | 1824 (71/1753) | | | 4 points loss of glares sensitivity in 2 years | <4 points GS loss | | 1.18 (0.7, 1.99) |
| | | 1824 (52/1772) | | | ≥5 points loss of glare sensitivity in 2 years | <4 points GS loss | | 1.3 (0.72, 2.37) |
| Gilhotra JS et al., 2001 | Cross-sectional | 3654 (969/2685) | 65.9 | Australia | Physician diagnosed | Participants still driving | OR (logistic regression) | 1.5 (1.2, 1.8) |
| Tam A et al., 2018 | Cross-sectional | 99 (15/84) | 71.5 | Canada | Physician diagnosed | Still driving participants with cataracts | Prevalence ratio (PR) | 4.79; p<0.013 |

*unadjusted results

**Table 4b(viii)** All studies (n=11) on other types of vision impairment and driving cessation, all suitable to be summarised narratively only

| Author and Year | Study Design | Total Participants (exposure/control) | Mean Age | Country | Type of VI | VI Definition | Comparator | Outcome Measure (OR, RR, HR?) | Effect Measure (with 95% CI) |
|---|---|---|---|---|---|---|---|---|---|
| **Included in Narrative Summaries Only – High Income Countries** | | | | | | | | | |
| Campbell MK et al., 1993 | Case control | 1656 (28/1628) | N/A | USA | Retinal detachment | Self-reported physician diagnosed | Current drivers | Prevalence (%) | Still driving = 14.25%. Not driving = 40.95% Those not driving have a higher percentage of detached retina than those still driving (p<0.05) |
| | | | | | Retinal haemorrhage | Self-reported physician diagnosed | | OR | Both genders = 3.86 (1.4, 10.4)* |
| | | | | | | | | | Females: 4.70 (1.2, 17.8); p<0.5 |
| | | | | | Vision impairment | Self-reported | | Prevalence (%) | Still driving = 13.65%. Not driving = 25.34% Those not driving have a higher percentage of other visual loss than those still driving (p<0.01) |
| DeCarlo D et al., 2003 | Cross-sectional | 126 (126/0) | 79 | USA | Maculopathy | exudative or non-exudative | Current drivers | Prevalence (%) | The type of AMD (exudative vs nonexudative) was not significant between the non-drivers and drivers (p=0.474). Nonexudative non-drivers: 50% (48/96), nonexudative drivers : 47% (14/30), exudative non- |

| | | | | | | | | | |
|---|---|---|---|---|---|---|---|---|---|
| | | | | | | | | | drivers: 50% (48/96), exudative drivers 53% (16/30). |
| Hajek A et al., 2019 | Cross-sectional | 549 (192/357) | 90.3 | Germany | Vision impairment | Severe impairment | Current drivers | OR | 0.06 (0.01, 0.59)* |
| | | | | | | Mild impairment | | | 0.56 (0.24, 1.35)* |
| Gallo JJ et al., 1999 | Case Control | 1920 (N/A) | N/A | USA | Vision impairment | Self-reported | Current drivers | OR | 1.86 (0.7, 4.9) |
| Keay et al., 2016 | Cross-sectional | 442 (148/294) | 73 | Australia | URE | Measured with autorefraction and lensometry | Cataract patients who are still driving | X^2 (Chi Square) | No significant differences between current drivers with URE (40% [99/263]) and former drivers with URE (51% [49/110]); p= 0.07 |
| Levecq L et al., 2013 | Cross-sectional | 1000 (346/654) | 71.3 | Belgium | Vision impairment | Physician diagnosed | N/A | Prevalence (%) | Among the 190 non-drivers, 47 (24.7%) stopped driving because of their impaired vision. |
| Marottoli RA et al., 1993 | Cross-sectional | 1331 (17/1314) | age, no.: 65-74 years = 484, 75-84 years = 105, 85+ years = 6 | USA | Vision impairment | Self-reported | Current drivers | Prevalence (%) | Out of the 17 drivers who reported poor vision at baseline (1983), 58.8% (9/17) of drivers who stopped driving by 1989. |
| Moon SH et a., 2020 | Cross-sectional | 2970 (1023/1947) | 71 | South Korea | Vision impairment | Self-reported | Current drivers | OR | 0.97 (0.83, 1.14)* |
| Robinson JL et a., 2021 | Cross-sectional | 335 (N/A) | 67.4 | USA | Vision impairment | Self-reported | Current drivers | X^2 (Chi Square) | Participants were less likely to be driving if they had noted vision-related concerns (p<0.001). |

| | | | | | | | | | |
|---|---|---|---|---|---|---|---|---|---|
| Tam A et al., 2018 | Cross-sectional | 99 (19/80) | 71.5 | Canada | Dark adaptation in glaucoma patients | Self-reported | Among patients with glaucoma | X^2 (Chi Square) | Dark adaptation significantly associated with driving cessation (p<0.001) |
| | | | | | | | | PR (Prevalence Ratio) | 1.47; p= 0.39 Individuals with self-perceived dark adaptation difficulties were not more likely to quit driving. |
| Zebardast N et al., 2015 | Cross-sectional | 2469 (132/2337) | 73.5 | USA | URE | Binocular presenting visual acuity of 20/30 or worse, improving to better than 20/30 with subjective refraction | Participants with normal vision. | OR | 2.1 (1.3, 3.6) |
| | | | | | Non-refractive visual impairment | Post-refraction binocular BCVA of 20/30 or worse | | | 3.7 (2.4, 5.7) |

*unadjusted results

**Table 4b(ix)** All studies (n=2; reporting on 4 RCTs in total) evaluating anti-VEGF therapy and driving cessation, suitable for narrative summaries only

| Author and Year | Study Design | Intervention (n) | Control (n) | Mean Age | Country | Vision Impairment | VI Definition | Comparator(s) | Outcome Measure | Effect measure (with 95% where appropriate) |
|---|---|---|---|---|---|---|---|---|---|---|
| Bressler N et al., 2013 | RCT | 478 | 238 | 77.7 | USA | AMD | MARINA trial: minimally | Sham injections or 0.3 mg of Ranibizumab or 0.5 mg of | Prevalence (%) + 95% CI | Among patients who had reported driving at |

| | | | | | | | | | | |
|---|---|---|---|---|---|---|---|---|---|---|
| | | | | | | | classic or occult AMD | Ranibizuman for 24 months | | baseline, 74% (146/197) sham patients and 87.8% (156/178) 0.5mg patients reported still driving at 12 months. Among patients who had reported driving at baseline, 67.2% (131/195) (95% CI 59.2-75.2) of sham patients and 78.4% (148/189) (95% CI 71.8-85.0) of 0.5mg ranibizumab patients reported still driving 24 months later. |

| | | | | | | | | | | |
|---|---|---|---|---|---|---|---|---|---|---|
| Bressler N et al., 2013 | RCT | 280 | 143 | 77.7 | USA | AMD | ANCHOR: classic neovascular AMD | Verteporfin photodynamic therapy (PDT) or 0.3 mg ranibizumab injections or 0.5 mg ranibizumab injections for 24 months | Prevalence (%) + 95% CI | Among patients who reported driving at baseline, 80.5% (77/96) PDT patients and 94.2% (86/91) 0.5 mg patients reported still driving at 12 months. Among patients who reported driving at baseline, 71.6% (67/94) (95% CI 60.8-82.4) of PDT patients and 91.4% (81/89) (95% CI 85.3-97.5) of 0.5 mg ranibizumab patients reported still driving 24 months later. |

| | | | | | | | | | | |
|---|---|---|---|---|---|---|---|---|---|---|
| Bressler N et al., 2016 | RCT | 502 | 257 | 62.3 | USA | DME | RIDE/RISE: any DME | Sham injections or 0.3 mg ranibizumab or 0.5 mg ranibizumab | Prevalence (%) + 95% Cl | For 0.3 mg ranibizumab compared to those treated with sham only, there was a 7% (-5.0 to 19) difference in the number of participants now driving (who were not driving at baseline) at 12 months. For 0.5 mg ranibizumab compared to those treated with sham only, there was a 14.4% (1.1, 27.7) difference in the number of participants now driving at 12 months. For 0.3 mg ranibizumab |

| | | | | | | | | | | |
|---|---|---|---|---|---|---|---|---|---|---|
| | | | | | | | | | | compared to those treated with sham only, there was a 12.5% (-0.9, 25.9) difference in the number of participants now driving at 24 months. For 0.5 mg ranibizumab compared to those treated with sham only, there was a 14.3% (0.7, 27.9) difference in the number of participants now driving at 24 months. |

| | | | | | | | | | | |
|---|---|---|---|---|---|---|---|---|---|---|
| Bressler N et al., 2016 | RCT | 234 | 111 | 62.3 | USA | DME | RESTORE: DME in a least 1 eye eligible for laser treatment and a VA letter score between 78 and 39 | PDT laser only or 0.5 mg + laser or 0.5 mg only | Prevalence (%) with 95% CI | After 12 months, 12.2% (6/49) of those who were not driving at baseline and were treated with 0.5 mg ranibizumab + laser have started driving. Compared to those treated with laser only, there was a 4.2% (-7.7, 16.1) difference in the number of participants now driving at 12 months. After 12 months, 8.9% (4/45) of those who were not driving at baseline and were treated |

| | | | | | | | | | | |
|---|---|---|---|---|---|---|---|---|---|---|
| | | | | | | | | | | with 0.5 mg ranibizumab only have started driving. Compared to those treated with laser only, there was a 0.9% (-10.3, 12.1) difference in the number of participants now driving at 12 months. |

**Table 4b(x)** All studies (n=2) evaluating cataract surgery and driving cessation, suitable for narrative summaries only

| Author and Year | Study Design | Total Participants (exposure/control) | Mean Age | Country | VI Definition | Comparator | Outcome Measure (OR, RR, HR?) | Effect Measure (with 95% CI) |
|---|---|---|---|---|---|---|---|---|
| Monestam E et al., 2005 | Prospective Cohort | 810 (N/A) | 74.7 | Sweden | Physician diagnosed cataracts | All cataract surgery patients, comparing pre and post cataract surgery outcomes. | Prevalence (%) | Before cataract surgery, 55% (224/407) were drivers while after surgery 70% (285/407) were drivers. 5 years after surgery 63% (189/300) of patients with a driving licence were still active drivers. 37% (67/183) of patients who did not drive before surgery started to drive after. 46% (31/67) of patients who did not fulfil the visual requirements for presenting VA and the 35% (24/67) who did not fulfil the requirements for BCVA for a |

| | | | | | | | | |
|---|---|---|---|---|---|---|---|---|
| | | | | | | | | legal licence could now legally drive. 82% (40/50) of patents who began to drive after the surgery were still driving 5 years later. |
| Monestam E et al., 1997 | Prospective cohort | 211 (N/A) | 41 | Sweden | Physician diagnosed cataracts | Driving status from all participants pre- and post-surgery. | Ratio (%) | The number of patients driving after surgery increased to 65% (137/211) (from 56%), but this was not significant. |

**Table 4b(xi)** All studies (n=1) evaluating anti-glaucoma therapy and driving cessation

| **Author and Year** | **Study Design** | **Total Participants (exposure/control)** | **Mean Age** | **Country** | **Vision impairment** | **VI Definition** | **Comparator** | **Outcome Measure (OR, RR, HR?)** | **Effect Measure (with 95% Cl)** |
|---|---|---|---|---|---|---|---|---|---|
| Stafford WR, 1981 | Cross-sectional | 240 (N/A) | age, no.: 35-49 years = 11, 50-65 years = 77, >65 years = 139 | USA | Glaucoma | Chronic open-angle glaucoma or ocular hypertension that has been adequately controlled for at least | Post- anti-glaucoma therapy outcomes in all participants. | Prevalence (%) | From the 229 patients who stated that the anti-glaucoma therapy side effects affected their |

| | | | | | | | | | |
|---|---|---|---|---|---|---|---|---|---|
| | | | | | | the previous 6 months | | | normal activity, 12% (28/229) said that they had to give up some normal activity. Out of the 28 patients, 16 mentioned giving up driving, particularly at night. |
