# Supplementary material for: Associations between vision impairment and vision-related interventions on crash risk and driving cessation: systematic review and meta-analysis
Source: BMJ Open. 2023 Aug 11;13(8):e065210. doi: 10.1136/bmjopen-2022-065210 (PMC10423787; doi:10.1136/bmjopen-2022-065210)
Supplement: Supplementary data [file bmjopen-2022-065210supp005.pdf]

Appendix 5a Associations between MVC involvement and vision impairment and vision-related intervention

<table>
<tr><th>Vision impairment</th><th>Outcome</th><th>Association</th></tr>
<tr><td>Glaucoma</td><td>Any MVC</td><td>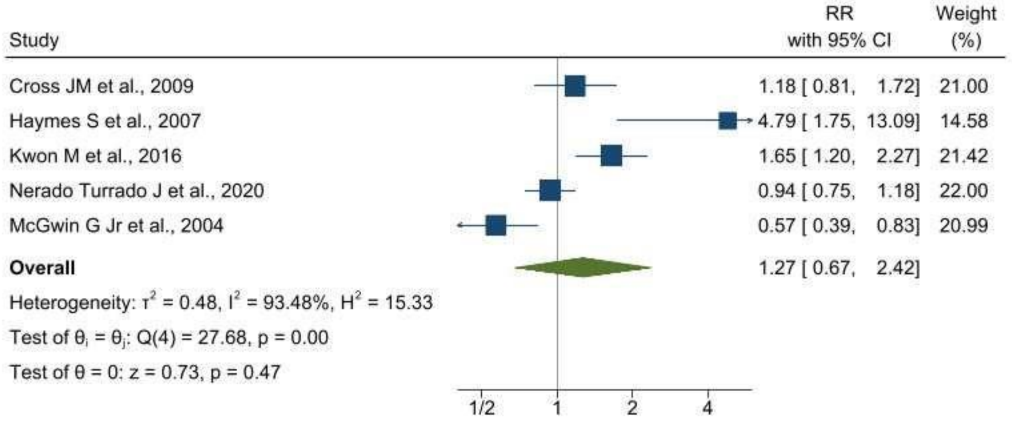
</td></tr>
<tr><td></td><td>At-fault MVC</td><td>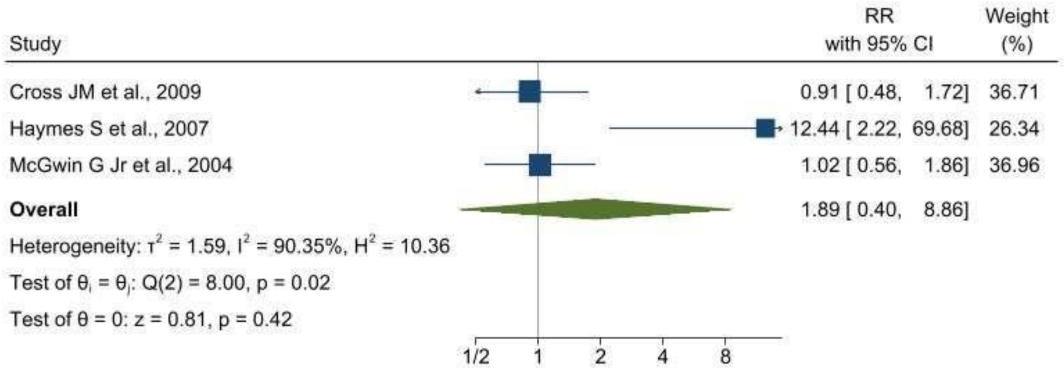
</td></tr>
</table>

<table>
<tr>
<td>Cataract</td>
<td>Any MVC</td>
<td>
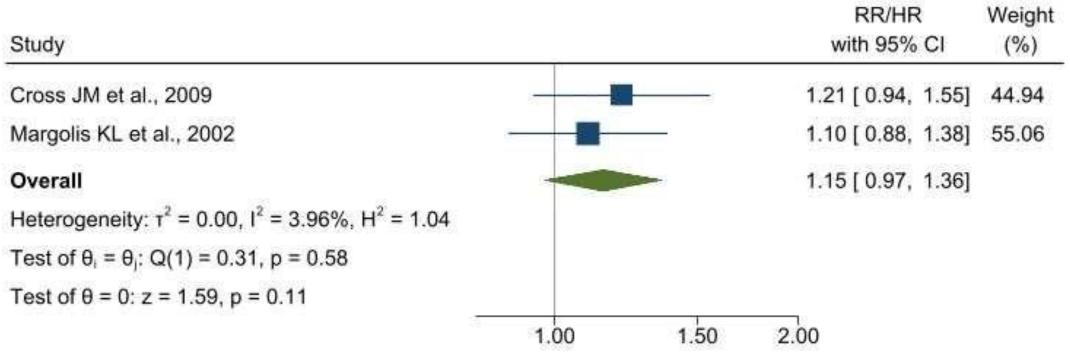

NOTE: POOLED ONE STUDY WITH RR AND ANOTHER WITH HR
</td>
</tr>
<tr>
<td>Stereopsis</td>
<td>Any MVC</td>
<td>
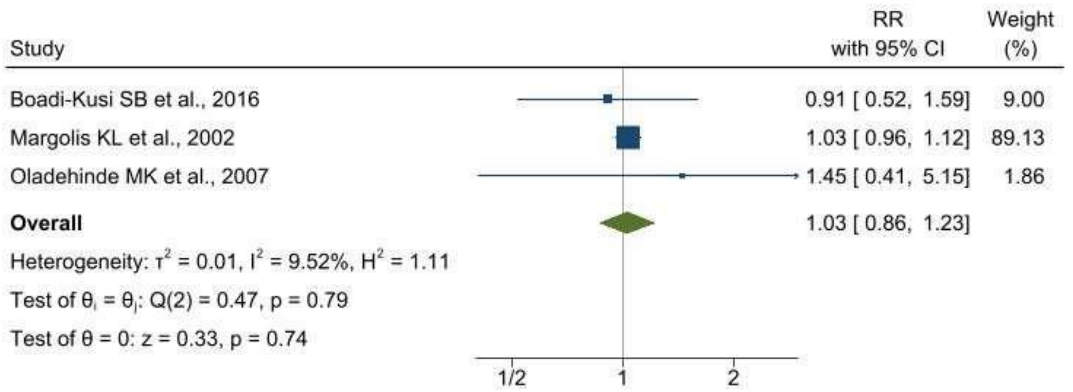

</td>
</tr>
</table>

<table>
<tr><td>Myopia</td><td>Any MVC</td><td>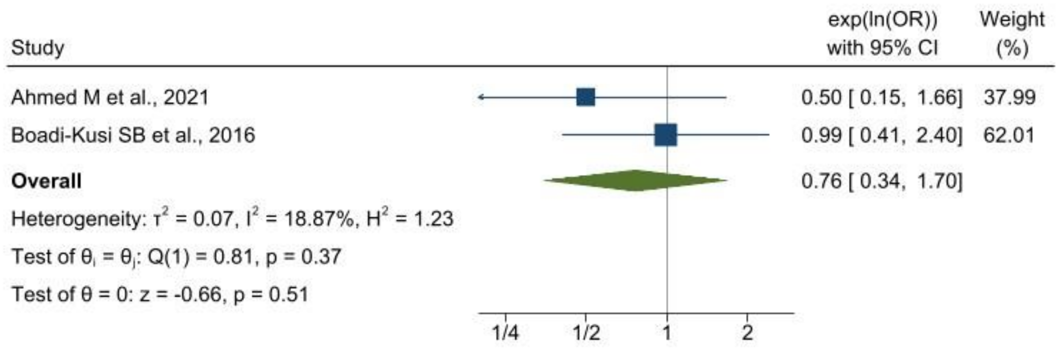
</td></tr>
</table>

<table>
<tr><td rowspan="2">VA ≤ 20/40</td><td>Any MVC</td><td>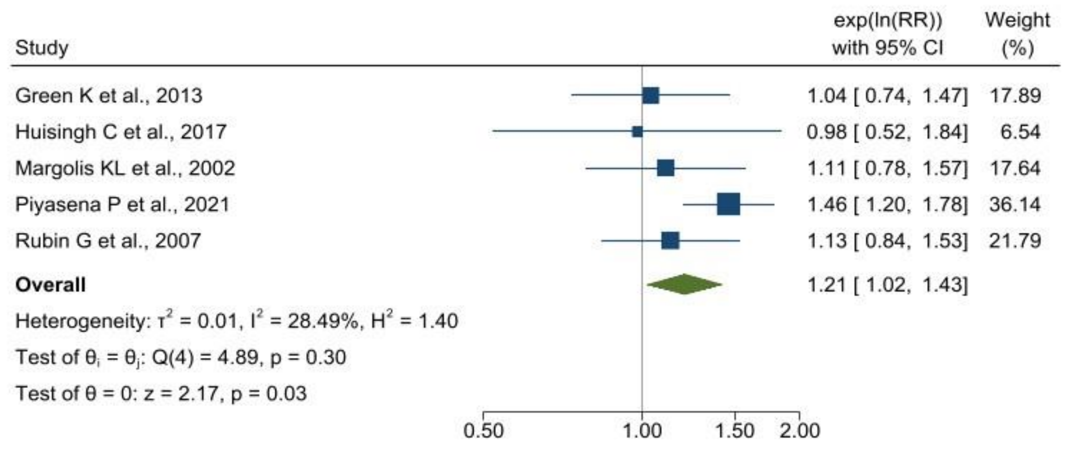
</td></tr>
<tr><td>At-fault MVC</td><td>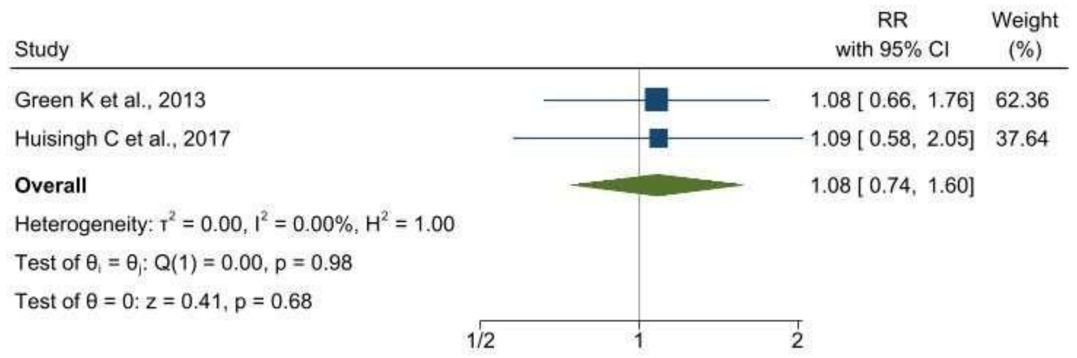
</td></tr>
</table>

<table>
<tr><td>Contrast Sensitivity Loss</td><td>Any MVC</td><td>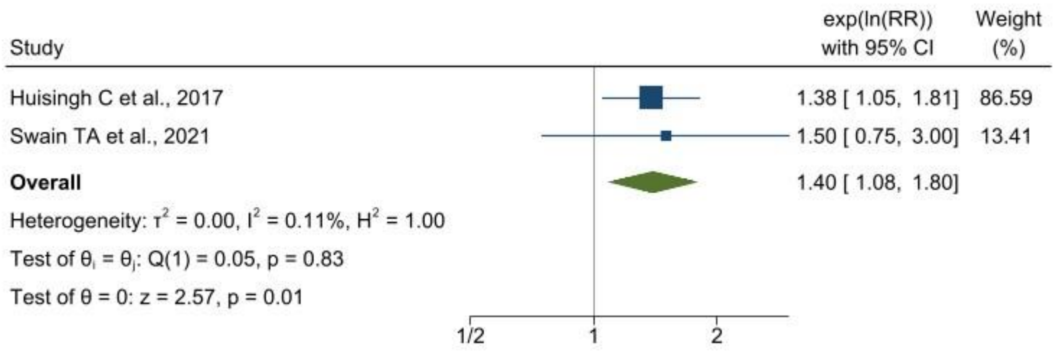
</td></tr>
<tr><td>Visual Field Loss</td><td>Any MVC</td><td>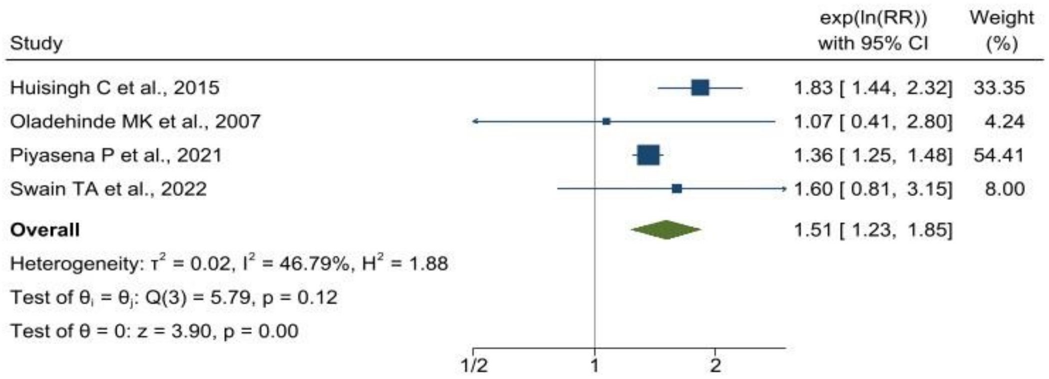
</td></tr>
<tr><td>Vision-related Intervention</td><td>Outcome</td><td>Association</td></tr>
</table>

**Cataract Surgery**	**Any MVC**	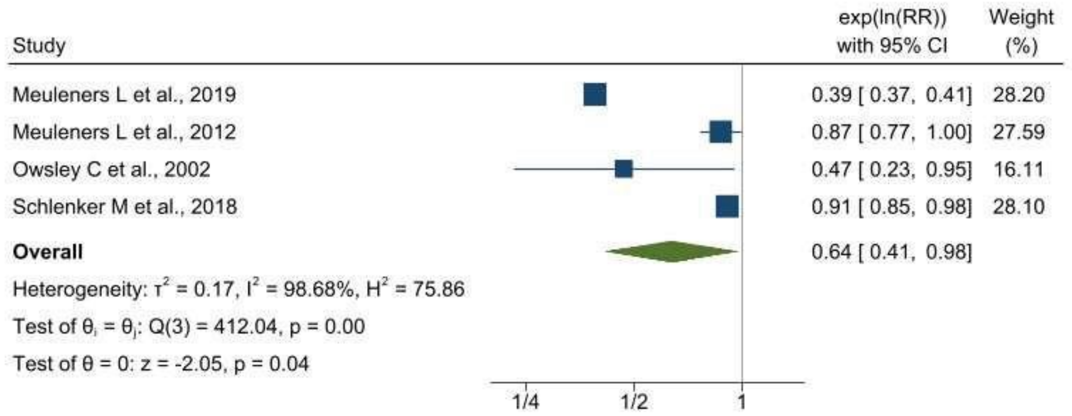<br><br>HOWEVER WHEN DROPPING SCHLENKER M ET AL., 2018 (UNADJUSTED EFFECT MEASURE):<br><br>**Overall** RR 0.55 [0.35, 0.92]<br>Heterogeneity: T^2 = 0.17, I^2 = 97.10%%, H^2 = 34.52<br>Test of $\theta_i = \theta_j$: Q(2) = 125.66, p = 0.00<br>Test of θ = 0: z = -2.30, p = 0.02

Appendix 5b Associations between driving cessation and vision impairments

**Vision impairment**	**Association**
**Glaucoma**	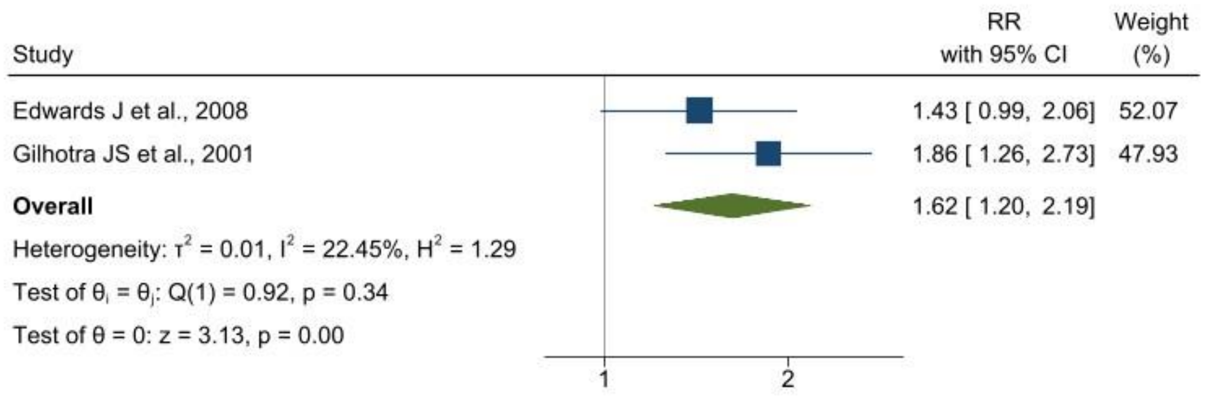
**AMD**	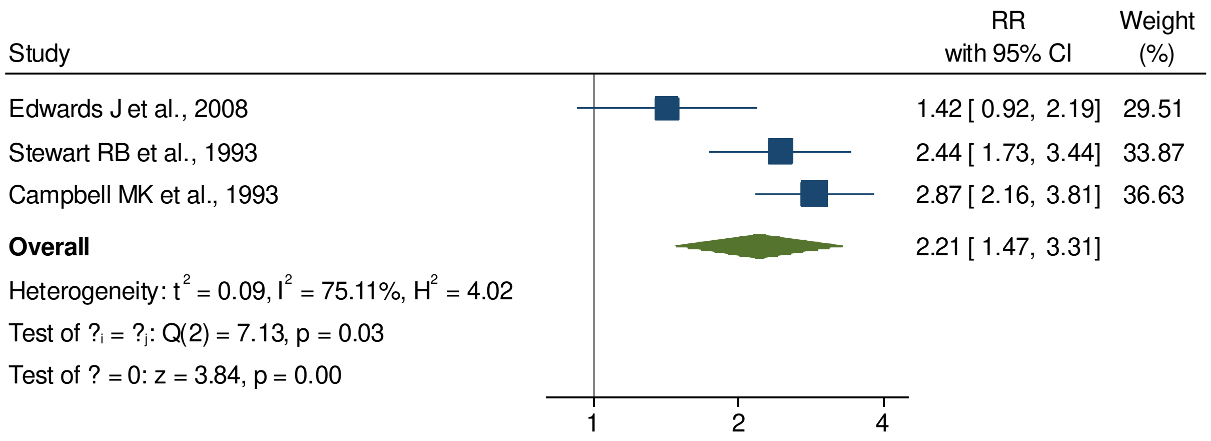
**Contrast Sensitivity**	

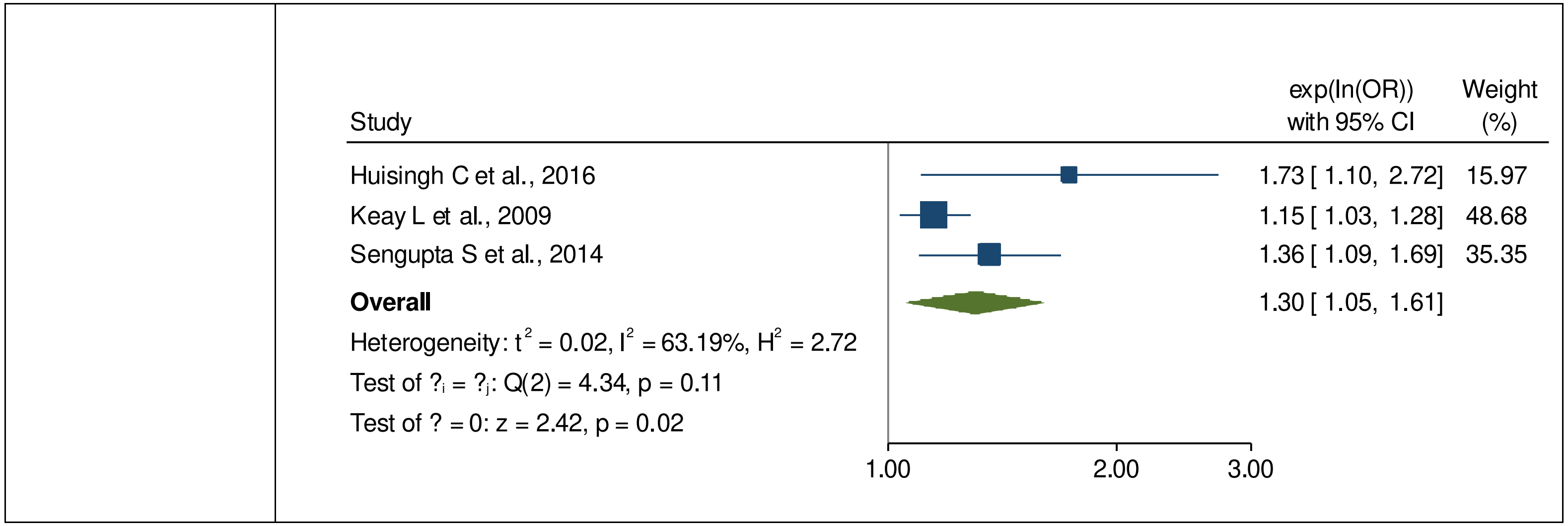
Study
exp(ln(OR)) with 95% CI
Weight (%)
Huisingh C et al., 2016
1.73 [1.10, 2.72]
15.97
Keay L et al., 2009
1.15 [1.03, 1.28]
48.68
Sengupta S et al., 2014
1.36 [1.09, 1.69]
35.35
Overall
1.30 [1.05, 1.61]
Heterogeneity: t^2 = 0.02, I^2 = 63.19%, H^2 = 2.72
Test of ?$_i$ = ?$_j$: Q(2) = 4.34, p = 0.11
Test of ? = 0: z = 2.42, p = 0.02
1.00
2.00
3.00
