# Supplementary material for: Associations between vision impairment and vision-related interventions on crash risk and driving cessation: systematic review and meta-analysis
Source: BMJ Open. 2023 Aug 11;13(8):e065210. doi: 10.1136/bmjopen-2022-065210 (PMC10423787; doi:10.1136/bmjopen-2022-065210)
Supplement: Author's manuscript [file bmjopen-2022-065210.draft_revisions.pdf]

BMJ Open

BMJ Open is committed to open peer review. As part of this commitment we make the peer review history of every article we publish publicly available.

When an article is published we post the peer reviewers' comments and the authors' responses online. We also post the versions of the paper that were used during peer review. These are the versions that the peer review comments apply to.

The versions of the paper that follow are the versions that were submitted during the peer review process. They are not the versions of record or the final published versions. They should not be cited or distributed as the published version of this manuscript.

BMJ Open is an open access journal and the full, final, typeset and author-corrected version of record of the manuscript is available on our site with no access controls, subscription charges or pay-per-view fees (http://bmjopen.bmj.com).

If you have any questions on BMJ Open's open peer review process please email info.bmjopen@bmj.com

BMJ Open

Associations between vision impairment and vision-related interventions on crash risk and driving cessation: systematic review and meta-analysis

Journal:	*BMJ Open*
Manuscript ID	bmjopen-2022-065210
Article Type:	Original research
Date Submitted by the Author:	01-Jun-2022
Complete List of Authors:	Nguyen, Helen; University of New South Wales,<br>Di Tanna, Gian Luca; George Institute for Global Health,<br>Coxon, Kristy; Western Sydney University - Campbelltown Campus, ; Translational Health Research Institute, Western Sydney University,<br>Brown, Julie; The George Institute for Global Health Injury Division<br>Ren, Kerrie; University of New South Wales<br>Ramke, Jacqueline; London School of Hygiene & Tropical Medicine,<br>Burton, Matthew J; London School of Hygiene and Tropical Medicine<br>Gordon, Iris; London School of Hygiene & Tropical Medicine<br>Zhang, Justine; London School of Hygiene and Tropical Medicine Faculty of Infectious and Tropical Diseases, International Centre for Eye Health, Clinical Research Department<br>Furtado, João; Universidade de São Paulo Faculdade de Medicina de Ribeirão Preto, Division of Ophthalmology<br>Mdala, Shaffi; Queen Elizabeth Central Hospital, Opthalmology Department<br>Kitema, Gatera Fiston; University of Rwanda College of Medicine and Health Sciences, Opthalmology Department<br>Keay, Lisa; University of New South Wales, School of Optometry and Vision Science; The George Institute for Global Health
Keywords:	PUBLIC HEALTH, OPHTHALMOLOGY, EPIDEMIOLOGY

SCHOLARONE™
Manuscripts

I, the Submitting Author has the right to grant and does grant on behalf of all authors of the Work (as defined in the below author licence), an exclusive licence and/or a non-exclusive licence for contributions from authors who are: i) UK Crown employees; ii) where BMJ has agreed a CC-BY licence shall apply, and/or iii) in accordance with the terms applicable for US Federal Government officers or employees acting as part of their official duties; on a worldwide, perpetual, irrevocable, royalty-free basis to BMJ Publishing Group Ltd ("BMJ") its licensees and where the relevant Journal is co-owned by BMJ to the co-owners of the Journal, to publish the Work in this journal and any other BMJ products and to exploit all rights, as set out in our licence.

The Submitting Author accepts and understands that any supply made under these terms is made by BMJ to the Submitting Author unless you are acting as an employee on behalf of your employer or a postgraduate student of an affiliated institution which is paying any applicable article publishing charge ("APC") for Open Access articles. Where the Submitting Author wishes to make the Work available on an Open Access basis (and intends to pay the relevant APC), the terms of reuse of such Open Access shall be governed by a Creative Commons licence – details of these licences and which Creative Commons licence will apply to this Work are set out in our licence referred to above.

Other than as permitted in any relevant BMJ Author's Self Archiving Policies, I confirm this Work has not been accepted for publication elsewhere, is not being considered for publication elsewhere and does not duplicate material already published. I confirm all authors consent to publication of this Work and authorise the granting of this licence.

For peer review only

Associations between vision impairment and vision-related interventions on crash risk and driving cessation: systematic review and meta-analysis

Authors: Helen Nguyen BMedSci (Hons)[1], Gian Luca Di Tanna PhD[2], Kristy Coxon PhD[3], Julie Brown PhD[2], Kerrie Ren MIPH[1], Jacqueline Ramke PhD[4,5], Matthew J Burton PhD[4,6], Iris Gordon MSc[4], Justine H Zhang MBBS[4,7], João M. Furtado PhD[8], Shaffi Mdala MSc [9], Gatera Fiston Kitema MScPHEC[10], Lisa Keay PhD[1,2]

Affiliations:

1. School of Optometry and Vision Science, Faculty of Science, The University of New South Wales, NSW, Australia

2. The George Institute for Global Health, Faculty of Medicine, University of New South Wales, NSW, Australia

3. School of Health Sciences, and the Translational Health Research Institute, Western Sydney University, NSW, Australia

4. International Centre for Eye Health, London School of Hygiene & Tropical Medicine, London, United Kingdom, WC1 E7HT

5. School of Optometry and Vision Science, University of Auckland, Auckland, New Zealand

6. National Institute for Health Research Biomedical Research Centre for Ophthalmology at Moorfields Eye Hospital NHS Foundation Trust and UCL Institute of Ophthalmology, London, UK

7. Royal Free Hospital, London, United Kingdom

8. Division of Ophthalmology, Ribeirão Preto Medical School, University of São Paulo, Ribeirão Preto, São Paulo, Brazil

9. Ophthalmology Department, Queen Elizabeth Central Hospital, Blantyre, Malawi

10. Ophthalmology Department, School of Health Sciences, University of Rwanda, Kigali, Rwanda

Corresponding Author: Lisa Keay

Postal Address: School of Optometry and Vision Science, The University of New South Wales, Sydney UNSW NSW 2052, Australia

Email: l.keay@unsw.edu.au

For peer review only

For peer review only - http://bmjopen.bmj.com/site/about/guidelines.xhtml

ABSTRACT

Objectives: To systematically investigate the associations between vision impairment and risk of motor vehicle crash (MVC) involvement, and evaluate vision-related interventions to reduce MVCs.

Design: Medline (Ovid), EMBASE and Global Health electronic databases were systematically searched from inception to March 2022 for observational and interventional English-language studies. Screening, data extraction and appraisals were completed by two reviewers independently. Where appropriate, measures of association were converted into risk ratios (RRs) or ORs (odd ratios) for meta-analysis.

Participants: Drivers of four-wheeled vehicle of all ages with no cognitive declines.

Primary and secondary outcomes: MVC involvement (primary) and driving cessation (secondary)

Results: 101 studies (n=778,052) were included after full-text review. 57 studies only involved older drivers (≥65 years) and 85 were in high-income settings. Visual field (four studies; RR 1.51 (95%Cl 1.23-1.85); p<0.001; I^2=46.79%), contrast sensitivity (two studies; RR 1.40 (95%Cl 1.08-1.80); p=0.01, I^2=0.11%), and visual acuity loss (5 studies; RR 1.21 (95%Cl 1.02, 1.43); p=0.03, I^2=28.49%) but not glaucoma (five studies, RR 1.27 (95%Cl 0.67-2.42); p=0.47; I^2=93.48%) or cataract (two studies RR 1.15 (95%Cl 0.97-1.36); p=0.11; I^2=3.96%) increased crash risk. Driving cessation was associated with glaucoma (two studies; RR 1.62 (95%Cl 1.20-2.19); p<0.001, I^2=22.45%), age-related macular degeneration (AMD) (three studies; RR 2.21 (95%Cl 1.47-3.31); p<0.001, I^2=75.11%) and reduced contrast sensitivity (three studies; RR 1.30 (95%Cl 1.05-1.61); p= 0.02; I^2=63.19%). Cataract surgery halved MVC risk (three studies; RR 0.55 (95%Cl 0.34-0.92); p= 0.02; I^2=97.10). Ranibizumab injections (four RCTs) prolonged driving in persons with AMD.

Conclusion: Impaired vision identified through a variety of measures is associated with both increased MVC involvement and cessation. Cataract surgery can reduce MVC risk. Despite literature being highly heterogeneous, this review shows that detection of vision problems and appropriate treatment is critical to road safety.

PROSPERO registration: CRD42020172153

For peer review only

Strengths and Limitations of this Study

- This is an up-to-date systematic review capturing literature on a variety of eye diseases and conditions, measures of vision such as visual acuity, contrast sensitivity, glare sensitivity, and visual field, and vision-related interventions and their associations on MVC involvement and driving cessation.
- There were no geographical or age restrictions placed on the population of focus allowing the global impact of vision impairment on driving to be documented for all age groups.
- Meta-analysis was limited due to heterogeneity in the outcome measures reported and the definitions of vision loss and or impairment used in each study. This heterogeneity also prohibited sub-group analyses by age and geographic location.
- Only statistical heterogeneity was assessed and not clinical or methodological.
- Publication bias was not assessed as there were less than ten studies included in each meta-analysis.

For peer review only

INTRODUCTION

Globalisation and economic development have made driving one of the main modes of transport worldwide and passenger vehicle travel is predicted to triple between 2015 and 2050.[(1)] Driving allows for independent mobility and enhances access to employment and education. Unfortunately, with more drivers on the roads, motor vehicle crashes (MVCs) and road traffic injuries are increasing worldwide. Approximately 1.35 million MVC-related fatalities occur each year with an additional 20-50 million people experiencing road-related injuries per annum.[(2)] The United Nations have therefore created targets within the Sustainable Development Goals (SDG) which aim to halve road deaths by 2020 (Target 2.6) and provide safe and sustainable transport systems for vulnerable road users (Target 11.2).[(3)]

Driving is a common and valued activity for many adults. Driving cessation limits independent mobility and has been linked to depressive symptoms and poorer health in older adults.[(4)] Functional declines in vision disproportionately impact older drivers, as they have higher prevalence of poor vision and eye diseases.[(5, 6)] Some countries have specific licensing requirements for older drivers,[(7)] however variations in visual driving standards across jurisdictions have made it difficult to assess whether these standards have safety benefits.[(8)]

This review is part of the *Lancet Global Health Commission* on Global Eye Health[(9)] and aimed to systematically evaluate the evidence to (1) investigate the associations between vision impairment and risk of MVC involvement across the lifespan, and (2) evaluate vision-related interventions to reduce MVCs. Since risks can be mitigated by driving retirement, this review also considered driving cessation as a secondary outcome.

METHODS

This systematic review was reported using PRISMA guidelines[(10)] (*Appendix 1*) using a published protocol.[(11)] An electronic database search on Medline (Ovid), EMBASE and Global Health was conducted from their inception to March 2020, and then updated in March 2022, with no geographic restrictions. *Appendix 2* details the search strategy with *Table* 1 describing the inclusion and exclusion criteria for studies.

For peer review only

Table 1 Study inclusion and exclusion criteria

Inclusion	Exclusion
• Interventional (RCTs) and observational (cohort, cross-sectional, case-control and case series) studies.<br>• Systematic reviews with meta-analyses<br>• Studies on drivers of four-wheeled motorised vehicles of all ages.<br>• Studies looking at the following exposures of interest: impairment in measures of vision (visual acuity (VA), contrast sensitivity (CS), visual field (VF), and glare sensitivity (GS)) or specific eye conditions including but not limited to glaucoma, cataracts, age-related macular degeneration (AMD), diabetic retinopathy (DR), stereopsis disorders and colour vision deficiencies.<br>• Studies on interventions such as vision screening, refractive correction, cataract surgery, anti-VEGF injections and other treatments to improve vision.	• Literature reviews and narrative systematic reviews.<br>• Commentary articles, dissertations, abstracts, editorials and conference presentations.<br>• Studies using simulators or investigated either self-regulatory driving behaviours (e.g. night driving avoidance), or self-reported measures of driving safety.<br>• To narrow the scope of the study, studies on populations with specific non-vision related medical conditions (e.g. dementia, epilepsy, stroke and history of medical events such as syncope), low vision or vision difficulties caused by other medical conditions (e.g. hemianopia caused by brain damage).

The population of focus was drivers of four-wheeled motorised vehicles, of all ages, with no cognitive declines. Exposures of interest included eye diseases (e.g. glaucoma, cataract, age-related macular degeneration, diabetic retinopathy) and conditions (e.g. refractive errors), and measures of vision such as, but not limited to, visual acuity and contrast sensitivity. Studies reporting on interventions focused on treatments that would improve vision. The primary outcome measure was MVC involvement identified from self-reported surveys or government/hospital administrative datasets. The secondary outcome was self-reported driving cessation. Due to the large volume of data collected, other surrogate measures of driving safety and driving performance planned in the original protocol were beyond the scope of this manuscript but will be reported in a separate systematic review.[11] Studies which used simulators, or investigated self-regulatory driving behaviours (e.g. night driving avoidance) through surveys were excluded.

All titles, abstracts, and full-texts were reviewed independently by two investigators using Covidence systematic review management software (Covidence non-profit SaaS Enterprise, Melbourne, Australia). All discrepancies were resolved via consultation with a third investigator. Similarly, data extraction was completed independently by two investigators using data extraction forms adapted from either the Joanna Briggs Institute (JBI) templates for observational and systematic review study designs, or Cochrane templates for interventional studies. Data extracted from the studies included design, participant and setting characteristics, exposure type and definition, intervention details (if any), outcome measures, and relevant effect measures.

Overall risk of bias for all included studies was assessed by two investigators independently with conflicts resolved by a third investigator. All quality assessments were conducted using the relevant

For peer review only - http://bmjopen.bmj.com/site/about/guidelines.xhtml

JBI critical appraisal tools.[12] Each question on the relevant tools were categorised into either selection, detection, confounding, validity, performance, attrition, or allocation bias by all authors. Each study was given an overall "score" for their bias depending on how each question was answered (Yes=1, No=0, Unsure or N/A=0). The final scores were used to assign each study as low, medium, or high risk of bias, with lower scores indicating higher risk of bias.

Statistical Analysis

Associations between vision impairments and vision-related interventions with MVC involvement and driving cessation were summarised with appropriate Hazard Ratios (HR), Risk Ratios (RR), or Odds Ratios (OR). Narrative summaries were reported using the SWiM guidelines.[13] Heterogeneity across studies were assessed using I^2 statistic. Meta-analysis was conducted by converting all effects measures into RR or OR. Random-effects meta-analysis was only conducted on studies which presented data with the same outcomes, exposures and comparators, and which reported on associations adjusted for confounders to reduce bias. Data from case-controls were not pooled for meta-analysis to minimise possible heterogeneity. No publication bias analysis was conducted as there were less than 10 studies in each meta-analysis. Reporting of the results were guided by the Meta-analysis of Observational Studies in Epidemiology (MOOSE) guidelines.[14]

Patient and Public Involvement

Only existing published literature were looked at in this review and therefore no patient or public involvement was present during the design or execution of the review. Public participation may be sought out for future dissemination of this review.

RESULTS

From the electronic database search, 5111 studies were identified after the removal of 2131 duplicates. After title and abstract screening, 243 studies remained for full-text review after which 142 studies were further excluded, leaving 101 studies for data extraction (*Figure 1*).

63 studies (31 cross-sectional, 19 cohort, 12 case-control and one systematic review with meta-analysis) reported on MVC involvement alone, 34 (21 cross-sectional, eight cohort, two case-control, one case series, and two RCTs) on driving cessation, and four (one cross-sectional, two cohort, and one case-control) on both MVC and cessation. When split by geographic regions, 48 studies from HICs and 15 studies from LMICs reported solely on MVC involvement, whilst all 34 studies looking at driving cessation only came from HICs. From the studies which reported on both MVC and driving cessation, only one was from a LMIC. Study breakdown according to driving outcome and vision impairment is shown in *Table 2(a-b)*. The majority of studies (84%) were set in HICs and 57 studies (56%) focused on older adults. However, when looking at the 16 studies set in LMICs, all but two had an average study population age of less than 65 years. From the total 101 studies, only 13 (seven from HICs, six from LMICs; 12 cross-sectional, one cohort) were categorised as high risk of bias with the rest rated as either low or medium (*Appendix 3*).

For peer review only - http://bmjopen.bmj.com/site/about/guidelines.xhtml

Table 2a Breakdown of studies reporting on vision-related associations by outcome measure

Driving Outcome	Vision Impairment	Region (HIC/LMIC)	Total No. of Studies
**Motor Vehicle Crash (MVC)**	Glaucoma	15 HICs; 1 LMIC	16
	Cataract	8 HICs	8
	AMD	6 HICs	6
	Diabetic Retinopathy	3 HICs	3
	Stereopsis Impairment	2 HICs; 3 LMICs	5
	Myopia	2 HICs; 2 LMICs	4
	Colour Blindness	1 HICs; 7 LMICs	8
	Contrast Sensitivity	13 HICs	13
	Visual Acuity	19 HICs; 9 LMICs	28
	Glare Sensitivity	3 HICs	3
	Visual Field Impairment	14 HICs; 6 LMICs	20
	Other†	13 HICs; 6 LMICs	19
**Driving Cessation**	Glaucoma	12 HICs; 1 LMIC	13
	Cataract	5 HICs	5
	AMD	5 HICs	5
	Contrast Sensitivity	8 HICs	8
	Visual Acuity	18 HICs	18
	Glare Sensitivity	3 HICs	3
	Visual Field Impairment	8 HICs	8
	Other‡	11 HICs	11

†unilateral vision impairment, general vision impairment, retinopathy, retinal detachment, poor visibility, refractive disorder, monocular vision impairment, hyperopia, amblyopia, diplopia, astigmatism, retinitis pigmentosa, stereoacuity

‡dark adaptation, age-related maculopathy, detached retina, non-refractive vision impairment, self-reported vision loss, retinal haemorrhage, uncorrected refractive error

For peer review only - http://bmjopen.bmj.com/site/about/guidelines.xhtml

2b Breakdown of studies reporting on a vision-related intervention by intervention type, vision impairment and outcome measure

Intervention	Vision Impairment	Driving Outcome	Region (HIC/LMIC)	No. of Studies
**Anti-VEGF Injections**	AMD	Driving Cessation	1 HIC	1
	Diabetic Macular Oedema (DME)		1 HIC	1
**Cataract Surgery**	Cataract	Motor vehicle crash	6 HICs	6
		Driving Cessation	2 HICs	2
**Corrective Lenses**	Refractive error	Motor vehicle crash	1 HICs	1
**Anti-glaucoma therapy**	Glaucoma	Driving Cessation	1 HICs	1

Raw data on studies reporting on MVCs[15-81] and driving cessation[70-73, 82-115] can be found in *Appendix 4a(i-xiv)* and *4b(i-ix),* respectively, with additional narrative summaries. Meta-analyses on associations are presented in *Figure 2a-b*; only 25 studies could be meta-analysed. *Figure 3* synthesises the narrative summaries and meta-analyses to show multiple associations of vision with MVCs and driving cessation. From *Figure 3* it can be seen that associations reported for eye diseases and measures of vision function were more consistent across studies looking at crashes compared to cessation. When considering vision-related interventions, only cataract surgery was shown to improve driving by minimising crash risk. The benefits of anti-VEGF injections on prolonging driving was more inconclusive and found to only help drivers with AMD but not diabetic macular oedema. However as a whole, the evidence from the literature on associations between vision impairment and crashes and cessation are mostly inconclusive and or mixed.

<u>Associations between eye diseases and conditions/measures of vision loss and motor vehicle crashes</u>

The results were mixed (16 studies, n=21,214 participants) for associations between glaucoma and MVCs.[24, 30, 38, 41, 43, 45, 46, 52, 54, 65, 67-72] As illustrated in *Figure 2a*, meta-analyses on five suitable studies,[24, 30, 38, 43, 72] found a glaucoma diagnosis did not significantly increase the risk of any MVC involvement (OR 1.27 (95% Cl 0.67- 2.42); p=0.47). Similarly, meta-analyses on three studies[24, 30, 43] looking at at-fault crashes also found no difference between drivers with and without glaucoma (RR 1.89 (95% Cl 0.40-8.86); p=0.42). Increased risk was evident with more severe glaucoma.[30, 38, 43, 46, 52, 65, 69, 70]

Out of the eight cataract studies (n=18,883) identified,[24, 40, 41, 45, 54, 56, 57, 72] most found self-reported, physician-diagnosed cataracts did not impact the likelihood of any type of MVC involvement, with meta-analysis showing no overall increased risk (*Figure 2a*; two studies; OR 1.15 (95% Cl 0.97-1.36); p=0.11).[24, 40] At-fault crash involvement was investigated by two studies however only one reported significant associations.[24, 56]

Meta-analysis could not be conducted on any studies looking at drivers with either AMD (five studies, n=4150)[24, 41, 44, 64, 66] or diabetic retinopathy (3 studies, n=4353),[24, 45, 54] however no studies found increased risk of MVC.

For peer review only - http://bmjopen.bmj.com/site/about/guidelines.xhtml

For peer review only

Impairments in stereopsis were not found to increase the risk of MVC involvement across the five studies identified (n=3253).[22, 33, 40, 51, 75] Meta-analysis on three studies showed no difference in crash involvement between those with and without stereopsis impairment (*Figure 2a*; RR 1.03 (95% CI 0.86-1.23); p=0.74).[22, 40, 51]

Summary of studies on myopia (four studies, n= 2039)[22, 23, 41, 74] also found no increased risk of MVC involvement which was supported by the meta-analysis of two studies in *Figure 2a* (OR 0.76 (95% CI 0.34-1.70; p=0.51).[22, 74] One study investigating persons with night myopia reported slightly more night-time MVCs in these drivers than those without night myopia (p=0.044).[23]

Colour vision deficiency and the risk of MVC involvement among commercial truck drivers was investigated in eight studies (n=7916);[15, 21, 22, 34, 51, 53, 59, 77] seven set in LMICs. Three studies found an association,[15, 51, 59] however their results were not combined due to reliance on Ishihara plates which do not reliably diagnose colour vision deficiency.

Visual acuity (VA) (28 studies, n=39,129) was not found to be associated with crash involvement by 19 studies,[17, 20, 22, 24, 27-29, 31, 33-36, 38, 40, 41, 45, 50-54, 57, 63, 68, 69, 73, 75, 77, 80] irrespective of crash scenario (at-fault or not-at-fault) and severity (injurious or non-injurious). Bilateral VA 20/40 or worse was found to impact risk of MVCs (meta-analysis five studies; RR 1.21 (95% CI 1.02-1.43); p= 0.03)[27, 31, 40, 73, 77] but not at-fault MVCs (two studies; RR 1.08 (95% CI 0.74-1.60); p= 0.68) (*Figure 2a*).[27, 31]

Mixed results were reported from 13 studies (n=17,941) looking at any MVC involvement and reduced contrast sensitivity (CS).[24, 27, 31, 35, 38, 40, 54, 57, 58, 73] However, due to heterogeneity in outcome measures reported and definition of reduced CS, the meta-analysis in *Figure 2a* was restricted to only two studies which found CS to increase crash risk (RR 1.40 (95% CI 1.08-1.80); p=0.01).[31, 79] When photopic and mesopic areas under the log contrast sensitivity were investigated with any and at-fault crash involvement, only lower mesopic peaks were found to be predictive.[58]

From the 20 studies (n=13,533) looking at visual field (VF) loss and crashes, only four were suitable for meta-analysis, which found increased risk of MVC with bilateral field loss (RR 1.51 (95% CI 1.23-1.85); p< 0.001) (*Figure 2a*).[32, 51, 77, 79] There were mixed results with 9/20 studies finding an increased risk,[31, 32, 38, 42, 54, 73, 77-79] 1/20 an association for a co-linear dependent variable [19] and 10/20 a null finding.[16, 17, 33, 34, 37, 51, 53, 59, 68, 69] The increased risks were found in association with severe, bilateral VF loss and field loss affecting both central and peripheral vision.

Most studies on glare sensitivity impairments (3 studies, n=3191) found weak to no associations with crash risk;[54, 57, 73] they were unable to be meta-analysed.

19 studies (n=100167) reported on other impairments including: unilateral vision impairment,[18] general vision impairment,[21, 25, 28, 39, 41, 59, 61, 74, 76, 80, 81] non-diabetic retinopathy,[41] retinal detachment,[72] other retinal disorders,[41] refractive disorder,[41] monocular vision impairment,[41, 50] presbyopia,[41, 74] hyperopia,[22, 74] amblyopia,[18, 60] diplopia,[41] astigmatism,[22, 41] retinitis pigmentosa[26] and stereoacuity.[54, 73] Most did not find associations with MVCs, however one study from the USA reported increased injurious MVC involvement with impaired stereoacuity.[54] Another study in the UK reported increased MVC involvement with moderate/severe amblyopia[41] whilst two other studies, one in Ethiopia[21] and the other in Bangladesh,[74] reported increased MVC involvement with self-reported bilateral visual impairment.

Impact of vision-related interventions on motor vehicle crashes

Most of the six studies (n=592,897) on cataract surgery found the risk of MVC to decrease following cataract surgery[41, 47-49, 55, 62] and the 3 studies suitable for meta-analysis estimated the risk to halve

For peer review only - http://bmjopen.bmj.com/site/about/guidelines.xhtml

(RR 0.55 (95% CI 0.34-0.92); p=0.02) (*Figure 2a*).[47, 48, 55] Greater reductions to crash risk are seen after first eye surgery compared to second eye.[47] Similarly, the risk of crashing in males post-surgery is lower than females.[49]

Corrective lenses for far and near vision refractive disorders were only investigated by one study which found no associations with crash risk.[41]

Associations between eye diseases and conditions/measures of vision loss and driving cessation

There were 13 studies (n=21,939) investigating associations between glaucoma and the likelihood of driving cessation with estimates ranging from an increased risk of 1.3 to increased odds of 4.[70-72, 87, 91, 92, 99, 100, 103, 109-111, 113] Meta-analysis of the two suitable studies, illustrated in *Figure 2b*, showed a diagnosis of glaucoma increased the risk of driving cessation by 63% (95% CI 1.20 - 2.19; p< 0.01).[87, 91]

Four studies (n=14,402) looked at cataract and driving cessation with three studies reporting an increased likelihood of driving cessation by over 1.5 times; none could be meta-analysed.[72, 99, 100, 106]

From the five studies (n=6183) identified,[85, 87, 99, 106, 108] three found the presence of AMD to be predictive of driving cessation, with meta-analysis on three suitable studies reporting the overall risk of cessation to increase by 2.21 (95% CI 1.47-3.31; p< 0.01) (*Figure 2b*).[85, 87, 108]

Even though the 18 identified studies (n=23,712) were highly heterogeneous,[73, 82, 86-88, 90, 91, 94-98, 103-106, 110, 111] impaired or "poor" VA was shown to increase the chances of driving cessation in most studies,[87, 103, 104, 106, 111] with better visual acuity decreasing the risk of cessation by up to 70%.[90] The two studies looking at visual acuity in persons with glaucoma, had mixed conclusions on the effect of visual acuity on driving cessation.[95, 110]

Eight studies (n=9602) looked at the impact of CS on driving cessation.[73, 88, 94, 96, 97, 103, 106, 111] From the studies which categorised contrast sensitivity as "poor", meta-analysis on three suitable studies found poor CS to significantly increase the risk of cessation (RR 1.30 (95% CI 1.05-1.61); p=0.02) (*Figure 2b*).[94, 96, 106] Another study reported participants who had a decline of 6 or more letters in their contrast sensitivity levels after two years, as measured by a Pelli-Robson chart, to have a 71% increased risk of driving cessation.[88]

VF loss and driving cessation was investigated by 8 studies (n=7988),[88, 94-97, 103, 105, 111] and all but one found associations.[105] The likelihood of cessation was generally greater with bilateral and or more severe field loss.[88, 94, 111] One study looking at persons with bilateral glaucoma found visual field loss to double the odds of cessation.[103]

Glare sensitivity (3 studies, n=5577) and was not found to be consistently associated with driving cessation.[88, 91, 110]

Eleven studies (n=12,897) looked at driving cessation with other types of vision impairment: dark adaptation,[110] age-related maculopathy,[86] retinal detachment,[85] non-refractive vision impairment,[112] general vision loss,[85, 89, 93, 98, 100, 114, 115] retinal haemorrhage,[85] and uncorrected refractive error.[97, 112] Only two studies, one reporting on retinal haemorrhage[85] and the other on non-refractive vision impairment and uncorrected refractive error,[112] found increased risk of driving cessation.

Impact of vision-related interventions on driving cessation

For peer review only - http://bmjopen.bmj.com/site/about/guidelines.xhtml

There were two studies reporting the driving status of participants after anti-VEGF therapy (0.5 mg ranibizumab) from 4 different RCTs; MARINA (n=716; 24 months; control=sham injections) and ANCHOR (n=423; 24 months; control=photodynamic therapy (PDT)) which targeted AMD,[83] and RIDE/RISE (n=759; 24 months; control=sham injections) and RESTORE (n=345; 12 months; control=PDT) which targeted diabetic macular oedema (DMO).[84] By the end of all four trials, only drivers with AMD but not DMO treated with anti-VEGF were shown to have marked differences with the control group for the number of people who continued driving from baseline (AMD: MARINA: p=0.035, ANCHOR: p=0.002; DMO: RIDE/RISE: p=0.655, RESTORE: p=0.125).

Both studies (n=1021) looking at driving status after cataract surgery reported an increase in the proportion of participants driving after successful surgery.[101, 102]

There was only one study (n=240) looking at driving after anti-glaucoma therapy (pilocarpine-epinephrine),[107] however this is an old study and this treatment is no longer in use.

DISCUSSION

This review synthesises diverse and complex evidence from 101 studies examining vision and its impact on MVCs and driving cessation across all ages. The majority of studies in this review focused on older adults and reported more associations between vision impairment and MVCs and or cessation compared to studies on younger populations. Research was mostly observational with few studies examining the impact of interventions to improve vision. Despite the narrative summaries reporting mixed associations between VA, CS and VF defects on crashes, meta-analysis found VA, CS and VF defects to be associated with increased MVC risk. This makes sense as driving is heavily reliant on detecting hazards and responding appropriately. The mixed results in the narrative summaries however support the emerging idea of adding visual processing and cognitive tests alongside visual assessments to produce more predictive measures of safe driving.[116] Cataract surgery was shown to halve the risk of crashing. Others have reported that following cataract surgery, driving difficulties, such as self-reported night driving ability, reduced by 88%[117] with improvements in contrast sensitivity linked to these changed perceptions.[118]

Variability in the relationships between vision and MVCs may be due to several reasons. Firstly, there are many different MVC scenarios based upon the driver's role (at-fault or not) and severity (injurious or non-injurious) which are not always differentiated in research studies. MVCs are also studied in a variety of ways from self-reports to analyses of large crash databases. This may cause reliability issues. For example, an American study found agreement between these two collection methods was poor when examining the total MVCs over a three-year period.[119] Crashes can also stem from external and vehicular factors which make drawing conclusions solely based on human factors inappropriate.[120] Self-regulation, jurisdictional control on vision standards for licensing, and driving cessation could all mitigate the risk of crash involvement. This may be why associations found between vision impairment and driving cessation were strong and consistent. A diagnosis of glaucoma or AMD, and poor contrast sensitivity were all found to increase the risk of driving cessation. Anti-VEGF injections could prolong driving for people with AMD. This is of importance as older adults greatly value independent mobility and regard driving as a vital activity for daily living.[121, 122] With driving cessation linked towards multiple negative health outcomes in older adults,[4] anti-VEGF injections can have wider health benefits beyond direct impact on vision.

This review also highlights the paucity of research from LMICs despite approximately 93% of all road-traffic related deaths occurring in these countries, particularly in Africa and amongst young road users.[2] Despite the UN's push, most LMICs still lag behind the SDG targets on halving road traffic

For peer review only - http://bmjopen.bmj.com/site/about/guidelines.xhtml

mortality set in the Decade of Action for Road Safety (2011-2020).[123] Previous systematic reviews point towards legislation-based interventions which modify behaviour, such as seat-belt and helmet use, to be the most effective at reducing road injuries and crash rates in LMICs.[124, 125] These interventions are in line with UN recommendations for improving infrastructure, vehicle safety standards, and safe road user behaviours in order to reach the targets set for SDG 3.6 and 11.2.[126] However, there is no mention of licensing standards which needs to be addressed as motorisation increases worldwide. Evidence from this global review supports vision standards for licensing to be updated, enforced and given higher priority in LMICs. Even though most LMICs do have guidelines on vision, especially for commercial drivers, it is apparent from the studies in this review that many drivers unfortunately do not satisfy these conditions. The evidence for a corresponding increase in MVCs in LMICs is not well established with only one systematic review identified looking at data from these regions.[77] However, considering the weight of evidence from HICs, legislative changes and enforcement to ensure compulsory vision examinations for drivers and access to eye care services should be prioritised by LMICs to reduce MVCs.

Older drivers tend to self-regulate their driving habits by reducing their driving mileage and radius and avoiding high-risk driving situations.[127] Vision impairments have been reported to increase the likelihood of self-regulation by 19%,[128] with older drivers who self-rate their vision as “poor” 15 times more likely to modify their driving than those who regard their vision as “excellent”.[122] Our findings are consistent with these patterns of self-regulation and a diagnosis of AMD or glaucoma were found in this review to be associated with driving cessation. It is likely that self-regulation is an intermediate step towards driving cessation encompassing reductions in driving frequencies and distance.[129] However, self-regulation has been reported as an insufficient compensatory measure to reduce crash risk amongst older drivers with a vision impairment,[130, 131] which would therefore explain why glaucoma, particularly more severe glaucoma, was still linked with crashes in some studies. The relationship between crash involvement and AMD, however, was inconclusive. This may be because AMD affects central vision, thus making declines in this field easily noticeable allowing individuals to appropriately adapt their driving behaviours. Laboratory studies simulating central vision impairments show negative impacts on driving performance and safety, particularly with increasing age and distraction.[132] Further research is needed on driving patterns and behaviours of individuals with eye diseases.

Few studies, all from LMICs, in this review reported associations between colour vision deficiency and crash risk. Unfortunately, based upon their high risk of bias, these studies were deemed unsuitable for meta-analysis. This does not mean, however, that their results should be dismissed. Previous simulation studies found persons with colour vision deficiency performed worse in driving simulations compared to those with normal colour vision.[133-135] However, these associations have not always been evident in studies of MVC risk.[136] This might be why recommendations proposed by the Commission Internationale de l’Eclairage, the international authority on lighting and signal lights, are for commercial drivers only.[134] Associations found in LMICs highlight issues regarding poor road infrastructure and lighting standards.[137] Further research is needed, with standardised diagnosis of colour vision deficiency and consideration of improvements to lighting and signals in the road environment in LMICs.

This review summarises global data on different eye diseases, declines in vision function and vision-related interventions, which makes the findings applicable worldwide considering motorisation and ongoing issues of vision loss, particularly in older people. There are, however, limitations which should be acknowledged. This review highlights the highly heterogeneous nature of research investigating the impact of vision on driving which limited formal synthesis of the data in meta-

For peer review only - http://bmjopen.bmj.com/site/about/guidelines.xhtml

analyses. There were great variations in the comparator group used in each study and there were inconsistent cut-off points amongst studies looking at continuous measures of vision function. This heterogeneity also prevented sub-group analyses comparing younger to older age groups and geographic regions. Clinical and methodological heterogeneity could not investigated, even though details on participant characteristics, relevant interventions and study designs were collected, due to the small number of studies included in each meta-analysis. Looking at these parameters, however, might have explained the high statistical heterogeneity in select meta-analyses. Further, grey literature and non-English studies were not included which may have introduced publication bias and limited the number of studies identified from LMICs. Future research incorporating these areas may provide a clearer picture on how vision impairment is affecting global road safety.

In conclusion, this review summarises the global literature on the impact of vision and vision-related interventions on driving as part of the *Lancet Global Health* Commission on Global Eye Health. Select measures of vision impairment such as visual field, visual acuity and contrast sensitivity loss, and eye diseases such as glaucoma and AMD, were found to be associated with either crashes or driving cessation, while interventions such as cataract surgery and anti-VEGF injections mitigated these outcomes. However, the current literature is highly heterogeneous and more high-quality studies are needed, especially from LMICs. Future studies should aim to address these issues to allow for the global context of vision impairment and driving safety to be better documented, which may assist in the achievement of the UN's SDG road safety targets.

AUTHORS' CONTRIBUTIONS

HN, LK and JR conceived the idea of for the review. IG constructed the search. Title and abstract screening and data extraction were completed by HN, KR, JR, JZ, JF, SM and GFK. HN drafted and revised the manuscript with suggestions from LK, JR, JB, KC, GLDT, KR, MJB, JHZ, IG, JF, SM and GFK who received the manuscript and provided feedback on the draft. The final version of the manuscript was approved by all authors.

ACKNOWLEDGEMENTS

None

DECLARATION OF INTERESTS

We declare no competing interests.

NUMBER OF TABLES: 2

NUMBER OF FIGURES: 3

NUMBER OF SUPPLEMENTARY MATERIAL: 4

WORD COUNT: 3851

DATA SHARING

All data collected for the study have been provided in the submitted supplementary tables and will be made available at the time of publication with the journal. The study protocol has been published and is publically available (10.1136/bmjopen-2020-040881).

FUNDING STATEMENT

For peer review only - http://bmjopen.bmj.com/site/about/guidelines.xhtml

HN is supported by the Australian Government Research Training Program (RTP) Scholarship (N/A). MJB is supported by the Wellcome Trust (207472/Z/17/Z). JR's appointment at the University of Auckland is funded by the Buchanan Charitable Foundation (N/A), New Zealand. The *Lancet Global Health* Commission on Global Eye Health was supported by grants from The Queen Elizabeth Diamond Jubilee Trust (N/A), Moorfields Eye Charity (GR001061), NIHR Moorfields Biomedical Research Centre (N/A), The Wellcome Trust (20190426_PH2), Sightsavers (N/A), The Fred Hollows Foundation (N/A), SEVA Foundation (N/A), British Council for the Prevention of Blindness (N/A), and the Christian Blind Mission (N/A).

REFERENCES

1. Acker A, Jari K. How transport demand will change through 2050 2019 [Available from: https://www.oecd-ilibrary.org/sites/transp_outlook-en-2019-en/1/1/4/index.html?itemId=/content/publication/transp_outlook-en-2019-en&_csp_=1b3375008054c148f41fef71cd42b552&itemIGO=oecd&itemContentType=book.
2. WHO. Road Traffic Injuries 2020 [Available from: https://www.who.int/news-room/fact-sheets/detail/road-traffic-injuries.
3. UN. #Envision2030: 17 goals to transform the world for persons with disabilities 2015 [Available from: https://www.un.org/development/desa/disabilities/envision2030.html.
4. Chihuri S, Mielenz TJ, DiMaggio CJ, Betz ME, DiGuiseppi C, Jones VC, et al. Driving Cessation and Health Outcomes in Older Adults. Journal of the American Geriatrics Society. 2016;64(2):332-41.
5. Owsley C, Ball K, Sloane ME, Roenker DL, Bruni JR. Visual/cognitive correlates of vehicle accidents in older drivers. Psychol Aging. 1991;6(3):403-15.
6. Wood JM, Black AA. Ocular disease and driving. Clinical & experimental optometry. 2016;99(5):395-401.
7. Bohensky M, Charlton J, Odell M, Keeffe J. Implications of vision testing for older driver licensing. Traffic injury prevention. 2008;9(4):304-13.
8. Yan MK, Kumar H, Kerr N, Medeiros FA, Sandhu SS, Crowston J, et al. Transnational review of visual standards for driving: How Australia compares with the rest of the world. Clinical & experimental ophthalmology. 2019;47(7):847-63.
9. Burton MJ, Ramke J, Marques AP, Bourne RRA, Congdon N, Jones I, et al. The Lancet Global Health Commission on Global Eye Health: vision beyond 2020. The Lancet Global health. 2021;9(4):e489-e551.
10. Shamseer L, Moher D, Clarke M, Ghersi D, Liberati A, Petticrew M, et al. Preferred reporting items for systematic review and meta-analysis protocols (PRISMA-P) 2015: elaboration and explanation. Bmj. 2015;350:g7647.
11. Nguyen H, Di Tanna GL, Coxon K, Brown J, Ren K, Ramke J, et al. Associations between vision impairment and driving and the effectiveness of vision-related interventions: protocol for a systematic review and meta-analysis. BMJ open. 2020;10(11):e040881.
12. Adelaide Uo. Critical Appraisal Tools N/A [Available from: https://joannabriggs.org/ebp/critical_appraisal_tools.
13. Campbell M, McKenzie JE, Sowden A, Katikireddi SV, Brennan SE, Ellis S, et al. Synthesis without meta-analysis (SWiM) in systematic reviews: reporting guideline. Bmj. 2020;368:l6890.
14. Stroup DF, Berlin JA, Morton SC, Olkin I, Williamson GD, Rennie D, et al. Meta-analysis of observational studies in epidemiology: a proposal for reporting. Meta-analysis Of Observational Studies in Epidemiology (MOOSE) group. Jama. 2000;283(15):2008-12.
15. Abebe Y, Wondmikun Y. Defective color perception among car drivers in Addis Ababa, Ethiopia. Traffic Injury Prevention. 2002;3(4):294-7.
16. Abraham EG, Ezepue UF, Umeh RE, Ekanem U. Causes of visual impairment among commercial motor vehicle drivers in Uyo metropolis, Akwa Ibom State, Nigeria. Orient Journal of Medicine. 2010;22(1/4):unpaginated.

For peer review only - http://bmjopen.bmj.com/site/about/guidelines.xhtml

17. Adekoya BJ, Owoeye JFA, Adepoju FG, Ajaiyeoba AI. Visual function survey of commercial intercity vehicle drivers in Ilorin, Nigeria. Canadian journal of ophthalmology Journal canadien d'ophtalmologie. 2009;44(3):261-4.
18. Baker JM, Drews-Botsch C, Pfeiffer MR, Curry AE. Driver licensing and motor vehicle crash rates among young adults with amblyopia and unilateral vision impairment. Journal of AAPOS : the official publication of the American Association for Pediatric Ophthalmology and Strabismus. 2019;23(4):230-2.
19. Ball K, Owsley C, Sloane ME, Roenker DL, Bruni JR. Visual attention problems as a predictor of vehicle crashes in older drivers. Investigative ophthalmology & visual science. 1993;34(11):3110-23.
20. Bekibele CO, Fawole OI, Bamgboye AE, Adekunle LV, Ajayi R, Baiyeroju AM. Prevalence of refractive error and attitude to spectacle use among drivers of public institutions in Ibadan, Nigeria. Annals of African medicine. 2007;6(1):26-30.
21. Biza M, Mossie A, Woldemichael K, Gelaw Y. Visual impairment and road traffic accidents among drivers in Jimma Town, Southwest Ethiopia. Ethiopian medical journal. 2013;51(2):123-32.
22. Boadi-Kusi SB, Kyei S, Asare FA, Owusu-Ansah A, Awuah A, Darko-Takyi C. Visual function among commercial vehicle drivers in the central region of Ghana. Journal of optometry. 2016;9(1):54-63.
23. Cohen Y, Zadok D, Barkana Y, Shochat Z, Ashkenazi I, Avni I, et al. Relationship between night myopia and night-time motor vehicle accidents. Acta ophthalmologica Scandinavica. 2007;85(4):367-70.
24. Cross JM, McGwin G, Jr., Rubin GS, Ball KK, West SK, Roenker DL, et al. Visual and medical risk factors for motor vehicle collision involvement among older drivers. The British journal of ophthalmology. 2009;93(3):400-4.
25. Emerole CG, Nneli RO. Visual indices of motor vehicle drivers in relation to road safety in Nigeria. Nigerian journal of physiological sciences : official publication of the Physiological Society of Nigeria. 2013;28(1):57-62.
26. Fishman GA, Anderson RJ, Stinson L, Haque A. Driving performance of retinitis pigmentosa patients. The British journal of ophthalmology. 1981;65(2):122-6.
27. Green KA, McGwin G, Jr., Owsley C. Associations between visual, hearing, and dual sensory impairments and history of motor vehicle collision involvement of older drivers. Journal of the American Geriatrics Society. 2013;61(2):252-7.
28. Gresset J, Meyer F. Risk of automobile accidents among elderly drivers with impairments or chronic diseases. Canadian journal of public health = Revue canadienne de sante publique. 1994;85(4):282-5.
29. Gresset JA, Meyer FM. Risk of accidents among elderly car drivers with visual acuity equal to 6/12 or 6/15 and lack of binocular vision. Ophthalmic & physiological optics : the journal of the British College of Ophthalmic Opticians (Optometrists). 1994;14(1):33-7.
30. Haymes SA, Leblanc RP, Nicolela MT, Chiasson LA, Chauhan BC. Risk of falls and motor vehicle collisions in glaucoma. Investigative ophthalmology & visual science. 2007;48(3):1149-55.
31. Huisingh C, Levitan EB, Irvin MR, MacLennan P, Wadley V, Owsley C. Visual Sensory and Visual-Cognitive Function and Rate of Crash and Near-Crash Involvement Among Older Drivers Using Naturalistic Driving Data. Investigative ophthalmology & visual science. 2017;58(7):2959-67.
32. Huisingh C, McGwin G, Wood J, Owsley C. The driving visual field and a history of motor vehicle collision involvement in older drivers: A population- based examination. Investigative Ophthalmology and Visual Science. 2015;56(1):132-8.
33. Humphriss D. Three South African studies on the relation between road accidents and drivers' vision. Ophthalmic & physiological optics : the journal of the British College of Ophthalmic Opticians (Optometrists). 1987;7(1):73-9.
34. Isawumi MA, Adeoti CO, Ubah JN, Oluwatimilehin IO, Raji RA. Ocular status of commercial drivers in Osun State, Nigeria. African journal of medicine and medical sciences. 2011;40(4):405-11.

For peer review only - http://bmjopen.bmj.com/site/about/guidelines.xhtml

35. Ivers RQ, Mitchell P, Cumming RG. Sensory impairment and driving: the Blue Mountains Eye Study. American journal of public health. 1999;89(1):85-7.
36. Keeffe JE, Jin CF, Weih LM, McCarty CA, Taylor HR. Vision impairment and older drivers: who's driving? The British journal of ophthalmology. 2002;86(10):1118-21.
37. Kristalovich L, Ben Mortenson W. Visual Field Impairment and Driver Fitness: A 1-Year Review of Crashes and Traffic Violations. The American journal of occupational therapy : official publication of the American Occupational Therapy Association. 2019;73(5):7305345010p1-p6.
38. Kwon M, Huisingh C, Rhodes LA, McGwin G, Jr., Wood JM, Owsley C. Association between Glaucoma and At-fault Motor Vehicle Collision Involvement among Older Drivers: A Population-based Study. Ophthalmology. 2016;123(1):109-16.
39. Maag U, Vanasse C, Dionne G, Laberge-Nadeau C. Taxi drivers' accidents: how binocular vision problems are related to their rate and severity in terms of the number of victims. Accident; analysis and prevention. 1997;29(2):217-24.
40. Margolis KL, Kerani RP, McGovern P, Songer T, Cauley JA, Ensrud KE. Risk factors for motor vehicle crashes in older women. Journals of Gerontology - Series A Biological Sciences and Medical Sciences. 2002;57(3):M186-M91.
41. McCloskey LW, Koepsell TD, Wolf ME, Buchner DM. Motor vehicle collision injuries and sensory impairments of older drivers. Age and ageing. 1994;23(4):267-73.
42. McGwin G, Jr., Huisingh C, Jain SG, Girkin CA, Owsley C. Binocular visual field impairment in glaucoma and at-fault motor vehicle collisions. Journal of glaucoma. 2015;24(2):138-43.
43. McGwin G, Jr., Mays A, Joiner W, Decarlo DK, McNeal S, Owsley C. Is glaucoma associated with motor vehicle collision involvement and driving avoidance? Investigative ophthalmology & visual science. 2004;45(11):3934-9.
44. McGwin G, Jr., Mitchell B, Searcey K, Albert MA, Feist R, Mason JO, 3rd, et al. Examining the association between age-related macular degeneration and motor vehicle collision involvement: a retrospective cohort study. The British journal of ophthalmology. 2013;97(9):1173-6.
45. McGwin G, Jr., Sims RV, Pulley L, Roseman JM. Relations among chronic medical conditions, medications, and automobile crashes in the elderly: a population-based case-control study. American journal of epidemiology. 2000;152(5):424-31.
46. McGwin G, Jr., Xie A, Mays A, Joiner W, DeCarlo DK, Hall TA, et al. Visual field defects and the risk of motor vehicle collisions among patients with glaucoma. Investigative ophthalmology & visual science. 2005;46(12):4437-41.
47. Meuleners LB, Brameld K, Fraser ML, Chow K. The impact of first- and second-eye cataract surgery on motor vehicle crashes and associated costs. Age and ageing. 2019;48(1):128-33.
48. Meuleners LB, Hendrie D, Lee AH, Ng JQ, Morlet N. The effectiveness of cataract surgery in reducing motor vehicle crashes: a whole population study using linked data. Ophthalmic epidemiology. 2012;19(1):23-8.
49. Meuleners LB, Ng JQ, Fraser M, Hendrie D, Morlet N. Impact of gender on first eye cataract surgery and motor vehicle crash risk for older drivers. Clinical & experimental ophthalmology. 2012;40(6):591-6.
50. Ogbonnaya CE, Uro-Chukwu HC, Sunday-Adeoye I. Ocular health status and visual fitness of commercial drivers in Abakaliki Metropolis, Nigeria. East African Medical Journal. 2018;95(7):1758-66.
51. Oladehinde MK, Adeoye AO, Adegbehingbe BO, Onakoya AO. Visual functions of commercial drivers in relation to road accidents in Nigeria. Indian journal of occupational and environmental medicine. 2007;11(2):71-5.
52. Ono T, Yuki K, Asaoka R, Kouyama K, Abe T, Tanabe S, et al. Glaucomatous visual field defect severity and the prevalence of motor vehicle collisions in Japanese: a hospital/clinic-based cross-sectional study. Journal of ophthalmology. 2015;2015(101524199):497067.

For peer review only - http://bmjopen.bmj.com/site/about/guidelines.xhtml

53. Ovenseri-Ogomo G, Adofo M. Poor vision, refractive errors and barriers to treatment among commercial vehicle drivers in the Cape Coast municipality. African health sciences. 2011;11(1):97-102.
54. Owsley C, McGwin G, Jr., Ball K. Vision impairment, eye disease, and injurious motor vehicle crashes in the elderly. Ophthalmic epidemiology. 1998;5(2):101-13.
55. Owsley C, McGwin G, Jr., Sloane M, Wells J, Stalvey BT, Gauthreaux S. Impact of cataract surgery on motor vehicle crash involvement by older adults. JAMA. 2002;288(7):841-9.
56. Owsley C, Stalvey B, Wells J, Sloane ME. Older drivers and cataract: driving habits and crash risk. The journals of gerontology Series A, Biological sciences and medical sciences. 1999;54(4):M203-11.
57. Owsley C, Stalvey BT, Wells J, Sloane ME, McGwin G, Jr. Visual risk factors for crash involvement in older drivers with cataract. Archives of ophthalmology (Chicago, Ill : 1960). 2001;119(6):881-7.
58. Owsley C, Swain T, Liu R, McGwin G, Jr., Kwon MY. Association of Photopic and Mesopic Contrast Sensitivity in older drivers with risk of motor vehicle collision using naturalistic driving data. BMC ophthalmology. 2020;20(1):47.
59. Pepple G, Adio A. Visual function of drivers and its relationship to road traffic accidents in Urban Africa. SpringerPlus. 2014;3(101597967):47.
60. Rahi JS, Cumberland PM, Peckham CS. Does amblyopia affect educational, health, and social outcomes? Findings from 1958 British birth cohort. BMJ (Clinical research ed). 2006;332(7545):820-5.
61. Runge JW. Medical conditions and driver crash risk: Do license restrictions affect public safety? Annals of Emergency Medicine. 2000;36(2):164-5.
62. Schlenker MB, Thiruchelvam D, Redelmeier DA. Association of Cataract Surgery With Traffic Crashes. JAMA ophthalmology. 2018;136(9):998-1007.
63. Sims RV, Owsley C, Allman RM, Ball K, Smoot TM. A preliminary assessment of the medical and functional factors associated with vehicle crashes by older adults. Journal of the American Geriatrics Society. 1998;46(5):556-61.
64. Szlyk JP, Pizzimenti CE, Fishman GA, Kelsch R, Wetzel LC, Kagan S, et al. A comparison of driving in older subjects with and without age-related macular degeneration. Archives of ophthalmology (Chicago, Ill : 1960). 1995;113(8):1033-40.
65. Tanabe S, Yuki K, Ozeki N, Shiba D, Abe T, Kouyama K, et al. The association between primary open-angle glaucoma and motor vehicle collisions. Investigative ophthalmology & visual science. 2011;52(7):4177-81.
66. Wood JM, Black AA, Mallon K, Kwan AS, Owsley C. Effects of Age-Related Macular Degeneration on Driving Performance. Investigative ophthalmology & visual science. 2018;59(1):273-9.
67. Wood JM, Black AA, Mallon K, Thomas R, Owsley C. Glaucoma and Driving: On-Road Driving Characteristics. PloS one. 2016;11(7):e0158318.
68. Yuki K, Asaoka R, Tsubota K. The relationship between central visual field damage and motor vehicle collisions in primary open-angle glaucoma patients. PloS one. 2014;9(12):e115572.
69. Yuki K, Awano-Tanabe S, Ono T, Shiba D, Murata H, Asaoka R, et al. Risk Factors for Motor Vehicle Collisions in Patients with Primary Open-Angle Glaucoma: A Multicenter Prospective Cohort Study. PloS one. 2016;11(11):e0166943.
70. Adler G, Bauer MJ, Rottunda S, Kuskowski M. Driving habits and patterns in older men with glaucoma. Social work in health care. 2005;40(3):75-87.
71. Deshmukh AV, Murthy GJ, Reddy A, Murthy PR, Kattige J, Murthy VR. Older Drivers and Glaucoma in India: Driving Habits and Crash Risks. Journal of glaucoma. 2019;28(10):896-900.
72. Naredo Turrado J, Orriols L, Contrand B, Zins M, Salmi LR, Lafont S, et al. Chronic medical conditions and their association with crash risk and changes in driving habits: a prospective study of

For peer review only

For peer review only - http://bmjopen.bmj.com/site/about/guidelines.xhtml

the GAZEL cohort. Injury prevention : journal of the International Society for Child and Adolescent Injury Prevention. 2020((Lafont) Universite Lyon, IFSTTAR, Universite Lyon 1, Bron, France).
73. Rubin GS, Ng ESW, Bandeen-Roche K, Keyl PM, Freeman EE, West SK. A prospective, population-based study of the role of visual impairment in motor vehicle crashes among older drivers: the SEE study. Investigative ophthalmology & visual science. 2007;48(4):1483-91.
74. Ahmed M, Patnaik JL, Whitestone N, Hossain MA, Alauddin M, Husain L, et al. Visual Impairment and Risk of Self-Reported Road Traffic Crashes Among Bus Drivers in Bangladesh. Asia-Pacific journal of ophthalmology (Philadelphia, Pa). 2022;11(1):72-8.
75. Alvarez-Peregrina C, Martinez-Perez C, Villa-Collar C, Sánchez-Tena M. Influence of Vision on Drivers: A Pilot Study. International journal of environmental research and public health. 2021;18(22).
76. Crizzle AM, Toxopeus R, Alphonsus K. Medical Conditions and Crash Risk in Commercial Motor Vehicle Drivers. Journal of occupational and environmental medicine. 2020;62(8):628-33.
77. Piyasena P, Olvera-Herrera VO, Chan VF, Clarke M, Wright DM, MacKenzie G, et al. Vision impairment and traffic safety outcomes in low-income and middle-income countries: a systematic review and meta-analysis. The Lancet Global health. 2021;9(10):e1411-e22.
78. Swain TA, McGwin G, Jr., Wood JM, Antin JF, Owsley C. Naturalistic Driving Techniques and Association of Visual Risk Factors With At-Fault Crashes and Near Crashes by Older Drivers With Vision Impairment. JAMA Ophthalmol. 2021;139(6):639-45.
79. Swain TA, McGwin G, Jr., Wood JM, Owsley C. Motion perception as a risk factor for motor vehicle collision involvement in drivers ≥ 70 years. Accid Anal Prev. 2021;151:105956.
80. Vofo BN, Ayuk DA, Pe'er J, Chichom-Mefire A, Tendongfor N, Nche EN. Visual assessment of commercial drivers in the South West Region of Cameroon. BMC Ophthalmol. 2021;21(1):148.
81. Wedenoja J, Kalsi J, Salenius S, Parkkari K, Kaarniranta K, Tervo T. The role of vision-related problems in fatal road accidents in Finland. Acta Ophthalmol. 2021;99(4):427-30.
82. Anstey KJ, Windsor TD, Luszcz MA, Andrews GR. Predicting driving cessation over 5 years in older adults: Psychological well-being and cognitive competence are stronger predictors than physical health. Journal of the American Geriatrics Society. 2006;54(1):121-6.
83. Bressler NM, Chang TS, Varma R, Suner I, Lee P, Dolan CM, et al. Driving ability reported by neovascular age-related macular degeneration patients after treatment with ranibizumab. Ophthalmology. 2013;120(1):160-8.
84. Bressler NM, Varma R, Mitchell P, Suner IJ, Dolan C, Ward J, et al. Effect of Ranibizumab on the Decision to Drive and Vision Function Relevant to Driving in Patients With Diabetic Macular Edema: Report From RESTORE, RIDE, and RISE Trials. JAMA ophthalmology. 2016;134(2):160-6.
85. Campbell MK, Bush TL, Hale WE. Medical conditions associated with driving cessation in community- dwelling, ambulatory elders. Journals of Gerontology. 1993;48(4):S230-S4.
86. DeCarlo DK, Scilley K, Wells J, Owsley C. Driving habits and health-related quality of life in patients with age-related maculopathy. Optometry and vision science : official publication of the American Academy of Optometry. 2003;80(3):207-13.
87. Edwards JD, Ross LA, Ackerman ML, Small BJ, Ball KK, Bradley S, et al. Longitudinal predictors of driving cessation among older adults from the ACTIVE clinical trial. The journals of gerontology Series B, Psychological sciences and social sciences. 2008;63(1):P6-12.
88. Freeman EE, Munoz B, Turano KA, West SK. Measures of visual function and time to driving cessation in older adults. Optometry and vision science : official publication of the American Academy of Optometry. 2005;82(8):765-73.
89. Gallo JJ, Rebok GW, Lesikar SE. The driving habits of adults aged 60 years and older. Journal of the American Geriatrics Society. 1999;47(3):335-41.
90. Garre-Olmo J, Pujol XP, Garriga OT, Lopez-Pousa S, Vilalta-Franch J. Biopsychosocial differences between drivers and non-drivers over the age of 74. Archives of gerontology and geriatrics. 2009;49(3):355-9.

For peer review only - http://bmjopen.bmj.com/site/about/guidelines.xhtml

91. Gilhotra JS, Mitchell P, Ivers R, Cumming RG. Impaired vision and other factors associated with driving cessation in the elderly: the Blue Mountains Eye Study. Clinical & experimental ophthalmology. 2001;29(3):104-7.
92. Goh YW, Ang GS, Azuara-Blanco A. Lifetime visual prognosis of patients with glaucoma. Clinical & experimental ophthalmology. 2011;39(8):766-70.
93. Hajek A, Brettschneider C, Eisele M, van den Bussche H, Wiese B, Mamone S, et al. Prevalence and determinants of driving habits in the oldest old: Results of the multicenter prospective AgeCoDe-AgeQualiDe study. Archives of gerontology and geriatrics. 2019;82(8214379, 7ax):245-50.
94. Huisingh C, McGwin G, Jr., Owsley C. Association of visual sensory function and higher-order visual processing skills with incident driving cessation. Clinical & experimental optometry. 2016;99(5):441-8.
95. Janz NK, Musch DC, Gillespie BW, Wren PA, Niziol LM, Collaborative Initial Glaucoma Treatment Study I. Evaluating clinical change and visual function concerns in drivers and nondrivers with glaucoma. Investigative ophthalmology & visual science. 2009;50(4):1718-25.
96. Keay L, Munoz B, Turano KA, Hassan SE, Munro CA, Duncan DD, et al. Visual and cognitive deficits predict stopping or restricting driving: the Salisbury Eye Evaluation Driving Study (SEEDS). Investigative ophthalmology & visual science. 2009;50(1):107-13.
97. Keay L, Palagyi A, Do V, White A, Lamoureux E, Ivers RQ, et al. Vision and driving status of older Australians with cataract: an investigation of public hospital waiting lists. Clinical & experimental optometry. 2016;99(5):449-55.
98. Levecq L, De Potter P, Jamart J. Visual acuity and factors influencing automobile driving status in 1,000 patients age 60 and older. Graefe's archive for clinical and experimental ophthalmology = Albrecht von Graefes Archiv fur klinische und experimentelle Ophthalmologie. 2013;251(3):881-7.
99. MacLeod KE, Satariano WA, Ragland DR. The Impact of Health Problems on Driving Status among Older Adults. Journal of transport & health. 2014;1(2):86-94.
100. Marottoli RA, Ostfeld AM, Merrill SS, Perlman GD, Foley DJ, Cooney LM, Jr. Driving cessation and changes in mileage driven among elderly individuals. Journal of gerontology. 1993;48(5):S255-60.
101. Monestam E, Lundquist B, Wachtmeister L. Visual function and car driving: longitudinal results 5 years after cataract surgery in a population. The British journal of ophthalmology. 2005;89(4):459-63.
102. Monestam E, Wachtmeister L. Impact of cataract surgery on car driving: a population based study in Sweden. The British journal of ophthalmology. 1997;81(1):16-22.
103. Ramulu PY, West SK, Munoz B, Jampel HD, Friedman DS. Driving cessation and driving limitation in glaucoma: the Salisbury Eye Evaluation Project. Ophthalmology. 2009;116(10):1846-53.
104. Ross LA, Anstey KJ, Kiely KM, Windsor TD, Byles JE, Luszcz MA, et al. Older drivers in Australia: trends in driving status and cognitive and visual impairment. Journal of the American Geriatrics Society. 2009;57(10):1868-73.
105. Segal-Gidan F, Varma R, Salazar X, Mack WJ. Factors influencing driving status in an older Latino population. Journal of aging and health. 2010;22(3):332-47.
106. Sengupta S, van Landingham SW, Solomon SD, Do DV, Friedman DS, Ramulu PY. Driving habits in older patients with central vision loss. Ophthalmology. 2014;121(3):727-32.
107. Stafford WR. Traditional antiglaucoma therapy: A clinical survey of side effects. Current Therapeutic Research - Clinical and Experimental. 1981;29(2):265-74.
108. Stewart RB, Moore MT, Marks RG, May FE, Hale WE. Driving cessation in the elderly: An analysis of symptoms, diseases, and medications. Journal of Geriatric Drug Therapy. 1993;8(2):45-60.
109. Takahashi A, Yuki K, Awano-Tanabe S, Ono T, Shiba D, Tsubota K. Association between glaucoma severity and driving cessation in subjects with primary open-angle glaucoma. BMC ophthalmology. 2018;18(1):122.

For peer review only - http://bmjopen.bmj.com/site/about/guidelines.xhtml

110. Tam ALC, Trope GE, Buys YM, Yang Y, Shen C, Jin Y-P. Self-perceived Impact of Glaucomatous Visual Field Loss and Visual Disabilities on Driving Difficulty and Cessation. Journal of glaucoma. 2018;27(11):981-6.
111. van Landingham SW, Hochberg C, Massof RW, Chan E, Friedman DS, Ramulu PY. Driving patterns in older adults with glaucoma. BMC ophthalmology. 2013;13(100967802):4.
112. Zebardast N, Swenor BK, van Landingham SW, Massof RW, Munoz B, West SK, et al. Comparing the Impact of Refractive and Nonrefractive Vision Loss on Functioning and Disability: The Salisbury Eye Evaluation. Ophthalmology. 2015;122(6):1102-10.
113. Kaleem MA, Rajjoub R, Schiefer C, Wall J, Applegate C, Tian J, et al. Characteristics of Glaucoma Patients Attending a Vision Rehabilitation Service. Ophthalmology Glaucoma. 2021;4(6):638-45.
114. Moon S, Park K. The Predictors of Driving Cessation among Older Drivers in Korea. International journal of environmental research and public health. 2020;17(19).
115. Robinson JL, Liu Y, Chen Q. Licensure and Driving Status among Visually Impaired Persons. Optom Vis Sci. 2021;98(12):1334-9.
116. Grundler W, Strasburger H. Visual attention outperforms visual-perceptual parameters required by law as an indicator of on-road driving performance. PloS one. 2020;15(8):e0236147.
117. Subzwari S, Desapriya E, Scime G, Babul S, Jivani K, Pike I. Effectiveness of cataract surgery in reducing driving-related difficulties: a systematic review and meta-analysis. Injury prevention : journal of the International Society for Child and Adolescent Injury Prevention. 2008;14(5):324-8.
118. Fraser ML, Meuleners LB, Lee AH, Ng JQ, Morlet N. Which visual measures affect change in driving difficulty after first eye cataract surgery? Accident; analysis and prevention. 2013;58:10-4.
119. Singletary BA, Do AN, Donnelly JP, Huisingh C, Mefford MT, Modi R, et al. Self-reported vs state-recorded motor vehicle collisions among older community dwelling individuals. Accident; analysis and prevention. 2017;101:22-7.
120. Haddon W, Jr. Energy damage and the ten countermeasure strategies. J Trauma. 1973;13(4):321-31.
121. Strogatz D, Mielenz TJ, Johnson AK, Baker IR, Robinson M, Mebust SP, et al. Importance of Driving and Potential Impact of Driving Cessation for Rural and Urban Older Adults. The Journal of rural health : official journal of the American Rural Health Association and the National Rural Health Care Association. 2020;36(1):88-93.
122. Unsworth CA, Wells Y, Browning C, Thomas SA, Kendig H. To continue, modify or relinquish driving: findings from a longitudinal study of healthy ageing. Gerontology. 2007;53(6):423-31.
123. Bhalla K, Mohan D, O'Neill B. How much would low- and middle-income countries benefit from addressing the key risk factors of road traffic injuries? International journal of injury control and safety promotion. 2020;27(1):83-90.
124. Forjuoh SN. Traffic-related injury prevention interventions for low-income countries. Injury control and safety promotion. 2003;10(1-2):109-18.
125. Staton C, Vissoci J, Gong E, Toomey N, Wafula R, Abdelgadir J, et al. Road Traffic Injury Prevention Initiatives: A Systematic Review and Metasummary of Effectiveness in Low and Middle Income Countries. PloS one. 2016;11(1):e0144971.
126. Europe UNECf. Road Safety for All: United Nations; 2020 [Available from: https://unece.org/transport/publications/road-safety-all-0.
127. Bergen G, West BA, Luo F, Bird DC, Freund K, Fortinsky RH, et al. How do older adult drivers self-regulate? Characteristics of self-regulation classes defined by latent class analysis. Journal of safety research. 2017;61:205-10.
128. Braitman KA, McCartt AT. Characteristics of older drivers who self-limit their driving. Annals of advances in automotive medicine Association for the Advancement of Automotive Medicine Annual Scientific Conference. 2008;52:245-54.

For peer review only - http://bmjopen.bmj.com/site/about/guidelines.xhtml

129. Harmon AC, Babulal G, Vivoda JM, Zikmund-Fisher BJ, Carr DB. Planning for a Nondriving Future: Behaviors and Beliefs Among Middle-Aged and Older Drivers. Geriatrics (Basel, Switzerland). 2018;3(2).
130. Owsley C, McGwin G, Jr., Phillips JM, McNeal SF, Stalvey BT. Impact of an educational program on the safety of high-risk, visually impaired, older drivers. American journal of preventive medicine. 2004;26(3):222-9.
131. Ross LA, Clay OJ, Edwards JD, Ball KK, Wadley VG, Vance DE, et al. Do older drivers at-risk for crashes modify their driving over time? The journals of gerontology Series B, Psychological sciences and social sciences. 2009;64(2):163-70.
132. Zhang CT, Bowers AR, Savage SW. The Effects of Age, Distraction, and Simulated Central Vision Impairment on Hazard Detection in a Driving Simulator. Optometry and vision science : official publication of the American Academy of Optometry. 2020;97(4):239-48.
133. Cole BL. Protan colour vision deficiency and road accidents. Clinical & experimental optometry. 2002;85(4):246-53.
134. Cole BL. Colour blindness and driving. Clinical & experimental optometry. 2016;99(5):484-7.
135. Atchison DA, Pedersen CA, Dain SJ, Wood JM. Traffic signal color recognition is a problem for both protan and deutan color-vision deficients. Human factors. 2003;45(3):495-503.
136. Tagarelli A, Piro A, Tagarelli G, Lantieri PB, Risso D, Olivieri RL. Colour blindness in everyday life and car driving. Acta ophthalmologica Scandinavica. 2004;82(4):436-42.
137. Dhibi M. Road safety determinants in low and middle income countries. International journal of injury control and safety promotion. 2019;26(1):99-107.

FIGURE LEGENDS

Figure 1 Flow chart of search with papers reporting on MVC and driving cessation

Figure 2a Associations between MVC involvement and vision impairment and vision-related intervention

Figure 2b Association between driving cessation and vision impairment

Figure 3 Network diagram illustrating strength of association of vision impairment on motor vehicle crashes (MVC) and driving cessation found by narrative summaries and meta-analyses of available evidence

For peer review only

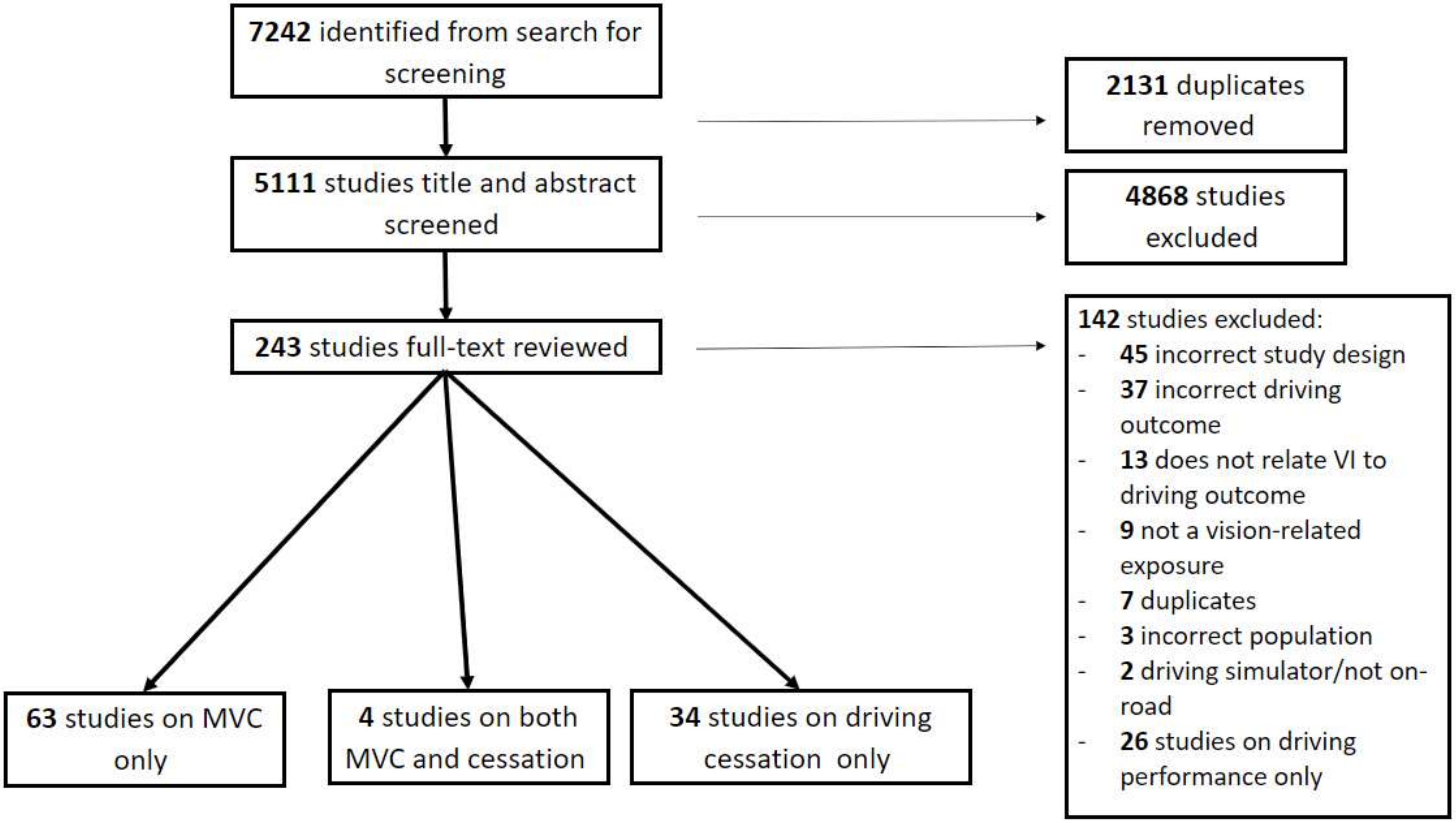

Figure 1 Flow chart of search with papers reporting on MVC and driving cessation

<table>
<tr><th>Vision impairment</th><th>Outcome</th><th>Association</th></tr>
<tr><td>Glaucoma</td><td>Any MVC</td><td>
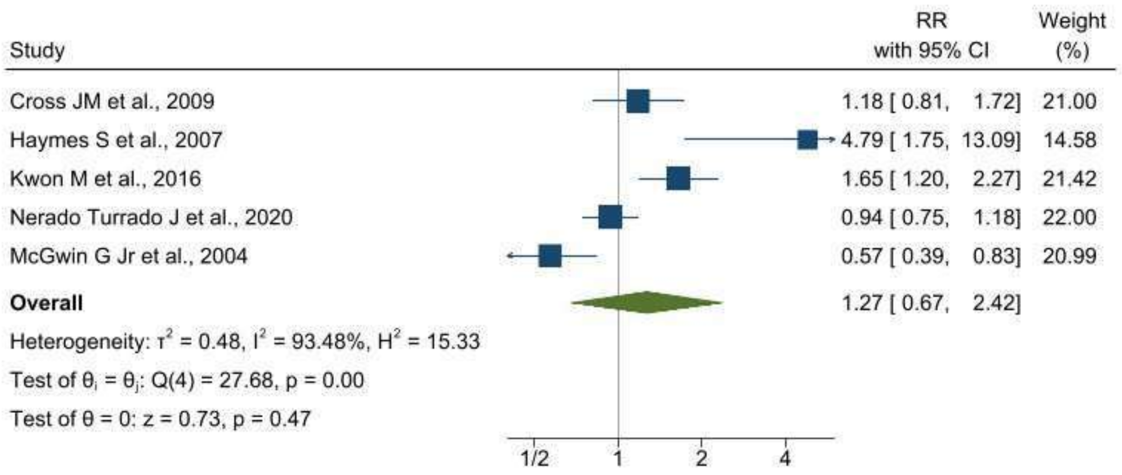

</td></tr>
<tr><td></td><td>At-fault MVC</td><td>
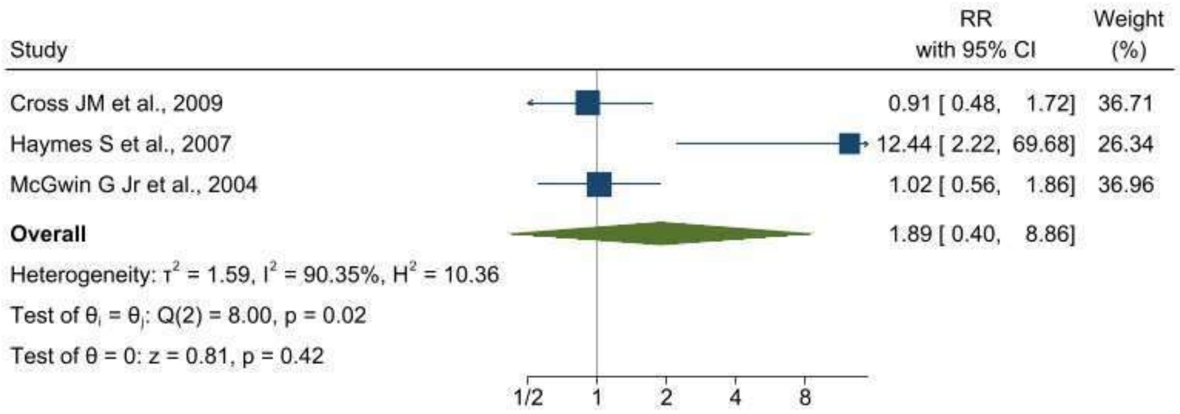

</td></tr>
</table>

<table>
<tr><td>Cataract</td><td>Any MVC</td><td>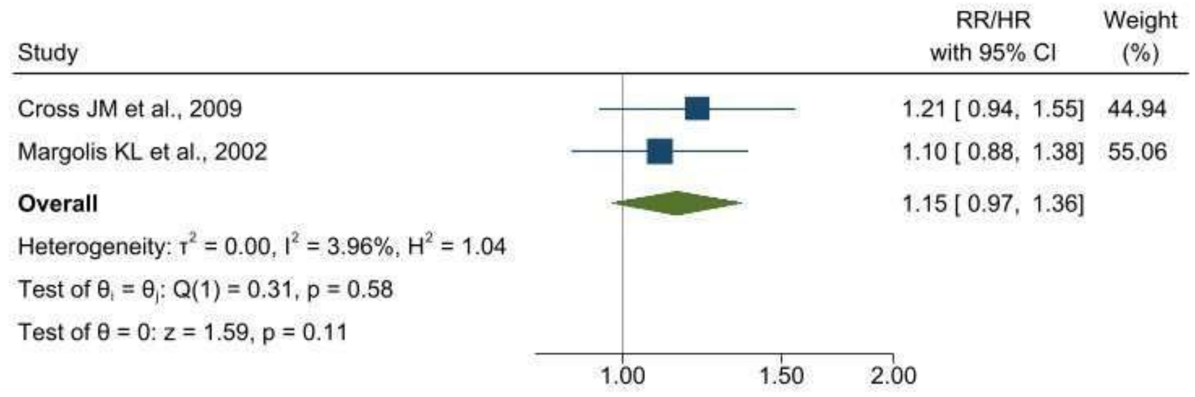

NOTE: POOLED ONE STUDY WITH RR AND ANOTHER WITH HR</td></tr>
<tr><td>Stereopsis</td><td>Any MVC</td><td>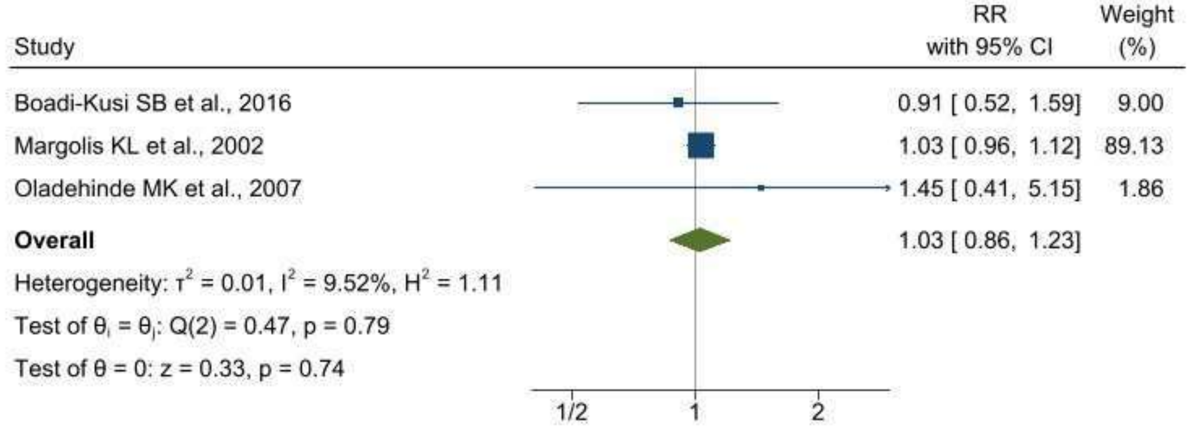
</td></tr>
</table>

Myopia	Any MVC	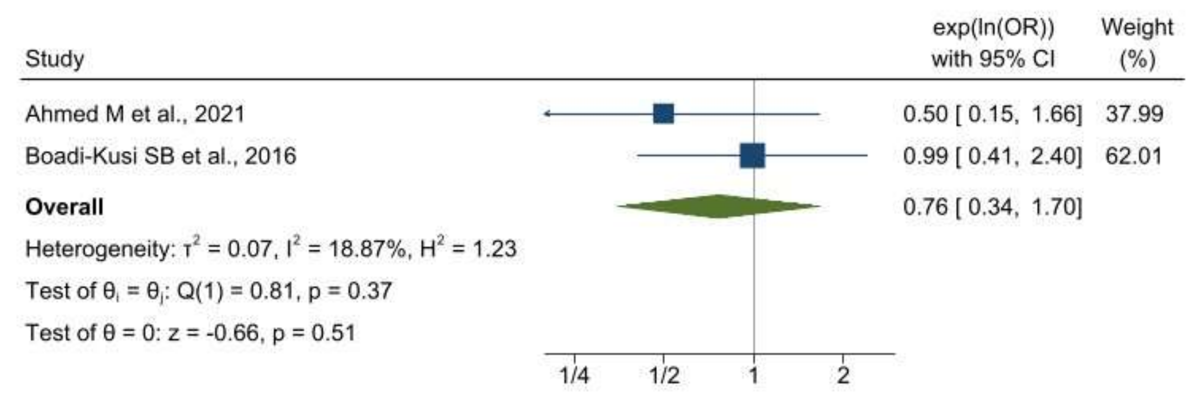

For peer review only

<table>
<tr><td rowspan="2">VA ≤ 20/40</td><td>Any MVC</td><td>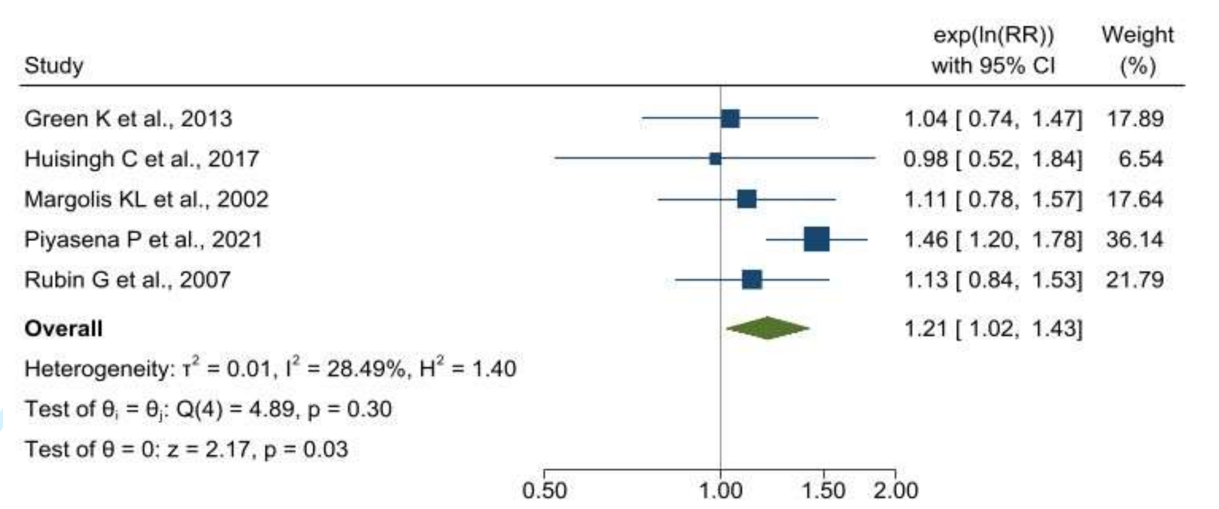
</td></tr>
<tr><td>At-fault MVC</td><td>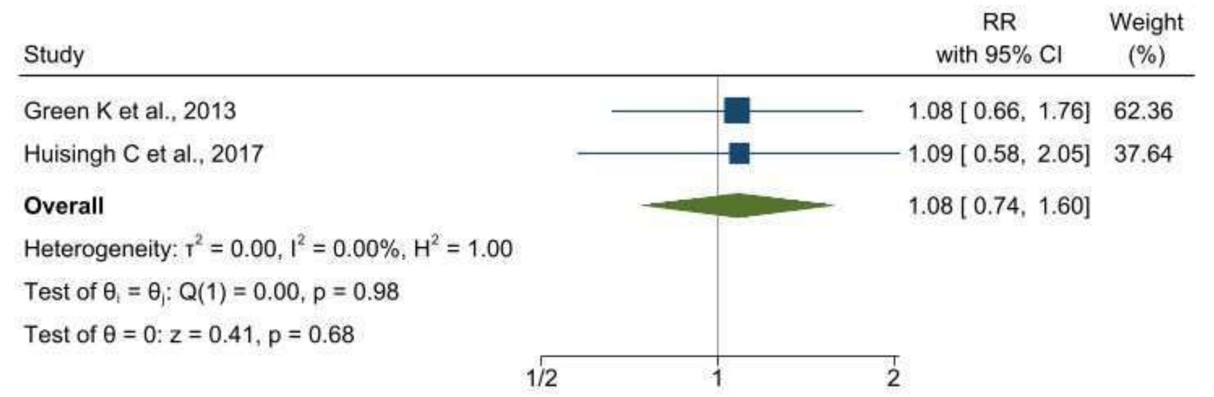
</td></tr>
</table>

<table>
<tr><td>Contrast Sensitivity Loss</td><td>Any MVC</td><td>
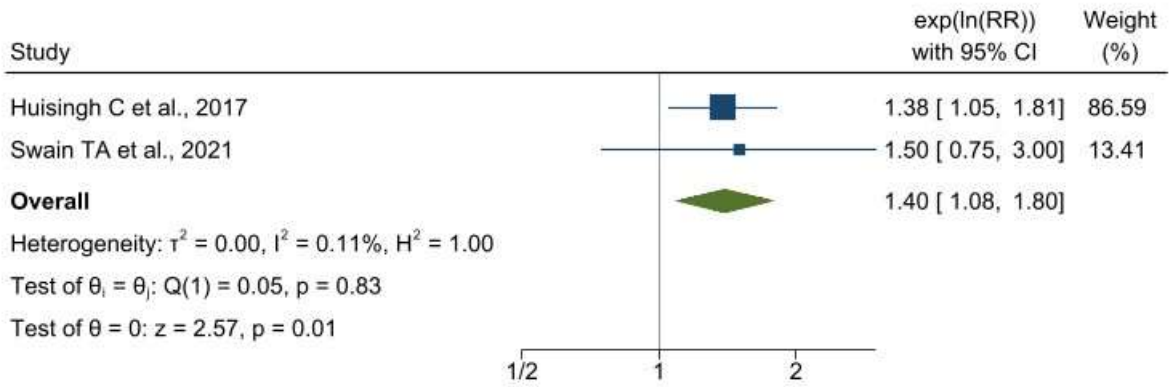

</td></tr>
<tr><td>Visual Field Loss</td><td>Any MVC</td><td>
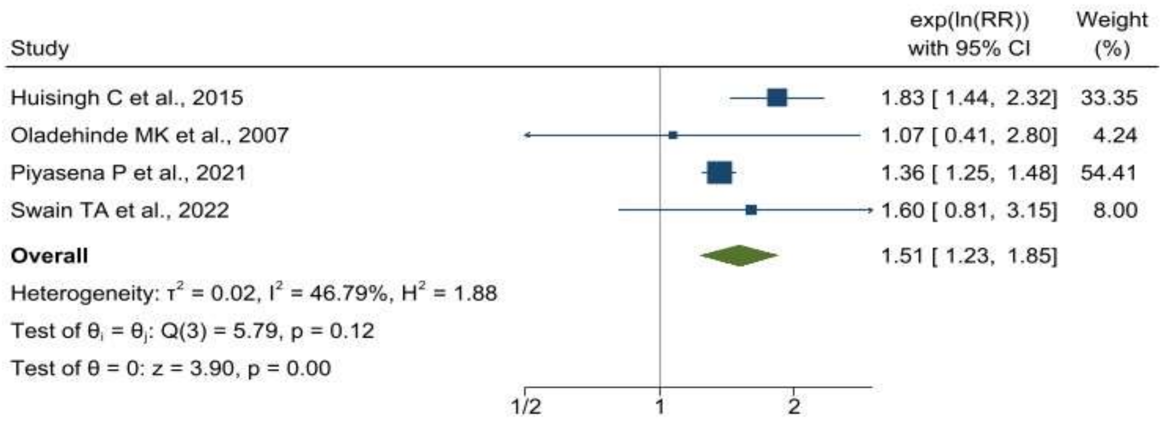

</td></tr>
<tr><td>Vision-related Intervention</td><td>Outcome</td><td>Association</td></tr>
</table>

**Cataract Surgery**	**Any MVC**	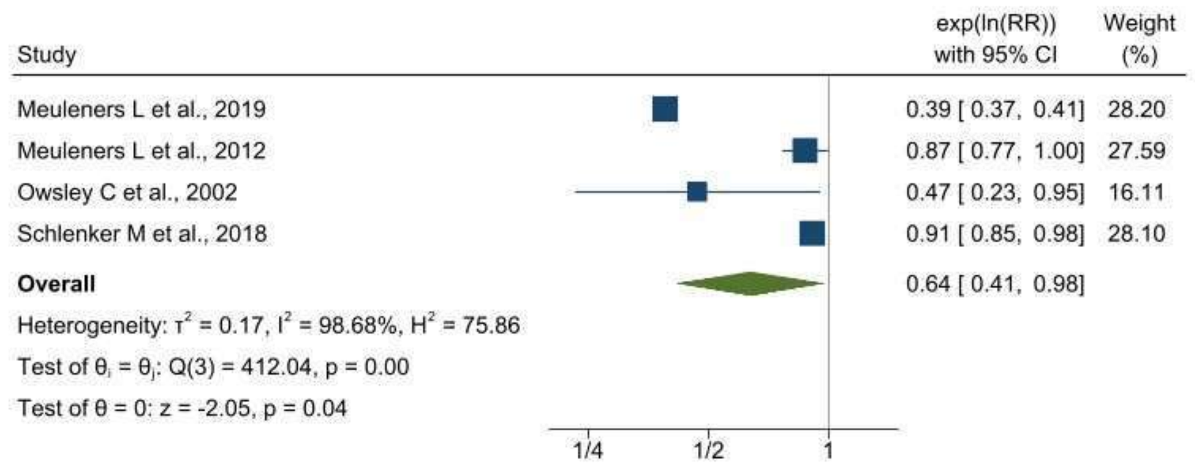<br>HOWEVER WHEN DROPPING SCHLENKER M ET AL., 2018 (UNADJUSTED EFFECT MEASURE):<br><br>**Overall** RR 0.55 [0.35, 0.92]<br>Heterogeneity: $T^2 = 0.17$, $I^2 = 97.10\%\%$, $H^2 = 34.52$<br>Test of $\theta_i = \theta_j$: $Q(2) = 125.66$, $p = 0.00$<br>Test of $\theta = 0$: $z = -2.30$, $p = 0.02$

Figure 2a Associations between MVC involvement and vision impairment and vision-related intervention

For peer review only

<table>
<tr><th>Vision impairment</th><th>Association</th></tr>
<tr><td>Glaucoma</td><td>
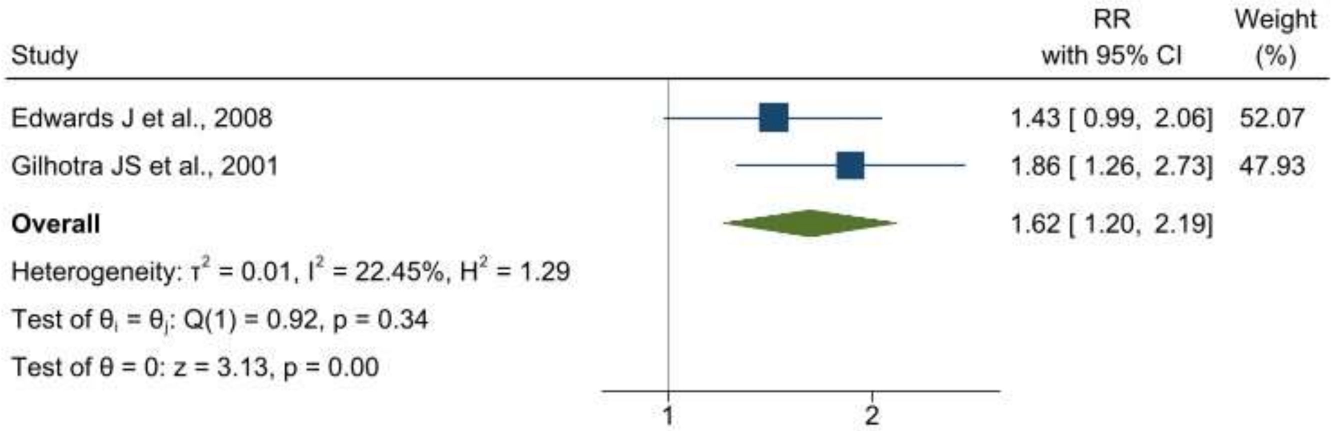

</td></tr>
<tr><td>AMD</td><td>
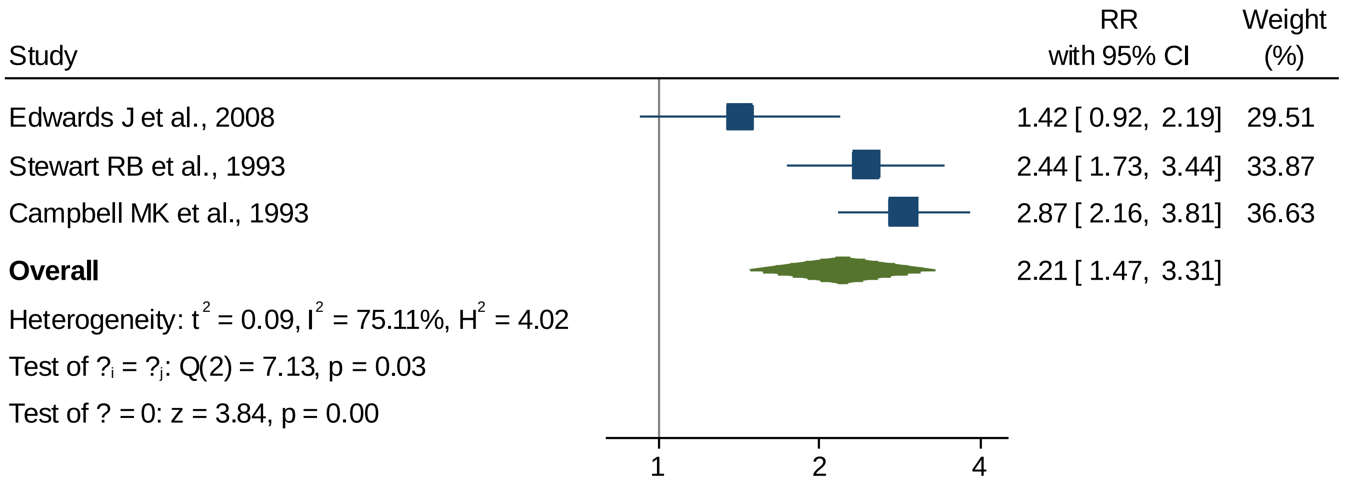

</td></tr>
<tr><td>Contrast Sensitivity</td><td></td></tr>
</table>

For peer review only - http://bmjopen.bmj.com/site/about/guidelines.xhtml

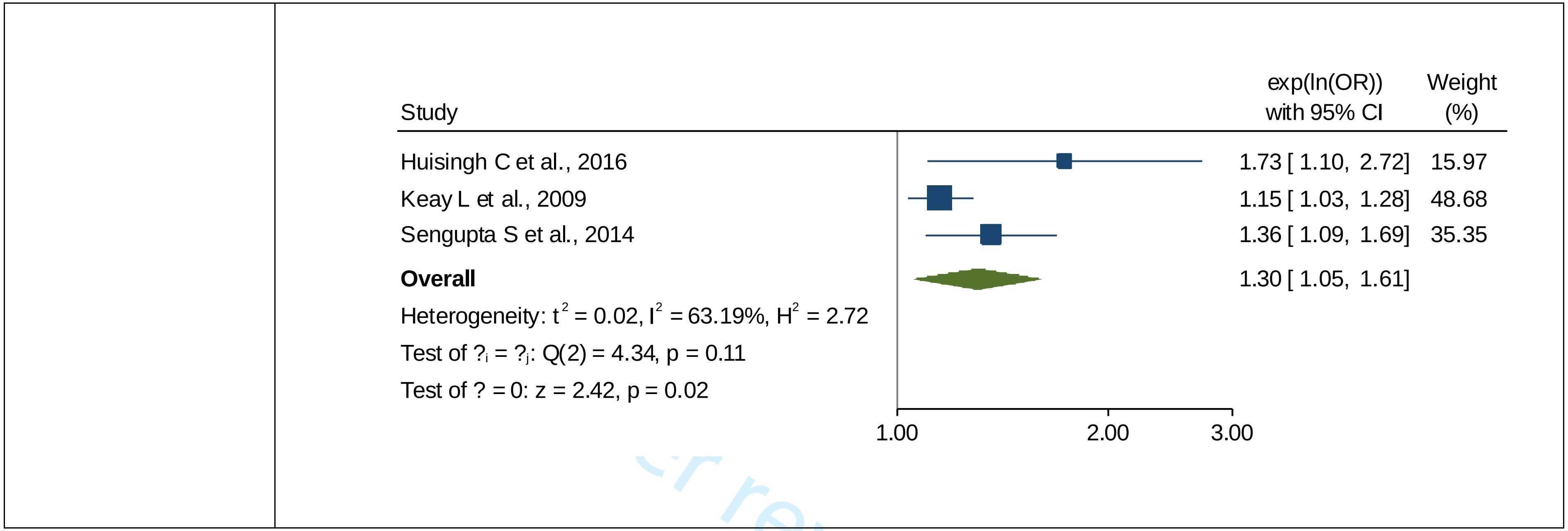

Figure 2b Associations between driving cessation and vision impairments

For peer review only

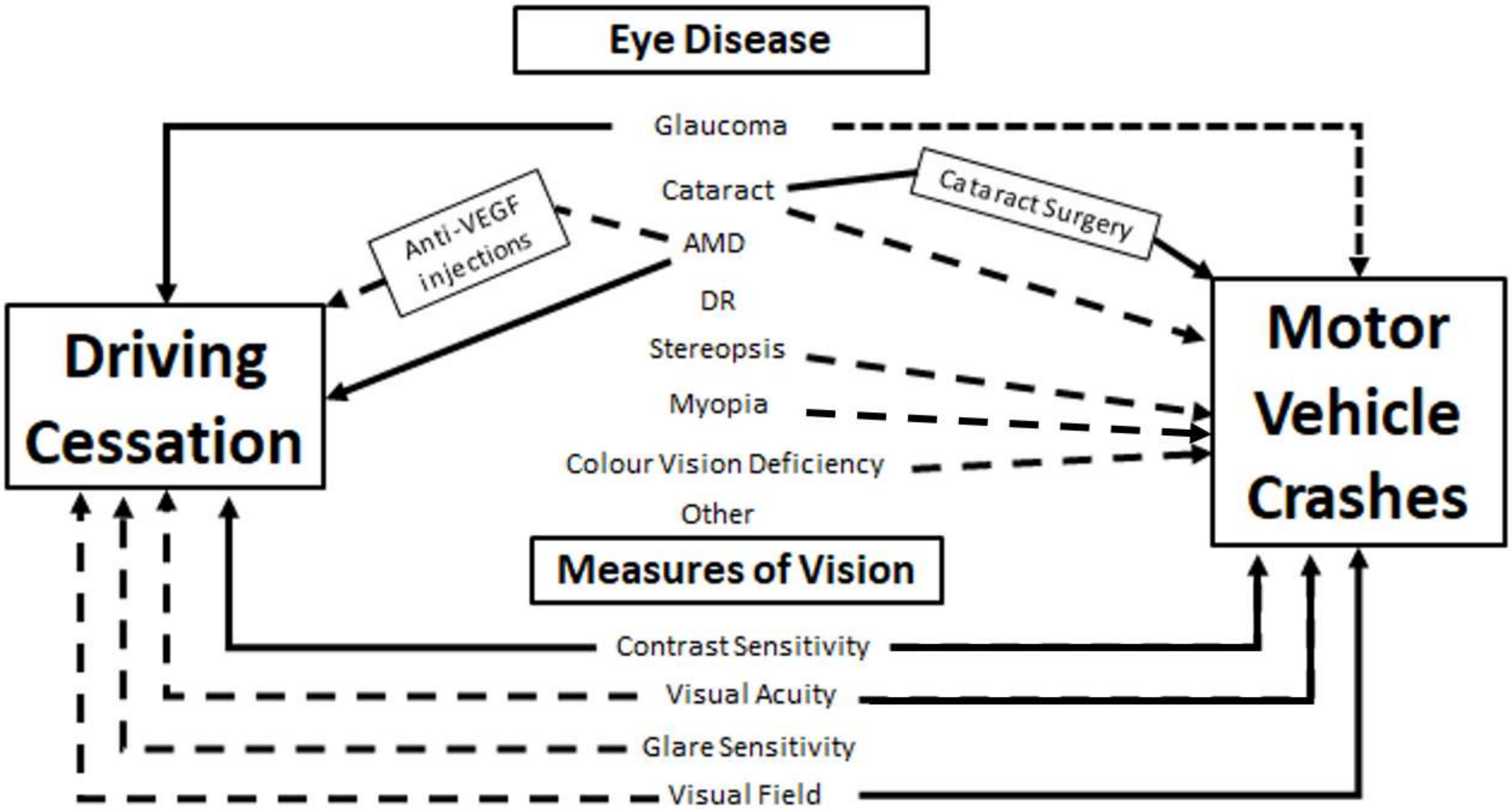

Figure 3 Network diagram illustrating strength of associations of vision impairment on motor vehicle crashes (MVC) and driving cessation found by narrative summaries and meta-analyses of available evidence

Consistent Associations

Inconclusive/ mixed

PRISMA 2020 Checklist

Section and Topic	Item #	Checklist item	Location where item is reported
**TITLE**			
Title	1	Identify the report as a systematic review.	pg.1
**ABSTRACT**			
Abstract	2	See the PRISMA 2020 for Abstracts checklist.	Appendix 1
**INTRODUCTION**			
Rationale	3	Describe the rationale for the review in the context of existing knowledge.	pg.4
Objectives	4	Provide an explicit statement of the objective(s) or question(s) the review addresses.	pg.4
**METHODS**			
Eligibility criteria	5	Specify the inclusion and exclusion criteria for the review and how studies were grouped for the syntheses.	pg.4,5, Table 1
Information sources	6	Specify all databases, registers, websites, organisations, reference lists and other sources searched or consulted to identify studies. Specify the date when each source was last searched or consulted.	pg.4
Search strategy	7	Present the full search strategies for all databases, registers and websites, including any filters and limits used.	Appendix 2
Selection process	8	Specify the methods used to decide whether a study met the inclusion criteria of the review, including how many reviewers screened each record and each report retrieved, whether they worked independently, and if applicable, details of automation tools used in the process.	pg.5
Data collection process	9	Specify the methods used to collect data from reports, including how many reviewers collected data from each report, whether they worked independently, any processes for obtaining or confirming data from study investigators, and if applicable, details of automation tools used in the process.	pg.5
Data items	10a	List and define all outcomes for which data were sought. Specify whether all results that were compatible with each outcome domain in each study were sought (e.g. for all measures, time points, analyses), and if not, the methods used to decide which results to collect.	pg.5
	10b	List and define all other variables for which data were sought (e.g. participant and intervention characteristics, funding sources). Describe any assumptions made about any missing or unclear information.	pg.5
Study risk of bias assessment	11	Specify the methods used to assess risk of bias in the included studies, including details of the tool(s) used, how many reviewers assessed each study and whether they worked independently, and if applicable, details of automation tools used in the process.	pg.5,6
Effect measures	12	Specify for each outcome the effect measure(s) (e.g. risk ratio, mean difference) used in the synthesis or presentation of results.	pg.6
Synthesis methods	13a	Describe the processes used to decide which studies were eligible for each synthesis (e.g. tabulating the study intervention characteristics and comparing against the planned groups for each synthesis (item #5)).	pg.6
	13b	Describe any methods required to prepare the data for presentation or synthesis, such as handling of missing summary statistics, or data conversions.	pg.6
	13c	Describe any methods used to tabulate or visually display results of individual studies and syntheses.	pg.6
	13d	Describe any methods used to synthesize results and provide a rationale for the choice(s). If meta-analysis was performed, describe the model(s), method(s) to identify the presence and extent of statistical heterogeneity, and software package(s) used.	pg.6
	13e	Describe any methods used to explore possible causes of heterogeneity among study results (e.g. subgroup analysis, meta-regression).	pg.6
	13f	Describe any sensitivity analyses conducted to assess robustness of the synthesized results.	pg.6
Reporting bias assessment	14	Describe any methods used to assess risk of bias due to missing results in a synthesis (arising from reporting biases).	N/A
Certainty	15	Describe any methods used to assess certainty (or confidence) in the body of evidence for an outcome.	N/A

For peer review only

For peer review only - http://bmjopen.bmj.com/site/about/guidelines.xhtml

PRISMA 2020 Checklist

Section and Topic	Item #	Checklist item	Location where item is reported
assessment			
**RESULTS**			
Study selection	16a	Describe the results of the search and selection process, from the number of records identified in the search to the number of studies included in the review, ideally using a flow diagram.	pg.6, Figure 1, Table 2a and 2b
	16b	Cite studies that might appear to meet the inclusion criteria, but which were excluded, and explain why they were excluded.	Figure 1
Study characteristics	17	Cite each included study and present its characteristics.	pg.8-11, Appendix 4a and 4b
Risk of bias in studies	18	Present assessments of risk of bias for each included study.	Appendix 3
Results of individual studies	19	For all outcomes, present, for each study: (a) summary statistics for each group (where appropriate) and (b) an effect estimate and its precision (e.g. confidence/credible interval), ideally using structured tables or plots.	pg.8-11, Appendix 4a and 4b
Results of syntheses	20a	For each synthesis, briefly summarise the characteristics and risk of bias among contributing studies.	pg.8-11, Appendix 4a and 4b
	20b	Present results of all statistical syntheses conducted. If meta-analysis was done, present for each the summary estimate and its precision (e.g. confidence/credible interval) and measures of statistical heterogeneity. If comparing groups, describe the direction of the effect.	pg.8-11, Appendix 4a and 4b
	20c	Present results of all investigations of possible causes of heterogeneity among study results.	pg.8-11, Figure 2a-b
	20d	Present results of all sensitivity analyses conducted to assess the robustness of the synthesized results.	N/A
Reporting biases	21	Present assessments of risk of bias due to missing results (arising from reporting biases) for each synthesis assessed.	N/A
Certainty of evidence	22	Present assessments of certainty (or confidence) in the body of evidence for each outcome assessed.	N/A
**DISCUSSION**			
Discussion	23a	Provide a general interpretation of the results in the context of other evidence.	pg.11-13
	23b	Discuss any limitations of the evidence included in the review.	pg.11-13
	23c	Discuss any limitations of the review processes used.	pg.12,13
	23d	Discuss implications of the results for practice, policy, and future research.	pg.11-13
**OTHER INFORMATION**			
Registration and protocol	24a	Provide registration information for the review, including register name and registration number, or state that the review was not registered.	pg.2
	24b	Indicate where the review protocol can be accessed, or state that a protocol was not prepared.	pg.4
	24c	Describe and explain any amendments to information provided at registration or in the protocol.	N/A
Support	25	Describe sources of financial or non-financial support for the review, and the role of the funders or sponsors in the review.	pg.14
Competing	26	Declare any competing interests of review authors.	pg.14

For peer review only

PRISMA 2020 Checklist

Section and Topic	Item #	Checklist item	Location where item is reported
interests			
Availability of data, code and other materials	27	Report which of the following are publicly available and where they can be found: template data collection forms; data extracted from included studies; data used for all analyses; analytic code; any other materials used in the review.	pg.13

PRISMA ABSTRACT CHECKLIST

Section and Topic	Item #	Checklist item	Reported (Yes/No)
**TITLE**			
Title	1	Identify the report as a systematic review.	Yes
**BACKGROUND**			
Objectives	2	Provide an explicit statement of the main objective(s) or question(s) the review addresses.	Yes
**METHODS**			
Eligibility criteria	3	Specify the inclusion and exclusion criteria for the review.	Yes
Information sources	4	Specify the information sources (e.g. databases, registers) used to identify studies and the date when each was last searched.	Yes
Risk of bias	5	Specify the methods used to assess risk of bias in the included studies.	Yes
Synthesis of results	6	Specify the methods used to present and synthesise results.	Yes
**RESULTS**			
Included studies	7	Give the total number of included studies and participants and summarise relevant characteristics of studies.	Yes
Synthesis of results	8	Present results for main outcomes, preferably indicating the number of included studies and participants for each. If meta-analysis was done, report the summary estimate and confidence/credible interval. If comparing groups, indicate the direction of the effect (i.e. which group is favoured).	Yes
**DISCUSSION**			
Limitations of evidence	9	Provide a brief summary of the limitations of the evidence included in the review (e.g. study risk of bias, inconsistency and imprecision).	Yes
Interpretation	10	Provide a general interpretation of the results and important implications.	Yes
**OTHER**			
Funding	11	Specify the primary source of funding for the review.	Yes
Registration	12	Provide the register name and registration number.	Yes

For peer review only

PRISMA 2020 Checklist

For peer review only

Appendix 2 Complete search strategy for review. While search terms were included for driving performance, driving scores and errors, the studies with outcome measures of driving performance was outside of the scope of this current manuscript and are reported elsewhere.

MEDLINE (OVID) search strategy

1. exp Eye Diseases/
2. exp Cataract Extraction/
3. Lens Implantation, Intraocular/
4. Lenses, Intraocular/
5. cataract$.tw.
6. ((intraocular or intra ocular) adj3 lens$).tw.
7. (IOL or IOLs).tw.
8. Vision Tests/
9. Visual Acuity/
10. exp Refractive Errors/
11. Visual Fields/
12. Visual Field Tests/
13. Contrast Sensitivity/
14. Depth Perception/
15. (visual adj2 (acuit$ or field$)).tw.
16. contrast sensitivity.tw.
17. (depth perception or stereopsis).tw.
18. ((impair$ or decreas$ or declin$) adj3 (vision or visual$ or sight$)).tw.
19. (improv$ adj3 (vision or visual$ or sight$)).tw.
20. ((visual or vision) adj2 function$).tw.
21. exp Vision, Ocular/
22. Vision Screening/
23. or/1-22
24. Mass Screening/
25. ((eye$ or sight or vision or visual$) adj2 (test$ or screen$ or exam$ or diagnos$ or assess$)).tw
26. 24 and 25
27. 23 or 26
28. exp Motor Vehicles/
29. exp Automobile Driving/
30. Accidents, Traffic/
31. (driver$ or driving).tw.
32. (automobile$ or car or cars or vehicle$).tw.
33. (motoring or motorcar or "motor car" or "motor cars").tw.
34. crash$.tw.
35. ((road or traffic) adj2 injur$).tw.
36. ((road or traffic or motor) adj2 (accident$ or incident$)).tw.
37. ((road or traffic or motor) adj2 collision$).tw.
38. or/28-37
39. epidemiologic studies/ or case-control studies/ or cohort studies/ or observational study/ or follow-up studies/ or longitudinal studies/ or prospective studies/ or retrospective studies/ or controlled before-after studies/ or cross-sectional studies/ or historically controlled study/ or interrupted time series analysis/
40. epidemiologic methods/ or focus groups/ or interviews as topic/ or exp "surveys and questionnaires"/

For peer review only

41. epidemiologic research design/ or control groups/ or cross-over studies/ or double-blind method/ or meta-analysis as topic/ or network meta-analysis/ or random allocation/ or single-blind method/
42. epidemiologic methods/ or clinical trials as topic/ or feasibility studies/ or multicenter studies as topic/ or pilot projects/ or sampling studies/ or twin studies as topic/
43. randomized controlled trial/ or controlled clinical trials as topic/ or randomized controlled trials as topic/
44. comparative study/ or evaluation studies/ or meta-analysis/ or review/ or multicenter study/ or "systematic review"/ or validation studies/
45. health surveys/
46. outcome assessment, health care/
47. risk factors/
48. self report/
49. (population or cohort or observation$ or intervention$ or prospective or retrospective or comparative).tw.
50. (questionnaire$ or survey$).tw.
51. (randomized or randomised or randomly or RCT).tw.
52. (systematic review or meta-analysis).tw.
53. (before adj2 after).tw.
54. (case$ adj2 control$).tw.
55. (cross adj1 section$).tw.
56. or/39-55
57. 27 and 38
58. 56 and 57
59. vehicle-controlled.tw.
60. (vehicle adj3 inject$).tw.
61. 59 or 60
62. 58 not 61
63. (animal$ or mouse or mice$ or dog or canine or rat or rats or primate$).ti.
64. (dry eye or cell$ or mutation$ or genes or genome or sequencing).ti.
65. or/63-64
66. 62 not 65
67. limit 66 to english language
68. exp case reports/
69. (case adj2 report$).tw.
70. 68 or 69
71. 67 not 70
72. limit 71 to (editorial or letter)
73. 71 not 72

EMBASE Search Strategy

1. exp eye disease/
2. exp cataract extraction/
3. lens implantation/
4. lens implant/
5. cataract$.tw.
6. ((intraocular or intra ocular) adj3 lens$).tw.
7. (IOL or IOLs).tw.
8. vision test/
9. visual acuity/

For peer review only

10. refractive error/
11. visual field/
12. perimetry/
13. contrast sensitivity/
14. depth perception/
15. (visual adj2 (acuit$ or field$)).tw.
16. contrast sensitivity.tw.
17. (depth perception or stereopsis).tw.
18. ((impair$ or decreas$ or declin$) adj3 (vision or visual$ or sight$)).tw.
19. (improv$ adj3 (vision or visual$ or sight$)).tw.
20. ((visual or vision) adj2 function$).tw.
21. vision/
22. or/1-21
23. mass screening/
24. ((eye$ or sight or vision or visual$) adj2 (test$ or screen$ or exam$ or diagnos$ or assess$)).tw.
25. 23 and 24
26. 22 or 25
27. exp car driving/
28. exp motor vehicle/
29. traffic accident/
30. (driver$ or driving).tw.
31. (automobile$ or car or cars or vehicle$).tw.
32. (motoring or motorcar or "motor car" or "motor cars").tw.
33. crash$.tw.
34. ((road or traffic) adj2 injur$).tw.
35. ((road or traffic or motor) adj2 (accident$ or incident$)).tw.
36. ((road or traffic or motor) adj2 collision$).tw.
37. or/27-36
38. study design/
39. controlled clinical trial/
40. case control study/
41. cohort analysis/
42. observational study/
43. follow up/
44. longitudinal study/
45. prospective study/
46. retrospective study/
47. epidemiology/
48. cross-sectional study/
49. control group/
50. crossover procedure/
51. "meta analysis (topic)"/
52. network meta-analysis/
53. randomization/
54. single blind procedure/
55. double blind procedure/
56. "clinical trial (topic)"/
57. "controlled clinical trial (topic)"/
58. "randomized controlled trial (topic)"/
59. "multicenter study (topic)"/
60. feasibility study/

For peer review only

61. pilot study/
62. comparative study/
63. evaluation study/
64. multicenter study/
65. randomized controlled trial/
66. meta analysis/
67. "systematic review"/
68. validation study/
69. interview/
70. questionnaire/
71. outcome assessment/
72. "systematic review (topic)"/
73. health survey/
74. risk factor/
75. self report/
76. evidence based practice/
77. (population or cohort or observation$ or intervention$ or prospective or retrospective or comparative).tw.
78. (questionnaire$ or survey$).tw.
79. (randomized or randomised or randomly or RCT).tw.
80. (systematic review or meta-analysis).tw.
81. (before adj2 after).tw.
82. (case$ adj2 control$).tw.
83. (cross adj1 section$).tw.
84. or/38-83
85. 26 and 37
86. 84 and 85
87. vehicle-controlled.tw.
88. (vehicle adj3 inject$).tw.
89. or/87-88
90. 86 not 89
91. (animal$ or mouse or mice$ or dog or canine or rat or rats or primate$).ti.
92. (dry eye or cell$ or mutation$ or genes or genome or sequencing).ti.
93. or/91-92
94. 90 not 93
95. limit 94 to conference abstract status
96. 94 not 95
97. limit 96 to english language
98. exp case report/
99. (case adj2 report$).tw.
100. or/98-99
101. 97 not 100
102. limit 101 to (conference paper or "conference review" or editorial or letter or note)
103. 101 not 102

GLOBAL HEALTH Search Strategy

1. exp eye diseases/
2. exp vision disorders/
3. cataract$.tw.
4. ((intraocular or intra ocular) adj3 lens$).tw.
5. (IOL or IOLs).tw.

For peer review only

6. (visual adj2 (acuit$ or field$)).tw.
7. contrast sensitivity.tw.
8. (depth perception or stereopsis).tw.
9. ((impair$ or decreas$ or declin$) adj3 (vision or visual$ or sight$)).tw.
10. (improv$ adj3 (vision or visual$ or sight$)).tw.
11. ((visual or vision) adj2 function$).tw.
12. ((eye$ or sight or vision or visual$) adj2 (test$ or screen$ or exam$ or diagnos$ or assess$)).tw.
13. or/1-12
14. drivers/
15. vehicles/
16. motor cars/
17. traffic/
18. traffic accidents/
19. (driver$ or driving).tw.
20. (automobile$ or car or cars or vehicle$).tw.
21. (motoring or motorcar or "motor car" or "motor cars").tw.
22. crash$.tw.
23. ((road or traffic) adj2 injur$).tw.
24. ((road or traffic or motor) adj2 (accident$ or incident$)).tw.
25. ((road or traffic or motor) adj2 collision$).tw.
26. or/14-25
27. cohort studies/
28. case-control studies/
29. longitudinal studies/
30. retrospective studies/
31. epidemiology/
32. exp clinical trials/
33. randomized controlled trials/
34. feasibility studies/
35. pilot projects/
36. meta-analysis/
37. systematic reviews/
38. reviews/
39. questionnaires/
40. surveys/
41. epidemiological surveys/
42. risk factors/
43. (population or cohort or observation$ or intervention$ or prospective or retrospective or comparative).tw.
44. (questionnaire$ or survey$).tw.
45. (randomized or randomised or randomly or RCT).tw.
46. (systematic review or meta-analysis).tw.
47. (before adj2 after).tw.
48. (case$ adj2 control$).tw.
49. (cross adj1 section$).tw.
50. or/27-49
51. 13 and 26
52. 50 and 51
53. (animal$ or mouse or mice$ or dog or canine or rat or rats or primate$).ti.
54. (dry eye or cell$ or mutation$ or genes or genome or sequencing).ti.
55. 53 or 54

For peer review only

56. 52 not 55
57. limit 56 to english language
58. case reports/
59. (case adj2 report$).tw.
60. 58 or 59
61. 57 not 60
62. limit 61 to (conference or conference paper or conference proceedings or correspondence or editorial or thesis)
63. 61 not 62

For peer review only

Appendix 3 Risk of Bias Assessment for all Included Studies

Analytical Cross-Sectional Study

Citation	Q1 (SB)	Q2 (SB)	Q3 (DB)	Q4 (C)	Q5 (C)	Q6(C)	Q7 (DB)	Q8 (V)	Risk of Bias*
**High Income Countries**									
Adler G, et al. 2005.	Y	Y	N	Y	Y	Y	Y	Y	L
Alvarez-Peregrina C et al., 2021	N	N	N	N	N	N	Y	N	H
Ball K, et al. 1993.	Y	N	U	U	N	N	U	Y	H
Cohen Y, et al. 2007.	Y	Y	Y	Y	N	N	Y	Y	M
Crizzle AM et al., 2020	Y	Y	U	U	N	N	Y	Y	M
Cross JM, et al. 2009.	U	U	Y	N	N	U	Y	Y	H
DeCarlo DK, et al. 2003.	Y	Y	N	U	U	U	Y	Y	M
Edwards JD, et al. 2008.	Y	Y	Y	N	Y	Y	Y	Y	L
Garre-Olmo J, et al. 2009.	Y	Y	Y	U	Y	Y	Y	Y	M
Gilhotra JS, et al. 2001.	Y	Y	Y	Y	Y	Y	Y	Y	L
Hajek A, et al. 2019.	Y	Y	N	N	Y	Y	Y	Y	M

For peer review only - http://bmjopen.bmj.com/site/about/guidelines.xhtml

Huisingh C, et al. 2015.	Y	Y	Y	Y	Y	Y	Y	Y	L
Ivers RQ, et al. 1999.	N	N	Y	N	Y	Y	N	Y	M
Kaleem MA et al., 2021	Y	Y	Y	Y	N	N	Y	N	M
Keay L, et al. 2016.	Y	Y	Y	Y	Y	Y	Y	Y	L
Kwon M, et al. 2016.	Y	Y	Y	Y	Y	Y	Y	Y	L
Levecq L, et al. 2013.	Y	Y	Y	Y	N	N	Y	Y	M
MacLeod KE, et al. 2014.	Y	Y	Y	Y	Y	Y	Y	Y	L
Marottoli RA, et al. 1993.	Y	Y	N	Y	U	Y	Y	Y	M
Moon SH & Park K et al., 2020	Y	Y	N	N	N	N	Y	Y	M
Ono T, et al. 2015.	Y	Y	Y	Y	Y	Y	N	Y	L
Owsley C, et al. 2001.	Y	Y	Y	Y	Y	Y	Y	Y	L
Owsley C, et al. 2020.	Y	Y	Y	Y	Y	Y	Y	Y	L
Ramulu PY, et al. 2009.	Y	Y	Y	Y	Y	Y	Y	Y	L

For peer review only - http://bmjopen.bmj.com/site/about/guidelines.xhtml

Robinson JL et al., 2021	Y	Y	Y	Y	N	N	Y	N	M
Ross LA, et al. 2009.	Y	Y	Y	Y	N	N	Y	Y	M
Runge JW. 2000.	N	N	U	U	U	U	Y	Y	H
Segal-Gidan F, et al. 2010.	Y	Y	Y	Y	Y	Y	Y	Y	L
Sengupta S, et al. 2014.	Y	Y	Y	Y	Y	Y	Y	Y	L
Stafford WR. 1981.	N	N	Y	U	N	N	Y	Y	H
Stewart RB, et al. 1993.	Y	Y	N	N	Y	Y	Y	Y	M
Swain TA et al., 2021	Y	Y	Y	Y	Y	Y	Y	Y	L
Tam ALC, et al. 2018.	Y	Y	Y	Y	N	N/A	Y	Y	M
Tanabe S, et al. 2011.	Y	Y	Y	Y	Y	Y	N	Y	L
van Landingham SW, et al. 2013.	Y	Y	Y	Y	U	U	Y	Y	M
Wedenoja J et al., 2021	Y	Y	U	U	N	N	Y	N	H
Yuki K, et al. 2014.	Y	Y	Y	Y	N	N	N	Y	M

For peer review only - http://bmjopen.bmj.com/site/about/guidelines.xhtml

Zebardast N, et al. 2015.	Y	Y	Y	Y	Y	Y	Y	Y	L
**Low Middle Income Countries**									
Abebe Y, et al. 2002.	Y	Y	Y	N	N	N	N	Y	M
Abraham EG, et al. 2010.	Y	N	N	N	N	N	N	Y	H
Adekoya BJ, et al. 2009.	Y	Y	Y	Y	N	N	N	Y	M
Ahmed M et al., 2021	N	N	Y	Y	Y	Y	Y	Y	M
Bekibele CO, et al. 2007.	N	Y	Y	Y	N	N	N	Y	M
Biza M, et al. 2013.	Y	Y	Y	Y	N	N	N	Y	M
Boadi-Kusi SB, et al. 2016.	Y	Y	N	Y	N	N	N	U	H
Emerole CG, et al. 2013.	N	N	Y	Y	N	N	N	N	H
Humphriss D. 1987.	Y	N	N	N	N	N/A	U	U	H
Isawumi MA, et al. 2011.	Y	Y	U	Y	N	N	N	U	H
Ogbonnaya CE, et al. 2018.	Y	Y	Y	Y	N	N	N	Y	M

For peer review only - http://bmjopen.bmj.com/site/about/guidelines.xhtml

Oladehinde MK, et al. 2007.	N	N	Y	Y	N	N	N	Y	H
Ovenseri-Ogomo G, et al. 2011.	Y	Y	Y	Y	N	N/A	N	Y	M
Pepple G, et al. 2014.	Y	Y	Y	N	N	N	N	Y	M
Vofo BN et al., 2021	Y	Y	Y	Y	N	N	Y	N	M

*Risk of bias scores: high (1-3), medium (4-6), and low (7-8)

SB= selection bias, DB= detection bias, C= confounding, V= validity

Case Control Study

**Citation**	**Q1 (SB)**	**Q2 (SB)**	**Q3 (SB)**	**Q4 (DB)**	**Q5 (PB)**	**Q6 (C)**	**Q7 (C)**	**Q8 (PB)**	**Q9 (V)**	**Q10 (V)**	**Risk of Bias***
**High Income Countries**											
Campbell MK, et al. 1993.	Y	U	Y	N	U	Y	Y	Y	U	U	M
Gallo JJ, et al. 1999.	Y	N	Y	U	U	Y	Y	Y	N	Y	M
Gresset JA, et al. 1994.	N	Y	Y	Y	Y	Y	Y	Y	Y	Y	L
Gresset JA, et al. 1994.	U	Y	Y	U	U	N	N	Y	Y	Y	M
McCloskey LW, et al. 1994.	Y	Y	Y	Y	Y	U	U	Y	Y	Y	M

For peer review only

McGwin G, et al. 2000.	Y	Y	Y	N	Y	Y	Y	Y	Y	Y	L
McGwin G, et al. 2004.	Y	U	Y	Y	Y	Y	Y	Y	Y	Y	L
McGwin G, et al. 2005.	Y	Y	Y	Y	Y	Y	Y	Y	Y	Y	L
Owsley C, et al. 1998.	Y	Y	Y	Y	Y	N	N	Y	Y	Y	M
Sims RV, et al. 1998.	Y	U	Y	Y	Y	N	N	N	Y	Y	M
Szlyk JP, et al. 1995.	Y	Y	Y	Y	Y	N	N	Y	Y	Y	M
Wood JM, et al. 2018.	Y	Y	N	Y	Y	Y	Y	Y	U	Y	M
Wood JM, et al. 2016.	Y	Y	Y	Y	Y	Y	Y	Y	U	Y	L
Owsley C, et al. 1999.	Y	Y	Y	Y	Y	Y	Y	Y	Y	Y	L
**Low Middle Income Countries**											
Deshmukh AV, et al. 2019.	Y	N	Y	Y	Y	N	N	Y	U	N	M

*Risk of bias scores: high (1-4), medium (5-8), and low (9-10)

SB= selection bias, DB= detection bias, PB = performance bias, C= confounding, V= validity

For peer review only

Case Series

Citation	Q1 (SB)	Q2 (DB)	Q3 (DB)	Q4 (SB)	Q5 (SB)	Q6 (SB)	Q7 (SB)	Q8 (AtB)	Q9 (SB)	Q10 (V)	Risk of Bias*
**High Income Countries**											
Goh YW, et al. 2011	Y	Y	Y	Y	Y	Y	Y	Y	N	Y	L

*Risk of bias scores: high (1-4), medium (5-8), and low (9-10)

SB= selection bias, DB= detection bias, AtB= attrition bias, V= validity

Cohort Study

Citation	Q1 (SB)	Q2 (PB)	Q3 (DB)	Q4 (C)	Q5 (C)	Q6 (SB)	Q7 (DB)	Q8 (V)	Q9 (AtB)	Q10 (AtB)	Q11 (V)	Risk of Bias*
**High Income Countries**												
Anstey KJ, et al. 2006.	Y	Y	Y	Y	Y	Y	Y	Y	Y	Y	Y	L
Baker JM, et al. 2019.	N	Y	N	Y	Y	Y	Y	Y	Y	N/A	Y	M
Fishman GA et al., 1981	Y	U	Y	Y	Y	N	N	U	N	N	N	H
Freeman EE, et al. 2005.	Y	Y	Y	Y	Y	Y	Y	Y	Y	N	Y	L
Green KA, et al. 2013.	Y	Y	Y	Y	Y	U	Y	Y	Y	N	Y	M

For peer review only

Haymes SA, et al. 2007.	Y	Y	Y	Y	Y	N/A	Y	Y	Y	N/A	Y	M
Huisingh C, et al. 2017.	Y	Y	Y	Y	Y	Y	Y	Y	Y	U	Y	L
Huisingh C, et al. 2016.	Y	Y	Y	Y	Y	Y	Y	Y	N	N	Y	M
Janz NK, et al. 2009.	Y	Y	Y	Y	Y	U	Y	Y	N	N	Y	M
Keay L, et al. 2009.	Y	Y	Y	Y	Y	Y	Y	Y	Y	Y	Y	L
Keeffe JE, et al. 2002.	Y	Y	Y	N	N/A	Y	N	Y	Y	U	U	M
Kristalovich L, et al. 2019.	U	Y	Y	N	N	Y	U	Y	N/A	N/A	Y	M
Maag U, et al. 1997.	Y	N	U	Y	Y	N	Y	Y	Y	N/A	Y	M
Margolis KL, et al. 2002.	Y	Y	Y	Y	Y	Y	Y	Y	Y	U	Y	L
McGwin G, et al. 2015.	Y	Y	Y	Y	Y	Y	Y	Y	Y	U	Y	L
McGwin G, et al. 2013.	Y	Y	Y	N	N/A	Y	Y	Y	U	U	Y	M
Meuleners LB,et al. 2019.	Y	Y	Y	Y	Y	Y	Y	Y	U	Y	Y	L

For peer review only - http://bmjopen.bmj.com/site/about/guidelines.xhtml

Meuleners LB, et al. 2012.	Y	Y	Y	Y	Y	Y	Y	Y	Y	N/A	Y	L
Meuleners LB, et al. 2012.	Y	Y	Y	Y	Y	Y	Y	Y	U	U	Y	M
Monestam E, et al. 2005.	Y	Y	Y	N	N	Y	Y	Y	N	N	U	M
Monestam E, et al. 1997.	Y	U	Y	N	N	Y	Y	Y	Y	N	Y	M
Naredo Turrado J, et al. 2020.	Y	Y	Y	Y	Y	U	Y	Y	Y	U	Y	M
Owsley C, et al. 2002.	Y	Y	Y	Y	Y	Y	Y	Y	Y	U	Y	L
Rahi JS, et al. 2006.	Y	Y	U	Y	Y	Y	N	Y	N	N	Y	M
Rubin GS, et al. 2007.	Y	Y	Y	Y	Y	Y	Y	Y	Y	N	Y	L
Schlenker MB, et al. 2018.	Y	Y	Y	Y	Y	Y	Y	Y	Y	U	Y	L
Swain TA et al., 2021	Y	Y	Y	Y	Y	Y	Y	Y	Y	U	Y	L
Takahashi A, et al. 2018.	U	Y	Y	Y	Y	Y	Y	Y	N	N	Y	M
Yuki K, et al. 2016.	Y	Y	Y	N	N/A	Y	N	N	Y	U	N	M

*Risk of bias scores: high (1-4), medium (5-9), and low (10-11)

SB= selection bias, PB= performance bias, DB= detection bias, C= confounding, AtB= attrition bias, V= validity

For peer review only

Systematic Reviews

Citation	Q1 (SB)	Q2 (SB)	Q3 (SB)	Q4 (SB)	Q5 (InB)	Q6 (InB)	Q7 (InB)	Q8 (C)	Q9 (PubB)	Q10 (V)	Q11 (V)	Risk of Bias
**High Income Countries**												
Piyasena P et al., 2021	Y	Y	Y	Y	Y	U	Y	Y	Y	Y	Y	L

Risk of bias scores: high (1-4), medium (5-9), and low (10-11)

SB= selection bias, InB= information bias, C= confounding, PubB= publication bias, V= validity

Randomised Controlled Trials

Citation	Q1 (AlB)	Q2 (PB)	Q3 (SB)	Q4 (PB)	Q5 (PB)	Q6 (AlB)	Q7 (C)	Q8 (AtB)	Q9 (V)	Q10 (DB)	Q11 (DB)	Q12 (V)	Q13 (V)	Risk of Bias
**High Income Countries**														
Bressler NM, et al. 2013.	Y	Y	Y	Y	Y	U	Y	Y	Y	Y	Y	Y	Y	L
Bressler NM, et al. 2016.	Y	Y	Y	Y	Y	Y	Y	Y	Y	Y	Y	Y	Y	L

*Risk of bias scores: high (1-6), medium (7-11), and low (12-13)

AlB = allocation bias, SB= selection bias, PB= performance bias, C= confounding, DB= detection bias, AtB= attrition bias, V= validity

For peer review only - http://bmjopen.bmj.com/site/about/guidelines.xhtml

Appendix 4a Raw data tables and additional narrative summaries of papers on motor vehicle crashes

Table 4a(i) All studies (n=16) on glaucoma and Motor Vehicle Crashes (MVC). Of the 16 studies, 5 studies were suitable for meta-analysis on associations with any MVC involvement and 2 studies on associations with at-fault MVC involvement

Additional Narrative Summary:
Associations between glaucoma and MVCs were mixed in the studies identified. Even though seven controlled studies found glaucoma to increase the odds of any, injurious, and at-fault MVC involvement, two studies found crash involvement to halve in drivers with glaucoma. Only one study looked at not-at-fault crashes, but found no associations (OR 1 (95% CI 0.4-2.5)). Drivers with more severe glaucoma, irrespective of whether it was in the better or worse eye, were involved in more MVCs and also had greater odds of any crash and at-fault crash involvement compared to drivers without glaucoma and drivers with mild glaucoma.

**Author and Year**	**Study Design**	**Participants/ Sample Size**	**Mean Age**	**Country**	**VI Definition**	**Comparator**	**Outcome Measure (OR, RR, HR, etc?)**	**Effect Measure (with 95% CI) + any description of results (if appropriate**
**Included in Meta-analysis (any MVC involvement)**								
Cross JM et al., 2009	Cross-sectional	3158 (249/2909)	71.9	USA	Self-reported physician diagnosed	Drivers without glaucoma	RR (rate ratio)	**Any MVC**: 1.18 (0.81, 1.72)
Haymes S et al., 2007	Retrospective Cohort	95 (48/47)	69	Canada	Diagnosis from glaucoma specialist, glaucomatous optic disc damage and corresponding visual field damage	Drivers without glaucoma	OR (logistic regression)	**Any MVC**: 6.62 (1.4, 31.23)
Kwon M et al., 2016	Cross-sectional	1899 (206/1693)	age, no.: 70-79 years =	USA	Physician diagnosed	Drivers without glaucoma	RR (rate ratio)	**Any MVC involvement**: 1.65 (1.2, 2.28)

			1358, 80-89 years = 502, 90-98 years = 39					
Naredo Turrado J et al., 2020	Prospective Cohort	11670 (525/11145)	62.4	France	Self-reported physician diagnosed	Drivers without glaucoma	OR	**Any MVC**: 0.93 (0.72, 1.22)
McGwin G Jr et al., 2004	Case Control	691 (576/115)	69.2	USA	ICD-9 codes 365.1 and 265.3	Drivers without glaucoma	RR (relative risk)	**Any MVC**: 0.58 (0.4, 0.83)
**Included in Meta-analysis (at-fault MVC involvement)**								
Cross JM et al., 2009	Cross-sectional	3158 (249/2909)	71.9	USA	Self-reported physician diagnosed	Drivers without glaucoma	RR (rate ratio)	**At-fault MVC**: 0.91 (0.48, 1.72)
Haymes S et al., 2007	Retrospective Cohort	95 (48/47)	69	Canada	Diagnosis from glaucoma specialist, glaucomatous optic disc damage and corresponding visual field damage	Drivers without glaucoma	OR (logistic regression)	**At-fault MVC**: 12.44 (1.08, 143.99)
McGwin G Jr et al., 2004	Case Control	691 (576/115)	69.2	USA	ICD-9 codes 365.1 and 265.3	Drivers without glaucoma	RR (relative risk)	**At-fault MVC**: 0.99 (0.54, 1.8)
**Included in Narrative Summaries Only – High Income Countries**								
Adler G et al., 2004	Cross-sectional	199 (52/147)	71.3	USA	Open-or closed-angle glaucoma	Drivers without glaucoma	Prevalence (%)	25% (13/52) of drivers with glaucoma had been in an MVC compared to

								25.9% (38/147) of drivers without glaucoma (p= 0.86).
Cross JM et al., 2009	Cross-sectional	3158 (249/2909)	71.9	USA	Self-reported physician diagnosed	Drivers without glaucoma	RR (rate ratio)	**Injurious MVC**: 0.63 (0.19, 2.06)
Haymes S et al., 2007	Retrospective Cohort	95 (48/47)	69	Canada	Diagnosis from glaucoma specialist, glaucomatous optic disc damage and corresponding visual field damage	Drivers without glaucoma	OR (logistic regression)	**Any MVC (state-reported)**: 3.21 (0.72, 14.27)
								**At-fault MVC (state-reported)**: 7.21 (0.46, 113.4)
							Prevalence (%)	27% (11/400 of drivers with glaucoma had been involved in an MVC compared to 7% (3/44) in drivers without glaucoma.<br><br>20% (8/40) of drivers with glaucoma were at-fault in an MVC compared to 2% (1/44) in drivers without glaucoma.

For peer review only

Kwon M et al., 2016	Cross-sectional	1899 (206/1693)	age, no.: 70-79 years = 1358, 80-89 years = 502, 90-98 years = 39	USA	Physician diagnosed	Drivers without glaucoma	Prevalence (%)	18% (37/206) of drivers with glaucoma were at-fault in a crash compared to 13% (219/1693) of drivers without glaucoma.
McCloskey L et al., 1994	Case Control	683 (42/641)	age, no.: 65-69 years = 264, 70-74 years = 195, 75-79 years = 138, 80+ years = 86	USA	Physician diagnosed (hospital data)	Age-matched drivers with glaucoma who have not been injured in a police-reported MVC in the same calendar year as their matched case.	RR (relative risk)	**Injurious MVC**: 1.5 (0.8, 2.9)*
							Prevalence (%)	7.7% (18/234) of all drivers who had an injurious crash also had glaucoma.
McGwin G Jr et al., 2000	Case Control	901 (447/454)	N/A	USA	Self-reported physician diagnosed	Not-at-fault drivers involved in crashes, without glaucoma	OR	**Not at-fault MVC**: 1 (0.4, 2.5)
							Prevalence (%)	5.2% (10/198) of not-at-fault crashes involved drivers with

For peer review only

								glaucoma. 6.9% (17/249) 0f at-fault crashes involved drivers with glaucoma.
McGwin G Jr et al., 2004	Case Control	691 (576/115)	69.2	USA	ICD-9 codes 365.1 and 265.3	Drivers without glaucoma	RR (relative risk)	**All MVC per person-time**: 0.57 (0.39, 0.83)
								**At-fault MVC per person-time**: 1.02 (0.56, 1.87)
							Prevalence (%)	27% (153/576) of drivers with glaucoma were involved in an MVC compared to 37% (42/115) of drivers without glaucoma.<br><br>15% (87/576) of drivers with glaucoma were at-fault in a crash compared to 12% (14/115) of drivers without glaucoma.
McGwin G Jr et al., 2005	Case Control	240 (120/120)	72.9	USA	ICD-9 codes 365.1 and 265.3, given an AGIS score	Drivers with glaucoma who have not had	OR	**At-fault MVC**: 1.7(0.7, 3.7)

For peer review only

					from visual fields examinations – **mild defect in better eye**	an MVC between 1994 and 2000.		
					ICD-9 codes 365.1 and 265.3, given an AGIS score from visual fields examinations **– moderate defect in better eye**		OR	**At-fault MVC:** 2 (0.7, 5.4)
					ICD-9 codes 365.1 and 265.3, given an AGIS score from visual fields examinations – **severe defect in better eye**		OR	**At-fault MVC:** 4.2 (0.9, 15.3)
					ICD-9 codes 365.1 and 265.3, given an AGIS score from visual fields examinations – **mild defect in worse eye**		OR	**At-fault MVC:** 1.9 (0.6, 6.1)
					ICD-9 codes 365.1 and 265.3, given an AGIS score from visual fields examinations – **moderate defect in worse eye**		OR	**At-fault MVC:** 4.2 (1.2, 15)

For peer review only

					ICD-9 codes 365.1 and 265.3, given an AGIS score from visual fields examinations – **severe defect in worse eye**		OR	**At-fault MVC:** 9 (2.4, 33.2)
					ICD-9 codes 365.1 and 265.3, given an AGIS score from visual fields examination – **moderate bilateral defect**		OR	**Any MVC:** 3.6 (1.4, 9.4)
					ICD-9 codes 365.1 and 265.3, given an AGIS score from visual fields examination – **severe bilateral defect**		OR	**Any MVC:** 4.4 (1.6, 12.4)
Owsley C et al., 1998	Case Control	294 (179/155)	71	USA	Physician diagnosed	Drivers without glaucoma	OR	**Injurious MVC**: 3.6 (1.2, 10.9)*
								**At-fault MVC**: 1.5 (0.5, 4.8)*
							Prevalence (%)	14.1% (11/78) of all injurious crash drivers had glaucoma. 6.3% (6/101) of all non-injurious crash drivers had glaucoma.

Ono T et al., 2015	Cross-sectional	386 (199/187)	64.7	Japan	**Mild POAG in the worse eye** as a visual field defect corresponding to a mean deviation (MD) of −6 dB or better	Drivers without glaucoma	OR (logistic regression)	**Any MVC**: 1.07 (0.55, 2.1)*
					**Moderate POAG in the worse eye** as an MD between −6 and −12 dB		OR (logistic regression)	**Any MVC**: 1.44 (0.68, 3.08)*
					**Severe POAG in the worse eye** as an MD of −12 dB or worse		OR (logistic regression)	**Any MVC**: 2.28 (1.07, 4.88)*
					**Mild POAG in the better eye** as a visual field defect corresponding to a mean deviation (MD) of −6 dB or better		OR (logistic regression)	**Any MVC**: 1.36 (0.78, 2.37)*
					**Moderate POAG in the better eye** as an MD between −6 and −12 dB		OR (logistic regression)	**Any MVC**: 1.82 (0.65, 5.11)*
					**Severe POAG in the better eye** as an MD of −12 dB or worse		OR (logistic regression)	**Any MVC**: 1.65 (0.39, 6.87)*

For peer review only

					Physician diagnosis of POAG in any eye		Prevalence (%)	22.6% (45/199) pf drivers with glaucoma have been in an MVC compared to 16% (30/187) of drivers without glaucoma.
Tanabe S et al., 2011	Cross-sectional	265 (121/144)	61.6	Japan	Mild POAG as a visual field defect corresponding to a mean deviation (MD) of -5 dB or better in both eyes, moderate POAG as corresponding to an MD of -5 to -10 dB in the worse eye, severe POAG as an MD of -10 dB or worse in the worse eye	Drivers free of ocular disease	OR	**Any MVC (severe glaucoma)**: 9.9 (2.1, 47.8)
							Prevalence (%)	6% (7/121) of drivers with glaucoma have been involved in an MVC compared to 3.5% (5/144) of drivers without glaucoma.<br><br>When dividing by glaucoma severity, 3.9% (2/51) or moderate and 25% (5/20) of severe glaucoma drivers have been involved in a crash.

For peer review only

Wood J et al., 2016	Case Control	145 (75/70)	72.9	Australia	Visual acuity better than 20/40 with one or both eyes and binocular visual fields with a horizontal extent of at least 110° within 10° above and below the horizontal midline	Age-matched controls without glaucoma	Prevalence (%)	4% (3/75) of glaucoma drivers had an MVC in the past 12 months compared to 6% (4/70) of drivers without glaucoma; difference was not significant (p= 0.64)
								19% (14/75) of drivers with glaucoma had an MVC in the past 5 years compared to 23% (16/70) of drivers without glaucoma; difference was not significant (p=0.56)
Yuki K et al., 2014	Cross-sectional	247 (147/0)	63.7	Japan	Severity categorised using Mills Glaucoma Staging system – **better eye**	Drivers without history of MVC	Prevalence (%)	Amongst drivers with a history of MVCs, 11.8% (6/51), 72.5% (37/51), 9.8% (5/51), and 5.9% (3/51) had a better eye glaucoma

For peer review only

								severity score of 0, 1, 2, 3 or more, respectively. Amongst drivers without a history of MVC, this glaucoma score were: 20.4% (40/196), 65.8% (129/196), 9.2% (18/196), and 4.6% (9/196). The differences between proportion of people assigned these scores in the two MVC groups was not significant (p= 0.86).
					Severity categorised using Mills Glaucoma Staging system – **worse eye**			Amongst drivers with a history of MVCs, 2% (1/51), 47.1% (24/51), 23.5% (5/51) and 5.9% (3/51) had a worse eye glaucoma severity score of 0, 1, 2, 3 or more,

For peer review only

								respectively. Amongst drivers without a history of MVCs, the glaucoma scores were: 2.6% (5/196), 54.6% (107/196), 24.5% (48/196), and 18.3% (36/196), respectively. The differences between proportion of people assigned these scores in the two MVC groups was not significant (p= 0.86).
Yuki K et al., 2016	Prospective Cohort	191 (191/0)	63.7	Japan	Primary open angle glaucoma (POAG)	Drivers with POAG but no history of MVCs.	Prevalence (%)	15% (28/191) of drivers with glaucoma have been involved in an MVC. Of these, 64.3% (18/28) had mild, 14.3% (4/28) has moderate, and 22.4% (6/27) had severe glaucoma.
**Included in Narrative Summaries Only – Low Middle Income Countries**								

For peer review only

Deshmukh AV et al., 2019	Case Control	150 (100/50)	64.5	India	Diagnosed glaucomatous optic nerve head changes and corresponding visual field defects, which satisfied Anderson criterion	Aged-matched (older than 40 years) non-glaucoma controls	Prevalence (%)	12.9% (11/85) of drivers with glaucoma had an MVC in the past 12 months compared to 70% (35/50) of drivers without glaucoma. This significance was significant (p<0.001).

*unadjusted results

For peer review only

Table 4a(ii) All studies (n=8) on cataract and Motor Vehicle Crashes (MVC) with meta-analyses suitable for 2 studies on associations with any MVC involvement

Author and Year	Study Design	Total Participants (exposed/control)	Mean Age/Age Range	Country	VI Definition	Comparator	Outcome Measure (OR, RR, HR, etc.?)	Effect Measure (with 95% CI) + any description of results (if appropriate)
**Included in Meta-analysis (any MVC involvement)**								
Cross JM et al., 2009	Cross-sectional	3158 (1165/1993)	71.9	USA	Self-reported physician diagnosed	Drivers without cataract	RR (rate ratio)	**Any MVC**: 1.21 (0.95, 1.55)
Margolis KL et al., 2002	Prospective Cohort	1416 (370/1046)	71.3	USA	Self-reported physician diagnosed	Drivers without cataracts.	HR	**Any MVC:** 1.1 (0.88, 1.38)
**Included in Narrative Summaries Only – High Income Countries**								
Cross JM et al., 2009	Cross-sectional	3158 (1165/1993)	71.9	USA	Self-reported physician diagnosed	Drivers without cataract	RR (rate ratio)	**Injurious MVC**: 1.5 (0.85, 2.64)
								**At-fault MVC**: 1.01 (0.69, 1.49)
McCloskey L et al., 1994	Case Control	683 (118/672)	age, no.: 65- 69 years = 264, 70-74 years = 195, 75-79 years = 138, 80+ years = 86	USA	Physician diagnosed (hospital data)	Age-matched drivers with cataracts who have not been injured in a police reported MVC in the same calendar year as their matched case.	RR (relative risk)	**Injurious MVC**: 1 (0.7, 1.16)*
							Prevalence (%)	17.9% (42/234) of all injurious MVCs involved drivers with cataract.

McGwin G Jr et al., 2000	Case Control	901 (447/454)	N/A	USA	Self-reported physician diagnosed	Not-at-fault drivers without cataract were involved in crashes	OR	**Not-at-fault MVC**: 1.1 (0.7, 1.8)
							Prevalence (%)	35.1% (69/198) of all not-at-fault crashes involved drives with cataract**.** 44.6% of all at-fault crashes involved drivers with cataract.
Naredo Turrado J et al., 2020	Prospective Cohort	11670 (525/11145)	62.4	France	Self-reported physician diagnosed	Drivers without cataract	OR	**Any MVC**: 1.27 (0.91, 1.76)*
Owsley C et al., 1998	Case Control	294 (179/155)	71	USA	Physician diagnosed	Drivers without cataract	OR	**Injurious MVC**: 1 (0.6, 1.8)*
								**Non-injurious MVC**: 1.1 (0.6, 1.8)*
							Prevalence (%)	47.4% (37/78) of injurious MVCs involved drivers with cataracts.
Owsley C et al., 1999	Case Control	384 (279/105)	69.9	USA	Cataract in one or both eyes from clinic notes with VA in one eye of 20/40 or worse and no previous	Drivers without cataract	RR (relative risk)	**At-fault MVC**: 2.46 (1, 6.16)
							X^2 (Chi Square)	The difference between the number of accidents between drivers with cataract and those

For peer review only

					cataract surgery in either eye			without cataract was non-significant (p= 0.19)
Owsley C et al., 2001	Cross-sectional	377 (274/103)	69.9	USA	Best-corrected VA of 20/40 or worse in worse eye eyes	Crash-free drivers	OR (logistic regression)	**Any MVC:** 1.26 (0.28, 5.59)
					Best-corrected VA of 20/40 or worse in better eyes			**Any MVC:** 1.39 (0.42, 4.62)

*unadjusted results

For peer review only

Table 4a(iii) All studies (n=3) on Age-Related Macular Degeneration (AMD) and Motor Vehicle Crashes (MVC) all suitable to only be summarised narratively

Author and Year	Study Design	Total Participants (exposed/ control)	Mean Age/ Age Range	Country	VI Definition	Comparator	Outcome Measure (OR, RR, HR etc.?)	Effect Measure (with 95% CI) + any description of results (if appropriate)
**Included in Narrative Summaries Only – High Income Countries**								
Cross JM et al., 2009	Cross-sectional	3158 (88/2070)	71.9	USA	Self-reported physician diagnosed	Participants without AMD	RR (rate ratio)	**Any MVC**: 0.57 (0.23, 1.39)
								**Injurious MVC**: 0.9 (0.11, 7.44)
								**At-fault MVC**: 0.95 (0.35, 2.56)
McCloskey L et al., 1994	Case Control	683 (25/658)	age, no.: 65-69 years = 264, 70-74 years = 195, 75-79 years = 138, 80+ years = 86	USA	Physician diagnosed (hospital data)	Age-matched drivers with AMD who had not been injured in a police-reported MVC in the same calendar year as their matched case.	RR (relative risk)	**Injurious MVC**: 0.9 (0.4, 2)*
							Prevalence (%)	3.8% (9/234) of drivers with AMD had a history of an MVC.
McGwin G Jr et al., 2013	Retrospective Cohort	205 (142/63)	72.7	USA	AREDS definition for early AMD	Participants without AMD	RR (rate ratio)	**Any MVC**: 0.48 (0.2, 1.18)* **Any MVC per 100 person-**

								**years**: 0.67 (0.32, 1.39)* **Any MVC per 1, 000, 000 person-miles**: 0.73 (0.36, 1.5)*
					AREDS definition for intermediate AMD			**Any MVC**: 0.22 (0.08, 0.64)* **Any MVC per 100 person-years**: 0.34 (0.13, 0.89)* **Any MVC per 1, 000, 000 person-miles**: 0.35 (0.13, 0.91)*
					AREDS definition for severe AMD			**Any MVC**: 0.46 (0.14, 1.54)* **Any MVC per 100 person-years**: 0.93 (0.31, 2.77)* **Any MVC per 1, 000, 000 person-miles**: 1.11 (0.38, 3.19)*
Szlyk et al., 1995	Case Control	21 (10/11)	73.2	USA	Physician diagnosed	Age-similar subjects with normal vision	X^2 (Chi Square)	X2= 4.68 (p<0.03); Age similar controls had more self-

For peer review only

								reported accidents than those with ARMD. The difference between the groups for the numbers of individuals involved in self-reported accidents was significant
						Younger control subjects with normal vision	X^2 (Chi Square)	X2= 8.06 (p=0.01); The number of self-reported accidents was significantly different between the younger control group and the ARMD group with the younger control group having more self-reported accidents.

For peer review only

Wood JM et al., 2018	Case Control	83 (33/50)	75.4	Australia	AREDS definition for late AMD	Aged-matched controls with no AMD	Prevalence with X^2 (Chi Square)	9% (3/33) of drivers with AMD had a crash in the past 12 months compared to 2% (1/50) of control drivers (p=0.28). 30% (10/33) of drivers with AMD had a history of 1 or more crashes in the past 5 years compared to 16% (8/50) of controls drivers (p=0.23).

*unadjusted results

For peer review only

Table 4a(iv) All studies (n=3) on diabetic retinopathy (DR) and Motor Vehicle Crashes (MVC) all suitable to only be summarised narratively

Author and Year	Study Design	Total Participants (Exposed/Control)	Mean Age	Country	VI Definition	Comparator	Outcome Measure (OR, RR, HR, etc.?)	Effect Measure (with 95% CI) + any description of results (if appropriate
**Included in Narrative Summaries Only – High Income Countries**								
Cross JM et al., 2009	Cross-sectional	3158 (98/3060)	71.9	USA	Self-reported physician diagnosed	Drivers without DR	RR (rate ratio)	**Any MVC**: 0.6 (0.26, 1.38)<br>**Injurious MVC**: 0.95 (0.18, 4.92)<br>**At-fault MVC**: 0.32 (0.08, 1.17)
McGwin G Jr et al., 2000	Case Control	901 (447/454)	N/A	USA	Self-reported physician diagnosed	Drivers without DR involved in not-at-fault crashes	OR	**Not at-fault MVC**: 1.9 (0.3, 10.9)<br>**At-fault MVC**: 1.1 (0.3, 3.8)
							Prevalence (%)	1.1% (2/198) of not at-fault crash drivers had DR. 1.6% (3/249) of all at-fault crash drivers had DR.
Owsley C et al., 1998	Case Control	294 (179/155)	71	USA	Physician diagnosed	Drivers without DR	OR	**Non-injurious MVCs**:1 (0.1, 7.5)*<br>**Injurious MVCs:** 0.7 (0.1, 8.2)*

*unadjusted results

For peer review only

Table 4a(v) All studies (n=5) on stereopsis impairment and Motor Vehicle Crashes (MVC) with meta-analysis suitable for 3 studies on associations with any MVC involvement

Author and Year	Study Design	Participants/ Sample Size	Mean Age	Country	VI Definition	Comparator	Outcome Measure (OR, RR, HR?)	Effect Measure (with 95% CI) + any description of results (if appropriate)
**Included in Meta-analysis (any MVC involvement)**								
Boadi-Kusi SB et al., 2016	Cross-sectional	520 (80/440)	39.2	Ghana	Physician diagnosed as abnormal.	Drivers with normal stereopsis	OR	**Any MVC:** 0.89 (0.44, 1.8)*
Margolis KL et al., 2002	Prospective Cohort	1416 (N/A)	71.3	USA	Physician diagnosed - distance depth perception per standard deviation change	Drivers with normal stereopsis.	HR	**Any MVC:** 1.01 (0.92, 1.11)
Oladehinde MK et al., 2007	Cross-sectional	215 (11/204)	41.5	Nigeria	Physician diagnosed - Visual acuity of 6/6 - 6/18 was normal, < 6/18 - 6/60 was classified as visual impairment and < 6/60 - 3/60 was classified as severe visual impairment while visual acuity less than 3/60 was classified as blindness.	Drivers with normal stereopsis	RR (risk ratio)	**Any MVC:** 1.45 (0.42, 5.3)*

For peer review only

**Included in Narrative Summaries Only – High Income Countries**								
Alvarez-Peregrina C et al., 2022	Cross-sectional	736 (55/681)	46.4	Spain	Physician diagnosed	Drivers with normal stereopsis.	X^2 (Chi Square)	Stereopsis was not linked with history of MVCs (p> 0.05).
**Included in Narrative Summaries Only – Low Middle Income Countries**								
Boadi-Kusi SB et al., 2016	Cross-sectional	520 (80/440)	39.2	Ghana	Physician diagnosed as abnormal.	Drivers with normal stereopsis	Prevalence (%)	25% (20/30) of drivers with abnormal stereopsis were involved in an MVC.
Humphriss D, 1987	Cross-sectional	366 (N/A)	N/A	South Africa	Visual acuity of at least 6/12 in each eye separately, or if one eye is below 6/12 then the second eye must be 616 or, wearing glasses and seeing binocularly the acuity must be 6/12. A lateral field of vision of 45 degrees is required.	Drivers with better stereopsis	Mean (SD)	Mean vision test score for stereopsis drivers without MVC involvement: 4.128<br><br>Mean vision test score for stereopsis drivers with MVC involvement: 5
Oladehinde MK et al., 2007	Cross-sectional	215 (11/204)	41.5	Nigeria	Physician diagnosed - Visual acuity of 6/6 - 6/18 was normal, < 6/18 - 6/60 was classified as visual	Drivers with normal stereopsis	Prevalence (%)	18.2% (2/11) of all drivers with abnormal stereopsis have been involved in an MVC

					impairment and < 6/60 - 3/60 was classified as severe visual impairment while visual acuity less than 3/60 was classified as blindness.			

*unadjusted results

For peer review only

Table 4a(vi) All studies (n=4) on myopia and Motor Vehicle Crashes (MVC), with 2 studies for meta-analysis

Author and Year	Study Design	Total Participants (exposed/control)	Mean Age	Country	VI Definition	Comparator	Outcome Measure (OR, RR, HR?)	Effect Measure (with 95% CI) + any description of results (if appropriate)
**Included in Meta-analysis (any MVC involvement)**								
Ahmed M et al., 2021	Cross-sectional	700 (62/638)	42.3	Bangladesh	Physician diagnosed	Drivers without myopia but with a history of MVCs	OR	**Any MVC:** 0.5 (0.15, 1.65)*
Boadi-Kusi SB et al., 2016	Cross-sectional	520 (10/510)	39.2	Ghana	Physician diagnosed – spherical power in the better eye of -0.50D or worse	Drivers without myopia but with a history of MVCs	OR	**Any MVC**: 0.99 (0.41, 2.4)*
**Included in Narrative Summaries Only – High Income Countries**								
Cohen Y et al., 2007	Cross-sectional	136 (34/102)	21	Israel	Night myopia: refraction in illumination and in total darkness in both eyes changed by 0.75 D or more	Drivers without night myopia	Fischer's Exact Test	No statistically significant difference in day time crashes between night myopia and normal subjects (p= 0.22).<br>Night myopia drivers had higher night-time crashes than non-night myopia drivers (p=0.044).

McCloskey L et al., 1994	Case Control	683 (235/448)	age, no.: 65- 69 years = 264, 70-74 years = 195, 75-79 years = 138, 80+ years = 86	USA	Physician diagnosed (hospital data)	Age-matched drivers with myopia who have not been injured in a police-reported MVC in the same calendar year as their matched case.	RR (relative risk)	**Injurious MVC**: 0.6 (0.1, 1)*

*unadjusted results

For peer review only

Table 4a(vii) All studies (n=8) on colour vision deficiency (CVD) and Motor Vehicle Crashes (MVC), all suitable to only be summarised narratively due to methodological limitations in non-standardised diagnosis of colour vision deficiencies

Additional Narrative Summary:
One study looking at the different types of colour deficiency found individuals with protan colour deficiency, measured by Hardy-Rand Rittler (HRR) pseudo-isochromatic plates, to report significantly more MVCs than those with deutan colour deficiency (p= 0.034).

Author and Year	Study Design	Total Participants (exposed/control)	Mean Age/Age Range	Country	VI Definition	Comparator	Outcome Measure (OR, RR, HR, etc.?)	Effect Measure (with 95% CI) + any description of results (if appropriate)
**Included in Narrative Summaries Only – High Income Countries**								
Piyasena P et al., 2021	Systematic review	15394 (254/15140)	39.3	N/A	Physician diagnosed	Drivers without colour deficiencies.	RR	**Any MVC:** 1.36 (1.01, 1.82)*
**Included in Narrative Summaries Only – Low Middle Income Countries**								
Abebe Y et al., 2002	Cross-sectional	1878 (85/1794)	33.5	Ethiopia	Physician diagnosed - Ishihara plates	Drivers without colour deficiencies.	OR	**Any MVC:** 1.94 (1.18, 3.17)
							Prevalence (%)	32% (27/85) of all drivers with colour blindness were involved in an MVC.
Biza M et al., 2013	Cross-sectional	249 (4/245)	33.6	Ethiopia	Physician diagnosed - Ishihara plates	Drivers without colour deficiencies.	OR	**Any MVC:** 2.34 (0.19, 28.58)
							Prevalence (%)	25% (1/4) of all drivers with colour blindness were involved in an MVC.
Boadi-Kusi SB et al., 2016	Cross-sectional	520 (37/483)	39.2	Ghana	Protan colour deficient – Hardy-Rand	Deutan colour deficient	Prevalence (%)	52.9% (9/17) of proton colour blindness drivers

For peer review only - http://bmjopen.bmj.com/site/about/guidelines.xhtml

					Rittler (HRR) pseudo-isochromatic plate			were involved in an MVC compared to 30.8% (4/13) of deutan colour blindness drivers; X2= 6.194 (p=0.034)
								35% (13/37) of all colour blind drivers were involved in an MVC.
							X^2 (Chi Square)	Protan colour blind drivers were more likely to report MVCs than deutan colour blind drivers: 6.194 (p= 0.034)
Isawumi MA et al., 2011	Cross-sectional	99 (6/93)	45.9	Nigeria	Physician diagnosed - Ishihara plates	Drivers with an MVC history without colour deficiencies.	X^2 (Chi Square)	X^2= 0.09, p=0.76 No significance between the number of MVC involvement in those with colour blindness and those without.
							Prevalence (%)	33% (2/6) of all drivers with colour blindness

								were involved in an MVC.
Oladehinde MK et al., 2007	Cross-sectional	215 (7/208)	41.5	Nigeria	Physician diagnosed - Ishihara plates	Drivers without a history of MVCs	RR (risk ratio)	**Any MVC:** 1.12 (10.3, 11.5)
							Prevalence (%)	2% (1/57) of all recorded MVCs involved colour blind drivers.
							X^2 (Chi Square)	There were no statistically significant associations between colour vision impairment and RTA: 2.3 (p= 0.1)
Ovenseri-Ogomo G et al., 2011	Cross-sectional	206 (7/199)	39.2	Ghana	VA < 6/18 in the better eye)	Drivers without a history of MVCs	X^2 (Chi Square)	No significance found for MVC involvement in drivers with colour blindness: X^2= 2.142, p=0.344
Pepple G et al., 2014	Cross-sectional	400 (262/138)	37.8	Nigeria	Physician diagnosed - Ishihara plates	Drivers without colour blindness.	RR (did not state test used)	**Any MVC**: 1.23 (p=0.4)*
							Prevalence (%)	56% (10/18) of drivers with colour blindness were involved in an MVC.

*unadjusted results

For peer review only

Table 4a(viii) All studies (n=28) on visual acuity (VA) impairment and Motor Vehicle Crashes (MVC) with meta-analysis suitable for 5 studies on any crash involvement and 2 on at-fault crashes

Additional Narrative Summary:
Results for injurious crashes were mixed; all non-significant. Similarly, there were no significant risks found for non-injurious and at-fault crash involvement, irrespective of worsening VA. One study looking at visual acuity in normal and low luminance also found poor acuity to not be a significant predictor of crash risk in both lighting conditions. A Japanese study, however, found the odds of crashing to increase by 20% in drivers with primary open angle glaucoma (POAG) experiencing worse eye declines of 0.01 LogMAR increments compared to those without VA changes.

Author and Year	Study Design	Total Participants (exposed/control)	Mean Age	Country	VI Definition	Comparator	Outcome Measure (OR, RR, HR?)	Effect Measure (with 95% CI) + any description of results (if appropriate)
**Included in Meta-analysis (any MVC involvement)**								
Green K et al., 2013	Retrospective Cohort	2000 (N/A)	Age, no.: 70-79 years = 1432, 80-89 years = 526, 90-99 years = 40	USA	VA worse than 20/40	Drivers with VA 20/40 or better	RR (rate ratio)	**Any MVC**: 1.04 (0.74, 1.48)
Huisingh C et a., 2017	Prospective Cohort	659 (35/624)	N/A	USA	Distance VA > 0.3logMAR	Drivers with VA 20/40 or better	RR (rate ratio)	**Any MVC:** 0.98 (0.52, 1.84)
Margolis KL et al., 2002	Prospective Cohort	1416 (N/A)	N/A	USA	20/40 or worse	Drivers with VA 20/40 or better	HR	**Any MVC:** 1.14 (0.73, 1.8)
Piyasena P et al., 2021	Systematic Review	15394 (710/14684)	39.3	N/A	Physician diagnosed	Drivers without a vision impairment	RR	**Any MVC:** 1.46 (1.2, 1.78)

For peer review only

Rubin G et al., 2007	Prospective Cohort	2520 (N/A)	age, no.: 65-69 years = 780, 70-74 years = 829, 77-79 years = 553, 80-85 years = 350	USA	Physician diagnosed – 15-letter loss of visual acuity (0.3 logMAR i.e. VA 20/40)	Drivers with a VA better than 20/40.	HR	**Any MVC:** 1.06 (0.77, 1.68)
**Included in Meta-analysis (at-fault MVC involvement)**								
Green K et al., 2013	Retrospective Cohort	2000 (N/A)	Age, no.: 70-79 years = 1432, 80-89 years = 526, 90-99 years = 40	USA	VA worse than 20/40 = impairment	Drivers with VA better than 20/40	RR (rate ratio)	**At-fault MVC**: 1.08 (0.71, 1.4)
Huisingh C et a., 2017	Prospective Cohort	659 (35/624)	N/A	USA	Distance VA > 0.3logMAR	Drivers with VA 20/40 or better	RR (rate ratio)	**At-fault MVC:** 1.09 (0.58, 2.05
**Included in Narrative Summary Only – High Income Countries**								
Alvarez-Peregrina C et al., 2021	Cross-sectional	736 (548/188)	46.4	Spain	Physician diagnosed - cut-off not defined in study	Drivers without a VA impairment	X^2 (Chi Square)	Poor VA was linked with increased risk of MVCs (p< 0.001).
Cross JM et al., 2009	Cross-sectional	3158 (1323/1835)	71.9	USA	VA worse 20/20 and better 20/40	Those with binocular acuity of 20/20 or better in any MVC	RR (rate ratio)	**Any MVC:** 1 (0.78, 1.29)

For peer review only

					VA worse 20/20 and better 20/40	Those with binocular acuity of 20/20 or better in injurious MVC		**Injurious MVC:** 0.54 (0.28, 1.01)
					VA worse 20/20 and better 20/40	Those with binocular acuity of 20/20 or better in at-fault MVC		**At-fault MVC:** 1.08 (0.72, 1.62)
					VA 20/40 or worse	Those with binocular acuity of 20/20 or better in any MVC		**Any MVC:** 1.24 (0.74, 2.09)
					VA 20/40 or worse	Those with binocular acuity of 20/20 or better in injurious MVC		**Injurious MVC:** 0.55 (0.11, 2.8)
					VA 20/40 or worse	Those with binocular acuity of 20/20 or better in at-fault MVC		**At-fault MVC:** 1.37 (0.66, 2.82)
Gresset J et al., 1994	Case Control	4036 (151/3885)	N/A	Canada	Physician diagnosed poor VA	Those with better VA	OR	**Any MVC:** 0.99 (0.71, 1.4)
							Prevalence (%)	8.4% (118/1400) of those involved in an MVC had poor VA.

For peer review only

Gresset J et al., 1994	Case Control	4021 (N/A)	N/A	Canada	VA equal to 6/12 or 6/15 and normal binocularity	Drivers with VA 20/40 or better	OR	**Any MVC:** 0.97 (0.68, 1.38)*
					VA equal to 6/12 or 6/15 and lack of binocular vision			**Any MVC:** 1.23 (0.88, 1.72)*
Huisingh C et a., 2017	Prospective Cohort	659 (35/624)	N/A	USA	Distance VA > 0.3logMAR	Drivers with VA 20/40 or better	RR (rate ratio)	**Major MVC:** 0.81 (0.29, 2.26)
								**Any MVC:** 1.29 (0.87, 1.93)
		659 (74/585)			Near VA > 0.3 logMAR			**Major MVC:** 1.54 (0.9, 2.63)
								**At-fault MVC:** 1.19 (0.77, 1.85)
Ivers R et al., 1999	Cross-sectional	3654 (N/A)	N/A	Australia	Best eye VA <20/40-20/60	drivers with Best eye VA >/=20/40	Prevalence ratio (PR)	**Any MVC:** 1.3 (0.6, 2.8)
					Best eye VA <20/60	drivers with Best eye VA >/=20/40		**Any MVC:** 1.2 (0.3, 5)
					Right eye VA <20/40-20/60	drivers with Right eye VA >/=20/40		**Any MVC:** 0.7 (0.3, 1.6)
					right eye VA<20/60	drivers with right eye VA >/=20/40		**Any MVC:** 2 (1.2, 3.5)
					left eye VA <20/40-20/60	drivers with left eye VA >/=20/40		**Any MVC:** 1.1 (0.5, 2)

For peer review only

					left eye VA<20/60	drivers with left eye VA >/=20/40		**Any MVC:** 1.1 (0.5, 24)
Keeffe JE et al., 2002	Retrospective Cohort	2594 (N/A)	62.5	Australia	Visual acuity <6/12	Drivers with better vision (>6/12)	X^2 (Chi Square)	People with impaired vision (<6/12) were no more likely to have an accident or to attribute that the accident was the result of impaired vision; X2= 0.175 (p>0.9)
							Prevalence (%)	9.5% (32/339) of participant involved in an MVC had poor VA.
Kwon M et al., 2016	Cross-sectional	1899 (145/1754)	age, no.: 70-79 years = 1358, 80-89 years = 502, 90-98 years = 39	USA	Low VA classified as <20/40 (0.3 logMAR)	Drivers with glaucoma and binocular VA ≥ 20/20	RR (rate ratio)	**Any MVC:** 1.51 (0.55, 4.16)
McCloskey L et al., 1994	Case Control	683	age, no.: 65- 69 years = 264, 70-74	USA	Uncorrected VA of 20/25 or 20/30	Drivers with VA 20/15 or 20/20	RR (relative risk)	**Injurious MVC**: 2.5 (0.8, 7.2)*

For peer review only

			years = 195, 75-79 years = 138, 80+ years = 86		Uncorrected VA of 20/40			**Injurious MVC:** 1.7 (0.6, 5.3)*
					Uncorrected VA 20/50 or 20/60			**Injurious MVC:** 2.4 (0.8, 7.2)*
					Uncorrected VA 20/70 of greater			**Injurious MVC:** 2.1 (0.7, 5.8)*
					Corrected VA 20/25 or 20/30			**Injurious MVC:** 0.7 (0.5, 1.1)*
					Corrected VA 20/40			**Injurious MVC:** 0.6 (0.3, 1.2)*
					Uncorrected VA 20/50 or 20/60			**Injurious MVC:** 0.3 (0.1, 0.9)*
					Uncorrected VA 20/70 of greater			**Injurious MVC:** 4.3 (0.5, 40.3)*
McGwin G Jr et al., 2000	Case Control	901 (104/797)	N/A	USA	Near vision impairment	Not-at-fault drivers involved in crashes without poor near vision	OR	**Not-at-fault MVC:** 1.6 (0.8, 3.3)
							Prevalence (%)	8% (16/198) of not-at-fault MVCs involved drivers with near vision impairment.<br><br>13.2% (33/249) of at-fault MVCs involved drivers

								with near vision impairment.
		901 (339/562)			Far vision impairment	Not-at-fault drivers involved in crashes without poor far vision	OR	**Not-at-fault MVC:** 1.1 (0.7, 1.7)
							Prevalence (%)	36% (71/198) of not-at-fault crashes involved drivers with far vision impairment.<br><br>41% (102/249) of at-fault crashes involved drivers with far vision impairment.
		901 (57/844)			Peripheral vision impairment	Not-at-fault drivers involved in crashes without poor peripheral vision	OR	**Not-at-fault MVC:** 1.6 (0.7, 3.9)
							Prevalence (%)	4.7% (9/198) of not-at-fault crashes involved drivers with peripheral vision impairment.<br><br>8.5% of at-fault crashes

For peer review only

								involved drivers with peripheral vision impairment.
Ono T et al., 2015	Cross-sectional	386 (N/A)	64.7	Japan	BCVA in the **better eye** LogMAR per 0.1 increment	POAG drivers with BCVA in both eyes of 0.7 or more	OR	**Any MVC:** 0.94 (0.87, 1.01)
Owsley C et al., 2001	Cross-sectional	377 (136/241)	69.9	USA	VA 20/25 - 20/30 in better eye	Drivers with VA 20/25 or better in the **better eye**	OR	**At-fault MVC:** 1.88 (0.72, 4.88)
		377 (118/259)			VA 20/35 - 20/50 in better eye			**At-fault MVC:** 2.54 (0.87, 7.47)
		377 (77/300)			worse than VA 20/50 in better eye			**At-fault MVC:** 1.75 (0.45, 6.85)
		377 (51/326)			VA 20/25 - 20/30 in worse eye	Drivers with VA 20/25 or better in the **worse eye**		**At-fault MVC:** 0.19 (0.03, 1.27)
		377 (67/310)			VA 20/35 - 20/50 in worse eye			**At-fault MVC:** 0.82 (0.19, 3.61)
		377 (110/267)			worse than VA 20/50 in worse eye			**At-fault MVC:** 0.74 (0.16, 3.52)
		377 (N/A)			VA impairment defined as worse than 20/50 in only 1 eye	Drivers with no VA impairment (better than VA 20/50)		**At-fault MVC:** 1.35 (0.58, 3.15)

For peer review only

		377 (N/A)			VA impairment defined as worse than 20/50 in both eyes	Drivers with no VA impairment (better than VA 20/50)		**At-fault MVC:** 1.01 (0.29, 3.45)
Owsley C et al., 1998	Case Control	294 (36/258)	71	USA	VA worse than 20/40	Drivers with VA 20/40 or better	OR	**Injurious MVC:** 1.6 (0.6, 3.8)* **Non-injurious MVC:** 1.6 (0.7, 3.6)*
Rubin G et al., 2007	Prospective Cohort	2520 (N/A)	age, no.: 65-69 years = 780, 70-74 years = 829, 77-79 years = 553, 80-85 years = 350	USA	Physician diagnosed – 15-letter loss of visual acuity (0.3 logMAR i.e. VA 20/40)	Drivers with a VA better than 20/40.	HR	**Any MVC (at low luminance):** 1.06 (0.75, 1.47)
Sims RV et al., 1998	Case Control	174 (N/A)	71.1	USA	Physician Diagnosed	Older drivers without crashes in 6 years preceding 1991	Univariate analysis using student t-tests	Mean (SD) VA of those with a history of MVCs was 0.09 (0.31), compared to 0.03 (0.19) in those without a history of MVCs (p= 0.001).
Yuki K et al., 2014	Cross-sectional	247 (N/A)	63.7	Japan	Physician diagnosed as better VA (LogMar)	Drivers with POAG but without a	Unpaired t-test with Benjamini's correction	Differences between the VA of those who had a history of

For peer review only

						history of MVCs		an MVC and those who did not was significant, p= 0.036
					Physician diagnosed as worse VA (logMar)			Differences between the VA of those who had a history of an MVC and those who did not was not significant, p= 0.6
Yuki K et al., 2016	Prospective Cohort	191 (N/A)	63.7	Japan	POAG with 0.01 logMAR increase in **worse eye**	Drivers with POAG but without a history of MVCs	OR	**Any MVC:** 1.2 (1.1, 1.4)*
					POAG with 0.001 increase logMAR in **the better eye**			**Any MVC:** 0.68 (0, 221)*
**Included in Narrative Summaries only – Low Income Countries**								
Adekoya BJ et al., 2009	Cross-sectional	399 (N/A)	44.7	Nigeria	VA 6/9 in the better eye	N/A – looked at all participants	X^2 (Chi Square)	Inadequate VA in the better eye is not associated with MVC involvement in the last 10 years; X2= 0.035 (p= 0.851)

					VA 6/24 in the better eye		X^2 (Chi Square)	Inadequate VA in the second eye is not associated with involvement in RTA in the last 10 years; X2= 0.372 (p= 0.542)
Bekibele CO et al., 2007	Cross-sectional	99 (16/83)	50.1	Nigeria	Presenting vision less than 6/9 and improved with the aid of a minimum of 0.5 Diopter lenses, with VA <6/18	Drivers without refractive error	OR	**Any MVC:** 1.2 (0.4, 3.7)*
Boadi-Kusi SB et al., 2016	Cross-sectional	520 (38/482)	39.2	Ghana	Visual acuity of less than 0.2, either monocularly or binocularly, was classified as poor vision	N/A	X^2 (Chi Square)	No statistically significantly associations between poor vision due to refractive error and MVC involvement: X2= 3.090 (p= 0.388)
Humphriss D, 1987	Cross-sectional	366 (N/A)	N/A	South Africa	Binocular	Better mean vision test scores for binocular VA	Mean (SD)	Drivers involved in accidents were more likely to have worse mean vision test

For peer review only

								scores (10.031) for binocular VA compared to accident-free drivers (10.847), p<0.001
					right eye monocular VA	Better mean vision test scores for right eye monocular VA	Mean (SD)	Drivers involved in accidents more likely to have worse mean vision test scores (9.219) for right eye monocular VA compared to accident-free drivers (10.100), p<0.001
					left eye monocular VA	Better mean vision test scores for left eye monocular VA	Mean (SD)	Drivers involved in accidents more likely to have worse mean vision test scores (9.031) for left eye monocular VA compared to accident-free drivers (10.024), p<0.001

For peer review only

					Worse eye monocular acuity	Better mean vision test scores for worse eye monocular VA	Mean (SD)	Drivers involved in accidents more likely to have worse mean vision test scores for depth perception (4.128) compared to accident-free drivers (5.000), p<0.001
Isawumi MA et al., 2011	Cross-sectional	99 (5/94)	45.9	Nigeria	Poor driving vision if VA <6/12 in either eye	Drivers with an MVC but with normal vision.	X^2 (Chi Square)	MVCs were not directly related with VA and vice versa; X2= 1.6 (p= 0.65)
Oladehinde MK et al., 2007	Cross-sectional	215	41.5	Nigeria	Visual acuity < 6/18 - 6/60 was classified as visual impairment and < 6/60 - 3/60 was classified as severe visual impairment. VA < 3/60 was classified as blindness.	Drivers with VA 20/20 - 20/40	RR (did not state test used)	**Any MVC:** 3.5 (2.38, 5.14)*
Ogbonnaya CE et al., 2018	Cross-sectional	103 (7/96)	43.2	Nigeria	Minimum VA of 6/9 in the	Drivers without vision	X^2 (Chi Square)	The relationship between visual

For peer review only

					better eye and 6/12 in the worse eye of commercial motor vehicle drivers. Visually unfit to drive if VA <6/12 in the poorer eye.	impairment and no MVC history.		acuity fitness for driving and self-reported history of MVC was not statistically significant; X2= 0.05 (p= 0.82).
Ovenseri-Ogomo G et al., 2011	Cross-sectional	206 (14/192)	39.2	Ghana	VA < 6/18 in the better eye	Drivers without a history of MVC	X^2 (Chi Square)	VA not associated with history of MVC involvement; X^2= 5.982 (p=0.05)
Vofo BN et al. 2021	Cross-sectional	207 (51/156)	41.8	Cameroon	VA < 0.5	Drivers with VA > 0.5	Mean (SD)	Drivers with VA < 0.5 had a higher than average number of MVCs (2.91 +/- 1.72) compared to drives with VA > 0.5 (1.01 +/- 1.33).

*unadjusted results

For peer review only

Table 4a(ix) All studies (n=13) on contrast sensitivity (CS) impairment and Motor Vehicle Crashes (MVC), with only two studies suitable for meta-analysis due to different CS cut-off points, type of crash outcome explored and comparators used for each study.

Author and Year	Study Design	Total Participants (exposure/control)	Mean Age	Country	VI Definition	Comparator	Outcome Measure (OR, RR, HR, etc. ?)	Effect Measure (with 95% CI) + any description of results (if appropriate)
**Included in Meta-analysis (any MVC involvement)**								
Huisingh C et al., 2017	Prospective Cohort	659 (291/368)	N/A	USA	CS in better eye (< 1.5)	Drivers with CS ≥ 1.5 in better eye	RR (rate ratio)	**Any MVC**: 1.22 (0.82, 1.81)
Swain TA et al., 2021	Cross-sectional	159 (17/142)	79.3	USA	CS of <1.5 log sensitivity in the worse eye	Drivers with CS of >1.5 log sensitivity in the worse eye	RR	**Any MVC:** 1.5 (0.8, 3.2)
**Included in Narrative Summaries Only – High Income Countries**								
Cross JM et al., 2009	Cross-sectional	3158 (1323/1835)	71.9	USA	CS is >=1.575 and <1.675	Drivers without binocular CS impairments	RR (rate ratio)	**Any MVC:** 0.91 (0.68, 1.23)
								**Injurious MVC:** 0.94 (0.56, 1.58)
								**At-fault MVC**: 0.72 (0.49, 1.05)
					CS is >=1.450 and <1.575	Drivers without binocular CS	RR (cox proportional hazards)	**Any MVC:** 0.72 (0.49, 1.05)
								**Injurious MVC:** 0.71 (0.32, 1.56)
								**At-fault MVC**: 0.87 (0.49, 1.56)
					CS is <1.450	Drivers without binocular CS	RR (cox proportional hazards)	**Any MVC:** 1.01 (0.66, 1.55)
								**Injurious MVC:** 0.49 (0.16, 2.37)

For peer review only

								**At-fault MVC:** 1.27 (0.68, 2.37)
Green K et al., 2013	Retrospective Cohort	2000 (N/A)	Age, no.: 70-79 years = 1432, 80-89 years = 526, 90-99 years = 40	USA	Impairment defined as <1.5 on Pelli-Robson chart.	Drivers without binocular CS impairments	RR (rate ratio)	**Any MVC**; 1.42 (1, 2.02)
								**At-fault MVC**: 1.52 (0.93, 2.68)
Huisingh C et al., 2017	Prospective Cohort	659 (291/368)	N/A	USA	CS in worse eye (< 1.5)	Drivers with CS ≥ 1.5 in worse eye		**Any MVC:** 1.38 (1.05, 1.81)
					CS in better eye (< 1.5)	Drivers with CS ≥ 1.5 in better eye		**Major crash involvement**: 1.29 (0.77, 2.18)
					CS in worse eye (< 1.5)	Drivers with CS ≥ 1.5 in worse eye		**Major crash involvement:** 1.54 (1.07, 2.23)
					CS in better eye (< 1.5)	Drivers with CS ≥ 1.5 in better eye		**At-fault MVC:** 1.28 (0.84, 1.94)
					CS in worse eye (< 1.5)	Drivers with CS ≥ 1.5 in worse eye		**At-fault MVC:** **1.44** (1.08, 1.93)
Ivers R et al., 1999	Cross-sectional	3654 (N/A)	N/A	Australia	Vectorvision CSV-1000 chart: 3 cycle per degree in best eye CS	Reference group ≤ 2 units compared with >2 on a scale of 1-8	PR (Prevalence Ratio)	**Any MVC:** 1.3 (0.7, 2.2)

For peer review only

					Vectorvision CSV-1000 chart: 6 cycle per degree in best eye CS eye CS			**Any MVC:** 1.2 (0.7, 2.1)
					Vectorvision CSV-1000 chart: 12 cycle per degree in best eye CS			**Any MVC:** 1.4 (0.8, 2.3)
					Vectorvision CSV-1000 chart: 18 cycle per degree in best eye CS			**Any MVC:** 1.4 (0.9, 2.3)
					Vectorvision CSV-1000 chart: 3 cycle per degree in right eye CS			**Any MVC:** 1.2 (0.8, 1.9)
					Vectorvision CSV-1000 chart: 6 cycle per degree in right eye CS eye CS			**Any MVC:** 1 (0.6, 1.5)
					Vectorvision CSV-1000 chart: 12 cycle per degree in right eye CS			**Any MVC:** 2 (1.2, 3.1)

For peer review only

					Vectorvision CSV-1000 chart: 18 cycle per degree in right eye CS			**Any MVC:** 1.3 (0.8, 2.2)
					Vectorvision CSV-1000 chart: 3 cycle per degree in left eye CS			**Any MVC:** 1 (0.6, 1.6)
					Vectorvision CSV-1000 chart: 6 cycle per degree in left eye CS eye CS			**Any MVC:** 1.1 (0.6, 1.7)
					Vectorvision CSV-1000 chart: 12 cycle per degree in left eye CS			**Any MVC:** 1.3 (0.8, 2.2)
					Vectorvision CSV-1000 chart: 18 cycle per degree in left eye CS			**Any MVC:** 1.3 (0.8, 2.1)
Kwon M et al., 2016	Cross-sectional	1899 (432/1467)	age, no.: 70-79 years = 1358, 80-89 years =	USA	Pelli-Robson chart measure of ≤ 1.6 log sensitivity was defined as an impairment.	Older drivers with glaucoma, without CS impairment	RR (rate ratio)	**Any MVC**: 0.72 (0.36, 1.42)

For peer review only

			502, 90-98 years = 39					
Margolis KL et al., 2002	Prospective Cohort	1416 (N/A)	71.3	USA	low spatial frequencies per standard deviation change	N/A – looked at MVC information from all participants from 1986-1995	HR	**Any MVC:** 0.99 (0.89, 1.1)
					high spatial frequencies per standard deviation change			**Any MVC:** 0.94 (0.85, 1.04)
Owsley C et al., 1998	Case Control	294 (56/238)	71	USA	Pelli-Robson chart measure of <= 1.5 log sensitivity was defined as an impairment.	Older drivers with log(CS) >1.5	OR	**Injurious MVC:** 0.9 (0.4, 1.8)*
								**Non-injurious MVC:** 0.7 (0.3, 1.3)*
Owsley C et al., 2001	Cross-sectional	377 (274/103)	69.9	USA	CS impairment defined as ≤1.25	Participants with no CS impairment (CS >=1.50)	OR	**At-fault MVC (better eye CS >1.35 – 2.50):** 1.18 (0.41, 3.36)
								**At-fault MVC (better eye CS >1.25-1.35):** 1.21 (0.4, 3.68)
								**At-fault MVC (better eye CS ≤1.25):** 3.78 (1.15, 12.48)

For peer review only

								**At-fault MVC (worse eye CS >1.35 – 2.50):** 3.28 (0.71, 14.17)
								**At-fault MVC (worse eye CS >1.25-1.35):** 4.36 (0.84, 22.7)
								**At-fault MVC (worse eye CS ≤1.25):** 7.86 (1.55, 39.79)
								**At-fault MVC (unilateral CS ≤1.25):** 2.7 (1.16, 6.51)
								**At-fault MVC (bilateral CS ≤1.25):** 5.78 (1.87, 18.86)
Owsley C et al., 2020	Cross-sectional	915 (179/155)	age, no.: 60-69 years = 310, 70-79 years = 396, 80-90 years =	USA	Low photopic area under the log CS function	Drivers with higher photopic peak log sensitivity	RR (rate ratio)	**All MVC:** 0.8 (0.61, 1.04)
								**At-fault MVC:** 0.77 (0.57, 1.03)
					Low photopic peak log sensitivity			**All MVC:** 0.8 (0.61, 1.04)
								**At-fault MVC:** 0.77 (0.58, 1.03)

For peer review only

			200, 90-99 years = 9		Low mesopic area under the log CS function	Drivers with higher mesopic peak log sensitivity		**All MVC:** 1.36 (1.06, 1.72)
								**At-fault MVC:** 1.28 (1.01, 1.63)
					Low mesopic peak log sensitivity			**All MVC**: 1.5 (1.16, 1.93)
								**At-fault MVC:** 1.38 (1.07, 1.78)
Rubin G et al., 2007	Prospective Cohort	2520 (N/A)	age, no.: 65-69 years = 780, 70-74 years = 829, 77-79 years = 553, 80-85 years = 350	USA	Pelli-Robson Chart: 6 letter worsening (worsening of 0.3 logCS units)	N/A – looked at driver with and without MVC in whole population.	HR	**Any MVC when CS < 1.7**: 0.75 (0.49, 1.21)
								**Any MVC when CS ≥ 1.7**: 1.25 (0.44, 5.65)
Swain TA et al., 2021	Prospective Cohort	154 (17/137)	79.3	USA	CS of <1.5 log sensitivity in the worse eye	Drivers with CS of >1.5 log sensitivity in the worse eye	RR	**At-fault or near crash involvement:** 2.7 (1.3, 5.5)

For peer review only

Table 4a(x) All studies (n=20) on visual field (VF) impairment and Motor Vehicle Crashes (MVC) with meta-analysis suitable for only four studies on associations with any MVC involvement

Additional Narrative Summary:
When comparing impairments in the upper, lower, left, right, vertical and horizontal visual fields, impairments on the left side were found to be the most significant predictor of crash involvement (data from US, driving on right side of road).

Author and Year	Study Design	Total Participants (exposure/control)	Mean Age	Country	VI Definition	Comparator	Outcome Measure (OR, RR, HR, etc.?)	Effect Measure (with 95% CI) + any description of results (if appropriate)
**Included in meta-analysis (any MVC involvement)**								
Huisingh C et al., 2015	Cross-sectional	2000 (N/A)	N/A	USA	Bilateral VF impairment	Drivers without visual field impairments	RR (rate ratio)	**Any MVC:** 1.4 (1.07, 1.83)
Oladehinde MK et al., 2007	Cross-sectional	215 (22/193)	41.5	Nigeria	Bilateral VF impairment	Drivers without a MVC history	RR (risk ratio)	**Any MVC:** 1.07 (0.98, 6.73)
Piyasena P et al., 2021	Systematic Review	15394 (337/15057)	39.3	N/A	Physician Diagnosed	Drivers without VF impairment	RR	**Any MVC:** 1.36 (1.25, 1.48)
Swain TA et al., 2021	Cross-sectional	159 (40/119)	79.3	USA	Overall VF loss of ≤ 22.4dB in the worse eye	Drivers with no overall VF loss in the worse eye	RR	**Any MVC:** 1.6 (0.8, 3.1)
**Included in Narrative Summaries Only – High Income Countries**								
Ball K et al., 1993	Cross-sectional	294 (N/A)	71	USA	Sensitivity loss in the 30 to 60 degree region of the visual field	N/A	Spearman's Correlation (r)	VF loss was significantly related to crash frequency however the LISREL model shows that it only has indirect effects

								on crash frequency but direct effects on UFOV which is the most significantly associated variable with crash frequency; 0.26
Huisingh C et al., 2015	Cross-sectional	2000 (N/A)	N/A	USA	Upper field impairments	Drivers without visual field impairments	RR (rate ratio)	**Any MVC:** 1.1 (0.83, 1.44)
					Lower Field Impairments			**Any MVC:** 1.4 (1.07, 1.82)
					Horizontal Meridian Impairments			**Any MVC:** 1.31 (1, 1.72)
					Vertical Meridian Impairments			**Any MVC:** 1.26 (0.97, 1.65)
					Left Side impairments			**Any MVC:** 1.49 (1.15, 1.92)
					Right side impairments			**Any MVC:** 1.16 (0.88, 1.53)
Huisingh C et al., 2017	Prospective Cohort	659 (406/253)	N/A	USA	Peripheral visual field loss at 70 or 85 degrees temporally in **either eye**	Drivers with no visual field loss in either eye	RR (rate ratio)	**Any MVC:** 1.08 (0.8, 1.47)
								**Major MVC:** 1.53 (1.02, 2.29)
								**At-fault MVC:** 0.98 (0.71, 1.37)

For peer review only

		659 (186/473)			Peripheral visual field loss at 70 or 85 degrees temporally in **both eye**	Drivers with no visual field loss in both eyes		**Any MVC:** 1.74 (1.18, 2.56)
								**Major MVC:** 2.32 (1.4, 3.83)
								**At-fault MVC:** 0.73 (1.14, 2.61)
Kwon M et al., 2016	Cross-sectional	1899 (N/A)	age, no.: 70-79 years = 1358, 80-89 years = 502, 90-98 years = 39	USA	Overall visual field loss ≤ 22.5 dB	Drivers (with glaucoma) without severe visual field loss.	RR (rate ratio)	**Any MVC:** 2.11 (1.09, 4.09)
					Upper visual field loss ≤ 22.5 dB			**Any MVC:** 2.37 (1.19, 4.74)
					Lower visual field loss ≤ 22.5 dB			**Any MVC:** 2.32 (1.13, 4.75)
					Left visual field loss ≤ 22.5 dB			**Any MVC:** 3.16 (1.55, 6.46)
					Right visual field loss ≤ 22.5 dB			**Any MVC:** 1.63 (0.84, 3.14)
					Horizontal meridian loss ≤ 22.5 dB			**Any MVC:** 1.78 (0.92, 3.44)
					Vertical meridian loss ≤ 22.5 dB			**Any MVC:** 1.09 (0.56, 2.11)
Kristalovich L et al., 2019	Retrospective cohort	445 (286/159)	N/A	Canada	Loss of at least 120 continuous degrees along the horizontal meridian and 15 continuous	Drivers with either no VFI or with VFI but meeting licensing standards	X^2 (Chi Square)	No significant difference in rate of crash between VFI/not meet licensing

For peer review only

					degrees above and below fixation with both eyes open and examined together.			standards and no VFI and VFI/meet licensing standards (p=0.402)
McGwin G Jr et al., 2015	Retrospective Cohort	438 (N/A)	72.8	USA	Binocular visual field **total deviations <7.25**	Drivers (with glaucoma) without severe visual field impairments	RR (rate ratio)	**At-fault MVC:** 1.5 (0.82, 2.74)
					Binocular visual impairment severely **impaired threshold** <20.4			**At-fault MVC:** 1.49 (0.81, 2.74)
					Binocular visual impairment severely impaired **pattern deviation <3.97**			**At-fault MVC:** 2.13 (1.21, 3.75)
Owsley C et al., 1998	Case Control	294 (36/258)	71	USA	Central 30 degree VF sensitivity: >10	Older drivers with central 30 degree VF sensitivity of 0-10	OR	**Injurious MVC:** 2.6 (1.1, 6.3)*
								**Non-injurious MVC:** 1.8 (0.8, 4.4)*
		294 (108/186)			Peripheral 20-60 degree VF sensitivity: >10	Older drivers with peripheral 30-60 degree VF sensitivity of 0-10		**Injurious MVC:** 2.4 (1.3, 4.5)*
								**Non-injurious MVC:** 1.8 (1, 3.1)*
Rubin G et al., 2007	Prospective Cohort	2520 (N/A)	age, no.: 65-69	USA	Binocular visual field <20 (loss of 15 points)	N/A – looked at drivers with and without MVC in	HR	**Any MVC:** 0.59 (0.34, 1)

For peer review only

For peer review only - http://bmjopen.bmj.com/site/about/guidelines.xhtml

			years = 780, 70-74 years = 829, 77-79 years = 553, 80-85 years = 350		Binocular visual field >=20 (loss of 15 points)	whole population.		**Any MVC:** 1.31 (1.31, 4.27)
Swain TA et al., 2021	Cross-sectional	159 (41/118)			Peripheral VF loss of ≤ 19.2dB in the worse eye	Drivers with no peripheral VF loss in the worse eye		**Any MVC:** 2.4 (1.3, 4.4)
		159 (41/118)			Superior VL loss of ≤ 22.0dB in the worse eye	Drivers with no superior VF loss in the worse eye		**Any MVC:** 0.7 (0.4, 1.5)
		159 (41/118)			Inferior VL loss of ≤ 22.1dB in the worse eye	Drivers with no inferior VF loss in the worse eye		**Any MVC:** 1.7 (0.4, 1.5)
		159 (40/119)			Left VL loss of ≤ 21.6dB in the worse eye	Drivers with no left VF loss in the worse eye		**Any MVC:** 1.7 (0.9, 3.2)
		159 (41/118)			Right VF loss of ≤ 21.8dB in the worse eye	Drivers with no right VF loss in the worse eye		**Any MVC:** 1.6 (0.9, 3)
Swain TA et al., 2021	Prospective Cohort	154 (38/116)	79.3	USA	Overall VF loss of ≤ 22.4dB in the worse eye	Drivers with no overall VF loss in the worse eye	RR	**At-fault or near crash:** 1.4 (0.8, 2.8)

For peer review only

		154 (40/114)			Peripheral VF loss of ≤ 19.2dB in the worse eye	Drivers with no peripheral VF loss in the worse eye		**At-fault or near crash:** 1.8 (1, 3.3)
		154 (43/111)			Superior VL loss of ≤ 22.0dB in the worse eye	Drivers with no superior VF loss in the worse eye		**At-fault or near crash:** (1.3 (0.7, 2.5)
		154 (41/113)			Inferior VL loss of ≤ 22.1dB in the worse eye	Drivers with no inferior VF loss in the worse eye		**At-fault or near crash:** (1.4, 0.8, 2.5)
		154 (42/112)			Left VL loss of ≤ 21.6dB in the worse eye	Drivers with no left VF loss in the worse eye		**At-fault or near crash:** 1.3 (0.7, 2.5)
		154 (36/118)			Right VF loss of ≤ 21.8dB in the worse eye	Drivers with no right VF loss in the worse eye		**At-fault or near crash:** 0.9 (0.5, 1.8)
Yuki K et al., 2014	Cross-sectional	247 (N/A)	63.7	Japan	N/A	POAG drivers without a MVC history	Mean (SD)	The mean IVF-MD (db) of glaucoma drivers with a history of MVCS was -0.6 (3.4) compared to -0.8 (3.7) in glaucoma drivers without a history of MVCs.
Yuki K et al., 2016	Prospective Cohort	191 (N/A)	63.7	Japan	POAG with **1dB increase in visual field**	POAG drivers without a MVC history	OR	**Any MVC:** 0.95 (0.8, 1.1)*
							Mean (SD)	Mean (SD) IVF-MD (dB) of glaucoma

For peer review only

								drivers with a history of MVC was -2.1 (3.9) compared to -1.6 (3.7) in glaucoma drivers without a history of MVCs.
**Included in Narrative Summaries Only – Low Middle Income Countries**								
Abraham EG et al., 2010	Cross-sectional	291 (13/278)	41.5	Nigeria	Cup-disc ratio >0.5 cup-disc disparity between the two eyes of up to 0.2 or more, abnormal disc pallor (localised or generalised)	Drivers without visual field impairments.	RR (relative risk)	**Any MVC:** 0.628*
Adekoya BJ et al., 2009	Cross-sectional	399 (21/378)	44.7	Nigeria	Presence of 1 or more abnormal quadrants on confrontation perimetry	N/A	X^2 (Chi Square)	Abnormal visual fields was not associated with MVC involvement in the last 10 years; X2= 1.715 (p= 0.19).
Humphriss D, 1987	Cross-sectional	366 (N/A)	N/A	South Africa	N/A	Drivers with no MVC history	Mean (SD)	Data not reported
Isawumi et al., 2011	Cross-sectional	99 (N/A)	45.9	Nigeria	N/A	Drivers with a MVC but	Prevalence (%)	21.1% (8/38) of drivers with an MVC also had

For peer review only

						without visual field loss		horizontal visual field loss.
Ovenseri-Ogomo G et al., 2011	Cross-sectional	206 (14/192)	39.2	Ghana	VA < 6/18 in the better eye	Drivers without a history of MVC	OR	Any MVC: 0.54 (0.016, 18.45)*
Pepple G et al., 2014	Cross-sectional	400 (16/384)	37.8	Nigeria	Physician diagnosed	Drivers without visual field impairments	RR (did not state test used)	**Any MVC:** 1.25*
							Prevalence (%)	56% (9/16) of those with visual field impairment were have been involved in an MVC.

*unadjusted results

For peer review only

Table 4a(xi) All studies (n=3) on glare sensitivity (GS) impairment and Motor Vehicle Crashes (MVC), all suitable to only be summarised narratively due to their different GS cut-off points, type of crash outcome explored and comparators

Author and Year	Study Design	Total Participants (exposed/control)	Mean Age/ Age Range	Country	VI Definition	Comparator	Outcome Measure (OR, RR, HR, etc.?)	Effect Measure (with 95% CI) + any description of results (if appropriate)
**Included in Narrative Summaries Only – High Income Countries**								
Owsley C et al., 1998	Case Control	294 (71/179)	71	USA	Measured using MCT-8000 (Vis Tech), defined as disability glare >0	Older drivers with disability glare <= 0	OR	**Injurious MVC**: 1.4 (0.8, 2.5)*
								**Non-injurious MVC:** 1.3 (0.9, 2.2)*
Owsley C et al., 2001	Cross-sectional	377 (274/103)	69.9	USA	Glare impairment defined as >=0.25, measured with Pelli-robson chart with BAT:	Those with disability glare <0.25 in the better/worse eye	OR	**At-fault MVC in the better eye:** 0.68 (0.22, 2.12)
					Glare impairment defined as >=0.25 in both eyes	those with no disability glare impairment (<0.25 score)	OR (logistic regression)	**At-fault MVC in the worse eye**: 0.62 (0.29, 1.33)
								**At-fault MVC in both eyes:** 0.46 (0.14, 1.53)
Rubin G et al., 2007	Prospective Cohort	2520 (N/A)	age, no.: 65-69 years = 780, 70-74 years = 829, 77-79 years =	USA	6 letter worsening (worsening of 0.3 logCS units) - measured using Pelli-Robson chart with BAT	N/A – looked at driver with and without MVC in whole population.	HR	**Any MVC (glare <3 letters)**: 0.46 (0.26, 0.89)
								**Any MVC (glare ≥ 3 letters)**: 2.3 (1.14, 16.78)

For peer review only

			553, 80-85 years = 350					

For peer review only

Table 4a(xii) All studies (n=19) on other types of vision impairment and Motor Vehicle Crashes (MVC), all suitable to only be summarised narratively

Author and Year	Study Design	Total Participants (exposure/control)	Mean Age/ Age Range	Country	Type of VI	VI definition	Comparator	Outcome Measure (OR, RR, HR, etc.?)	Effect Measure (with 95% CI) + any description of results (if appropriate)
**Included in Narrative Summaries only – High Income Countries**									
Baker JM et al., 2019	Retrospective Cohort	66253 (62/66191)	20.8	USA	Unilateral vision impairment	ICD-9 diagnostic codes (369.6-369.8)	Young adult drivers without unilateral vision impairment	HR	**Any MVC**: 1.08 (0.6, 1.95)
		66253 (352/65901)			Amblyopia	Using the ICD-9 diagnostic codes (368.00 - 368.03) in the HER with diganosis noted in medical record from age 6	Young adult drivers without amblyopia	HR	**Any MVC**: 1.08 (0.85, 1.38)
Crizzle AM et al., 2020	Cross-sectional	3346 (513/2833)	61.5	Canada	Vision impairment	Physician diagnosed	Drivers without vision impairment	Univariate log rank test	Vision impairment was not associated with history of MVCs (p=0.9178).

Fishman GA et al., 1981	Retrospective Cohort	129 (42/87)	37.3	USA	Retinitis Pigmentosa	Physician diagnosed	Drivers free from ophthalmic or general defects	X^2 (Chi Square)	Statistical significant difference in accidents recorded over 5 years between retinitis pigmentosa patients (50%; 21/42)) and controls (29%; 25/62); p= 0.02
Gresset J et al., 1994	Case Control	4036 (15/4021)	N/A	Canada	Monocularity	Physician diagnosed	Male drivers who had no accident during their 70th year in 1988 and 1989	OR	**Any MVC**: 0.95 (0.32, 2.77)
		4036 (327/3709)			Visual impairment			OR	**Any MVC:** 1.07 (0.84, 1.36)
Maag U et al., 1997	Retrospective Cohort	116 (N/A)	N/A	Canada	Vision impairment	Non stereoscopic vision (> 160 seconds); an acuity of 20/40 for the better eye and zero in the other.	Drivers in good health	Mean (SD)	Average total number of crashes in people with good health with a taxi per year (SD): 0.218 (0.501)<br>Average total number of crashes in people with binocular vision problems with

For peer review only

									taxi per year (SD): 0.369 (0.595); the difference was statistically significant (p= 0.01)
McCloskey L et al., 1994	Case Control	683 (10/673)	age, no.: 65-69 years = 264, 70-74 years = 195, 75-79 years = 138, 80+ years = 86	USA	Retinopathy	Physician diagnosed (hospital data)	Age-matched drivers with retinopathy who have not been injured in a police reported MVC in the same calendar year as their matched case.	RR (relative risk)	**Injurious MVC**: 0.6 (0.1, 2.6)*
		683 (37/646)			Retinal disorders	Physician diagnosed (hospital data)	Age-matched drivers with other retinal disorders who have not been injured in a police	RR (relative risk)	**Injurious MVC**: 0.8 (0.4, 1.6)*

For peer review only

							reported MVC in the same calendar year as their matched case.		
		683 (394/289)			Hypermetropia	Physician diagnosed (hospital data)	Age-matched drivers with hypermetropia who have not been injured in a police reported MVC in the same calendar year as their matched case.	RR (relative risk)	**Injurious MVC**: 0.9 (0.7, 1.4)*
		683 (544/139)			Presbyopia	Physician diagnosed (hospital data)	Age-matched drivers with presbyopia who have not been injured in a police reported MVC in the same calendar year as their	RR (relative risk)	**Injurious MVC**: 1 (0.6, 1.8)*

For peer review only

							matched case.		
		683 (339/344)			Astigmatism	Physician diagnosed (hospital data)	Age-matched drivers with astigmatism who have not been injured in a police reported MVC in the same calendar year as their matched case	RR (relative risk)	**Injurious MVC**: 0.9 (0.7, 1.4)*
		638 (597/41)			Refractive disorder	Physician diagnosed (hospital data)	Age-matched drivers with refractive disorders who have not been injured in a police reported MVC in the same calendar year as their matched case	RR (Mantel-Haenszel)	**Injurious MVC**: 0.3 (0.1, 0.8)*
		638 (6/632)			Monocular vision	Physician diagnosed (hospital data)	Age-matched drivers with monocular vision who have not	RR (relative risk)	**Injurious MVC**: 0.7 (0.1, 4.1)*

For peer review only

							been injured in a police reported MVC in the same calendar year as their matched case		
		638 (10/628)			Diplopia	Physician diagnosed (hospital data)	Age-matched drivers with diplopia who have not been injured in a police reported MVC in the same calendar year as their matched case	RR (relative risk)	**Injurious MVC**: 1.2 (0.4, 4.2)*
		638 (13/625)			Vision/ophthalmic conditions	Physician diagnosed (hospital data)	Age-matched drivers with other vision and opthalmic conditions who have not been injured in a police reported MVC in the same	RR (relative risk)	**Injurious MVC**: 0.6 (0.2, 1.6)*

For peer review only

							calendar year as their matched case		
Naredo Turrado J et al., 2020	Prospective Cohort	11670 (11/11659)	62.4	France	Retinal detachment	Self-reported physician diagnosed	Drivers without retinal detachment	OR	**Any MVC**: 0.99 (0.37, 2.7)
Owsley C et al., 1998	Case Control	294 (N/A)	71	USA	Stereoacuity	Scores ≥ 500 arcseconds on TNO test	Older drivers with stereoacuity <500 arcseconds	OR (logistic regression)	**Injurious MVC**: 2.2 (1.1, 1.4)*<br>**Non-injurious MVC**: 1.2 (0.7, 2.3)*
Pepple G et al., 2014	Cross-sectional	400 (32/368)	37.8	Nigeri a	Vision impairment	Physician diagnosed	Drivers without a vision impairment	RR (did not state test used)	**Any MVC**: 0.62 (p= 0.46)
Rubin G et al., 2007	Prospective Cohort	2520 (545/2066)	age, no.: 65-69 years = 780, 70-74 years = 829, 77-79 years = 553, 80-85 years = 350	USA	Stereoacuity	Stereodeficient was defined at failing the test at 457 arc seconds.	Drivers who were not sterodeficient	HR (cox proportion al hazard)	**Any MVC**: 1.44 (0,88, 2.27)

For peer review only

Runge JW, 2000	Cross-sectional	N/A	N/A	USA	Vision Impairment	Physician diagnosed	Drivers without vision impairment s	RR (relative risk)	**At-fault MVC**: 1.51*<br><br>The at-fault crash rate of those with a vision impairment was 1.14 compared to those without an impairment (0.75).
Rahi J et al., 2006	Retrospective Cohort	8661 (429/8432)	N/A	UK	Amblyopia	Mild = acuity 6/6 in one eye and 6/9 or 6/12 in the other and unilateral visual loss	People with normal vision in each eye	OR	**Any MVC**: 1.28 (0.87, 1.89)
						Moderate/severe = acuity of 6/6 in one eye and 6/18 or worse in the other and unilateral visual loss, with or without strabismus, earlier in childhood.	People with normal vision in each eye	OR (ordinal regression)	**Any MVC**: 2.33 (1.29, 4.2)
Wedenoja J et al., 2021	Cross-sectional	N/A	N/A	Finland	Vision impairment	Physician diagnosed	Drivers without	Prevalence	Only 1.3% (13/968) of all fatal MVCs were

For peer review only

							vision impairment.		caused by vision-related problems.
**Included in Narrative Summaries Only – Low Middle Income Countries**									
Ahmed M et al., 2021	Cross-sectional	700 (492/208)	42.3	Bangla desh	Near or distance visual impairment	Presenting VA ≥ 6/7.5 in the better eye and or presence of presbyopia.	Drivers without near or distance visual impairment but with a history of MVCs.	OR	**Any MVC:** 2.45 (1.09, 5.49)
		700 (125/575)	42.3	Bangla desh	Hyperopia	Physician diagnosed	Drivers without hyperopia but with a history of MVCs.	OR	**Any MVC:** 1.1 (0.56, 2.23)*
		700 (11/689)	42.3	Bangla desh	Presbyopia	Physician diagnosed	Drivers without presbyopia but with a history of MVCs.	OR	**Any MVC:** 1.7 (0.96, 3.01)*
		700 (N/A)	42.3	Bangla desh	Any distance refractive error	Physician diagnosed	Drivers without any distance refractive error but with a history of MVCs.	OR	**Any MVC:** 1.66 (0.88, 3.12)*
Biza M et al., 2013	Cross-sectional	249 (13/236)	33.6	Ethiop ia	Visual impairment	VA <6/18-6/60 was classified as	Drivers with a MVC but no	OR	**Any MVC (both eyes**

						moderate visual impairment and <6/60-3/60 was classified as severe VI while VA less than 3/60 was classified as blindness.	VA impairment		**impairment)**:42.82 (2.53, 724.03)
									**Any MVC (right eye impairment)**: 0.03 (0.004, 0.28)*
									**Any MVC (left eye impairment)**: 0.09 (0.01, 0.97)*
Boadi-Kusi SB et al., 2016	Cross-sectional	520 (66/454)	39.2	Ghana	Hyperopia	Hyperopia defined as the spherical power in the better eye of +1.00D or more	Drivers with a history of MVC but no hyperopia	OR	**Any MVC**: 0 (0, 0);
		520 (30/490)			Astigmatism	Astigmatism was defined as −0.50D cylinder or worse in the better eye	Drivers with a history of MVC but no astigmatism	OR	**Any MVC**: 0.885 (0.32, 2.5)*
Emerole C et al., 2013	Cross-sectional	280 (102/178)	N/A	Nigeria	Vision impairment causing poor visibility.	Physician diagnosed with VA of 6/30 classified as abnormal.	N/A – compared with a "control" group but paper never explained what/who	Prevalence (%)	119 (79.3%) participants in the study group had an MVC history.<br><br>40.3% (448/119) participants in the study group

For peer review only

							the control group was.		and 70.6% (36/51) in the control group listed poor visibility as the cause of their MVC involvement ($p < 0.05$).
Ogbonnaya CE et al., 2018	Cross-sectional	103 (9/94)	43.2	Nigeria	Monocular vision impairment	Physician diagnosed	Drivers with monocular impairment but with no MVC history	X^2 (Chi Square)	The relationship between monocular visual impairment and self-reported history of RTA was not statistically significant; X2 =0.045, (p= 0.85)
		103 (7/96)			Monocular blindness	Physician diagnosed	Drivers with monocular blindness but with no MVC history	X^2 (Chi Square)	The relationship between monocular blindness and self-reported history of RTA was not statistically significant; X2 =0.358 (p= 0.55)
Vofo BN et al., 2021	Cross-sectional	207 (51/156)	41.8	Cameroon	Self-reported vision impairment.	Self-reported	Drivers without self-reported	X^2 (Chi Square)	Drivers with self-reported VI were involved in significantly

							vision impairment		higher number of MVCs (72.5%) than those with self-reported good vision (55.8%) ($p< 0.05$)
									Drivers with self-reported VI had higher average number of MVCs over previous 10 years (1.75 +/- 1.64) than drivers with self-reported good vision (1.03 +/- 1.40 ($p< 0.05$).

*unadjusted results

For peer review only

Table 4a(xiii) All studies (n=6) evaluating cataract surgery and Motor Vehicle Crashes (MVC) with meta-analysis suitable for 3 studies on the associations with any MVC involvement

Author and Year	Study Design	Total Participants (exposure/control)	Mean Age	Country	VI Definition	Comparator	Outcome Measure (OR, RR, HR?)	Effect Measure (with 95% CI)
**Included in Meta-Analysis (any MVC involvement)**								
Meuleners L et al., 2012	Retrospective Cohort	27827 (N/A)	age, no.: 60-69 years = 6609, 70-79 years = 14506, 80+ years = 6712	Australia	Physician diagnosed	Crashes before surgery	RR (risk ratio)	0.87 (0.76, 0.99)
Meuleners L et al., 2019	Retrospective Cohort	2849 (N/A)	age, no.: 60-64 years = 347, 65-69 years = 482, 70-74 years = 720, 75-79 years = 719, 80-84 years = 454, 85 +	Australia	Physician diagnosed cataract	Crashes before surgery	RR (risk ratio)	0.39 (0.37, 0.41)

For peer review only

			years = 127					
Owsley C et al., 2002	Prospective Cohort	277 (174/103)	71.3	USA	Cataract in 1 or both eyes with best-corrected VA of 20/40 or worse	Crashes before surgery	RR (rate ratio)	0.47 (0.23, 0.94)
**Included in Narrative Summaries Only – High Income Countries**								
McCloskey L et al., 1994	Case Control	683 (235/448)	age, no.: 65- 69 years = 264, 70-74 years = 195, 75-79 years = 138, 80+ years = 88	USA	Self-reported physician diagnosed cataracts	Drivers who experienced no injuries in a crash.	RR (relative risk)	**Post-surgery with lens implant:**1 (0.5, 2.3)*
Meuleners L et al., 2012	Retrospective Cohort	Males: 1091 (513/611)	age, no.: 60-69 years = 447, 70-79 years = 823, 80 + years = 445	Australia	Physician diagnosed	No. of pre cataract surgery police reported crashes in all participants.	RR (risk ratio)	**Males:** 0.84 (0.72, 0.99)
		Females: 624 (308/330)						**Females**: 0.99 (0.75, 1.16)
Meuleners L et al., 2019	Retrospective Cohort	2849 (N/A)	age, no.: 60-64 years = 347, 65-69 years	Australia	Physician diagnosed cataract	Crashes before surgery	RR (risk ratio)	**After 2nd eye cataract surgery:** 0.77 (0.75, 0.78)

For peer review only

			= 482, 70-74 years = 720, 75-79 years = 719, 80-84 years = 454, 85 + years = 127					
Schlenker M et al., 2018	Prospective cohort	559546 (N/A)	76	Canada	Physician diagnosed	No. of pre cataract surgery crashes in all participants	OR	0.91 (0.84, 0.97)*

*unadjusted results

For peer review only

Table 4a(xiv) All studies (n=1) evaluating corrective lens wear to improve refractive error and Motor Vehicle Crashes (MVC)

**Author and Year**	**Study Design**	**Total Participants (exposure/control)**	**Mean Age**	**Country**	**Vision impairment**	**VI Definition**	**Comparator**	**Outcome Measure (OR, RR, HR?)**	**Effect Measure (with 95% CI)**
McCloskey L et al., 1994	Case Control	683 (235/448)	age, no.: 65- 69 years = 264, 70-74 years = 195, 75-79 years = 138, 80+ years = 94	USA	Refractive Error	Use of corrective lenses for any reason (far or near vision)	Drivers who experienced no crash-related injuries	RR (risk ratio)	0.6 (0.3, 1.1)*
								Prevalence (%)	% with condition, cases: 91% (214/235) % with condition, controls: 94.6% (424/448)

* unadjusted results

For peer review only

Appendix 4b Raw data tables and additional narrative summaries of papers on driving cessation

Table 4b(i) All studies (n=13) on glaucoma and driving cessation with meta-analysis suitable for 2 studies

Additional Narrative Summaries:
Persons with bilateral glaucoma (OR 2.6 (95% Cl 1.4-4.8); p= 0.002) were more likely to stop driving but those with unilateral glaucoma were not (OR 1.5 (95% Cl 0.7-2.9); p= 0.3) with one Japanese study reporting individuals with severe POAG in the better eye to have an approximately 11.5 times greater odds of driving cessation than persons without POAG.

Author and Year	Study Design	Total Participants (exposure/control)	Mean Age	Country	VI Definition	Comparator	Outcome Measure (OR, RR, HR?)	Effect Measure (with 95% Cl)
**Included in Meta-analysis**								
Edwards J et al., 2008	Cross-sectional	1656 (152/1504)	72.95	USA	Self-reported physician diagnosed	Participants without glaucoma	HR	1.47 (0.98, 2.19); p=0.06
Gilhotra JS et al., 2001	Cross-sectional	3654 (61/3593)	65.9	Australia	Self-reported and physician diagnosed	Participants without glaucoma	OR	2.2 (1.3, 3.9)
**Included in Narrative Summaries Only – High Income Countries**								
Adler G et al., 2004	Cross-sectional	199 (52/147)	71.3	USA	Open-or closed-angle glaucoma	Participants without glaucoma	X^2 (Chi Square)	Drivers with glaucoma were no more likely than controls to have made plans for driving cessation; p=0.49
Edwards J et al., 2008	Cross-sectional	1656 (152/1504)	72.95	USA	Self-reported physician diagnosed	Participants without glaucoma	Prevalence (%)	8.6% (125/1450) of current drivers had glaucoma compared to 13.9% (28/199) of non-drivers with glaucoma.
Gilhotra JS et al., 2001	Cross-sectional	3654 (61/3593)	65.9	Australia	Open-angled	Participants without glaucoma	Prevalence (%)	2% (37/2379) of current drivers had glaucoma compared to 5% (24/451) of non-drivers with glaucoma.

Goh Y et al., 2011	Case Series	77 (77/0)	71.8	UK	Physician diagnosed	Participants with glaucoma and other ocular pathologies	OR	At clinic presentation: 4.99 (1.2, 20.6)* Glaucoma patients with other ocular pathologies were more likely to fail the driving criteria and give up driving than patients with only glaucoma.
								At last clinic visit: 4.37 (1.6, 11.8) Glaucoma patients with other ocular pathologies were more likely to fail the driving criteria and give up driving than patients with only glaucoma.
Kaleem MA et al., 2021	Cross-sectional	191 (191/0)	77	USA	Physician diagnosed	Drivers with glaucoma but with either better VA or CS.	Prevalence (%) and X^2 (Chi Square)	78% of participants reported that they had stopped driving.
								Participants with worse VA were more likely to stop driving ($p< 0.05$)
								Participants with worse CS were more likely to stop driving ($p< 0.01$).
MacLeod K et al., 2014	Cross-sectional	1279 (67/1211)	age, no.: 55-64 years = 233, 65-	USA	Self-reported physician diagnosed	Ex-drivers without glaucoma	RR (risk ratio)	1.3
							Attributable Risk	1.6

For peer review only

			74 years = 499, 75+ years = 547				Prevalence (%)	7.4% (6/79) of non-driving participants had glaucoma compared to 5.7% (5/79) who did not have glaucoma.
Marottoli RA et al., 1993	Cross-sectional	1331 (28/1303)	age, no.: 65-74 years = 484, 75-84 years = 105, 85+ years = 6	USA	Self-reported physician diagnosed	Participants without glaucoma	Prevalence (%)	From the 28 participants who reported glaucoma at baseline (1983), 42.9% (12/28) stopped driving by 198 compared to 22.2% (125/564) of people who did not have glaucoma and who also stopped driving.
Naredo Turrado J et al., 2020	Prospective cohort	11670 (525/11144)	62.4	France	Self-reported physician diagnosed	Participants without glaucoma	HR	1.6, p>0.05
Ramulu P et al., 2009	Cross-sectional	1135 (138/997)	79.7	USA	Bilateral or unilateral	Participants without glaucoma	OR	Bilateral: 2.6 (1.4, 4.8)<br><br>Stopped driving for over 8 years (bilateral): 3 (1.4, 6.4)*<br><br>Stopped driving less than 2 years ago (bilateral): 3.6 (1.5, 5.8)
								Unilateral: 1.5 (0.7, 2.9)<br><br>Stopped driving less than 2 years ago (unilateral): 2.4 (1, 6)
							Prevalence (%)	40.6% (28/68) of all participants with

For peer review only

								bilateral glaucoma were not driving. 21.4% (15/70) of all with unilateral glaucoma were not driving. 15% (150/997) of all without glaucoma were not driving.
Takahashi A et al., 2018	Prospective cohort	359 (211/148)	54	Japan	Mild POAG at baseline	Participants without glaucoma	OR	No association found (data not shown)
					Moderate POAG in the better eye at the 3 year follow-up			37.7 (3.7, 383.8)
					Severe POAG in the better eye at baseline			11.52 (2.87, 46.35)
					Severe POAG in the better eye at 3-year follow-up			52.8 (3.5, 797)
							Prevalence (%)	5.3% (8/152) of those with mild glaucoma were no longer driving.<br><br>21% (7/33) of those with moderate/severe glaucoma were no longer driving.<br><br>A total of 8.1% (15/185) of all participants with glaucoma were not driving compared to 1/3% (1/80) of drivers without glaucoma who

								were also no longer driving.
Tam A et al., 2018	Cross-sectional	99 (99/0)	71.5	Canada	Glaucoma severity was defined by the visual field mean deviation (MD) in the better eye and classified into 2 groups: mild (MD >−6 dB) and moderate/severe (MD ≤−6 dB), corrected visual acuity in the better eye ≥20/50	Mild/moderate glaucoma patients	Prevalence (%)	33% (15/46) of mild/moderate glaucoma reported driving cessation compared to 8% (4/53) of mild glaucoma patients; p= 0.002
vanLandingham et al., 2013	Cross-sectional	139 (81/58)	70.1	USA	Physician diagnosed	Glaucoma suspect controls	OR	4 (1.1, 4.7); p=0.03
							Prevalence (%)	22.5% (18/81) of participants with glaucoma were no longer driving.
**Included in Narrative Summaries Only – Low Middle Income Countries**								
Deshmukh AV et al., 2019	Case Control	150 (100/50)	64.5	India	Anderson criterion	Drivers without glaucoma	Prevalence (%)	16% (16/100) of those with glaucoma has stopped driving.

*unadjusted results

For peer review only

Table 4b(ii) All studies (n=4) on cataract and driving cessation, all suitable to be summarised narratively only

Additional Narrative Summaries:
One study with sex disaggregated analysis found male drivers to be 7.01 times more likely to stop driving compared to female drivers who only had a 3.67 odds of driving cessation. Only one study examined the impact of a diagnosis of wet AMD but did not find any significant associations.

Author and Year	Study Design	Total Participants (exposure/control)	Mean Age	Country	VI Definition	Comparator	Outcome Measure (OR, RR, HR?)	Effect Measure (with 95% CI)
**Included in Narrative Summaries Only – High Income Countries**								
MacLeod K et al., 2014	Cross-sectional	1279 (278/1001)	age, no.: 55-64 years = 233, 65-74 years = 499, 75+ years = 547	USA	Self-reported physician diagnosed	Ex-drivers without cataract.	RR (risk ratio)	1.5
							Attributable risk	10.5, p<0.1
							Prevalence (%)	8% (6/79) of participants with cataracts no longer drove compared to 5.2% (4/79) with no cataracts.
Marottoli RA et al., 1993	Cross-sectional	1331 (105/1226)	age, no.: 65-74 years = 484, 75-84 years = 105, 85+ years = 6	USA	Self-reported physician diagnosed	Current Drivers	OR	2.29 (1.28, 4.1)
							Prevalence (%)	45.7% (48/105) of participant with cataracts were no longer driving compared to 18.4% (90/488) of those who were no longer driving and did not have cataracts.
Naredo Turrado J et al., 2020	Prospective cohort	11670 (291/11379)	62.4	France	Self-reported physician diagnosed	Current drivers	HR	1.79, p>0.05
Sengupta S et al., 2014	Cross-sectional	122 (N/A)	72.4	USA	Physician diagnosed	Participants without cataract/PCSO in better eye.	PR (Prevalence Ratio)	Presence of cataract/PCO in the better seeing eye did not show any significant association

For peer review only

								with driving cessation; p>0.5

*unadjusted results

Table 4b(iii) All studies (n=5) on AMD and driving cessation with meta-analysis suitable for 3 studies

**Author and Year**	**Study Design**	**Total Participants (exposure/control)**	**Mean Age**	**Country**	**VI Definition**	**Comparator**	**Outcome Measure (OR, RR, HR?)**	**Effect Measure (with 95% CI)**
				**Included in Meta-analysis**				
Campbell MK et al., 1993	Case Control	1656 (276/1380)	N/A	USA	Self-reported physician diagnosed	Current drivers	OR	4.25 (2.6, 7); p<0.001
Edwards J et al., 2008	Cross-sectional	1656 (89/1567)	72.95	USA	Self-reported physician diagnosed	Current drivers	HR	1.46 (0.91, 2.36); p=0.12
Stewart RB et al., 1993	Cross-sectional	1470 (N/A)	78.1	USA	Self-reported physician diagnosed	Current drivers	OR	3.32 (1.91, 5.77); p=0.0001
				**Included in Narrative Summaries Only – High Income Countries**				
Campbell MK et al., 1993	Case Control	1656 (276/1380)	N/A	USA	Self-reported physician diagnosed	Current drivers	OR	**Male**: 7.01 (3.1, 15.9); p<0.001)*
								**Female**: 3.67 (2.0, 6.8), p<0.001*
							Prevalence (%)	5.06% (70/1379) of participants still driving had AMD compared to 17.88% (50/277) of non-drivers with AMD.
Edwards J et al., 2008	Cross-sectional	1656 (89/1567)	72.95	USA	Self-reported physician diagnosed	Current drivers	Prevalence (%)	4.9% (71/1457) of participant still driving had AMD compared to 9.5% (19/198) of non-driving participant with AMD.

For peer review only

MacLeod K et al., 2014	Cross-sectional	1279 (48/1231)	age, no.: 55-64 years = 233, 65-74 years = 499, 75+ years = 547	USA	Self-reported physician diagnosed	Ex-drivers without AMD.	RR (risk ratio)	2.3
							Attributable risk	4.5, p<0.01
							Prevalence (%)	12.7% (10/79) of ex-drivers had AMD compared to 5.6% (4/79) of ex-drivers without AMD.
Sengupta S et al., 2014	Cross-sectional	122 (64/58)	72.4	USA	Physician reported wet AMD	Participants without AMD	OR	**Any eye:** 1.9 (0.5, 7.3)
								**Worse eye:** 0.6 (0.1, 3.3)
								**Better eye:** 2.7 (0.6, 11.5)
							Prevalence (%)	74.6% (48/64) of participant with AMD were still driving compared.
								More participants in the AMD group (25.4%) had stopped driving compared to those without AMD (6.9%); p= 0.006
Stewart RB et al., 1993	Cross-sectional	1470 (N/A)	78.1	USA	Self-reported physician diagnosed	Current drivers	Prevalence (%)	59.8% (35/58) of participant with AMD were still driving.

*unadjusted results

Table 4b(iv) All studies (n=18) on visual acuity (VA) impairment and driving cessation, all suitable to only be summarised narratively due to their different VA cut-off points and comparators

**Author and Year**	**Study Design**	**Total Participants (exposure/control)**	**Mean Age**	**Country**	**VI Definition**	**Comparator**	**Outcome Measure (OR, RR, HR?)**	**Effect Measure (with 95% CI)**
**Included in Narrative Summaries Only – High Income Countries**								
Anstey K et al., 2006	Prospective Cohort	1466 (446/1020)	age no.: 70-74 years = 378, 75-79 years = 353, 80-84 years = 339, 85+ years = 396	Australia	Corrected distance VA at 3 metres in best eye at 6/12 or worse	Participants with VA better than 6/12 (i.e. better than 20/40)	OR	Visit 2: 1.91 (0.51, 7.13)<br>Visit 3: 1.84 (0.68, 4.99)<br>Visit 4: 1.15 (0.55, 2.41)
DeCarlo D et al., 2003	Cross-sectional	126 (N/A)	79	USA	Better eye	Current drivers	Mean (SD)	VA in the better eye was worse in non-drivers (1.03 +/- 0.39) than drivers (0.74 +/- 0.34).
					Worse eye			VA in the worse eye was worse in non-drivers (1.58 +/- 0.43) than drivers (1.18 +/- 0.42).

For peer review only - http://bmjopen.bmj.com/site/about/guidelines.xhtml

Edwards J et al., 2008	Cross-sectional	1656 (N/A)	72.95	USA	ETDRS chart with scores assigned from 0 to 90 (e.g. score of 0 = Snellen score of 20/125, score of 90 = Snellen score of 20/16)	Current drivers	HR (multivariate model)	0.91 (0.791, 1.046); p=0.184
							HR (cox regression)	0.69 (0.61, 0.78); p<0.001
Freeman E et al., 2005	Prospective cohort	1824 (263/1561)	73.4	USA	≥ 0.1 and <0.3 logMAR at baseline	<0.1 logMAR as baseline	HR	1.27 (0.96, 1.69)
		1824 (63/1498)			≥0.3 logMAR VA at baseline	<0.1 logMAR as baseline		1.23 (0.69, 2.18)
		1824 (329/1495)			1-2 lines VA loss	<1 line loss in VA		1.25 (0.96, 1.65)
		1824 (134/1690)			>2 lines VA loss	<1 line loss in VA		1.26 (0.87, 1.84)
Garre-Olmo J et al., 2009	Cross-sectional	875 (N/A)	81.7	Spain	Self-reported	Drivers without impaired VA	OR	0.379 (0.201, 0.714); p=0.003*
Gilhotra JS et al., 2001	Cross-sectional	3654 (80/3574)	65.9	Australia	BCVA worse than 6/12 in the better eye	Current drivers	OR	4 (2.5, 3.9)
		3654 (283/3371)			Presenting VA worse than 6/12 in the better eye			2.5 (1.9, 3.4)
							Prevalence (%)	11% (49/452) of participants have stopped driving have VA >20/40 compared to the 1% (21/2379) who are still driving with VA >20/40.
Huisingh C et al., 2016	Prospective Cohort	1995 (161/1834)	77.2	USA	logMar <0.3	Drivers without VA impairment	HR	0.83 (0.49, 1.42)
							Mean (SD)	VA of those who stopped driving (0.097 [0.15]) compared to those still driving (0.051 [0.13]).

For peer review only

							Prevalence (%)	90.9% (149/164) of those not driving had a VA of ≤20/40 compared to 9.2% (15/164) who stopped driving with a VA of >20/40.
Janz N et al., 2009	Prospective Cohort	607 (N/A)	age, no.: 25-49 years = 131, 50-64 years = 240, 65-74 years = 177	USA	Better eye at 6 months	Driving vs. non-drivers	2-sample t-test	Mean (SD) of VA in drivers (87.7 [4.9]) vs. non-drivers (85.1 [5.4]); p<0.001
							Linear regression	Mean (SD) of VA in drivers (87.7 [4.9]) vs. non-drivers (85.1 [5.4]); p=0.012
					Better eye at 54 months		2-sample t-test	Mean (SD) of VA in drivers (86.9 [5.7]) vs. Non-drivers (83.2 [6.9]); p= 0.025
							Linear regression	Mean (SD) of VA in drivers (86.9 [5.7]) vs. Non-drivers (83.2 [6.9]); p=0.458
					Mean (SD) difference in VA in better eye from 6 months to 54 months	Remained drivers vs. became non-drivers	Linear regression	Changes in Mean (SD) in VA of drivers (-0.4[0.6]) vs. became non-drivers (3.9[0.7]); p=0.001
					Worse eye at 6 months	Driving vs. non-drivers	2-sample t-test	Mean (SD) of VA in drivers (83.2 [7.5]) vs. non-drivers (79.7 [11.0]); p= 0.007
							Linear regression	Mean (SD) of VA in drivers (83.2 [7.5]) vs. non-drivers (79.7 [11.0]);

For peer review only

								p= 0.095
					Worse eye at 54 months		2-sample t-test	Mean (SD) of VA in drivers (81.5 [10.6]) vs. non-drivers (75.3 [14.4]); p=0.001
							Linear regression	Mean (SD) of VA in driver (81.5 [10.6]) vs. non-driver: 75.3 (14.4); p=0.003
					Mean (SD) difference in VA in worse eye from 6 months to 54 months	Remained drivers vs. became non-drivers	Linear regression	Mean (SD) of VA in drivers (1.4[1.3]) vs. became non-drivers: -5.5(2.1); p=0.054
Keay L et al., 2009	Prospective Study	1425 (N/A)	75	USA	LogMAR scale	Whole population	Mean (SD)	mean(SD) of VA statistically significant different between those who stopped driving 0.08 (0.014) and those who continued driving -0.01(0.11); p=0.0006
Keay et al., 2016	Cross-sectional	442 (N/A)	73	Australia	High contrast vision	Current drivers with cataracts NOTE: all participants had cataracts	OR	1.21 (1.07, 1.37)
					Binocular		X^2 (Chi Square)	p<0.001
					Better eye			p<0.001
					Worse Eye			p<0.001
Levecq L et al., 2013	Cross-sectional	1000 (N/A)	71.3	Belgium	Physician diagnosed binocular VA worse than 20/40	Current drivers	X^2 (Chi Square)	Right eye: Mean VA of current drivers (0.31) was significantly better than those who gave up driving due to vision (0.25); p=0.016
								Left eye: Mean VA in current drivers (0.31) was significantly

For peer review only

								better than those who gave up driving due to vision (0.24); p=0.004
								Both eyes:<br>Mean VA in current drivers (0.36)<br>Was significantly better than those who gave up driving due to vision (0.31); p=0.031
Ramulu P et al., 2009	Cross-sectional	1135 (N/A)	79.7	USA	Binocular acuity 0.1 logMAR or worse in better eye.	Drivers without 0.1 logMAR binocular.	OR	1.5, p<0.001
Ross L et al., 2009	Cross-sectional	5206 (1062/4144)	76.3	Australia	Physician diagnosed with participants categorised into having a VA LogMAR 0.3 or better, or worse than LogMar 0.3.	Participants with normal vision (logMAR of or better than 0.3).	OR	2.08 (2.56, 1.69)*
Rubin G et al., 2007	Prospective Cohort	2520 (N/A)	age, no.: 65-69 years = 780, 70-74 years = 829, 77-79 years = 553, 80-85 years = 350	USA	15 letter loss (logMAR 0.3)	Current Drivers	Prevalence (%)	Of those no longer driving: 84% (604/719) had VA ≤ 20/40 whilst 16% (115/719) had VA > 20/40.

For peer review only

Segal-Gidan F et al., 2010	Cross-sectional	421 (44/377)	72	USA	Mild vision impairment was defined at the BCVA in the better eye (20/40-20/63)	Current drivers	OR	5.53 (1.45, 20.98)
		421 (23/377)			Moderate/severe vision impairment was defined as BCVA in the better eye (20/80 or worse)			13.23 (1.45, 120.3)
Sengupta S et al., 2014	Cross-sectional	122 (N/A)	72.4	USA	Worse VA in the better eye (1 line loss of vision)	1 line worse in better eye acuity (logMAR) in all participants	OR	Low VA in either eye: 1.4 (1.1, 1.9); p<0.001 Low VA in better eye: 1.5 (1.2, 1.9); p<0.001
							Mean (SD)	Participants who had stopped driving (logMAR VA 0.77) had significantly worse vision in the better seeing eye than those still driving (LogMAR VA 0.08); p=0.001
Tam A et al., 2018	Cross-sectional	99 (N/A)	71.5	Canada	Physician diagnosed	N/A – looked at VA in whole population (all glaucoma patients)	X^2 (Chi Square)	Best corrected VA not associated with cessation; p=0.18
								Declines in central vision was significantly associated with driving cessation; p= 0.001
								Declines in near vision was significantly associated with driving cessation; p= 0.001
								Declines in peripheral vision was significantly associated

For peer review only

								with driving cessation; p= 0.001
vanLandingham S et al., 2013	Cross-sectional	139 (N/A)	70.1	USA	1 line worse in the better eye	Glaucoma suspect controls	OR	1.3 (1, 1.8); p<0.05
						Moderate VA loss in glaucoma cases		Severe VA loss: 1.5 (1.2, 1.8); p< 0.05

*unadjusted results

Table 4b(v) All studies (n=8) on contrast sensitivity (CS) impairment and driving cessation with 3 studies suitable for meta-analysis

Additional Narrative Summaries:
CS was measured either as a continuous measure, or categorised as "poor" according to normative cut-points, with one study using both measures.

Author and Year	Study Design	Total Participants (exposure/control)	Mean Age	Country	VI Definition	Comparator	Outcome Measure (OR, RR, HR?)	Effect Measure (with 95% Cl)
**Included in Meta-analysis**								
Huisingh C et al., 2016	Prospective cohort	1995 (130/1865)	77.2	USA	<1.5 score on Pelli-Robson chart	Drivers with no bilateral CS impairment	HR	1.73 (1.1, 2.72)
							Mean (SD)	The mean log CS of current drivers was 1.68 (0.13) compared to 1.61 (0.16) in non-drivers.
							Prevalence (%)	5.8% (106/1831) of current drivers had a log CS <1.5, compared to 14.6% (24/164) who stopped driving.
Keay L et al., 2009	Prospective cohort	1425 (N/A)	75	USA	Per letter lost	Drivers with no bilateral CS impairment	OR	1.15 (1.03, 1.28)*
					Better eye CS		Mean (SD)	CS in better eye of those who stopped driving 32.4(4.1) significantly different between those who continued driving 35.3(2.2); p<0.001
		122 (N/A)	72.4	USA			OR	1.36 (1.1, 1.7); p<0.05

Sengupta S et al., 2014	Cross-sectional				Binocular CS 1 letter worse	Drivers with no bilateral CS impairment	X^2 (Chi Square)	Those who stopped driving had significantly worse CS (log CS 1.8) compared to those still driving (log CS 1.2); p=0.03
**Included in Narrative Summaries Only – High Income Countries**								
Freeman E et al., 2005	Prospective cohort	1824 (725/1099)	73.4	USA	>=32 and <36 letters CS at baseline	Baseline CS equal to or more than 36 letters.	HR	1.26 (0.97, 1.63)
		1824 (158/1666)			<32 letters at baseline			1.46 (0.98, 2.17)
		1824 (79/1725)			5 letter CS loss in 2 years	Less than 5 letter CS loss		1.33 (0.8, 2.22)
		1824 (86/1738)			>= 6 letter CS loss in 2 years			1.71 (1.01, 2.9)
Keay L et al., 2016	Cross-sectional	442 (N/A)	73	Australia	0.12 log units drop in CS.	Cataract patients who are still driving	OR	1.29 (1.11, 1.49)
							Prevalence (%)	17% (45/263) of current drivers and 35% (37/110) of former drivers had a CS <1 log decrease by at follow-up; p< 0.001
							Mean (SD)	The worse eye CS in current drivers was 1.27 (+/- 0.36) compared to 1.11 (+/- 0.41) in former drivers; p< 0.001
Ramulu P et al., 2009	Cross-sectional	1135 (N/A)	79.7	USA	5 letters worse in better eye	Current drivers without 5 letters worse in better eye CS.	OR	3, p<0.001
Rubin G et al., 2007	Prospective Cohort	2520 (N/A)	Age, no,: 65-69 years =	USA	Log CS ≥ 1.65	Current drivers	Prevalence (%)	49.1% (884/1801) participants had stopped driving.

For peer review only

			780, 70-74 years = 829, 77-79 years = 553, 80-85 years = 350		Log CS 1.35-1.65			54% (973/1801) had stopped driving.
					Log CS <1.35			96.9% (1745/1801) had stopped driving.
vanLandingham S et al., 2013	Cross-sectional	139 (N/A)	70.1	USA	Binocular CS 1 letter worse	Glaucoma suspect controls	OR	1.3 (1.2, 1.4); p<0.05

*unadjusted results

Table 4b(vi) All studies (n=8) on visual field (VF) impairment and driving cessation, all suitable to only be summarised narratively due to their different VF cut-off points and comparators

Author and Year	Study Design	Total Participants (exposure/control)	Mean Age	Country	VI Definition	Comparator	Outcome Measure (OR, RR, HR?)	Effect Measure (with 95% CI)
**Included in Narrative Summaries Only – High Income Countries**								
Freeman E et al., 2005	Prospective Cohort	1824 (659/1165)	73.4	USA	>1 and <= 8 points of central visual field at baseline	Equal to or greater than 1 points missed at baseline central VF	HR	1.34 (1.02, 1.76)
		1824 (174/1650)			>9 points of central visual field at baseline	Equal to or greater than 1 points missed at baseline central VF		1.81 (1.23, 2.66)
		1824 (65/1759)			5-7 points of central visual field loss in 2 years	<5 central VF loss		1.01 (0.6, 1.72)

For peer review only

		1824 (92/1732)			>=8 points of central visual field loss in 2 years	<5 central VF loss		0.83 (0.53, 1.29)
		1824 (632/1192)			>9 and <=18 points of peripheral visual field at baseline	Less than or equal to 9 points missed at baseline peripheral VF		1.51 (1.14, 1.98)
		1824 (180/1644)			>18 points of peripheral visual field at baseline	Less than or equal to 9 points missed at baseline peripheral VF		1.73 (1.14, 1.98)
		1824 (106/1718)			6-7 points of peripheral visual field loss in 2 years	<6 points loss of peripheral VF		1.04 (0.65, 1.65)
		1824 (88/1736)			>= 8 points of peripheral visual field loss in 2 years	<6 points loss of peripheral VF		1.91 (1.23, 2.96)
Huisingh C et al., 2016	Prospective cohort	1995 (493/1502)	77.2	USA	sensitivity <=22.5 dB	Participants without VF impairment	HR	1.78 (1.29, 2.46)
Janz N et al., 2009	Prospective cohort	607 (N/A)	age, no.: 25-49 years = 131, 50-64 years = 240, 65-74 years = 187	USA	Better eye at 6 months	Drivers vs. non-drivers	2-sample t-test	Mean (SD) MD of drivers (-2.1 [2.7]), vs. non-drivers (-2.9 [3.0)]; p=0.014
							Liner regression	Mean (SD) MD of drivers (-2.1 [2.7]) vs. non-drivers (-2.9 [3.0]); p=0.966
					Better eye at 54 months		2-sample t-test	Mean (SD) MD of drivers (-1.9[3.1]) vs. non-drivers (-3.5 [3.7]); p<0.001

For peer review only

							Linear regression	Mean (SD) MD of drivers (-1.9[3.1]) vs. non-driver: -(3.5[3.7]);<br>p= 0.007
					Mean (SD) difference in VA in better eye from 6 months to 54 months	Remain drivers vs. became non-drivers	Linear regression	Difference in mean (SD) MD of drivers (0.2 [2.1]) vs. became non-drivers (-0.7 [2.7]);<br>p=0.008
					Worse eye at 6 months	Drivers vs. non-drivers	2-sample t-test	Mean (SD) MD of drivers (-5.7 [4.9]) vs. non-drivers (-5.9 [4.0]);<br>p=0.014
							Liner regression	Mean (SD) MD of drivers (-5.7 [4.9]) vs. non-drivers (-5.9 [4.0]);<br>p=0.429
					Worse eye at 54 months		2-sample t-test	Mean (SD) MD of drivers (-5.4 [5.2]) vs. non-drivers (-7.0 [4.9]);<br>p=0.012
							Linear regression	Mean (SD) MD of drivers (-5.4 [5.2]) vs. non-drivers (-7.0 [4.9]);<br>p=0.080
					Mean (SD) difference in VA in worse eye from 6 months to 54 months	Remain drivers vs. became non-drivers	Linear regression	Difference in mean (SD) MD of drivers (0.3 [0.4]), vs. became non-drivers (-1.3 [0.7]);<br>p= 0.013
Keay L et al., 2009	Prospective Cohort	1425 (N/A)	75	USA	Bilateral VF points missing	Whole population	Mean (SD)	Mean(SD) of bilateral VF points<br>missing was statistically

For peer review only

For peer review only - http://bmjopen.bmj.com/site/about/guidelines.xhtml

								significant different between those who stopped driving 9.8(17.1) and those who continued driving 1.98(5.1); p=0.001
Keay L et al., 2016	Cross-sectional	442 (N/A)	73	Australia	Points missed on bilateral VF.	Current drivers with cataracts NOTE: all participants in this study had cataracts.	X^2 (Chi Square)	Median (IQR) of current drivers: 3 (0-10) vs. Median (IQR) of former drivers: 8 (1-19); p= 0.02
Ramulu P et al., 2009	Cross-sectional	1135 (N/A)	79.7	USA	Bilateral VF damage in glaucoma participants	Participants without glaucoma	OR	2 (1.6, 2.5)
							Prevalence (%)	21% (14/68) of participants with bilateral VF loss in the lowest tertile (less than 3 dB of VF loss in better-eye) had stopped driving.<br><br>36% (24/68) of participants with VF loss in the middle tertile (better-eye VF mean deviation between −3 and −9 dB) had stopped driving.<br><br>52% (35/68) of participants with VF loss in the highest tertile (better eye VF mean deviation <-9 dB) had stopped driving.
Segal-Gidan F et al., 2010	Cross-sectional	421 (30/391)	72	USA	Unilateral	Current drivers	OR	1.91 (0.63, 5.76)
		421 (108/318)			Bilateral, mild			2.05 (0.74, 5.66)

For peer review only

		421 (93/328)			Bilateral, moderate/severe			2.84 (0.92, 8.78)
vanLandingham S et al., 2013	Cross-sectional	139 (N/A)	70.1	USA	5 dB worse in the better eye	Glaucoma suspect controls	OR	1.7 (1.1, 2.5); p= 0.008
**NOTE:** There are a range of different study designs as well as cut-off points and areas of VF investigated in the identified studies. Due to methodological differences between each study, meta-analysis was limited and narrative reviews have been used instead to synthesise data.								

*unadjusted results

Table 4b(vii) All studies (n=3) on glare sensitivity (GS) impairment and driving cessation, all suitable to only be summarised narratively due to their different GS cut-off points and comparators

Author and Year	Study Design	Total Participants (exposure/control)	Mean Age	Country	VI Definition	Comparator	Outcome Measure (OR, RR, HR?)	Effect Measure (with 95% CI)
**Included in Narrative Summaries Only – High Income Countries**								
Freeman E et al., 2005	Prospective Cohort	1824 (702/1122)	73.4	USA	3-4 points of glare sensitivity at baseline	≤2 points difference with baseline glare GS	HR	0.78 (0.61, 0.99)
		1824 (206/1618)			≥5 points of glare sensitivity at baseline			0.9 (0.63, 1.28)
		1824 (71/1753)			4 points loss of glares sensitivity in 2 years	<4 points GS loss		1.18 (0.7, 1.99)
		1824 (52/1772)			≥5 points loss of glare sensitivity in 2 years	<4 points GS loss		1.3 (0.72, 2.37)
Gilhotra JS et al., 2001	Cross-sectional	3654 (969/2685)	65.9	Australia	Physician diagnosed	Participants still driving	OR (logistic regression)	1.5 (1.2, 1.8)
Tam A et al., 2018	Cross-sectional	99 (15/84)	71.5	Canada	Physician diagnosed	Still driving participants with cataracts	Prevalence ratio (PR)	4.79; p<0.013

For peer review only

*unadjusted results

Table 4b(viii) All studies (n=11) on other types of vision impairment and driving cessation, all suitable to be summarised narratively only

Author and Year	Study Design	Total Participants (exposure/control)	Mean Age	Country	Type of VI	VI Definition	Comparator	Outcome Measure (OR, RR, HR?)	Effect Measure (with 95% CI)
**Included in Narrative Summaries Only – High Income Countries**									
Campbell MK et al., 1993	Case control	1656 (28/1628)	N/A	USA	Retinal detachment	Self-reported physician diagnosed	Current drivers	Prevalence (%)	Still driving = 14.25%. Not driving = 40.95% Those not driving have a higher percentage of detached retina than those still driving (p<0.05)
					Retinal haemorrhage	Self-reported physician diagnosed		OR	Both genders = 3.86 (1.4, 10.4)*
									Females: 4.70 (1.2, 17.8); p<0.5
					Vision impairment	Self-reported		Prevalence (%)	Still driving = 13.65%. Not driving = 25.34% Those not driving have a higher percentage of other visual loss than those still driving (p<0.01)
DeCarlo D et al., 2003	Cross-sectional	126 (126/0)	79	USA	Maculopathy	exudative or non-exudative	Current drivers	Prevalence (%)	The type of AMD (exudative vs nonexudative) was not significant between the non-drivers and drivers (p=0.474). Nonexudative non-drivers: 50% (48/96), nonexudative drivers : 47% (14/30), exudative non-

For peer review only

									drivers: 50% (48/96), exudative drivers 53% (16/30).
Hajek A et al., 2019	Cross-sectional	549 (192/357)	90.3	Germany	Vision impairment	Severe impairment	Current drivers	OR	0.06 (0.01, 0.59)*
						Mild impairment			0.56 (0.24, 1.35)*
Gallo JJ et al., 1999	Case Control	1920 (N/A)	N/A	USA	Vision impairment	Self-reported	Current drivers	OR	1.86 (0.7, 4.9)
Keay et al., 2016	Cross-sectional	442 (148/294)	73	Australia	URE	Measured with autorefraction and lensometry	Cataract patients who are still driving	X^2 (Chi Square)	No significant differences between current drivers with URE (40% [99/263]) and former drivers with URE (51% [49/110]); p= 0.07
Levecq L et al., 2013	Cross-sectional	1000 (346/654)	71.3	Belgium	Vision impairment	Physician diagnosed	N/A	Prevalence (%)	Among the 190 non-drivers, 47 (24.7%) stopped driving because of their impaired vision.
Marottoli RA et al., 1993	Cross-sectional	1331 (17/1314)	age, no.: 65-74 years = 484, 75-84 years = 105, 85+ years = 6	USA	Vision impairment	Self-reported	Current drivers	Prevalence (%)	Out of the 17 drivers who reported poor vision at baseline (1983), 58.8% (9/17) of drivers who stopped driving by 1989.
Moon SH et a., 2020	Cross-sectional	2970 (1023/1947)	71	South Korea	Vision impairment	Self-reported	Current drivers	OR	0.97 (0.83, 1.14)*
Robinson JL et a., 2021	Cross-sectional	335 (N/A)	67.4	USA	Vision impairment	Self-reported	Current drivers	X^2 (Chi Square)	Participants were less likely to be driving if they had noted vision-related concerns (p<0.001).

For peer review only

Tam A et al., 2018	Cross-sectional	99 (19/80)	71.5	Canada	Dark adaptation in glaucoma patients	Self-reported	Among patients with glaucoma	X^2 (Chi Square)	Dark adaptation significantly associated with driving cessation (p<0.001)
								PR (Prevalence Ratio)	1.47; p= 0.39 Individuals with self-perceived dark adaptation difficulties were not more likely to quit driving.
Zebardast N et al., 2015	Cross-sectional	2469 (132/2337)	73.5	USA	URE	Binocular presenting visual acuity of 20/30 or worse, improving to better than 20/30 with subjective refraction	Participants with normal vision.	OR	2.1 (1.3, 3.6)
					Non-refractive visual impairment	Post-refraction binocular BCVA of 20/30 or worse			3.7 (2.4, 5.7)

*unadjusted results

Table 4b(ix) All studies (n=2; reporting on 4 RCTs in total) evaluating anti-VEGF therapy and driving cessation, suitable for narrative summaries only

**Author and Year**	**Study Design**	**Intervention (n)**	**Control (n)**	**Mean Age**	**Country**	**Vision Impairment**	**VI Definition**	**Comparator(s)**	**Outcome Measure**	**Effect measure (with 95% where appropriate)**
Bressler N et al., 2013	RCT	478	238	77.7	USA	AMD	MARINA trial: minimally	Sham injections or 0.3 mg of Ranibizumab or 0.5 mg of	Prevalence (%) + 95% CI	Among patients who had reported driving at

For peer review only

							classic or occult AMD	Ranibizuman for 24 months		baseline, 74% (146/197) sham patients and 87.8% (156/178) 0.5mg patients reported still driving at 12 months. Among patients who had reported driving at baseline, 67.2% (131/195) (95% CI 59.2-75.2) of sham patients and 78.4% (148/189) (95% CI 71.8-85.0) of 0.5mg ranibizumab patients reported still driving 24 months later.

Bressler N et al., 2013	RCT	280	143	77.7	USA	AMD	ANCHOR: classic neovascular AMD	Verteporfin photodynamic therapy (PDT) or 0.3 mg ranibizumab injections or 0.5 mg ranibizumab injections for 24 months	Prevalence (%) + 95% CI	Among patients who reported driving at baseline, 80.5% (77/96) PDT patients and 94.2% (86/91) 0.5 mg patients reported still driving at 12 months. Among patients who reported driving at baseline, 71.6% (67/94) (95% CI 60.8-82.4) of PDT patients and 91.4% (81/89) (95% CI 85.3-97.5) of 0.5 mg ranibizumab patients reported still driving 24 months later.

For peer review only

Bressler N et al., 2016	RCT	502	257	62.3	USA	DME	RIDE/RISE: any DME	Sham injections or 0.3 mg ranibizumab or 0.5 mg ranibizumab	Prevalence (%) + 95% CI	For 0.3 mg ranibizumab compared to those treated with sham only, there was a 7% (-5.0 to 19) difference in the number of participants now driving (who were not driving at baseline) at 12 months. For 0.5 mg ranibizumab compared to those treated with sham only, there was a 14.4% (1.1, 27.7) difference in the number of participants now driving at 12 months. For 0.3 mg ranibizumab

For peer review only

										compared to those treated with sham only, there was a 12.5% (-0.9, 25.9) difference in the number of participants now driving at 24 months. For 0.5 mg ranibizumab compared to those treated with sham only, there was a 14.3% (0.7, 27.9) difference in the number of participants now driving at 24 months.

For peer review only

Bressler N et al., 2016	RCT	234	111	62.3	USA	DME	RESTORE: DME in a least 1 eye eligible for laser treatment and a VA letter score between 78 and 39	PDT laser only or 0.5 mg + laser or 0.5 mg only	Prevalence (%) with 95% CI	After 12 months, 12.2% (6/49) of those who were not driving at baseline and were treated with 0.5 mg ranibizumab + laser have started driving. Compared to those treated with laser only, there was a 4.2% (-7.7, 16.1) difference in the number of participants now driving at 12 months. After 12 months, 8.9% (4/45) of those who were not driving at baseline and were treated

For peer review only

											with 0.5 mg ranibizumab only have started driving. Compared to those treated with laser only, there was a 0.9% (-10.3, 12.1) difference in the number of participants now driving at 12 months.

Table 4b(x) All studies (n=2) evaluating cataract surgery and driving cessation, suitable for narrative summaries only

**Author and Year**	**Study Design**	**Total Participants (exposure/control)**	**Mean Age**	**Country**	**VI Definition**	**Comparator**	**Outcome Measure (OR, RR, HR?)**	**Effect Measure (with 95% CI)**
Monestam E et al., 2005	Prospective Cohort	810 (N/A)	74.7	Sweden	Physician diagnosed cataracts	All cataract surgery patients, comparing pre and post cataract surgery outcomes.	Prevalence (%)	Before cataract surgery, 55% (224/407) were drivers while after surgery 70% (285/407) were drivers. 5 years after surgery 63% (189/300) of patients with a driving licence were still active drivers. 37% (67/183) of patients who did not drive before surgery started to drive after. 46% (31/67) of patients who did not fulfil the visual requirements for presenting VA and the 35% (24/67) who did not fulfil the requirements for BCVA for a

For peer review only

								legal licence could now legally drive. 82% (40/50) of patents who began to drive after the surgery were still driving 5 years later.
Monestam E et al., 1997	Prospective cohort	211 (N/A)	41	Sweden	Physician diagnosed cataracts	Driving status from all participants pre- and post-surgery.	Ratio (%)	The number of patients driving after surgery increased to 65% (137/211) (from 56%), but this was not significant.

Table 4b(xi) All studies (n=1) evaluating anti-glaucoma therapy and driving cessation

Author and Year	Study Design	Total Participants (exposure/control)	Mean Age	Country	Vision impairment	VI Definition	Comparator	Outcome Measure (OR, RR, HR?)	Effect Measure (with 95% CI)
Stafford WR, 1981	Cross-sectional	240 (N/A)	age, no.: 35-49 years = 11, 50-65 years = 77, >65 years = 139	USA	Glaucoma	Chronic open-angle glaucoma or ocular hypertension that has been adequately controlled for at least	Post- anti-glaucoma therapy outcomes in all participants.	Prevalence (%)	From the 229 patients who stated that the anti-glaucoma therapy side effects affected their

For peer review only - http://bmjopen.bmj.com/site/about/guidelines.xhtml

						the previous 6 months			normal activity, 12% (28/229) said that they had to give up some normal activity. Out of the 28 patients, 16 mentioned giving up driving, particularly at night.

For peer review only

BMJ Open

Associations between vision impairment and vision-related interventions on crash risk and driving cessation: systematic review and meta-analysis

Journal:	*BMJ Open*
Manuscript ID	bmjopen-2022-065210.R1
Article Type:	Original research
Date Submitted by the Author:	15-Mar-2023

Complete List of Authors:	Nguyen, Helen; University of New South Wales School of Optometry and Vision Science<br>Di Tanna, Gian Luca; George Institute for Global Health; University of Applied Sciences and Arts of Southern Switzerland<br>Coxon, Kristy; Western Sydney University - Campbelltown Campus; Western Sydney University Translational Health Research Institute,<br>Brown, Julie; The George Institute for Global Health Injury Division<br>Ren, Kerrie; University of New South Wales School of Optometry and Vision Science<br>Ramke, Jacqueline; London School of Hygiene & Tropical Medicine,<br>Burton, Matthew J; London School of Hygiene & Tropical Medicine<br>Gordon, Iris; London School of Hygiene & Tropical Medicine<br>Zhang, Justine; London School of Hygiene and Tropical Medicine Faculty of Infectious and Tropical Diseases, International Centre for Eye Health, Clinical Research Department<br>Furtado, João; Universidade de São Paulo Faculdade de Medicina de Ribeirão Preto, Division of Ophthalmology<br>Mdala, Shaffi; Queen Elizabeth Central Hospital, Opthalmology Department<br>Kitema, Gatera Fiston; University of Rwanda College of Medicine and Health Sciences, Opthalmology Department<br>Keay, Lisa; University of New South Wales, School of Optometry and Vision Science; The George Institute for Global Health

**Primary Subject Heading**:	Ophthalmology
Secondary Subject Heading:	Public health
Keywords:	PUBLIC HEALTH, OPHTHALMOLOGY, EPIDEMIOLOGY

SCHOLARONE™
Manuscripts

I, the Submitting Author has the right to grant and does grant on behalf of all authors of the Work (as defined in the below author licence), an exclusive licence and/or a non-exclusive licence for contributions from authors who are: i) UK Crown employees; ii) where BMJ has agreed a CC-BY licence shall apply, and/or iii) in accordance with the terms applicable for US Federal Government officers or employees acting as part of their official duties; on a worldwide, perpetual, irrevocable, royalty-free basis to BMJ Publishing Group Ltd ("BMJ") its licensees and where the relevant Journal is co-owned by BMJ to the co-owners of the Journal, to publish the Work in this journal and any other BMJ products and to exploit all rights, as set out in our licence.

The Submitting Author accepts and understands that any supply made under these terms is made by BMJ to the Submitting Author unless you are acting as an employee on behalf of your employer or a postgraduate student of an affiliated institution which is paying any applicable article publishing charge ("APC") for Open Access articles. Where the Submitting Author wishes to make the Work available on an Open Access basis (and intends to pay the relevant APC), the terms of reuse of such Open Access shall be governed by a Creative Commons licence – details of these licences and which Creative Commons licence will apply to this Work are set out in our licence referred to above.

Other than as permitted in any relevant BMJ Author's Self Archiving Policies, I confirm this Work has not been accepted for publication elsewhere, is not being considered for publication elsewhere and does not duplicate material already published. I confirm all authors consent to publication of this Work and authorise the granting of this licence.

For peer review only

Associations between vision impairment and vision-related interventions on crash risk and driving cessation: systematic review and meta-analysis

Authors: Helen Nguyen BMedSci (Hons)[1], Gian Luca Di Tanna PhD[2], Kristy Coxon PhD[3], Julie Brown PhD[2], Kerrie Ren MIPH[1], Jacqueline Ramke PhD[4,5], Matthew J Burton PhD[4,6], Iris Gordon MSc[4], Justine H Zhang MBBS[4,7], João M. Furtado PhD[8], Shaffi Mdala MSc [9], Gatera Fiston Kitema MScPHEC[10], Lisa Keay PhD[1,2]

Affiliations:

1. School of Optometry and Vision Science, Faculty of Science, The University of New South Wales, NSW, Australia

2. The George Institute for Global Health, Faculty of Medicine, University of New South Wales, NSW, Australia

3. School of Health Sciences, and the Translational Health Research Institute, Western Sydney University, NSW, Australia

4. International Centre for Eye Health, London School of Hygiene & Tropical Medicine, London, United Kingdom, WC1 E7HT

5. School of Optometry and Vision Science, University of Auckland, Auckland, New Zealand

6. National Institute for Health Research Biomedical Research Centre for Ophthalmology at Moorfields Eye Hospital NHS Foundation Trust and UCL Institute of Ophthalmology, London, UK

7. Royal Free Hospital, London, United Kingdom

8. Division of Ophthalmology, Ribeirão Preto Medical School, University of São Paulo, Ribeirão Preto, São Paulo, Brazil

9. Ophthalmology Department, Queen Elizabeth Central Hospital, Blantyre, Malawi

10. Ophthalmology Department, School of Health Sciences, University of Rwanda, Kigali, Rwanda

Corresponding Author: Lisa Keay

Postal Address: School of Optometry and Vision Science, The University of New South Wales, Sydney UNSW NSW 2052, Australia

Email: l.keay@unsw.edu.au

For peer review only - http://bmjopen.bmj.com/site/about/guidelines.xhtml

ABSTRACT

Objectives: To systematically investigate the associations between vision impairment and risk of motor vehicle crash (MVC) involvement, and evaluate vision-related interventions to reduce MVCs.

Design: Medline (Ovid), EMBASE and Global Health electronic databases were systematically searched from inception to March 2022 for observational and interventional English-language studies. Screening, data extraction and appraisals using the JBI appraisal tools were completed by two reviewers independently. Where appropriate, measures of association were converted into risk ratios (RRs) or ORs (odd ratios) for meta-analysis.

Participants: Drivers of four-wheeled vehicle of all ages with no cognitive declines.

Primary and secondary outcomes: MVC involvement (primary) and driving cessation (secondary)

Results: 101 studies (n=778,052) were included after full-text review. 57 studies only involved older drivers (≥65 years) and 85 were in high-income settings. Heterogeneity in the data meant that most meta-analyses were underpowered as only 25 studies, further split into different groups of eye diseases and measures of vision, could be meta-analysed. The limited evidence from the meta-analyses suggests that visual field defects (four studies; RR 1.51 (95%Cl 1.23-1.85); p<0.001; I^2=46.79%), and contrast sensitivity (two studies; RR 1.40 (95%Cl 1.08-1.80); p=0.01, I^2=0.11%) and visual acuity loss (5 studies; RR 1.21 (95%Cl 1.02, 1.43); p=0.03, I^2=28.49%) may increase crash risk. The results are more inconclusive for available evidence for associations of glaucoma (five studies, RR 1.27 (95%Cl 0.67-2.42); p=0.47; I^2=93.48%) and cataract (two studies RR 1.15 (95%Cl 0.97-1.36); p=0.11; I^2=3.96%) with crashes. Driving cessation may also be linked with glaucoma (two studies; RR 1.62 (95%Cl 1.20-2.19); p<0.001, I^2=22.45%), age-related macular degeneration (AMD) (three studies; RR 2.21 (95%Cl 1.47-3.31); p<0.001, I^2=75.11%) and reduced contrast sensitivity (three studies; RR 1.30 (95%Cl 1.05-1.61); p= 0.02; I^2=63.19%). Cataract surgery halved MVC risk (three studies; RR 0.55 (95%Cl 0.34-0.92); p= 0.02; I^2=97.10). Ranibizumab injections (four RCTs) prolonged driving in persons with AMD.

Conclusion: Impaired vision identified through a variety of measures is associated with both increased MVC involvement and cessation. Cataract surgery can reduce MVC risk. Despite literature being highly heterogeneous, this review shows that detection of vision problems and appropriate treatment is critical to road safety.

PROSPERO registration: CRD42020172153

For peer review only

Strengths and Limitations of this Study

- This is an up-to-date systematic review capturing literature on a variety of eye diseases and conditions, measures of vision such as visual acuity, contrast sensitivity, glare sensitivity, and visual field, and vision-related interventions and their associations on MVC involvement and driving cessation.
- There were no geographical or age restrictions placed on the population of focus allowing the global impact of vision impairment on driving to be documented for all age groups.
- Meta-analysis was limited due to heterogeneity in the outcome measures reported and the definitions of vision loss and or impairment used in each study. This heterogeneity also prohibited sub-group analyses by age and geographic location.
- Only statistical heterogeneity was assessed and not clinical or methodological.
- Publication bias was not assessed as there were less than ten studies included in each meta-analysis.

For peer review only

INTRODUCTION

Globalisation and economic development have made driving one of the main modes of transport worldwide and passenger vehicle travel is predicted to triple between 2015 and 2050.[1] Driving allows for independent mobility and enhances access to employment and education. Unfortunately, with more drivers on the roads, motor vehicle crashes (MVCs) and road traffic injuries are increasing worldwide. Approximately 1.35 million MVC-related fatalities occur each year with an additional 20-50 million people experiencing road-related injuries per annum.[2] The United Nations have therefore created targets within the Sustainable Development Goals (SDG) which aim to halve road deaths by 2020 (Target 3.6) and provide safe and sustainable transport systems for vulnerable road users (Target 11.2).[3]

Driving is a common and valued activity for many adults. Driving cessation limits independent mobility and has been linked to depressive symptoms and poorer health in older adults.[4] Functional declines in vision disproportionately impact older drivers, as they have higher prevalence of poor vision and eye diseases.[5, 6] Some countries have specific licensing requirements for older drivers,[7] however variations in visual driving standards across jurisdictions have made it difficult to assess whether these standards have safety benefits.[8]

This review was completed in collaboration with the *Lancet Global Health Commission* on Global Eye Health[9] and aimed to systematically evaluate the evidence to (1) investigate the associations between vision impairment and risk of MVC involvement across the lifespan, and (2) evaluate vision-related interventions to reduce MVCs. Since risks can be mitigated by driving retirement, this review also considered driving cessation as a secondary outcome.

METHODS

This systematic review was reported using PRISMA guidelines[10] (*Appendix 1*) using a published protocol.[11] An electronic database search on Medline (Ovid), EMBASE and Global Health was conducted from their inception to March 2020, and then updated in March 2022, with no geographic restrictions. *Appendix 2* details the search strategy with *Table* 1 describing the inclusion and exclusion criteria for studies.

For peer review only

Table 1 Study inclusion and exclusion criteria

Inclusion	Exclusion
• Interventional (RCTs) and observational (cohort, cross-sectional, case-control and case series) studies.<br>• Systematic reviews with meta-analyses<br>• Studies on drivers of four-wheeled motorised vehicles of all ages.<br>• Studies looking at the following exposures of interest: impairment in measures of vision (visual acuity (VA), contrast sensitivity (CS), visual field (VF), and glare sensitivity (GS)) or specific eye conditions including but not limited to glaucoma, cataracts, age-related macular degeneration (AMD), diabetic retinopathy (DR), stereopsis disorders and colour vision deficiencies.<br>• Studies on interventions such as vision screening, refractive correction, cataract surgery, anti-VEGF injections and other treatments to improve vision.	• Literature reviews and narrative systematic reviews.<br>• Commentary articles, dissertations, abstracts, editorials and conference presentations.<br>• Studies using simulators or investigated either self-regulatory driving behaviours (e.g. night driving avoidance), or self-reported measures of driving safety.<br>• To narrow the scope of the study, studies on populations with specific non-vision related medical conditions (e.g. dementia, epilepsy, stroke and history of medical events such as syncope), low vision or vision difficulties caused by other medical conditions (e.g. hemianopia caused by brain damage).<br>• Studies which simulated vision impairment.

The population of focus was drivers of four-wheeled motorised vehicles, of all ages, with no cognitive declines. Exposures of interest included eye diseases (e.g. glaucoma, cataract, age-related macular degeneration, diabetic retinopathy) and conditions (e.g. refractive errors), and measures of vision such as, but not limited to, visual acuity and contrast sensitivity. Studies reporting on interventions focused on treatments that would improve vision. The primary outcome measure was MVC involvement identified from self-reported surveys or government/hospital administrative datasets. The secondary outcome was self-reported driving cessation. Due to the large volume of data collected, other surrogate measures of driving safety and driving performance planned in the original protocol were beyond the scope of this manuscript but will be reported in a separate systematic review.[11] Studies which used simulators, or investigated self-regulatory driving behaviours (e.g. night driving avoidance) through surveys were excluded.

All titles, abstracts, and full-texts were reviewed independently by two investigators using Covidence systematic review management software (Covidence non-profit SaaS Enterprise, Melbourne, Australia). All discrepancies were resolved via consultation with a third investigator. Similarly, data extraction was completed independently by two investigators using data extraction forms adapted from either the Joanna Briggs Institute (JBI) templates for observational and systematic review study designs, or Cochrane templates for interventional studies. Data extracted from the studies included design, participant and setting characteristics, exposure type and definition, intervention details (if any), outcome measures, and relevant effect measures.

Overall risk of bias for all included studies was assessed by two investigators independently with conflicts resolved by a third investigator. All quality assessments were conducted using the relevant

For peer review only - http://bmjopen.bmj.com/site/about/guidelines.xhtml

JBI critical appraisal tools.[12] Each question on the relevant tools were categorised into either selection, detection, confounding, validity, performance, attrition, or allocation bias by all authors. Thus, a range of biases were considered appropriate to this research question. Each study was given an overall "score" on each question answered where a higher score represented less bias in the study design and execution. Based on how the questions were asked, a "yes" indicated that some sort of measure to limit bias was undertaken. The final scores were used to assign each study as low, medium, or high risk of bias, with lower scores indicating higher risk of bias.

Statistical Analysis

Associations between vision impairments and vision-related interventions with MVC involvement and driving cessation were summarised with appropriate Hazard Ratios (HR), Risk Ratios (RR), or Odds Ratios (OR). Narrative summaries were reported using the SWiM guidelines.[13] Heterogeneity across studies were assessed using I^2 statistic. Meta-analysis was conducted by converting all effects measures into RR or OR. Random-effects meta-analysis was only conducted on studies which presented data with the same outcomes, exposures and comparators, and which reported on associations adjusted for confounders to reduce bias. Data from case-controls were not pooled for meta-analysis to minimise possible heterogeneity. No publication bias analysis was conducted as there were less than 10 studies in each meta-analysis. Reporting of the results were guided by the Meta-analysis of Observational Studies in Epidemiology (MOOSE) guidelines.[14] All analyses were completed using STATA v17.

Patient and Public Involvement

Only existing published literature were looked at in this review and therefore no patient or public involvement was present during the design or execution of the review. Public participation may be sought out for future dissemination of this review.

RESULTS

From the electronic database search, 5111 studies were identified after the removal of 2131 duplicates. After title and abstract screening, 243 studies remained for full-text review after which 142 studies were further excluded, leaving 101 studies for data extraction (*Figure 1*).

63 studies (31 cross-sectional, 19 cohort, 12 case-control and one systematic review with meta-analysis) reported on MVC involvement alone, 34 (21 cross-sectional, eight cohort, two case-control, one case series, and two RCTs) on driving cessation, and four (one cross-sectional, two cohort, and one case-control) on both MVC and cessation. When split by geographic regions, 48 studies from HICs and 15 studies from LMICs reported solely on MVC involvement, whilst all 34 studies looking at driving cessation only came from HICs. From the studies which reported on both MVC and driving cessation, only one was from a LMIC. Study breakdown according to driving outcome and vision impairment is shown in *Table 2(a-b)*. The majority of studies (84%) were set in HICs and 57 studies (56%) focused on older adults. However, when looking at the 16 studies set in LMICs, all but two had an average study population age of less than 65 years. From the total 101 studies, only 13 (seven from HICs, six from LMICs; 12 cross-sectional, one cohort) were categorised as high risk of bias with the rest rated as either low or medium (*Appendix 3*).

For peer review only - http://bmjopen.bmj.com/site/about/guidelines.xhtml

Table 2a Breakdown of studies reporting on vision-related associations by outcome measure

Driving Outcome	Vision Impairment	Region (HIC/LMIC)	Total No. of Studies
**Motor Vehicle Crash (MVC)**	Glaucoma	15 HICs; 1 LMIC	16
	Cataract	8 HICs	8
	AMD	6 HICs	6
	Diabetic Retinopathy	3 HICs	3
	Stereopsis Impairment	2 HICs; 3 LMICs	5
	Myopia	2 HICs; 2 LMICs	4
	Colour Blindness	1 HICs; 7 LMICs	8
	Contrast Sensitivity	13 HICs	13
	Visual Acuity	19 HICs; 9 LMICs	28
	Glare Sensitivity	3 HICs	3
	Visual Field Impairment	14 HICs; 6 LMICs	20
	Other†	13 HICs; 6 LMICs	19
**Driving Cessation**	Glaucoma	12 HICs; 1 LMIC	13
	Cataract	5 HICs	5
	AMD	5 HICs	5
	Contrast Sensitivity	8 HICs	8
	Visual Acuity	18 HICs	18
	Glare Sensitivity	3 HICs	3
	Visual Field Impairment	8 HICs	8
	Other‡	11 HICs	11

†unilateral vision impairment, general vision impairment, retinopathy, retinal detachment, poor visibility, refractive disorder, monocular vision impairment, hyperopia, amblyopia, diplopia, astigmatism, retinitis pigmentosa, stereoacuity

‡dark adaptation, age-related maculopathy, detached retina, non-refractive vision impairment, self-reported vision loss, retinal haemorrhage, uncorrected refractive error

For peer review only

For peer review only - http://bmjopen.bmj.com/site/about/guidelines.xhtml

2b Breakdown of studies reporting on a vision-related intervention by intervention type, vision impairment and outcome measure

Intervention	Vision Impairment	Driving Outcome	Region (HIC/LMIC)	No. of Studies
**Anti-VEGF Injections**	AMD	Driving Cessation	1 HIC	1
	Diabetic Macular Oedema (DME)		1 HIC	1
**Cataract Surgery**	Cataract	Motor vehicle crash	6 HICs	6
		Driving Cessation	2 HICs	2
**Corrective Lenses**	Refractive error	Motor vehicle crash	1 HICs	1
**Anti-glaucoma therapy**	Glaucoma	Driving Cessation	1 HICs	1

Raw data on studies reporting on MVCs[15-81] and driving cessation[70-73, 82-115] can be found in *Appendix 4a(i-xiv)* and *4b(i-ix),* respectively, with additional narrative summaries. Meta-analyses on associations are presented in *Appendix 5a-b*; only 25 studies could be meta-analysed. Studies were not included in the meta-analysis if different comparators were used, different driving outcomes were analysed (any MVC involvement, at-fault MVCs, injurious and non-injurious MVCs), or different cut-off points or definitions for vision impairment. For example, there were studies that looked at bilateral VA at 6/12 and worse while there were others that looked at unilateral VA being "poor" but without a formal definition of what "poor" acuity meant. Studies rated as having a high bias were also excluded from the meta-analyses. *Figure 2* synthesises the narrative summaries to show multiple associations of vision with MVCs and driving cessation. From *Figure 2* it can be seen that associations reported for eye diseases and measures of vision function were more consistent across studies looking at cessation compared to crashes. When considering vision-related interventions, only cataract surgery was shown to improve driving by minimising crash risk. The benefits of anti-VEGF injections on prolonging driving was more inconclusive and found to only help drivers with AMD but not diabetic macular oedema. However as a whole, the evidence from the literature on associations between vision impairment and crashes and cessation are mostly inconclusive and or mixed.

<u>Associations between eye diseases and conditions/measures of vision loss and motor vehicle crashes</u>

The results were mixed (16 studies, n=21,214 participants) for associations between glaucoma and MVCs.[24, 30, 38, 41, 43, 45, 46, 52, 54, 65, 67-72] As illustrated in *Appendix 5 2a*, meta-analyses found a glaucoma diagnosis to not increase the risk of any MVC involvement (OR 1.27 (95% CI 0.67-2.42); p=0.47) however this estimate has a wide confidence interval limiting the power to investigate this association.[24, 30, 38, 43, 72] Other studies were excluded from the meta-analysis as there was no similarity on the comparators used, how glaucoma was categorised (mild vs. severe, unilateral vs. bilateral) and the crash outcomes investigated (any MVC involvement, injurious vs. non-injurious, at-fault). Similarly, meta-analyses on three studies[24, 30, 43] looking at at-fault crashes also found no difference between drivers with and without glaucoma (RR 1.89 (95% CI 0.40-8.86); p=0.42). Increased risk was evident with more severe glaucoma.[30, 38, 43, 46, 52, 65, 69, 70]

For peer review only - http://bmjopen.bmj.com/site/about/guidelines.xhtml
For peer review only

Out of the eight cataract studies (n=18,883) identified,[24, 40, 41, 45, 54, 56, 57, 72] most found self-reported, physician-diagnosed cataracts did not impact the likelihood of any type of MVC involvement. Meta-analysis suggests that were no increased risk (*Appendix 5a*; OR 1.15 (95% CI 0.97-1.36); p=0.11);[24, 40] however this was underpowered with only two studies used for analysis. At-fault crash involvement was investigated by two studies however only one reported significant associations.[24, 56]

Meta-analysis could not be conducted on any studies looking at drivers with either AMD (five studies, n=4150)[24, 41, 44, 64, 66] or diabetic retinopathy (3 studies, n=4353),[24, 45, 54] however no studies found increased risk of MVC. No studies were meta-analysed as studies on AMD all had different comparator or different grades of AMD and MVC type, while studies on DR had different comparators and looked at different crash outcomes.

Impairments in stereopsis were not found to increase the risk of MVC involvement across the five studies identified (n=3253).[22, 33, 40, 51, 75] Meta-analysis on three studies showed no difference in crash involvement between those with and without stereopsis impairment (*Appendix 5a*; RR 1.03 (95% CI 0.86-1.23); p=0.74).[22, 40, 51]

Summary of studies on myopia (four studies, n= 2039)[22, 23, 41, 74] also found no increased risk of MVC involvement. A combination of two of these studies in meta-analysis (*Appendix 5a*) also did not find evidence of an association (OR 0.76 (95% CI 0.34-1.70; p=0.51),[22, 74] noting limitation of sample size for concrete conclusions to be made. One study investigating persons with night myopia reported slightly more night-time MVCs in these drivers than those without night myopia (p=0.044).[23]

Colour vision deficiency and the risk of MVC involvement among commercial truck drivers was investigated in eight studies (n=7916);[15, 21, 22, 34, 51, 53, 59, 77] seven set in LMICs. Three studies found an association,[15, 51, 59] however their results were not combined due to reliance on Ishihara plates which do not reliably diagnose colour vision deficiency.

Visual acuity (VA) (28 studies, n=39,129) was not found to be associated with crash involvement by 19 studies,[17, 20, 22, 24, 27-29, 31, 33-36, 38, 40, 41, 45, 50-54, 57, 63, 68, 69, 73, 75, 77, 80] irrespective of crash scenario (at-fault or not-at-fault) and severity (injurious or non-injurious). Bilateral VA 20/40 or worse may impact risk of MVCs (meta-analysis five studies; RR 1.21 (95% CI 1.02-1.43); p= 0.03).[27, 31, 40, 73, 77] Combining two studies found no evidence for an associations with 'not at-fault' MVCs (RR 1.08 (95% CI 0.74-1.60); p= 0.68) (*Appendix 5a*),[27, 31] however there was limited power to explore associations.

Mixed results were reported from 13 studies (n=17,941) looking at any MVC involvement and reduced contrast sensitivity (CS).[24, 27, 31, 35, 38, 40, 54, 57, 58, 73] However, due to heterogeneity in outcome measures reported and definition of reduced CS, the meta-analysis in *Appendix 5a* was restricted to only two studies which found CS to increase crash risk (RR 1.40 (95% CI 1.08-1.80); p=0.01).[31, 79] When photopic and mesopic areas under the log contrast sensitivity were investigated with any and at-fault crash involvement, only lower mesopic peaks were found to be predictive.[58]

From the 20 studies (n=13,533) looking at visual field (VF) loss and crashes, heterogeneity in the definition of VF loss and the crash outcomes investigated meant that only four were meta-analysed. The results suggests an increased risk of MVC with bilateral field loss (RR 1.51 (95% CI 1.23-1.85); p< 0.001) (*Appendix 5a*).[32, 51, 77, 79] There were mixed results with 9/20 studies finding an increased risk,[31, 32, 38, 42, 54, 73, 77-79] 1/20 an association for a co-linear dependent variable

For peer review only - http://bmjopen.bmj.com/site/about/guidelines.xhtml

[19] and 10/20 a null finding.[16, 17, 33, 34, 37, 51, 53, 59, 68, 69] The increased risks were found in association with severe, bilateral VF loss and field loss affecting both central and peripheral vision.

Most studies on glare sensitivity impairments (3 studies, n=3191) found weak to no associations with crash risk;[54, 57, 73] they were unable to be meta-analysed.

19 studies (n=100167) reported on other impairments including: unilateral vision impairment,[18] general vision impairment,[21, 25, 28, 39, 41, 59, 61, 74, 76, 80, 81] non-diabetic retinopathy,[41] retinal detachment,[72] other retinal disorders,[41] refractive disorder,[41] monocular vision impairment,[41, 50] presbyopia,[41, 74] hyperopia,[22, 74] amblyopia,[18, 60] diplopia,[41] astigmatism,[22, 41] retinitis pigmentosa[26] and stereoacuity.[54, 73] Most did not find associations with MVCs, however one study from the USA reported increased injurious MVC involvement with impaired stereoacuity.[54] Another study in the UK reported increased MVC involvement with moderate/severe amblyopia[41] whilst two other studies, one in Ethiopia[21] and the other in Bangladesh,[74] reported increased MVC involvement with self-reported bilateral visual impairment.

Impact of vision-related interventions on motor vehicle crashes

Most of the six studies (n=592,897) on cataract surgery found the risk of MVC to decrease following cataract surgery[41, 47-49, 55, 62] and the 3 studies suitable for meta-analysis estimated the risk to halve (RR 0.55 (95% Cl 0.34-0.92); p=0.02) (*Appendix 5a*).[47, 48, 55] Greater reductions to crash risk are seen after first eye surgery compared to second eye.[47] Similarly, the risk of crashing in males post-surgery is lower than females.[49]

Corrective lenses for far and near vision refractive disorders were only investigated by one study which found no associations with crash risk.[41]

Associations between eye diseases and conditions/measures of vision loss and driving cessation

There were 13 studies (n=21,939) investigating associations between glaucoma and the likelihood of driving cessation with estimates ranging from an increased risk of 1.3 to increased odds of 4.[70-72, 87, 91, 92, 99, 100, 103, 109-111, 113] The meta-analysis in *Appendix 5b* suggests a diagnosis of glaucoma to increase the risk of driving cessation by 63% (95% Cl 1.20 - 2.19; $p< 0.01$),[87, 91] however this analysis only contained two studies.

Four studies (n=14,402) looked at cataract and driving cessation with three studies reporting an increased likelihood of driving cessation by over 1.5 times; none could be meta-analysed.[72, 99, 100, 106]

From the five studies (n=6183) identified,[85, 87, 99, 106, 108] three found the presence of AMD to be predictive of driving cessation, with meta-analysis on three suitable studies reporting the overall risk of cessation to increase by 2.21 (95% Cl 1.47-3.31; $p< 0.01$) (*Appendix 5b*).[85, 87, 108]

Even though the 18 identified studies (n=23,712) were highly heterogeneous,[73, 82, 86-88, 90, 91, 94-98, 103-106, 110, 111] impaired or “poor” VA was shown to increase the chances of driving cessation in most studies,[87, 103, 104, 106, 111] with better visual acuity decreasing the risk of cessation by up to 70%.[90] The two studies looking at visual acuity in persons with glaucoma, had mixed conclusions on the effect of visual acuity on driving cessation.[95, 110]

Eight studies (n=9602) looked at the impact of CS on driving cessation.[73, 88, 94, 96, 97, 103, 106, 111] From the studies which categorised contrast sensitivity as “poor”, meta-analysis found poor CS to increase the risk of cessation (RR 1.30 (95% Cl 1.05-1.61); p=0.02) (*Appendix 5b*).[94, 96, 106] Another study reported participants who had a decline of 6 or more letters in their contrast

For peer review only - http://bmjopen.bmj.com/site/about/guidelines.xhtml

sensitivity levels after two years, as measured by a Pelli-Robson chart, to have a 71% increased risk of driving cessation.[88]

VF loss and driving cessation was investigated by 8 studies (n=7988),[88, 94-97, 103, 105, 111] and all but one found associations.[105] The likelihood of cessation was generally greater with bilateral and or more severe field loss.[88, 94, 111] One study looking at persons with bilateral glaucoma found visual field loss to double the odds of cessation.[103]

Glare sensitivity (3 studies, n=5577) and was not found to be consistently associated with driving cessation.[88, 91, 110]

Eleven studies (n=12,897) looked at driving cessation with other types of vision impairment: dark adaptation,[110] age-related maculopathy,[86] retinal detachment,[85] non-refractive vision impairment,[112] general vision loss,[85, 89, 93, 98, 100, 114, 115] retinal haemorrhage,[85] and uncorrected refractive error.[97, 112] Only two studies, one reporting on retinal haemorrhage[85] and the other on non-refractive vision impairment and uncorrected refractive error,[112] found increased risk of driving cessation.

Impact of vision-related interventions on driving cessation

There were two studies reporting the driving status of participants after anti-VEGF therapy (0.5 mg ranibizumab) from 4 different RCTs; MARINA (n=716; 24 months; control=sham injections) and ANCHOR (n=423; 24 months; control=photodynamic therapy (PDT)) which targeted AMD,[83] and RIDE/RISE (n=759; 24 months; control=sham injections) and RESTORE (n=345; 12 months; control=PDT) which targeted diabetic macular oedema (DMO).[84] By the end of all four trials, only drivers with AMD but not DMO treated with anti-VEGF were shown to have marked differences with the control group for the number of people who continued driving from baseline (AMD: MARINA: p=0.035, ANCHOR: p=0.002; DMO: RIDE/RISE: p=0.655, RESTORE: p=0.125).

Both studies (n=1021) looking at driving status after cataract surgery reported an increase in the proportion of participants driving after successful surgery.[101, 102]

There was only one study (n=240) looking at driving after anti-glaucoma therapy (pilocarpine-epinephrine),[107] however this is an old study and this treatment is no longer in use.

DISCUSSION

This review synthesises diverse and complex evidence from 101 studies examining vision and its impact on MVCs and driving cessation across all ages. The majority of studies in this review focused on older adults and reported more associations between vision impairment and MVCs and or cessation compared to studies on younger populations. Research was mostly observational with few studies examining the impact of interventions to improve vision. The studies excluded from the meta-analysis tended to have mixed results regarding the associations between the vision impairment and driving outcome, whereas the studies in the meta-analyses were more consistent showing definitive associations for VA, CS and VF defects. Nonetheless, the mixed results in the narrative summaries however support the emerging idea of adding visual processing and cognitive tests alongside visual assessments to produce more predictive measures of safe driving.[116] When looking at the vision-related interventions, cataract surgery was shown to halve the risk of crashing. Others have reported that following cataract surgery, driving difficulties, such as self-reported night driving ability, reduced by 88%[117] with improvements in contrast sensitivity linked to these changed perceptions.[118]

For peer review only - http://bmjopen.bmj.com/site/about/guidelines.xhtml

Variability in the relationships between vision and MVCs may be due to several reasons. The first set of reasons surround how MVCs are defined and investigated in the literature. Firstly, there are many different MVC scenarios based upon the driver's role (at-fault or not) and severity (injurious or non-injurious) which are not always differentiated in research studies. MVCs are also studied in a variety of ways from self-reports to analyses of large crash databases. This may cause reliability issues. For example, an American study found agreement between these two collection methods was poor when examining the total MVCs over a three-year period.[119] Crashes can also stem from external and vehicular factors which make drawing conclusions solely based on human factors inappropriate.[120] Self-regulation, jurisdictional control on vision standards for licensing, and driving cessation could all mitigate the risk of crash involvement. The second set of reasons have to do with the vision impairment themselves and the severity of the impairment. The studies which reported increase crash risk, associated with diagnosis of an eye disease, evaluated more severe forms of the disease and worse functions of vision. Studies examining impact of a diagnosis of a disease tended to report no associations. For example, the lack of association between a diagnosis of cataract and MVC could be because the cataract is mild and is not having a significant impact on CS. A parallel review from our group has found greater defects in these measures to worsen driving performance and increase errors, which can theoretically lead to more crashes.[121] It is therefore critical to capture the severity of an eye disease and/or the actual level of vision impairment when impact of disease status on crash risk . As seen in this review, even though glaucoma, cataract and AMD had mixed or no associations with crashing, their corresponding measures of vision, mainly VF, CS and VA respectively, were definitively associated. This may be why associations found between vision impairment and driving cessation were strong and consistent. A diagnosis of glaucoma or AMD, and poor contrast sensitivity were all found to increase the risk of driving cessation. Anti-VEGF injections could prolong driving for people with AMD. This is of importance as older adults greatly value independent mobility and regard driving as a vital activity for daily living.[122, 123] With driving cessation linked towards multiple negative health outcomes in older adults,[4] anti-VEGF injections can have wider health benefits beyond direct impact on vision.

This review also highlights the paucity of research from LMICs despite approximately 93% of all road-traffic related deaths occurring in these countries, particularly in Africa and amongst young road users.[2] Despite the UN's push, most LMICs still lag behind the SDG targets on halving road traffic mortality set in the Decade of Action for Road Safety (2011-2020).[124] Previous systematic reviews point towards legislation-based interventions which modify behaviour, such as seat-belt and helmet use, to be the most effective at reducing road injuries and crash rates in LMICs.[125, 126] These interventions are in line with UN recommendations for improving infrastructure, vehicle safety standards, and safe road user behaviours in order to reach the targets set for SDG 3.6 and 11.2.[127] However, there is no mention of licensing standards which needs to be addressed as motorisation increases worldwide. Evidence from this global review supports vision standards for licensing to be updated, enforced and given higher priority in LMICs. Even though most LMICs do have guidelines on vision, especially for commercial drivers, it is apparent from the studies in this review that many drivers unfortunately do not satisfy these conditions. This may be because many people in LMICs lack access to eye-health care services. The evidence for a corresponding increase in MVCs in LMICs is not well established with only one systematic review identified looking at data from these regions.[77] Though data from HICs can inform research and policy development in LMICs, increasing the evidence base from LMICs will ensure that interventions to reduce MVCs and maintain access to driving in LMICs can be reflective of the local context.

Older drivers tend to self-regulate their driving habits by reducing their driving mileage and radius and avoiding high-risk driving situations.[128] Vision impairments have been reported to increase

For peer review only - http://bmjopen.bmj.com/site/about/guidelines.xhtml

the likelihood of self-regulation by 19%,[129] with older drivers who self-rate their vision as "poor" 15 times more likely to modify their driving than those who regard their vision as "excellent".[123] Our findings are consistent with these patterns of self-regulation and a diagnosis of AMD or glaucoma were found in this review to be associated with driving cessation. It is likely that self-regulation is an intermediate step towards driving cessation encompassing reductions in driving frequencies and distance.[130] However, self-regulation has been reported as an insufficient compensatory measure to reduce crash risk amongst older drivers with a vision impairment,[131, 132] which would therefore explain why glaucoma, particularly more severe glaucoma, was still linked with crashes in some studies. The relationship between crash involvement and AMD, however, was inconclusive. This may be because AMD affects central vision, thus making declines in this field easily noticeable allowing individuals to appropriately adapt their driving behaviours. Laboratory studies simulating central vision impairments show negative impacts on driving performance and safety, particularly with increasing age and distraction.[133] Further research is needed on driving patterns and behaviours of individuals with eye diseases.

Few studies, all from LMICs, in this review reported associations between colour vision deficiency and crash risk. Unfortunately, based upon their high risk of bias, these studies were deemed unsuitable for meta-analysis. This does not mean, however, that their results should be dismissed. Previous simulation studies found persons with colour vision deficiency performed worse in driving simulations compared to those with normal colour vision.[134-136] However, these associations have not always been evident in studies of MVC risk.[137] This might be why recommendations proposed by the Commission Internationale de l'Eclairage, the international authority on lighting and signal lights, are for commercial drivers only.[135] Associations found in LMICs highlight issues regarding poor road infrastructure and lighting standards.[138] Further research is needed, with standardised diagnosis of colour vision deficiency and consideration of improvements to lighting and signals in the road environment in LMICs.

This review summarises global data on different eye diseases, declines in vision function and vision-related interventions, which makes the findings applicable worldwide considering motorisation and ongoing issues of vision loss, particularly in older people. There are, however, limitations which should be acknowledged. This review highlights the highly heterogeneous nature of research investigating the impact of vision on driving which unfortunately presented several methodological limitations. Firstly, only a small number of studies could be synthesized for meta-analyses due to differences in study design. The underpowered meta-analyses meant that no absolute conclusions can be made from these results alone. It is therefore imperative that the meta-analyses results be considered alongside the narrative summaries to gain a full picture of the literature in this field. There were great variations in the comparator group used in each study and there were inconsistent cut-off points amongst studies looking at continuous measures of vision function. This heterogeneity also prevented sub-group analyses comparing younger to older age groups and geographic regions. Clinical and methodological heterogeneity could not be investigated, even though details on participant characteristics, relevant interventions and study designs were collected, due to the small number of studies included in each meta-analysis. Looking at these parameters, however, might have explained the high statistical heterogeneity in select meta-analyses. The published meta-analysis, however, was summarised narratively to ensure duplicate studies were not included in this evidence synthesis. Grey literature and non-English studies were not included which may have introduced publication bias and limited the number of studies identified from LMICs. Future research incorporating these areas may provide a clearer picture on how vision impairment is affecting global road safety.

For peer review only - http://bmjopen.bmj.com/site/about/guidelines.xhtml

In conclusion, this review summarises the global literature on the impact of vision and vision-related interventions on driving as part of the *Lancet Global Health* Commission on Global Eye Health. Select measures of vision impairment such as visual field, visual acuity and contrast sensitivity loss, and eye diseases such as glaucoma and AMD, were found to be associated with either crashes or driving cessation, while interventions such as cataract surgery and anti-VEGF injections mitigated these outcomes. However, the current literature is highly heterogeneous, and more studies are needed from LMICs to ensure that what is known about vision and driving in these settings. Future studies should aim to address these issues to allow for the global context of vision impairment and driving safety to be better documented, which may assist in the achievement of the UN's SDG road safety targets.

AUTHORS' CONTRIBUTIONS

HN, LK and JR conceived the idea of for the review. IG constructed the search. Title and abstract screening and data extraction were completed by HN, KR, JR, JHZ, JF, SM and GFK. HN drafted and revised the manuscript with suggestions from LK, JR, JB, KC, GLDT, KR, MJB, JHZ, IG, JF, SM and GFK who received the manuscript and provided feedback on the draft. The final version of the manuscript was approved by all authors.

FUNDING STATEMENT

HN is supported by the Australian Government Research Training Program (RTP) Scholarship (N/A). MJB is supported by the Wellcome Trust (207472/Z/17/Z). JR's appointment at the University of Auckland is funded by the Buchanan Charitable Foundation (N/A), New Zealand. The *Lancet Global Health* Commission on Global Eye Health was supported by grants from The Queen Elizabeth Diamond Jubilee Trust (N/A), Moorfields Eye Charity (GR001061), NIHR Moorfields Biomedical Research Centre (N/A), The Wellcome Trust (20190426_PH2), Sightsavers (N/A), The Fred Hollows Foundation (N/A), SEVA Foundation (N/A), British Council for the Prevention of Blindness (N/A), and the Christian Blind Mission (N/A).

ACKNOWLEDGEMENTS

None

COMPETING OF INTERESTS

None declared

ETHICS APPROVAL

Not applicable

DATA SHARING

All data relevant to the study are included in the article or uploaded as supplementary information.

REFERENCES

1. Acker A, Jari K. How transport demand will change through 2050 2019 [Available from: https://www.oecd-ilibrary.org/sites/transp_outlook-en-2019-

For peer review only - http://bmjopen.bmj.com/site/about/guidelines.xhtml

en/1/1/4/index.html?itemId=/content/publication/transp_outlook-en-2019-en&_csp_=1b3375008054c148f41fef71cd42b552&itemIGO=oecd&itemContentType=book.
2. World Health Organisation. Road Traffic Injuries 2020 [Available from: https://www.who.int/news-room/fact-sheets/detail/road-traffic-injuries.
3. United Nations. #Envision2030: 17 goals to transform the world for persons with disabilities 2015 [Available from: https://www.un.org/development/desa/disabilities/envision2030.html.
4. Chihuri S, Mielenz TJ, DiMaggio CJ, Betz ME, DiGuiseppi C, Jones VC, et al. Driving Cessation and Health Outcomes in Older Adults. Journal of the American Geriatrics Society. 2016;64(2):332-41.
5. Owsley C, Ball K, Sloane ME, Roenker DL, Bruni JR. Visual/cognitive correlates of vehicle accidents in older drivers. Psychol Aging. 1991;6(3):403-15.
6. Wood JM, Black AA. Ocular disease and driving. Clinical & experimental optometry. 2016;99(5):395-401.
7. Bohensky M, Charlton J, Odell M, Keeffe J. Implications of vision testing for older driver licensing. Traffic injury prevention. 2008;9(4):304-13.
8. Yan MK, Kumar H, Kerr N, Medeiros FA, Sandhu SS, Crowston J, et al. Transnational review of visual standards for driving: How Australia compares with the rest of the world. Clinical & experimental ophthalmology. 2019;47(7):847-63.
9. Burton MJ, Ramke J, Marques AP, Bourne RRA, Congdon N, Jones I, et al. The Lancet Global Health Commission on Global Eye Health: vision beyond 2020. The Lancet Global health. 2021;9(4):e489-e551.
10. Shamseer L, Moher D, Clarke M, Ghersi D, Liberati A, Petticrew M, et al. Preferred reporting items for systematic review and meta-analysis protocols (PRISMA-P) 2015: elaboration and explanation. BMJ (Clinical research ed). 2015;350:g7647.
11. Nguyen H, Di Tanna GL, Coxon K, Brown J, Ren K, Ramke J, et al. Associations between vision impairment and driving and the effectiveness of vision-related interventions: protocol for a systematic review and meta-analysis. BMJ open. 2020;10(11):e040881.
12. University of Adelaide. Critical Appraisal Tools N/A [Available from: https://joannabriggs.org/ebp/critical_appraisal_tools.
13. Campbell M, McKenzie JE, Sowden A, Katikireddi SV, Brennan SE, Ellis S, et al. Synthesis without meta-analysis (SWiM) in systematic reviews: reporting guideline. BMJ (Clinical research ed). 2020;368:l6890.
14. Stroup DF, Berlin JA, Morton SC, Olkin I, Williamson GD, Rennie D, et al. Meta-analysis of observational studies in epidemiology: a proposal for reporting. Meta-analysis Of Observational Studies in Epidemiology (MOOSE) group. Jama. 2000;283(15):2008-12.
15. Abebe Y, Wondmikun Y. Defective color perception among car drivers in Addis Ababa, Ethiopia. Traffic Injury Prevention. 2002;3(4):294-7.
16. Abraham EG, Ezepue UF, Umeh RE, Ekanem U. Causes of visual impairment among commercial motor vehicle drivers in Uyo metropolis, Akwa Ibom State, Nigeria. Orient Journal of Medicine. 2010;22(1/4):unpaginated.
17. Adekoya BJ, Owoeye JFA, Adepoju FG, Ajaiyeoba AI. Visual function survey of commercial intercity vehicle drivers in Ilorin, Nigeria. Canadian journal of ophthalmology Journal canadien d'ophtalmologie. 2009;44(3):261-4.
18. Baker JM, Drews-Botsch C, Pfeiffer MR, Curry AE. Driver licensing and motor vehicle crash rates among young adults with amblyopia and unilateral vision impairment. Journal of AAPOS : the official publication of the American Association for Pediatric Ophthalmology and Strabismus. 2019;23(4):230-2.
19. Ball K, Owsley C, Sloane ME, Roenker DL, Bruni JR. Visual attention problems as a predictor of vehicle crashes in older drivers. Investigative ophthalmology & visual science. 1993;34(11):3110-23.

For peer review only - http://bmjopen.bmj.com/site/about/guidelines.xhtml

20. Bekibele CO, Fawole OI, Bamgboye AE, Adekunle LV, Ajayi R, Baiyeroju AM. Prevalence of refractive error and attitude to spectacle use among drivers of public institutions in Ibadan, Nigeria. Annals of African medicine. 2007;6(1):26-30.
21. Biza M, Mossie A, Woldemichael K, Gelaw Y. Visual impairment and road traffic accidents among drivers in Jimma Town, Southwest Ethiopia. Ethiopian medical journal. 2013;51(2):123-32.
22. Boadi-Kusi SB, Kyei S, Asare FA, Owusu-Ansah A, Awuah A, Darko-Takyi C. Visual function among commercial vehicle drivers in the central region of Ghana. Journal of optometry. 2016;9(1):54-63.
23. Cohen Y, Zadok D, Barkana Y, Shochat Z, Ashkenazi I, Avni I, et al. Relationship between night myopia and night-time motor vehicle accidents. Acta ophthalmologica Scandinavica. 2007;85(4):367-70.
24. Cross JM, McGwin G, Jr., Rubin GS, Ball KK, West SK, Roenker DL, et al. Visual and medical risk factors for motor vehicle collision involvement among older drivers. The British journal of ophthalmology. 2009;93(3):400-4.
25. Emerole CG, Nneli RO. Visual indices of motor vehicle drivers in relation to road safety in Nigeria. Nigerian journal of physiological sciences : official publication of the Physiological Society of Nigeria. 2013;28(1):57-62.
26. Fishman GA, Anderson RJ, Stinson L, Haque A. Driving performance of retinitis pigmentosa patients. The British journal of ophthalmology. 1981;65(2):122-6.
27. Green KA, McGwin G, Jr., Owsley C. Associations between visual, hearing, and dual sensory impairments and history of motor vehicle collision involvement of older drivers. Journal of the American Geriatrics Society. 2013;61(2):252-7.
28. Gresset J, Meyer F. Risk of automobile accidents among elderly drivers with impairments or chronic diseases. Canadian journal of public health = Revue canadienne de sante publique. 1994;85(4):282-5.
29. Gresset JA, Meyer FM. Risk of accidents among elderly car drivers with visual acuity equal to 6/12 or 6/15 and lack of binocular vision. Ophthalmic & physiological optics : the journal of the British College of Ophthalmic Opticians (Optometrists). 1994;14(1):33-7.
30. Haymes SA, Leblanc RP, Nicolela MT, Chiasson LA, Chauhan BC. Risk of falls and motor vehicle collisions in glaucoma. Investigative ophthalmology & visual science. 2007;48(3):1149-55.
31. Huisingh C, Levitan EB, Irvin MR, MacLennan P, Wadley V, Owsley C. Visual Sensory and Visual-Cognitive Function and Rate of Crash and Near-Crash Involvement Among Older Drivers Using Naturalistic Driving Data. Investigative ophthalmology & visual science. 2017;58(7):2959-67.
32. Huisingh C, McGwin G, Wood J, Owsley C. The driving visual field and a history of motor vehicle collision involvement in older drivers: A population- based examination. Investigative Ophthalmology and Visual Science. 2015;56(1):132-8.
33. Humphriss D. Three South African studies on the relation between road accidents and drivers' vision. Ophthalmic & physiological optics : the journal of the British College of Ophthalmic Opticians (Optometrists). 1987;7(1):73-9.
34. Isawumi MA, Adeoti CO, Ubah JN, Oluwatimilehin IO, Raji RA. Ocular status of commercial drivers in Osun State, Nigeria. African journal of medicine and medical sciences. 2011;40(4):405-11.
35. Ivers RQ, Mitchell P, Cumming RG. Sensory impairment and driving: the Blue Mountains Eye Study. American journal of public health. 1999;89(1):85-7.
36. Keeffe JE, Jin CF, Weih LM, McCarty CA, Taylor HR. Vision impairment and older drivers: who's driving? The British journal of ophthalmology. 2002;86(10):1118-21.
37. Kristalovich L, Ben Mortenson W. Visual Field Impairment and Driver Fitness: A 1-Year Review of Crashes and Traffic Violations. The American journal of occupational therapy : official publication of the American Occupational Therapy Association. 2019;73(5):7305345010p1-p6.
38. Kwon M, Huisingh C, Rhodes LA, McGwin G, Jr., Wood JM, Owsley C. Association between Glaucoma and At-fault Motor Vehicle Collision Involvement among Older Drivers: A Population-based Study. Ophthalmology. 2016;123(1):109-16.

For peer review only - http://bmjopen.bmj.com/site/about/guidelines.xhtml

39. Maag U, Vanasse C, Dionne G, Laberge-Nadeau C. Taxi drivers' accidents: how binocular vision problems are related to their rate and severity in terms of the number of victims. Accident; analysis and prevention. 1997;29(2):217-24.
40. Margolis KL, Kerani RP, McGovern P, Songer T, Cauley JA, Ensrud KE. Risk factors for motor vehicle crashes in older women. Journals of Gerontology - Series A Biological Sciences and Medical Sciences. 2002;57(3):M186-M91.
41. McCloskey LW, Koepsell TD, Wolf ME, Buchner DM. Motor vehicle collision injuries and sensory impairments of older drivers. Age and ageing. 1994;23(4):267-73.
42. McGwin G, Jr., Huisingh C, Jain SG, Girkin CA, Owsley C. Binocular visual field impairment in glaucoma and at-fault motor vehicle collisions. Journal of glaucoma. 2015;24(2):138-43.
43. McGwin G, Jr., Mays A, Joiner W, Decarlo DK, McNeal S, Owsley C. Is glaucoma associated with motor vehicle collision involvement and driving avoidance? Investigative ophthalmology & visual science. 2004;45(11):3934-9.
44. McGwin G, Jr., Mitchell B, Searcey K, Albert MA, Feist R, Mason JO, 3rd, et al. Examining the association between age-related macular degeneration and motor vehicle collision involvement: a retrospective cohort study. The British journal of ophthalmology. 2013;97(9):1173-6.
45. McGwin G, Jr., Sims RV, Pulley L, Roseman JM. Relations among chronic medical conditions, medications, and automobile crashes in the elderly: a population-based case-control study. American journal of epidemiology. 2000;152(5):424-31.
46. McGwin G, Jr., Xie A, Mays A, Joiner W, DeCarlo DK, Hall TA, et al. Visual field defects and the risk of motor vehicle collisions among patients with glaucoma. Investigative ophthalmology & visual science. 2005;46(12):4437-41.
47. Meuleners LB, Brameld K, Fraser ML, Chow K. The impact of first- and second-eye cataract surgery on motor vehicle crashes and associated costs. Age and ageing. 2019;48(1):128-33.
48. Meuleners LB, Hendrie D, Lee AH, Ng JQ, Morlet N. The effectiveness of cataract surgery in reducing motor vehicle crashes: a whole population study using linked data. Ophthalmic epidemiology. 2012;19(1):23-8.
49. Meuleners LB, Ng JQ, Fraser M, Hendrie D, Morlet N. Impact of gender on first eye cataract surgery and motor vehicle crash risk for older drivers. Clinical & experimental ophthalmology. 2012;40(6):591-6.
50. Ogbonnaya CE, Uro-Chukwu HC, Sunday-Adeoye I. Ocular health status and visual fitness of commercial drivers in Abakaliki Metropolis, Nigeria. East African Medical Journal. 2018;95(7):1758-66.
51. Oladehinde MK, Adeoye AO, Adegbehingbe BO, Onakoya AO. Visual functions of commercial drivers in relation to road accidents in Nigeria. Indian journal of occupational and environmental medicine. 2007;11(2):71-5.
52. Ono T, Yuki K, Asaoka R, Kouyama K, Abe T, Tanabe S, et al. Glaucomatous visual field defect severity and the prevalence of motor vehicle collisions in Japanese: a hospital/clinic-based cross-sectional study. Journal of ophthalmology. 2015;2015(101524199):497067.
53. Ovenseri-Ogomo G, Adofo M. Poor vision, refractive errors and barriers to treatment among commercial vehicle drivers in the Cape Coast municipality. African health sciences. 2011;11(1):97-102.
54. Owsley C, McGwin G, Jr., Ball K. Vision impairment, eye disease, and injurious motor vehicle crashes in the elderly. Ophthalmic epidemiology. 1998;5(2):101-13.
55. Owsley C, McGwin G, Jr., Sloane M, Wells J, Stalvey BT, Gauthreaux S. Impact of cataract surgery on motor vehicle crash involvement by older adults. JAMA. 2002;288(7):841-9.
56. Owsley C, Stalvey B, Wells J, Sloane ME. Older drivers and cataract: driving habits and crash risk. The journals of gerontology Series A, Biological sciences and medical sciences. 1999;54(4):M203-11.

For peer review only - http://bmjopen.bmj.com/site/about/guidelines.xhtml

57. Owsley C, Stalvey BT, Wells J, Sloane ME, McGwin G, Jr. Visual risk factors for crash involvement in older drivers with cataract. Archives of ophthalmology (Chicago, Ill : 1960). 2001;119(6):881-7.
58. Owsley C, Swain T, Liu R, McGwin G, Jr., Kwon MY. Association of Photopic and Mesopic Contrast Sensitivity in older drivers with risk of motor vehicle collision using naturalistic driving data. BMC ophthalmology. 2020;20(1):47.
59. Pepple G, Adio A. Visual function of drivers and its relationship to road traffic accidents in Urban Africa. SpringerPlus. 2014;3(101597967):47.
60. Rahi JS, Cumberland PM, Peckham CS. Does amblyopia affect educational, health, and social outcomes? Findings from 1958 British birth cohort. BMJ (Clinical research ed). 2006;332(7545):820-5.
61. Runge JW. Medical conditions and driver crash risk: Do license restrictions affect public safety? Annals of Emergency Medicine. 2000;36(2):164-5.
62. Schlenker MB, Thiruchelvam D, Redelmeier DA. Association of Cataract Surgery With Traffic Crashes. JAMA ophthalmology. 2018;136(9):998-1007.
63. Sims RV, Owsley C, Allman RM, Ball K, Smoot TM. A preliminary assessment of the medical and functional factors associated with vehicle crashes by older adults. Journal of the American Geriatrics Society. 1998;46(5):556-61.
64. Szlyk JP, Pizzimenti CE, Fishman GA, Kelsch R, Wetzel LC, Kagan S, et al. A comparison of driving in older subjects with and without age-related macular degeneration. Archives of ophthalmology (Chicago, Ill : 1960). 1995;113(8):1033-40.
65. Tanabe S, Yuki K, Ozeki N, Shiba D, Abe T, Kouyama K, et al. The association between primary open-angle glaucoma and motor vehicle collisions. Investigative ophthalmology & visual science. 2011;52(7):4177-81.
66. Wood JM, Black AA, Mallon K, Kwan AS, Owsley C. Effects of Age-Related Macular Degeneration on Driving Performance. Investigative ophthalmology & visual science. 2018;59(1):273-9.
67. Wood JM, Black AA, Mallon K, Thomas R, Owsley C. Glaucoma and Driving: On-Road Driving Characteristics. PloS one. 2016;11(7):e0158318.
68. Yuki K, Asaoka R, Tsubota K. The relationship between central visual field damage and motor vehicle collisions in primary open-angle glaucoma patients. PloS one. 2014;9(12):e115572.
69. Yuki K, Awano-Tanabe S, Ono T, Shiba D, Murata H, Asaoka R, et al. Risk Factors for Motor Vehicle Collisions in Patients with Primary Open-Angle Glaucoma: A Multicenter Prospective Cohort Study. PloS one. 2016;11(11):e0166943.
70. Adler G, Bauer MJ, Rottunda S, Kuskowski M. Driving habits and patterns in older men with glaucoma. Social work in health care. 2005;40(3):75-87.
71. Deshmukh AV, Murthy GJ, Reddy A, Murthy PR, Kattige J, Murthy VR. Older Drivers and Glaucoma in India: Driving Habits and Crash Risks. Journal of glaucoma. 2019;28(10):896-900.
72. Naredo Turrado J, Orriols L, Contrand B, Zins M, Salmi LR, Lafont S, et al. Chronic medical conditions and their association with crash risk and changes in driving habits: a prospective study of the GAZEL cohort. Injury prevention : journal of the International Society for Child and Adolescent Injury Prevention. 2020((Lafont) Universite Lyon, IFSTTAR, Universite Lyon 1, Bron, France).
73. Rubin GS, Ng ESW, Bandeen-Roche K, Keyl PM, Freeman EE, West SK. A prospective, population-based study of the role of visual impairment in motor vehicle crashes among older drivers: the SEE study. Investigative ophthalmology & visual science. 2007;48(4):1483-91.
74. Ahmed M, Patnaik JL, Whitestone N, Hossain MA, Alauddin M, Husain L, et al. Visual Impairment and Risk of Self-Reported Road Traffic Crashes Among Bus Drivers in Bangladesh. Asia-Pacific journal of ophthalmology (Philadelphia, Pa). 2022;11(1):72-8.
75. Alvarez-Peregrina C, Martinez-Perez C, Villa-Collar C, Sánchez-Tena M. Influence of Vision on Drivers: A Pilot Study. International journal of environmental research and public health. 2021;18(22).

For peer review only - http://bmjopen.bmj.com/site/about/guidelines.xhtml

76. Crizzle AM, Toxopeus R, Alphonsus K. Medical Conditions and Crash Risk in Commercial Motor Vehicle Drivers. Journal of occupational and environmental medicine. 2020;62(8):628-33.
77. Piyasena P, Olvera-Herrera VO, Chan VF, Clarke M, Wright DM, MacKenzie G, et al. Vision impairment and traffic safety outcomes in low-income and middle-income countries: a systematic review and meta-analysis. The Lancet Global health. 2021;9(10):e1411-e22.
78. Swain TA, McGwin G, Jr., Wood JM, Antin JF, Owsley C. Naturalistic Driving Techniques and Association of Visual Risk Factors With At-Fault Crashes and Near Crashes by Older Drivers With Vision Impairment. JAMA ophthalmology. 2021;139(6):639-45.
79. Swain TA, McGwin G, Jr., Wood JM, Owsley C. Motion perception as a risk factor for motor vehicle collision involvement in drivers ≥ 70 years. Accid Anal Prev. 2021;151:105956.
80. Vofo BN, Ayuk DA, Pe'er J, Chichom-Mefire A, Tendongfor N, Nche EN. Visual assessment of commercial drivers in the South West Region of Cameroon. BMC Ophthalmol. 2021;21(1):148.
81. Wedenoja J, Kalsi J, Salenius S, Parkkari K, Kaarniranta K, Tervo T. The role of vision-related problems in fatal road accidents in Finland. Acta Ophthalmol. 2021;99(4):427-30.
82. Anstey KJ, Windsor TD, Luszcz MA, Andrews GR. Predicting driving cessation over 5 years in older adults: Psychological well-being and cognitive competence are stronger predictors than physical health. Journal of the American Geriatrics Society. 2006;54(1):121-6.
83. Bressler NM, Chang TS, Varma R, Suner I, Lee P, Dolan CM, et al. Driving ability reported by neovascular age-related macular degeneration patients after treatment with ranibizumab. Ophthalmology. 2013;120(1):160-8.
84. Bressler NM, Varma R, Mitchell P, Suner IJ, Dolan C, Ward J, et al. Effect of Ranibizumab on the Decision to Drive and Vision Function Relevant to Driving in Patients With Diabetic Macular Edema: Report From RESTORE, RIDE, and RISE Trials. JAMA ophthalmology. 2016;134(2):160-6.
85. Campbell MK, Bush TL, Hale WE. Medical conditions associated with driving cessation in community- dwelling, ambulatory elders. Journals of Gerontology. 1993;48(4):S230-S4.
86. DeCarlo DK, Scilley K, Wells J, Owsley C. Driving habits and health-related quality of life in patients with age-related maculopathy. Optometry and vision science : official publication of the American Academy of Optometry. 2003;80(3):207-13.
87. Edwards JD, Ross LA, Ackerman ML, Small BJ, Ball KK, Bradley S, et al. Longitudinal predictors of driving cessation among older adults from the ACTIVE clinical trial. The journals of gerontology Series B, Psychological sciences and social sciences. 2008;63(1):P6-12.
88. Freeman EE, Munoz B, Turano KA, West SK. Measures of visual function and time to driving cessation in older adults. Optometry and vision science : official publication of the American Academy of Optometry. 2005;82(8):765-73.
89. Gallo JJ, Rebok GW, Lesikar SE. The driving habits of adults aged 60 years and older. Journal of the American Geriatrics Society. 1999;47(3):335-41.
90. Garre-Olmo J, Pujol XP, Garriga OT, Lopez-Pousa S, Vilalta-Franch J. Biopsychosocial differences between drivers and non-drivers over the age of 74. Archives of gerontology and geriatrics. 2009;49(3):355-9.
91. Gilhotra JS, Mitchell P, Ivers R, Cumming RG. Impaired vision and other factors associated with driving cessation in the elderly: the Blue Mountains Eye Study. Clinical & experimental ophthalmology. 2001;29(3):104-7.
92. Goh YW, Ang GS, Azuara-Blanco A. Lifetime visual prognosis of patients with glaucoma. Clinical & experimental ophthalmology. 2011;39(8):766-70.
93. Hajek A, Brettschneider C, Eisele M, van den Bussche H, Wiese B, Mamone S, et al. Prevalence and determinants of driving habits in the oldest old: Results of the multicenter prospective AgeCoDe-AgeQualiDe study. Archives of gerontology and geriatrics. 2019;82(8214379, 7ax):245-50.
94. Huisingh C, McGwin G, Jr., Owsley C. Association of visual sensory function and higher-order visual processing skills with incident driving cessation. Clinical & experimental optometry. 2016;99(5):441-8.

For peer review only - http://bmjopen.bmj.com/site/about/guidelines.xhtml

95. Janz NK, Musch DC, Gillespie BW, Wren PA, Niziol LM, Collaborative Initial Glaucoma Treatment Study I. Evaluating clinical change and visual function concerns in drivers and nondrivers with glaucoma. Investigative ophthalmology & visual science. 2009;50(4):1718-25.
96. Keay L, Munoz B, Turano KA, Hassan SE, Munro CA, Duncan DD, et al. Visual and cognitive deficits predict stopping or restricting driving: the Salisbury Eye Evaluation Driving Study (SEEDS). Investigative ophthalmology & visual science. 2009;50(1):107-13.
97. Keay L, Palagyi A, Do V, White A, Lamoureux E, Ivers RQ, et al. Vision and driving status of older Australians with cataract: an investigation of public hospital waiting lists. Clinical & experimental optometry. 2016;99(5):449-55.
98. Levecq L, De Potter P, Jamart J. Visual acuity and factors influencing automobile driving status in 1,000 patients age 60 and older. Graefe's archive for clinical and experimental ophthalmology = Albrecht von Graefes Archiv fur klinische und experimentelle Ophthalmologie. 2013;251(3):881-7.
99. MacLeod KE, Satariano WA, Ragland DR. The Impact of Health Problems on Driving Status among Older Adults. Journal of transport & health. 2014;1(2):86-94.
100. Marottoli RA, Ostfeld AM, Merrill SS, Perlman GD, Foley DJ, Cooney LM, Jr. Driving cessation and changes in mileage driven among elderly individuals. Journal of gerontology. 1993;48(5):S255-60.
101. Monestam E, Lundquist B, Wachtmeister L. Visual function and car driving: longitudinal results 5 years after cataract surgery in a population. The British journal of ophthalmology. 2005;89(4):459-63.
102. Monestam E, Wachtmeister L. Impact of cataract surgery on car driving: a population based study in Sweden. The British journal of ophthalmology. 1997;81(1):16-22.
103. Ramulu PY, West SK, Munoz B, Jampel HD, Friedman DS. Driving cessation and driving limitation in glaucoma: the Salisbury Eye Evaluation Project. Ophthalmology. 2009;116(10):1846-53.
104. Ross LA, Anstey KJ, Kiely KM, Windsor TD, Byles JE, Luszcz MA, et al. Older drivers in Australia: trends in driving status and cognitive and visual impairment. Journal of the American Geriatrics Society. 2009;57(10):1868-73.
105. Segal-Gidan F, Varma R, Salazar X, Mack WJ. Factors influencing driving status in an older Latino population. Journal of aging and health. 2010;22(3):332-47.
106. Sengupta S, van Landingham SW, Solomon SD, Do DV, Friedman DS, Ramulu PY. Driving habits in older patients with central vision loss. Ophthalmology. 2014;121(3):727-32.
107. Stafford WR. Traditional antiglaucoma therapy: A clinical survey of side effects. Current Therapeutic Research - Clinical and Experimental. 1981;29(2):265-74.
108. Stewart RB, Moore MT, Marks RG, May FE, Hale WE. Driving cessation in the elderly: An analysis of symptoms, diseases, and medications. Journal of Geriatric Drug Therapy. 1993;8(2):45-60.
109. Takahashi A, Yuki K, Awano-Tanabe S, Ono T, Shiba D, Tsubota K. Association between glaucoma severity and driving cessation in subjects with primary open-angle glaucoma. BMC ophthalmology. 2018;18(1):122.
110. Tam ALC, Trope GE, Buys YM, Yang Y, Shen C, Jin Y-P. Self-perceived Impact of Glaucomatous Visual Field Loss and Visual Disabilities on Driving Difficulty and Cessation. Journal of glaucoma. 2018;27(11):981-6.
111. van Landingham SW, Hochberg C, Massof RW, Chan E, Friedman DS, Ramulu PY. Driving patterns in older adults with glaucoma. BMC ophthalmology. 2013;13(100967802):4.
112. Zebardast N, Swenor BK, van Landingham SW, Massof RW, Munoz B, West SK, et al. Comparing the Impact of Refractive and Nonrefractive Vision Loss on Functioning and Disability: The Salisbury Eye Evaluation. Ophthalmology. 2015;122(6):1102-10.
113. Kaleem MA, Rajjoub R, Schiefer C, Wall J, Applegate C, Tian J, et al. Characteristics of Glaucoma Patients Attending a Vision Rehabilitation Service. Ophthalmology Glaucoma. 2021;4(6):638-45.

For peer review only

For peer review only - http://bmjopen.bmj.com/site/about/guidelines.xhtml

114. Moon S, Park K. The Predictors of Driving Cessation among Older Drivers in Korea. International journal of environmental research and public health. 2020;17(19).
115. Robinson JL, Liu Y, Chen Q. Licensure and Driving Status among Visually Impaired Persons. Optom Vis Sci. 2021;98(12):1334-9.
116. Grundler W, Strasburger H. Visual attention outperforms visual-perceptual parameters required by law as an indicator of on-road driving performance. PloS one. 2020;15(8):e0236147.
117. Subzwari S, Desapriya E, Scime G, Babul S, Jivani K, Pike I. Effectiveness of cataract surgery in reducing driving-related difficulties: a systematic review and meta-analysis. Injury prevention : journal of the International Society for Child and Adolescent Injury Prevention. 2008;14(5):324-8.
118. Fraser ML, Meuleners LB, Lee AH, Ng JQ, Morlet N. Which visual measures affect change in driving difficulty after first eye cataract surgery? Accident; analysis and prevention. 2013;58:10-4.
119. Singletary BA, Do AN, Donnelly JP, Huisingh C, Mefford MT, Modi R, et al. Self-reported vs state-recorded motor vehicle collisions among older community dwelling individuals. Accident; analysis and prevention. 2017;101:22-7.
120. Haddon W, Jr. Energy damage and the ten countermeasure strategies. J Trauma. 1973;13(4):321-31.
121. Nguyen H, Luca Di Tanna G, Coxon K, Brown J, Ren K, Ramke J, et al. Associations between vision impairment and driving performance and the effectiveness of vision-related interventions: A systematic review. Transportation Research Interdisciplinary Perspectives. 2023;17:100753.
122. Strogatz D, Mielenz TJ, Johnson AK, Baker IR, Robinson M, Mebust SP, et al. Importance of Driving and Potential Impact of Driving Cessation for Rural and Urban Older Adults. The Journal of rural health : official journal of the American Rural Health Association and the National Rural Health Care Association. 2020;36(1):88-93.
123. Unsworth CA, Wells Y, Browning C, Thomas SA, Kendig H. To continue, modify or relinquish driving: findings from a longitudinal study of healthy ageing. Gerontology. 2007;53(6):423-31.
124. Bhalla K, Mohan D, O'Neill B. How much would low- and middle-income countries benefit from addressing the key risk factors of road traffic injuries? International journal of injury control and safety promotion. 2020;27(1):83-90.
125. Forjuoh SN. Traffic-related injury prevention interventions for low-income countries. Injury control and safety promotion. 2003;10(1-2):109-18.
126. Staton C, Vissoci J, Gong E, Toomey N, Wafula R, Abdelgadir J, et al. Road Traffic Injury Prevention Initiatives: A Systematic Review and Metasummary of Effectiveness in Low and Middle Income Countries. PloS one. 2016;11(1):e0144971.
127. Europe UNECf. Road Safety for All: United Nations; 2020 [Available from: https://unece.org/transport/publications/road-safety-all-0.
128. Bergen G, West BA, Luo F, Bird DC, Freund K, Fortinsky RH, et al. How do older adult drivers self-regulate? Characteristics of self-regulation classes defined by latent class analysis. Journal of safety research. 2017;61:205-10.
129. Braitman KA, McCartt AT. Characteristics of older drivers who self-limit their driving. Annals of advances in automotive medicine Association for the Advancement of Automotive Medicine Annual Scientific Conference. 2008;52:245-54.
130. Harmon AC, Babulal G, Vivoda JM, Zikmund-Fisher BJ, Carr DB. Planning for a Nondriving Future: Behaviors and Beliefs Among Middle-Aged and Older Drivers. Geriatrics (Basel, Switzerland). 2018;3(2).
131. Owsley C, McGwin G, Jr., Phillips JM, McNeal SF, Stalvey BT. Impact of an educational program on the safety of high-risk, visually impaired, older drivers. American journal of preventive medicine. 2004;26(3):222-9.
132. Ross LA, Clay OJ, Edwards JD, Ball KK, Wadley VG, Vance DE, et al. Do older drivers at-risk for crashes modify their driving over time? The journals of gerontology Series B, Psychological sciences and social sciences. 2009;64(2):163-70.

For peer review only - http://bmjopen.bmj.com/site/about/guidelines.xhtml

133. Zhang CT, Bowers AR, Savage SW. The Effects of Age, Distraction, and Simulated Central Vision Impairment on Hazard Detection in a Driving Simulator. Optometry and vision science : official publication of the American Academy of Optometry. 2020;97(4):239-48.
134. Cole BL. Protan colour vision deficiency and road accidents. Clinical & experimental optometry. 2002;85(4):246-53.
135. Cole BL. Colour blindness and driving. Clinical & experimental optometry. 2016;99(5):484-7.
136. Atchison DA, Pedersen CA, Dain SJ, Wood JM. Traffic signal color recognition is a problem for both protan and deutan color-vision deficients. Human factors. 2003;45(3):495-503.
137. Tagarelli A, Piro A, Tagarelli G, Lantieri PB, Risso D, Olivieri RL. Colour blindness in everyday life and car driving. Acta ophthalmologica Scandinavica. 2004;82(4):436-42.
138. Dhibi M. Road safety determinants in low and middle income countries. International journal of injury control and safety promotion. 2019;26(1):99-107.

FIGURE LEGENDS

Figure 1 Flow chart of search with papers reporting on MVC and driving cessation

Figure 2 Network diagram illustrating strength of association of vision impairment on motor vehicle crashes (MVC) and driving cessation found by narrative summaries

For peer review only

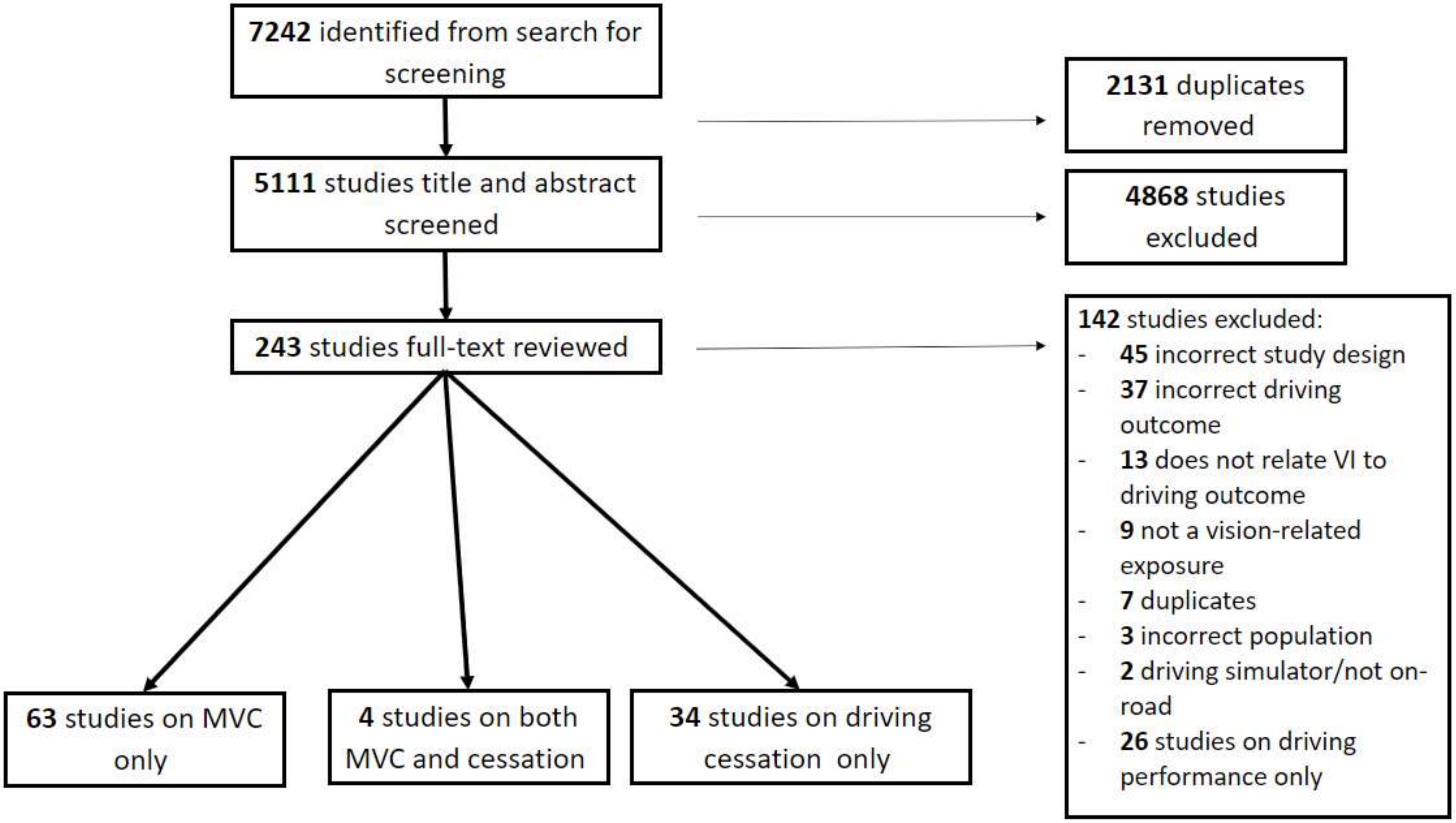

Figure 1 Flow chart of search with papers reporting on MVC and driving cessation

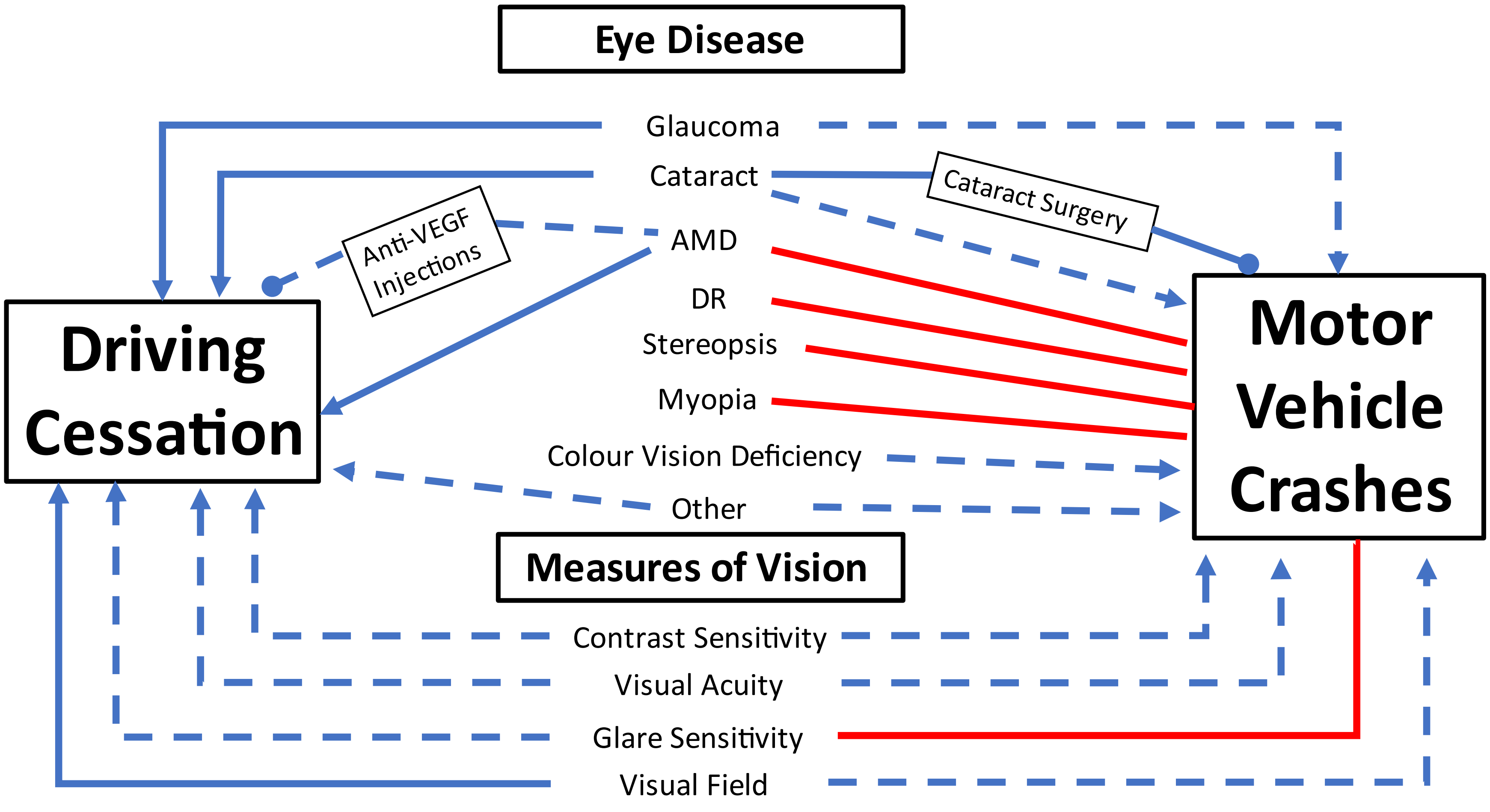

Figure 2 Network diagram illustrating strength of associations of vision impairment on motor vehicle crashes (MVC) and driving cessation found by narrative summaries

Consistent associations of an increased risk of the driving outcome = solid blue line with an arrowhead; Inconsistent associations of either an increased risk or no risk of the driving outcome = dashed blue line with an arrowhead; Consistent associations of a decreased risk of the driving outcome = solid blue line with a closed circle; Inconsistent associations of a decreased risk or no change in risk of the driving outcome = dashed blue line with a closed circle; No associations found with the driving outcome = solid red line

PRISMA 2020 Checklist

Section and Topic	Item #	Checklist item	Location where item is reported
**TITLE**			
Title	1	Identify the report as a systematic review.	pg.1
**ABSTRACT**			
Abstract	2	See the PRISMA 2020 for Abstracts checklist.	Appendix 1
**INTRODUCTION**			
Rationale	3	Describe the rationale for the review in the context of existing knowledge.	pg.4
Objectives	4	Provide an explicit statement of the objective(s) or question(s) the review addresses.	pg.4
**METHODS**			
Eligibility criteria	5	Specify the inclusion and exclusion criteria for the review and how studies were grouped for the syntheses.	pg.4,5, Table 1
Information sources	6	Specify all databases, registers, websites, organisations, reference lists and other sources searched or consulted to identify studies. Specify the date when each source was last searched or consulted.	pg.4
Search strategy	7	Present the full search strategies for all databases, registers and websites, including any filters and limits used.	Appendix 2
Selection process	8	Specify the methods used to decide whether a study met the inclusion criteria of the review, including how many reviewers screened each record and each report retrieved, whether they worked independently, and if applicable, details of automation tools used in the process.	pg.5
Data collection process	9	Specify the methods used to collect data from reports, including how many reviewers collected data from each report, whether they worked independently, any processes for obtaining or confirming data from study investigators, and if applicable, details of automation tools used in the process.	pg.5
Data items	10a	List and define all outcomes for which data were sought. Specify whether all results that were compatible with each outcome domain in each study were sought (e.g. for all measures, time points, analyses), and if not, the methods used to decide which results to collect.	pg.5
	10b	List and define all other variables for which data were sought (e.g. participant and intervention characteristics, funding sources). Describe any assumptions made about any missing or unclear information.	pg.5
Study risk of bias assessment	11	Specify the methods used to assess risk of bias in the included studies, including details of the tool(s) used, how many reviewers assessed each study and whether they worked independently, and if applicable, details of automation tools used in the process.	pg.5,6
Effect measures	12	Specify for each outcome the effect measure(s) (e.g. risk ratio, mean difference) used in the synthesis or presentation of results.	pg.6
Synthesis methods	13a	Describe the processes used to decide which studies were eligible for each synthesis (e.g. tabulating the study intervention characteristics and comparing against the planned groups for each synthesis (item #5)).	pg.6
	13b	Describe any methods required to prepare the data for presentation or synthesis, such as handling of missing summary statistics, or data conversions.	pg.6
	13c	Describe any methods used to tabulate or visually display results of individual studies and syntheses.	pg.6
	13d	Describe any methods used to synthesize results and provide a rationale for the choice(s). If meta-analysis was performed, describe the model(s), method(s) to identify the presence and extent of statistical heterogeneity, and software package(s) used.	pg.6
	13e	Describe any methods used to explore possible causes of heterogeneity among study results (e.g. subgroup analysis, meta-regression).	pg.6
	13f	Describe any sensitivity analyses conducted to assess robustness of the synthesized results.	pg.6
Reporting bias assessment	14	Describe any methods used to assess risk of bias due to missing results in a synthesis (arising from reporting biases).	N/A
Certainty	15	Describe any methods used to assess certainty (or confidence) in the body of evidence for an outcome.	N/A

For peer review only - http://bmjopen.bmj.com/site/about/guidelines.xhtml

PRISMA 2020 Checklist

Section and Topic	Item #	Checklist item	Location where item is reported
assessment			
**RESULTS**			
Study selection	16a	Describe the results of the search and selection process, from the number of records identified in the search to the number of studies included in the review, ideally using a flow diagram.	pg.6, Figure 1, Table 2a and 2b
	16b	Cite studies that might appear to meet the inclusion criteria, but which were excluded, and explain why they were excluded.	Figure 1
Study characteristics	17	Cite each included study and present its characteristics.	pg.8-11, Appendix 4a and 4b
Risk of bias in studies	18	Present assessments of risk of bias for each included study.	Appendix 3
Results of individual studies	19	For all outcomes, present, for each study: (a) summary statistics for each group (where appropriate) and (b) an effect estimate and its precision (e.g. confidence/credible interval), ideally using structured tables or plots.	pg.8-11, Appendix 4a and 4b
Results of syntheses	20a	For each synthesis, briefly summarise the characteristics and risk of bias among contributing studies.	pg.8-11, Appendix 4a and 4b
	20b	Present results of all statistical syntheses conducted. If meta-analysis was done, present for each the summary estimate and its precision (e.g. confidence/credible interval) and measures of statistical heterogeneity. If comparing groups, describe the direction of the effect.	pg.8-11, Appendix 4a and 4b
	20c	Present results of all investigations of possible causes of heterogeneity among study results.	pg.8-11, Figure 2a-b
	20d	Present results of all sensitivity analyses conducted to assess the robustness of the synthesized results.	N/A
Reporting biases	21	Present assessments of risk of bias due to missing results (arising from reporting biases) for each synthesis assessed.	N/A
Certainty of evidence	22	Present assessments of certainty (or confidence) in the body of evidence for each outcome assessed.	N/A
**DISCUSSION**			
Discussion	23a	Provide a general interpretation of the results in the context of other evidence.	pg.11-13
	23b	Discuss any limitations of the evidence included in the review.	pg.11-13
	23c	Discuss any limitations of the review processes used.	pg.12,13
	23d	Discuss implications of the results for practice, policy, and future research.	pg.11-13
**OTHER INFORMATION**			
Registration and protocol	24a	Provide registration information for the review, including register name and registration number, or state that the review was not registered.	pg.2
	24b	Indicate where the review protocol can be accessed, or state that a protocol was not prepared.	pg.4
	24c	Describe and explain any amendments to information provided at registration or in the protocol.	N/A
Support	25	Describe sources of financial or non-financial support for the review, and the role of the funders or sponsors in the review.	pg.14
Competing	26	Declare any competing interests of review authors.	pg.14

For peer review only

PRISMA 2020 Checklist

Section and Topic	Item #	Checklist item	Location where item is reported
interests			
Availability of data, code and other materials	27	Report which of the following are publicly available and where they can be found: template data collection forms; data extracted from included studies; data used for all analyses; analytic code; any other materials used in the review.	pg.13

PRISMA ABSTRACT CHECKLIST

Section and Topic	Item #	Checklist item	Reported (Yes/No)
**TITLE**			
Title	1	Identify the report as a systematic review.	Yes
**BACKGROUND**			
Objectives	2	Provide an explicit statement of the main objective(s) or question(s) the review addresses.	Yes
**METHODS**			
Eligibility criteria	3	Specify the inclusion and exclusion criteria for the review.	Yes
Information sources	4	Specify the information sources (e.g. databases, registers) used to identify studies and the date when each was last searched.	Yes
Risk of bias	5	Specify the methods used to assess risk of bias in the included studies.	Yes
Synthesis of results	6	Specify the methods used to present and synthesise results.	Yes
**RESULTS**			
Included studies	7	Give the total number of included studies and participants and summarise relevant characteristics of studies.	Yes
Synthesis of results	8	Present results for main outcomes, preferably indicating the number of included studies and participants for each. If meta-analysis was done, report the summary estimate and confidence/credible interval. If comparing groups, indicate the direction of the effect (i.e. which group is favoured).	Yes
**DISCUSSION**			
Limitations of evidence	9	Provide a brief summary of the limitations of the evidence included in the review (e.g. study risk of bias, inconsistency and imprecision).	Yes
Interpretation	10	Provide a general interpretation of the results and important implications.	Yes
**OTHER**			
Funding	11	Specify the primary source of funding for the review.	Yes
Registration	12	Provide the register name and registration number.	Yes

For peer review only

PRISMA 2020 Checklist

For peer review only

Appendix 2 Complete search strategy for review. While search terms were included for driving performance, driving scores and errors, the studies with outcome measures of driving performance was outside of the scope of this current manuscript and are reported elsewhere.

MEDLINE (OVID) search strategy

1. exp Eye Diseases/
2. exp Cataract Extraction/
3. Lens Implantation, Intraocular/
4. Lenses, Intraocular/
5. cataract$.tw.
6. ((intraocular or intra ocular) adj3 lens$).tw.
7. (IOL or IOLs).tw.
8. Vision Tests/
9. Visual Acuity/
10. exp Refractive Errors/
11. Visual Fields/
12. Visual Field Tests/
13. Contrast Sensitivity/
14. Depth Perception/
15. (visual adj2 (acuit$ or field$)).tw.
16. contrast sensitivity.tw.
17. (depth perception or stereopsis).tw.
18. ((impair$ or decreas$ or declin$) adj3 (vision or visual$ or sight$)).tw.
19. (improv$ adj3 (vision or visual$ or sight$)).tw.
20. ((visual or vision) adj2 function$).tw.
21. exp Vision, Ocular/
22. Vision Screening/
23. or/1-22
24. Mass Screening/
25. ((eye$ or sight or vision or visual$) adj2 (test$ or screen$ or exam$ or diagnos$ or assess$)).tw
26. 24 and 25
27. 23 or 26
28. exp Motor Vehicles/
29. exp Automobile Driving/
30. Accidents, Traffic/
31. (driver$ or driving).tw.
32. (automobile$ or car or cars or vehicle$).tw.
33. (motoring or motorcar or "motor car" or "motor cars").tw.
34. crash$.tw.
35. ((road or traffic) adj2 injur$).tw.
36. ((road or traffic or motor) adj2 (accident$ or incident$)).tw.
37. ((road or traffic or motor) adj2 collision$).tw.
38. or/28-37
39. epidemiologic studies/ or case-control studies/ or cohort studies/ or observational study/ or follow-up studies/ or longitudinal studies/ or prospective studies/ or retrospective studies/ or controlled before-after studies/ or cross-sectional studies/ or historically controlled study/ or interrupted time series analysis/
40. epidemiologic methods/ or focus groups/ or interviews as topic/ or exp "surveys and questionnaires"/

For peer review only

41. epidemiologic research design/ or control groups/ or cross-over studies/ or double-blind method/ or meta-analysis as topic/ or network meta-analysis/ or random allocation/ or single-blind method/
42. epidemiologic methods/ or clinical trials as topic/ or feasibility studies/ or multicenter studies as topic/ or pilot projects/ or sampling studies/ or twin studies as topic/
43. randomized controlled trial/ or controlled clinical trials as topic/ or randomized controlled trials as topic/
44. comparative study/ or evaluation studies/ or meta-analysis/ or review/ or multicenter study/ or "systematic review"/ or validation studies/
45. health surveys/
46. outcome assessment, health care/
47. risk factors/
48. self report/
49. (population or cohort or observation$ or intervention$ or prospective or retrospective or comparative).tw.
50. (questionnaire$ or survey$).tw.
51. (randomized or randomised or randomly or RCT).tw.
52. (systematic review or meta-analysis).tw.
53. (before adj2 after).tw.
54. (case$ adj2 control$).tw.
55. (cross adj1 section$).tw.
56. or/39-55
57. 27 and 38
58. 56 and 57
59. vehicle-controlled.tw.
60. (vehicle adj3 inject$).tw.
61. 59 or 60
62. 58 not 61
63. (animal$ or mouse or mice$ or dog or canine or rat or rats or primate$).ti.
64. (dry eye or cell$ or mutation$ or genes or genome or sequencing).ti.
65. or/63-64
66. 62 not 65
67. limit 66 to english language
68. exp case reports/
69. (case adj2 report$).tw.
70. 68 or 69
71. 67 not 70
72. limit 71 to (editorial or letter)
73. 71 not 72

EMBASE Search Strategy

1. exp eye disease/
2. exp cataract extraction/
3. lens implantation/
4. lens implant/
5. cataract$.tw.
6. ((intraocular or intra ocular) adj3 lens$).tw.
7. (IOL or IOLs).tw.
8. vision test/
9. visual acuity/

For peer review only

10. refractive error/
11. visual field/
12. perimetry/
13. contrast sensitivity/
14. depth perception/
15. (visual adj2 (acuit$ or field$)).tw.
16. contrast sensitivity.tw.
17. (depth perception or stereopsis).tw.
18. ((impair$ or decreas$ or declin$) adj3 (vision or visual$ or sight$)).tw.
19. (improv$ adj3 (vision or visual$ or sight$)).tw.
20. ((visual or vision) adj2 function$).tw.
21. vision/
22. or/1-21
23. mass screening/
24. ((eye$ or sight or vision or visual$) adj2 (test$ or screen$ or exam$ or diagnos$ or assess$)).tw.
25. 23 and 24
26. 22 or 25
27. exp car driving/
28. exp motor vehicle/
29. traffic accident/
30. (driver$ or driving).tw.
31. (automobile$ or car or cars or vehicle$).tw.
32. (motoring or motorcar or "motor car" or "motor cars").tw.
33. crash$.tw.
34. ((road or traffic) adj2 injur$).tw.
35. ((road or traffic or motor) adj2 (accident$ or incident$)).tw.
36. ((road or traffic or motor) adj2 collision$).tw.
37. or/27-36
38. study design/
39. controlled clinical trial/
40. case control study/
41. cohort analysis/
42. observational study/
43. follow up/
44. longitudinal study/
45. prospective study/
46. retrospective study/
47. epidemiology/
48. cross-sectional study/
49. control group/
50. crossover procedure/
51. "meta analysis (topic)"/
52. network meta-analysis/
53. randomization/
54. single blind procedure/
55. double blind procedure/
56. "clinical trial (topic)"/
57. "controlled clinical trial (topic)"/
58. "randomized controlled trial (topic)"/
59. "multicenter study (topic)"/
60. feasibility study/

For peer review only

61. pilot study/
62. comparative study/
63. evaluation study/
64. multicenter study/
65. randomized controlled trial/
66. meta analysis/
67. "systematic review"/
68. validation study/
69. interview/
70. questionnaire/
71. outcome assessment/
72. "systematic review (topic)"/
73. health survey/
74. risk factor/
75. self report/
76. evidence based practice/
77. (population or cohort or observation$ or intervention$ or prospective or retrospective or comparative).tw.
78. (questionnaire$ or survey$).tw.
79. (randomized or randomised or randomly or RCT).tw.
80. (systematic review or meta-analysis).tw.
81. (before adj2 after).tw.
82. (case$ adj2 control$).tw.
83. (cross adj1 section$).tw.
84. or/38-83
85. 26 and 37
86. 84 and 85
87. vehicle-controlled.tw.
88. (vehicle adj3 inject$).tw.
89. or/87-88
90. 86 not 89
91. (animal$ or mouse or mice$ or dog or canine or rat or rats or primate$).ti.
92. (dry eye or cell$ or mutation$ or genes or genome or sequencing).ti.
93. or/91-92
94. 90 not 93
95. limit 94 to conference abstract status
96. 94 not 95
97. limit 96 to english language
98. exp case report/
99. (case adj2 report$).tw.
100. or/98-99
101. 97 not 100
102. limit 101 to (conference paper or "conference review" or editorial or letter or note)
103. 101 not 102

GLOBAL HEALTH Search Strategy

1. exp eye diseases/
2. exp vision disorders/
3. cataract$.tw.
4. ((intraocular or intra ocular) adj3 lens$).tw.
5. (IOL or IOLs).tw.

For peer review only

6. (visual adj2 (acuit$ or field$)).tw.
7. contrast sensitivity.tw.
8. (depth perception or stereopsis).tw.
9. ((impair$ or decreas$ or declin$) adj3 (vision or visual$ or sight$)).tw.
10. (improv$ adj3 (vision or visual$ or sight$)).tw.
11. ((visual or vision) adj2 function$).tw.
12. ((eye$ or sight or vision or visual$) adj2 (test$ or screen$ or exam$ or diagnos$ or assess$)).tw.
13. or/1-12
14. drivers/
15. vehicles/
16. motor cars/
17. traffic/
18. traffic accidents/
19. (driver$ or driving).tw.
20. (automobile$ or car or cars or vehicle$).tw.
21. (motoring or motorcar or "motor car" or "motor cars").tw.
22. crash$.tw.
23. ((road or traffic) adj2 injur$).tw.
24. ((road or traffic or motor) adj2 (accident$ or incident$)).tw.
25. ((road or traffic or motor) adj2 collision$).tw.
26. or/14-25
27. cohort studies/
28. case-control studies/
29. longitudinal studies/
30. retrospective studies/
31. epidemiology/
32. exp clinical trials/
33. randomized controlled trials/
34. feasibility studies/
35. pilot projects/
36. meta-analysis/
37. systematic reviews/
38. reviews/
39. questionnaires/
40. surveys/
41. epidemiological surveys/
42. risk factors/
43. (population or cohort or observation$ or intervention$ or prospective or retrospective or comparative).tw.
44. (questionnaire$ or survey$).tw.
45. (randomized or randomised or randomly or RCT).tw.
46. (systematic review or meta-analysis).tw.
47. (before adj2 after).tw.
48. (case$ adj2 control$).tw.
49. (cross adj1 section$).tw.
50. or/27-49
51. 13 and 26
52. 50 and 51
53. (animal$ or mouse or mice$ or dog or canine or rat or rats or primate$).ti.
54. (dry eye or cell$ or mutation$ or genes or genome or sequencing).ti.
55. 53 or 54

For peer review only

56. 52 not 55
57. limit 56 to english language
58. case reports/
59. (case adj2 report$).tw.
60. 58 or 59
61. 57 not 60
62. limit 61 to (conference or conference paper or conference proceedings or correspondence or editorial or thesis)
63. 61 not 62

For peer review only

Appendix 3 Risk of Bias Assessment for all Included Studies

Analytical Cross-Sectional Study

**Citation**	**Q1 (SB)**	**Q2 (SB)**	**Q3 (DB)**	**Q4 (C)**	**Q5 (C)**	**Q6(C)**	**Q7 (DB)**	**Q8 (V)**	**Risk of Bias***
**High Income Countries**									
Adler G, et al. 2005.	Y	Y	N	Y	Y	Y	Y	Y	L
Alvarez-Peregrina C et al., 2021	N	N	N	N	N	N	Y	N	H
Ball K, et al. 1993.	Y	N	U	U	N	N	U	Y	H
Cohen Y, et al. 2007.	Y	Y	Y	Y	N	N	Y	Y	M
Crizzle AM et al., 2020	Y	Y	U	U	N	N	Y	Y	M
Cross JM, et al. 2009.	U	U	Y	N	N	U	Y	Y	H
DeCarlo DK, et al. 2003.	Y	Y	N	U	U	U	Y	Y	M
Edwards JD, et al. 2008.	Y	Y	Y	N	Y	Y	Y	Y	L
Garre-Olmo J, et al. 2009.	Y	Y	Y	U	Y	Y	Y	Y	M
Gilhotra JS, et al. 2001.	Y	Y	Y	Y	Y	Y	Y	Y	L
Hajek A, et al. 2019.	Y	Y	N	N	Y	Y	Y	Y	M

For peer review only - http://bmjopen.bmj.com/site/about/guidelines.xhtml

Huisingh C, et al. 2015.	Y	Y	Y	Y	Y	Y	Y	Y	L
Ivers RQ, et al. 1999.	N	N	Y	N	Y	Y	N	Y	M
Kaleem MA et al., 2021	Y	Y	Y	Y	N	N	Y	N	M
Keay L, et al. 2016.	Y	Y	Y	Y	Y	Y	Y	Y	L
Kwon M, et al. 2016.	Y	Y	Y	Y	Y	Y	Y	Y	L
Levecq L, et al. 2013.	Y	Y	Y	Y	N	N	Y	Y	M
MacLeod KE, et al. 2014.	Y	Y	Y	Y	Y	Y	Y	Y	L
Marottoli RA, et al. 1993.	Y	Y	N	Y	U	Y	Y	Y	M
Moon SH & Park K et al., 2020	Y	Y	N	N	N	N	Y	Y	M
Ono T, et al. 2015.	Y	Y	Y	Y	Y	Y	N	Y	L
Owsley C, et al. 2001.	Y	Y	Y	Y	Y	Y	Y	Y	L
Owsley C, et al. 2020.	Y	Y	Y	Y	Y	Y	Y	Y	L
Ramulu PY, et al. 2009.	Y	Y	Y	Y	Y	Y	Y	Y	L

For peer review only

Robinson JL et al., 2021	Y	Y	Y	Y	N	N	Y	N	M
Ross LA, et al. 2009.	Y	Y	Y	Y	N	N	Y	Y	M
Runge JW. 2000.	N	N	U	U	U	U	Y	Y	H
Segal-Gidan F, et al. 2010.	Y	Y	Y	Y	Y	Y	Y	Y	L
Sengupta S, et al. 2014.	Y	Y	Y	Y	Y	Y	Y	Y	L
Stafford WR. 1981.	N	N	Y	U	N	N	Y	Y	H
Stewart RB, et al. 1993.	Y	Y	N	N	Y	Y	Y	Y	M
Swain TA et al., 2021	Y	Y	Y	Y	Y	Y	Y	Y	L
Tam ALC, et al. 2018.	Y	Y	Y	Y	N	N/A	Y	Y	M
Tanabe S, et al. 2011.	Y	Y	Y	Y	Y	Y	N	Y	L
van Landingham SW, et al. 2013.	Y	Y	Y	Y	U	U	Y	Y	M
Wedenoja J et al., 2021	Y	Y	U	U	N	N	Y	N	H
Yuki K, et al. 2014.	Y	Y	Y	Y	N	N	N	Y	M

For peer review only - http://bmjopen.bmj.com/site/about/guidelines.xhtml

Zebardast N, et al. 2015.	Y	Y	Y	Y	Y	Y	Y	Y	L
**Low Middle Income Countries**									
Abebe Y, et al. 2002.	Y	Y	Y	N	N	N	N	Y	M
Abraham EG, et al. 2010.	Y	N	N	N	N	N	N	Y	H
Adekoya BJ, et al. 2009.	Y	Y	Y	Y	N	N	N	Y	M
Ahmed M et al., 2021	N	N	Y	Y	Y	Y	Y	Y	M
Bekibele CO, et al. 2007.	N	Y	Y	Y	N	N	N	Y	M
Biza M, et al. 2013.	Y	Y	Y	Y	N	N	N	Y	M
Boadi-Kusi SB, et al. 2016.	Y	Y	N	Y	N	N	N	U	H
Emerole CG, et al. 2013.	N	N	Y	Y	N	N	N	N	H
Humphriss D. 1987.	Y	N	N	N	N	N/A	U	U	H
Isawumi MA, et al. 2011.	Y	Y	U	Y	N	N	N	U	H
Ogbonnaya CE, et al. 2018.	Y	Y	Y	Y	N	N	N	Y	M

For peer review only

Oladehinde MK, et al. 2007.	N	N	Y	Y	N	N	N	Y	H
Ovenseri-Ogomo G, et al. 2011.	Y	Y	Y	Y	N	N/A	N	Y	M
Pepple G, et al. 2014.	Y	Y	Y	N	N	N	N	Y	M
Vofo BN et al., 2021	Y	Y	Y	Y	N	N	Y	N	M

*Risk of bias scores: high (1-3), medium (4-6), and low (7-8)

SB= selection bias, DB= detection bias, C= confounding, V= validity

Case Control Study

**Citation**	**Q1 (SB)**	**Q2 (SB)**	**Q3 (SB)**	**Q4 (DB)**	**Q5 (PB)**	**Q6 (C)**	**Q7 (C)**	**Q8 (PB)**	**Q9 (V)**	**Q10 (V)**	**Risk of Bias***
**High Income Countries**											
Campbell MK, et al. 1993.	Y	U	Y	N	U	Y	Y	Y	U	U	M
Gallo JJ, et al. 1999.	Y	N	Y	U	U	Y	Y	Y	N	Y	M
Gresset JA, et al. 1994.	N	Y	Y	Y	Y	Y	Y	Y	Y	Y	L
Gresset JA, et al. 1994.	U	Y	Y	U	U	N	N	Y	Y	Y	M
McCloskey LW, et al. 1994.	Y	Y	Y	Y	Y	U	U	Y	Y	Y	M

For peer review only

McGwin G, et al. 2000.	Y	Y	Y	N	Y	Y	Y	Y	Y	Y	L
McGwin G, et al. 2004.	Y	U	Y	Y	Y	Y	Y	Y	Y	Y	L
McGwin G, et al. 2005.	Y	Y	Y	Y	Y	Y	Y	Y	Y	Y	L
Owsley C, et al. 1998.	Y	Y	Y	Y	Y	N	N	Y	Y	Y	M
Sims RV, et al. 1998.	Y	U	Y	Y	Y	N	N	N	Y	Y	M
Szlyk JP, et al. 1995.	Y	Y	Y	Y	Y	N	N	Y	Y	Y	M
Wood JM, et al. 2018.	Y	Y	N	Y	Y	Y	Y	Y	U	Y	M
Wood JM, et al. 2016.	Y	Y	Y	Y	Y	Y	Y	Y	U	Y	L
Owsley C, et al. 1999.	Y	Y	Y	Y	Y	Y	Y	Y	Y	Y	L
**Low Middle Income Countries**											
Deshmukh AV, et al. 2019.	Y	N	Y	Y	Y	N	N	Y	U	N	M

*Risk of bias scores: high (1-4), medium (5-8), and low (9-10)

SB= selection bias, DB= detection bias, PB = performance bias, C= confounding, V= validity

For peer review only - http://bmjopen.bmj.com/site/about/guidelines.xhtml

Case Series

Citation	Q1 (SB)	Q2 (DB)	Q3 (DB)	Q4 (SB)	Q5 (SB)	Q6 (SB)	Q7 (SB)	Q8 (AtB)	Q9 (SB)	Q10 (V)	Risk of Bias*
**High Income Countries**											
Goh YW, et al. 2011	Y	Y	Y	Y	Y	Y	Y	Y	N	Y	L

*Risk of bias scores: high (1-4), medium (5-8), and low (9-10)
SB= selection bias, DB= detection bias, AtB= attrition bias, V= validity

Cohort Study

Citation	Q1 (SB)	Q2 (PB)	Q3 (DB)	Q4 (C)	Q5 (C)	Q6 (SB)	Q7 (DB)	Q8 (V)	Q9 (AtB)	Q10 (AtB)	Q11 (V)	Risk of Bias*
**High Income Countries**												
Anstey KJ, et al. 2006.	Y	Y	Y	Y	Y	Y	Y	Y	Y	Y	Y	L
Baker JM, et al. 2019.	N	Y	N	Y	Y	Y	Y	Y	Y	N/A	Y	M
Fishman GA et al., 1981	Y	U	Y	Y	Y	N	N	U	N	N	N	H
Freeman EE, et al. 2005.	Y	Y	Y	Y	Y	Y	Y	Y	Y	N	Y	L
Green KA, et al. 2013.	Y	Y	Y	Y	Y	U	Y	Y	Y	N	Y	M

For peer review only

For peer review only - http://bmjopen.bmj.com/site/about/guidelines.xhtml

Haymes SA, et al. 2007.	Y	Y	Y	Y	Y	N/A	Y	Y	Y	N/A	Y	M
Huisingh C, et al. 2017.	Y	Y	Y	Y	Y	Y	Y	Y	Y	U	Y	L
Huisingh C, et al. 2016.	Y	Y	Y	Y	Y	Y	Y	Y	N	N	Y	M
Janz NK, et al. 2009.	Y	Y	Y	Y	Y	U	Y	Y	N	N	Y	M
Keay L, et al. 2009.	Y	Y	Y	Y	Y	Y	Y	Y	Y	Y	Y	L
Keeffe JE, et al. 2002.	Y	Y	Y	N	N/A	Y	N	Y	Y	U	U	M
Kristalovich L, et al. 2019.	U	Y	Y	N	N	Y	U	Y	N/A	N/A	Y	M
Maag U, et al. 1997.	Y	N	U	Y	Y	N	Y	Y	Y	N/A	Y	M
Margolis KL, et al. 2002.	Y	Y	Y	Y	Y	Y	Y	Y	Y	U	Y	L
McGwin G, et al. 2015.	Y	Y	Y	Y	Y	Y	Y	Y	Y	U	Y	L
McGwin G, et al. 2013.	Y	Y	Y	N	N/A	Y	Y	Y	U	U	Y	M
Meuleners LB,et al. 2019.	Y	Y	Y	Y	Y	Y	Y	Y	U	Y	Y	L

For peer review only - http://bmjopen.bmj.com/site/about/guidelines.xhtml

Meuleners LB, et al. 2012.	Y	Y	Y	Y	Y	Y	Y	Y	Y	N/A	Y	L
Meuleners LB, et al. 2012.	Y	Y	Y	Y	Y	Y	Y	Y	U	U	Y	M
Monestam E, et al. 2005.	Y	Y	Y	N	N	Y	Y	Y	N	N	U	M
Monestam E, et al. 1997.	Y	U	Y	N	N	Y	Y	Y	Y	N	Y	M
Naredo Turrado J, et al. 2020.	Y	Y	Y	Y	Y	U	Y	Y	Y	U	Y	M
Owsley C, et al. 2002.	Y	Y	Y	Y	Y	Y	Y	Y	Y	U	Y	L
Rahi JS, et al. 2006.	Y	Y	U	Y	Y	Y	N	Y	N	N	Y	M
Rubin GS, et al. 2007.	Y	Y	Y	Y	Y	Y	Y	Y	Y	N	Y	L
Schlenker MB, et al. 2018.	Y	Y	Y	Y	Y	Y	Y	Y	Y	U	Y	L
Swain TA et al., 2021	Y	Y	Y	Y	Y	Y	Y	Y	Y	U	Y	L
Takahashi A, et al. 2018.	U	Y	Y	Y	Y	Y	Y	Y	N	N	Y	M
Yuki K, et al. 2016.	Y	Y	Y	N	N/A	Y	N	N	Y	U	N	M

*Risk of bias scores: high (1-4), medium (5-9), and low (10-11)

SB= selection bias, PB= performance bias, DB= detection bias, C= confounding, AtB= attrition bias, V= validity

For peer review only

Systematic Reviews

Citation	Q1 (SB)	Q2 (SB)	Q3 (SB)	Q4 (SB)	Q5 (InB)	Q6 (InB)	Q7 (InB)	Q8 (C)	Q9 (PubB)	Q10 (V)	Q11 (V)	Risk of Bias
**High Income Countries**												
Piyasena P et al., 2021	Y	Y	Y	Y	Y	U	Y	Y	Y	Y	Y	L

Risk of bias scores: high (1-4), medium (5-9), and low (10-11)

SB= selection bias, InB= information bias, C= confounding, PubB= publication bias, V= validity

Randomised Controlled Trials

Citation	Q1 (AlB)	Q2 (PB)	Q3 (SB)	Q4 (PB)	Q5 (PB)	Q6 (AlB)	Q7 (C)	Q8 (AtB)	Q9 (V)	Q10 (DB)	Q11 (DB)	Q12 (V)	Q13 (V)	Risk of Bias
**High Income Countries**														
Bressler NM, et al. 2013.	Y	Y	Y	Y	Y	U	Y	Y	Y	Y	Y	Y	Y	L
Bressler NM, et al. 2016.	Y	Y	Y	Y	Y	Y	Y	Y	Y	Y	Y	Y	Y	L

*Risk of bias scores: high (1-6), medium (7-11), and low (12-13)

AlB = allocation bias, SB= selection bias, PB= performance bias, C= confounding, DB= detection bias, AtB= attrition bias, V= validity

Appendix 4a Raw data tables and additional narrative summaries of papers on motor vehicle crashes

Table 4a(i) All studies (n=16) on glaucoma and Motor Vehicle Crashes (MVC). Of the 16 studies, 5 studies were suitable for meta-analysis on associations with any MVC involvement and 2 studies on associations with at-fault MVC involvement

Additional Narrative Summary:
Associations between glaucoma and MVCs were mixed in the studies identified. Even though seven controlled studies found glaucoma to increase the odds of any, injurious, and at-fault MVC involvement, two studies found crash involvement to halve in drivers with glaucoma. Only one study looked at not-at-fault crashes, but found no associations (OR 1 (95% CI 0.4-2.5)). Drivers with more severe glaucoma, irrespective of whether it was in the better or worse eye, were involved in more MVCs and also had greater odds of any crash and at-fault crash involvement compared to drivers without glaucoma and drivers with mild glaucoma.

**Author and Year**	**Study Design**	**Participants/ Sample Size**	**Mean Age**	**Country**	**VI Definition**	**Comparator**	**Outcome Measure (OR, RR, HR, etc?)**	**Effect Measure (with 95% CI) + any description of results (if appropriate**
**Included in Meta-analysis (any MVC involvement)**								
Cross JM et al., 2009	Cross-sectional	3158 (249/2909)	71.9	USA	Self-reported physician diagnosed	Drivers without glaucoma	RR (rate ratio)	**Any MVC**: 1.18 (0.81, 1.72)
Haymes S et al., 2007	Retrospective Cohort	95 (48/47)	69	Canada	Diagnosis from glaucoma specialist, glaucomatous optic disc damage and corresponding visual field damage	Drivers without glaucoma	OR (logistic regression)	**Any MVC**: 6.62 (1.4, 31.23)
Kwon M et al., 2016	Cross-sectional	1899 (206/1693)	age, no.: 70-79 years =	USA	Physician diagnosed	Drivers without glaucoma	RR (rate ratio)	**Any MVC involvement**: 1.65 (1.2, 2.28)

			1358, 80-89 years = 502, 90-98 years = 39					
Naredo Turrado J et al., 2020	Prospective Cohort	11670 (525/11145)	62.4	France	Self-reported physician diagnosed	Drivers without glaucoma	OR	**Any MVC**: 0.93 (0.72, 1.22)
McGwin G Jr et al., 2004	Case Control	691 (576/115)	69.2	USA	ICD-9 codes 365.1 and 265.3	Drivers without glaucoma	RR (relative risk)	**Any MVC**: 0.58 (0.4, 0.83)
**Included in Meta-analysis (at-fault MVC involvement)**								
Cross JM et al., 2009	Cross-sectional	3158 (249/2909)	71.9	USA	Self-reported physician diagnosed	Drivers without glaucoma	RR (rate ratio)	**At-fault MVC**: 0.91 (0.48, 1.72)
Haymes S et al., 2007	Retrospective Cohort	95 (48/47)	69	Canada	Diagnosis from glaucoma specialist, glaucomatous optic disc damage and corresponding visual field damage	Drivers without glaucoma	OR (logistic regression)	**At-fault MVC**: 12.44 (1.08, 143.99)
McGwin G Jr et al., 2004	Case Control	691 (576/115)	69.2	USA	ICD-9 codes 365.1 and 265.3	Drivers without glaucoma	RR (relative risk)	**At-fault MVC**: 0.99 (0.54, 1.8)
**Included in Narrative Summaries Only – High Income Countries**								
Adler G et al., 2004	Cross-sectional	199 (52/147)	71.3	USA	Open-or closed-angle glaucoma	Drivers without glaucoma	Prevalence (%)	25% (13/52) of drivers with glaucoma had been in an MVC compared to

								25.9% (38/147) of drivers without glaucoma (p= 0.86).
Cross JM et al., 2009	Cross-sectional	3158 (249/2909)	71.9	USA	Self-reported physician diagnosed	Drivers without glaucoma	RR (rate ratio)	**Injurious MVC**: 0.63 (0.19, 2.06)
Haymes S et al., 2007	Retrospective Cohort	95 (48/47)	69	Canada	Diagnosis from glaucoma specialist, glaucomatous optic disc damage and corresponding visual field damage	Drivers without glaucoma	OR (logistic regression)	**Any MVC (state-reported)**: 3.21 (0.72, 14.27)
								**At-fault MVC (state-reported)**: 7.21 (0.46, 113.4)
							Prevalence (%)	27% (11/400 of drivers with glaucoma had been involved in an MVC compared to 7% (3/44) in drivers without glaucoma.<br><br>20% (8/40) of drivers with glaucoma were at-fault in an MVC compared to 2% (1/44) in drivers without glaucoma.

For peer review only

Kwon M et al., 2016	Cross-sectional	1899 (206/1693)	age, no.: 70-79 years = 1358, 80-89 years = 502, 90-98 years = 39	USA	Physician diagnosed	Drivers without glaucoma	Prevalence (%)	18% (37/206) of drivers with glaucoma were at-fault in a crash compared to 13% (219/1693) of drivers without glaucoma.
McCloskey L et al., 1994	Case Control	683 (42/641)	age, no.: 65-69 years = 264, 70-74 years = 195, 75-79 years = 138, 80+ years = 86	USA	Physician diagnosed (hospital data)	Age-matched drivers with glaucoma who have not been injured in a police-reported MVC in the same calendar year as their matched case.	RR (relative risk)	**Injurious MVC**: 1.5 (0.8, 2.9)*
							Prevalence (%)	7.7% (18/234) of all drivers who had an injurious crash also had glaucoma.
McGwin G Jr et al., 2000	Case Control	901 (447/454)	N/A	USA	Self-reported physician diagnosed	Not-at-fault drivers involved in crashes, without glaucoma	OR	**Not at-fault MVC**: 1 (0.4, 2.5)
							Prevalence (%)	5.2% (10/198) of not-at-fault crashes involved drivers with

								glaucoma. 6.9% (17/249) 0f at-fault crashes involved drivers with glaucoma.
McGwin G Jr et al., 2004	Case Control	691 (576/115)	69.2	USA	ICD-9 codes 365.1 and 265.3	Drivers without glaucoma	RR (relative risk)	**All MVC per person-time**: 0.57 (0.39, 0.83)
								**At-fault MVC per person-time**: 1.02 (0.56, 1.87)
							Prevalence (%)	27% (153/576) of drivers with glaucoma were involved in an MVC compared to 37% (42/115) of drivers without glaucoma.<br><br>15% (87/576) of drivers with glaucoma were at-fault in a crash compared to 12% (14/115) of drivers without glaucoma.
McGwin G Jr et al., 2005	Case Control	240 (120/120)	72.9	USA	ICD-9 codes 365.1 and 265.3, given an AGIS score	Drivers with glaucoma who have not had	OR	**At-fault MVC**: 1.7(0.7, 3.7)

For peer review only

					from visual fields examinations – **mild defect in better eye**	an MVC between 1994 and 2000.		
					ICD-9 codes 365.1 and 265.3, given an AGIS score from visual fields examinations **– moderate defect in better eye**		OR	**At-fault MVC:** 2 (0.7, 5.4)
					ICD-9 codes 365.1 and 265.3, given an AGIS score from visual fields examinations – **severe defect in better eye**		OR	**At-fault MVC:** 4.2 (0.9, 15.3)
					ICD-9 codes 365.1 and 265.3, given an AGIS score from visual fields examinations – **mild defect in worse eye**		OR	**At-fault MVC:** 1.9 (0.6, 6.1)
					ICD-9 codes 365.1 and 265.3, given an AGIS score from visual fields examinations – **moderate defect in worse eye**		OR	**At-fault MVC:** 4.2 (1.2, 15)

For peer review only

					ICD-9 codes 365.1 and 265.3, given an AGIS score from visual fields examinations – **severe defect in worse eye**		OR	**At-fault MVC:** 9 (2.4, 33.2)
					ICD-9 codes 365.1 and 265.3, given an AGIS score from visual fields examination – **moderate bilateral defect**		OR	**Any MVC:** 3.6 (1.4, 9.4)
					ICD-9 codes 365.1 and 265.3, given an AGIS score from visual fields examination – **severe bilateral defect**		OR	**Any MVC:** 4.4 (1.6, 12.4)
Owsley C et al., 1998	Case Control	294 (179/155)	71	USA	Physician diagnosed	Drivers without glaucoma	OR	**Injurious MVC**: 3.6 (1.2, 10.9)*
								**At-fault MVC**: 1.5 (0.5, 4.8)*
							Prevalence (%)	14.1% (11/78) of all injurious crash drivers had glaucoma. 6.3% (6/101) of all non-injurious crash drivers had glaucoma.

For peer review only

Ono T et al., 2015	Cross-sectional	386 (199/187)	64.7	Japan	**Mild POAG in the worse eye** as a visual field defect corresponding to a mean deviation (MD) of −6 dB or better	Drivers without glaucoma	OR (logistic regression)	**Any MVC**: 1.07 (0.55, 2.1)*
					**Moderate POAG in the worse eye** as an MD between −6 and −12 dB		OR (logistic regression)	**Any MVC**: 1.44 (0.68, 3.08)*
					**Severe POAG in the worse eye** as an MD of −12 dB or worse		OR (logistic regression)	**Any MVC**: 2.28 (1.07, 4.88)*
					**Mild POAG in the better eye** as a visual field defect corresponding to a mean deviation (MD) of −6 dB or better		OR (logistic regression)	**Any MVC**: 1.36 (0.78, 2.37)*
					**Moderate POAG in the better eye** as an MD between −6 and −12 dB		OR (logistic regression)	**Any MVC**: 1.82 (0.65, 5.11)*
					**Severe POAG in the better eye** as an MD of −12 dB or worse		OR (logistic regression)	**Any MVC**: 1.65 (0.39, 6.87)*

					Physician diagnosis of POAG in any eye		Prevalence (%)	22.6% (45/199) pf drivers with glaucoma have been in an MVC compared to 16% (30/187) of drivers without glaucoma.
Tanabe S et al., 2011	Cross-sectional	265 (121/144)	61.6	Japan	Mild POAG as a visual field defect corresponding to a mean deviation (MD) of -5 dB or better in both eyes, moderate POAG as corresponding to an MD of -5 to -10 dB in the worse eye, severe POAG as an MD of -10 dB or worse in the worse eye	Drivers free of ocular disease	OR	**Any MVC (severe glaucoma)**: 9.9 (2.1, 47.8)
							Prevalence (%)	6% (7/121) of drivers with glaucoma have been involved in an MVC compared to 3.5% (5/144) of drivers without glaucoma.<br><br>When dividing by glaucoma severity, 3.9% (2/51) or moderate and 25% (5/20) of severe glaucoma drivers have been involved in a crash.

Wood J et al., 2016	Case Control	145 (75/70)	72.9	Australia	Visual acuity better than 20/40 with one or both eyes and binocular visual fields with a horizontal extent of at least 110° within 10° above and below the horizontal midline	Age-matched controls without glaucoma	Prevalence (%)	4% (3/75) of glaucoma drivers had an MVC in the past 12 months compared to 6% (4/70) of drivers without glaucoma; difference was not significant (p= 0.64)
								19% (14/75) of drivers with glaucoma had an MVC in the past 5 years compared to 23% (16/70) of drivers without glaucoma; difference was not significant (p=0.56)
Yuki K et al., 2014	Cross-sectional	247 (147/0)	63.7	Japan	Severity categorised using Mills Glaucoma Staging system – **better eye**	Drivers without history of MVC	Prevalence (%)	Amongst drivers with a history of MVCs, 11.8% (6/51), 72.5% (37/51), 9.8% (5/51), and 5.9% (3/51) had a better eye glaucoma

For peer review only

								severity score of 0, 1, 2, 3 or more, respectively. Amongst drivers without a history of MVC, this glaucoma score were: 20.4% (40/196), 65.8% (129/196), 9.2% (18/196), and 4.6% (9/196). The differences between proportion of people assigned these scores in the two MVC groups was not significant (p= 0.86).
					Severity categorised using Mills Glaucoma Staging system – **worse eye**			Amongst drivers with a history of MVCs, 2% (1/51), 47.1% (24/51), 23.5% (5/51) and 5.9% (3/51) had a worse eye glaucoma severity score of 0, 1, 2, 3 or more,

For peer review only

								respectively. Amongst drivers without a history of MVCs, the glaucoma scores were: 2.6% (5/196), 54.6% (107/196), 24.5% (48/196), and 18.3% (36/196), respectively. The differences between proportion of people assigned these scores in the two MVC groups was not significant (p= 0.86).
Yuki K et al., 2016	Prospective Cohort	191 (191/0)	63.7	Japan	Primary open angle glaucoma (POAG)	Drivers with POAG but no history of MVCs.	Prevalence (%)	15% (28/191) of drivers with glaucoma have been involved in an MVC. Of these, 64.3% (18/28) had mild, 14.3% (4/28) has moderate, and 22.4% (6/27) had severe glaucoma.
**Included in Narrative Summaries Only – Low Middle Income Countries**								

For peer review only

Deshmukh AV et al., 2019	Case Control	150 (100/50)	64.5	India	Diagnosed glaucomatous optic nerve head changes and corresponding visual field defects, which satisfied Anderson criterion	Aged-matched (older than 40 years) non-glaucoma controls	Prevalence (%)	12.9% (11/85) of drivers with glaucoma had an MVC in the past 12 months compared to 70% (35/50) of drivers without glaucoma. This significance was significant (p<0.001).

*unadjusted results

For peer review only

Table 4a(ii) All studies (n=8) on cataract and Motor Vehicle Crashes (MVC) with meta-analyses suitable for 2 studies on associations with any MVC involvement

Author and Year	Study Design	Total Participants (exposed/control)	Mean Age/Age Range	Country	VI Definition	Comparator	Outcome Measure (OR, RR, HR, etc.?)	Effect Measure (with 95% CI) + any description of results (if appropriate)
**Included in Meta-analysis (any MVC involvement)**								
Cross JM et al., 2009	Cross-sectional	3158 (1165/1993)	71.9	USA	Self-reported physician diagnosed	Drivers without cataract	RR (rate ratio)	**Any MVC**: 1.21 (0.95, 1.55)
Margolis KL et al., 2002	Prospective Cohort	1416 (370/1046)	71.3	USA	Self-reported physician diagnosed	Drivers without cataracts.	HR	**Any MVC:** 1.1 (0.88, 1.38)
**Included in Narrative Summaries Only – High Income Countries**								
Cross JM et al., 2009	Cross-sectional	3158 (1165/1993)	71.9	USA	Self-reported physician diagnosed	Drivers without cataract	RR (rate ratio)	**Injurious MVC**: 1.5 (0.85, 2.64)<br>**At-fault MVC**: 1.01 (0.69, 1.49)
McCloskey L et al., 1994	Case Control	683 (118/672)	age, no.: 65- 69 years = 264, 70-74 years = 195, 75-79 years = 138, 80+ years = 86	USA	Physician diagnosed (hospital data)	Age-matched drivers with cataracts who have not been injured in a police reported MVC in the same calendar year as their matched case.	RR (relative risk)<br>Prevalence (%)	**Injurious MVC**: 1 (0.7, 1.16)*<br>17.9% (42/234) of all injurious MVCs involved drivers with cataract.

For peer review

McGwin G Jr et al., 2000	Case Control	901 (447/454)	N/A	USA	Self-reported physician diagnosed	Not-at-fault drivers without cataract were involved in crashes	OR	**Not-at-fault MVC**: 1.1 (0.7, 1.8)
							Prevalence (%)	35.1% (69/198) of all not-at-fault crashes involved drives with cataract**.** 44.6% of all at-fault crashes involved drivers with cataract.
Naredo Turrado J et al., 2020	Prospective Cohort	11670 (525/11145)	62.4	France	Self-reported physician diagnosed	Drivers without cataract	OR	**Any MVC**: 1.27 (0.91, 1.76)*
Owsley C et al., 1998	Case Control	294 (179/155)	71	USA	Physician diagnosed	Drivers without cataract	OR	**Injurious MVC**: 1 (0.6, 1.8)*
								**Non-injurious MVC**: 1.1 (0.6, 1.8)*
							Prevalence (%)	47.4% (37/78) of injurious MVCs involved drivers with cataracts.
Owsley C et al., 1999	Case Control	384 (279/105)	69.9	USA	Cataract in one or both eyes from clinic notes with VA in one eye of 20/40 or worse and no previous	Drivers without cataract	RR (relative risk)	**At-fault MVC**: 2.46 (1, 6.16)
							X^2 (Chi Square)	The difference between the number of accidents between drivers with cataract and those

					cataract surgery in either eye			without cataract was non-significant (p= 0.19)
Owsley C et al., 2001	Cross-sectional	377 (274/103)	69.9	USA	Best-corrected VA of 20/40 or worse in worse eye eyes	Crash-free drivers	OR (logistic regression)	**Any MVC:** 1.26 (0.28, 5.59)
					Best-corrected VA of 20/40 or worse in better eyes			**Any MVC:** 1.39 (0.42, 4.62)

*unadjusted results

For peer review only

Table 4a(iii) All studies (n=3) on Age-Related Macular Degeneration (AMD) and Motor Vehicle Crashes (MVC) all suitable to only be summarised narratively

Author and Year	Study Design	Total Participants (exposed/ control)	Mean Age/ Age Range	Country	VI Definition	Comparator	Outcome Measure (OR, RR, HR etc.?)	Effect Measure (with 95% CI) + any description of results (if appropriate)
**Included in Narrative Summaries Only – High Income Countries**								
Cross JM et al., 2009	Cross-sectional	3158 (88/2070)	71.9	USA	Self-reported physician diagnosed	Participants without AMD	RR (rate ratio)	**Any MVC**: 0.57 (0.23, 1.39)
								**Injurious MVC**: 0.9 (0.11, 7.44)
								**At-fault MVC**: 0.95 (0.35, 2.56)
McCloskey L et al., 1994	Case Control	683 (25/658)	age, no.: 65-69 years = 264, 70-74 years = 195, 75-79 years = 138, 80+ years = 86	USA	Physician diagnosed (hospital data)	Age-matched drivers with AMD who had not been injured in a police-reported MVC in the same calendar year as their matched case.	RR (relative risk)	**Injurious MVC**: 0.9 (0.4, 2)*
							Prevalence (%)	3.8% (9/234) of drivers with AMD had a history of an MVC.
McGwin G Jr et al., 2013	Retrospective Cohort	205 (142/63)	72.7	USA	AREDS definition for early AMD	Participants without AMD	RR (rate ratio)	**Any MVC**: 0.48 (0.2, 1.18)* **Any MVC per 100 person-**

For peer review only

								**years**: 0.67 (0.32, 1.39)* **Any MVC per 1, 000, 000 person-miles**: 0.73 (0.36, 1.5)*
					AREDS definition for intermediate AMD			**Any MVC**: 0.22 (0.08, 0.64)* **Any MVC per 100 person-years**: 0.34 (0.13, 0.89)* **Any MVC per 1, 000, 000 person-miles**: 0.35 (0.13, 0.91)*
					AREDS definition for severe AMD			**Any MVC**: 0.46 (0.14, 1.54)* **Any MVC per 100 person-years**: 0.93 (0.31, 2.77)* **Any MVC per 1, 000, 000 person-miles**: 1.11 (0.38, 3.19)*
Szlyk et al., 1995	Case Control	21 (10/11)	73.2	USA	Physician diagnosed	Age-similar subjects with normal vision	X^2 (Chi Square)	X2= 4.68 (p<0.03); Age similar controls had more self-

For peer review only

								reported accidents than those with ARMD. The difference between the groups for the numbers of individuals involved in self-reported accidents was significant
						Younger control subjects with normal vision	X^2 (Chi Square)	X2= 8.06 (p=0.01); The number of self-reported accidents was significantly different between the younger control group and the ARMD group with the younger control group having more self-reported accidents.

For peer review only

Wood JM et al., 2018	Case Control	83 (33/50)	75.4	Australia	AREDS definition for late AMD	Aged-matched controls with no AMD	Prevalence with X^2 (Chi Square)	9% (3/33) of drivers with AMD had a crash in the past 12 months compared to 2% (1/50) of control drivers (p=0.28). 30% (10/33) of drivers with AMD had a history of 1 or more crashes in the past 5 years compared to 16% (8/50) of controls drivers (p=0.23).

*unadjusted results

For peer review only

Table 4a(iv) All studies (n=3) on diabetic retinopathy (DR) and Motor Vehicle Crashes (MVC) all suitable to only be summarised narratively

Author and Year	Study Design	Total Participants (Exposed/Control)	Mean Age	Country	VI Definition	Comparator	Outcome Measure (OR, RR, HR, etc.?)	Effect Measure (with 95% CI) + any description of results (if appropriate
**Included in Narrative Summaries Only – High Income Countries**								
Cross JM et al., 2009	Cross-sectional	3158 (98/3060)	71.9	USA	Self-reported physician diagnosed	Drivers without DR	RR (rate ratio)	**Any MVC**: 0.6 (0.26, 1.38)
								**Injurious MVC**: 0.95 (0.18, 4.92)
								**At-fault MVC**: 0.32 (0.08, 1.17)
McGwin G Jr et al., 2000	Case Control	901 (447/454)	N/A	USA	Self-reported physician diagnosed	Drivers without DR involved in not-at-fault crashes	OR	**Not at-fault MVC**: 1.9 (0.3, 10.9)
								**At-fault MVC**: 1.1 (0.3, 3.8)
							Prevalence (%)	1.1% (2/198) of not at-fault crash drivers had DR. 1.6% (3/249) of all at-fault crash drivers had DR.
Owsley C et al., 1998	Case Control	294 (179/155)	71	USA	Physician diagnosed	Drivers without DR	OR	**Non-injurious MVCs**:1 (0.1, 7.5)*
								**Injurious MVCs:** 0.7 (0.1, 8.2)*

*unadjusted results

For peer review only

Table 4a(v) All studies (n=5) on stereopsis impairment and Motor Vehicle Crashes (MVC) with meta-analysis suitable for 3 studies on associations with any MVC involvement

**Author and Year**	**Study Design**	**Participants/ Sample Size**	**Mean Age**	**Country**	**VI Definition**	**Comparator**	**Outcome Measure (OR, RR, HR?)**	**Effect Measure (with 95% CI) + any description of results (if appropriate)**
**Included in Meta-analysis (any MVC involvement)**								
Boadi-Kusi SB et al., 2016	Cross-sectional	520 (80/440)	39.2	Ghana	Physician diagnosed as abnormal.	Drivers with normal stereopsis	OR	**Any MVC:** 0.89 (0.44, 1.8)*
Margolis KL et al., 2002	Prospective Cohort	1416 (N/A)	71.3	USA	Physician diagnosed - distance depth perception per standard deviation change	Drivers with normal stereopsis.	HR	**Any MVC:** 1.01 (0.92, 1.11)
Oladehinde MK et al., 2007	Cross-sectional	215 (11/204)	41.5	Nigeria	Physician diagnosed - Visual acuity of 6/6 - 6/18 was normal, < 6/18 - 6/60 was classified as visual impairment and < 6/60 - 3/60 was classified as severe visual impairment while visual acuity less than 3/60 was classified as blindness.	Drivers with normal stereopsis	RR (risk ratio)	**Any MVC:** 1.45 (0.42, 5.3)*

**Included in Narrative Summaries Only – High Income Countries**								
Alvarez-Peregrina C et al., 2022	Cross-sectional	736 (55/681)	46.4	Spain	Physician diagnosed	Drivers with normal stereopsis.	X^2 (Chi Square)	Stereopsis was not linked with history of MVCs (p> 0.05).
**Included in Narrative Summaries Only – Low Middle Income Countries**								
Boadi-Kusi SB et al., 2016	Cross-sectional	520 (80/440)	39.2	Ghana	Physician diagnosed as abnormal.	Drivers with normal stereopsis	Prevalence (%)	25% (20/30) of drivers with abnormal stereopsis were involved in an MVC.
Humphriss D, 1987	Cross-sectional	366 (N/A)	N/A	South Africa	Visual acuity of at least 6/12 in each eye separately, or if one eye is below 6/12 then the second eye must be 616 or, wearing glasses and seeing binocularly the acuity must be 6/12. A lateral field of vision of 45 degrees is required.	Drivers with better stereopsis	Mean (SD)	Mean vision test score for stereopsis drivers without MVC involvement: 4.128<br><br>Mean vision test score for stereopsis drivers with MVC involvement: 5
Oladehinde MK et al., 2007	Cross-sectional	215 (11/204)	41.5	Nigeria	Physician diagnosed - Visual acuity of 6/6 - 6/18 was normal, < 6/18 - 6/60 was classified as visual	Drivers with normal stereopsis	Prevalence (%)	18.2% (2/11) of all drivers with abnormal stereopsis have been involved in an MVC

For peer review only

					impairment and < 6/60 - 3/60 was classified as severe visual impairment while visual acuity less than 3/60 was classified as blindness.			

*unadjusted results

For peer review only

Table 4a(vi) All studies (n=4) on myopia and Motor Vehicle Crashes (MVC), with 2 studies for meta-analysis

Author and Year	Study Design	Total Participants (exposed/control)	Mean Age	Country	VI Definition	Comparator	Outcome Measure (OR, RR, HR?)	Effect Measure (with 95% CI) + any description of results (if appropriate)
**Included in Meta-analysis (any MVC involvement)**								
Ahmed M et al., 2021	Cross-sectional	700 (62/638)	42.3	Bangladesh	Physician diagnosed	Drivers without myopia but with a history of MVCs	OR	**Any MVC:** 0.5 (0.15, 1.65)*
Boadi-Kusi SB et al., 2016	Cross-sectional	520 (10/510)	39.2	Ghana	Physician diagnosed – spherical power in the better eye of -0.50D or worse	Drivers without myopia but with a history of MVCs	OR	**Any MVC**: 0.99 (0.41, 2.4)*
**Included in Narrative Summaries Only – High Income Countries**								
Cohen Y et al., 2007	Cross-sectional	136 (34/102)	21	Israel	Night myopia: refraction in illumination and in total darkness in both eyes changed by 0.75 D or more	Drivers without night myopia	Fischer's Exact Test	No statistically significant difference in day time crashes between night myopia and normal subjects (p= 0.22).<br>Night myopia drivers had higher night-time crashes than non-night myopia drivers (p=0.044).

For peer review only

McCloskey L et al., 1994	Case Control	683 (235/448)	age, no.: 65- 69 years = 264, 70-74 years = 195, 75-79 years = 138, 80+ years = 86	USA	Physician diagnosed (hospital data)	Age-matched drivers with myopia who have not been injured in a police-reported MVC in the same calendar year as their matched case.	RR (relative risk)	**Injurious MVC**: 0.6 (0.1, 1)*

*unadjusted results

For peer review only

Table 4a(vii) All studies (n=8) on colour vision deficiency (CVD) and Motor Vehicle Crashes (MVC), all suitable to only be summarised narratively due to methodological limitations in non-standardised diagnosis of colour vision deficiencies

Additional Narrative Summary:
One study looking at the different types of colour deficiency found individuals with protan colour deficiency, measured by Hardy-Rand Rittler (HRR) pseudo-isochromatic plates, to report significantly more MVCs than those with deutan colour deficiency (p= 0.034).

Author and Year	Study Design	Total Participants (exposed/control)	Mean Age/Age Range	Country	VI Definition	Comparator	Outcome Measure (OR, RR, HR, etc.?)	Effect Measure (with 95% CI) + any description of results (if appropriate)
**Included in Narrative Summaries Only – High Income Countries**								
Piyasena P et al., 2021	Systematic review	15394 (254/15140)	39.3	N/A	Physician diagnosed	Drivers without colour deficiencies.	RR	**Any MVC:** 1.36 (1.01, 1.82)*
**Included in Narrative Summaries Only – Low Middle Income Countries**								
Abebe Y et al., 2002	Cross-sectional	1878 (85/1794)	33.5	Ethiopia	Physician diagnosed - Ishihara plates	Drivers without colour deficiencies.	OR	**Any MVC:** 1.94 (1.18, 3.17)
							Prevalence (%)	32% (27/85) of all drivers with colour blindness were involved in an MVC.
Biza M et al., 2013	Cross-sectional	249 (4/245)	33.6	Ethiopia	Physician diagnosed - Ishihara plates	Drivers without colour deficiencies.	OR	**Any MVC:** 2.34 (0.19, 28.58)
							Prevalence (%)	25% (1/4) of all drivers with colour blindness were involved in an MVC.
Boadi-Kusi SB et al., 2016	Cross-sectional	520 (37/483)	39.2	Ghana	Protan colour deficient – Hardy-Rand	Deutan colour deficient	Prevalence (%)	52.9% (9/17) of proton colour blindness drivers

For peer review only

					Rittler (HRR) pseudo-isochromatic plate			were involved in an MVC compared to 30.8% (4/13) of deutan colour blindness drivers; X2= 6.194 (p=0.034)
								35% (13/37) of all colour blind drivers were involved in an MVC.
							X^2 (Chi Square)	Protan colour blind drivers were more likely to report MVCs than deutan colour blind drivers: 6.194 (p= 0.034)
Isawumi MA et al., 2011	Cross-sectional	99 (6/93)	45.9	Nigeria	Physician diagnosed - Ishihara plates	Drivers with an MVC history without colour deficiencies.	X^2 (Chi Square)	X^2= 0.09, p=0.76 No significance between the number of MVC involvement in those with colour blindness and those without.
							Prevalence (%)	33% (2/6) of all drivers with colour blindness

For peer review only

								were involved in an MVC.
Oladehinde MK et al., 2007	Cross-sectional	215 (7/208)	41.5	Nigeria	Physician diagnosed - Ishihara plates	Drivers without a history of MVCs	RR (risk ratio)	**Any MVC:** 1.12 (10.3, 11.5)
							Prevalence (%)	2% (1/57) of all recorded MVCs involved colour blind drivers.
							X^2 (Chi Square)	There were no statistically significant associations between colour vision impairment and RTA: 2.3 (p= 0.1)
Ovenseri-Ogomo G et al., 2011	Cross-sectional	206 (7/199)	39.2	Ghana	VA < 6/18 in the better eye)	Drivers without a history of MVCs	X^2 (Chi Square)	No significance found for MVC involvement in drivers with colour blindness: X^2= 2.142, p=0.344
Pepple G et al., 2014	Cross-sectional	400 (262/138)	37.8	Nigeria	Physician diagnosed - Ishihara plates	Drivers without colour blindness.	RR (did not state test used)	**Any MVC**: 1.23 (p=0.4)*
							Prevalence (%)	56% (10/18) of drivers with colour blindness were involved in an MVC.

*unadjusted results

For peer review only

Table 4a(viii) All studies (n=28) on visual acuity (VA) impairment and Motor Vehicle Crashes (MVC) with meta-analysis suitable for 5 studies on any crash involvement and 2 on at-fault crashes

Additional Narrative Summary:
Results for injurious crashes were mixed; all non-significant. Similarly, there were no significant risks found for non-injurious and at-fault crash involvement, irrespective of worsening VA. One study looking at visual acuity in normal and low luminance also found poor acuity to not be a significant predictor of crash risk in both lighting conditions. A Japanese study, however, found the odds of crashing to increase by 20% in drivers with primary open angle glaucoma (POAG) experiencing worse eye declines of 0.01 LogMAR increments compared to those without VA changes.

**Author and Year**	**Study Design**	**Total Participants (exposed/control)**	**Mean Age**	**Country**	**VI Definition**	**Comparator**	**Outcome Measure (OR, RR, HR?)**	**Effect Measure (with 95% CI) + any description of results (if appropriate)**
**Included in Meta-analysis (any MVC involvement)**								
Green K et al., 2013	Retrospective Cohort	2000 (N/A)	Age, no.: 70-79 years = 1432, 80-89 years = 526, 90-99 years = 40	USA	VA worse than 20/40	Drivers with VA 20/40 or better	RR (rate ratio)	**Any MVC**: 1.04 (0.74, 1.48)
Huisingh C et a., 2017	Prospective Cohort	659 (35/624)	N/A	USA	Distance VA > 0.3logMAR	Drivers with VA 20/40 or better	RR (rate ratio)	**Any MVC:** 0.98 (0.52, 1.84)
Margolis KL et al., 2002	Prospective Cohort	1416 (N/A)	N/A	USA	20/40 or worse	Drivers with VA 20/40 or better	HR	**Any MVC:** 1.14 (0.73, 1.8)
Piyasena P et al., 2021	Systematic Review	15394 (710/14684)	39.3	N/A	Physician diagnosed	Drivers without a vision impairment	RR	**Any MVC:** 1.46 (1.2, 1.78)

For peer review only

Rubin G et al., 2007	Prospective Cohort	2520 (N/A)	age, no.: 65-69 years = 780, 70-74 years = 829, 77-79 years = 553, 80-85 years = 350	USA	Physician diagnosed – 15-letter loss of visual acuity (0.3 logMAR i.e. VA 20/40)	Drivers with a VA better than 20/40.	HR	**Any MVC:** 1.06 (0.77, 1.68)
**Included in Meta-analysis (at-fault MVC involvement)**								
Green K et al., 2013	Retrospective Cohort	2000 (N/A)	Age, no.: 70-79 years = 1432, 80-89 years = 526, 90-99 years = 40	USA	VA worse than 20/40 = impairment	Drivers with VA better than 20/40	RR (rate ratio)	**At-fault MVC**: 1.08 (0.71, 1.4)
Huisingh C et a., 2017	Prospective Cohort	659 (35/624)	N/A	USA	Distance VA > 0.3logMAR	Drivers with VA 20/40 or better	RR (rate ratio)	**At-fault MVC:** 1.09 (0.58, 2.05
**Included in Narrative Summary Only – High Income Countries**								
Alvarez-Peregrina C et al., 2021	Cross-sectional	736 (548/188)	46.4	Spain	Physician diagnosed - cut-off not defined in study	Drivers without a VA impairment	X^2 (Chi Square)	Poor VA was linked with increased risk of MVCs (p< 0.001).
Cross JM et al., 2009	Cross-sectional	3158 (1323/1835)	71.9	USA	VA worse 20/20 and better 20/40	Those with binocular acuity of 20/20 or better in any MVC	RR (rate ratio)	**Any MVC:** 1 (0.78, 1.29)

					VA worse 20/20 and better 20/40	Those with binocular acuity of 20/20 or better in injurious MVC		**Injurious MVC:** 0.54 (0.28, 1.01)
					VA worse 20/20 and better 20/40	Those with binocular acuity of 20/20 or better in at-fault MVC		**At-fault MVC:** 1.08 (0.72, 1.62)
					VA 20/40 or worse	Those with binocular acuity of 20/20 or better in any MVC		**Any MVC:** 1.24 (0.74, 2.09)
					VA 20/40 or worse	Those with binocular acuity of 20/20 or better in injurious MVC		**Injurious MVC:** 0.55 (0.11, 2.8)
					VA 20/40 or worse	Those with binocular acuity of 20/20 or better in at-fault MVC		**At-fault MVC:** 1.37 (0.66, 2.82)
Gresset J et al., 1994	Case Control	4036 (151/3885)	N/A	Canada	Physician diagnosed poor VA	Those with better VA	OR	**Any MVC:** 0.99 (0.71, 1.4)
							Prevalence (%)	8.4% (118/1400) of those involved in an MVC had poor VA.

For peer review only

Gresset J et al., 1994	Case Control	4021 (N/A)	N/A	Canada	VA equal to 6/12 or 6/15 and normal binocularity	Drivers with VA 20/40 or better	OR	**Any MVC**: 0.97 (0.68, 1.38)*
					VA equal to 6/12 or 6/15 and lack of binocular vision			**Any MVC:** 1.23 (0.88, 1.72)*
Huisingh C et a., 2017	Prospective Cohort	659 (35/624)	N/A	USA	Distance VA > 0.3logMAR	Drivers with VA 20/40 or better	RR (rate ratio)	**Major MVC:** 0.81 (0.29, 2.26)
								**Any MVC:** 1.29 (0.87, 1.93)
		659 (74/585)			Near VA > 0.3 logMAR			**Major MVC:** 1.54 (0.9, 2.63)
								**At-fault MVC:** 1.19 (0.77, 1.85)
Ivers R et al., 1999	Cross-sectional	3654 (N/A)	N/A	Australia	Best eye VA <20/40-20/60	drivers with Best eye VA >/=20/40	Prevalence ratio (PR)	**Any MVC:** 1.3 (0.6, 2.8)
					Best eye VA <20/60	drivers with Best eye VA >/=20/40		**Any MVC:** 1.2 (0.3, 5)
					Right eye VA <20/40-20/60	drivers with Right eye VA >/=20/40		**Any MVC:** 0.7 (0.3, 1.6)
					right eye VA<20/60	drivers with right eye VA >/=20/40		**Any MVC:** 2 (1.2, 3.5)
					left eye VA <20/40-20/60	drivers with left eye VA >/=20/40		**Any MVC:** 1.1 (0.5, 2)

For peer review only

					left eye VA<20/60	drivers with left eye VA >/=20/40		**Any MVC:** 1.1 (0.5, 24)
Keeffe JE et al., 2002	Retrospective Cohort	2594 (N/A)	62.5	Australia	Visual acuity <6/12	Drivers with better vision (>6/12)	X^2 (Chi Square)	People with impaired vision (<6/12) were no more likely to have an accident or to attribute that the accident was the result of impaired vision; X2= 0.175 (p>0.9)
							Prevalence (%)	9.5% (32/339) of participant involved in an MVC had poor VA.
Kwon M et al., 2016	Cross-sectional	1899 (145/1754)	age, no.: 70-79 years = 1358, 80-89 years = 502, 90-98 years = 39	USA	Low VA classified as <20/40 (0.3 logMAR)	Drivers with glaucoma and binocular VA ≥ 20/20	RR (rate ratio)	**Any MVC:** 1.51 (0.55, 4.16)
McCloskey L et al., 1994	Case Control	683	age, no.: 65- 69 years = 264, 70-74	USA	Uncorrected VA of 20/25 or 20/30	Drivers with VA 20/15 or 20/20	RR (relative risk)	**Injurious MVC**: 2.5 (0.8, 7.2)*

For peer review only

			years = 195, 75-79 years = 138, 80+ years = 86		Uncorrected VA of 20/40			**Injurious MVC:** 1.7 (0.6, 5.3)*
					Uncorrected VA 20/50 or 20/60			**Injurious MVC:** 2.4 (0.8, 7.2)*
					Uncorrected VA 20/70 of greater			**Injurious MVC:** 2.1 (0.7, 5.8)*
					Corrected VA 20/25 or 20/30			**Injurious MVC:** 0.7 (0.5, 1.1)*
					Corrected VA 20/40			**Injurious MVC:** 0.6 (0.3, 1.2)*
					Uncorrected VA 20/50 or 20/60			**Injurious MVC:** 0.3 (0.1, 0.9)*
					Uncorrected VA 20/70 of greater			**Injurious MVC:** 4.3 (0.5, 40.3)*
McGwin G Jr et al., 2000	Case Control	901 (104/797)	N/A	USA	Near vision impairment	Not-at-fault drivers involved in crashes without poor near vision	OR	**Not-at-fault MVC:** 1.6 (0.8, 3.3)
							Prevalence (%)	8% (16/198) of not-at-fault MVCs involved drivers with near vision impairment.<br><br>13.2% (33/249) of at-fault MVCs involved drivers

For peer review only

								with near vision impairment.
		901 (339/562)			Far vision impairment	Not-at-fault drivers involved in crashes without poor far vision	OR	**Not-at-fault MVC:** 1.1 (0.7, 1.7)
							Prevalence (%)	36% (71/198) of not-at-fault crashes involved drivers with far vision impairment.<br><br>41% (102/249) of at-fault crashes involved drivers with far vision impairment.
		901 (57/844)			Peripheral vision impairment	Not-at-fault drivers involved in crashes without poor peripheral vision	OR	**Not-at-fault MVC:** 1.6 (0.7, 3.9)
							Prevalence (%)	4.7% (9/198) of not-at-fault crashes involved drivers with peripheral vision impairment.<br><br>8.5% of at-fault crashes

For peer review only

								involved drivers with peripheral vision impairment.
Ono T et al., 2015	Cross-sectional	386 (N/A)	64.7	Japan	BCVA in the **better eye** LogMAR per 0.1 increment	POAG drivers with BCVA in both eyes of 0.7 or more	OR	**Any MVC:** 0.94 (0.87, 1.01)
Owsley C et al., 2001	Cross-sectional	377 (136/241)	69.9	USA	VA 20/25 - 20/30 in better eye	Drivers with VA 20/25 or better in the **better eye**	OR	**At-fault MVC:** 1.88 (0.72, 4.88)
		377 (118/259)			VA 20/35 - 20/50 in better eye			**At-fault MVC:** 2.54 (0.87, 7.47)
		377 (77/300)			worse than VA 20/50 in better eye			**At-fault MVC:** 1.75 (0.45, 6.85)
		377 (51/326)			VA 20/25 - 20/30 in worse eye	Drivers with VA 20/25 or better in the **worse eye**		**At-fault MVC:** 0.19 (0.03, 1.27)
		377 (67/310)			VA 20/35 - 20/50 in worse eye			**At-fault MVC:** 0.82 (0.19, 3.61)
		377 (110/267)			worse than VA 20/50 in worse eye			**At-fault MVC:** 0.74 (0.16, 3.52)
		377 (N/A)			VA impairment defined as worse than 20/50 in only 1 eye	Drivers with no VA impairment (better than VA 20/50)		**At-fault MVC:** 1.35 (0.58, 3.15)

For peer review only

		377 (N/A)			VA impairment defined as worse than 20/50 in both eyes	Drivers with no VA impairment (better than VA 20/50)		**At-fault MVC:** 1.01 (0.29, 3.45)
Owsley C et al., 1998	Case Control	294 (36/258)	71	USA	VA worse than 20/40	Drivers with VA 20/40 or better	OR	**Injurious MVC:** 1.6 (0.6, 3.8)*<br>**Non-injurious MVC:** 1.6 (0.7, 3.6)*
Rubin G et al., 2007	Prospective Cohort	2520 (N/A)	age, no.: 65-69 years = 780, 70-74 years = 829, 77-79 years = 553, 80-85 years = 350	USA	Physician diagnosed – 15-letter loss of visual acuity (0.3 logMAR i.e. VA 20/40)	Drivers with a VA better than 20/40.	HR	**Any MVC (at low luminance):** 1.06 (0.75, 1.47)
Sims RV et al., 1998	Case Control	174 (N/A)	71.1	USA	Physician Diagnosed	Older drivers without crashes in 6 years preceding 1991	Univariate analysis using student t-tests	Mean (SD) VA of those with a history of MVCs was 0.09 (0.31), compared to 0.03 (0.19) in those without a history of MVCs (p= 0.001).
Yuki K et al., 2014	Cross-sectional	247 (N/A)	63.7	Japan	Physician diagnosed as better VA (LogMar)	Drivers with POAG but without a	Unpaired t-test with Benjamini's correction	Differences between the VA of those who had a history of

For peer review only

						history of MVCs		an MVC and those who did not was significant, p= 0.036
					Physician diagnosed as worse VA (logMar)			Differences between the VA of those who had a history of an MVC and those who did not was not significant, p= 0.6
Yuki K et al., 2016	Prospective Cohort	191 (N/A)	63.7	Japan	POAG with 0.01 logMAR increase in **worse eye**	Drivers with POAG but without a history of MVCs	OR	**Any MVC:** 1.2 (1.1, 1.4)*
					POAG with 0.001 increase logMAR in **the better eye**			**Any MVC:** 0.68 (0, 221)*
**Included in Narrative Summaries only – Low Income Countries**								
Adekoya BJ et al., 2009	Cross-sectional	399 (N/A)	44.7	Nigeria	VA 6/9 in the better eye	N/A – looked at all participants	X^2 (Chi Square)	Inadequate VA in the better eye is not associated with MVC involvement in the last 10 years; X2= 0.035 (p= 0.851)

For peer review only

					VA 6/24 in the better eye		X^2 (Chi Square)	Inadequate VA in the second eye is not associated with involvement in RTA in the last 10 years; X2= 0.372 (p= 0.542)
Bekibele CO et al., 2007	Cross-sectional	99 (16/83)	50.1	Nigeria	Presenting vision less than 6/9 and improved with the aid of a minimum of 0.5 Diopter lenses, with VA <6/18	Drivers without refractive error	OR	**Any MVC:** 1.2 (0.4, 3.7)*
Boadi-Kusi SB et al., 2016	Cross-sectional	520 (38/482)	39.2	Ghana	Visual acuity of less than 0.2, either monocularly or binocularly, was classified as poor vision	N/A	X^2 (Chi Square)	No statistically significantly associations between poor vision due to refractive error and MVC involvement: X2= 3.090 (p= 0.388)
Humphriss D, 1987	Cross-sectional	366 (N/A)	N/A	South Africa	Binocular	Better mean vision test scores for binocular VA	Mean (SD)	Drivers involved in accidents were more likely to have worse mean vision test

For peer review only

								scores (10.031) for binocular VA compared to accident-free drivers (10.847), p<0.001
					right eye monocular VA	Better mean vision test scores for right eye monocular VA	Mean (SD)	Drivers involved in accidents more likely to have worse mean vision test scores (9.219) for right eye monocular VA compared to accident-free drivers (10.100), p<0.001
					left eye monocular VA	Better mean vision test scores for left eye monocular VA	Mean (SD)	Drivers involved in accidents more likely to have worse mean vision test scores (9.031) for left eye monocular VA compared to accident-free drivers (10.024), p<0.001

For peer review only

					Worse eye monocular acuity	Better mean vision test scores for worse eye monocular VA	Mean (SD)	Drivers involved in accidents more likely to have worse mean vision test scores for depth perception (4.128) compared to accident-free drivers (5.000), p<0.001
Isawumi MA et al., 2011	Cross-sectional	99 (5/94)	45.9	Nigeria	Poor driving vision if VA <6/12 in either eye	Drivers with an MVC but with normal vision.	X^2 (Chi Square)	MVCs were not directly related with VA and vice versa; X2= 1.6 (p= 0.65)
Oladehinde MK et al., 2007	Cross-sectional	215	41.5	Nigeria	Visual acuity < 6/18 - 6/60 was classified as visual impairment and < 6/60 - 3/60 was classified as severe visual impairment. VA < 3/60 was classified as blindness.	Drivers with VA 20/20 - 20/40	RR (did not state test used)	**Any MVC:** 3.5 (2.38, 5.14)*
Ogbonnaya CE et al., 2018	Cross-sectional	103 (7/96)	43.2	Nigeria	Minimum VA of 6/9 in the	Drivers without vision	X^2 (Chi Square)	The relationship between visual

For peer review only

					better eye and 6/12 in the worse eye of commercial motor vehicle drivers. Visually unfit to drive if VA <6/12 in the poorer eye.	impairment and no MVC history.		acuity fitness for driving and self-reported history of MVC was not statistically significant; X2= 0.05 (p= 0.82).
Ovenseri-Ogomo G et al., 2011	Cross-sectional	206 (14/192)	39.2	Ghana	VA < 6/18 in the better eye	Drivers without a history of MVC	X^2 (Chi Square)	VA not associated with history of MVC involvement; X^2= 5.982 (p=0.05)
Vofo BN et al. 2021	Cross-sectional	207 (51/156)	41.8	Cameroon	VA < 0.5	Drivers with VA > 0.5	Mean (SD)	Drivers with VA < 0.5 had a higher than average number of MVCs (2.91 +/- 1.72) compared to drives with VA > 0.5 (1.01 +/- 1.33).

*unadjusted results

For peer review only

Table 4a(ix) All studies (n=13) on contrast sensitivity (CS) impairment and Motor Vehicle Crashes (MVC), with only two studies suitable for meta-analysis due to different CS cut-off points, type of crash outcome explored and comparators used for each study.

**Author and Year**	**Study Design**	**Total Participants (exposure/control)**	**Mean Age**	**Country**	**VI Definition**	**Comparator**	**Outcome Measure (OR, RR, HR, etc. ?)**	**Effect Measure (with 95% CI) + any description of results (if appropriate)**
**Included in Meta-analysis (any MVC involvement)**								
Huisingh C et al., 2017	Prospective Cohort	659 (291/368)	N/A	USA	CS in better eye (< 1.5)	Drivers with CS ≥ 1.5 in better eye	RR (rate ratio)	**Any MVC**: 1.22 (0.82, 1.81)
Swain TA et al., 2021	Cross-sectional	159 (17/142)	79.3	USA	CS of <1.5 log sensitivity in the worse eye	Drivers with CS of >1.5 log sensitivity in the worse eye	RR	**Any MVC:** 1.5 (0.8, 3.2)
**Included in Narrative Summaries Only – High Income Countries**								
Cross JM et al., 2009	Cross-sectional	3158 (1323/1835)	71.9	USA	CS is >=1.575 and <1.675	Drivers without binocular CS impairments	RR (rate ratio)	**Any MVC:** 0.91 (0.68, 1.23)
								**Injurious MVC:** 0.94 (0.56, 1.58)
								**At-fault MVC**: 0.72 (0.49, 1.05)
					CS is >=1.450 and <1.575	Drivers without binocular CS	RR (cox proportional hazards)	**Any MVC:** 0.72 (0.49, 1.05)
								**Injurious MVC:** 0.71 (0.32, 1.56)
								**At-fault MVC**: 0.87 (0.49, 1.56)
					CS is <1.450	Drivers without binocular CS	RR (cox proportional hazards)	**Any MVC:** 1.01 (0.66, 1.55)
								**Injurious MVC:** 0.49 (0.16, 2.37)

For peer review only

								**At-fault MVC:** 1.27 (0.68, 2.37)
Green K et al., 2013	Retrospective Cohort	2000 (N/A)	Age, no.: 70-79 years = 1432, 80-89 years = 526, 90-99 years = 40	USA	Impairment defined as <1.5 on Pelli-Robson chart.	Drivers without binocular CS impairments	RR (rate ratio)	**Any MVC**; 1.42 (1, 2.02)<br>**At-fault MVC**: 1.52 (0.93, 2.68)
Huisingh C et al., 2017	Prospective Cohort	659 (291/368)	N/A	USA	CS in worse eye (< 1.5)	Drivers with CS ≥ 1.5 in worse eye		**Any MVC:** 1.38 (1.05, 1.81)
					CS in better eye (< 1.5)	Drivers with CS ≥ 1.5 in better eye		**Major crash involvement**: 1.29 (0.77, 2.18)
					CS in worse eye (< 1.5)	Drivers with CS ≥ 1.5 in worse eye		**Major crash involvement:** 1.54 (1.07, 2.23)
					CS in better eye (< 1.5)	Drivers with CS ≥ 1.5 in better eye		**At-fault MVC:** 1.28 (0.84, 1.94)
					CS in worse eye (< 1.5)	Drivers with CS ≥ 1.5 in worse eye		**At-fault MVC: 1.44** (1.08, 1.93)
Ivers R et al., 1999	Cross-sectional	3654 (N/A)	N/A	Australia	Vectorvision CSV-1000 chart: 3 cycle per degree in best eye CS	Reference group ≤ 2 units compared with >2 on a scale of 1-8	PR (Prevalence Ratio)	**Any MVC:** 1.3 (0.7, 2.2)

For peer review only

					Vectorvision CSV-1000 chart: 6 cycle per degree in best eye CS eye CS			**Any MVC:** 1.2 (0.7, 2.1)
					Vectorvision CSV-1000 chart: 12 cycle per degree in best eye CS			**Any MVC:** 1.4 (0.8, 2.3)
					Vectorvision CSV-1000 chart: 18 cycle per degree in best eye CS			**Any MVC:** 1.4 (0.9, 2.3)
					Vectorvision CSV-1000 chart: 3 cycle per degree in right eye CS			**Any MVC:** 1.2 (0.8, 1.9)
					Vectorvision CSV-1000 chart: 6 cycle per degree in right eye CS eye CS			**Any MVC:** 1 (0.6, 1.5)
					Vectorvision CSV-1000 chart: 12 cycle per degree in right eye CS			**Any MVC:** 2 (1.2, 3.1)

For peer review only

					Vectorvision CSV-1000 chart: 18 cycle per degree in right eye CS			**Any MVC:** 1.3 (0.8, 2.2)
					Vectorvision CSV-1000 chart: 3 cycle per degree in left eye CS			**Any MVC:** 1 (0.6, 1.6)
					Vectorvision CSV-1000 chart: 6 cycle per degree in left eye CS eye CS			**Any MVC:** 1.1 (0.6, 1.7)
					Vectorvision CSV-1000 chart: 12 cycle per degree in left eye CS			**Any MVC:** 1.3 (0.8, 2.2)
					Vectorvision CSV-1000 chart: 18 cycle per degree in left eye CS			**Any MVC:** 1.3 (0.8, 2.1)
Kwon M et al., 2016	Cross-sectional	1899 (432/1467)	age, no.: 70-79 years = 1358, 80-89 years =	USA	Pelli-Robson chart measure of ≤ 1.6 log sensitivity was defined as an impairment.	Older drivers with glaucoma, without CS impairment	RR (rate ratio)	**Any MVC**: 0.72 (0.36, 1.42)

For peer review only

			502, 90-98 years = 39					
Margolis KL et al., 2002	Prospective Cohort	1416 (N/A)	71.3	USA	low spatial frequencies per standard deviation change	N/A – looked at MVC information from all participants from 1986-1995	HR	**Any MVC:** 0.99 (0.89, 1.1)
					high spatial frequencies per standard deviation change			**Any MVC:** 0.94 (0.85, 1.04)
Owsley C et al., 1998	Case Control	294 (56/238)	71	USA	Pelli-Robson chart measure of <= 1.5 log sensitivity was defined as an impairment.	Older drivers with log(CS) >1.5	OR	**Injurious MVC:** 0.9 (0.4, 1.8)*
								**Non-injurious MVC:** 0.7 (0.3, 1.3)*
Owsley C et al., 2001	Cross-sectional	377 (274/103)	69.9	USA	CS impairment defined as ≤1.25	Participants with no CS impairment (CS >=1.50)	OR	**At-fault MVC (better eye CS >1.35 – 2.50):** 1.18 (0.41, 3.36)
								**At-fault MVC (better eye CS >1.25-1.35):** 1.21 (0.4, 3.68)
								**At-fault MVC (better eye CS ≤1.25):** 3.78 (1.15, 12.48)

For peer review only

								**At-fault MVC (worse eye CS >1.35 – 2.50):** 3.28 (0.71, 14.17)
								**At-fault MVC (worse eye CS >1.25-1.35):** 4.36 (0.84, 22.7)
								**At-fault MVC (worse eye CS ≤1.25):** 7.86 (1.55, 39.79)
								**At-fault MVC (unilateral CS ≤1.25):** 2.7 (1.16, 6.51)
								**At-fault MVC (bilateral CS ≤1.25):** 5.78 (1.87, 18.86)
Owsley C et al., 2020	Cross-sectional	915 (179/155)	age, no.: 60-69 years = 310, 70-79 years = 396, 80-90 years =	USA	Low photopic area under the log CS function	Drivers with higher photopic peak log sensitivity	RR (rate ratio)	**All MVC:** 0.8 (0.61, 1.04)
								**At-fault MVC:** 0.77 (0.57, 1.03)
					Low photopic peak log sensitivity			**All MVC:** 0.8 (0.61, 1.04)
								**At-fault MVC:** 0.77 (0.58, 1.03)

For peer review only

			200, 90-99 years = 9		Low mesopic area under the log CS function	Drivers with higher mesopic peak log sensitivity		**All MVC:** 1.36 (1.06, 1.72)<br>**At-fault MVC:** 1.28 (1.01, 1.63)
					Low mesopic peak log sensitivity			**All MVC**: 1.5 (1.16, 1.93)<br>**At-fault MVC:** 1.38 (1.07, 1.78)
Rubin G et al., 2007	Prospective Cohort	2520 (N/A)	age, no.: 65-69 years = 780, 70-74 years = 829, 77-79 years = 553, 80-85 years = 350	USA	Pelli-Robson Chart: 6 letter worsening (worsening of 0.3 logCS units)	N/A – looked at driver with and without MVC in whole population.	HR	**Any MVC when CS < 1.7**: 0.75 (0.49, 1.21)<br>**Any MVC when CS ≥ 1.7**: 1.25 (0.44, 5.65)
Swain TA et al., 2021	Prospective Cohort	154 (17/137)	79.3	USA	CS of <1.5 log sensitivity in the worse eye	Drivers with CS of >1.5 log sensitivity in the worse eye	RR	**At-fault or near crash involvement:** 2.7 (1.3, 5.5)

For peer review only

Table 4a(x) All studies (n=20) on visual field (VF) impairment and Motor Vehicle Crashes (MVC) with meta-analysis suitable for only four studies on associations with any MVC involvement

Additional Narrative Summary:
When comparing impairments in the upper, lower, left, right, vertical and horizontal visual fields, impairments on the left side were found to be the most significant predictor of crash involvement (data from US, driving on right side of road).

Author and Year	Study Design	Total Participants (exposure/control)	Mean Age	Country	VI Definition	Comparator	Outcome Measure (OR, RR, HR, etc.?)	Effect Measure (with 95% CI) + any description of results (if appropriate)
**Included in meta-analysis (any MVC involvement)**								
Huisingh C et al., 2015	Cross-sectional	2000 (N/A)	N/A	USA	Bilateral VF impairment	Drivers without visual field impairments	RR (rate ratio)	**Any MVC:** 1.4 (1.07, 1.83)
Oladehinde MK et al., 2007	Cross-sectional	215 (22/193)	41.5	Nigeria	Bilateral VF impairment	Drivers without a MVC history	RR (risk ratio)	**Any MVC:** 1.07 (0.98, 6.73)
Piyasena P et al., 2021	Systematic Review	15394 (337/15057)	39.3	N/A	Physician Diagnosed	Drivers without VF impairment	RR	**Any MVC:** 1.36 (1.25, 1.48)
Swain TA et al., 2021	Cross-sectional	159 (40/119)	79.3	USA	Overall VF loss of ≤ 22.4dB in the worse eye	Drivers with no overall VF loss in the worse eye	RR	**Any MVC:** 1.6 (0.8, 3.1)
**Included in Narrative Summaries Only – High Income Countries**								
Ball K et al., 1993	Cross-sectional	294 (N/A)	71	USA	Sensitivity loss in the 30 to 60 degree region of the visual field	N/A	Spearman's Correlation (r)	VF loss was significantly related to crash frequency however the LISREL model shows that it only has indirect effects

For peer review only

								on crash frequency but direct effects on UFOV which is the most significantly associated variable with crash frequency; 0.26
Huisingh C et al., 2015	Cross-sectional	2000 (N/A)	N/A	USA	Upper field impairments	Drivers without visual field impairments	RR (rate ratio)	**Any MVC:** 1.1 (0.83, 1.44)
					Lower Field Impairments			**Any MVC:** 1.4 (1.07, 1.82)
					Horizontal Meridian Impairments			**Any MVC:** 1.31 (1, 1.72)
					Vertical Meridian Impairments			**Any MVC:** 1.26 (0.97, 1.65)
					Left Side impairments			**Any MVC:** 1.49 (1.15, 1.92)
					Right side impairments			**Any MVC:** 1.16 (0.88, 1.53)
Huisingh C et al., 2017	Prospective Cohort	659 (406/253)	N/A	USA	Peripheral visual field loss at 70 or 85 degrees temporally in **either eye**	Drivers with no visual field loss in either eye	RR (rate ratio)	**Any MVC:** 1.08 (0.8, 1.47)<br>**Major MVC:** 1.53 (1.02, 2.29)<br>**At-fault MVC:** 0.98 (0.71, 1.37)

For peer review only

		659 (186/473)			Peripheral visual field loss at 70 or 85 degrees temporally in **both eye**	Drivers with no visual field loss in both eyes		**Any MVC:** 1.74 (1.18, 2.56)
								**Major MVC:** 2.32 (1.4, 3.83)
								**At-fault MVC:** 0.73 (1.14, 2.61)
Kwon M et al., 2016	Cross-sectional	1899 (N/A)	age, no.: 70-79 years = 1358, 80-89 years = 502, 90-98 years = 39	USA	Overall visual field loss ≤ 22.5 dB	Drivers (with glaucoma) without severe visual field loss.	RR (rate ratio)	**Any MVC:** 2.11 (1.09, 4.09)
					Upper visual field loss ≤ 22.5 dB			**Any MVC:** 2.37 (1.19, 4.74)
					Lower visual field loss ≤ 22.5 dB			**Any MVC:** 2.32 (1.13, 4.75)
					Left visual field loss ≤ 22.5 dB			**Any MVC:** 3.16 (1.55, 6.46)
					Right visual field loss ≤ 22.5 dB			**Any MVC:** 1.63 (0.84, 3.14)
					Horizontal meridian loss ≤ 22.5 dB			**Any MVC:** 1.78 (0.92, 3.44)
					Vertical meridian loss ≤ 22.5 dB			**Any MVC:** 1.09 (0.56, 2.11)
Kristalovich L et al., 2019	Retrospective cohort	445 (286/159)	N/A	Canada	Loss of at least 120 continuous degrees along the horizontal meridian and 15 continuous	Drivers with either no VFI or with VFI but meeting licensing standards	X^2 (Chi Square)	No significant difference in rate of crash between VFI/not meet licensing

					degrees above and below fixation with both eyes open and examined together.			standards and no VFI and VFI/meet licensing standards (p=0.402)
McGwin G Jr et al., 2015	Retrospective Cohort	438 (N/A)	72.8	USA	Binocular visual field **total deviations <7.25**	Drivers (with glaucoma) without severe visual field impairments	RR (rate ratio)	**At-fault MVC:** 1.5 (0.82, 2.74)
					Binocular visual impairment severely **impaired threshold** <20.4			**At-fault MVC:** 1.49 (0.81, 2.74)
					Binocular visual impairment severely impaired **pattern deviation <3.97**			**At-fault MVC:** 2.13 (1.21, 3.75)
Owsley C et al., 1998	Case Control	294 (36/258)	71	USA	Central 30 degree VF sensitivity: >10	Older drivers with central 30 degree VF sensitivity of 0-10	OR	**Injurious MVC:** 2.6 (1.1, 6.3)*
								**Non-injurious MVC:** 1.8 (0.8, 4.4)*
		294 (108/186)			Peripheral 20-60 degree VF sensitivity: >10	Older drivers with peripheral 30-60 degree VF sensitivity of 0-10		**Injurious MVC:** 2.4 (1.3, 4.5)*
								**Non-injurious MVC:** 1.8 (1, 3.1)*
Rubin G et al., 2007	Prospective Cohort	2520 (N/A)	age, no.: 65-69	USA	Binocular visual field <20 (loss of 15 points)	N/A – looked at drivers with and without MVC in	HR	**Any MVC:** 0.59 (0.34, 1)

For peer review only

			years = 780, 70-74 years = 829, 77-79 years = 553, 80-85 years = 350		Binocular visual field >=20 (loss of 15 points)	whole population.		**Any MVC:** 1.31 (1.31, 4.27)
Swain TA et al., 2021	Cross-sectional	159 (41/118)			Peripheral VF loss of ≤ 19.2dB in the worse eye	Drivers with no peripheral VF loss in the worse eye		**Any MVC:** 2.4 (1.3, 4.4)
		159 (41/118)			Superior VL loss of ≤ 22.0dB in the worse eye	Drivers with no superior VF loss in the worse eye		**Any MVC:** 0.7 (0.4, 1.5)
		159 (41/118)			Inferior VL loss of ≤ 22.1dB in the worse eye	Drivers with no inferior VF loss in the worse eye		**Any MVC:** 1.7 (0.4, 1.5)
		159 (40/119)			Left VL loss of ≤ 21.6dB in the worse eye	Drivers with no left VF loss in the worse eye		**Any MVC:** 1.7 (0.9, 3.2)
		159 (41/118)			Right VF loss of ≤ 21.8dB in the worse eye	Drivers with no right VF loss in the worse eye		**Any MVC:** 1.6 (0.9, 3)
Swain TA et al., 2021	Prospective Cohort	154 (38/116)	79.3	USA	Overall VF loss of ≤ 22.4dB in the worse eye	Drivers with no overall VF loss in the worse eye	RR	**At-fault or near crash:** 1.4 (0.8, 2.8)

For peer review only

		154 (40/114)			Peripheral VF loss of ≤ 19.2dB in the worse eye	Drivers with no peripheral VF loss in the worse eye		**At-fault or near crash:** 1.8 (1, 3.3)
		154 (43/111)			Superior VL loss of ≤ 22.0dB in the worse eye	Drivers with no superior VF loss in the worse eye		**At-fault or near crash:** (1.3 (0.7, 2.5)
		154 (41/113)			Inferior VL loss of ≤ 22.1dB in the worse eye	Drivers with no inferior VF loss in the worse eye		**At-fault or near crash:** (1.4, 0.8, 2.5)
		154 (42/112)			Left VL loss of ≤ 21.6dB in the worse eye	Drivers with no left VF loss in the worse eye		**At-fault or near crash:** 1.3 (0.7, 2.5)
		154 (36/118)			Right VF loss of ≤ 21.8dB in the worse eye	Drivers with no right VF loss in the worse eye		**At-fault or near crash:** 0.9 (0.5, 1.8)
Yuki K et al., 2014	Cross-sectional	247 (N/A)	63.7	Japan	N/A	POAG drivers without a MVC history	Mean (SD)	The mean IVF-MD (db) of glaucoma drivers with a history of MVCS was -0.6 (3.4) compared to - 0.8 (3.7) in glaucoma drivers without a history of MVCs.
Yuki K et al., 2016	Prospective Cohort	191 (N/A)	63.7	Japan	POAG with **1dB increase in visual field**	POAG drivers without a MVC history	OR	**Any MVC:** 0.95 (0.8, 1.1)*
							Mean (SD)	Mean (SD) IVF-MD (dB) of glaucoma

For peer review only

								drivers with a history of MVC was -2.1 (3.9) compared to -1.6 (3.7) in glaucoma drivers without a history of MVCs.
**Included in Narrative Summaries Only – Low Middle Income Countries**								
Abraham EG et al., 2010	Cross-sectional	291 (13/278)	41.5	Nigeria	Cup-disc ratio >0.5 cup-disc disparity between the two eyes of up to 0.2 or more, abnormal disc pallor (localised or generalised)	Drivers without visual field impairments.	RR (relative risk)	**Any MVC:** 0.628*
Adekoya BJ et al., 2009	Cross-sectional	399 (21/378)	44.7	Nigeria	Presence of 1 or more abnormal quadrants on confrontation perimetry	N/A	X^2 (Chi Square)	Abnormal visual fields was not associated with MVC involvement in the last 10 years; X2= 1.715 (p= 0.19).
Humphriss D, 1987	Cross-sectional	366 (N/A)	N/A	South Africa	N/A	Drivers with no MVC history	Mean (SD)	Data not reported
Isawumi et al., 2011	Cross-sectional	99 (N/A)	45.9	Nigeria	N/A	Drivers with a MVC but	Prevalence (%)	21.1% (8/38) of drivers with an MVC also had

						without visual field loss		horizontal visual field loss.
Ovenseri-Ogomo G et al., 2011	Cross-sectional	206 (14/192)	39.2	Ghana	VA < 6/18 in the better eye	Drivers without a history of MVC	OR	Any MVC: 0.54 (0.016, 18.45)*
Pepple G et al., 2014	Cross-sectional	400 (16/384)	37.8	Nigeria	Physician diagnosed	Drivers without visual field impairments	RR (did not state test used)	**Any MVC:** 1.25*
							Prevalence (%)	56% (9/16) of those with visual field impairment were have been involved in an MVC.

*unadjusted results

For peer review only

Table 4a(xi) All studies (n=3) on glare sensitivity (GS) impairment and Motor Vehicle Crashes (MVC), all suitable to only be summarised narratively due to their different GS cut-off points, type of crash outcome explored and comparators

Author and Year	Study Design	Total Participants (exposed/control)	Mean Age/ Age Range	Country	VI Definition	Comparator	Outcome Measure (OR, RR, HR, etc.?)	Effect Measure (with 95% CI) + any description of results (if appropriate)
**Included in Narrative Summaries Only – High Income Countries**								
Owsley C et al., 1998	Case Control	294 (71/179)	71	USA	Measured using MCT-8000 (Vis Tech), defined as disability glare >0	Older drivers with disability glare <= 0	OR	**Injurious MVC**: 1.4 (0.8, 2.5)*
								**Non-injurious MVC:** 1.3 (0.9, 2.2)*
Owsley C et al., 2001	Cross-sectional	377 (274/103)	69.9	USA	Glare impairment defined as >=0.25, measured with Pelli-robson chart with BAT:	Those with disability glare <0.25 in the better/worse eye	OR	**At-fault MVC in the better eye:** 0.68 (0.22, 2.12)
					Glare impairment defined as >=0.25 in both eyes	those with no disability glare impairment (<0.25 score)	OR (logistic regression)	**At-fault MVC in the worse eye**: 0.62 (0.29, 1.33)
								**At-fault MVC in both eyes:** 0.46 (0.14, 1.53)
Rubin G et al., 2007	Prospective Cohort	2520 (N/A)	age, no.: 65-69 years = 780, 70-74 years = 829, 77-79 years =	USA	6 letter worsening (worsening of 0.3 logCS units) - measured using Pelli-Robson chart with BAT	N/A – looked at driver with and without MVC in whole population.	HR	**Any MVC (glare <3 letters)**: 0.46 (0.26, 0.89)
								**Any MVC (glare ≥ 3 letters)**: 2.3 (1.14, 16.78)

			553, 80-85 years = 350					

For peer review only

Table 4a(xii) All studies (n=19) on other types of vision impairment and Motor Vehicle Crashes (MVC), all suitable to only be summarised narratively

Author and Year	Study Design	Total Participants (exposure/control)	Mean Age/ Age Range	Country	Type of VI	VI definition	Comparator	Outcome Measure (OR, RR, HR, etc.?)	Effect Measure (with 95% CI) + any description of results (if appropriate)
**Included in Narrative Summaries only – High Income Countries**									
Baker JM et al., 2019	Retrospective Cohort	66253 (62/66191)	20.8	USA	Unilateral vision impairment	ICD-9 diagnostic codes (369.6-369.8)	Young adult drivers without unilateral vision impairment	HR	**Any MVC**: 1.08 (0.6, 1.95)
		66253 (352/65901)			Amblyopia	Using the ICD-9 diagnostic codes (368.00 - 368.03) in the HER with diganosis noted in medical record from age 6	Young adult drivers without amblyopia	HR	**Any MVC**: 1.08 (0.85, 1.38)
Crizzle AM et al., 2020	Cross-sectional	3346 (513/2833)	61.5	Canada	Vision impairment	Physician diagnosed	Drivers without vision impairment	Univariate log rank test	Vision impairment was not associated with history of MVCs (p=0.9178).

For peer review only

Fishman GA et al., 1981	Retrospective Cohort	129 (42/87)	37.3	USA	Retinitis Pigmentosa	Physician diagnosed	Drivers free from ophthalmic or general defects	X^2 (Chi Square)	Statistical significant difference in accidents recorded over 5 years between retinitis pigmentosa patients (50%; 21/42)) and controls (29%; 25/62); p= 0.02
Gresset J et al., 1994	Case Control	4036 (15/4021)	N/A	Canada	Monocularity	Physician diagnosed	Male drivers who had no accident during their 70th year in 1988 and 1989	OR	**Any MVC**: 0.95 (0.32, 2.77)
		4036 (327/3709)			Visual impairment			OR	**Any MVC:** 1.07 (0.84, 1.36)
Maag U et al., 1997	Retrospective Cohort	116 (N/A)	N/A	Canada	Vision impairment	Non stereoscopic vision (> 160 seconds); an acuity of 20/40 for the better eye and zero in the other.	Drivers in good health	Mean (SD)	Average total number of crashes in people with good health with a taxi per year (SD): 0.218 (0.501)<br>Average total number of crashes in people with binocular vision problems with

									taxi per year (SD): 0.369 (0.595); the difference was statistically significant (p= 0.01)
McCloskey L et al., 1994	Case Control	683 (10/673)	age, no.: 65-69 years = 264, 70-74 years = 195, 75-79 years = 138, 80+ years = 86	USA	Retinopathy	Physician diagnosed (hospital data)	Age-matched drivers with retinopathy who have not been injured in a police reported MVC in the same calendar year as their matched case.	RR (relative risk)	**Injurious MVC**: 0.6 (0.1, 2.6)*
		683 (37/646)			Retinal disorders	Physician diagnosed (hospital data)	Age-matched drivers with other retinal disorders who have not been injured in a police	RR (relative risk)	**Injurious MVC**: 0.8 (0.4, 1.6)*

For peer review only

							reported MVC in the same calendar year as their matched case.		
		683 (394/289)			Hypermetropia	Physician diagnosed (hospital data)	Age-matched drivers with hypermetropia who have not been injured in a police reported MVC in the same calendar year as their matched case.	RR (relative risk)	**Injurious MVC**: 0.9 (0.7, 1.4)*
		683 (544/139)			Presbyopia	Physician diagnosed (hospital data)	Age-matched drivers with presbyopia who have not been injured in a police reported MVC in the same calendar year as their	RR (relative risk)	**Injurious MVC**: 1 (0.6, 1.8)*

For peer review only

							matched case.		
		683 (339/344)			Astigmatism	Physician diagnosed (hospital data)	Age-matched drivers with astigmatism who have not been injured in a police reported MVC in the same calendar year as their matched case	RR (relative risk)	**Injurious MVC**: 0.9 (0.7, 1.4)*
		638 (597/41)			Refractive disorder	Physician diagnosed (hospital data)	Age-matched drivers with refractive disorders who have not been injured in a police reported MVC in the same calendar year as their matched case	RR (Mantel-Haenszel)	**Injurious MVC**: 0.3 (0.1, 0.8)*
		638 (6/632)			Monocular vision	Physician diagnosed (hospital data)	Age-matched drivers with monocular vision who have not	RR (relative risk)	**Injurious MVC**: 0.7 (0.1, 4.1)*

For peer review only

							been injured in a police reported MVC in the same calendar year as their matched case		
		638 (10/628)			Diplopia	Physician diagnosed (hospital data)	Age-matched drivers with diplopia who have not been injured in a police reported MVC in the same calendar year as their matched case	RR (relative risk)	**Injurious MVC**: 1.2 (0.4, 4.2)*
		638 (13/625)			Vision/ophthalmic conditions	Physician diagnosed (hospital data)	Age-matched drivers with other vision and opthalmic conditions who have not been injured in a police reported MVC in the same	RR (relative risk)	**Injurious MVC**: 0.6 (0.2, 1.6)*

For peer review only

							calendar year as their matched case		
Naredo Turrado J et al., 2020	Prospective Cohort	11670 (11/11659)	62.4	France	Retinal detachment	Self-reported physician diagnosed	Drivers without retinal detachment	OR	**Any MVC**: 0.99 (0.37, 2.7)
Owsley C et al., 1998	Case Control	294 (N/A)	71	USA	Stereoacuity	Scores ≥ 500 arcseconds on TNO test	Older drivers with stereoacuity <500 arcseconds	OR (logistic regression)	**Injurious MVC**: 2.2 (1.1, 1.4)*<br>**Non-injurious MVC**: 1.2 (0.7, 2.3)*
Pepple G et al., 2014	Cross-sectional	400 (32/368)	37.8	Nigeri a	Vision impairment	Physician diagnosed	Drivers without a vision impairment	RR (did not state test used)	**Any MVC**: 0.62 (p= 0.46)
Rubin G et al., 2007	Prospective Cohort	2520 (545/2066)	age, no.: 65-69 years = 780, 70-74 years = 829, 77-79 years = 553, 80-85 years = 350	USA	Stereoacuity	Stereodeficient was defined at failing the test at 457 arc seconds.	Drivers who were not sterodeficient	HR (cox proportion al hazard)	**Any MVC**: 1.44 (0,88, 2.27)

For peer review only

Runge JW, 2000	Cross-sectional	N/A	N/A	USA	Vision Impairment	Physician diagnosed	Drivers without vision impairment s	RR (relative risk)	**At-fault MVC**: 1.51*<br><br>The at-fault crash rate of those with a vision impairment was 1.14 compared to those without an impairment (0.75).
Rahi J et al., 2006	Retrospective Cohort	8661 (429/8432)	N/A	UK	Amblyopia	Mild = acuity 6/6 in one eye and 6/9 or 6/12 in the other and unilateral visual loss	People with normal vision in each eye	OR	**Any MVC**: 1.28 (0.87, 1.89)
						Moderate/severe = acuity of 6/6 in one eye and 6/18 or worse in the other and unilateral visual loss, with or without strabismus, earlier in childhood.	People with normal vision in each eye	OR (ordinal regression)	**Any MVC**: 2.33 (1.29, 4.2)
Wedenoja J et al., 2021	Cross-sectional	N/A	N/A	Finland	Vision impairment	Physician diagnosed	Drivers without	Prevalence	Only 1.3% (13/968) of all fatal MVCs were

For peer review only

							vision impairment.		caused by vision-related problems.
**Included in Narrative Summaries Only – Low Middle Income Countries**									
Ahmed M et al., 2021	Cross-sectional	700 (492/208)	42.3	Bangla desh	Near or distance visual impairment	Presenting VA ≥ 6/7.5 in the better eye and or presence of presbyopia.	Drivers without near or distance visual impairment but with a history of MVCs.	OR	**Any MVC:** 2.45 (1.09, 5.49)
		700 (125/575)	42.3	Bangla desh	Hyperopia	Physician diagnosed	Drivers without hyperopia but with a history of MVCs.	OR	**Any MVC:** 1.1 (0.56, 2.23)*
		700 (11/689)	42.3	Bangla desh	Presbyopia	Physician diagnosed	Drivers without presbyopia but with a history of MVCs.	OR	**Any MVC:** 1.7 (0.96, 3.01)*
		700 (N/A)	42.3	Bangla desh	Any distance refractive error	Physician diagnosed	Drivers without any distance refractive error but with a history of MVCs.	OR	**Any MVC:** 1.66 (0.88, 3.12)*
Biza M et al., 2013	Cross-sectional	249 (13/236)	33.6	Ethiop ia	Visual impairment	VA <6/18-6/60 was classified as	Drivers with a MVC but no	OR	**Any MVC (both eyes**

						moderate visual impairment and <6/60-3/60 was classified as severe VI while VA less than 3/60 was classified as blindness.	VA impairment		**impairment)**:42. 82 (2.53, 724.03)
									**Any MVC (right eye impairment)**: 0.03 (0.004, 0.28)*
									**Any MVC (left eye impairment)**: 0.09 (0.01, 0.97)*
Boadi-Kusi SB et al., 2016	Cross-sectional	520 (66/454)	39.2	Ghana	Hyperopia	Hyperopia defined as the spherical power in the better eye of +1.00D or more	Drivers with a history of MVC but no hyperopia	OR	**Any MVC**: 0 (0, 0);
		520 (30/490)			Astigmatism	Astigmatism was defined as −0.50D cylinder or worse in the better eye	Drivers with a history of MVC but no astigmatism	OR	**Any MVC**: 0.885 (0.32, 2.5)*
Emerole C et al., 2013	Cross-sectional	280 (102/178)	N/A	Nigeri a	Vision impairment causing poor visibility.	Physician diagnosed with VA of 6/30 classified as abnormal.	N/A – compared with a "control" group but paper never explained what/who	Prevalence (%)	119 (79.3%) participants in the study group had an MVC history.<br><br>40.3% (448/119) participants in the study group

							the control group was.		and 70.6% (36/51) in the control group listed poor visibility as the cause of their MVC involvement ($p < 0.05$).
Ogbonnaya CE et al., 2018	Cross-sectional	103 (9/94)	43.2	Nigeria	Monocular vision impairment	Physician diagnosed	Drivers with monocular impairment but with no MVC history	X^2 (Chi Square)	The relationship between monocular visual impairment and self-reported history of RTA was not statistically significant; X2 =0.045, (p= 0.85)
		103 (7/96)			Monocular blindness	Physician diagnosed	Drivers with monocular blindness but with no MVC history	X^2 (Chi Square)	The relationship between monocular blindness and self-reported history of RTA was not statistically significant; X2 =0.358 (p= 0.55)
Vofo BN et al., 2021	Cross-sectional	207 (51/156)	41.8	Cameroon	Self-reported vision impairment.	Self-reported	Drivers without self-reported	X^2 (Chi Square)	Drivers with self-reported VI were involved in significantly

							vision impairment		higher number of MVCs (72.5%) than those with self-reported good vision (55.8%) ($p < 0.05$)
									Drivers with self-reported VI had higher average number of MVCs over previous 10 years (1.75 +/- 1.64) than drivers with self-reported good vision (1.03 +/- 1.40 ($p < 0.05$).

*unadjusted results

For peer review only

Table 4a(xiii) All studies (n=6) evaluating cataract surgery and Motor Vehicle Crashes (MVC) with meta-analysis suitable for 3 studies on the associations with any MVC involvement

**Author and Year**	**Study Design**	**Total Participants (exposure/control)**	**Mean Age**	**Country**	**VI Definition**	**Comparator**	**Outcome Measure (OR, RR, HR?)**	**Effect Measure (with 95% CI)**
**Included in Meta-Analysis (any MVC involvement)**								
Meuleners L et al., 2012	Retrospective Cohort	27827 (N/A)	age, no.: 60-69 years = 6609, 70-79 years = 14506, 80+ years = 6712	Australia	Physician diagnosed	Crashes before surgery	RR (risk ratio)	0.87 (0.76, 0.99)
Meuleners L et al., 2019	Retrospective Cohort	2849 (N/A)	age, no.: 60-64 years = 347, 65-69 years = 482, 70-74 years = 720, 75-79 years = 719, 80-84 years = 454, 85 +	Australia	Physician diagnosed cataract	Crashes before surgery	RR (risk ratio)	0.39 (0.37, 0.41)

For peer review only

			years = 127					
Owsley C et al., 2002	Prospective Cohort	277 (174/103)	71.3	USA	Cataract in 1 or both eyes with best-corrected VA of 20/40 or worse	Crashes before surgery	RR (rate ratio)	0.47 (0.23, 0.94)
**Included in Narrative Summaries Only – High Income Countries**								
McCloskey L et al., 1994	Case Control	683 (235/448)	age, no.: 65- 69 years = 264, 70-74 years = 195, 75-79 years = 138, 80+ years = 88	USA	Self-reported physician diagnosed cataracts	Drivers who experienced no injuries in a crash.	RR (relative risk)	**Post-surgery with lens implant:**1 (0.5, 2.3)*
Meuleners L et al., 2012	Retrospective Cohort	Males: 1091 (513/611)<br>Females: 624 (308/330)	age, no.: 60-69 years = 447, 70-79 years = 823, 80 + years = 445	Australia	Physician diagnosed	No. of pre cataract surgery police reported crashes in all participants.	RR (risk ratio)	**Males:** 0.84 (0.72, 0.99)<br>**Females**: 0.99 (0.75, 1.16)
Meuleners L et al., 2019	Retrospective Cohort	2849 (N/A)	age, no.: 60-64 years = 347, 65-69 years	Australia	Physician diagnosed cataract	Crashes before surgery	RR (risk ratio)	**After 2nd eye cataract surgery:** 0.77 (0.75, 0.78)

			= 482, 70-74 years = 720, 75-79 years = 719, 80-84 years = 454, 85 + years = 127					
Schlenker M et al., 2018	Prospective cohort	559546 (N/A)	76	Canada	Physician diagnosed	No. of pre cataract surgery crashes in all participants	OR	0.91 (0.84, 0.97)*

*unadjusted results

For peer review only

Table 4a(xiv) All studies (n=1) evaluating corrective lens wear to improve refractive error and Motor Vehicle Crashes (MVC)

**Author and Year**	**Study Design**	**Total Participants (exposure/control)**	**Mean Age**	**Country**	**Vision impairment**	**VI Definition**	**Comparator**	**Outcome Measure (OR, RR, HR?)**	**Effect Measure (with 95% CI)**
McCloskey L et al., 1994	Case Control	683 (235/448)	age, no.: 65- 69 years = 264, 70-74 years = 195, 75-79 years = 138, 80+ years = 94	USA	Refractive Error	Use of corrective lenses for any reason (far or near vision)	Drivers who experienced no crash-related injuries	RR (risk ratio)	0.6 (0.3, 1.1)*
								Prevalence (%)	% with condition, cases: 91% (214/235) % with condition, controls: 94.6% (424/448)

* unadjusted results

For peer review only

Appendix 4b Raw data tables and additional narrative summaries of papers on driving cessation

Table 4b(i) All studies (n=13) on glaucoma and driving cessation with meta-analysis suitable for 2 studies

Additional Narrative Summaries:
Persons with bilateral glaucoma (OR 2.6 (95% Cl 1.4-4.8); p= 0.002) were more likely to stop driving but those with unilateral glaucoma were not (OR 1.5 (95% Cl 0.7-2.9); p= 0.3) with one Japanese study reporting individuals with severe POAG in the better eye to have an approximately 11.5 times greater odds of driving cessation than persons without POAG.

**Author and Year**	**Study Design**	**Total Participants (exposure/control)**	**Mean Age**	**Country**	**VI Definition**	**Comparator**	**Outcome Measure (OR, RR, HR?)**	**Effect Measure (with 95% Cl)**
**Included in Meta-analysis**								
Edwards J et al., 2008	Cross-sectional	1656 (152/1504)	72.95	USA	Self-reported physician diagnosed	Participants without glaucoma	HR	1.47 (0.98, 2.19); p=0.06
Gilhotra JS et al., 2001	Cross-sectional	3654 (61/3593)	65.9	Australia	Self-reported and physician diagnosed	Participants without glaucoma	OR	2.2 (1.3, 3.9)
**Included in Narrative Summaries Only – High Income Countries**								
Adler G et al., 2004	Cross-sectional	199 (52/147)	71.3	USA	Open-or closed-angle glaucoma	Participants without glaucoma	X^2 (Chi Square)	Drivers with glaucoma were no more likely than controls to have made plans for driving cessation; p=0.49
Edwards J et al., 2008	Cross-sectional	1656 (152/1504)	72.95	USA	Self-reported physician diagnosed	Participants without glaucoma	Prevalence (%)	8.6% (125/1450) of current drivers had glaucoma compared to 13.9% (28/199) of non-drivers with glaucoma.
Gilhotra JS et al., 2001	Cross-sectional	3654 (61/3593)	65.9	Australia	Open-angled	Participants without glaucoma	Prevalence (%)	2% (37/2379) of current drivers had glaucoma compared to 5% (24/451) of non-drivers with glaucoma.

Goh Y et al., 2011	Case Series	77 (77/0)	71.8	UK	Physician diagnosed	Participants with glaucoma and other ocular pathologies	OR	At clinic presentation: 4.99 (1.2, 20.6)* Glaucoma patients with other ocular pathologies were more likely to fail the driving criteria and give up driving than patients with only glaucoma.
								At last clinic visit: 4.37 (1.6, 11.8) Glaucoma patients with other ocular pathologies were more likely to fail the driving criteria and give up driving than patients with only glaucoma.
Kaleem MA et al., 2021	Cross-sectional	191 (191/0)	77	USA	Physician diagnosed	Drivers with glaucoma but with either better VA or CS.	Prevalence (%) and X^2 (Chi Square)	78% of participants reported that they had stopped driving.
								Participants with worse VA were more likely to stop driving ($p < 0.05$)
								Participants with worse CS were more likely to stop driving ($p < 0.01$).
MacLeod K et al., 2014	Cross-sectional	1279 (67/1211)	age, no.: 55-64 years = 233, 65-	USA	Self-reported physician diagnosed	Ex-drivers without glaucoma	RR (risk ratio)	1.3
							Attributable Risk	1.6

For peer review only

			74 years = 499, 75+ years = 547				Prevalence (%)	7.4% (6/79) of non-driving participants had glaucoma compared to 5.7% (5/79) who did not have glaucoma.
Marottoli RA et al., 1993	Cross-sectional	1331 (28/1303)	age, no.: 65-74 years = 484, 75-84 years = 105, 85+ years = 6	USA	Self-reported physician diagnosed	Participants without glaucoma	Prevalence (%)	From the 28 participants who reported glaucoma at baseline (1983), 42.9% (12/28) stopped driving by 198 compared to 22.2% (125/564) of people who did not have glaucoma and who also stopped driving.
Naredo Turrado J et al., 2020	Prospective cohort	11670 (525/11144)	62.4	France	Self-reported physician diagnosed	Participants without glaucoma	HR	1.6, p>0.05
Ramulu P et al., 2009	Cross-sectional	1135 (138/997)	79.7	USA	Bilateral or unilateral	Participants without glaucoma	OR	Bilateral: 2.6 (1.4, 4.8)<br><br>Stopped driving for over 8 years (bilateral): 3 (1.4, 6.4)*<br><br>Stopped driving less than 2 years ago (bilateral): 3.6 (1.5, 5.8)
								Unilateral: 1.5 (0.7, 2.9)<br><br>Stopped driving less than 2 years ago (unilateral): 2.4 (1, 6)
							Prevalence (%)	40.6% (28/68) of all participants with

For peer review only

								bilateral glaucoma were not driving. 21.4% (15/70) of all with unilateral glaucoma were not driving.<br>15% (150/997) of all without glaucoma were not driving.
Takahashi A et al., 2018	Prospective cohort	359 (211/148)	54	Japan	Mild POAG at baseline	Participants without glaucoma	OR	No association found (data not shown)
					Moderate POAG in the better eye at the 3 year follow-up			37.7 (3.7, 383.8)
					Severe POAG in the better eye at baseline			11.52 (2.87, 46.35)
					Severe POAG in the better eye at 3-year follow-up			52.8 (3.5, 797)
							Prevalence (%)	5.3% (8/152) of those with mild glaucoma were no longer driving.<br><br>21% (7/33) of those with moderate/severe glaucoma were no longer driving.<br><br>A total of 8.1% (15/185) of all participants with glaucoma were not driving compared to 1/3% (1/80) of drivers without glaucoma who

For peer review only

								were also no longer driving.
Tam A et al., 2018	Cross-sectional	99 (99/0)	71.5	Canada	Glaucoma severity was defined by the visual field mean deviation (MD) in the better eye and classified into 2 groups: mild (MD >−6 dB) and moderate/severe (MD ≤−6 dB), corrected visual acuity in the better eye ≥20/50	Mild/moderate glaucoma patients	Prevalence (%)	33% (15/46) of mild/moderate glaucoma reported driving cessation compared to 8% (4/53) of mild glaucoma patients; p= 0.002
vanLandingham et al., 2013	Cross-sectional	139 (81/58)	70.1	USA	Physician diagnosed	Glaucoma suspect controls	OR	4 (1.1, 4.7); p=0.03
							Prevalence (%)	22.5% (18/81) of participants with glaucoma were no longer driving.
**Included in Narrative Summaries Only – Low Middle Income Countries**								
Deshmukh AV et al., 2019	Case Control	150 (100/50)	64.5	India	Anderson criterion	Drivers without glaucoma	Prevalence (%)	16% (16/100) of those with glaucoma has stopped driving.

*unadjusted results

For peer review only

Table 4b(ii) All studies (n=4) on cataract and driving cessation, all suitable to be summarised narratively only

Additional Narrative Summaries:
One study with sex disaggregated analysis found male drivers to be 7.01 times more likely to stop driving compared to female drivers who only had a 3.67 odds of driving cessation. Only one study examined the impact of a diagnosis of wet AMD but did not find any significant associations.

Author and Year	Study Design	Total Participants (exposure/control)	Mean Age	Country	VI Definition	Comparator	Outcome Measure (OR, RR, HR?)	Effect Measure (with 95% CI)
**Included in Narrative Summaries Only – High Income Countries**								
MacLeod K et al., 2014	Cross-sectional	1279 (278/1001)	age, no.: 55-64 years = 233, 65-74 years = 499, 75+ years = 547	USA	Self-reported physician diagnosed	Ex-drivers without cataract.	RR (risk ratio)	1.5
							Attributable risk	10.5, p<0.1
							Prevalence (%)	8% (6/79) of participants with cataracts no longer drove compared to 5.2% (4/79) with no cataracts.
Marottoli RA et al., 1993	Cross-sectional	1331 (105/1226)	age, no.: 65-74 years = 484, 75-84 years = 105, 85+ years = 6	USA	Self-reported physician diagnosed	Current Drivers	OR	2.29 (1.28, 4.1)
							Prevalence (%)	45.7% (48/105) of participant with cataracts were no longer driving compared to 18.4% (90/488) of those who were no longer driving and did not have cataracts.
Naredo Turrado J et al., 2020	Prospective cohort	11670 (291/11379)	62.4	France	Self-reported physician diagnosed	Current drivers	HR	1.79, p>0.05
Sengupta S et al., 2014	Cross-sectional	122 (N/A)	72.4	USA	Physician diagnosed	Participants without cataract/PCSO in better eye.	PR (Prevalence Ratio)	Presence of cataract/PCO in the better seeing eye did not show any significant association

For peer review only

								with driving cessation; p>0.5

*unadjusted results

Table 4b(iii) All studies (n=5) on AMD and driving cessation with meta-analysis suitable for 3 studies

Author and Year	Study Design	Total Participants (exposure/control)	Mean Age	Country	VI Definition	Comparator	Outcome Measure (OR, RR, HR?)	Effect Measure (with 95% CI)
**Included in Meta-analysis**								
Campbell MK et al., 1993	Case Control	1656 (276/1380)	N/A	USA	Self-reported physician diagnosed	Current drivers	OR	4.25 (2.6, 7); p<0.001
Edwards J et al., 2008	Cross-sectional	1656 (89/1567)	72.95	USA	Self-reported physician diagnosed	Current drivers	HR	1.46 (0.91, 2.36); p=0.12
Stewart RB et al., 1993	Cross-sectional	1470 (N/A)	78.1	USA	Self-reported physician diagnosed	Current drivers	OR	3.32 (1.91, 5.77); p=0.0001
**Included in Narrative Summaries Only – High Income Countries**								
Campbell MK et al., 1993	Case Control	1656 (276/1380)	N/A	USA	Self-reported physician diagnosed	Current drivers	OR	**Male**: 7.01 (3.1, 15.9); p<0.001)*
								**Female**: 3.67 (2.0, 6.8), p<0.001*
							Prevalence (%)	5.06% (70/1379) of participants still driving had AMD compared to 17.88% (50/277) of non-drivers with AMD.
Edwards J et al., 2008	Cross-sectional	1656 (89/1567)	72.95	USA	Self-reported physician diagnosed	Current drivers	Prevalence (%)	4.9% (71/1457) of participant still driving had AMD compared to 9.5% (19/198) of non-driving participant with AMD.

For peer review only

MacLeod K et al., 2014	Cross-sectional	1279 (48/1231)	age, no.: 55-64 years = 233, 65-74 years = 499, 75+ years = 547	USA	Self-reported physician diagnosed	Ex-drivers without AMD.	RR (risk ratio)	2.3
							Attributable risk	4.5, p<0.01
							Prevalence (%)	12.7% (10/79) of ex-drivers had AMD compared to 5.6% (4/79) of ex-drivers without AMD.
Sengupta S et al., 2014	Cross-sectional	122 (64/58)	72.4	USA	Physician reported wet AMD	Participants without AMD	OR	**Any eye:** 1.9 (0.5, 7.3)
								**Worse eye:** 0.6 (0.1, 3.3)
								**Better eye:** 2.7 (0.6, 11.5)
							Prevalence (%)	74.6% (48/64) of participant with AMD were still driving compared.
								More participants in the AMD group (25.4%) had stopped driving compared to those without AMD (6.9%); p= 0.006
Stewart RB et al., 1993	Cross-sectional	1470 (N/A)	78.1	USA	Self-reported physician diagnosed	Current drivers	Prevalence (%)	59.8% (35/58) of participant with AMD were still driving.

*unadjusted results

For peer review only

Table 4b(iv) All studies (n=18) on visual acuity (VA) impairment and driving cessation, all suitable to only be summarised narratively due to their different VA cut-off points and comparators

Author and Year	Study Design	Total Participants (exposure/control)	Mean Age	Country	VI Definition	Comparator	Outcome Measure (OR, RR, HR?)	Effect Measure (with 95% CI)
**Included in Narrative Summaries Only – High Income Countries**								
Anstey K et al., 2006	Prospective Cohort	1466 (446/1020)	age no.: 70-74 years = 378, 75-79 years = 353, 80-84 years = 339, 85+ years = 396	Australia	Corrected distance VA at 3 metres in best eye at 6/12 or worse	Participants with VA better than 6/12 (i.e. better than 20/40)	OR	Visit 2: 1.91 (0.51, 7.13)<br>Visit 3: 1.84 (0.68, 4.99)<br>Visit 4: 1.15 (0.55, 2.41)
DeCarlo D et al., 2003	Cross-sectional	126 (N/A)	79	USA	Better eye	Current drivers	Mean (SD)	VA in the better eye was worse in non-drivers (1.03 +/- 0.39) than drivers (0.74 +/- 0.34).
					Worse eye			VA in the worse eye was worse in non-drivers (1.58 +/- 0.43) than drivers (1.18 +/- 0.42).

For peer review only

Edwards J et al., 2008	Cross-sectional	1656 (N/A)	72.95	USA	ETDRS chart with scores assigned from 0 to 90 (e.g. score of 0 = Snellen score of 20/125, score of 90 = Snellen score of 20/16)	Current drivers	HR (multivariate model)	0.91 (0.791, 1.046); p=0.184
							HR (cox regression)	0.69 (0.61, 0.78); p<0.001
Freeman E et al., 2005	Prospective cohort	1824 (263/1561)	73.4	USA	≥ 0.1 and <0.3 logMAR at baseline	<0.1 logMAR as baseline	HR	1.27 (0.96, 1.69)
		1824 (63/1498)			≥0.3 logMAR VA at baseline	<0.1 logMAR as baseline		1.23 (0.69, 2.18)
		1824 (329/1495)			1-2 lines VA loss	<1 line loss in VA		1.25 (0.96, 1.65)
		1824 (134/1690)			>2 lines VA loss	<1 line loss in VA		1.26 (0.87, 1.84)
Garre-Olmo J et al., 2009	Cross-sectional	875 (N/A)	81.7	Spain	Self-reported	Drivers without impaired VA	OR	0.379 (0.201, 0.714); p=0.003*
Gilhotra JS et al., 2001	Cross-sectional	3654 (80/3574)	65.9	Australia	BCVA worse than 6/12 in the better eye	Current drivers	OR	4 (2.5, 3.9)
		3654 (283/3371)			Presenting VA worse than 6/12 in the better eye			2.5 (1.9, 3.4)
							Prevalence (%)	11% (49/452) of participants have stopped driving have VA >20/40 compared to the 1% (21/2379) who are still driving with VA >20/40.
Huisingh C et al., 2016	Prospective Cohort	1995 (161/1834)	77.2	USA	logMar <0.3	Drivers without VA impairment	HR	0.83 (0.49, 1.42)
							Mean (SD)	VA of those who stopped driving (0.097 [0.15]) compared to those still driving (0.051 [0.13]).

For peer review only

							Prevalence (%)	90.9% (149/164) of those not driving had a VA of ≤20/40 compared to 9.2% (15/164) who stopped driving with a VA of >20/40.
Janz N et al., 2009	Prospective Cohort	607 (N/A)	age, no.: 25-49 years = 131, 50-64 years = 240, 65-74 years = 177	USA	Better eye at 6 months	Driving vs. non-drivers	2-sample t-test	Mean (SD) of VA in drivers (87.7 [4.9]) vs. non-drivers (85.1 [5.4]); p<0.001
							Linear regression	Mean (SD) of VA in drivers (87.7 [4.9]) vs. non-drivers (85.1 [5.4]); p=0.012
					Better eye at 54 months		2-sample t-test	Mean (SD) of VA in drivers (86.9 [5.7]) vs. Non-drivers (83.2 [6.9]); p= 0.025
							Linear regression	Mean (SD) of VA in drivers (86.9 [5.7]) vs. Non-drivers (83.2 [6.9]); p=0.458
					Mean (SD) difference in VA in better eye from 6 months to 54 months	Remained drivers vs. became non-drivers	Linear regression	Changes in Mean (SD) in VA of drivers (-0.4[0.6]) vs. became non-drivers (3.9[0.7]); p=0.001
					Worse eye at 6 months	Driving vs. non-drivers	2-sample t-test	Mean (SD) of VA in drivers (83.2 [7.5]) vs. non-drivers (79.7 [11.0]); p= 0.007
							Linear regression	Mean (SD) of VA in drivers (83.2 [7.5]) vs. non-drivers (79.7 [11.0]);

For peer review only

								p= 0.095
					Worse eye at 54 months		2-sample t-test	Mean (SD) of VA in drivers (81.5 [10.6]) vs. non-drivers (75.3 [14.4]); p=0.001
							Linear regression	Mean (SD) of VA in driver (81.5 [10.6]) vs. non-driver: 75.3 (14.4); p=0.003
					Mean (SD) difference in VA in worse eye from 6 months to 54 months	Remained drivers vs. became non-drivers	Linear regression	Mean (SD) of VA in drivers (1.4[1.3]) vs. became non-drivers: -5.5(2.1); p=0.054
Keay L et al., 2009	Prospective Study	1425 (N/A)	75	USA	LogMAR scale	Whole population	Mean (SD)	mean(SD) of VA statistically significant different between those who stopped driving 0.08 (0.014) and those who continued driving -0.01(0.11); p=0.0006
Keay et al., 2016	Cross-sectional	442 (N/A)	73	Australia	High contrast vision	Current drivers with cataracts NOTE: all participants had cataracts	OR	1.21 (1.07, 1.37)
					Binocular		X^2 (Chi Square)	p<0.001
					Better eye			p<0.001
					Worse Eye			p<0.001
Levecq L et al., 2013	Cross-sectional	1000 (N/A)	71.3	Belgium	Physician diagnosed binocular VA worse than 20/40	Current drivers	X^2 (Chi Square)	Right eye: Mean VA of current drivers (0.31) was significantly better than those who gave up driving due to vision (0.25); p=0.016
								Left eye: Mean VA in current drivers (0.31) was significantly

								better than those who gave up driving due to vision (0.24); p=0.004
								Both eyes:<br>Mean VA in current drivers (0.36)<br>Was significantly better than those who gave up driving due to vision (0.31); p=0.031
Ramulu P et al., 2009	Cross-sectional	1135 (N/A)	79.7	USA	Binocular acuity 0.1 logMAR or worse in better eye.	Drivers without 0.1 logMAR binocular.	OR	1.5, p<0.001
Ross L et al., 2009	Cross-sectional	5206 (1062/4144)	76.3	Australia	Physician diagnosed with participants categorised into having a VA LogMAR 0.3 or better, or worse than LogMar 0.3.	Participants with normal vision (logMAR of or better than 0.3).	OR	2.08 (2.56, 1.69)*
Rubin G et al., 2007	Prospective Cohort	2520 (N/A)	age, no.: 65-69 years = 780, 70-74 years = 829, 77-79 years = 553, 80-85 years = 350	USA	15 letter loss (logMAR 0.3)	Current Drivers	Prevalence (%)	Of those no longer driving: 84% (604/719) had VA ≤ 20/40 whilst 16% (115/719) had VA > 20/40.

For peer review only

Segal-Gidan F et al., 2010	Cross-sectional	421 (44/377)	72	USA	Mild vision impairment was defined at the BCVA in the better eye (20/40-20/63)	Current drivers	OR	5.53 (1.45, 20.98)
		421 (23/377)			Moderate/severe vision impairment was defined as BCVA in the better eye (20/80 or worse)			13.23 (1.45, 120.3)
Sengupta S et al., 2014	Cross-sectional	122 (N/A)	72.4	USA	Worse VA in the better eye (1 line loss of vision)	1 line worse in better eye acuity (logMAR) in all participants	OR	Low VA in either eye: 1.4 (1.1, 1.9); p<0.001 Low VA in better eye: 1.5 (1.2, 1.9); p<0.001
							Mean (SD)	Participants who had stopped driving (logMAR VA 0.77) had significantly worse vision in the better seeing eye than those still driving (LogMAR VA 0.08); p=0.001
Tam A et al., 2018	Cross-sectional	99 (N/A)	71.5	Canada	Physician diagnosed	N/A – looked at VA in whole population (all glaucoma patients)	X^2 (Chi Square)	Best corrected VA not associated with cessation; p=0.18
								Declines in central vision was significantly associated with driving cessation; p= 0.001
								Declines in near vision was significantly associated with driving cessation; p= 0.001
								Declines in peripheral vision was significantly associated

For peer review only

								with driving cessation; p= 0.001
vanLandingham S et al., 2013	Cross-sectional	139 (N/A)	70.1	USA	1 line worse in the better eye	Glaucoma suspect controls	OR	1.3 (1, 1.8); p<0.05
						Moderate VA loss in glaucoma cases		Severe VA loss: 1.5 (1.2, 1.8); p< 0.05

*unadjusted results

Table 4b(v) All studies (n=8) on contrast sensitivity (CS) impairment and driving cessation with 3 studies suitable for meta-analysis

Additional Narrative Summaries:
CS was measured either as a continuous measure, or categorised as “poor” according to normative cut-points, with one study using both measures.

**Author and Year**	**Study Design**	**Total Participants (exposure/control)**	**Mean Age**	**Country**	**VI Definition**	**Comparator**	**Outcome Measure (OR, RR, HR?)**	**Effect Measure (with 95% Cl)**
				**Included in Meta-analysis**				
Huisingh C et al., 2016	Prospective cohort	1995 (130/1865)	77.2	USA	<1.5 score on Pelli-Robson chart	Drivers with no bilateral CS impairment	HR	1.73 (1.1, 2.72)
							Mean (SD)	The mean log CS of current drivers was 1.68 (0.13) compared to 1.61 (0.16) in non-drivers.
							Prevalence (%)	5.8% (106/1831) of current drivers had a log CS <1.5, compared to 14.6% (24/164) who stopped driving.
Keay L et al., 2009	Prospective cohort	1425 (N/A)	75	USA	Per letter lost	Drivers with no bilateral CS impairment	OR	1.15 (1.03, 1.28)*
					Better eye CS		Mean (SD)	CS in better eye of those who stopped driving 32.4(4.1) significantly different between those who continued driving 35.3(2.2); p<0.001
		122 (N/A)	72.4	USA			OR	1.36 (1.1, 1.7); p<0.05

For peer review only

Sengupta S et al., 2014	Cross-sectional				Binocular CS 1 letter worse	Drivers with no bilateral CS impairment	X^2 (Chi Square)	Those who stopped driving had significantly worse CS (log CS 1.8) compared to those still driving (log CS 1.2); p=0.03
**Included in Narrative Summaries Only – High Income Countries**								
Freeman E et al., 2005	Prospective cohort	1824 (725/1099)	73.4	USA	>=32 and <36 letters CS at baseline	Baseline CS equal to or more than 36 letters.	HR	1.26 (0.97, 1.63)
		1824 (158/1666)			<32 letters at baseline			1.46 (0.98, 2.17)
		1824 (79/1725)			5 letter CS loss in 2 years	Less than 5 letter CS loss		1.33 (0.8, 2.22)
		1824 (86/1738)			>= 6 letter CS loss in 2 years			1.71 (1.01, 2.9)
Keay L et al., 2016	Cross-sectional	442 (N/A)	73	Australia	0.12 log units drop in CS.	Cataract patients who are still driving	OR	1.29 (1.11, 1.49)
							Prevalence (%)	17% (45/263) of current drivers and 35% (37/110) of former drivers had a CS <1 log decrease by at follow-up; p< 0.001
							Mean (SD)	The worse eye CS in current drivers was 1.27 (+/- 0.36) compared to 1.11 (+/- 0.41) in former drivers; p< 0.001
Ramulu P et al., 2009	Cross-sectional	1135 (N/A)	79.7	USA	5 letters worse in better eye	Current drivers without 5 letters worse in better eye CS.	OR	3, p<0.001
Rubin G et al., 2007	Prospective Cohort	2520 (N/A)	Age, no,: 65-69 years =	USA	Log CS ≥ 1.65	Current drivers	Prevalence (%)	49.1% (884/1801) participants had stopped driving.

For peer review only

			780, 70-74 years = 829, 77-79 years = 553, 80-85 years = 350		Log CS 1.35-1.65			54% (973/1801) had stopped driving.
					Log CS <1.35			96.9% (1745/1801) had stopped driving.
vanLandingham S et al., 2013	Cross-sectional	139 (N/A)	70.1	USA	Binocular CS 1 letter worse	Glaucoma suspect controls	OR	1.3 (1.2, 1.4); p<0.05

*unadjusted results

Table 4b(vi) All studies (n=8) on visual field (VF) impairment and driving cessation, all suitable to only be summarised narratively due to their different VF cut-off points and comparators

**Author and Year**	**Study Design**	**Total Participants (exposure/control)**	**Mean Age**	**Country**	**VI Definition**	**Comparator**	**Outcome Measure (OR, RR, HR?)**	**Effect Measure (with 95% CI)**
**Included in Narrative Summaries Only – High Income Countries**								
Freeman E et al., 2005	Prospective Cohort	1824 (659/1165)	73.4	USA	>1 and <= 8 points of central visual field at baseline	Equal to or greater than 1 points missed at baseline central VF	HR	1.34 (1.02, 1.76)
		1824 (174/1650)			>9 points of central visual field at baseline	Equal to or greater than 1 points missed at baseline central VF		1.81 (1.23, 2.66)
		1824 (65/1759)			5-7 points of central visual field loss in 2 years	<5 central VF loss		1.01 (0.6, 1.72)

For peer review only

		1824 (92/1732)			>=8 points of central visual field loss in 2 years	<5 central VF loss		0.83 (0.53, 1.29)
		1824 (632/1192)			>9 and <=18 points of peripheral visual field at baseline	Less than or equal to 9 points missed at baseline peripheral VF		1.51 (1.14, 1.98)
		1824 (180/1644)			>18 points of peripheral visual field at baseline	Less than or equal to 9 points missed at baseline peripheral VF		1.73 (1.14, 1.98)
		1824 (106/1718)			6-7 points of peripheral visual field loss in 2 years	<6 points loss of peripheral VF		1.04 (0.65, 1.65)
		1824 (88/1736)			>= 8 points of peripheral visual field loss in 2 years	<6 points loss of peripheral VF		1.91 (1.23, 2.96)
Huisingh C et al., 2016	Prospective cohort	1995 (493/1502)	77.2	USA	sensitivity <=22.5 dB	Participants without VF impairment	HR	1.78 (1.29, 2.46)
Janz N et al., 2009	Prospective cohort	607 (N/A)	age, no.: 25-49 years = 131, 50-64 years = 240, 65-74 years = 187	USA	Better eye at 6 months	Drivers vs. non-drivers	2-sample t-test	Mean (SD) MD of drivers (-2.1 [2.7]), vs. non-drivers (-2.9 [3.0]); p=0.014
							Liner regression	Mean (SD) MD of drivers (-2.1 [2.7]) vs. non-drivers (-2.9 [3.0]); p=0.966
					Better eye at 54 months		2-sample t-test	Mean (SD) MD of drivers (-1.9[3.1]) vs. non-drivers (-3.5 [3.7]); p<0.001

For peer review only

							Linear regression	Mean (SD) MD of drivers (-1.9[3.1]) vs. non-driver: -(3.5[3.7]); p= 0.007
					Mean (SD) difference in VA in better eye from 6 months to 54 months	Remain drivers vs. became non-drivers	Linear regression	Difference in mean (SD) MD of drivers (0.2 [2.1]) vs. became non-drivers (-0.7 [2.7]); p=0.008
					Worse eye at 6 months	Drivers vs. non-drivers	2-sample t-test	Mean (SD) MD of drivers (-5.7 [4.9]) vs. non-drivers (-5.9 [4.0]); p=0.014
							Liner regression	Mean (SD) MD of drivers (-5.7 [4.9]) vs. non-drivers (-5.9 [4.0]); p=0.429
					Worse eye at 54 months		2-sample t-test	Mean (SD) MD of drivers (-5.4 [5.2]) vs. non-drivers (-7.0 [4.9]); p=0.012
							Linear regression	Mean (SD) MD of drivers (-5.4 [5.2]) vs. non-drivers (-7.0 [4.9]); p=0.080
					Mean (SD) difference in VA in worse eye from 6 months to 54 months	Remain drivers vs. became non-drivers	Linear regression	Difference in mean (SD) MD of drivers (0.3 [0.4]), vs. became non-drivers (-1.3 [0.7]); p= 0.013
Keay L et al., 2009	Prospective Cohort	1425 (N/A)	75	USA	Bilateral VF points missing	Whole population	Mean (SD)	Mean(SD) of bilateral VF points missing was statistically

For peer review only

								significant different between those who stopped driving 9.8(17.1) and those who continued driving 1.98(5.1); p=0.001
Keay L et al., 2016	Cross-sectional	442 (N/A)	73	Australia	Points missed on bilateral VF.	Current drivers with cataracts NOTE: all participants in this study had cataracts.	X^2 (Chi Square)	Median (IQR) of current drivers: 3 (0-10) vs. Median (IQR) of former drivers: 8 (1-19); p= 0.02
Ramulu P et al., 2009	Cross-sectional	1135 (N/A)	79.7	USA	Bilateral VF damage in glaucoma participants	Participants without glaucoma	OR	2 (1.6, 2.5)
							Prevalence (%)	21% (14/68) of participants with bilateral VF loss in the lowest tertile (less than 3 dB of VF loss in better-eye) had stopped driving.<br><br>36% (24/68) of participants with VF loss in the middle tertile (better-eye VF mean deviation between −3 and −9 dB) had stopped driving.<br><br>52% (35/68) of participants with VF loss in the highest tertile (better eye VF mean deviation <-9 dB) had stopped driving.
Segal-Gidan F et al., 2010	Cross-sectional	421 (30/391)	72	USA	Unilateral	Current drivers	OR	1.91 (0.63, 5.76)
		421 (108/318)			Bilateral, mild			2.05 (0.74, 5.66)

For peer review only

		421 (93/328)			Bilateral, moderate/severe			2.84 (0.92, 8.78)
vanLandingham S et al., 2013	Cross-sectional	139 (N/A)	70.1	USA	5 dB worse in the better eye	Glaucoma suspect controls	OR	1.7 (1.1, 2.5); p= 0.008
**NOTE:** There are a range of different study designs as well as cut-off points and areas of VF investigated in the identified studies. Due to methodological differences between each study, meta-analysis was limited and narrative reviews have been used instead to synthesise data.								

*unadjusted results

For peer review only

Table 4b(vii) All studies (n=3) on glare sensitivity (GS) impairment and driving cessation, all suitable to only be summarised narratively due to their different GS cut-off points and comparators

**Author and Year**	**Study Design**	**Total Participants (exposure/control)**	**Mean Age**	**Country**	**VI Definition**	**Comparator**	**Outcome Measure (OR, RR, HR?)**	**Effect Measure (with 95% CI)**
**Included in Narrative Summaries Only – High Income Countries**								
Freeman E et al., 2005	Prospective Cohort	1824 (702/1122)	73.4	USA	3-4 points of glare sensitivity at baseline	≤2 points difference with baseline glare GS	HR	0.78 (0.61, 0.99)
		1824 (206/1618)			≥5 points of glare sensitivity at baseline			0.9 (0.63, 1.28)
		1824 (71/1753)			4 points loss of glares sensitivity in 2 years	<4 points GS loss		1.18 (0.7, 1.99)
		1824 (52/1772)			≥5 points loss of glare sensitivity in 2 years	<4 points GS loss		1.3 (0.72, 2.37)
Gilhotra JS et al., 2001	Cross-sectional	3654 (969/2685)	65.9	Australia	Physician diagnosed	Participants still driving	OR (logistic regression)	1.5 (1.2, 1.8)
Tam A et al., 2018	Cross-sectional	99 (15/84)	71.5	Canada	Physician diagnosed	Still driving participants with cataracts	Prevalence ratio (PR)	4.79; p<0.013

*unadjusted results

Table 4b(viii) All studies (n=11) on other types of vision impairment and driving cessation, all suitable to be summarised narratively only

Author and Year	Study Design	Total Participants (exposure/control)	Mean Age	Country	Type of VI	VI Definition	Comparator	Outcome Measure (OR, RR, HR?)	Effect Measure (with 95% CI)
**Included in Narrative Summaries Only – High Income Countries**									
Campbell MK et al., 1993	Case control	1656 (28/1628)	N/A	USA	Retinal detachment	Self-reported physician diagnosed	Current drivers	Prevalence (%)	Still driving = 14.25%. Not driving = 40.95% Those not driving have a higher percentage of detached retina than those still driving (p<0.05)
					Retinal haemorrhage	Self-reported physician diagnosed		OR	Both genders = 3.86 (1.4, 10.4)*
									Females: 4.70 (1.2, 17.8); p<0.5
					Vision impairment	Self-reported		Prevalence (%)	Still driving = 13.65%. Not driving = 25.34% Those not driving have a higher percentage of other visual loss than those still driving (p<0.01)
DeCarlo D et al., 2003	Cross-sectional	126 (126/0)	79	USA	Maculopathy	exudative or non-exudative	Current drivers	Prevalence (%)	The type of AMD (exudative vs nonexudative) was not significant between the non-drivers and drivers (p=0.474). Nonexudative non-drivers: 50% (48/96), nonexudative drivers : 47% (14/30), exudative non-

For peer review only

									drivers: 50% (48/96), exudative drivers 53% (16/30).
Hajek A et al., 2019	Cross-sectional	549 (192/357)	90.3	Germany	Vision impairment	Severe impairment	Current drivers	OR	0.06 (0.01, 0.59)*
						Mild impairment			0.56 (0.24, 1.35)*
Gallo JJ et al., 1999	Case Control	1920 (N/A)	N/A	USA	Vision impairment	Self-reported	Current drivers	OR	1.86 (0.7, 4.9)
Keay et al., 2016	Cross-sectional	442 (148/294)	73	Australia	URE	Measured with autorefraction and lensometry	Cataract patients who are still driving	X^2 (Chi Square)	No significant differences between current drivers with URE (40% [99/263]) and former drivers with URE (51% [49/110]); p= 0.07
Levecq L et al., 2013	Cross-sectional	1000 (346/654)	71.3	Belgium	Vision impairment	Physician diagnosed	N/A	Prevalence (%)	Among the 190 non-drivers, 47 (24.7%) stopped driving because of their impaired vision.
Marottoli RA et al., 1993	Cross-sectional	1331 (17/1314)	age, no.: 65-74 years = 484, 75-84 years = 105, 85+ years = 6	USA	Vision impairment	Self-reported	Current drivers	Prevalence (%)	Out of the 17 drivers who reported poor vision at baseline (1983), 58.8% (9/17) of drivers who stopped driving by 1989.
Moon SH et a., 2020	Cross-sectional	2970 (1023/1947)	71	South Korea	Vision impairment	Self-reported	Current drivers	OR	0.97 (0.83, 1.14)*
Robinson JL et a., 2021	Cross-sectional	335 (N/A)	67.4	USA	Vision impairment	Self-reported	Current drivers	X^2 (Chi Square)	Participants were less likely to be driving if they had noted vision-related concerns (p<0.001).

For peer review only

Tam A et al., 2018	Cross-sectional	99 (19/80)	71.5	Canada	Dark adaptation in glaucoma patients	Self-reported	Among patients with glaucoma	X^2 (Chi Square)	Dark adaptation significantly associated with driving cessation (p<0.001)
								PR (Prevalence Ratio)	1.47; p= 0.39 Individuals with self-perceived dark adaptation difficulties were not more likely to quit driving.
Zebardast N et al., 2015	Cross-sectional	2469 (132/2337)	73.5	USA	URE	Binocular presenting visual acuity of 20/30 or worse, improving to better than 20/30 with subjective refraction	Participants with normal vision.	OR	2.1 (1.3, 3.6)
					Non-refractive visual impairment	Post-refraction binocular BCVA of 20/30 or worse			3.7 (2.4, 5.7)

*unadjusted results

Table 4b(ix) All studies (n=2; reporting on 4 RCTs in total) evaluating anti-VEGF therapy and driving cessation, suitable for narrative summaries only

Author and Year	Study Design	Intervention (n)	Control (n)	Mean Age	Country	Vision Impairment	VI Definition	Comparator(s)	Outcome Measure	Effect measure (with 95% where appropriate)
Bressler N et al., 2013	RCT	478	238	77.7	USA	AMD	MARINA trial: minimally	Sham injections or 0.3 mg of Ranibizumab or 0.5 mg of	Prevalence (%) + 95% CI	Among patients who had reported driving at

For peer review only

							classic or occult AMD	Ranibizuman for 24 months		baseline, 74% (146/197) sham patients and 87.8% (156/178) 0.5mg patients reported still driving at 12 months. Among patients who had reported driving at baseline, 67.2% (131/195) (95% CI 59.2-75.2) of sham patients and 78.4% (148/189) (95% CI 71.8-85.0) of 0.5mg ranibizumab patients reported still driving 24 months later.

For peer review only

Bressler N et al., 2013	RCT	280	143	77.7	USA	AMD	ANCHOR: classic neovascular AMD	Verteporfin photodynamic therapy (PDT) or 0.3 mg ranibizumab injections or 0.5 mg ranibizumab injections for 24 months	Prevalence (%) + 95% CI	Among patients who reported driving at baseline, 80.5% (77/96) PDT patients and 94.2% (86/91) 0.5 mg patients reported still driving at 12 months. Among patients who reported driving at baseline, 71.6% (67/94) (95% CI 60.8-82.4) of PDT patients and 91.4% (81/89) (95% CI 85.3-97.5) of 0.5 mg ranibizumab patients reported still driving 24 months later.

For peer review only

Bressler N et al., 2016	RCT	502	257	62.3	USA	DME	RIDE/RISE: any DME	Sham injections or 0.3 mg ranibizumab or 0.5 mg ranibizumab	Prevalence (%) + 95% CI	For 0.3 mg ranibizumab compared to those treated with sham only, there was a 7% (-5.0 to 19) difference in the number of participants now driving (who were not driving at baseline) at 12 months. For 0.5 mg ranibizumab compared to those treated with sham only, there was a 14.4% (1.1, 27.7) difference in the number of participants now driving at 12 months. For 0.3 mg ranibizumab

For peer review only

										compared to those treated with sham only, there was a 12.5% (-0.9, 25.9) difference in the number of participants now driving at 24 months. For 0.5 mg ranibizumab compared to those treated with sham only, there was a 14.3% (0.7, 27.9) difference in the number of participants now driving at 24 months.

For peer review only

Bressler N et al., 2016	RCT	234	111	62.3	USA	DME	RESTORE: DME in a least 1 eye eligible for laser treatment and a VA letter score between 78 and 39	PDT laser only or 0.5 mg + laser or 0.5 mg only	Prevalence (%) with 95% CI	After 12 months, 12.2% (6/49) of those who were not driving at baseline and were treated with 0.5 mg ranibizumab + laser have started driving. Compared to those treated with laser only, there was a 4.2% (-7.7, 16.1) difference in the number of participants now driving at 12 months. After 12 months, 8.9% (4/45) of those who were not driving at baseline and were treated

For peer review only

										with 0.5 mg ranibizumab only have started driving. Compared to those treated with laser only, there was a 0.9% (-10.3, 12.1) difference in the number of participants now driving at 12 months.

For peer review only

Table 4b(x) All studies (n=2) evaluating cataract surgery and driving cessation, suitable for narrative summaries only

Author and Year	Study Design	Total Participants (exposure/control)	Mean Age	Country	VI Definition	Comparator	Outcome Measure (OR, RR, HR?)	Effect Measure (with 95% CI)
Monestam E et al., 2005	Prospective Cohort	810 (N/A)	74.7	Sweden	Physician diagnosed cataracts	All cataract surgery patients, comparing pre and post cataract surgery outcomes.	Prevalence (%)	Before cataract surgery, 55% (224/407) were drivers while after surgery 70% (285/407) were drivers. 5 years after surgery 63% (189/300) of patients with a driving licence were still active drivers. 37% (67/183) of patients who did not drive before surgery started to drive after. 46% (31/67) of patients who did not fulfil the visual requirements for presenting VA and the 35% (24/67) who did not fulfil the requirements for BCVA for a

For peer review only

								legal licence could now legally drive. 82% (40/50) of patents who began to drive after the surgery were still driving 5 years later.
Monestam E et al., 1997	Prospective cohort	211 (N/A)	41	Sweden	Physician diagnosed cataracts	Driving status from all participants pre- and post-surgery.	Ratio (%)	The number of patients driving after surgery increased to 65% (137/211) (from 56%), but this was not significant.

Table 4b(xi) All studies (n=1) evaluating anti-glaucoma therapy and driving cessation

**Author and Year**	**Study Design**	**Total Participants (exposure/control)**	**Mean Age**	**Country**	**Vision impairment**	**VI Definition**	**Comparator**	**Outcome Measure (OR, RR, HR?)**	**Effect Measure (with 95% CI)**
Stafford WR, 1981	Cross-sectional	240 (N/A)	age, no.: 35-49 years = 11, 50-65 years = 77, >65 years = 139	USA	Glaucoma	Chronic open-angle glaucoma or ocular hypertension that has been adequately controlled for at least	Post- anti-glaucoma therapy outcomes in all participants.	Prevalence (%)	From the 229 patients who stated that the anti-glaucoma therapy side effects affected their

For peer review only

						the previous 6 months			normal activity, 12% (28/229) said that they had to give up some normal activity. Out of the 28 patients, 16 mentioned giving up driving, particularly at night.

For peer review only

Appendix 5a Associations between MVC involvement and vision impairment and vision-related intervention

<table>
<tr><th>Vision impairment</th><th>Outcome</th><th>Association</th></tr>
<tr><td>Glaucoma</td><td>Any MVC</td><td>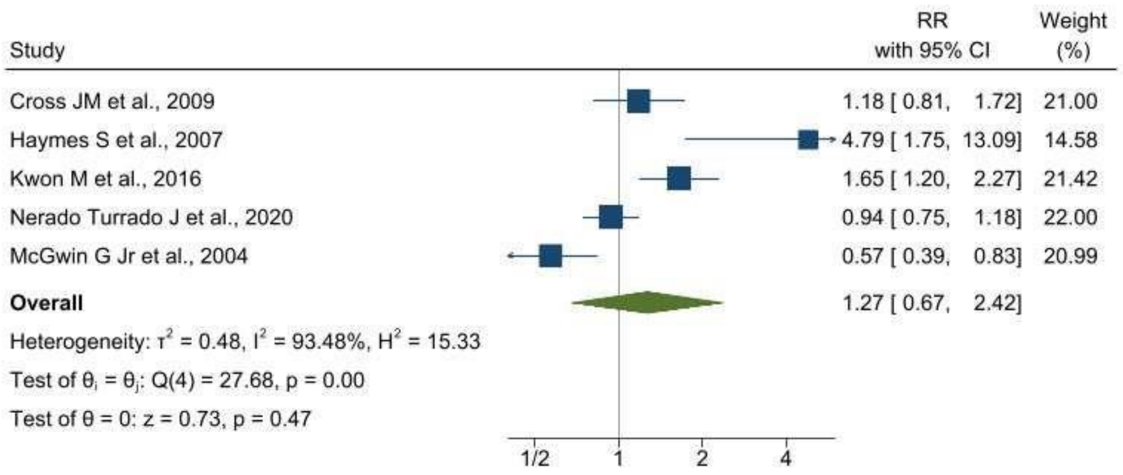
</td></tr>
<tr><td></td><td>At-fault MVC</td><td>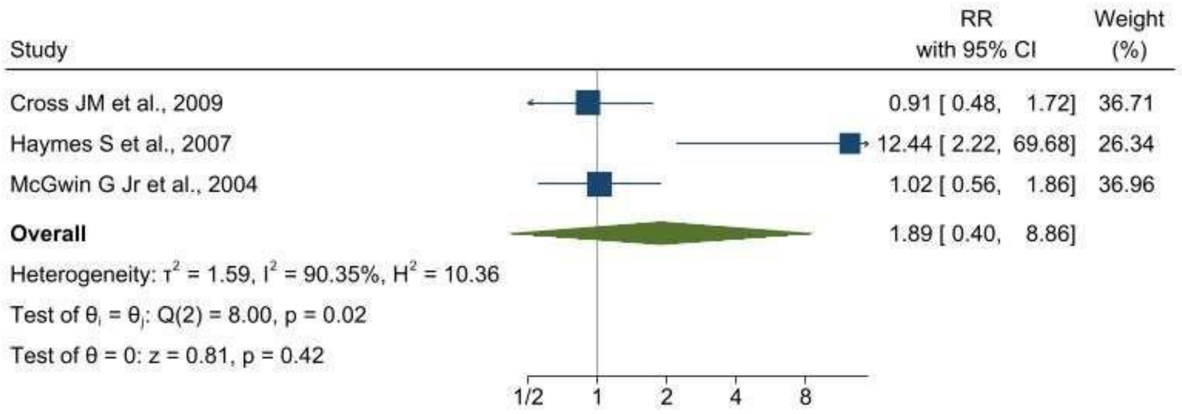
</td></tr>
</table>

**Cataract**	**Any MVC**	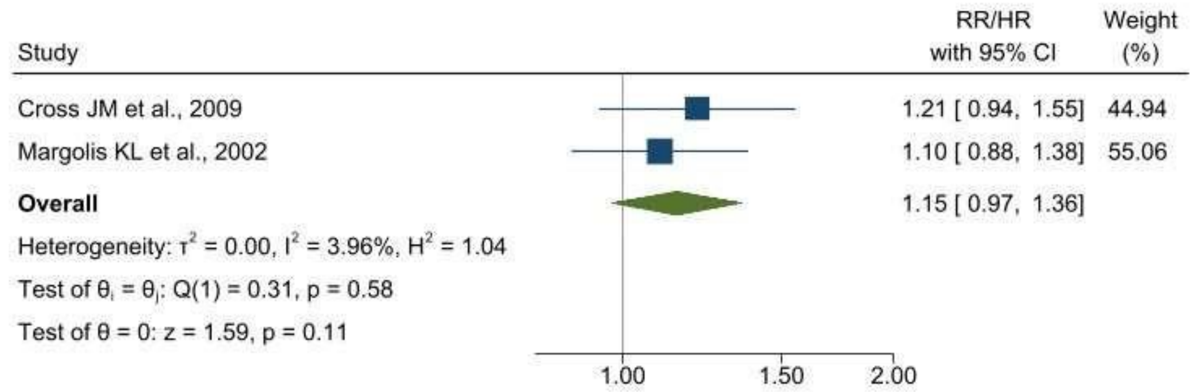 NOTE: POOLED ONE STUDY WITH RR AND ANOTHER WITH HR
**Stereopsis**	**Any MVC**	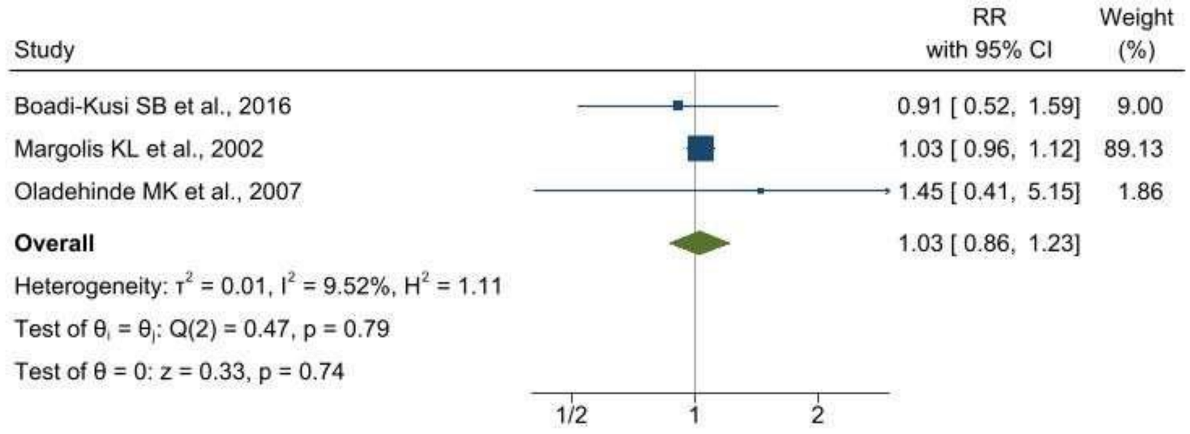

**Myopia**	**Any MVC**	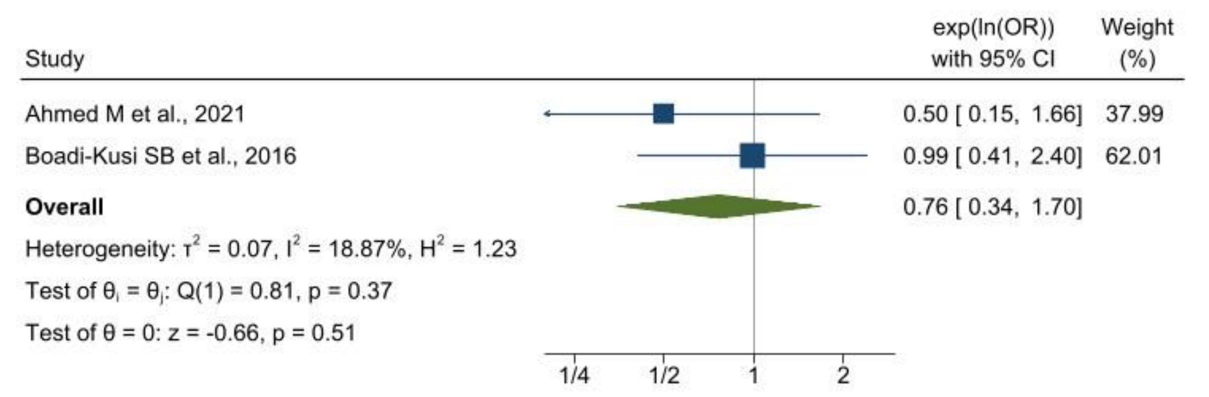

For peer review only

**VA ≤ 20/40**	**Any MVC**	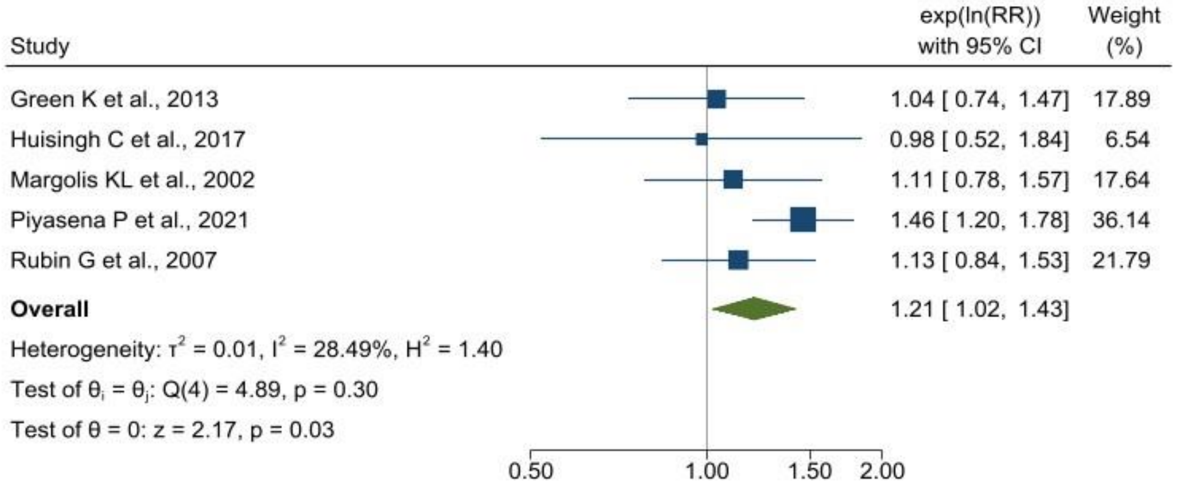
	**At-fault MVC**	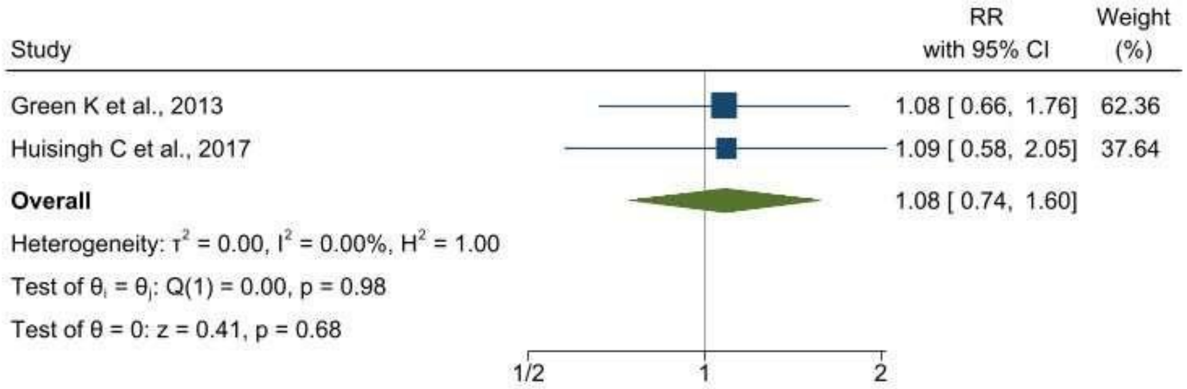

**Contrast Sensitivity Loss**	**Any MVC**	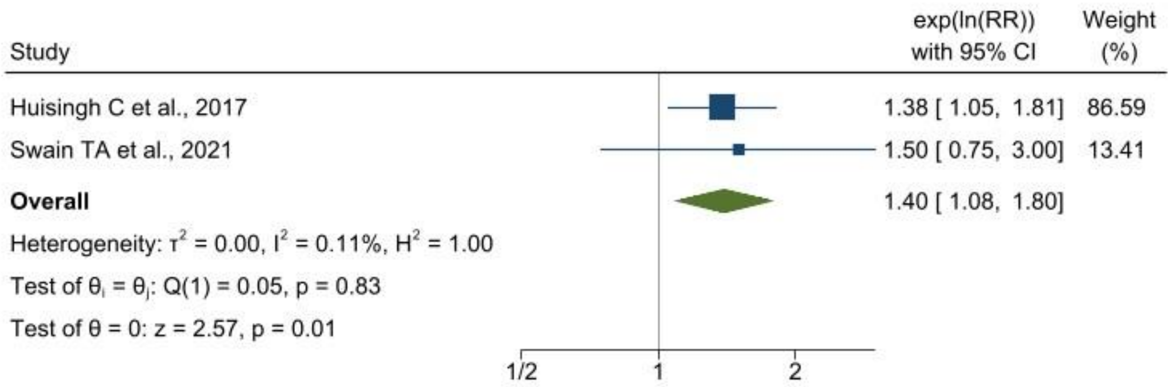
**Visual Field Loss**	**Any MVC**	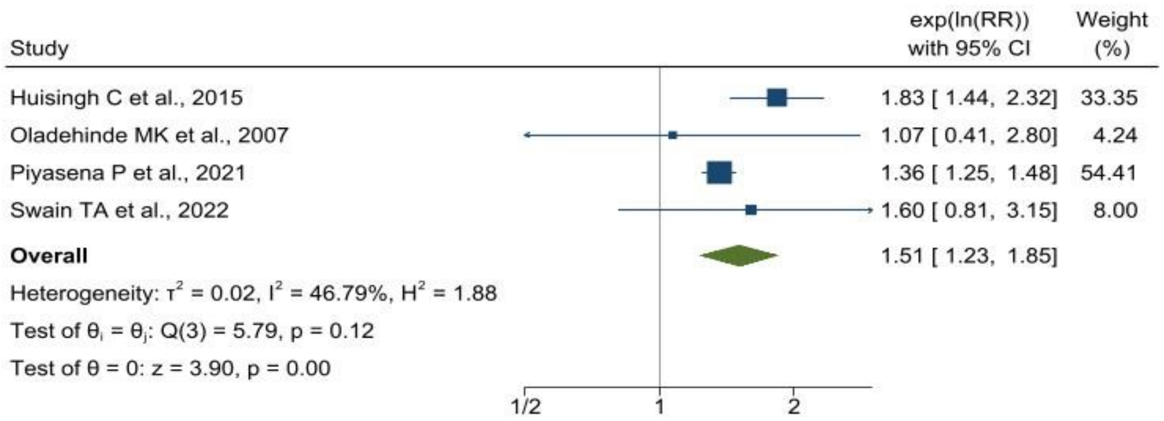
**Vision-related Intervention**	**Outcome**	**Association**

<table>
<tr><th>Cataract Surgery</th><th>Any MVC</th><th>
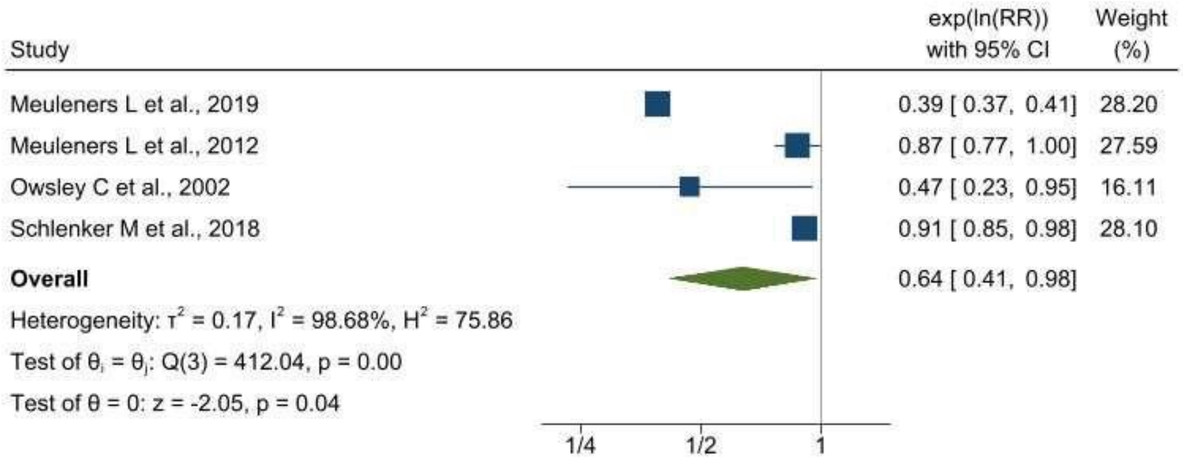

HOWEVER WHEN DROPPING SCHLENKER M ET AL., 2018 (UNADJUSTED EFFECT MEASURE):

Overall RR 0.55 [0.35, 0.92]
Heterogeneity: $T^2 = 0.17$, $I^2 = 97.10\%\%$, $H^2 = 34.52$
Test of $\theta_i = \theta_j$: Q(2) = 125.66, p = 0.00
Test of $\theta = 0$: z = -2.30, p = 0.02
</th></tr>
</table>

Appendix 5b Associations between driving cessation and vision impairments

Vision impairment	Association
**Glaucoma**	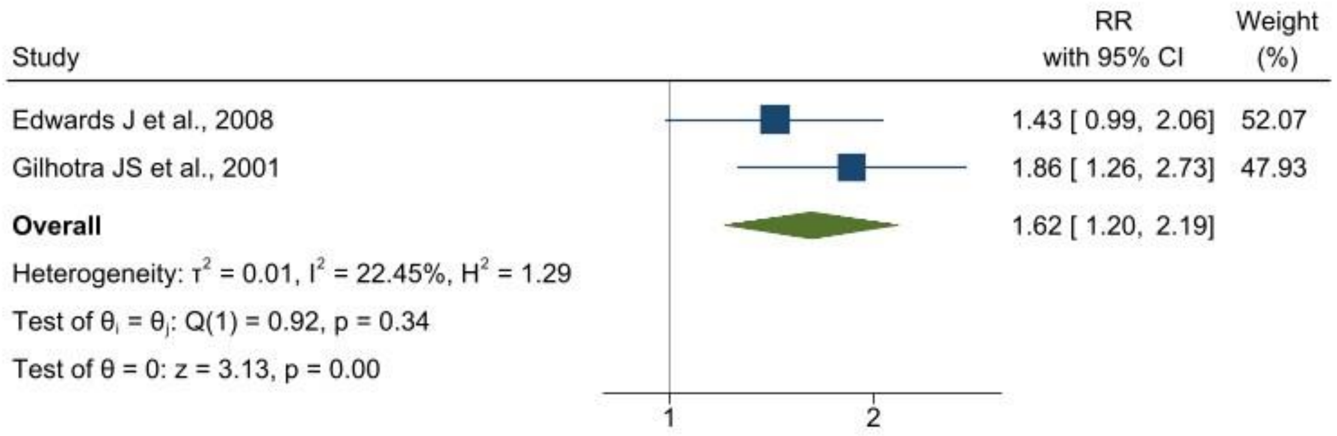
**AMD**	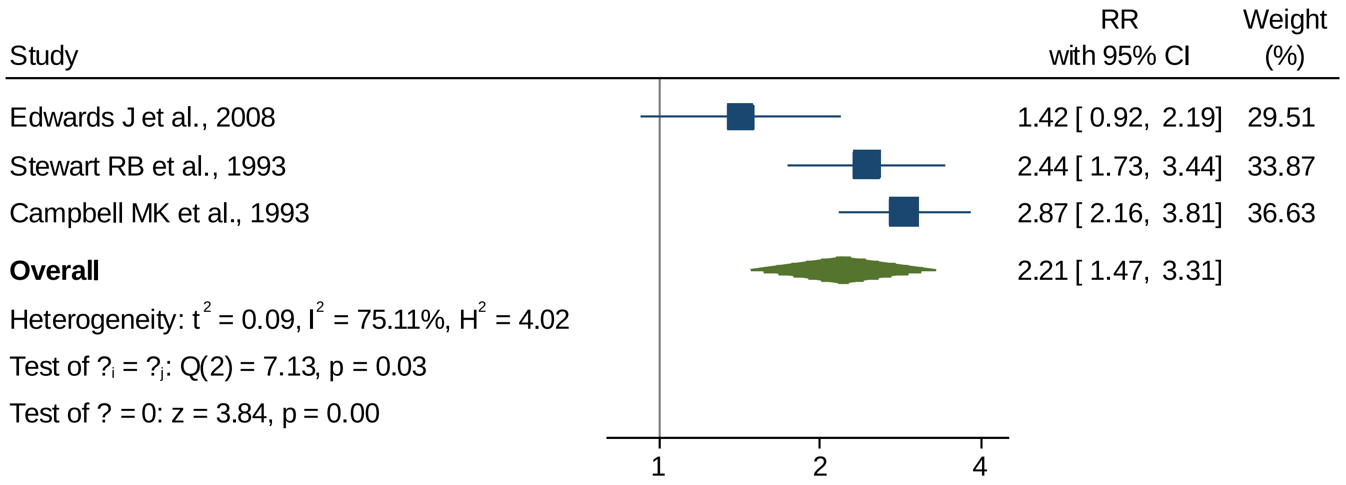
**Contrast Sensitivity**	

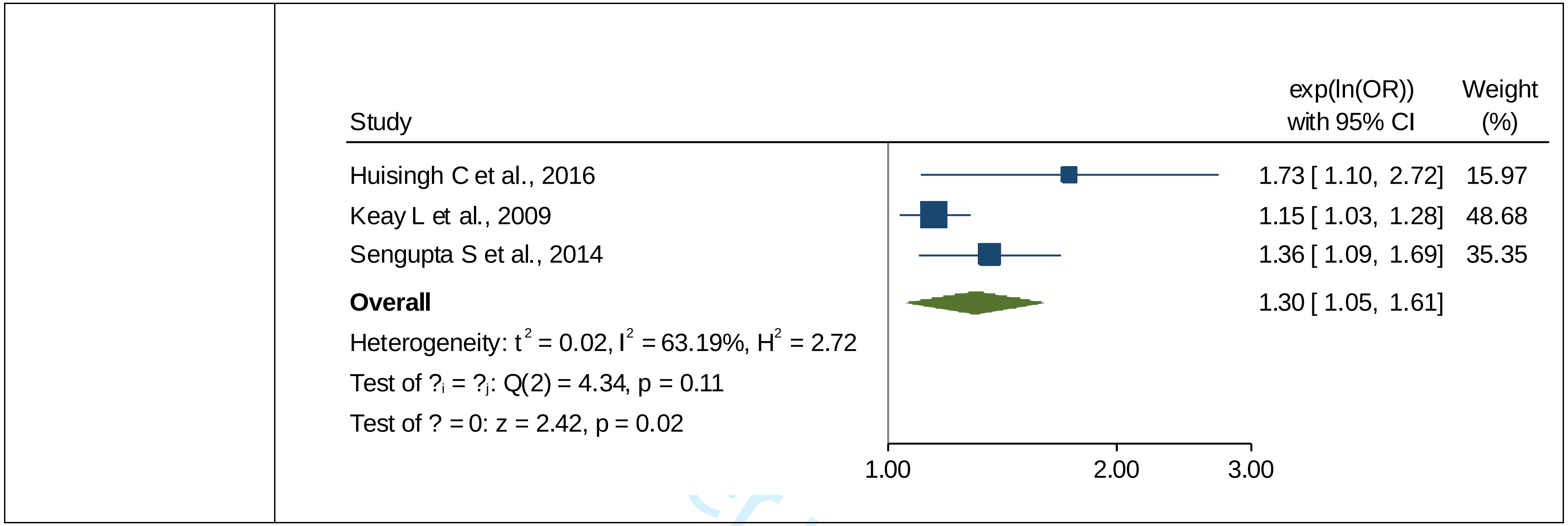

For peer review only - http://bmjopen.bmj.com/site/about/guidelines.xhtml

For peer review only

BMJ Open

Associations between vision impairment and vision-related interventions on crash risk and driving cessation: systematic review and meta-analysis

Journal:	*BMJ Open*
Manuscript ID	bmjopen-2022-065210.R2
Article Type:	Original research
Date Submitted by the Author:	28-Jul-2023
Complete List of Authors:	Nguyen, Helen; University of New South Wales School of Optometry and Vision Science<br>Di Tanna, Gian Luca; George Institute for Global Health; University of Applied Sciences and Arts of Southern Switzerland<br>Coxon, Kristy; Western Sydney University - Campbelltown Campus; Western Sydney University Translational Health Research Institute,<br>Brown, Julie; The George Institute for Global Health Injury Division<br>Ren, Kerrie; University of New South Wales School of Optometry and Vision Science<br>Ramke, Jacqueline; London School of Hygiene & Tropical Medicine,<br>Burton, Matthew J; London School of Hygiene & Tropical Medicine<br>Gordon, Iris; London School of Hygiene & Tropical Medicine<br>Zhang, Justine; London School of Hygiene and Tropical Medicine Faculty of Infectious and Tropical Diseases, International Centre for Eye Health, Clinical Research Department<br>Furtado, João; Universidade de São Paulo Faculdade de Medicina de Ribeirão Preto, Division of Ophthalmology<br>Mdala, Shaffi; Queen Elizabeth Central Hospital, Opthalmology Department<br>Kitema, Gatera Fiston; University of Rwanda College of Medicine and Health Sciences, Opthalmology Department<br>Keay, Lisa; University of New South Wales, School of Optometry and Vision Science; The George Institute for Global Health
**Primary Subject Heading**:	Ophthalmology
Secondary Subject Heading:	Public health
Keywords:	PUBLIC HEALTH, OPHTHALMOLOGY, EPIDEMIOLOGY

SCHOLARONE™
Manuscripts

I, the Submitting Author has the right to grant and does grant on behalf of all authors of the Work (as defined in the below author licence), an exclusive licence and/or a non-exclusive licence for contributions from authors who are: i) UK Crown employees; ii) where BMJ has agreed a CC-BY licence shall apply, and/or iii) in accordance with the terms applicable for US Federal Government officers or employees acting as part of their official duties; on a worldwide, perpetual, irrevocable, royalty-free basis to BMJ Publishing Group Ltd ("BMJ") its licensees and where the relevant Journal is co-owned by BMJ to the co-owners of the Journal, to publish the Work in this journal and any other BMJ products and to exploit all rights, as set out in our licence.

The Submitting Author accepts and understands that any supply made under these terms is made by BMJ to the Submitting Author unless you are acting as an employee on behalf of your employer or a postgraduate student of an affiliated institution which is paying any applicable article publishing charge ("APC") for Open Access articles. Where the Submitting Author wishes to make the Work available on an Open Access basis (and intends to pay the relevant APC), the terms of reuse of such Open Access shall be governed by a Creative Commons licence – details of these licences and which Creative Commons licence will apply to this Work are set out in our licence referred to above.

Other than as permitted in any relevant BMJ Author's Self Archiving Policies, I confirm this Work has not been accepted for publication elsewhere, is not being considered for publication elsewhere and does not duplicate material already published. I confirm all authors consent to publication of this Work and authorise the granting of this licence.

For peer review only

Associations between vision impairment and vision-related interventions on crash risk and driving cessation: systematic review and meta-analysis

Authors: Helen Nguyen BMedSci (Hons)[1], Gian Luca Di Tanna PhD[2], Kristy Coxon PhD[3], Julie Brown PhD[2], Kerrie Ren MIPH[1], Jacqueline Ramke PhD[4,5], Matthew J Burton PhD[4,6], Iris Gordon MSc[4], Justine H Zhang MBBS[4,7], João M. Furtado PhD[8], Shaffi Mdala MSc [9], Gatera Fiston Kitema MScPHEC[10], Lisa Keay PhD[1,2]

Affiliations:

1. School of Optometry and Vision Science, Faculty of Science, The University of New South Wales, NSW, Australia

2. The George Institute for Global Health, Faculty of Medicine, University of New South Wales, NSW, Australia

3. School of Health Sciences, and the Translational Health Research Institute, Western Sydney University, NSW, Australia

4. International Centre for Eye Health, London School of Hygiene & Tropical Medicine, London, United Kingdom, WC1 E7HT

5. School of Optometry and Vision Science, University of Auckland, Auckland, New Zealand

6. National Institute for Health Research Biomedical Research Centre for Ophthalmology at Moorfields Eye Hospital NHS Foundation Trust and UCL Institute of Ophthalmology, London, UK

7. Royal Free Hospital, London, United Kingdom

8. Division of Ophthalmology, Ribeirão Preto Medical School, University of São Paulo, Ribeirão Preto, São Paulo, Brazil

9. Ophthalmology Department, Queen Elizabeth Central Hospital, Blantyre, Malawi

10. Ophthalmology Department, School of Health Sciences, University of Rwanda, Kigali, Rwanda

Corresponding Author: Lisa Keay

Postal Address: School of Optometry and Vision Science, The University of New South Wales, Sydney UNSW NSW 2052, Australia

Email: l.keay@unsw.edu.au

For peer review only

ABSTRACT

Objectives: To systematically investigate the associations between vision impairment and risk of motor vehicle crash (MVC) involvement, and evaluate vision-related interventions to reduce MVCs.

Design: Medline (Ovid), EMBASE and Global Health electronic databases were systematically searched from inception to March 2022 for observational and interventional English-language studies. Screening, data extraction and appraisals using the JBI appraisal tools were completed by two reviewers independently. Where appropriate, measures of association were converted into risk ratios (RRs) or ORs (odd ratios) for meta-analysis.

Participants: Drivers of four-wheeled vehicle of all ages with no cognitive declines.

Primary and secondary outcomes: MVC involvement (primary) and driving cessation (secondary)

Results: 101 studies (n=778,052) were included after full-text review. 57 studies only involved older drivers (≥65 years) and 85 were in high-income settings. Heterogeneity in the data meant that most meta-analyses were underpowered as only 25 studies, further split into different groups of eye diseases and measures of vision, could be meta-analysed. The limited evidence from the meta-analyses suggests that visual field defects (four studies; RR 1.51 (95%Cl 1.23-1.85); p<0.001; I^2=46.79%), and contrast sensitivity (two studies; RR 1.40 (95%Cl 1.08-1.80); p=0.01, I^2=0.11%) and visual acuity loss (5 studies; RR 1.21 (95%Cl 1.02, 1.43); p=0.03, I^2=28.49%) may increase crash risk. The results are more inconclusive for available evidence for associations of glaucoma (five studies, RR 1.27 (95%Cl 0.67-2.42); p=0.47; I^2=93.48%) and cataract (two studies RR 1.15 (95%Cl 0.97-1.36); p=0.11; I^2=3.96%) with crashes. Driving cessation may also be linked with glaucoma (two studies; RR 1.62 (95%Cl 1.20-2.19); p<0.001, I^2=22.45%), age-related macular degeneration (AMD) (three studies; RR 2.21 (95%Cl 1.47-3.31); p<0.001, I^2=75.11%) and reduced contrast sensitivity (three studies; RR 1.30 (95%Cl 1.05-1.61); p= 0.02; I^2=63.19%). Cataract surgery halved MVC risk (three studies; RR 0.55 (95%Cl 0.34-0.92); p= 0.02; I^2=97.10). Ranibizumab injections (four RCTs) prolonged driving in persons with AMD.

Conclusion: Impaired vision identified through a variety of measures is associated with both increased MVC involvement and cessation. Cataract surgery can reduce MVC risk. Despite literature being highly heterogeneous, this review shows that detection of vision problems and appropriate treatment is critical to road safety.

PROSPERO registration: CRD42020172153

For peer review only - http://bmjopen.bmj.com/site/about/guidelines.xhtml

Strengths and Limitations of this Study

- This is an up-to-date systematic review capturing literature on a variety of eye diseases and conditions, measures of vision such as visual acuity, contrast sensitivity, glare sensitivity, and visual field, and vision-related interventions and their associations on MVC involvement and driving cessation.
- There were no geographical or age restrictions placed on the population of focus allowing the global impact of vision impairment on driving to be documented for all age groups.
- Meta-analysis was limited due to heterogeneity in the outcome measures reported and the definitions of vision loss and or impairment used in each study. This heterogeneity also prohibited sub-group analyses by age and geographic location.
- Only statistical heterogeneity was assessed and not clinical or methodological.
- Publication bias was not assessed as there were less than ten studies included in each meta-analysis.

For peer review only

For peer review only - http://bmjopen.bmj.com/site/about/guidelines.xhtml

INTRODUCTION

Globalisation and economic development have made driving one of the main modes of transport worldwide and passenger vehicle travel is predicted to triple between 2015 and 2050.[1] Driving allows for independent mobility and enhances access to employment and education. Unfortunately, with more drivers on the roads, motor vehicle crashes (MVCs) and road traffic injuries are increasing worldwide. Approximately 1.35 million MVC-related fatalities occur each year with an additional 20-50 million people experiencing road-related injuries per annum.[2] The United Nations have therefore created targets within the Sustainable Development Goals (SDG) which aim to halve road deaths by 2020 (Target 3.6) and provide safe and sustainable transport systems for vulnerable road users (Target 11.2).[3]

Driving is a common and valued activity for many adults. Driving cessation limits independent mobility and has been linked to depressive symptoms and poorer health in older adults.[4] Functional declines in vision disproportionately impact older drivers, as they have higher prevalence of poor vision and eye diseases.[5, 6] Some countries have specific licensing requirements for older drivers,[7] however variations in visual driving standards across jurisdictions have made it difficult to assess whether these standards have safety benefits.[8]

This review was completed in collaboration with the *Lancet Global Health Commission* on Global Eye Health[9] and aimed to systematically evaluate the evidence to (1) investigate the associations between vision impairment and risk of MVC involvement across the lifespan, and (2) evaluate vision-related interventions to reduce MVCs. Since risks can be mitigated by driving retirement, this review also considered driving cessation as a secondary outcome.

METHODS

This systematic review was reported using PRISMA guidelines[10] (*Appendix 1*) using a published protocol.[11] An electronic database search on Medline (Ovid), EMBASE and Global Health was conducted from their inception to March 2020, and then updated in March 2022, with no geographic restrictions. *Appendix 2* details the search strategy with *Table* 1 describing the inclusion and exclusion criteria for studies.

For peer review only

Table 1 Study inclusion and exclusion criteria

Inclusion	Exclusion
• Interventional (RCTs) and observational (cohort, cross-sectional, case-control and case series) studies.<br>• Systematic reviews with meta-analyses<br>• Studies on drivers of four-wheeled motorised vehicles of all ages.<br>• Studies looking at the following exposures of interest: impairment in measures of vision (visual acuity (VA), contrast sensitivity (CS), visual field (VF), and glare sensitivity (GS)) or specific eye conditions including but not limited to glaucoma, cataracts, age-related macular degeneration (AMD), diabetic retinopathy (DR), stereopsis disorders and colour vision deficiencies.<br>• Studies on interventions such as vision screening, refractive correction, cataract surgery, anti-VEGF injections and other treatments to improve vision.	• Literature reviews and narrative systematic reviews.<br>• Commentary articles, dissertations, abstracts, editorials and conference presentations.<br>• Studies using simulators or investigated either self-regulatory driving behaviours (e.g. night driving avoidance), or self-reported measures of driving safety.<br>• To narrow the scope of the study, studies on populations with specific non-vision related medical conditions (e.g. dementia, epilepsy, stroke and history of medical events such as syncope), low vision or vision difficulties caused by other medical conditions (e.g. hemianopia caused by brain damage).<br>• Studies which simulated vision impairment.

The population of focus was drivers of four-wheeled motorised vehicles, of all ages, with no cognitive declines. Exposures of interest included eye diseases (e.g. glaucoma, cataract, age-related macular degeneration, diabetic retinopathy) and conditions (e.g. refractive errors), and measures of vision such as, but not limited to, visual acuity and contrast sensitivity. Studies reporting on interventions focused on treatments that would improve vision. The primary outcome measure was MVC involvement identified from self-reported surveys or government/hospital administrative datasets. The secondary outcome was self-reported driving cessation. Due to the large volume of data collected, other surrogate measures of driving safety and driving performance planned in the original protocol were beyond the scope of this manuscript but will be reported in a separate systematic review.[11] Studies which used simulators, or investigated self-regulatory driving behaviours (e.g. night driving avoidance) through surveys were excluded.

All titles, abstracts, and full-texts were reviewed independently by two investigators using Covidence systematic review management software (Covidence non-profit SaaS Enterprise, Melbourne, Australia). All discrepancies were resolved via consultation with a third investigator. Similarly, data extraction was completed independently by two investigators using data extraction forms adapted from either the Joanna Briggs Institute (JBI) templates for observational and systematic review study designs, or Cochrane templates for interventional studies. Data extracted from the studies included design, participant and setting characteristics, exposure type and definition, intervention details (if any), outcome measures, and relevant effect measures.

Overall risk of bias for all included studies was assessed by two investigators independently with conflicts resolved by a third investigator. All quality assessments were conducted using the relevant

For peer review only - http://bmjopen.bmj.com/site/about/guidelines.xhtml

JBI critical appraisal tools.[12] Each question on the relevant tools were categorised into either selection, detection, confounding, validity, performance, attrition, or allocation bias by all authors. Thus, a range of biases were considered appropriate to this research question. Each study was given an overall “score” on each question answered where a higher score represented less bias in the study design and execution. Based on how the questions were asked, a “yes” indicated that some sort of measure to limit bias was undertaken. The final scores were used to assign each study as low, medium, or high risk of bias, with lower scores indicating higher risk of bias.

Statistical Analysis

Associations between vision impairments and vision-related interventions with MVC involvement and driving cessation were summarised with appropriate Hazard Ratios (HR), Risk Ratios (RR), or Odds Ratios (OR). Narrative summaries were reported using the SWiM guidelines.[13] Heterogeneity across studies were assessed using I^2 statistic. Meta-analysis was conducted by converting all effects measures into RR or OR. Random-effects meta-analysis was only conducted on studies which presented data with the same outcomes, exposures and comparators, and which reported on associations adjusted for confounders to reduce bias. Data from case-controls were not pooled for meta-analysis to minimise possible heterogeneity. No publication bias analysis was conducted as there were less than 10 studies in each meta-analysis. Reporting of the results were guided by the Meta-analysis of Observational Studies in Epidemiology (MOOSE) guidelines.[14] All analyses were completed using STATA v17.

Patient and Public Involvement

Only existing published literature were looked at in this review and therefore no patient or public involvement was present during the design or execution of the review. Public participation may be sought out for future dissemination of this review.

RESULTS

From the electronic database search, 5111 studies were identified after the removal of 2131 duplicates. After title and abstract screening, 243 studies remained for full-text review after which 142 studies were further excluded, leaving 101 studies for data extraction (*Figure 1*).

63 studies (31 cross-sectional, 19 cohort, 12 case-control and one systematic review with meta-analysis) reported on MVC involvement alone, 34 (21 cross-sectional, eight cohort, two case-control, one case series, and two RCTs) on driving cessation, and four (one cross-sectional, two cohort, and one case-control) on both MVC and cessation. When split by geographic regions, 48 studies from HICs and 15 studies from LMICs reported solely on MVC involvement, whilst all 34 studies looking at driving cessation only came from HICs. From the studies which reported on both MVC and driving cessation, only one was from a LMIC. Study breakdown according to driving outcome and vision impairment is shown in *Table 2(a-b)*. The majority of studies (84%) were set in HICs and 57 studies (56%) focused on older adults. However, when looking at the 16 studies set in LMICs, all but two had an average study population age of less than 65 years. From the total 101 studies, only 13 (seven from HICs, six from LMICs; 12 cross-sectional, one cohort) were categorised as high risk of bias with the rest rated as either low or medium (*Appendix 3*).

For peer review only - http://bmjopen.bmj.com/site/about/guidelines.xhtml

Table 2a Breakdown of studies reporting on vision-related associations by outcome measure

Driving Outcome	Vision Impairment	Region (HIC/LMIC)	Total No. of Studies
**Motor Vehicle Crash (MVC)**	Glaucoma	15 HICs; 1 LMIC	16
	Cataract	8 HICs	8
	AMD	6 HICs	6
	Diabetic Retinopathy	3 HICs	3
	Stereopsis Impairment	2 HICs; 3 LMICs	5
	Myopia	2 HICs; 2 LMICs	4
	Colour Blindness	1 HICs; 7 LMICs	8
	Contrast Sensitivity	13 HICs	13
	Visual Acuity	19 HICs; 9 LMICs	28
	Glare Sensitivity	3 HICs	3
	Visual Field Impairment	14 HICs; 6 LMICs	20
	Other†	13 HICs; 6 LMICs	19
**Driving Cessation**	Glaucoma	12 HICs; 1 LMIC	13
	Cataract	5 HICs	5
	AMD	5 HICs	5
	Contrast Sensitivity	8 HICs	8
	Visual Acuity	18 HICs	18
	Glare Sensitivity	3 HICs	3
	Visual Field Impairment	8 HICs	8
	Other‡	11 HICs	11

†unilateral vision impairment, general vision impairment, retinopathy, retinal detachment, poor visibility, refractive disorder, monocular vision impairment, hyperopia, amblyopia, diplopia, astigmatism, retinitis pigmentosa, stereoacuity

‡dark adaptation, age-related maculopathy, detached retina, non-refractive vision impairment, self-reported vision loss, retinal haemorrhage, uncorrected refractive error

For peer review only - http://bmjopen.bmj.com/site/about/guidelines.xhtml

2b Breakdown of studies reporting on a vision-related intervention by intervention type, vision impairment and outcome measure

Intervention	Vision Impairment	Driving Outcome	Region (HIC/LMIC)	No. of Studies
**Anti-VEGF Injections**	AMD	Driving Cessation	1 HIC	1
	Diabetic Macular Oedema (DME)		1 HIC	1
**Cataract Surgery**	Cataract	Motor vehicle crash	6 HICs	6
		Driving Cessation	2 HICs	2
**Corrective Lenses**	Refractive error	Motor vehicle crash	1 HICs	1
**Anti-glaucoma therapy**	Glaucoma	Driving Cessation	1 HICs	1

Raw data on studies reporting on MVCs[15-81] and driving cessation[70-73, 82-115] can be found in *Appendix 4a(i-xiv)* and *4b(i-ix),* respectively, with additional narrative summaries. Meta-analyses on associations are presented in *Appendix 5a-b*; only 25 studies could be meta-analysed. Studies were not included in the meta-analysis if different comparators were used, different driving outcomes were analysed (any MVC involvement, at-fault MVCs, injurious and non-injurious MVCs), or different cut-off points or definitions for vision impairment. For example, there were studies that looked at bilateral VA at 6/12 and worse while there were others that looked at unilateral VA being "poor" but without a formal definition of what "poor" acuity meant. Studies rated as having a high bias were also excluded from the meta-analyses. *Figure 2* synthesises the narrative summaries to show multiple associations of vision with MVCs and driving cessation. From *Figure 2* it can be seen that associations reported for eye diseases and measures of vision function were more consistent across studies looking at cessation compared to crashes. When considering vision-related interventions, only cataract surgery was shown to improve driving by minimising crash risk. The benefits of anti-VEGF injections on prolonging driving was more inconclusive and found to only help drivers with AMD but not diabetic macular oedema. However as a whole, the evidence from the literature on associations between vision impairment and crashes and cessation are mostly inconclusive and or mixed.

<u>Associations between eye diseases and conditions/measures of vision loss and motor vehicle crashes</u>

The results were mixed (16 studies, n=21,214 participants) for associations between glaucoma and MVCs.[24, 30, 38, 41, 43, 45, 46, 52, 54, 65, 67-72] As illustrated in *Appendix 5a*, meta-analyses found a glaucoma diagnosis to not increase the risk of any MVC involvement (OR 1.27 (95% CI 0.67-2.42); p=0.47) however this estimate has a wide confidence interval limiting the power to investigate this association.[24, 30, 38, 43, 72] Other studies were excluded from the meta-analysis as there was no similarity on the comparators used, how glaucoma was categorised (mild vs. severe, unilateral vs. bilateral) and the crash outcomes investigated (any MVC involvement, injurious vs. non-injurious, at-fault). Similarly, meta-analyses on three studies[24, 30, 43] looking at at-fault crashes also found no difference between drivers with and without glaucoma (RR 1.89 (95% CI 0.40-8.86); p=0.42). Increased risk was evident with more severe glaucoma.[30, 38, 43, 46, 52, 65, 69, 70]

For peer review only - http://bmjopen.bmj.com/site/about/guidelines.xhtml

Out of the eight cataract studies (n=18,883) identified,[24, 40, 41, 45, 54, 56, 57, 72] most found self-reported, physician-diagnosed cataracts did not impact the likelihood of any type of MVC involvement. Meta-analysis suggests that were no increased risk (*Appendix 5a*; OR 1.15 (95% CI 0.97-1.36); p=0.11);[24, 40] however this was underpowered with only two studies used for analysis. At-fault crash involvement was investigated by two studies however only one reported significant associations.[24, 56]

Meta-analysis could not be conducted on any studies looking at drivers with either AMD (five studies, n=4150)[24, 41, 44, 64, 66] or diabetic retinopathy (3 studies, n=4353),[24, 45, 54] however no studies found increased risk of MVC. No studies were meta-analysed as studies on AMD all had different comparator or different grades of AMD and MVC type, while studies on DR had different comparators and looked at different crash outcomes.

Impairments in stereopsis were not found to increase the risk of MVC involvement across the five studies identified (n=3253).[22, 33, 40, 51, 75] Meta-analysis on three studies showed no difference in crash involvement between those with and without stereopsis impairment (*Appendix 5a*; RR 1.03 (95% CI 0.86-1.23); p=0.74).[22, 40, 51]

Summary of studies on myopia (four studies, n= 2039)[22, 23, 41, 74] also found no increased risk of MVC involvement. A combination of two of these studies in meta-analysis (*Appendix 5a*) also did not find evidence of an association (OR 0.76 (95% CI 0.34-1.70; p=0.51),[22, 74] noting limitation of sample size for concrete conclusions to be made. One study investigating persons with night myopia reported slightly more night-time MVCs in these drivers than those without night myopia (p=0.044).[23]

Colour vision deficiency and the risk of MVC involvement among commercial truck drivers was investigated in eight studies (n=7916);[15, 21, 22, 34, 51, 53, 59, 77] seven set in LMICs. Three studies found an association,[15, 51, 59] however their results were not combined due to reliance on Ishihara plates which do not reliably diagnose colour vision deficiency.

Visual acuity (VA) (28 studies, n=39,129) was not found to be associated with crash involvement by 19 studies,[17, 20, 22, 24, 27-29, 31, 33-36, 38, 40, 41, 45, 50-54, 57, 63, 68, 69, 73, 75, 77, 80] irrespective of crash scenario (at-fault or not-at-fault) and severity (injurious or non-injurious). Bilateral VA 20/40 or worse may impact risk of MVCs (meta-analysis five studies; RR 1.21 (95% CI 1.02-1.43); p= 0.03).[27, 31, 40, 73, 77] Combining two studies found no evidence for an associations with 'not at-fault' MVCs (RR 1.08 (95% CI 0.74-1.60); p= 0.68) (*Appendix 5a*),[27, 31] however there was limited power to explore associations.

Mixed results were reported from 13 studies (n=17,941) looking at any MVC involvement and reduced contrast sensitivity (CS).[24, 27, 31, 35, 38, 40, 54, 57, 58, 73] However, due to heterogeneity in outcome measures reported and definition of reduced CS, the meta-analysis in *Appendix 5a* was restricted to only two studies which found CS to increase crash risk (RR 1.40 (95% CI 1.08-1.80); p=0.01).[31, 79] When photopic and mesopic areas under the log contrast sensitivity were investigated with any and at-fault crash involvement, only lower mesopic peaks were found to be predictive.[58]

From the 20 studies (n=13,533) looking at visual field (VF) loss and crashes, heterogeneity in the definition of VF loss and the crash outcomes investigated meant that only four were meta-analysed. The results suggests an increased risk of MVC with bilateral field loss (RR 1.51 (95% CI 1.23-1.85); p< 0.001) (*Appendix 5a*).[32, 51, 77, 79] There were mixed results with 9/20 studies finding an increased risk,[31, 32, 38, 42, 54, 73, 77-79] 1/20 an association for a co-linear dependent variable

For peer review only - http://bmjopen.bmj.com/site/about/guidelines.xhtml

[19] and 10/20 a null finding.[16, 17, 33, 34, 37, 51, 53, 59, 68, 69] The increased risks were found in association with severe, bilateral VF loss and field loss affecting both central and peripheral vision.

Most studies on glare sensitivity impairments (3 studies, n=3191) found weak to no associations with crash risk;[54, 57, 73] they were unable to be meta-analysed.

19 studies (n=100167) reported on other impairments including: unilateral vision impairment,[18] general vision impairment,[21, 25, 28, 39, 41, 59, 61, 74, 76, 80, 81] non-diabetic retinopathy,[41] retinal detachment,[72] other retinal disorders,[41] refractive disorder,[41] monocular vision impairment,[41, 50] presbyopia,[41, 74] hyperopia,[22, 74] amblyopia,[18, 60] diplopia,[41] astigmatism,[22, 41] retinitis pigmentosa[26] and stereoacuity.[54, 73] Most did not find associations with MVCs, however one study from the USA reported increased injurious MVC involvement with impaired stereoacuity.[54] Another study in the UK reported increased MVC involvement with moderate/severe amblyopia[41] whilst two other studies, one in Ethiopia[21] and the other in Bangladesh,[74] reported increased MVC involvement with self-reported bilateral visual impairment.

Impact of vision-related interventions on motor vehicle crashes

Most of the six studies (n=592,897) on cataract surgery found the risk of MVC to decrease following cataract surgery[41, 47-49, 55, 62] and the 3 studies suitable for meta-analysis estimated the risk to halve (RR 0.55 (95% CI 0.34-0.92); p=0.02) (*Appendix 5a*).[47, 48, 55] Greater reductions to crash risk are seen after first eye surgery compared to second eye.[47] Similarly, the risk of crashing in males post-surgery is lower than females.[49]

Corrective lenses for far and near vision refractive disorders were only investigated by one study which found no associations with crash risk.[41]

Associations between eye diseases and conditions/measures of vision loss and driving cessation

There were 13 studies (n=21,939) investigating associations between glaucoma and the likelihood of driving cessation with estimates ranging from an increased risk of 1.3 to increased odds of 4.[70-72, 87, 91, 92, 99, 100, 103, 109-111, 113] The meta-analysis in *Appendix 5b* suggests a diagnosis of glaucoma to increase the risk of driving cessation by 63% (95% CI 1.20 - 2.19; p< 0.01),[87, 91] however this analysis only contained two studies.

Four studies (n=14,402) looked at cataract and driving cessation with three studies reporting an increased likelihood of driving cessation by over 1.5 times; none could be meta-analysed.[72, 99, 100, 106]

From the five studies (n=6183) identified,[85, 87, 99, 106, 108] three found the presence of AMD to be predictive of driving cessation, with meta-analysis on three suitable studies reporting the overall risk of cessation to increase by 2.21 (95% CI 1.47-3.31; p< 0.01) (*Appendix 5b*).[85, 87, 108]

Even though the 18 identified studies (n=23,712) were highly heterogeneous,[73, 82, 86-88, 90, 91, 94-98, 103-106, 110, 111] impaired or "poor" VA was shown to increase the chances of driving cessation in most studies,[87, 103, 104, 106, 111] with better visual acuity decreasing the risk of cessation by up to 70%.[90] The two studies looking at visual acuity in persons with glaucoma, had mixed conclusions on the effect of visual acuity on driving cessation.[95, 110]

Eight studies (n=9602) looked at the impact of CS on driving cessation.[73, 88, 94, 96, 97, 103, 106, 111] From the studies which categorised contrast sensitivity as "poor", meta-analysis found poor CS to increase the risk of cessation (RR 1.30 (95% CI 1.05-1.61); p=0.02) (*Appendix 5b*).[94, 96, 106] Another study reported participants who had a decline of 6 or more letters in their contrast

For peer review only - http://bmjopen.bmj.com/site/about/guidelines.xhtml

sensitivity levels after two years, as measured by a Pelli-Robson chart, to have a 71% increased risk of driving cessation.[88]

VF loss and driving cessation was investigated by 8 studies (n=7988),[88, 94-97, 103, 105, 111] and all but one found associations.[105] The likelihood of cessation was generally greater with bilateral and or more severe field loss.[88, 94, 111] One study looking at persons with bilateral glaucoma found visual field loss to double the odds of cessation.[103]

Glare sensitivity (3 studies, n=5577) and was not found to be consistently associated with driving cessation.[88, 91, 110]

Eleven studies (n=12,897) looked at driving cessation with other types of vision impairment: dark adaptation,[110] age-related maculopathy,[86] retinal detachment,[85] non-refractive vision impairment,[112] general vision loss,[85, 89, 93, 98, 100, 114, 115] retinal haemorrhage,[85] and uncorrected refractive error.[97, 112] Only two studies, one reporting on retinal haemorrhage[85] and the other on non-refractive vision impairment and uncorrected refractive error,[112] found increased risk of driving cessation.

Impact of vision-related interventions on driving cessation

There were two studies reporting the driving status of participants after anti-VEGF therapy (0.5 mg ranibizumab) from 4 different RCTs; MARINA (n=716; 24 months; control=sham injections) and ANCHOR (n=423; 24 months; control=photodynamic therapy (PDT)) which targeted AMD,[83] and RIDE/RISE (n=759; 24 months; control=sham injections) and RESTORE (n=345; 12 months; control=PDT) which targeted diabetic macular oedema (DMO).[84] By the end of all four trials, only drivers with AMD but not DMO treated with anti-VEGF were shown to have marked differences with the control group for the number of people who continued driving from baseline (AMD: MARINA: p=0.035, ANCHOR: p=0.002; DMO: RIDE/RISE: p=0.655, RESTORE: p=0.125).

Both studies (n=1021) looking at driving status after cataract surgery reported an increase in the proportion of participants driving after successful surgery.[101, 102]

There was only one study (n=240) looking at driving after anti-glaucoma therapy (pilocarpine-epinephrine),[107] however this is an old study and this treatment is no longer in use.

DISCUSSION

This review synthesises diverse and complex evidence from 101 studies examining vision and its impact on MVCs and driving cessation across all ages. The majority of studies in this review focused on older adults and reported more associations between vision impairment and MVCs and or cessation compared to studies on younger populations. Research was mostly observational with few studies examining the impact of interventions to improve vision. The studies excluded from the meta-analysis tended to have mixed results regarding the associations between the vision impairment and driving outcome, whereas the studies in the meta-analyses were more consistent showing definitive associations for VA, CS and VF defects. Nonetheless, the mixed results in the narrative summaries however support the emerging idea of adding visual processing and cognitive tests alongside visual assessments to produce more predictive measures of safe driving.[116] When looking at the vision-related interventions, cataract surgery was shown to halve the risk of crashing. Others have reported that following cataract surgery, driving difficulties, such as self-reported night driving ability, reduced by 88%[117] with improvements in contrast sensitivity linked to these changed perceptions.[118]

For peer review only - http://bmjopen.bmj.com/site/about/guidelines.xhtml

Variability in the relationships between vision and MVCs may be due to several reasons. The first set of reasons surround how MVCs are defined and investigated in the literature. Firstly, there are many different MVC scenarios based upon the driver's role (at-fault or not) and severity (injurious or non-injurious) which are not always differentiated in research studies. MVCs are also studied in a variety of ways from self-reports to analyses of large crash databases. This may cause reliability issues. For example, an American study found agreement between these two collection methods was poor when examining the total MVCs over a three-year period.[119] Crashes can also stem from external and vehicular factors which make drawing conclusions solely based on human factors inappropriate.[120] Self-regulation, jurisdictional control on vision standards for licensing, and driving cessation could all mitigate the risk of crash involvement. The second set of reasons have to do with the vision impairment themselves and the severity of the impairment. The studies which reported increase crash risk, associated with diagnosis of an eye disease, evaluated more severe forms of the disease and worse functions of vision. Studies examining impact of a diagnosis of a disease tended to report no associations. For example, the lack of association between a diagnosis of cataract and MVC could be because the cataract is mild and is not having a significant impact on CS. A parallel review from our group has found greater defects in these measures to worsen driving performance and increase errors, which can theoretically lead to more crashes.[121] It is therefore critical to capture the severity of an eye disease and/or the actual level of vision impairment when impact of disease status on crash risk . As seen in this review, even though glaucoma, cataract and AMD had mixed or no associations with crashing, their corresponding measures of vision, mainly VF, CS and VA respectively, were definitively associated. This may be why associations found between vision impairment and driving cessation were strong and consistent. A diagnosis of glaucoma or AMD, and poor contrast sensitivity were all found to increase the risk of driving cessation. Anti-VEGF injections could prolong driving for people with AMD. This is of importance as older adults greatly value independent mobility and regard driving as a vital activity for daily living.[122, 123] With driving cessation linked towards multiple negative health outcomes in older adults,[4] anti-VEGF injections can have wider health benefits beyond direct impact on vision.

This review also highlights the paucity of research from LMICs despite approximately 93% of all road-traffic related deaths occurring in these countries, particularly in Africa and amongst young road users.[2] Despite the UN's push, most LMICs still lag behind the SDG targets on halving road traffic mortality set in the Decade of Action for Road Safety (2011-2020).[124] Previous systematic reviews point towards legislation-based interventions which modify behaviour, such as seat-belt and helmet use, to be the most effective at reducing road injuries and crash rates in LMICs.[125, 126] These interventions are in line with UN recommendations for improving infrastructure, vehicle safety standards, and safe road user behaviours in order to reach the targets set for SDG 3.6 and 11.2.[127] However, there is no mention of licensing standards which needs to be addressed as motorisation increases worldwide. Evidence from this global review supports vision standards for licensing to be updated, enforced and given higher priority in LMICs. Even though most LMICs do have guidelines on vision, especially for commercial drivers, it is apparent from the studies in this review that many drivers unfortunately do not satisfy these conditions. This may be because many people in LMICs lack access to eye-health care services. The evidence for a corresponding increase in MVCs in LMICs is not well established with only one systematic review identified looking at data from these regions.[77] Though data from HICs can inform research and policy development in LMICs, increasing the evidence base from LMICs will ensure that interventions to reduce MVCs and maintain access to driving in LMICs can be reflective of the local context.

Older drivers tend to self-regulate their driving habits by reducing their driving mileage and radius and avoiding high-risk driving situations.[128] Vision impairments have been reported to increase

For peer review only - http://bmjopen.bmj.com/site/about/guidelines.xhtml

the likelihood of self-regulation by 19%,[129] with older drivers who self-rate their vision as “poor” 15 times more likely to modify their driving than those who regard their vision as “excellent”.[123] Our findings are consistent with these patterns of self-regulation and a diagnosis of AMD or glaucoma were found in this review to be associated with driving cessation. It is likely that self-regulation is an intermediate step towards driving cessation encompassing reductions in driving frequencies and distance.[130] However, self-regulation has been reported as an insufficient compensatory measure to reduce crash risk amongst older drivers with a vision impairment,[131, 132] which would therefore explain why glaucoma, particularly more severe glaucoma, was still linked with crashes in some studies. The relationship between crash involvement and AMD, however, was inconclusive. This may be because AMD affects central vision, thus making declines in this field easily noticeable allowing individuals to appropriately adapt their driving behaviours. Laboratory studies simulating central vision impairments show negative impacts on driving performance and safety, particularly with increasing age and distraction.[133] Further research is needed on driving patterns and behaviours of individuals with eye diseases.

Few studies, all from LMICs, in this review reported associations between colour vision deficiency and crash risk. Unfortunately, based upon their high risk of bias, these studies were deemed unsuitable for meta-analysis. This does not mean, however, that their results should be dismissed. Previous simulation studies found persons with colour vision deficiency performed worse in driving simulations compared to those with normal colour vision.[134-136] However, these associations have not always been evident in studies of MVC risk.[137] This might be why recommendations proposed by the Commission Internationale de l’Eclairage, the international authority on lighting and signal lights, are for commercial drivers only.[135] Associations found in LMICs highlight issues regarding poor road infrastructure and lighting standards.[138] Further research is needed, with standardised diagnosis of colour vision deficiency and consideration of improvements to lighting and signals in the road environment in LMICs.

This review summarises global data on different eye diseases, declines in vision function and vision-related interventions, which makes the findings applicable worldwide considering motorisation and ongoing issues of vision loss, particularly in older people. There are, however, limitations which should be acknowledged. This review highlights the highly heterogeneous nature of research investigating the impact of vision on driving which unfortunately presented several methodological limitations. Firstly, only a small number of studies could be synthesized for meta-analyses due to differences in study design. The underpowered meta-analyses meant that no absolute conclusions can be made from these results alone. It is therefore imperative that the meta-analyses results be considered alongside the narrative summaries to gain a full picture of the literature in this field. Further, this review did not consider how comorbidities, alongside vision impairment, can impact the risk of crash and driving cessation. Older adults with a vision impairment have been found to be twice as likely than those without a vision impairment to have five or more physical and/or cognitive comorbidities.[139] It is possible that the association with vision is confounded by the impact of comorbidities. Unfortunately, not all the studies included in this review reported on the comorbidities of their participants, limiting our ability to explore this possible source of bias and the extent to how this might have explained the heterogeneity of the pooled estimates via meta-regression. There were great variations in the comparator group used in each study and there were inconsistent cut-off points amongst studies looking at continuous measures of vision function. This heterogeneity also prevented sub-group analyses comparing younger to older age groups and geographic regions. Clinical and methodological heterogeneity could not be investigated, even though details on participant characteristics, relevant interventions and study designs were collected, due to the small number of studies included in each meta-analysis. Looking at these

For peer review only - http://bmjopen.bmj.com/site/about/guidelines.xhtml

parameters, however, might have explained the high statistical heterogeneity in select meta-analyses. The published meta-analysis, however, was summarised narratively to ensure duplicate studies were not included in this evidence synthesis. Grey literature and non-English studies were not included which may have introduced publication bias and limited the number of studies identified from LMICs. Future research incorporating these areas may provide a clearer picture on how vision impairment is affecting global road safety.

In conclusion, this review summarises the global literature on the impact of vision and vision-related interventions on driving as part of the *Lancet Global Health* Commission on Global Eye Health. Select measures of vision impairment such as visual field, visual acuity and contrast sensitivity loss, and eye diseases such as glaucoma and AMD, were found to be associated with either crashes or driving cessation, while interventions such as cataract surgery and anti-VEGF injections mitigated these outcomes. However, the current literature is highly heterogeneous, and more studies are needed from LMICs to ensure that what is known about vision and driving in these settings. Future studies should aim to address these issues to allow for the global context of vision impairment and driving safety to be better documented, which may assist in the achievement of the UN's SDG road safety targets.

AUTHORS' CONTRIBUTIONS

HN, LK and JR conceived the idea of for the review. IG constructed the search. Title and abstract screening and data extraction were completed by HN, KR, JR, JHZ, JF, SM and GFK. HN drafted and revised the manuscript with suggestions from LK, JR, JB, KC, GLDT, KR, MJB, JHZ, IG, JF, SM and GFK who received the manuscript and provided feedback on the draft. The final version of the manuscript was approved by all authors.

FUNDING STATEMENT

HN is supported by the Australian Government Research Training Program (RTP) Scholarship (N/A). MJB is supported by the Wellcome Trust (207472/Z/17/Z). JR's appointment at the University of Auckland is funded by the Buchanan Charitable Foundation (N/A), New Zealand. The *Lancet Global Health* Commission on Global Eye Health was supported by grants from The Queen Elizabeth Diamond Jubilee Trust (N/A), Moorfields Eye Charity (GR001061), NIHR Moorfields Biomedical Research Centre (N/A), The Wellcome Trust (20190426_PH2), Sightsavers (N/A), The Fred Hollows Foundation (N/A), SEVA Foundation (N/A), British Council for the Prevention of Blindness (N/A), and the Christian Blind Mission (N/A).

ACKNOWLEDGEMENTS

None

COMPETING OF INTERESTS

None declared

ETHICS APPROVAL

Not applicable

DATA SHARING

All data relevant to the study are included in the article or uploaded as supplementary information.

For peer review only

REFERENCES

1. Acker A, Jari K. How transport demand will change through 2050 2019 [Available from: https://www.oecd-ilibrary.org/sites/transp_outlook-en-2019-en/1/1/4/index.html?itemId=/content/publication/transp_outlook-en-2019-en&_csp_=1b3375008054c148f41fef71cd42b552&itemIGO=oecd&itemContentType=book.
2. World Health Organisation. Road Traffic Injuries 2020 [Available from: https://www.who.int/news-room/fact-sheets/detail/road-traffic-injuries.
3. United Nations. #Envision2030: 17 goals to transform the world for persons with disabilities 2015 [Available from: https://www.un.org/development/desa/disabilities/envision2030.html.
4. Chihuri S, Mielenz TJ, DiMaggio CJ, Betz ME, DiGuiseppi C, Jones VC, et al. Driving Cessation and Health Outcomes in Older Adults. Journal of the American Geriatrics Society. 2016;64(2):332-41.
5. Owsley C, Ball K, Sloane ME, Roenker DL, Bruni JR. Visual/cognitive correlates of vehicle accidents in older drivers. Psychol Aging. 1991;6(3):403-15.
6. Wood JM, Black AA. Ocular disease and driving. Clinical & experimental optometry. 2016;99(5):395-401.
7. Bohensky M, Charlton J, Odell M, Keeffe J. Implications of vision testing for older driver licensing. Traffic injury prevention. 2008;9(4):304-13.
8. Yan MK, Kumar H, Kerr N, Medeiros FA, Sandhu SS, Crowston J, et al. Transnational review of visual standards for driving: How Australia compares with the rest of the world. Clinical & experimental ophthalmology. 2019;47(7):847-63.
9. Burton MJ, Ramke J, Marques AP, Bourne RRA, Congdon N, Jones I, et al. The Lancet Global Health Commission on Global Eye Health: vision beyond 2020. The Lancet Global health. 2021;9(4):e489-e551.
10. Shamseer L, Moher D, Clarke M, Ghersi D, Liberati A, Petticrew M, et al. Preferred reporting items for systematic review and meta-analysis protocols (PRISMA-P) 2015: elaboration and explanation. BMJ (Clinical research ed). 2015;350:g7647.
11. Nguyen H, Di Tanna GL, Coxon K, Brown J, Ren K, Ramke J, et al. Associations between vision impairment and driving and the effectiveness of vision-related interventions: protocol for a systematic review and meta-analysis. BMJ open. 2020;10(11):e040881.
12. Univeristy of Adelaide. Critical Appraisal Tools N/A [Available from: https://joannabriggs.org/ebp/critical_appraisal_tools.
13. Campbell M, McKenzie JE, Sowden A, Katikireddi SV, Brennan SE, Ellis S, et al. Synthesis without meta-analysis (SWiM) in systematic reviews: reporting guideline. BMJ (Clinical research ed). 2020;368:l6890.
14. Stroup DF, Berlin JA, Morton SC, Olkin I, Williamson GD, Rennie D, et al. Meta-analysis of observational studies in epidemiology: a proposal for reporting. Meta-analysis Of Observational Studies in Epidemiology (MOOSE) group. Jama. 2000;283(15):2008-12.
15. Abebe Y, Wondmikun Y. Defective color perception among car drivers in Addis Ababa, Ethiopia. Traffic Injury Prevention. 2002;3(4):294-7.
16. Abraham EG, Ezepue UF, Umeh RE, Ekanem U. Causes of visual impairment among commercial motor vehicle drivers in Uyo metropolis, Akwa Ibom State, Nigeria. Orient Journal of Medicine. 2010;22(1/4):unpaginated.
17. Adekoya BJ, Owoeye JFA, Adepoju FG, Ajaiyeoba AI. Visual function survey of commercial intercity vehicle drivers in Ilorin, Nigeria. Canadian journal of ophthalmology Journal canadien d'ophtalmologie. 2009;44(3):261-4.
18. Baker JM, Drews-Botsch C, Pfeiffer MR, Curry AE. Driver licensing and motor vehicle crash rates among young adults with amblyopia and unilateral vision impairment. Journal of AAPOS : the

For peer review only - http://bmjopen.bmj.com/site/about/guidelines.xhtml

official publication of the American Association for Pediatric Ophthalmology and Strabismus. 2019;23(4):230-2.
19. Ball K, Owsley C, Sloane ME, Roenker DL, Bruni JR. Visual attention problems as a predictor of vehicle crashes in older drivers. Investigative ophthalmology & visual science. 1993;34(11):3110-23.
20. Bekibele CO, Fawole OI, Bamgboye AE, Adekunle LV, Ajayi R, Baiyeroju AM. Prevalence of refractive error and attitude to spectacle use among drivers of public institutions in Ibadan, Nigeria. Annals of African medicine. 2007;6(1):26-30.
21. Biza M, Mossie A, Woldemichael K, Gelaw Y. Visual impairment and road traffic accidents among drivers in Jimma Town, Southwest Ethiopia. Ethiopian medical journal. 2013;51(2):123-32.
22. Boadi-Kusi SB, Kyei S, Asare FA, Owusu-Ansah A, Awuah A, Darko-Takyi C. Visual function among commercial vehicle drivers in the central region of Ghana. Journal of optometry. 2016;9(1):54-63.
23. Cohen Y, Zadok D, Barkana Y, Shochat Z, Ashkenazi I, Avni I, et al. Relationship between night myopia and night-time motor vehicle accidents. Acta ophthalmologica Scandinavica. 2007;85(4):367-70.
24. Cross JM, McGwin G, Jr., Rubin GS, Ball KK, West SK, Roenker DL, et al. Visual and medical risk factors for motor vehicle collision involvement among older drivers. The British journal of ophthalmology. 2009;93(3):400-4.
25. Emerole CG, Nneli RO. Visual indices of motor vehicle drivers in relation to road safety in Nigeria. Nigerian journal of physiological sciences : official publication of the Physiological Society of Nigeria. 2013;28(1):57-62.
26. Fishman GA, Anderson RJ, Stinson L, Haque A. Driving performance of retinitis pigmentosa patients. The British journal of ophthalmology. 1981;65(2):122-6.
27. Green KA, McGwin G, Jr., Owsley C. Associations between visual, hearing, and dual sensory impairments and history of motor vehicle collision involvement of older drivers. Journal of the American Geriatrics Society. 2013;61(2):252-7.
28. Gresset J, Meyer F. Risk of automobile accidents among elderly drivers with impairments or chronic diseases. Canadian journal of public health = Revue canadienne de sante publique. 1994;85(4):282-5.
29. Gresset JA, Meyer FM. Risk of accidents among elderly car drivers with visual acuity equal to 6/12 or 6/15 and lack of binocular vision. Ophthalmic & physiological optics : the journal of the British College of Ophthalmic Opticians (Optometrists). 1994;14(1):33-7.
30. Haymes SA, Leblanc RP, Nicolela MT, Chiasson LA, Chauhan BC. Risk of falls and motor vehicle collisions in glaucoma. Investigative ophthalmology & visual science. 2007;48(3):1149-55.
31. Huisingh C, Levitan EB, Irvin MR, MacLennan P, Wadley V, Owsley C. Visual Sensory and Visual-Cognitive Function and Rate of Crash and Near-Crash Involvement Among Older Drivers Using Naturalistic Driving Data. Investigative ophthalmology & visual science. 2017;58(7):2959-67.
32. Huisingh C, McGwin G, Wood J, Owsley C. The driving visual field and a history of motor vehicle collision involvement in older drivers: A population- based examination. Investigative Ophthalmology and Visual Science. 2015;56(1):132-8.
33. Humphriss D. Three South African studies on the relation between road accidents and drivers' vision. Ophthalmic & physiological optics : the journal of the British College of Ophthalmic Opticians (Optometrists). 1987;7(1):73-9.
34. Isawumi MA, Adeoti CO, Ubah JN, Oluwatimilehin IO, Raji RA. Ocular status of commercial drivers in Osun State, Nigeria. African journal of medicine and medical sciences. 2011;40(4):405-11.
35. Ivers RQ, Mitchell P, Cumming RG. Sensory impairment and driving: the Blue Mountains Eye Study. American journal of public health. 1999;89(1):85-7.
36. Keeffe JE, Jin CF, Weih LM, McCarty CA, Taylor HR. Vision impairment and older drivers: who's driving? The British journal of ophthalmology. 2002;86(10):1118-21.

For peer review only - http://bmjopen.bmj.com/site/about/guidelines.xhtml

37. Kristalovich L, Ben Mortenson W. Visual Field Impairment and Driver Fitness: A 1-Year Review of Crashes and Traffic Violations. The American journal of occupational therapy : official publication of the American Occupational Therapy Association. 2019;73(5):7305345010p1-p6.
38. Kwon M, Huisingh C, Rhodes LA, McGwin G, Jr., Wood JM, Owsley C. Association between Glaucoma and At-fault Motor Vehicle Collision Involvement among Older Drivers: A Population-based Study. Ophthalmology. 2016;123(1):109-16.
39. Maag U, Vanasse C, Dionne G, Laberge-Nadeau C. Taxi drivers' accidents: how binocular vision problems are related to their rate and severity in terms of the number of victims. Accident; analysis and prevention. 1997;29(2):217-24.
40. Margolis KL, Kerani RP, McGovern P, Songer T, Cauley JA, Ensrud KE. Risk factors for motor vehicle crashes in older women. Journals of Gerontology - Series A Biological Sciences and Medical Sciences. 2002;57(3):M186-M91.
41. McCloskey LW, Koepsell TD, Wolf ME, Buchner DM. Motor vehicle collision injuries and sensory impairments of older drivers. Age and ageing. 1994;23(4):267-73.
42. McGwin G, Jr., Huisingh C, Jain SG, Girkin CA, Owsley C. Binocular visual field impairment in glaucoma and at-fault motor vehicle collisions. Journal of glaucoma. 2015;24(2):138-43.
43. McGwin G, Jr., Mays A, Joiner W, Decarlo DK, McNeal S, Owsley C. Is glaucoma associated with motor vehicle collision involvement and driving avoidance? Investigative ophthalmology & visual science. 2004;45(11):3934-9.
44. McGwin G, Jr., Mitchell B, Searcey K, Albert MA, Feist R, Mason JO, 3rd, et al. Examining the association between age-related macular degeneration and motor vehicle collision involvement: a retrospective cohort study. The British journal of ophthalmology. 2013;97(9):1173-6.
45. McGwin G, Jr., Sims RV, Pulley L, Roseman JM. Relations among chronic medical conditions, medications, and automobile crashes in the elderly: a population-based case-control study. American journal of epidemiology. 2000;152(5):424-31.
46. McGwin G, Jr., Xie A, Mays A, Joiner W, DeCarlo DK, Hall TA, et al. Visual field defects and the risk of motor vehicle collisions among patients with glaucoma. Investigative ophthalmology & visual science. 2005;46(12):4437-41.
47. Meuleners LB, Brameld K, Fraser ML, Chow K. The impact of first- and second-eye cataract surgery on motor vehicle crashes and associated costs. Age and ageing. 2019;48(1):128-33.
48. Meuleners LB, Hendrie D, Lee AH, Ng JQ, Morlet N. The effectiveness of cataract surgery in reducing motor vehicle crashes: a whole population study using linked data. Ophthalmic epidemiology. 2012;19(1):23-8.
49. Meuleners LB, Ng JQ, Fraser M, Hendrie D, Morlet N. Impact of gender on first eye cataract surgery and motor vehicle crash risk for older drivers. Clinical & experimental ophthalmology. 2012;40(6):591-6.
50. Ogbonnaya CE, Uro-Chukwu HC, Sunday-Adeoye I. Ocular health status and visual fitness of commercial drivers in Abakaliki Metropolis, Nigeria. East African Medical Journal. 2018;95(7):1758-66.
51. Oladehinde MK, Adeoye AO, Adegbehingbe BO, Onakoya AO. Visual functions of commercial drivers in relation to road accidents in Nigeria. Indian journal of occupational and environmental medicine. 2007;11(2):71-5.
52. Ono T, Yuki K, Asaoka R, Kouyama K, Abe T, Tanabe S, et al. Glaucomatous visual field defect severity and the prevalence of motor vehicle collisions in Japanese: a hospital/clinic-based cross-sectional study. Journal of ophthalmology. 2015;2015(101524199):497067.
53. Ovenseri-Ogomo G, Adofo M. Poor vision, refractive errors and barriers to treatment among commercial vehicle drivers in the Cape Coast municipality. African health sciences. 2011;11(1):97-102.
54. Owsley C, McGwin G, Jr., Ball K. Vision impairment, eye disease, and injurious motor vehicle crashes in the elderly. Ophthalmic epidemiology. 1998;5(2):101-13.

For peer review only - http://bmjopen.bmj.com/site/about/guidelines.xhtml

55. Owsley C, McGwin G, Jr., Sloane M, Wells J, Stalvey BT, Gauthreaux S. Impact of cataract surgery on motor vehicle crash involvement by older adults. JAMA. 2002;288(7):841-9.
56. Owsley C, Stalvey B, Wells J, Sloane ME. Older drivers and cataract: driving habits and crash risk. The journals of gerontology Series A, Biological sciences and medical sciences. 1999;54(4):M203-11.
57. Owsley C, Stalvey BT, Wells J, Sloane ME, McGwin G, Jr. Visual risk factors for crash involvement in older drivers with cataract. Archives of ophthalmology (Chicago, Ill : 1960). 2001;119(6):881-7.
58. Owsley C, Swain T, Liu R, McGwin G, Jr., Kwon MY. Association of Photopic and Mesopic Contrast Sensitivity in older drivers with risk of motor vehicle collision using naturalistic driving data. BMC ophthalmology. 2020;20(1):47.
59. Pepple G, Adio A. Visual function of drivers and its relationship to road traffic accidents in Urban Africa. SpringerPlus. 2014;3(101597967):47.
60. Rahi JS, Cumberland PM, Peckham CS. Does amblyopia affect educational, health, and social outcomes? Findings from 1958 British birth cohort. BMJ (Clinical research ed). 2006;332(7545):820-5.
61. Runge JW. Medical conditions and driver crash risk: Do license restrictions affect public safety? Annals of Emergency Medicine. 2000;36(2):164-5.
62. Schlenker MB, Thiruchelvam D, Redelmeier DA. Association of Cataract Surgery With Traffic Crashes. JAMA ophthalmology. 2018;136(9):998-1007.
63. Sims RV, Owsley C, Allman RM, Ball K, Smoot TM. A preliminary assessment of the medical and functional factors associated with vehicle crashes by older adults. Journal of the American Geriatrics Society. 1998;46(5):556-61.
64. Szlyk JP, Pizzimenti CE, Fishman GA, Kelsch R, Wetzel LC, Kagan S, et al. A comparison of driving in older subjects with and without age-related macular degeneration. Archives of ophthalmology (Chicago, Ill : 1960). 1995;113(8):1033-40.
65. Tanabe S, Yuki K, Ozeki N, Shiba D, Abe T, Kouyama K, et al. The association between primary open-angle glaucoma and motor vehicle collisions. Investigative ophthalmology & visual science. 2011;52(7):4177-81.
66. Wood JM, Black AA, Mallon K, Kwan AS, Owsley C. Effects of Age-Related Macular Degeneration on Driving Performance. Investigative ophthalmology & visual science. 2018;59(1):273-9.
67. Wood JM, Black AA, Mallon K, Thomas R, Owsley C. Glaucoma and Driving: On-Road Driving Characteristics. PloS one. 2016;11(7):e0158318.
68. Yuki K, Asaoka R, Tsubota K. The relationship between central visual field damage and motor vehicle collisions in primary open-angle glaucoma patients. PloS one. 2014;9(12):e115572.
69. Yuki K, Awano-Tanabe S, Ono T, Shiba D, Murata H, Asaoka R, et al. Risk Factors for Motor Vehicle Collisions in Patients with Primary Open-Angle Glaucoma: A Multicenter Prospective Cohort Study. PloS one. 2016;11(11):e0166943.
70. Adler G, Bauer MJ, Rottunda S, Kuskowski M. Driving habits and patterns in older men with glaucoma. Social work in health care. 2005;40(3):75-87.
71. Deshmukh AV, Murthy GJ, Reddy A, Murthy PR, Kattige J, Murthy VR. Older Drivers and Glaucoma in India: Driving Habits and Crash Risks. Journal of glaucoma. 2019;28(10):896-900.
72. Naredo Turrado J, Orriols L, Contrand B, Zins M, Salmi LR, Lafont S, et al. Chronic medical conditions and their association with crash risk and changes in driving habits: a prospective study of the GAZEL cohort. Injury prevention : journal of the International Society for Child and Adolescent Injury Prevention. 2020((Lafont) Universite Lyon, IFSTTAR, Universite Lyon 1, Bron, France).
73. Rubin GS, Ng ESW, Bandeen-Roche K, Keyl PM, Freeman EE, West SK. A prospective, population-based study of the role of visual impairment in motor vehicle crashes among older drivers: the SEE study. Investigative ophthalmology & visual science. 2007;48(4):1483-91.

For peer review only - http://bmjopen.bmj.com/site/about/guidelines.xhtml

74. Ahmed M, Patnaik JL, Whitestone N, Hossain MA, Alauddin M, Husain L, et al. Visual Impairment and Risk of Self-Reported Road Traffic Crashes Among Bus Drivers in Bangladesh. Asia-Pacific journal of ophthalmology (Philadelphia, Pa). 2022;11(1):72-8.
75. Alvarez-Peregrina C, Martinez-Perez C, Villa-Collar C, Sánchez-Tena M. Influence of Vision on Drivers: A Pilot Study. International journal of environmental research and public health. 2021;18(22).
76. Crizzle AM, Toxopeus R, Alphonsus K. Medical Conditions and Crash Risk in Commercial Motor Vehicle Drivers. Journal of occupational and environmental medicine. 2020;62(8):628-33.
77. Piyasena P, Olvera-Herrera VO, Chan VF, Clarke M, Wright DM, MacKenzie G, et al. Vision impairment and traffic safety outcomes in low-income and middle-income countries: a systematic review and meta-analysis. The Lancet Global health. 2021;9(10):e1411-e22.
78. Swain TA, McGwin G, Jr., Wood JM, Antin JF, Owsley C. Naturalistic Driving Techniques and Association of Visual Risk Factors With At-Fault Crashes and Near Crashes by Older Drivers With Vision Impairment. JAMA ophthalmology. 2021;139(6):639-45.
79. Swain TA, McGwin G, Jr., Wood JM, Owsley C. Motion perception as a risk factor for motor vehicle collision involvement in drivers ≥ 70 years. Accid Anal Prev. 2021;151:105956.
80. Vofo BN, Ayuk DA, Pe'er J, Chichom-Mefire A, Tendongfor N, Nche EN. Visual assessment of commercial drivers in the South West Region of Cameroon. BMC Ophthalmol. 2021;21(1):148.
81. Wedenoja J, Kalsi J, Salenius S, Parkkari K, Kaarniranta K, Tervo T. The role of vision-related problems in fatal road accidents in Finland. Acta Ophthalmol. 2021;99(4):427-30.
82. Anstey KJ, Windsor TD, Luszcz MA, Andrews GR. Predicting driving cessation over 5 years in older adults: Psychological well-being and cognitive competence are stronger predictors than physical health. Journal of the American Geriatrics Society. 2006;54(1):121-6.
83. Bressler NM, Chang TS, Varma R, Suner I, Lee P, Dolan CM, et al. Driving ability reported by neovascular age-related macular degeneration patients after treatment with ranibizumab. Ophthalmology. 2013;120(1):160-8.
84. Bressler NM, Varma R, Mitchell P, Suner IJ, Dolan C, Ward J, et al. Effect of Ranibizumab on the Decision to Drive and Vision Function Relevant to Driving in Patients With Diabetic Macular Edema: Report From RESTORE, RIDE, and RISE Trials. JAMA ophthalmology. 2016;134(2):160-6.
85. Campbell MK, Bush TL, Hale WE. Medical conditions associated with driving cessation in community- dwelling, ambulatory elders. Journals of Gerontology. 1993;48(4):S230-S4.
86. DeCarlo DK, Scilley K, Wells J, Owsley C. Driving habits and health-related quality of life in patients with age-related maculopathy. Optometry and vision science : official publication of the American Academy of Optometry. 2003;80(3):207-13.
87. Edwards JD, Ross LA, Ackerman ML, Small BJ, Ball KK, Bradley S, et al. Longitudinal predictors of driving cessation among older adults from the ACTIVE clinical trial. The journals of gerontology Series B, Psychological sciences and social sciences. 2008;63(1):P6-12.
88. Freeman EE, Munoz B, Turano KA, West SK. Measures of visual function and time to driving cessation in older adults. Optometry and vision science : official publication of the American Academy of Optometry. 2005;82(8):765-73.
89. Gallo JJ, Rebok GW, Lesikar SE. The driving habits of adults aged 60 years and older. Journal of the American Geriatrics Society. 1999;47(3):335-41.
90. Garre-Olmo J, Pujol XP, Garriga OT, Lopez-Pousa S, Vilalta-Franch J. Biopsychosocial differences between drivers and non-drivers over the age of 74. Archives of gerontology and geriatrics. 2009;49(3):355-9.
91. Gilhotra JS, Mitchell P, Ivers R, Cumming RG. Impaired vision and other factors associated with driving cessation in the elderly: the Blue Mountains Eye Study. Clinical & experimental ophthalmology. 2001;29(3):104-7.
92. Goh YW, Ang GS, Azuara-Blanco A. Lifetime visual prognosis of patients with glaucoma. Clinical & experimental ophthalmology. 2011;39(8):766-70.

For peer review only - http://bmjopen.bmj.com/site/about/guidelines.xhtml

93. Hajek A, Brettschneider C, Eisele M, van den Bussche H, Wiese B, Mamone S, et al. Prevalence and determinants of driving habits in the oldest old: Results of the multicenter prospective AgeCoDe-AgeQualiDe study. Archives of gerontology and geriatrics. 2019;82(8214379, 7ax):245-50.
94. Huisingh C, McGwin G, Jr., Owsley C. Association of visual sensory function and higher-order visual processing skills with incident driving cessation. Clinical & experimental optometry. 2016;99(5):441-8.
95. Janz NK, Musch DC, Gillespie BW, Wren PA, Niziol LM, Collaborative Initial Glaucoma Treatment Study I. Evaluating clinical change and visual function concerns in drivers and nondrivers with glaucoma. Investigative ophthalmology & visual science. 2009;50(4):1718-25.
96. Keay L, Munoz B, Turano KA, Hassan SE, Munro CA, Duncan DD, et al. Visual and cognitive deficits predict stopping or restricting driving: the Salisbury Eye Evaluation Driving Study (SEEDS). Investigative ophthalmology & visual science. 2009;50(1):107-13.
97. Keay L, Palagyi A, Do V, White A, Lamoureux E, Ivers RQ, et al. Vision and driving status of older Australians with cataract: an investigation of public hospital waiting lists. Clinical & experimental optometry. 2016;99(5):449-55.
98. Levecq L, De Potter P, Jamart J. Visual acuity and factors influencing automobile driving status in 1,000 patients age 60 and older. Graefe's archive for clinical and experimental ophthalmology = Albrecht von Graefes Archiv fur klinische und experimentelle Ophthalmologie. 2013;251(3):881-7.
99. MacLeod KE, Satariano WA, Ragland DR. The Impact of Health Problems on Driving Status among Older Adults. Journal of transport & health. 2014;1(2):86-94.
100. Marottoli RA, Ostfeld AM, Merrill SS, Perlman GD, Foley DJ, Cooney LM, Jr. Driving cessation and changes in mileage driven among elderly individuals. Journal of gerontology. 1993;48(5):S255-60.
101. Monestam E, Lundquist B, Wachtmeister L. Visual function and car driving: longitudinal results 5 years after cataract surgery in a population. The British journal of ophthalmology. 2005;89(4):459-63.
102. Monestam E, Wachtmeister L. Impact of cataract surgery on car driving: a population based study in Sweden. The British journal of ophthalmology. 1997;81(1):16-22.
103. Ramulu PY, West SK, Munoz B, Jampel HD, Friedman DS. Driving cessation and driving limitation in glaucoma: the Salisbury Eye Evaluation Project. Ophthalmology. 2009;116(10):1846-53.
104. Ross LA, Anstey KJ, Kiely KM, Windsor TD, Byles JE, Luszcz MA, et al. Older drivers in Australia: trends in driving status and cognitive and visual impairment. Journal of the American Geriatrics Society. 2009;57(10):1868-73.
105. Segal-Gidan F, Varma R, Salazar X, Mack WJ. Factors influencing driving status in an older Latino population. Journal of aging and health. 2010;22(3):332-47.
106. Sengupta S, van Landingham SW, Solomon SD, Do DV, Friedman DS, Ramulu PY. Driving habits in older patients with central vision loss. Ophthalmology. 2014;121(3):727-32.
107. Stafford WR. Traditional antiglaucoma therapy: A clinical survey of side effects. Current Therapeutic Research - Clinical and Experimental. 1981;29(2):265-74.
108. Stewart RB, Moore MT, Marks RG, May FE, Hale WE. Driving cessation in the elderly: An analysis of symptoms, diseases, and medications. Journal of Geriatric Drug Therapy. 1993;8(2):45-60.
109. Takahashi A, Yuki K, Awano-Tanabe S, Ono T, Shiba D, Tsubota K. Association between glaucoma severity and driving cessation in subjects with primary open-angle glaucoma. BMC ophthalmology. 2018;18(1):122.
110. Tam ALC, Trope GE, Buys YM, Yang Y, Shen C, Jin Y-P. Self-perceived Impact of Glaucomatous Visual Field Loss and Visual Disabilities on Driving Difficulty and Cessation. Journal of glaucoma. 2018;27(11):981-6.
111. van Landingham SW, Hochberg C, Massof RW, Chan E, Friedman DS, Ramulu PY. Driving patterns in older adults with glaucoma. BMC ophthalmology. 2013;13(100967802):4.

For peer review only - http://bmjopen.bmj.com/site/about/guidelines.xhtml

112. Zebardast N, Swenor BK, van Landingham SW, Massof RW, Munoz B, West SK, et al. Comparing the Impact of Refractive and Nonrefractive Vision Loss on Functioning and Disability: The Salisbury Eye Evaluation. Ophthalmology. 2015;122(6):1102-10.
113. Kaleem MA, Rajjoub R, Schiefer C, Wall J, Applegate C, Tian J, et al. Characteristics of Glaucoma Patients Attending a Vision Rehabilitation Service. Ophthalmology Glaucoma. 2021;4(6):638-45.
114. Moon S, Park K. The Predictors of Driving Cessation among Older Drivers in Korea. International journal of environmental research and public health. 2020;17(19).
115. Robinson JL, Liu Y, Chen Q. Licensure and Driving Status among Visually Impaired Persons. Optom Vis Sci. 2021;98(12):1334-9.
116. Grundler W, Strasburger H. Visual attention outperforms visual-perceptual parameters required by law as an indicator of on-road driving performance. PloS one. 2020;15(8):e0236147.
117. Subzwari S, Desapriya E, Scime G, Babul S, Jivani K, Pike I. Effectiveness of cataract surgery in reducing driving-related difficulties: a systematic review and meta-analysis. Injury prevention : journal of the International Society for Child and Adolescent Injury Prevention. 2008;14(5):324-8.
118. Fraser ML, Meuleners LB, Lee AH, Ng JQ, Morlet N. Which visual measures affect change in driving difficulty after first eye cataract surgery? Accident; analysis and prevention. 2013;58:10-4.
119. Singletary BA, Do AN, Donnelly JP, Huisingh C, Mefford MT, Modi R, et al. Self-reported vs state-recorded motor vehicle collisions among older community dwelling individuals. Accident; analysis and prevention. 2017;101:22-7.
120. Haddon W, Jr. Energy damage and the ten countermeasure strategies. J Trauma. 1973;13(4):321-31.
121. Nguyen H, Luca Di Tanna G, Coxon K, Brown J, Ren K, Ramke J, et al. Associations between vision impairment and driving performance and the effectiveness of vision-related interventions: A systematic review. Transportation Research Interdisciplinary Perspectives. 2023;17:100753.
122. Strogatz D, Mielenz TJ, Johnson AK, Baker IR, Robinson M, Mebust SP, et al. Importance of Driving and Potential Impact of Driving Cessation for Rural and Urban Older Adults. The Journal of rural health : official journal of the American Rural Health Association and the National Rural Health Care Association. 2020;36(1):88-93.
123. Unsworth CA, Wells Y, Browning C, Thomas SA, Kendig H. To continue, modify or relinquish driving: findings from a longitudinal study of healthy ageing. Gerontology. 2007;53(6):423-31.
124. Bhalla K, Mohan D, O'Neill B. How much would low- and middle-income countries benefit from addressing the key risk factors of road traffic injuries? International journal of injury control and safety promotion. 2020;27(1):83-90.
125. Forjuoh SN. Traffic-related injury prevention interventions for low-income countries. Injury control and safety promotion. 2003;10(1-2):109-18.
126. Staton C, Vissoci J, Gong E, Toomey N, Wafula R, Abdelgadir J, et al. Road Traffic Injury Prevention Initiatives: A Systematic Review and Metasummary of Effectiveness in Low and Middle Income Countries. PloS one. 2016;11(1):e0144971.
127. United Nations Economic Commission for Europe. Road Safety for All: United Nations; 2020 [Available from: https://unece.org/transport/publications/road-safety-all-0.
128. Bergen G, West BA, Luo F, Bird DC, Freund K, Fortinsky RH, et al. How do older adult drivers self-regulate? Characteristics of self-regulation classes defined by latent class analysis. Journal of safety research. 2017;61:205-10.
129. Braitman KA, McCartt AT. Characteristics of older drivers who self-limit their driving. Annals of advances in automotive medicine Association for the Advancement of Automotive Medicine Annual Scientific Conference. 2008;52:245-54.
130. Harmon AC, Babulal G, Vivoda JM, Zikmund-Fisher BJ, Carr DB. Planning for a Nondriving Future: Behaviors and Beliefs Among Middle-Aged and Older Drivers. Geriatrics (Basel, Switzerland). 2018;3(2).

For peer review only - http://bmjopen.bmj.com/site/about/guidelines.xhtml

131. Owsley C, McGwin G, Jr., Phillips JM, McNeal SF, Stalvey BT. Impact of an educational program on the safety of high-risk, visually impaired, older drivers. American journal of preventive medicine. 2004;26(3):222-9.
132. Ross LA, Clay OJ, Edwards JD, Ball KK, Wadley VG, Vance DE, et al. Do older drivers at-risk for crashes modify their driving over time? The journals of gerontology Series B, Psychological sciences and social sciences. 2009;64(2):163-70.
133. Zhang CT, Bowers AR, Savage SW. The Effects of Age, Distraction, and Simulated Central Vision Impairment on Hazard Detection in a Driving Simulator. Optometry and vision science : official publication of the American Academy of Optometry. 2020;97(4):239-48.
134. Cole BL. Protan colour vision deficiency and road accidents. Clinical & experimental optometry. 2002;85(4):246-53.
135. Cole BL. Colour blindness and driving. Clinical & experimental optometry. 2016;99(5):484-7.
136. Atchison DA, Pedersen CA, Dain SJ, Wood JM. Traffic signal color recognition is a problem for both protan and deutan color-vision deficients. Human factors. 2003;45(3):495-503.
137. Tagarelli A, Piro A, Tagarelli G, Lantieri PB, Risso D, Olivieri RL. Colour blindness in everyday life and car driving. Acta ophthalmologica Scandinavica. 2004;82(4):436-42.
138. Dhibi M. Road safety determinants in low and middle income countries. International journal of injury control and safety promotion. 2019;26(1):99-107.
139. Court H, McLean G, Guthrie B, Mercer SW, Smith DJ. Visual impairment is associated with physical and mental comorbidities in older adults: a cross-sectional study. BMC medicine. 2014;12:181.

FIGURE LEGENDS

Figure 1 Flow chart of search with papers reporting on MVC and driving cessation

Figure 2 Network diagram illustrating strength of association of vision impairment on motor vehicle crashes (MVC) and driving cessation found by narrative summaries

For peer review only

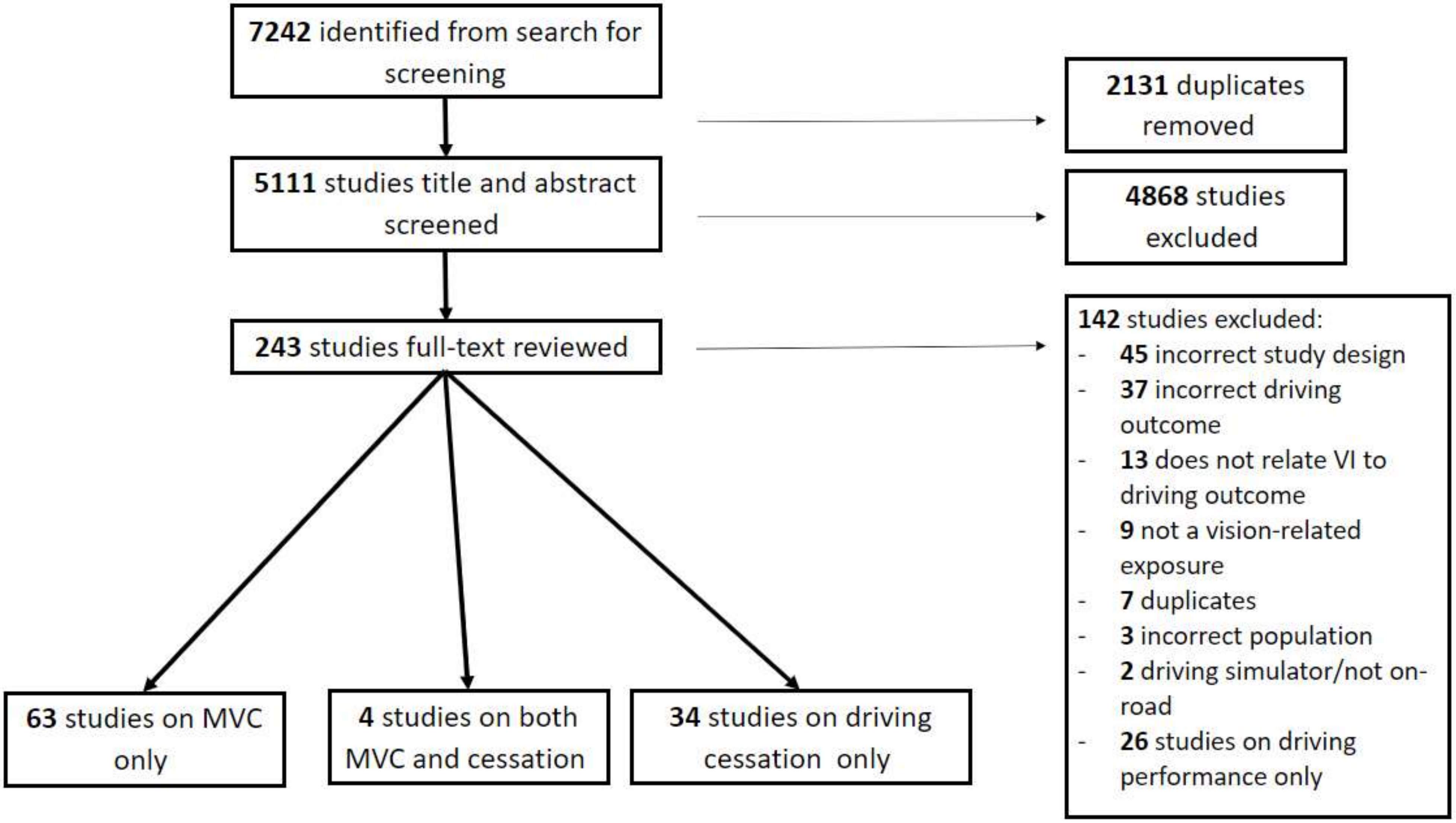

Figure 1 Flow chart of search with papers reporting on MVC and driving cessation

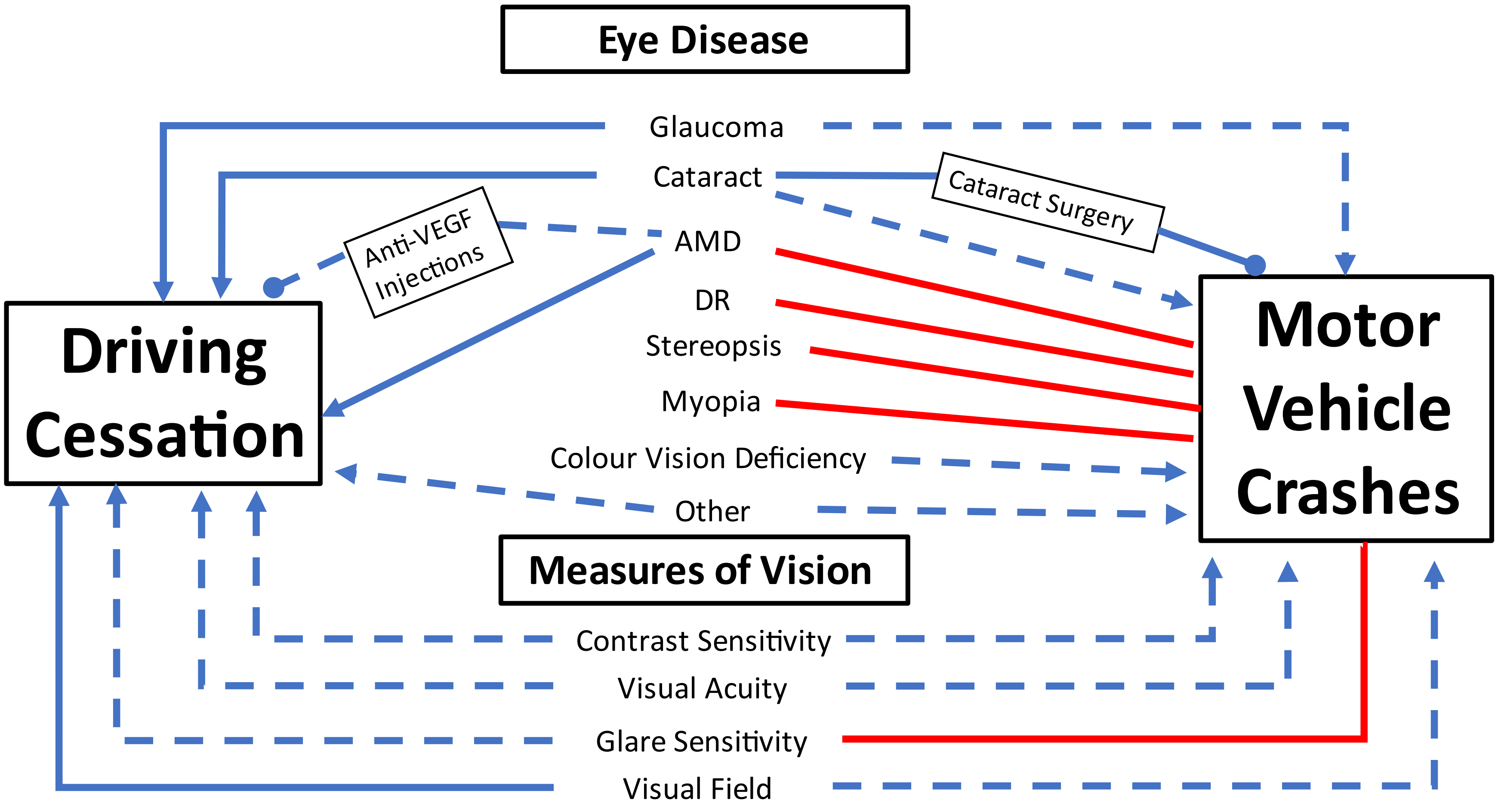

Figure 2 Network diagram illustrating strength of associations of vision impairment on motor vehicle crashes (MVC) and driving cessation found by narrative summaries

Consistent associations of an increased risk of the driving outcome = solid blue line with an arrowhead; Inconsistent associations of either an increased risk or no risk of the driving outcome = dashed blue line with an arrowhead; Consistent associations of a decreased risk of the driving outcome = solid blue line with a closed circle; Inconsistent associations of a decreased risk or no change in risk of the driving outcome = dashed blue line with a closed circle; No associations found with the driving outcome = solid red line

PRISMA 2020 Checklist

Section and Topic	Item #	Checklist item	Location where item is reported
**TITLE**			
Title	1	Identify the report as a systematic review.	pg.1
**ABSTRACT**			
Abstract	2	See the PRISMA 2020 for Abstracts checklist.	Appendix 1
**INTRODUCTION**			
Rationale	3	Describe the rationale for the review in the context of existing knowledge.	pg.4
Objectives	4	Provide an explicit statement of the objective(s) or question(s) the review addresses.	pg.4
**METHODS**			
Eligibility criteria	5	Specify the inclusion and exclusion criteria for the review and how studies were grouped for the syntheses.	pg.4,5, Table 1
Information sources	6	Specify all databases, registers, websites, organisations, reference lists and other sources searched or consulted to identify studies. Specify the date when each source was last searched or consulted.	pg.4
Search strategy	7	Present the full search strategies for all databases, registers and websites, including any filters and limits used.	Appendix 2
Selection process	8	Specify the methods used to decide whether a study met the inclusion criteria of the review, including how many reviewers screened each record and each report retrieved, whether they worked independently, and if applicable, details of automation tools used in the process.	pg.5
Data collection process	9	Specify the methods used to collect data from reports, including how many reviewers collected data from each report, whether they worked independently, any processes for obtaining or confirming data from study investigators, and if applicable, details of automation tools used in the process.	pg.5
Data items	10a	List and define all outcomes for which data were sought. Specify whether all results that were compatible with each outcome domain in each study were sought (e.g. for all measures, time points, analyses), and if not, the methods used to decide which results to collect.	pg.5
	10b	List and define all other variables for which data were sought (e.g. participant and intervention characteristics, funding sources). Describe any assumptions made about any missing or unclear information.	pg.5
Study risk of bias assessment	11	Specify the methods used to assess risk of bias in the included studies, including details of the tool(s) used, how many reviewers assessed each study and whether they worked independently, and if applicable, details of automation tools used in the process.	pg.5,6
Effect measures	12	Specify for each outcome the effect measure(s) (e.g. risk ratio, mean difference) used in the synthesis or presentation of results.	pg.6
Synthesis methods	13a	Describe the processes used to decide which studies were eligible for each synthesis (e.g. tabulating the study intervention characteristics and comparing against the planned groups for each synthesis (item #5)).	pg.6
	13b	Describe any methods required to prepare the data for presentation or synthesis, such as handling of missing summary statistics, or data conversions.	pg.6
	13c	Describe any methods used to tabulate or visually display results of individual studies and syntheses.	pg.6
	13d	Describe any methods used to synthesize results and provide a rationale for the choice(s). If meta-analysis was performed, describe the model(s), method(s) to identify the presence and extent of statistical heterogeneity, and software package(s) used.	pg.6
	13e	Describe any methods used to explore possible causes of heterogeneity among study results (e.g. subgroup analysis, meta-regression).	pg.6
	13f	Describe any sensitivity analyses conducted to assess robustness of the synthesized results.	pg.6
Reporting bias assessment	14	Describe any methods used to assess risk of bias due to missing results in a synthesis (arising from reporting biases).	N/A
Certainty	15	Describe any methods used to assess certainty (or confidence) in the body of evidence for an outcome.	N/A

For peer review only

PRISMA 2020 Checklist

Section and Topic	Item #	Checklist item	Location where item is reported
assessment			
**RESULTS**			
Study selection	16a	Describe the results of the search and selection process, from the number of records identified in the search to the number of studies included in the review, ideally using a flow diagram.	pg.6, Figure 1, Table 2a and 2b
	16b	Cite studies that might appear to meet the inclusion criteria, but which were excluded, and explain why they were excluded.	Figure 1
Study characteristics	17	Cite each included study and present its characteristics.	pg.8-11, Appendix 4a and 4b
Risk of bias in studies	18	Present assessments of risk of bias for each included study.	Appendix 3
Results of individual studies	19	For all outcomes, present, for each study: (a) summary statistics for each group (where appropriate) and (b) an effect estimate and its precision (e.g. confidence/credible interval), ideally using structured tables or plots.	pg.8-11, Appendix 4a and 4b
Results of syntheses	20a	For each synthesis, briefly summarise the characteristics and risk of bias among contributing studies.	pg.8-11, Appendix 4a and 4b
	20b	Present results of all statistical syntheses conducted. If meta-analysis was done, present for each the summary estimate and its precision (e.g. confidence/credible interval) and measures of statistical heterogeneity. If comparing groups, describe the direction of the effect.	pg.8-11, Appendix 4a and 4b
	20c	Present results of all investigations of possible causes of heterogeneity among study results.	pg.8-11, Figure 2a-b
	20d	Present results of all sensitivity analyses conducted to assess the robustness of the synthesized results.	N/A
Reporting biases	21	Present assessments of risk of bias due to missing results (arising from reporting biases) for each synthesis assessed.	N/A
Certainty of evidence	22	Present assessments of certainty (or confidence) in the body of evidence for each outcome assessed.	N/A
**DISCUSSION**			
Discussion	23a	Provide a general interpretation of the results in the context of other evidence.	pg.11-13
	23b	Discuss any limitations of the evidence included in the review.	pg.11-13
	23c	Discuss any limitations of the review processes used.	pg.12,13
	23d	Discuss implications of the results for practice, policy, and future research.	pg.11-13
**OTHER INFORMATION**			
Registration and protocol	24a	Provide registration information for the review, including register name and registration number, or state that the review was not registered.	pg.2
	24b	Indicate where the review protocol can be accessed, or state that a protocol was not prepared.	pg.4
	24c	Describe and explain any amendments to information provided at registration or in the protocol.	N/A
Support	25	Describe sources of financial or non-financial support for the review, and the role of the funders or sponsors in the review.	pg.14
Competing	26	Declare any competing interests of review authors.	pg.14

For peer review only

PRISMA 2020 Checklist

Section and Topic	Item #	Checklist item	Location where item is reported
interests			
Availability of data, code and other materials	27	Report which of the following are publicly available and where they can be found: template data collection forms; data extracted from included studies; data used for all analyses; analytic code; any other materials used in the review.	pg.13

PRISMA ABSTRACT CHECKLIST

Section and Topic	Item #	Checklist item	Reported (Yes/No)
**TITLE**			
Title	1	Identify the report as a systematic review.	Yes
**BACKGROUND**			
Objectives	2	Provide an explicit statement of the main objective(s) or question(s) the review addresses.	Yes
**METHODS**			
Eligibility criteria	3	Specify the inclusion and exclusion criteria for the review.	Yes
Information sources	4	Specify the information sources (e.g. databases, registers) used to identify studies and the date when each was last searched.	Yes
Risk of bias	5	Specify the methods used to assess risk of bias in the included studies.	Yes
Synthesis of results	6	Specify the methods used to present and synthesise results.	Yes
**RESULTS**			
Included studies	7	Give the total number of included studies and participants and summarise relevant characteristics of studies.	Yes
Synthesis of results	8	Present results for main outcomes, preferably indicating the number of included studies and participants for each. If meta-analysis was done, report the summary estimate and confidence/credible interval. If comparing groups, indicate the direction of the effect (i.e. which group is favoured).	Yes
**DISCUSSION**			
Limitations of evidence	9	Provide a brief summary of the limitations of the evidence included in the review (e.g. study risk of bias, inconsistency and imprecision).	Yes
Interpretation	10	Provide a general interpretation of the results and important implications.	Yes
**OTHER**			
Funding	11	Specify the primary source of funding for the review.	Yes
Registration	12	Provide the register name and registration number.	Yes

For peer review only

PRISMA 2020 Checklist

For peer review only

Appendix 2 Complete search strategy for review. While search terms were included for driving performance, driving scores and errors, the studies with outcome measures of driving performance was outside of the scope of this current manuscript and are reported elsewhere.

MEDLINE (OVID) search strategy

1. exp Eye Diseases/
2. exp Cataract Extraction/
3. Lens Implantation, Intraocular/
4. Lenses, Intraocular/
5. cataract$.tw.
6. ((intraocular or intra ocular) adj3 lens$).tw.
7. (IOL or IOLs).tw.
8. Vision Tests/
9. Visual Acuity/
10. exp Refractive Errors/
11. Visual Fields/
12. Visual Field Tests/
13. Contrast Sensitivity/
14. Depth Perception/
15. (visual adj2 (acuit$ or field$)).tw.
16. contrast sensitivity.tw.
17. (depth perception or stereopsis).tw.
18. ((impair$ or decreas$ or declin$) adj3 (vision or visual$ or sight$)).tw.
19. (improv$ adj3 (vision or visual$ or sight$)).tw.
20. ((visual or vision) adj2 function$).tw.
21. exp Vision, Ocular/
22. Vision Screening/
23. or/1-22
24. Mass Screening/
25. ((eye$ or sight or vision or visual$) adj2 (test$ or screen$ or exam$ or diagnos$ or assess$)).tw
26. 24 and 25
27. 23 or 26
28. exp Motor Vehicles/
29. exp Automobile Driving/
30. Accidents, Traffic/
31. (driver$ or driving).tw.
32. (automobile$ or car or cars or vehicle$).tw.
33. (motoring or motorcar or "motor car" or "motor cars").tw.
34. crash$.tw.
35. ((road or traffic) adj2 injur$).tw.
36. ((road or traffic or motor) adj2 (accident$ or incident$)).tw.
37. ((road or traffic or motor) adj2 collision$).tw.
38. or/28-37
39. epidemiologic studies/ or case-control studies/ or cohort studies/ or observational study/ or follow-up studies/ or longitudinal studies/ or prospective studies/ or retrospective studies/ or controlled before-after studies/ or cross-sectional studies/ or historically controlled study/ or interrupted time series analysis/
40. epidemiologic methods/ or focus groups/ or interviews as topic/ or exp "surveys and questionnaires"/

For peer review only

41. epidemiologic research design/ or control groups/ or cross-over studies/ or double-blind method/ or meta-analysis as topic/ or network meta-analysis/ or random allocation/ or single-blind method/
42. epidemiologic methods/ or clinical trials as topic/ or feasibility studies/ or multicenter studies as topic/ or pilot projects/ or sampling studies/ or twin studies as topic/
43. randomized controlled trial/ or controlled clinical trials as topic/ or randomized controlled trials as topic/
44. comparative study/ or evaluation studies/ or meta-analysis/ or review/ or multicenter study/ or "systematic review"/ or validation studies/
45. health surveys/
46. outcome assessment, health care/
47. risk factors/
48. self report/
49. (population or cohort or observation$ or intervention$ or prospective or retrospective or comparative).tw.
50. (questionnaire$ or survey$).tw.
51. (randomized or randomised or randomly or RCT).tw.
52. (systematic review or meta-analysis).tw.
53. (before adj2 after).tw.
54. (case$ adj2 control$).tw.
55. (cross adj1 section$).tw.
56. or/39-55
57. 27 and 38
58. 56 and 57
59. vehicle-controlled.tw.
60. (vehicle adj3 inject$).tw.
61. 59 or 60
62. 58 not 61
63. (animal$ or mouse or mice$ or dog or canine or rat or rats or primate$).ti.
64. (dry eye or cell$ or mutation$ or genes or genome or sequencing).ti.
65. or/63-64
66. 62 not 65
67. limit 66 to english language
68. exp case reports/
69. (case adj2 report$).tw.
70. 68 or 69
71. 67 not 70
72. limit 71 to (editorial or letter)
73. 71 not 72

EMBASE Search Strategy

1. exp eye disease/
2. exp cataract extraction/
3. lens implantation/
4. lens implant/
5. cataract$.tw.
6. ((intraocular or intra ocular) adj3 lens$).tw.
7. (IOL or IOLs).tw.
8. vision test/
9. visual acuity/

For peer review only

10. refractive error/
11. visual field/
12. perimetry/
13. contrast sensitivity/
14. depth perception/
15. (visual adj2 (acuit$ or field$)).tw.
16. contrast sensitivity.tw.
17. (depth perception or stereopsis).tw.
18. ((impair$ or decreas$ or declin$) adj3 (vision or visual$ or sight$)).tw.
19. (improv$ adj3 (vision or visual$ or sight$)).tw.
20. ((visual or vision) adj2 function$).tw.
21. vision/
22. or/1-21
23. mass screening/
24. ((eye$ or sight or vision or visual$) adj2 (test$ or screen$ or exam$ or diagnos$ or assess$)).tw.
25. 23 and 24
26. 22 or 25
27. exp car driving/
28. exp motor vehicle/
29. traffic accident/
30. (driver$ or driving).tw.
31. (automobile$ or car or cars or vehicle$).tw.
32. (motoring or motorcar or "motor car" or "motor cars").tw.
33. crash$.tw.
34. ((road or traffic) adj2 injur$).tw.
35. ((road or traffic or motor) adj2 (accident$ or incident$)).tw.
36. ((road or traffic or motor) adj2 collision$).tw.
37. or/27-36
38. study design/
39. controlled clinical trial/
40. case control study/
41. cohort analysis/
42. observational study/
43. follow up/
44. longitudinal study/
45. prospective study/
46. retrospective study/
47. epidemiology/
48. cross-sectional study/
49. control group/
50. crossover procedure/
51. "meta analysis (topic)"/
52. network meta-analysis/
53. randomization/
54. single blind procedure/
55. double blind procedure/
56. "clinical trial (topic)"/
57. "controlled clinical trial (topic)"/
58. "randomized controlled trial (topic)"/
59. "multicenter study (topic)"/
60. feasibility study/

For peer review only

61. pilot study/
62. comparative study/
63. evaluation study/
64. multicenter study/
65. randomized controlled trial/
66. meta analysis/
67. "systematic review"/
68. validation study/
69. interview/
70. questionnaire/
71. outcome assessment/
72. "systematic review (topic)"/
73. health survey/
74. risk factor/
75. self report/
76. evidence based practice/
77. (population or cohort or observation$ or intervention$ or prospective or retrospective or comparative).tw.
78. (questionnaire$ or survey$).tw.
79. (randomized or randomised or randomly or RCT).tw.
80. (systematic review or meta-analysis).tw.
81. (before adj2 after).tw.
82. (case$ adj2 control$).tw.
83. (cross adj1 section$).tw.
84. or/38-83
85. 26 and 37
86. 84 and 85
87. vehicle-controlled.tw.
88. (vehicle adj3 inject$).tw.
89. or/87-88
90. 86 not 89
91. (animal$ or mouse or mice$ or dog or canine or rat or rats or primate$).ti.
92. (dry eye or cell$ or mutation$ or genes or genome or sequencing).ti.
93. or/91-92
94. 90 not 93
95. limit 94 to conference abstract status
96. 94 not 95
97. limit 96 to english language
98. exp case report/
99. (case adj2 report$).tw.
100. or/98-99
101. 97 not 100
102. limit 101 to (conference paper or "conference review" or editorial or letter or note)
103. 101 not 102

GLOBAL HEALTH Search Strategy

1. exp eye diseases/
2. exp vision disorders/
3. cataract$.tw.
4. ((intraocular or intra ocular) adj3 lens$).tw.
5. (IOL or IOLs).tw.

For peer review only

6. (visual adj2 (acuit$ or field$)).tw.
7. contrast sensitivity.tw.
8. (depth perception or stereopsis).tw.
9. ((impair$ or decreas$ or declin$) adj3 (vision or visual$ or sight$)).tw.
10. (improv$ adj3 (vision or visual$ or sight$)).tw.
11. ((visual or vision) adj2 function$).tw.
12. ((eye$ or sight or vision or visual$) adj2 (test$ or screen$ or exam$ or diagnos$ or assess$)).tw.
13. or/1-12
14. drivers/
15. vehicles/
16. motor cars/
17. traffic/
18. traffic accidents/
19. (driver$ or driving).tw.
20. (automobile$ or car or cars or vehicle$).tw.
21. (motoring or motorcar or "motor car" or "motor cars").tw.
22. crash$.tw.
23. ((road or traffic) adj2 injur$).tw.
24. ((road or traffic or motor) adj2 (accident$ or incident$)).tw.
25. ((road or traffic or motor) adj2 collision$).tw.
26. or/14-25
27. cohort studies/
28. case-control studies/
29. longitudinal studies/
30. retrospective studies/
31. epidemiology/
32. exp clinical trials/
33. randomized controlled trials/
34. feasibility studies/
35. pilot projects/
36. meta-analysis/
37. systematic reviews/
38. reviews/
39. questionnaires/
40. surveys/
41. epidemiological surveys/
42. risk factors/
43. (population or cohort or observation$ or intervention$ or prospective or retrospective or comparative).tw.
44. (questionnaire$ or survey$).tw.
45. (randomized or randomised or randomly or RCT).tw.
46. (systematic review or meta-analysis).tw.
47. (before adj2 after).tw.
48. (case$ adj2 control$).tw.
49. (cross adj1 section$).tw.
50. or/27-49
51. 13 and 26
52. 50 and 51
53. (animal$ or mouse or mice$ or dog or canine or rat or rats or primate$).ti.
54. (dry eye or cell$ or mutation$ or genes or genome or sequencing).ti.
55. 53 or 54

For peer review only

56. 52 not 55
57. limit 56 to english language
58. case reports/
59. (case adj2 report$).tw.
60. 58 or 59
61. 57 not 60
62. limit 61 to (conference or conference paper or conference proceedings or correspondence or editorial or thesis)
63. 61 not 62

For peer review only

Appendix 3 Risk of Bias Assessment for all Included Studies

Analytical Cross-Sectional Study

**Citation**	**Q1 (SB)**	**Q2 (SB)**	**Q3 (DB)**	**Q4 (C)**	**Q5 (C)**	**Q6(C)**	**Q7 (DB)**	**Q8 (V)**	**Risk of Bias***
**High Income Countries**									
Adler G, et al. 2005.	Y	Y	N	Y	Y	Y	Y	Y	L
Alvarez-Peregrina C et al., 2021	N	N	N	N	N	N	Y	N	H
Ball K, et al. 1993.	Y	N	U	U	N	N	U	Y	H
Cohen Y, et al. 2007.	Y	Y	Y	Y	N	N	Y	Y	M
Crizzle AM et al., 2020	Y	Y	U	U	N	N	Y	Y	M
Cross JM, et al. 2009.	U	U	Y	N	N	U	Y	Y	H
DeCarlo DK, et al. 2003.	Y	Y	N	U	U	U	Y	Y	M
Edwards JD, et al. 2008.	Y	Y	Y	N	Y	Y	Y	Y	L
Garre-Olmo J, et al. 2009.	Y	Y	Y	U	Y	Y	Y	Y	M
Gilhotra JS, et al. 2001.	Y	Y	Y	Y	Y	Y	Y	Y	L
Hajek A, et al. 2019.	Y	Y	N	N	Y	Y	Y	Y	M

For peer review only - http://bmjopen.bmj.com/site/about/guidelines.xhtml

Huisingh C, et al. 2015.	Y	Y	Y	Y	Y	Y	Y	Y	L
Ivers RQ, et al. 1999.	N	N	Y	N	Y	Y	N	Y	M
Kaleem MA et al., 2021	Y	Y	Y	Y	N	N	Y	N	M
Keay L, et al. 2016.	Y	Y	Y	Y	Y	Y	Y	Y	L
Kwon M, et al. 2016.	Y	Y	Y	Y	Y	Y	Y	Y	L
Levecq L, et al. 2013.	Y	Y	Y	Y	N	N	Y	Y	M
MacLeod KE, et al. 2014.	Y	Y	Y	Y	Y	Y	Y	Y	L
Marottoli RA, et al. 1993.	Y	Y	N	Y	U	Y	Y	Y	M
Moon SH & Park K et al., 2020	Y	Y	N	N	N	N	Y	Y	M
Ono T, et al. 2015.	Y	Y	Y	Y	Y	Y	N	Y	L
Owsley C, et al. 2001.	Y	Y	Y	Y	Y	Y	Y	Y	L
Owsley C, et al. 2020.	Y	Y	Y	Y	Y	Y	Y	Y	L
Ramulu PY, et al. 2009.	Y	Y	Y	Y	Y	Y	Y	Y	L

For peer review only

Robinson JL et al., 2021	Y	Y	Y	Y	N	N	Y	N	M
Ross LA, et al. 2009.	Y	Y	Y	Y	N	N	Y	Y	M
Runge JW. 2000.	N	N	U	U	U	U	Y	Y	H
Segal-Gidan F, et al. 2010.	Y	Y	Y	Y	Y	Y	Y	Y	L
Sengupta S, et al. 2014.	Y	Y	Y	Y	Y	Y	Y	Y	L
Stafford WR. 1981.	N	N	Y	U	N	N	Y	Y	H
Stewart RB, et al. 1993.	Y	Y	N	N	Y	Y	Y	Y	M
Swain TA et al., 2021	Y	Y	Y	Y	Y	Y	Y	Y	L
Tam ALC, et al. 2018.	Y	Y	Y	Y	N	N/A	Y	Y	M
Tanabe S, et al. 2011.	Y	Y	Y	Y	Y	Y	N	Y	L
van Landingham SW, et al. 2013.	Y	Y	Y	Y	U	U	Y	Y	M
Wedenoja J et al., 2021	Y	Y	U	U	N	N	Y	N	H
Yuki K, et al. 2014.	Y	Y	Y	Y	N	N	N	Y	M

For peer review only - http://bmjopen.bmj.com/site/about/guidelines.xhtml

Zebardast N, et al. 2015.	Y	Y	Y	Y	Y	Y	Y	Y	L
**Low Middle Income Countries**									
Abebe Y, et al. 2002.	Y	Y	Y	N	N	N	N	Y	M
Abraham EG, et al. 2010.	Y	N	N	N	N	N	N	Y	H
Adekoya BJ, et al. 2009.	Y	Y	Y	Y	N	N	N	Y	M
Ahmed M et al., 2021	N	N	Y	Y	Y	Y	Y	Y	M
Bekibele CO, et al. 2007.	N	Y	Y	Y	N	N	N	Y	M
Biza M, et al. 2013.	Y	Y	Y	Y	N	N	N	Y	M
Boadi-Kusi SB, et al. 2016.	Y	Y	N	Y	N	N	N	U	H
Emerole CG, et al. 2013.	N	N	Y	Y	N	N	N	N	H
Humphriss D. 1987.	Y	N	N	N	N	N/A	U	U	H
Isawumi MA, et al. 2011.	Y	Y	U	Y	N	N	N	U	H
Ogbonnaya CE, et al. 2018.	Y	Y	Y	Y	N	N	N	Y	M

For peer review only

Oladehinde MK, et al. 2007.	N	N	Y	Y	N	N	N	Y	H
Ovenseri-Ogomo G, et al. 2011.	Y	Y	Y	Y	N	N/A	N	Y	M
Pepple G, et al. 2014.	Y	Y	Y	N	N	N	N	Y	M
Vofo BN et al., 2021	Y	Y	Y	Y	N	N	Y	N	M

*Risk of bias scores: high (1-3), medium (4-6), and low (7-8)

SB= selection bias, DB= detection bias, C= confounding, V= validity

For peer review only

Case Control Study

**Citation**	**Q1 (SB)**	**Q2 (SB)**	**Q3 (SB)**	**Q4 (DB)**	**Q5 (PB)**	**Q6 (C)**	**Q7 (C)**	**Q8 (PB)**	**Q9 (V)**	**Q10 (V)**	**Risk of Bias***
**High Income Countries**											
Campbell MK, et al. 1993.	Y	U	Y	N	U	Y	Y	Y	U	U	M
Gallo JJ, et al. 1999.	Y	N	Y	U	U	Y	Y	Y	N	Y	M
Gresset JA, et al. 1994.	N	Y	Y	Y	Y	Y	Y	Y	Y	Y	L
Gresset JA, et al. 1994.	U	Y	Y	U	U	N	N	Y	Y	Y	M
McCloskey LW, et al. 1994.	Y	Y	Y	Y	Y	U	U	Y	Y	Y	M

For peer review only - http://bmjopen.bmj.com/site/about/guidelines.xhtml

McGwin G, et al. 2000.	Y	Y	Y	N	Y	Y	Y	Y	Y	Y	L
McGwin G, et al. 2004.	Y	U	Y	Y	Y	Y	Y	Y	Y	Y	L
McGwin G, et al. 2005.	Y	Y	Y	Y	Y	Y	Y	Y	Y	Y	L
Owsley C, et al. 1998.	Y	Y	Y	Y	Y	N	N	Y	Y	Y	M
Sims RV, et al. 1998.	Y	U	Y	Y	Y	N	N	N	Y	Y	M
Szlyk JP, et al. 1995.	Y	Y	Y	Y	Y	N	N	Y	Y	Y	M
Wood JM, et al. 2018.	Y	Y	N	Y	Y	Y	Y	Y	U	Y	M
Wood JM, et al. 2016.	Y	Y	Y	Y	Y	Y	Y	Y	U	Y	L
Owsley C, et al. 1999.	Y	Y	Y	Y	Y	Y	Y	Y	Y	Y	L
**Low Middle Income Countries**											
Deshmukh AV, et al. 2019.	Y	N	Y	Y	Y	N	N	Y	U	N	M

*Risk of bias scores: high (1-4), medium (5-8), and low (9-10)

SB= selection bias, DB= detection bias, PB = performance bias, C= confounding, V= validity

For peer review only

For peer review only - http://bmjopen.bmj.com/site/about/guidelines.xhtml

Case Series

Citation	Q1 (SB)	Q2 (DB)	Q3 (DB)	Q4 (SB)	Q5 (SB)	Q6 (SB)	Q7 (SB)	Q8 (AtB)	Q9 (SB)	Q10 (V)	Risk of Bias*
**High Income Countries**											
Goh YW, et al. 2011	Y	Y	Y	Y	Y	Y	Y	Y	N	Y	L

*Risk of bias scores: high (1-4), medium (5-8), and low (9-10)
SB= selection bias, DB= detection bias, AtB= attrition bias, V= validity

Cohort Study

Citation	Q1 (SB)	Q2 (PB)	Q3 (DB)	Q4 (C)	Q5 (C)	Q6 (SB)	Q7 (DB)	Q8 (V)	Q9 (AtB)	Q10 (AtB)	Q11 (V)	Risk of Bias*
**High Income Countries**												
Anstey KJ, et al. 2006.	Y	Y	Y	Y	Y	Y	Y	Y	Y	Y	Y	L
Baker JM, et al. 2019.	N	Y	N	Y	Y	Y	Y	Y	Y	N/A	Y	M
Fishman GA et al., 1981	Y	U	Y	Y	Y	N	N	U	N	N	N	H
Freeman EE, et al. 2005.	Y	Y	Y	Y	Y	Y	Y	Y	Y	N	Y	L
Green KA, et al. 2013.	Y	Y	Y	Y	Y	U	Y	Y	Y	N	Y	M

For peer review only - http://bmjopen.bmj.com/site/about/guidelines.xhtml

Haymes SA, et al. 2007.	Y	Y	Y	Y	Y	N/A	Y	Y	Y	N/A	Y	M
Huisingh C, et al. 2017.	Y	Y	Y	Y	Y	Y	Y	Y	Y	U	Y	L
Huisingh C, et al. 2016.	Y	Y	Y	Y	Y	Y	Y	Y	N	N	Y	M
Janz NK, et al. 2009.	Y	Y	Y	Y	Y	U	Y	Y	N	N	Y	M
Keay L, et al. 2009.	Y	Y	Y	Y	Y	Y	Y	Y	Y	Y	Y	L
Keeffe JE, et al. 2002.	Y	Y	Y	N	N/A	Y	N	Y	Y	U	U	M
Kristalovich L, et al. 2019.	U	Y	Y	N	N	Y	U	Y	N/A	N/A	Y	M
Maag U, et al. 1997.	Y	N	U	Y	Y	N	Y	Y	Y	N/A	Y	M
Margolis KL, et al. 2002.	Y	Y	Y	Y	Y	Y	Y	Y	Y	U	Y	L
McGwin G, et al. 2015.	Y	Y	Y	Y	Y	Y	Y	Y	Y	U	Y	L
McGwin G, et al. 2013.	Y	Y	Y	N	N/A	Y	Y	Y	U	U	Y	M
Meuleners LB,et al. 2019.	Y	Y	Y	Y	Y	Y	Y	Y	U	Y	Y	L

For peer review only

Meuleners LB, et al. 2012.	Y	Y	Y	Y	Y	Y	Y	Y	Y	N/A	Y	L
Meuleners LB, et al. 2012.	Y	Y	Y	Y	Y	Y	Y	Y	U	U	Y	M
Monestam E, et al. 2005.	Y	Y	Y	N	N	Y	Y	Y	N	N	U	M
Monestam E, et al. 1997.	Y	U	Y	N	N	Y	Y	Y	Y	N	Y	M
Naredo Turrado J, et al. 2020.	Y	Y	Y	Y	Y	U	Y	Y	Y	U	Y	M
Owsley C, et al. 2002.	Y	Y	Y	Y	Y	Y	Y	Y	Y	U	Y	L
Rahi JS, et al. 2006.	Y	Y	U	Y	Y	Y	N	Y	N	N	Y	M
Rubin GS, et al. 2007.	Y	Y	Y	Y	Y	Y	Y	Y	Y	N	Y	L
Schlenker MB, et al. 2018.	Y	Y	Y	Y	Y	Y	Y	Y	Y	U	Y	L
Swain TA et al., 2021	Y	Y	Y	Y	Y	Y	Y	Y	Y	U	Y	L
Takahashi A, et al. 2018.	U	Y	Y	Y	Y	Y	Y	Y	N	N	Y	M
Yuki K, et al. 2016.	Y	Y	Y	N	N/A	Y	N	N	Y	U	N	M

*Risk of bias scores: high (1-4), medium (5-9), and low (10-11)
SB= selection bias, PB= performance bias, DB= detection bias, C= confounding, AtB= attrition bias, V= validity

For peer review only

Systematic Reviews

Citation	Q1 (SB)	Q2 (SB)	Q3 (SB)	Q4 (SB)	Q5 (InB)	Q6 (InB)	Q7 (InB)	Q8 (C)	Q9 (PubB)	Q10 (V)	Q11 (V)	Risk of Bias
**High Income Countries**												
Piyasena P et al., 2021	Y	Y	Y	Y	Y	U	Y	Y	Y	Y	Y	L

Risk of bias scores: high (1-4), medium (5-9), and low (10-11)
SB= selection bias, InB= information bias, C= confounding, PubB= publication bias, V= validity

Randomised Controlled Trials

Citation	Q1 (AlB)	Q2 (PB)	Q3 (SB)	Q4 (PB)	Q5 (PB)	Q6 (AlB)	Q7 (C)	Q8 (AtB)	Q9 (V)	Q10 (DB)	Q11 (DB)	Q12 (V)	Q13 (V)	Risk of Bias
**High Income Countries**														
Bressler NM, et al. 2013.	Y	Y	Y	Y	Y	U	Y	Y	Y	Y	Y	Y	Y	L
Bressler NM, et al. 2016.	Y	Y	Y	Y	Y	Y	Y	Y	Y	Y	Y	Y	Y	L

*Risk of bias scores: high (1-6), medium (7-11), and low (12-13)
AlB = allocation bias, SB= selection bias, PB= performance bias, C= confounding, DB= detection bias, AtB= attrition bias, V= validity

For peer review only - http://bmjopen.bmj.com/site/about/guidelines.xhtml

Appendix 4a Raw data tables and additional narrative summaries of papers on motor vehicle crashes

Table 4a(i) All studies (n=16) on glaucoma and Motor Vehicle Crashes (MVC). Of the 16 studies, 5 studies were suitable for meta-analysis on associations with any MVC involvement and 2 studies on associations with at-fault MVC involvement

Additional Narrative Summary:
Associations between glaucoma and MVCs were mixed in the studies identified. Even though seven controlled studies found glaucoma to increase the odds of any, injurious, and at-fault MVC involvement, two studies found crash involvement to halve in drivers with glaucoma. Only one study looked at not-at-fault crashes, but found no associations (OR 1 (95% CI 0.4-2.5)). Drivers with more severe glaucoma, irrespective of whether it was in the better or worse eye, were involved in more MVCs and also had greater odds of any crash and at-fault crash involvement compared to drivers without glaucoma and drivers with mild glaucoma.

Author and Year	Study Design	Participants/ Sample Size	Mean Age	Country	VI Definition	Comparator	Outcome Measure (OR, RR, HR, etc?)	Effect Measure (with 95% CI) + any description of results (if appropriate
**Included in Meta-analysis (any MVC involvement)**								
Cross JM et al., 2009	Cross-sectional	3158 (249/2909)	71.9	USA	Self-reported physician diagnosed	Drivers without glaucoma	RR (rate ratio)	**Any MVC**: 1.18 (0.81, 1.72)
Haymes S et al., 2007	Retrospective Cohort	95 (48/47)	69	Canada	Diagnosis from glaucoma specialist, glaucomatous optic disc damage and corresponding visual field damage	Drivers without glaucoma	OR (logistic regression)	**Any MVC**: 6.62 (1.4, 31.23)
Kwon M et al., 2016	Cross-sectional	1899 (206/1693)	age, no.: 70-79 years =	USA	Physician diagnosed	Drivers without glaucoma	RR (rate ratio)	**Any MVC involvement**: 1.65 (1.2, 2.28)

For peer review only

			1358, 80-89 years = 502, 90-98 years = 39					
Naredo Turrado J et al., 2020	Prospective Cohort	11670 (525/11145)	62.4	France	Self-reported physician diagnosed	Drivers without glaucoma	OR	**Any MVC**: 0.93 (0.72, 1.22)
McGwin G Jr et al., 2004	Case Control	691 (576/115)	69.2	USA	ICD-9 codes 365.1 and 265.3	Drivers without glaucoma	RR (relative risk)	**Any MVC**: 0.58 (0.4, 0.83)
**Included in Meta-analysis (at-fault MVC involvement)**								
Cross JM et al., 2009	Cross-sectional	3158 (249/2909)	71.9	USA	Self-reported physician diagnosed	Drivers without glaucoma	RR (rate ratio)	**At-fault MVC**: 0.91 (0.48, 1.72)
Haymes S et al., 2007	Retrospective Cohort	95 (48/47)	69	Canada	Diagnosis from glaucoma specialist, glaucomatous optic disc damage and corresponding visual field damage	Drivers without glaucoma	OR (logistic regression)	**At-fault MVC**: 12.44 (1.08, 143.99)
McGwin G Jr et al., 2004	Case Control	691 (576/115)	69.2	USA	ICD-9 codes 365.1 and 265.3	Drivers without glaucoma	RR (relative risk)	**At-fault MVC**: 0.99 (0.54, 1.8)
**Included in Narrative Summaries Only – High Income Countries**								
Adler G et al., 2004	Cross-sectional	199 (52/147)	71.3	USA	Open-or closed-angle glaucoma	Drivers without glaucoma	Prevalence (%)	25% (13/52) of drivers with glaucoma had been in an MVC compared to

For peer review only

								25.9% (38/147) of drivers without glaucoma (p= 0.86).
Cross JM et al., 2009	Cross-sectional	3158 (249/2909)	71.9	USA	Self-reported physician diagnosed	Drivers without glaucoma	RR (rate ratio)	**Injurious MVC**: 0.63 (0.19, 2.06)
Haymes S et al., 2007	Retrospective Cohort	95 (48/47)	69	Canada	Diagnosis from glaucoma specialist, glaucomatous optic disc damage and corresponding visual field damage	Drivers without glaucoma	OR (logistic regression)	**Any MVC (state-reported)**: 3.21 (0.72, 14.27)
								**At-fault MVC (state-reported)**: 7.21 (0.46, 113.4)
							Prevalence (%)	27% (11/400 of drivers with glaucoma had been involved in an MVC compared to 7% (3/44) in drivers without glaucoma.<br><br>20% (8/40) of drivers with glaucoma were at-fault in an MVC compared to 2% (1/44) in drivers without glaucoma.

For peer review only

Kwon M et al., 2016	Cross-sectional	1899 (206/1693)	age, no.: 70-79 years = 1358, 80-89 years = 502, 90-98 years = 39	USA	Physician diagnosed	Drivers without glaucoma	Prevalence (%)	18% (37/206) of drivers with glaucoma were at-fault in a crash compared to 13% (219/1693) of drivers without glaucoma.
McCloskey L et al., 1994	Case Control	683 (42/641)	age, no.: 65-69 years = 264, 70-74 years = 195, 75-79 years = 138, 80+ years = 86	USA	Physician diagnosed (hospital data)	Age-matched drivers with glaucoma who have not been injured in a police-reported MVC in the same calendar year as their matched case.	RR (relative risk)	**Injurious MVC**: 1.5 (0.8, 2.9)*
							Prevalence (%)	7.7% (18/234) of all drivers who had an injurious crash also had glaucoma.
McGwin G Jr et al., 2000	Case Control	901 (447/454)	N/A	USA	Self-reported physician diagnosed	Not-at-fault drivers involved in crashes, without glaucoma	OR	**Not at-fault MVC**: 1 (0.4, 2.5)
							Prevalence (%)	5.2% (10/198) of not-at-fault crashes involved drivers with

For peer review only

								glaucoma. 6.9% (17/249) 0f at-fault crashes involved drivers with glaucoma.
McGwin G Jr et al., 2004	Case Control	691 (576/115)	69.2	USA	ICD-9 codes 365.1 and 265.3	Drivers without glaucoma	RR (relative risk)	**All MVC per person-time**: 0.57 (0.39, 0.83)
								**At-fault MVC per person-time**: 1.02 (0.56, 1.87)
							Prevalence (%)	27% (153/576) of drivers with glaucoma were involved in an MVC compared to 37% (42/115) of drivers without glaucoma.<br><br>15% (87/576) of drivers with glaucoma were at-fault in a crash compared to 12% (14/115) of drivers without glaucoma.
McGwin G Jr et al., 2005	Case Control	240 (120/120)	72.9	USA	ICD-9 codes 365.1 and 265.3, given an AGIS score	Drivers with glaucoma who have not had	OR	**At-fault MVC**: 1.7(0.7, 3.7)

For peer review only

					from visual fields examinations – **mild defect in better eye**	an MVC between 1994 and 2000.		
					ICD-9 codes 365.1 and 265.3, given an AGIS score from visual fields examinations **– moderate defect in better eye**		OR	**At-fault MVC:** 2 (0.7, 5.4)
					ICD-9 codes 365.1 and 265.3, given an AGIS score from visual fields examinations – **severe defect in better eye**		OR	**At-fault MVC:** 4.2 (0.9, 15.3)
					ICD-9 codes 365.1 and 265.3, given an AGIS score from visual fields examinations – **mild defect in worse eye**		OR	**At-fault MVC:** 1.9 (0.6, 6.1)
					ICD-9 codes 365.1 and 265.3, given an AGIS score from visual fields examinations – **moderate defect in worse eye**		OR	**At-fault MVC:** 4.2 (1.2, 15)

For peer review only

					ICD-9 codes 365.1 and 265.3, given an AGIS score from visual fields examinations – **severe defect in worse eye**		OR	**At-fault MVC:** 9 (2.4, 33.2)
					ICD-9 codes 365.1 and 265.3, given an AGIS score from visual fields examination – **moderate bilateral defect**		OR	**Any MVC:** 3.6 (1.4, 9.4)
					ICD-9 codes 365.1 and 265.3, given an AGIS score from visual fields examination – **severe bilateral defect**		OR	**Any MVC:** 4.4 (1.6, 12.4)
Owsley C et al., 1998	Case Control	294 (179/155)	71	USA	Physician diagnosed	Drivers without glaucoma	OR	**Injurious MVC**: 3.6 (1.2, 10.9)*
								**At-fault MVC**: 1.5 (0.5, 4.8)*
							Prevalence (%)	14.1% (11/78) of all injurious crash drivers had glaucoma.<br>6.3% (6/101) of all non-injurious crash drivers had glaucoma.

For peer review only - http://bmjopen.bmj.com/site/about/guidelines.xhtml

Ono T et al., 2015	Cross-sectional	386 (199/187)	64.7	Japan	**Mild POAG in the worse eye** as a visual field defect corresponding to a mean deviation (MD) of −6 dB or better	Drivers without glaucoma	OR (logistic regression)	**Any MVC**: 1.07 (0.55, 2.1)*
					**Moderate POAG in the worse eye** as an MD between −6 and −12 dB		OR (logistic regression)	**Any MVC**: 1.44 (0.68, 3.08)*
					**Severe POAG in the worse eye** as an MD of −12 dB or worse		OR (logistic regression)	**Any MVC**: 2.28 (1.07, 4.88)*
					**Mild POAG in the better eye** as a visual field defect corresponding to a mean deviation (MD) of −6 dB or better		OR (logistic regression)	**Any MVC**: 1.36 (0.78, 2.37)*
					**Moderate POAG in the better eye** as an MD between −6 and −12 dB		OR (logistic regression)	**Any MVC**: 1.82 (0.65, 5.11)*
					**Severe POAG in the better eye** as an MD of −12 dB or worse		OR (logistic regression)	**Any MVC**: 1.65 (0.39, 6.87)*

For peer review only

					Physician diagnosis of POAG in any eye		Prevalence (%)	22.6% (45/199) pf drivers with glaucoma have been in an MVC compared to 16% (30/187) of drivers without glaucoma.
Tanabe S et al., 2011	Cross-sectional	265 (121/144)	61.6	Japan	Mild POAG as a visual field defect corresponding to a mean deviation (MD) of -5 dB or better in both eyes, moderate POAG as corresponding to an MD of -5 to -10 dB in the worse eye, severe POAG as an MD of -10 dB or worse in the worse eye	Drivers free of ocular disease	OR	**Any MVC (severe glaucoma)**: 9.9 (2.1, 47.8)
							Prevalence (%)	6% (7/121) of drivers with glaucoma have been involved in an MVC compared to 3.5% (5/144) of drivers without glaucoma.<br><br>When dividing by glaucoma severity, 3.9% (2/51) or moderate and 25% (5/20) of severe glaucoma drivers have been involved in a crash.

For peer review only

Wood J et al., 2016	Case Control	145 (75/70)	72.9	Australia	Visual acuity better than 20/40 with one or both eyes and binocular visual fields with a horizontal extent of at least 110° within 10° above and below the horizontal midline	Age-matched controls without glaucoma	Prevalence (%)	4% (3/75) of glaucoma drivers had an MVC in the past 12 months compared to 6% (4/70) of drivers without glaucoma; difference was not significant (p= 0.64)
								19% (14/75) of drivers with glaucoma had an MVC in the past 5 years compared to 23% (16/70) of drivers without glaucoma; difference was not significant (p=0.56)
Yuki K et al., 2014	Cross-sectional	247 (147/0)	63.7	Japan	Severity categorised using Mills Glaucoma Staging system – **better eye**	Drivers without history of MVC	Prevalence (%)	Amongst drivers with a history of MVCs, 11.8% (6/51), 72.5% (37/51), 9.8% (5/51), and 5.9% (3/51) had a better eye glaucoma

For peer review only

								severity score of 0, 1, 2, 3 or more, respectively. Amongst drivers without a history of MVC, this glaucoma score were: 20.4% (40/196), 65.8% (129/196), 9.2% (18/196), and 4.6% (9/196). The differences between proportion of people assigned these scores in the two MVC groups was not significant (p= 0.86).
					Severity categorised using Mills Glaucoma Staging system – **worse eye**			Amongst drivers with a history of MVCs, 2% (1/51), 47.1% (24/51), 23.5% (5/51) and 5.9% (3/51) had a worse eye glaucoma severity score of 0, 1, 2, 3 or more,

For peer review only

								respectively. Amongst drivers without a history of MVCs, the glaucoma scores were: 2.6% (5/196), 54.6% (107/196), 24.5% (48/196), and 18.3% (36/196), respectively. The differences between proportion of people assigned these scores in the two MVC groups was not significant (p= 0.86).
Yuki K et al., 2016	Prospective Cohort	191 (191/0)	63.7	Japan	Primary open angle glaucoma (POAG)	Drivers with POAG but no history of MVCs.	Prevalence (%)	15% (28/191) of drivers with glaucoma have been involved in an MVC. Of these, 64.3% (18/28) had mild, 14.3% (4/28) has moderate, and 22.4% (6/27) had severe glaucoma.
**Included in Narrative Summaries Only – Low Middle Income Countries**								

For peer review only

Deshmukh AV et al., 2019	Case Control	150 (100/50)	64.5	India	Diagnosed glaucomatous optic nerve head changes and corresponding visual field defects, which satisfied Anderson criterion	Aged-matched (older than 40 years) non-glaucoma controls	Prevalence (%)	12.9% (11/85) of drivers with glaucoma had an MVC in the past 12 months compared to 70% (35/50) of drivers without glaucoma. This significance was significant (p<0.001).

*unadjusted results

For peer review only

Table 4a(ii) All studies (n=8) on cataract and Motor Vehicle Crashes (MVC) with meta-analyses suitable for 2 studies on associations with any MVC involvement

Author and Year	Study Design	Total Participants (exposed/control)	Mean Age/Age Range	Country	VI Definition	Comparator	Outcome Measure (OR, RR, HR, etc.?)	Effect Measure (with 95% CI) + any description of results (if appropriate)
**Included in Meta-analysis (any MVC involvement)**								
Cross JM et al., 2009	Cross-sectional	3158 (1165/1993)	71.9	USA	Self-reported physician diagnosed	Drivers without cataract	RR (rate ratio)	**Any MVC**: 1.21 (0.95, 1.55)
Margolis KL et al., 2002	Prospective Cohort	1416 (370/1046)	71.3	USA	Self-reported physician diagnosed	Drivers without cataracts.	HR	**Any MVC:** 1.1 (0.88, 1.38)
**Included in Narrative Summaries Only – High Income Countries**								
Cross JM et al., 2009	Cross-sectional	3158 (1165/1993)	71.9	USA	Self-reported physician diagnosed	Drivers without cataract	RR (rate ratio)	**Injurious MVC**: 1.5 (0.85, 2.64)
								**At-fault MVC**: 1.01 (0.69, 1.49)
McCloskey L et al., 1994	Case Control	683 (118/672)	age, no.: 65- 69 years = 264, 70-74 years = 195, 75-79 years = 138, 80+ years = 86	USA	Physician diagnosed (hospital data)	Age-matched drivers with cataracts who have not been injured in a police reported MVC in the same calendar year as their matched case.	RR (relative risk)	**Injurious MVC**: 1 (0.7, 1.16)*
							Prevalence (%)	17.9% (42/234) of all injurious MVCs involved drivers with cataract.

For peer review

McGwin G Jr et al., 2000	Case Control	901 (447/454)	N/A	USA	Self-reported physician diagnosed	Not-at-fault drivers without cataract were involved in crashes	OR	**Not-at-fault MVC**: 1.1 (0.7, 1.8)
							Prevalence (%)	35.1% (69/198) of all not-at-fault crashes involved drives with cataract**.** 44.6% of all at-fault crashes involved drivers with cataract.
Naredo Turrado J et al., 2020	Prospective Cohort	11670 (525/11145)	62.4	France	Self-reported physician diagnosed	Drivers without cataract	OR	**Any MVC**: 1.27 (0.91, 1.76)*
Owsley C et al., 1998	Case Control	294 (179/155)	71	USA	Physician diagnosed	Drivers without cataract	OR	**Injurious MVC**: 1 (0.6, 1.8)*
								**Non-injurious MVC**: 1.1 (0.6, 1.8)*
							Prevalence (%)	47.4% (37/78) of injurious MVCs involved drivers with cataracts.
Owsley C et al., 1999	Case Control	384 (279/105)	69.9	USA	Cataract in one or both eyes from clinic notes with VA in one eye of 20/40 or worse and no previous	Drivers without cataract	RR (relative risk)	**At-fault MVC**: 2.46 (1, 6.16)
							X^2 (Chi Square)	The difference between the number of accidents between drivers with cataract and those

For peer review only

					cataract surgery in either eye			without cataract was non-significant (p= 0.19)
Owsley C et al., 2001	Cross-sectional	377 (274/103)	69.9	USA	Best-corrected VA of 20/40 or worse in worse eye eyes	Crash-free drivers	OR (logistic regression)	**Any MVC:** 1.26 (0.28, 5.59)
					Best-corrected VA of 20/40 or worse in better eyes			**Any MVC:** 1.39 (0.42, 4.62)

*unadjusted results

For peer review only

Table 4a(iii) All studies (n=3) on Age-Related Macular Degeneration (AMD) and Motor Vehicle Crashes (MVC) all suitable to only be summarised narratively

Author and Year	Study Design	Total Participants (exposed/ control)	Mean Age/ Age Range	Country	VI Definition	Comparator	Outcome Measure (OR, RR, HR etc.?)	Effect Measure (with 95% CI) + any description of results (if appropriate)
**Included in Narrative Summaries Only – High Income Countries**								
Cross JM et al., 2009	Cross-sectional	3158 (88/2070)	71.9	USA	Self-reported physician diagnosed	Participants without AMD	RR (rate ratio)	**Any MVC**: 0.57 (0.23, 1.39)
								**Injurious MVC**: 0.9 (0.11, 7.44)
								**At-fault MVC**: 0.95 (0.35, 2.56)
McCloskey L et al., 1994	Case Control	683 (25/658)	age, no.: 65-69 years = 264, 70-74 years = 195, 75-79 years = 138, 80+ years = 86	USA	Physician diagnosed (hospital data)	Age-matched drivers with AMD who had not been injured in a police-reported MVC in the same calendar year as their matched case.	RR (relative risk)	**Injurious MVC**: 0.9 (0.4, 2)*
							Prevalence (%)	3.8% (9/234) of drivers with AMD had a history of an MVC.
McGwin G Jr et al., 2013	Retrospective Cohort	205 (142/63)	72.7	USA	AREDS definition for early AMD	Participants without AMD	RR (rate ratio)	**Any MVC**: 0.48 (0.2, 1.18)* **Any MVC per 100 person-**

For peer review only

								**years**: 0.67 (0.32, 1.39)* **Any MVC per 1, 000, 000 person-miles**: 0.73 (0.36, 1.5)*
					AREDS definition for intermediate AMD			**Any MVC**: 0.22 (0.08, 0.64)* **Any MVC per 100 person-years**: 0.34 (0.13, 0.89)* **Any MVC per 1, 000, 000 person-miles**: 0.35 (0.13, 0.91)*
					AREDS definition for severe AMD			**Any MVC**: 0.46 (0.14, 1.54)* **Any MVC per 100 person-years**: 0.93 (0.31, 2.77)* **Any MVC per 1, 000, 000 person-miles**: 1.11 (0.38, 3.19)*
Szlyk et al., 1995	Case Control	21 (10/11)	73.2	USA	Physician diagnosed	Age-similar subjects with normal vision	X^2 (Chi Square)	X2= 4.68 (p<0.03); Age similar controls had more self-

For peer review only

								reported accidents than those with ARMD. The difference between the groups for the numbers of individuals involved in self-reported accidents was significant
						Younger control subjects with normal vision	X^2 (Chi Square)	X2= 8.06 (p=0.01); The number of self-reported accidents was significantly different between the younger control group and the ARMD group with the younger control group having more self-reported accidents.

For peer review only

Wood JM et al., 2018	Case Control	83 (33/50)	75.4	Australia	AREDS definition for late AMD	Aged-matched controls with no AMD	Prevalence with X^2 (Chi Square)	9% (3/33) of drivers with AMD had a crash in the past 12 months compared to 2% (1/50) of control drivers (p=0.28). 30% (10/33) of drivers with AMD had a history of 1 or more crashes in the past 5 years compared to 16% (8/50) of controls drivers (p=0.23).

*unadjusted results

For peer review only

Table 4a(iv) All studies (n=3) on diabetic retinopathy (DR) and Motor Vehicle Crashes (MVC) all suitable to only be summarised narratively

Author and Year	Study Design	Total Participants (Exposed/Control)	Mean Age	Country	VI Definition	Comparator	Outcome Measure (OR, RR, HR, etc.?)	Effect Measure (with 95% CI) + any description of results (if appropriate
**Included in Narrative Summaries Only – High Income Countries**								
Cross JM et al., 2009	Cross-sectional	3158 (98/3060)	71.9	USA	Self-reported physician diagnosed	Drivers without DR	RR (rate ratio)	**Any MVC**: 0.6 (0.26, 1.38)
								**Injurious MVC**: 0.95 (0.18, 4.92)
								**At-fault MVC**: 0.32 (0.08, 1.17)
McGwin G Jr et al., 2000	Case Control	901 (447/454)	N/A	USA	Self-reported physician diagnosed	Drivers without DR involved in not-at-fault crashes	OR	**Not at-fault MVC**: 1.9 (0.3, 10.9)
								**At-fault MVC**: 1.1 (0.3, 3.8)
							Prevalence (%)	1.1% (2/198) of not at-fault crash drivers had DR. 1.6% (3/249) of all at-fault crash drivers had DR.
Owsley C et al., 1998	Case Control	294 (179/155)	71	USA	Physician diagnosed	Drivers without DR	OR	**Non-injurious MVCs**:1 (0.1, 7.5)*
								**Injurious MVCs:** 0.7 (0.1, 8.2)*

*unadjusted results

Table 4a(v) All studies (n=5) on stereopsis impairment and Motor Vehicle Crashes (MVC) with meta-analysis suitable for 3 studies on associations with any MVC involvement

**Author and Year**	**Study Design**	**Participants/ Sample Size**	**Mean Age**	**Country**	**VI Definition**	**Comparator**	**Outcome Measure (OR, RR, HR?)**	**Effect Measure (with 95% CI) + any description of results (if appropriate)**
**Included in Meta-analysis (any MVC involvement)**								
Boadi-Kusi SB et al., 2016	Cross-sectional	520 (80/440)	39.2	Ghana	Physician diagnosed as abnormal.	Drivers with normal stereopsis	OR	**Any MVC:** 0.89 (0.44, 1.8)*
Margolis KL et al., 2002	Prospective Cohort	1416 (N/A)	71.3	USA	Physician diagnosed - distance depth perception per standard deviation change	Drivers with normal stereopsis.	HR	**Any MVC:** 1.01 (0.92, 1.11)
Oladehinde MK et al., 2007	Cross-sectional	215 (11/204)	41.5	Nigeria	Physician diagnosed - Visual acuity of 6/6 - 6/18 was normal, < 6/18 - 6/60 was classified as visual impairment and < 6/60 - 3/60 was classified as severe visual impairment while visual acuity less than 3/60 was classified as blindness.	Drivers with normal stereopsis	RR (risk ratio)	**Any MVC:** 1.45 (0.42, 5.3)*

**Included in Narrative Summaries Only – High Income Countries**								
Alvarez-Peregrina C et al., 2022	Cross-sectional	736 (55/681)	46.4	Spain	Physician diagnosed	Drivers with normal stereopsis.	X^2 (Chi Square)	Stereopsis was not linked with history of MVCs (p> 0.05).
**Included in Narrative Summaries Only – Low Middle Income Countries**								
Boadi-Kusi SB et al., 2016	Cross-sectional	520 (80/440)	39.2	Ghana	Physician diagnosed as abnormal.	Drivers with normal stereopsis	Prevalence (%)	25% (20/30) of drivers with abnormal stereopsis were involved in an MVC.
Humphriss D, 1987	Cross-sectional	366 (N/A)	N/A	South Africa	Visual acuity of at least 6/12 in each eye separately, or if one eye is below 6/12 then the second eye must be 616 or, wearing glasses and seeing binocularly the acuity must be 6/12. A lateral field of vision of 45 degrees is required.	Drivers with better stereopsis	Mean (SD)	Mean vision test score for stereopsis drivers without MVC involvement: 4.128<br><br>Mean vision test score for stereopsis drivers with MVC involvement: 5
Oladehinde MK et al., 2007	Cross-sectional	215 (11/204)	41.5	Nigeria	Physician diagnosed - Visual acuity of 6/6 - 6/18 was normal, < 6/18 - 6/60 was classified as visual	Drivers with normal stereopsis	Prevalence (%)	18.2% (2/11) of all drivers with abnormal stereopsis have been involved in an MVC

For peer review only

					impairment and < 6/60 - 3/60 was classified as severe visual impairment while visual acuity less than 3/60 was classified as blindness.			

*unadjusted results

For peer review only

Table 4a(vi) All studies (n=4) on myopia and Motor Vehicle Crashes (MVC), with 2 studies for meta-analysis

Author and Year	Study Design	Total Participants (exposed/control)	Mean Age	Country	VI Definition	Comparator	Outcome Measure (OR, RR, HR?)	Effect Measure (with 95% CI) + any description of results (if appropriate)
**Included in Meta-analysis (any MVC involvement)**								
Ahmed M et al., 2021	Cross-sectional	700 (62/638)	42.3	Bangladesh	Physician diagnosed	Drivers without myopia but with a history of MVCs	OR	**Any MVC:** 0.5 (0.15, 1.65)*
Boadi-Kusi SB et al., 2016	Cross-sectional	520 (10/510)	39.2	Ghana	Physician diagnosed – spherical power in the better eye of -0.50D or worse	Drivers without myopia but with a history of MVCs	OR	**Any MVC**: 0.99 (0.41, 2.4)*
**Included in Narrative Summaries Only – High Income Countries**								
Cohen Y et al., 2007	Cross-sectional	136 (34/102)	21	Israel	Night myopia: refraction in illumination and in total darkness in both eyes changed by 0.75 D or more	Drivers without night myopia	Fischer's Exact Test	No statistically significant difference in day time crashes between night myopia and normal subjects (p= 0.22).<br>Night myopia drivers had higher night-time crashes than non-night myopia drivers (p=0.044).

McCloskey L et al., 1994	Case Control	683 (235/448)	age, no.: 65- 69 years = 264, 70-74 years = 195, 75-79 years = 138, 80+ years = 86	USA	Physician diagnosed (hospital data)	Age-matched drivers with myopia who have not been injured in a police-reported MVC in the same calendar year as their matched case.	RR (relative risk)	**Injurious MVC**: 0.6 (0.1, 1)*

*unadjusted results

For peer review only

Table 4a(vii) All studies (n=8) on colour vision deficiency (CVD) and Motor Vehicle Crashes (MVC), all suitable to only be summarised narratively due to methodological limitations in non-standardised diagnosis of colour vision deficiencies

Additional Narrative Summary:
One study looking at the different types of colour deficiency found individuals with protan colour deficiency, measured by Hardy-Rand Rittler (HRR) pseudo-isochromatic plates, to report significantly more MVCs than those with deutan colour deficiency (p= 0.034).

**Author and Year**	**Study Design**	**Total Participants (exposed/control)**	**Mean Age/Age Range**	**Country**	**VI Definition**	**Comparator**	**Outcome Measure (OR, RR, HR, etc.?)**	**Effect Measure (with 95% Cl) + any description of results (if appropriate)**
**Included in Narrative Summaries Only – High Income Countries**								
Piyasena P et al., 2021	Systematic review	15394 (254/15140)	39.3	N/A	Physician diagnosed	Drivers without colour deficiencies.	RR	**Any MVC:** 1.36 (1.01, 1.82)*
**Included in Narrative Summaries Only – Low Middle Income Countries**								
Abebe Y et al., 2002	Cross-sectional	1878 (85/1794)	33.5	Ethiopia	Physician diagnosed - Ishihara plates	Drivers without colour deficiencies.	OR	**Any MVC:** 1.94 (1.18, 3.17)
							Prevalence (%)	32% (27/85) of all drivers with colour blindness were involved in an MVC.
Biza M et al., 2013	Cross-sectional	249 (4/245)	33.6	Ethiopia	Physician diagnosed - Ishihara plates	Drivers without colour deficiencies.	OR	**Any MVC:** 2.34 (0.19, 28.58)
							Prevalence (%)	25% (1/4) of all drivers with colour blindness were involved in an MVC.
Boadi-Kusi SB et al., 2016	Cross-sectional	520 (37/483)	39.2	Ghana	Protan colour deficient – Hardy-Rand	Deutan colour deficient	Prevalence (%)	52.9% (9/17) of proton colour blindness drivers

For peer review only

					Rittler (HRR) pseudo-isochromatic plate			were involved in an MVC compared to 30.8% (4/13) of deutan colour blindness drivers; X2= 6.194 (p=0.034)
								35% (13/37) of all colour blind drivers were involved in an MVC.
							X^2 (Chi Square)	Protan colour blind drivers were more likely to report MVCs than deutan colour blind drivers: 6.194 (p= 0.034)
Isawumi MA et al., 2011	Cross-sectional	99 (6/93)	45.9	Nigeria	Physician diagnosed - Ishihara plates	Drivers with an MVC history without colour deficiencies.	X^2 (Chi Square)	X^2= 0.09, p=0.76 No significance between the number of MVC involvement in those with colour blindness and those without.
							Prevalence (%)	33% (2/6) of all drivers with colour blindness

								were involved in an MVC.
Oladehinde MK et al., 2007	Cross-sectional	215 (7/208)	41.5	Nigeria	Physician diagnosed - Ishihara plates	Drivers without a history of MVCs	RR (risk ratio)	**Any MVC:** 1.12 (10.3, 11.5)
							Prevalence (%)	2% (1/57) of all recorded MVCs involved colour blind drivers.
							X^2 (Chi Square)	There were no statistically significant associations between colour vision impairment and RTA: 2.3 (p= 0.1)
Ovenseri-Ogomo G et al., 2011	Cross-sectional	206 (7/199)	39.2	Ghana	VA < 6/18 in the better eye)	Drivers without a history of MVCs	X^2 (Chi Square)	No significance found for MVC involvement in drivers with colour blindness: X^2= 2.142, p=0.344
Pepple G et al., 2014	Cross-sectional	400 (262/138)	37.8	Nigeria	Physician diagnosed - Ishihara plates	Drivers without colour blindness.	RR (did not state test used)	**Any MVC**: 1.23 (p=0.4)*
							Prevalence (%)	56% (10/18) of drivers with colour blindness were involved in an MVC.

*unadjusted results

For peer review only

Table 4a(viii) All studies (n=28) on visual acuity (VA) impairment and Motor Vehicle Crashes (MVC) with meta-analysis suitable for 5 studies on any crash involvement and 2 on at-fault crashes

Additional Narrative Summary:
Results for injurious crashes were mixed; all non-significant. Similarly, there were no significant risks found for non-injurious and at-fault crash involvement, irrespective of worsening VA. One study looking at visual acuity in normal and low luminance also found poor acuity to not be a significant predictor of crash risk in both lighting conditions. A Japanese study, however, found the odds of crashing to increase by 20% in drivers with primary open angle glaucoma (POAG) experiencing worse eye declines of 0.01 LogMAR increments compared to those without VA changes.

Author and Year	Study Design	Total Participants (exposed/control)	Mean Age	Country	VI Definition	Comparator	Outcome Measure (OR, RR, HR?)	Effect Measure (with 95% CI) + any description of results (if appropriate)
**Included in Meta-analysis (any MVC involvement)**								
Green K et al., 2013	Retrospective Cohort	2000 (N/A)	Age, no.: 70-79 years = 1432, 80-89 years = 526, 90-99 years = 40	USA	VA worse than 20/40	Drivers with VA 20/40 or better	RR (rate ratio)	**Any MVC**: 1.04 (0.74, 1.48)
Huisingh C et a., 2017	Prospective Cohort	659 (35/624)	N/A	USA	Distance VA > 0.3logMAR	Drivers with VA 20/40 or better	RR (rate ratio)	**Any MVC:** 0.98 (0.52, 1.84)
Margolis KL et al., 2002	Prospective Cohort	1416 (N/A)	N/A	USA	20/40 or worse	Drivers with VA 20/40 or better	HR	**Any MVC:** 1.14 (0.73, 1.8)
Piyasena P et al., 2021	Systematic Review	15394 (710/14684)	39.3	N/A	Physician diagnosed	Drivers without a vision impairment	RR	**Any MVC:** 1.46 (1.2, 1.78)

For peer review only - http://bmjopen.bmj.com/site/about/guidelines.xhtml

Rubin G et al., 2007	Prospective Cohort	2520 (N/A)	age, no.: 65-69 years = 780, 70-74 years = 829, 77-79 years = 553, 80-85 years = 350	USA	Physician diagnosed – 15-letter loss of visual acuity (0.3 logMAR i.e. VA 20/40)	Drivers with a VA better than 20/40.	HR	**Any MVC:** 1.06 (0.77, 1.68)
**Included in Meta-analysis (at-fault MVC involvement)**								
Green K et al., 2013	Retrospective Cohort	2000 (N/A)	Age, no.: 70-79 years = 1432, 80-89 years = 526, 90-99 years = 40	USA	VA worse than 20/40 = impairment	Drivers with VA better than 20/40	RR (rate ratio)	**At-fault MVC**: 1.08 (0.71, 1.4)
Huisingh C et a., 2017	Prospective Cohort	659 (35/624)	N/A	USA	Distance VA > 0.3logMAR	Drivers with VA 20/40 or better	RR (rate ratio)	**At-fault MVC:** 1.09 (0.58, 2.05
**Included in Narrative Summary Only – High Income Countries**								
Alvarez-Peregrina C et al., 2021	Cross-sectional	736 (548/188)	46.4	Spain	Physician diagnosed - cut-off not defined in study	Drivers without a VA impairment	X^2 (Chi Square)	Poor VA was linked with increased risk of MVCs (p< 0.001).
Cross JM et al., 2009	Cross-sectional	3158 (1323/1835)	71.9	USA	VA worse 20/20 and better 20/40	Those with binocular acuity of 20/20 or better in any MVC	RR (rate ratio)	**Any MVC:** 1 (0.78, 1.29)

For peer review only

					VA worse 20/20 and better 20/40	Those with binocular acuity of 20/20 or better in injurious MVC		**Injurious MVC:** 0.54 (0.28, 1.01)
					VA worse 20/20 and better 20/40	Those with binocular acuity of 20/20 or better in at-fault MVC		**At-fault MVC:** 1.08 (0.72, 1.62)
					VA 20/40 or worse	Those with binocular acuity of 20/20 or better in any MVC		**Any MVC:** 1.24 (0.74, 2.09)
					VA 20/40 or worse	Those with binocular acuity of 20/20 or better in injurious MVC		**Injurious MVC:** 0.55 (0.11, 2.8)
					VA 20/40 or worse	Those with binocular acuity of 20/20 or better in at-fault MVC		**At-fault MVC:** 1.37 (0.66, 2.82)
Gresset J et al., 1994	Case Control	4036 (151/3885)	N/A	Canada	Physician diagnosed poor VA	Those with better VA	OR	**Any MVC:** 0.99 (0.71, 1.4)
							Prevalence (%)	8.4% (118/1400) of those involved in an MVC had poor VA.

Gresset J et al., 1994	Case Control	4021 (N/A)	N/A	Canada	VA equal to 6/12 or 6/15 and normal binocularity	Drivers with VA 20/40 or better	OR	**Any MVC:** 0.97 (0.68, 1.38)*
					VA equal to 6/12 or 6/15 and lack of binocular vision			**Any MVC:** 1.23 (0.88, 1.72)*
Huisingh C et a., 2017	Prospective Cohort	659 (35/624)	N/A	USA	Distance VA > 0.3logMAR	Drivers with VA 20/40 or better	RR (rate ratio)	**Major MVC:** 0.81 (0.29, 2.26)
								**Any MVC:** 1.29 (0.87, 1.93)
		659 (74/585)			Near VA > 0.3 logMAR			**Major MVC:** 1.54 (0.9, 2.63)
								**At-fault MVC:** 1.19 (0.77, 1.85)
Ivers R et al., 1999	Cross-sectional	3654 (N/A)	N/A	Australia	Best eye VA <20/40-20/60	drivers with Best eye VA >/=20/40	Prevalence ratio (PR)	**Any MVC:** 1.3 (0.6, 2.8)
					Best eye VA <20/60	drivers with Best eye VA >/=20/40		**Any MVC:** 1.2 (0.3, 5)
					Right eye VA <20/40-20/60	drivers with Right eye VA >/=20/40		**Any MVC:** 0.7 (0.3, 1.6)
					right eye VA<20/60	drivers with right eye VA >/=20/40		**Any MVC:** 2 (1.2, 3.5)
					left eye VA <20/40-20/60	drivers with left eye VA >/=20/40		**Any MVC:** 1.1 (0.5, 2)

For peer review only

					left eye VA<20/60	drivers with left eye VA >/=20/40		**Any MVC:** 1.1 (0.5, 24)
Keeffe JE et al., 2002	Retrospective Cohort	2594 (N/A)	62.5	Australia	Visual acuity <6/12	Drivers with better vision (>6/12)	X^2 (Chi Square)	People with impaired vision (<6/12) were no more likely to have an accident or to attribute that the accident was the result of impaired vision; X2= 0.175 (p>0.9)
							Prevalence (%)	9.5% (32/339) of participant involved in an MVC had poor VA.
Kwon M et al., 2016	Cross-sectional	1899 (145/1754)	age, no.: 70-79 years = 1358, 80-89 years = 502, 90-98 years = 39	USA	Low VA classified as <20/40 (0.3 logMAR)	Drivers with glaucoma and binocular VA ≥ 20/20	RR (rate ratio)	**Any MVC:** 1.51 (0.55, 4.16)
McCloskey L et al., 1994	Case Control	683	age, no.: 65- 69 years = 264, 70-74	USA	Uncorrected VA of 20/25 or 20/30	Drivers with VA 20/15 or 20/20	RR (relative risk)	**Injurious MVC**: 2.5 (0.8, 7.2)*

For peer review only

			years = 195, 75-79 years = 138, 80+ years = 86		Uncorrected VA of 20/40			**Injurious MVC:** 1.7 (0.6, 5.3)*
					Uncorrected VA 20/50 or 20/60			**Injurious MVC:** 2.4 (0.8, 7.2)*
					Uncorrected VA 20/70 of greater			**Injurious MVC:** 2.1 (0.7, 5.8)*
					Corrected VA 20/25 or 20/30			**Injurious MVC:** 0.7 (0.5, 1.1)*
					Corrected VA 20/40			**Injurious MVC:** 0.6 (0.3, 1.2)*
					Uncorrected VA 20/50 or 20/60			**Injurious MVC:** 0.3 (0.1, 0.9)*
					Uncorrected VA 20/70 of greater			**Injurious MVC:** 4.3 (0.5, 40.3)*
McGwin G Jr et al., 2000	Case Control	901 (104/797)	N/A	USA	Near vision impairment	Not-at-fault drivers involved in crashes without poor near vision	OR	**Not-at-fault MVC:** 1.6 (0.8, 3.3)
							Prevalence (%)	8% (16/198) of not-at-fault MVCs involved drivers with near vision impairment.<br><br>13.2% (33/249) of at-fault MVCs involved drivers

								with near vision impairment.
		901 (339/562)			Far vision impairment	Not-at-fault drivers involved in crashes without poor far vision	OR	**Not-at-fault MVC:** 1.1 (0.7, 1.7)
							Prevalence (%)	36% (71/198) of not-at-fault crashes involved drivers with far vision impairment.<br><br>41% (102/249) of at-fault crashes involved drivers with far vision impairment.
		901 (57/844)			Peripheral vision impairment	Not-at-fault drivers involved in crashes without poor peripheral vision	OR	**Not-at-fault MVC:** 1.6 (0.7, 3.9)
							Prevalence (%)	4.7% (9/198) of not-at-fault crashes involved drivers with peripheral vision impairment.<br><br>8.5% of at-fault crashes

For peer review only

								involved drivers with peripheral vision impairment.
Ono T et al., 2015	Cross-sectional	386 (N/A)	64.7	Japan	BCVA in the **better eye** LogMAR per 0.1 increment	POAG drivers with BCVA in both eyes of 0.7 or more	OR	**Any MVC:** 0.94 (0.87, 1.01)
Owsley C et al., 2001	Cross-sectional	377 (136/241)	69.9	USA	VA 20/25 - 20/30 in better eye	Drivers with VA 20/25 or better in the **better eye**	OR	**At-fault MVC:** 1.88 (0.72, 4.88)
		377 (118/259)			VA 20/35 - 20/50 in better eye			**At-fault MVC:** 2.54 (0.87, 7.47)
		377 (77/300)			worse than VA 20/50 in better eye			**At-fault MVC:** 1.75 (0.45, 6.85)
		377 (51/326)			VA 20/25 - 20/30 in worse eye	Drivers with VA 20/25 or better in the **worse eye**		**At-fault MVC:** 0.19 (0.03, 1.27)
		377 (67/310)			VA 20/35 - 20/50 in worse eye			**At-fault MVC:** 0.82 (0.19, 3.61)
		377 (110/267)			worse than VA 20/50 in worse eye			**At-fault MVC:** 0.74 (0.16, 3.52)
		377 (N/A)			VA impairment defined as worse than 20/50 in only 1 eye	Drivers with no VA impairment (better than VA 20/50)		**At-fault MVC:** 1.35 (0.58, 3.15)

For peer review only

		377 (N/A)			VA impairment defined as worse than 20/50 in both eyes	Drivers with no VA impairment (better than VA 20/50)		**At-fault MVC:** 1.01 (0.29, 3.45)
Owsley C et al., 1998	Case Control	294 (36/258)	71	USA	VA worse than 20/40	Drivers with VA 20/40 or better	OR	**Injurious MVC:** 1.6 (0.6, 3.8)* **Non-injurious MVC:** 1.6 (0.7, 3.6)*
Rubin G et al., 2007	Prospective Cohort	2520 (N/A)	age, no.: 65-69 years = 780, 70-74 years = 829, 77-79 years = 553, 80-85 years = 350	USA	Physician diagnosed – 15-letter loss of visual acuity (0.3 logMAR i.e. VA 20/40)	Drivers with a VA better than 20/40.	HR	**Any MVC (at low luminance):** 1.06 (0.75, 1.47)
Sims RV et al., 1998	Case Control	174 (N/A)	71.1	USA	Physician Diagnosed	Older drivers without crashes in 6 years preceding 1991	Univariate analysis using student t-tests	Mean (SD) VA of those with a history of MVCs was 0.09 (0.31), compared to 0.03 (0.19) in those without a history of MVCs (p= 0.001).
Yuki K et al., 2014	Cross-sectional	247 (N/A)	63.7	Japan	Physician diagnosed as better VA (LogMar)	Drivers with POAG but without a	Unpaired t-test with Benjamini's correction	Differences between the VA of those who had a history of

For peer review only

						history of MVCs		an MVC and those who did not was significant, p= 0.036
					Physician diagnosed as worse VA (logMar)			Differences between the VA of those who had a history of an MVC and those who did not was not significant, p= 0.6
Yuki K et al., 2016	Prospective Cohort	191 (N/A)	63.7	Japan	POAG with 0.01 logMAR increase in **worse eye**	Drivers with POAG but without a history of MVCs	OR	**Any MVC:** 1.2 (1.1, 1.4)*
					POAG with 0.001 increase logMAR in **the better eye**			**Any MVC:** 0.68 (0, 221)*
**Included in Narrative Summaries only – Low Income Countries**								
Adekoya BJ et al., 2009	Cross-sectional	399 (N/A)	44.7	Nigeria	VA 6/9 in the better eye	N/A – looked at all participants	X^2 (Chi Square)	Inadequate VA in the better eye is not associated with MVC involvement in the last 10 years; X2= 0.035 (p= 0.851)

For peer review only

					VA 6/24 in the better eye		X^2 (Chi Square)	Inadequate VA in the second eye is not associated with involvement in RTA in the last 10 years; X2= 0.372 (p= 0.542)
Bekibele CO et al., 2007	Cross-sectional	99 (16/83)	50.1	Nigeria	Presenting vision less than 6/9 and improved with the aid of a minimum of 0.5 Diopter lenses, with VA <6/18	Drivers without refractive error	OR	**Any MVC:** 1.2 (0.4, 3.7)*
Boadi-Kusi SB et al., 2016	Cross-sectional	520 (38/482)	39.2	Ghana	Visual acuity of less than 0.2, either monocularly or binocularly, was classified as poor vision	N/A	X^2 (Chi Square)	No statistically significantly associations between poor vision due to refractive error and MVC involvement: X2= 3.090 (p= 0.388)
Humphriss D, 1987	Cross-sectional	366 (N/A)	N/A	South Africa	Binocular	Better mean vision test scores for binocular VA	Mean (SD)	Drivers involved in accidents were more likely to have worse mean vision test

For peer review only

								scores (10.031) for binocular VA compared to accident-free drivers (10.847), p<0.001
					right eye monocular VA	Better mean vision test scores for right eye monocular VA	Mean (SD)	Drivers involved in accidents more likely to have worse mean vision test scores (9.219) for right eye monocular VA compared to accident-free drivers (10.100), p<0.001
					left eye monocular VA	Better mean vision test scores for left eye monocular VA	Mean (SD)	Drivers involved in accidents more likely to have worse mean vision test scores (9.031) for left eye monocular VA compared to accident-free drivers (10.024), p<0.001

For peer review only

					Worse eye monocular acuity	Better mean vision test scores for worse eye monocular VA	Mean (SD)	Drivers involved in accidents more likely to have worse mean vision test scores for depth perception (4.128) compared to accident-free drivers (5.000), p<0.001
Isawumi MA et al., 2011	Cross-sectional	99 (5/94)	45.9	Nigeria	Poor driving vision if VA <6/12 in either eye	Drivers with an MVC but with normal vision.	X^2 (Chi Square)	MVCs were not directly related with VA and vice versa; X2= 1.6 (p= 0.65)
Oladehinde MK et al., 2007	Cross-sectional	215	41.5	Nigeria	Visual acuity < 6/18 - 6/60 was classified as visual impairment and < 6/60 - 3/60 was classified as severe visual impairment. VA < 3/60 was classified as blindness.	Drivers with VA 20/20 - 20/40	RR (did not state test used)	**Any MVC:** 3.5 (2.38, 5.14)*
Ogbonnaya CE et al., 2018	Cross-sectional	103 (7/96)	43.2	Nigeria	Minimum VA of 6/9 in the	Drivers without vision	X^2 (Chi Square)	The relationship between visual

For peer review only

					better eye and 6/12 in the worse eye of commercial motor vehicle drivers. Visually unfit to drive if VA <6/12 in the poorer eye.	impairment and no MVC history.		acuity fitness for driving and self-reported history of MVC was not statistically significant; X2= 0.05 (p= 0.82).
Ovenseri-Ogomo G et al., 2011	Cross-sectional	206 (14/192)	39.2	Ghana	VA < 6/18 in the better eye	Drivers without a history of MVC	X^2 (Chi Square)	VA not associated with history of MVC involvement; X^2= 5.982 (p=0.05)
Vofo BN et al. 2021	Cross-sectional	207 (51/156)	41.8	Cameroon	VA < 0.5	Drivers with VA > 0.5	Mean (SD)	Drivers with VA < 0.5 had a higher than average number of MVCs (2.91 +/- 1.72) compared to drives with VA > 0.5 (1.01 +/- 1.33).

*unadjusted results

For peer review only

Table 4a(ix) All studies (n=13) on contrast sensitivity (CS) impairment and Motor Vehicle Crashes (MVC), with only two studies suitable for meta-analysis due to different CS cut-off points, type of crash outcome explored and comparators used for each study.

Author and Year	Study Design	Total Participants (exposure/control)	Mean Age	Country	VI Definition	Comparator	Outcome Measure (OR, RR, HR, etc. ?)	Effect Measure (with 95% CI) + any description of results (if appropriate)
**Included in Meta-analysis (any MVC involvement)**								
Huisingh C et al., 2017	Prospective Cohort	659 (291/368)	N/A	USA	CS in better eye (< 1.5)	Drivers with CS ≥ 1.5 in better eye	RR (rate ratio)	**Any MVC**: 1.22 (0.82, 1.81)
Swain TA et al., 2021	Cross-sectional	159 (17/142)	79.3	USA	CS of <1.5 log sensitivity in the worse eye	Drivers with CS of >1.5 log sensitivity in the worse eye	RR	**Any MVC:** 1.5 (0.8, 3.2)
**Included in Narrative Summaries Only – High Income Countries**								
Cross JM et al., 2009	Cross-sectional	3158 (1323/1835)	71.9	USA	CS is >=1.575 and <1.675	Drivers without binocular CS impairments	RR (rate ratio)	**Any MVC:** 0.91 (0.68, 1.23)
								**Injurious MVC:** 0.94 (0.56, 1.58)
								**At-fault MVC**: 0.72 (0.49, 1.05)
					CS is >=1.450 and <1.575	Drivers without binocular CS	RR (cox proportional hazards)	**Any MVC:** 0.72 (0.49, 1.05)
								**Injurious MVC:** 0.71 (0.32, 1.56)
								**At-fault MVC**: 0.87 (0.49, 1.56)
					CS is <1.450	Drivers without binocular CS	RR (cox proportional hazards)	**Any MVC:** 1.01 (0.66, 1.55)
								**Injurious MVC:** 0.49 (0.16, 2.37)

For peer review only

								**At-fault MVC:** 1.27 (0.68, 2.37)
Green K et al., 2013	Retrospective Cohort	2000 (N/A)	Age, no.: 70-79 years = 1432, 80-89 years = 526, 90-99 years = 40	USA	Impairment defined as <1.5 on Pelli-Robson chart.	Drivers without binocular CS impairments	RR (rate ratio)	**Any MVC**; 1.42 (1, 2.02)<br>**At-fault MVC**: 1.52 (0.93, 2.68)
Huisingh C et al., 2017	Prospective Cohort	659 (291/368)	N/A	USA	CS in worse eye (< 1.5)	Drivers with CS ≥ 1.5 in worse eye		**Any MVC:** 1.38 (1.05, 1.81)
					CS in better eye (< 1.5)	Drivers with CS ≥ 1.5 in better eye		**Major crash involvement**: 1.29 (0.77, 2.18)
					CS in worse eye (< 1.5)	Drivers with CS ≥ 1.5 in worse eye		**Major crash involvement:** 1.54 (1.07, 2.23)
					CS in better eye (< 1.5)	Drivers with CS ≥ 1.5 in better eye		**At-fault MVC:** 1.28 (0.84, 1.94)
					CS in worse eye (< 1.5)	Drivers with CS ≥ 1.5 in worse eye		**At-fault MVC: 1.44** (1.08, 1.93)
Ivers R et al., 1999	Cross-sectional	3654 (N/A)	N/A	Australia	Vectorvision CSV-1000 chart: 3 cycle per degree in best eye CS	Reference group ≤ 2 units compared with >2 on a scale of 1-8	PR (Prevalence Ratio)	**Any MVC:** 1.3 (0.7, 2.2)

For peer review only

					Vectorvision CSV-1000 chart: 6 cycle per degree in best eye CS eye CS			**Any MVC:** 1.2 (0.7, 2.1)
					Vectorvision CSV-1000 chart: 12 cycle per degree in best eye CS			**Any MVC:** 1.4 (0.8, 2.3)
					Vectorvision CSV-1000 chart: 18 cycle per degree in best eye CS			**Any MVC:** 1.4 (0.9, 2.3)
					Vectorvision CSV-1000 chart: 3 cycle per degree in right eye CS			**Any MVC:** 1.2 (0.8, 1.9)
					Vectorvision CSV-1000 chart: 6 cycle per degree in right eye CS eye CS			**Any MVC:** 1 (0.6, 1.5)
					Vectorvision CSV-1000 chart: 12 cycle per degree in right eye CS			**Any MVC:** 2 (1.2, 3.1)

For peer review only

					Vectorvision CSV-1000 chart: 18 cycle per degree in right eye CS			**Any MVC:** 1.3 (0.8, 2.2)
					Vectorvision CSV-1000 chart: 3 cycle per degree in left eye CS			**Any MVC:** 1 (0.6, 1.6)
					Vectorvision CSV-1000 chart: 6 cycle per degree in left eye CS eye CS			**Any MVC:** 1.1 (0.6, 1.7)
					Vectorvision CSV-1000 chart: 12 cycle per degree in left eye CS			**Any MVC:** 1.3 (0.8, 2.2)
					Vectorvision CSV-1000 chart: 18 cycle per degree in left eye CS			**Any MVC:** 1.3 (0.8, 2.1)
Kwon M et al., 2016	Cross-sectional	1899 (432/1467)	age, no.: 70-79 years = 1358, 80-89 years =	USA	Pelli-Robson chart measure of ≤ 1.6 log sensitivity was defined as an impairment.	Older drivers with glaucoma, without CS impairment	RR (rate ratio)	**Any MVC**: 0.72 (0.36, 1.42)

For peer review only

			502, 90-98 years = 39					
Margolis KL et al., 2002	Prospective Cohort	1416 (N/A)	71.3	USA	low spatial frequencies per standard deviation change	N/A – looked at MVC information from all participants from 1986-1995	HR	**Any MVC:** 0.99 (0.89, 1.1)
					high spatial frequencies per standard deviation change			**Any MVC:** 0.94 (0.85, 1.04)
Owsley C et al., 1998	Case Control	294 (56/238)	71	USA	Pelli-Robson chart measure of <= 1.5 log sensitivity was defined as an impairment.	Older drivers with log(CS) >1.5	OR	**Injurious MVC:** 0.9 (0.4, 1.8)*
								**Non-injurious MVC:** 0.7 (0.3, 1.3)*
Owsley C et al., 2001	Cross-sectional	377 (274/103)	69.9	USA	CS impairment defined as ≤1.25	Participants with no CS impairment (CS >=1.50)	OR	**At-fault MVC (better eye CS >1.35 – 2.50):** 1.18 (0.41, 3.36)
								**At-fault MVC (better eye CS >1.25-1.35):** 1.21 (0.4, 3.68)
								**At-fault MVC (better eye CS ≤1.25):** 3.78 (1.15, 12.48)

For peer review only

								**At-fault MVC (worse eye CS >1.35 – 2.50):** 3.28 (0.71, 14.17)
								**At-fault MVC (worse eye CS >1.25-1.35):** 4.36 (0.84, 22.7)
								**At-fault MVC (worse eye CS ≤1.25):** 7.86 (1.55, 39.79)
								**At-fault MVC (unilateral CS ≤1.25):** 2.7 (1.16, 6.51)
								**At-fault MVC (bilateral CS ≤1.25):** 5.78 (1.87, 18.86)
Owsley C et al., 2020	Cross-sectional	915 (179/155)	age, no.: 60-69 years = 310, 70-79 years = 396, 80-90 years =	USA	Low photopic area under the log CS function	Drivers with higher photopic peak log sensitivity	RR (rate ratio)	**All MVC:** 0.8 (0.61, 1.04)
								**At-fault MVC:** 0.77 (0.57, 1.03)
					Low photopic peak log sensitivity			**All MVC:** 0.8 (0.61, 1.04)
								**At-fault MVC:** 0.77 (0.58, 1.03)

For peer review only

			200, 90-99 years = 9		Low mesopic area under the log CS function	Drivers with higher mesopic peak log sensitivity		**All MVC:** 1.36 (1.06, 1.72)
								**At-fault MVC:** 1.28 (1.01, 1.63)
					Low mesopic peak log sensitivity			**All MVC**: 1.5 (1.16, 1.93)
								**At-fault MVC:** 1.38 (1.07, 1.78)
Rubin G et al., 2007	Prospective Cohort	2520 (N/A)	age, no.: 65-69 years = 780, 70-74 years = 829, 77-79 years = 553, 80-85 years = 350	USA	Pelli-Robson Chart: 6 letter worsening (worsening of 0.3 logCS units)	N/A – looked at driver with and without MVC in whole population.	HR	**Any MVC when CS < 1.7**: 0.75 (0.49, 1.21)
								**Any MVC when CS ≥ 1.7**: 1.25 (0.44, 5.65)
Swain TA et al., 2021	Prospective Cohort	154 (17/137)	79.3	USA	CS of <1.5 log sensitivity in the worse eye	Drivers with CS of >1.5 log sensitivity in the worse eye	RR	**At-fault or near crash involvement:** 2.7 (1.3, 5.5)

For peer review only

Table 4a(x) All studies (n=20) on visual field (VF) impairment and Motor Vehicle Crashes (MVC) with meta-analysis suitable for only four studies on associations with any MVC involvement

Additional Narrative Summary:
When comparing impairments in the upper, lower, left, right, vertical and horizontal visual fields, impairments on the left side were found to be the most significant predictor of crash involvement (data from US, driving on right side of road).

Author and Year	Study Design	Total Participants (exposure/control)	Mean Age	Country	VI Definition	Comparator	Outcome Measure (OR, RR, HR, etc.?)	Effect Measure (with 95% CI) + any description of results (if appropriate)
**Included in meta-analysis (any MVC involvement)**								
Huisingh C et al., 2015	Cross-sectional	2000 (N/A)	N/A	USA	Bilateral VF impairment	Drivers without visual field impairments	RR (rate ratio)	**Any MVC:** 1.4 (1.07, 1.83)
Oladehinde MK et al., 2007	Cross-sectional	215 (22/193)	41.5	Nigeria	Bilateral VF impairment	Drivers without a MVC history	RR (risk ratio)	**Any MVC:** 1.07 (0.98, 6.73)
Piyasena P et al., 2021	Systematic Review	15394 (337/15057)	39.3	N/A	Physician Diagnosed	Drivers without VF impairment	RR	**Any MVC:** 1.36 (1.25, 1.48)
Swain TA et al., 2021	Cross-sectional	159 (40/119)	79.3	USA	Overall VF loss of ≤ 22.4dB in the worse eye	Drivers with no overall VF loss in the worse eye	RR	**Any MVC:** 1.6 (0.8, 3.1)
**Included in Narrative Summaries Only – High Income Countries**								
Ball K et al., 1993	Cross-sectional	294 (N/A)	71	USA	Sensitivity loss in the 30 to 60 degree region of the visual field	N/A	Spearman's Correlation (r)	VF loss was significantly related to crash frequency however the LISREL model shows that it only has indirect effects

								on crash frequency but direct effects on UFOV which is the most significantly associated variable with crash frequency; 0.26
Huisingh C et al., 2015	Cross-sectional	2000 (N/A)	N/A	USA	Upper field impairments	Drivers without visual field impairments	RR (rate ratio)	**Any MVC:** 1.1 (0.83, 1.44)
					Lower Field Impairments			**Any MVC:** 1.4 (1.07, 1.82)
					Horizontal Meridian Impairments			**Any MVC:** 1.31 (1, 1.72)
					Vertical Meridian Impairments			**Any MVC:** 1.26 (0.97, 1.65)
					Left Side impairments			**Any MVC:** 1.49 (1.15, 1.92)
					Right side impairments			**Any MVC:** 1.16 (0.88, 1.53)
Huisingh C et al., 2017	Prospective Cohort	659 (406/253)	N/A	USA	Peripheral visual field loss at 70 or 85 degrees temporally in **either eye**	Drivers with no visual field loss in either eye	RR (rate ratio)	**Any MVC:** 1.08 (0.8, 1.47)
								**Major MVC:** 1.53 (1.02, 2.29)
								**At-fault MVC:** 0.98 (0.71, 1.37)

For peer review only

		659 (186/473)			Peripheral visual field loss at 70 or 85 degrees temporally in **both eye**	Drivers with no visual field loss in both eyes		**Any MVC:** 1.74 (1.18, 2.56)<br>**Major MVC:** 2.32 (1.4, 3.83)<br>**At-fault MVC:** 0.73 (1.14, 2.61)
Kwon M et al., 2016	Cross-sectional	1899 (N/A)	age, no.: 70-79 years = 1358, 80-89 years = 502, 90-98 years = 39	USA	Overall visual field loss ≤ 22.5 dB	Drivers (with glaucoma) without severe visual field loss.	RR (rate ratio)	**Any MVC:** 2.11 (1.09, 4.09)
					Upper visual field loss ≤ 22.5 dB			**Any MVC:** 2.37 (1.19, 4.74)
					Lower visual field loss ≤ 22.5 dB			**Any MVC:** 2.32 (1.13, 4.75)
					Left visual field loss ≤ 22.5 dB			**Any MVC:** 3.16 (1.55, 6.46)
					Right visual field loss ≤ 22.5 dB			**Any MVC:** 1.63 (0.84, 3.14)
					Horizontal meridian loss ≤ 22.5 dB			**Any MVC:** 1.78 (0.92, 3.44)
					Vertical meridian loss ≤ 22.5 dB			**Any MVC:** 1.09 (0.56, 2.11)
Kristalovich L et al., 2019	Retrospective cohort	445 (286/159)	N/A	Canada	Loss of at least 120 continuous degrees along the horizontal meridian and 15 continuous	Drivers with either no VFI or with VFI but meeting licensing standards	X^2 (Chi Square)	No significant difference in rate of crash between VFI/not meet licensing

For peer review only

					degrees above and below fixation with both eyes open and examined together.			standards and no VFI and VFI/meet licensing standards (p=0.402)
McGwin G Jr et al., 2015	Retrospective Cohort	438 (N/A)	72.8	USA	Binocular visual field **total deviations <7.25**	Drivers (with glaucoma) without severe visual field impairments	RR (rate ratio)	**At-fault MVC:** 1.5 (0.82, 2.74)
					Binocular visual impairment severely **impaired threshold** <20.4			**At-fault MVC:** 1.49 (0.81, 2.74)
					Binocular visual impairment severely impaired **pattern deviation <3.97**			**At-fault MVC:** 2.13 (1.21, 3.75)
Owsley C et al., 1998	Case Control	294 (36/258)	71	USA	Central 30 degree VF sensitivity: >10	Older drivers with central 30 degree VF sensitivity of 0-10	OR	**Injurious MVC:** 2.6 (1.1, 6.3)*
								**Non-injurious MVC:** 1.8 (0.8, 4.4)*
		294 (108/186)			Peripheral 20-60 degree VF sensitivity: >10	Older drivers with peripheral 30-60 degree VF sensitivity of 0-10		**Injurious MVC:** 2.4 (1.3, 4.5)*
								**Non-injurious MVC:** 1.8 (1, 3.1)*
Rubin G et al., 2007	Prospective Cohort	2520 (N/A)	age, no.: 65-69	USA	Binocular visual field <20 (loss of 15 points)	N/A – looked at drivers with and without MVC in	HR	**Any MVC:** 0.59 (0.34, 1)

For peer review only

			years = 780, 70-74 years = 829, 77-79 years = 553, 80-85 years = 350		Binocular visual field >=20 (loss of 15 points)	whole population.		**Any MVC:** 1.31 (1.31, 4.27)
Swain TA et al., 2021	Cross-sectional	159 (41/118)			Peripheral VF loss of ≤ 19.2dB in the worse eye	Drivers with no peripheral VF loss in the worse eye		**Any MVC:** 2.4 (1.3, 4.4)
		159 (41/118)			Superior VL loss of ≤ 22.0dB in the worse eye	Drivers with no superior VF loss in the worse eye		**Any MVC:** 0.7 (0.4, 1.5)
		159 (41/118)			Inferior VL loss of ≤ 22.1dB in the worse eye	Drivers with no inferior VF loss in the worse eye		**Any MVC:** 1.7 (0.4, 1.5)
		159 (40/119)			Left VL loss of ≤ 21.6dB in the worse eye	Drivers with no left VF loss in the worse eye		**Any MVC:** 1.7 (0.9, 3.2)
		159 (41/118)			Right VF loss of ≤ 21.8dB in the worse eye	Drivers with no right VF loss in the worse eye		**Any MVC:** 1.6 (0.9, 3)
Swain TA et al., 2021	Prospective Cohort	154 (38/116)	79.3	USA	Overall VF loss of ≤ 22.4dB in the worse eye	Drivers with no overall VF loss in the worse eye	RR	**At-fault or near crash:** 1.4 (0.8, 2.8)

For peer review only - http://bmjopen.bmj.com/site/about/guidelines.xhtml

		154 (40/114)			Peripheral VF loss of ≤ 19.2dB in the worse eye	Drivers with no peripheral VF loss in the worse eye		**At-fault or near crash:** 1.8 (1, 3.3)
		154 (43/111)			Superior VL loss of ≤ 22.0dB in the worse eye	Drivers with no superior VF loss in the worse eye		**At-fault or near crash:** (1.3 (0.7, 2.5)
		154 (41/113)			Inferior VL loss of ≤ 22.1dB in the worse eye	Drivers with no inferior VF loss in the worse eye		**At-fault or near crash:** (1.4, 0.8, 2.5)
		154 (42/112)			Left VL loss of ≤ 21.6dB in the worse eye	Drivers with no left VF loss in the worse eye		**At-fault or near crash:** 1.3 (0.7, 2.5)
		154 (36/118)			Right VF loss of ≤ 21.8dB in the worse eye	Drivers with no right VF loss in the worse eye		**At-fault or near crash:** 0.9 (0.5, 1.8)
Yuki K et al., 2014	Cross-sectional	247 (N/A)	63.7	Japan	N/A	POAG drivers without a MVC history	Mean (SD)	The mean IVF-MD (db) of glaucoma drivers with a history of MVCS was -0.6 (3.4) compared to -0.8 (3.7) in glaucoma drivers without a history of MVCs.
Yuki K et al., 2016	Prospective Cohort	191 (N/A)	63.7	Japan	POAG with **1dB increase in visual field**	POAG drivers without a MVC history	OR	**Any MVC:** 0.95 (0.8, 1.1)*
							Mean (SD)	Mean (SD) IVF-MD (dB) of glaucoma

For peer review only

								drivers with a history of MVC was -2.1 (3.9) compared to -1.6 (3.7) in glaucoma drivers without a history of MVCs.
**Included in Narrative Summaries Only – Low Middle Income Countries**								
Abraham EG et al., 2010	Cross-sectional	291 (13/278)	41.5	Nigeria	Cup-disc ratio >0.5 cup-disc disparity between the two eyes of up to 0.2 or more, abnormal disc pallor (localised or generalised)	Drivers without visual field impairments.	RR (relative risk)	**Any MVC:** 0.628*
Adekoya BJ et al., 2009	Cross-sectional	399 (21/378)	44.7	Nigeria	Presence of 1 or more abnormal quadrants on confrontation perimetry	N/A	X^2 (Chi Square)	Abnormal visual fields was not associated with MVC involvement in the last 10 years; X2= 1.715 (p= 0.19).
Humphriss D, 1987	Cross-sectional	366 (N/A)	N/A	South Africa	N/A	Drivers with no MVC history	Mean (SD)	Data not reported
Isawumi et al., 2011	Cross-sectional	99 (N/A)	45.9	Nigeria	N/A	Drivers with a MVC but	Prevalence (%)	21.1% (8/38) of drivers with an MVC also had

For peer review only

						without visual field loss		horizontal visual field loss.
Ovenseri-Ogomo G et al., 2011	Cross-sectional	206 (14/192)	39.2	Ghana	VA < 6/18 in the better eye	Drivers without a history of MVC	OR	Any MVC: 0.54 (0.016, 18.45)*
Pepple G et al., 2014	Cross-sectional	400 (16/384)	37.8	Nigeria	Physician diagnosed	Drivers without visual field impairments	RR (did not state test used)	**Any MVC:** 1.25*
							Prevalence (%)	56% (9/16) of those with visual field impairment were have been involved in an MVC.

*unadjusted results

For peer review only

Table 4a(xi) All studies (n=3) on glare sensitivity (GS) impairment and Motor Vehicle Crashes (MVC), all suitable to only be summarised narratively due to their different GS cut-off points, type of crash outcome explored and comparators

Author and Year	Study Design	Total Participants (exposed/control)	Mean Age/ Age Range	Country	VI Definition	Comparator	Outcome Measure (OR, RR, HR, etc.?)	Effect Measure (with 95% CI) + any description of results (if appropriate)
**Included in Narrative Summaries Only – High Income Countries**								
Owsley C et al., 1998	Case Control	294 (71/179)	71	USA	Measured using MCT-8000 (Vis Tech), defined as disability glare >0	Older drivers with disability glare <= 0	OR	**Injurious MVC**: 1.4 (0.8, 2.5)*
								**Non-injurious MVC:** 1.3 (0.9, 2.2)*
Owsley C et al., 2001	Cross-sectional	377 (274/103)	69.9	USA	Glare impairment defined as >=0.25, measured with Pelli-robson chart with BAT:	Those with disability glare <0.25 in the better/worse eye	OR	**At-fault MVC in the better eye:** 0.68 (0.22, 2.12)
					Glare impairment defined as >=0.25 in both eyes	those with no disability glare impairment (<0.25 score)	OR (logistic regression)	**At-fault MVC in the worse eye**: 0.62 (0.29, 1.33)
								**At-fault MVC in both eyes:** 0.46 (0.14, 1.53)
Rubin G et al., 2007	Prospective Cohort	2520 (N/A)	age, no.: 65-69 years = 780, 70-74 years = 829, 77-79 years =	USA	6 letter worsening (worsening of 0.3 logCS units) - measured using Pelli-Robson chart with BAT	N/A – looked at driver with and without MVC in whole population.	HR	**Any MVC (glare <3 letters)**: 0.46 (0.26, 0.89)
								**Any MVC (glare ≥ 3 letters)**: 2.3 (1.14, 16.78)

			553, 80-85 years = 350					

For peer review only

Table 4a(xii) All studies (n=19) on other types of vision impairment and Motor Vehicle Crashes (MVC), all suitable to only be summarised narratively

Author and Year	Study Design	Total Participants (exposure/control)	Mean Age/ Age Range	Country	Type of VI	VI definition	Comparator	Outcome Measure (OR, RR, HR, etc.?)	Effect Measure (with 95% CI) + any description of results (if appropriate)
**Included in Narrative Summaries only – High Income Countries**									
Baker JM et al., 2019	Retrospective Cohort	66253 (62/66191)	20.8	USA	Unilateral vision impairment	ICD-9 diagnostic codes (369.6-369.8)	Young adult drivers without unilateral vision impairment	HR	**Any MVC**: 1.08 (0.6, 1.95)
		66253 (352/65901)			Amblyopia	Using the ICD-9 diagnostic codes (368.00 - 368.03) in the HER with diganosis noted in medical record from age 6	Young adult drivers without amblyopia	HR	**Any MVC**: 1.08 (0.85, 1.38)
Crizzle AM et al., 2020	Cross-sectional	3346 (513/2833)	61.5	Canada	Vision impairment	Physician diagnosed	Drivers without vision impairment	Univariate log rank test	Vision impairment was not associated with history of MVCs (p=0.9178).

For peer review only

Fishman GA et al., 1981	Retrospective Cohort	129 (42/87)	37.3	USA	Retinitis Pigmentosa	Physician diagnosed	Drivers free from ophthalmic or general defects	X^2 (Chi Square)	Statistical significant difference in accidents recorded over 5 years between retinitis pigmentosa patients (50%; 21/42)) and controls (29%; 25/62); p= 0.02
Gresset J et al., 1994	Case Control	4036 (15/4021)	N/A	Canada	Monocularity	Physician diagnosed	Male drivers who had no accident during their 70th year in 1988 and 1989	OR	**Any MVC**: 0.95 (0.32, 2.77)
		4036 (327/3709)			Visual impairment			OR	**Any MVC:** 1.07 (0.84, 1.36)
Maag U et al., 1997	Retrospective Cohort	116 (N/A)	N/A	Canada	Vision impairment	Non stereoscopic vision (> 160 seconds); an acuity of 20/40 for the better eye and zero in the other.	Drivers in good health	Mean (SD)	Average total number of crashes in people with good health with a taxi per year (SD): 0.218 (0.501) Average total number of crashes in people with binocular vision problems with

For peer review only

									taxi per year (SD): 0.369 (0.595); the difference was statistically significant (p= 0.01)
McCloskey L et al., 1994	Case Control	683 (10/673)	age, no.: 65-69 years = 264, 70-74 years = 195, 75-79 years = 138, 80+ years = 86	USA	Retinopathy	Physician diagnosed (hospital data)	Age-matched drivers with retinopathy who have not been injured in a police reported MVC in the same calendar year as their matched case.	RR (relative risk)	**Injurious MVC**: 0.6 (0.1, 2.6)*
		683 (37/646)			Retinal disorders	Physician diagnosed (hospital data)	Age-matched drivers with other retinal disorders who have not been injured in a police	RR (relative risk)	**Injurious MVC**: 0.8 (0.4, 1.6)*

For peer review only

							reported MVC in the same calendar year as their matched case.		
		683 (394/289)			Hypermetropia	Physician diagnosed (hospital data)	Age-matched drivers with hypermetropia who have not been injured in a police reported MVC in the same calendar year as their matched case.	RR (relative risk)	**Injurious MVC**: 0.9 (0.7, 1.4)*
		683 (544/139)			Presbyopia	Physician diagnosed (hospital data)	Age-matched drivers with presbyopia who have not been injured in a police reported MVC in the same calendar year as their	RR (relative risk)	**Injurious MVC**: 1 (0.6, 1.8)*

For peer review only

							matched case.		
		683 (339/344)			Astigmatism	Physician diagnosed (hospital data)	Age-matched drivers with astigmatism who have not been injured in a police reported MVC in the same calendar year as their matched case	RR (relative risk)	**Injurious MVC**: 0.9 (0.7, 1.4)*
		638 (597/41)			Refractive disorder	Physician diagnosed (hospital data)	Age-matched drivers with refractive disorders who have not been injured in a police reported MVC in the same calendar year as their matched case	RR (Mantel-Haenszel)	**Injurious MVC**: 0.3 (0.1, 0.8)*
		638 (6/632)			Monocular vision	Physician diagnosed (hospital data)	Age-matched drivers with monocular vision who have not	RR (relative risk)	**Injurious MVC**: 0.7 (0.1, 4.1)*

For peer review only

							been injured in a police reported MVC in the same calendar year as their matched case		
		638 (10/628)			Diplopia	Physician diagnosed (hospital data)	Age-matched drivers with diplopia who have not been injured in a police reported MVC in the same calendar year as their matched case	RR (relative risk)	**Injurious MVC**: 1.2 (0.4, 4.2)*
		638 (13/625)			Vision/ophthalmic conditions	Physician diagnosed (hospital data)	Age-matched drivers with other vision and opthalmic conditions who have not been injured in a police reported MVC in the same	RR (relative risk)	**Injurious MVC**: 0.6 (0.2, 1.6)*

							calendar year as their matched case		
Naredo Turrado J et al., 2020	Prospective Cohort	11670 (11/11659)	62.4	France	Retinal detachment	Self-reported physician diagnosed	Drivers without retinal detachment	OR	**Any MVC**: 0.99 (0.37, 2.7)
Owsley C et al., 1998	Case Control	294 (N/A)	71	USA	Stereoacuity	Scores ≥ 500 arcseconds on TNO test	Older drivers with stereoacuity <500 arcseconds	OR (logistic regression)	**Injurious MVC**: 2.2 (1.1, 1.4)*<br>**Non-injurious MVC**: 1.2 (0.7, 2.3)*
Pepple G et al., 2014	Cross-sectional	400 (32/368)	37.8	Nigeria	Vision impairment	Physician diagnosed	Drivers without a vision impairment	RR (did not state test used)	**Any MVC**: 0.62 (p= 0.46)
Rubin G et al., 2007	Prospective Cohort	2520 (545/2066)	age, no.: 65-69 years = 780, 70-74 years = 829, 77-79 years = 553, 80-85 years = 350	USA	Stereoacuity	Stereodeficient was defined at failing the test at 457 arc seconds.	Drivers who were not sterodeficient	HR (cox proportional hazard)	**Any MVC**: 1.44 (0,88, 2.27)

For peer review only

Runge JW, 2000	Cross-sectional	N/A	N/A	USA	Vision Impairment	Physician diagnosed	Drivers without vision impairment s	RR (relative risk)	**At-fault MVC**: 1.51*<br><br>The at-fault crash rate of those with a vision impairment was 1.14 compared to those without an impairment (0.75).
Rahi J et al., 2006	Retrospective Cohort	8661 (429/8432)	N/A	UK	Amblyopia	Mild = acuity 6/6 in one eye and 6/9 or 6/12 in the other and unilateral visual loss	People with normal vision in each eye	OR	**Any MVC**: 1.28 (0.87, 1.89)
						Moderate/severe = acuity of 6/6 in one eye and 6/18 or worse in the other and unilateral visual loss, with or without strabismus, earlier in childhood.	People with normal vision in each eye	OR (ordinal regression)	**Any MVC**: 2.33 (1.29, 4.2)
Wedenoja J et al., 2021	Cross-sectional	N/A	N/A	Finland	Vision impairment	Physician diagnosed	Drivers without	Prevalence	Only 1.3% (13/968) of all fatal MVCs were

For peer review only

							vision impairment.		caused by vision-related problems.
**Included in Narrative Summaries Only – Low Middle Income Countries**									
Ahmed M et al., 2021	Cross-sectional	700 (492/208)	42.3	Bangla desh	Near or distance visual impairment	Presenting VA ≥ 6/7.5 in the better eye and or presence of presbyopia.	Drivers without near or distance visual impairment but with a history of MVCs.	OR	**Any MVC:** 2.45 (1.09, 5.49)
		700 (125/575)	42.3	Bangla desh	Hyperopia	Physician diagnosed	Drivers without hyperopia but with a history of MVCs.	OR	**Any MVC:** 1.1 (0.56, 2.23)*
		700 (11/689)	42.3	Bangla desh	Presbyopia	Physician diagnosed	Drivers without presbyopia but with a history of MVCs.	OR	**Any MVC:** 1.7 (0.96, 3.01)*
		700 (N/A)	42.3	Bangla desh	Any distance refractive error	Physician diagnosed	Drivers without any distance refractive error but with a history of MVCs.	OR	**Any MVC:** 1.66 (0.88, 3.12)*
Biza M et al., 2013	Cross-sectional	249 (13/236)	33.6	Ethiop ia	Visual impairment	VA <6/18-6/60 was classified as	Drivers with a MVC but no	OR	**Any MVC (both eyes**

For peer review only - http://bmjopen.bmj.com/site/about/guidelines.xhtml

						moderate visual impairment and <6/60-3/60 was classified as severe VI while VA less than 3/60 was classified as blindness.	VA impairment		**impairment)**:42.82 (2.53, 724.03)<br>**Any MVC (right eye impairment)**: 0.03 (0.004, 0.28)*<br>**Any MVC (left eye impairment)**: 0.09 (0.01, 0.97)*
Boadi-Kusi SB et al., 2016	Cross-sectional	520 (66/454)	39.2	Ghana	Hyperopia	Hyperopia defined as the spherical power in the better eye of +1.00D or more	Drivers with a history of MVC but no hyperopia	OR	**Any MVC**: 0 (0, 0);
		520 (30/490)			Astigmatism	Astigmatism was defined as −0.50D cylinder or worse in the better eye	Drivers with a history of MVC but no astigmatism	OR	**Any MVC**: 0.885 (0.32, 2.5)*
Emerole C et al., 2013	Cross-sectional	280 (102/178)	N/A	Nigeri a	Vision impairment causing poor visibility.	Physician diagnosed with VA of 6/30 classified as abnormal.	N/A – compared with a "control" group but paper never explained what/who	Prevalence (%)	119 (79.3%) participants in the study group had an MVC history.<br><br>40.3% (448/119) participants in the study group

For peer review only

							the control group was.		and 70.6% (36/51) in the control group listed poor visibility as the cause of their MVC involvement ($p < 0.05$).
Ogbonnaya CE et al., 2018	Cross-sectional	103 (9/94)	43.2	Nigeria	Monocular vision impairment	Physician diagnosed	Drivers with monocular impairment but with no MVC history	X^2 (Chi Square)	The relationship between monocular visual impairment and self-reported history of RTA was not statistically significant; X2 =0.045, (p= 0.85)
		103 (7/96)			Monocular blindness	Physician diagnosed	Drivers with monocular blindness but with no MVC history	X^2 (Chi Square)	The relationship between monocular blindness and self-reported history of RTA was not statistically significant; X2 =0.358 (p= 0.55)
Vofo BN et al., 2021	Cross-sectional	207 (51/156)	41.8	Cameroon	Self-reported vision impairment.	Self-reported	Drivers without self-reported	X^2 (Chi Square)	Drivers with self-reported VI were involved in significantly

For peer review only

							vision impairment		higher number of MVCs (72.5%) than those with self-reported good vision (55.8%) (p< 0.05)
									Drivers with self-reported VI had higher average number of MVCs over previous 10 years (1.75 +/- 1.64) than drivers with self-reported good vision (1.03 +/- 1.40 (p< 0.05).

*unadjusted results

For peer review only

Table 4a(xiii) All studies (n=6) evaluating cataract surgery and Motor Vehicle Crashes (MVC) with meta-analysis suitable for 3 studies on the associations with any MVC involvement

**Author and Year**	**Study Design**	**Total Participants (exposure/control)**	**Mean Age**	**Country**	**VI Definition**	**Comparator**	**Outcome Measure (OR, RR, HR?)**	**Effect Measure (with 95% CI)**
**Included in Meta-Analysis (any MVC involvement)**								
Meuleners L et al., 2012	Retrospective Cohort	27827 (N/A)	age, no.: 60-69 years = 6609, 70-79 years = 14506, 80+ years = 6712	Australia	Physician diagnosed	Crashes before surgery	RR (risk ratio)	0.87 (0.76, 0.99)
Meuleners L et al., 2019	Retrospective Cohort	2849 (N/A)	age, no.: 60-64 years = 347, 65-69 years = 482, 70-74 years = 720, 75-79 years = 719, 80-84 years = 454, 85 +	Australia	Physician diagnosed cataract	Crashes before surgery	RR (risk ratio)	0.39 (0.37, 0.41)

For peer review only

			years = 127					
Owsley C et al., 2002	Prospective Cohort	277 (174/103)	71.3	USA	Cataract in 1 or both eyes with best-corrected VA of 20/40 or worse	Crashes before surgery	RR (rate ratio)	0.47 (0.23, 0.94)
**Included in Narrative Summaries Only – High Income Countries**								
McCloskey L et al., 1994	Case Control	683 (235/448)	age, no.: 65- 69 years = 264, 70-74 years = 195, 75-79 years = 138, 80+ years = 88	USA	Self-reported physician diagnosed cataracts	Drivers who experienced no injuries in a crash.	RR (relative risk)	**Post-surgery with lens implant:**1 (0.5, 2.3)*
Meuleners L et al., 2012	Retrospective Cohort	Males: 1091 (513/611)	age, no.: 60-69 years = 447, 70-79 years = 823, 80 + years = 445	Australia	Physician diagnosed	No. of pre cataract surgery police reported crashes in all participants.	RR (risk ratio)	**Males:** 0.84 (0.72, 0.99)
		Females: 624 (308/330)						**Females**: 0.99 (0.75, 1.16)
Meuleners L et al., 2019	Retrospective Cohort	2849 (N/A)	age, no.: 60-64 years = 347, 65-69 years	Australia	Physician diagnosed cataract	Crashes before surgery	RR (risk ratio)	**After 2nd eye cataract surgery:** 0.77 (0.75, 0.78)

For peer review only

			= 482, 70-74 years = 720, 75-79 years = 719, 80-84 years = 454, 85 + years = 127					
Schlenker M et al., 2018	Prospective cohort	559546 (N/A)	76	Canada	Physician diagnosed	No. of pre cataract surgery crashes in all participants	OR	0.91 (0.84, 0.97)*

*unadjusted results

For peer review only

Table 4a(xiv) All studies (n=1) evaluating corrective lens wear to improve refractive error and Motor Vehicle Crashes (MVC)

Author and Year	Study Design	Total Participants (exposure/control)	Mean Age	Country	Vision impairment	VI Definition	Comparator	Outcome Measure (OR, RR, HR?)	Effect Measure (with 95% CI)
McCloskey L et al., 1994	Case Control	683 (235/448)	age, no.: 65- 69 years = 264, 70-74 years = 195, 75-79 years = 138, 80+ years = 94	USA	Refractive Error	Use of corrective lenses for any reason (far or near vision)	Drivers who experienced no crash-related injuries	RR (risk ratio)	0.6 (0.3, 1.1)*
								Prevalence (%)	% with condition, cases: 91% (214/235) % with condition, controls: 94.6% (424/448)

* unadjusted results

For peer review only

Appendix 4b Raw data tables and additional narrative summaries of papers on driving cessation

Table 4b(i) All studies (n=13) on glaucoma and driving cessation with meta-analysis suitable for 2 studies

Additional Narrative Summaries:
Persons with bilateral glaucoma (OR 2.6 (95% Cl 1.4-4.8); p= 0.002) were more likely to stop driving but those with unilateral glaucoma were not (OR 1.5 (95% Cl 0.7-2.9); p= 0.3) with one Japanese study reporting individuals with severe POAG in the better eye to have an approximately 11.5 times greater odds of driving cessation than persons without POAG.

**Author and Year**	**Study Design**	**Total Participants (exposure/control)**	**Mean Age**	**Country**	**VI Definition**	**Comparator**	**Outcome Measure (OR, RR, HR?)**	**Effect Measure (with 95% Cl)**
**Included in Meta-analysis**								
Edwards J et al., 2008	Cross-sectional	1656 (152/1504)	72.95	USA	Self-reported physician diagnosed	Participants without glaucoma	HR	1.47 (0.98, 2.19); p=0.06
Gilhotra JS et al., 2001	Cross-sectional	3654 (61/3593)	65.9	Australia	Self-reported and physician diagnosed	Participants without glaucoma	OR	2.2 (1.3, 3.9)
**Included in Narrative Summaries Only – High Income Countries**								
Adler G et al., 2004	Cross-sectional	199 (52/147)	71.3	USA	Open-or closed-angle glaucoma	Participants without glaucoma	X^2 (Chi Square)	Drivers with glaucoma were no more likely than controls to have made plans for driving cessation; p=0.49
Edwards J et al., 2008	Cross-sectional	1656 (152/1504)	72.95	USA	Self-reported physician diagnosed	Participants without glaucoma	Prevalence (%)	8.6% (125/1450) of current drivers had glaucoma compared to 13.9% (28/199) of non-drivers with glaucoma.
Gilhotra JS et al., 2001	Cross-sectional	3654 (61/3593)	65.9	Australia	Open-angled	Participants without glaucoma	Prevalence (%)	2% (37/2379) of current drivers had glaucoma compared to 5% (24/451) of non-drivers with glaucoma.

For peer review only - http://bmjopen.bmj.com/site/about/guidelines.xhtml

Goh Y et al., 2011	Case Series	77 (77/0)	71.8	UK	Physician diagnosed	Participants with glaucoma and other ocular pathologies	OR	At clinic presentation: 4.99 (1.2, 20.6)* Glaucoma patients with other ocular pathologies were more likely to fail the driving criteria and give up driving than patients with only glaucoma.
								At last clinic visit: 4.37 (1.6, 11.8) Glaucoma patients with other ocular pathologies were more likely to fail the driving criteria and give up driving than patients with only glaucoma.
Kaleem MA et al., 2021	Cross-sectional	191 (191/0)	77	USA	Physician diagnosed	Drivers with glaucoma but with either better VA or CS.	Prevalence (%) and X^2 (Chi Square)	78% of participants reported that they had stopped driving.
								Participants with worse VA were more likely to stop driving ($p < 0.05$)
								Participants with worse CS were more likely to stop driving ($p < 0.01$).
MacLeod K et al., 2014	Cross-sectional	1279 (67/1211)	age, no.: 55-64 years = 233, 65-	USA	Self-reported physician diagnosed	Ex-drivers without glaucoma	RR (risk ratio)	1.3
							Attributable Risk	1.6

For peer review only

			74 years = 499, 75+ years = 547				Prevalence (%)	7.4% (6/79) of non-driving participants had glaucoma compared to 5.7% (5/79) who did not have glaucoma.
Marottoli RA et al., 1993	Cross-sectional	1331 (28/1303)	age, no.: 65-74 years = 484, 75-84 years = 105, 85+ years = 6	USA	Self-reported physician diagnosed	Participants without glaucoma	Prevalence (%)	From the 28 participants who reported glaucoma at baseline (1983), 42.9% (12/28) stopped driving by 198 compared to 22.2% (125/564) of people who did not have glaucoma and who also stopped driving.
Naredo Turrado J et al., 2020	Prospective cohort	11670 (525/11144)	62.4	France	Self-reported physician diagnosed	Participants without glaucoma	HR	1.6, p>0.05
Ramulu P et al., 2009	Cross-sectional	1135 (138/997)	79.7	USA	Bilateral or unilateral	Participants without glaucoma	OR	Bilateral: 2.6 (1.4, 4.8)<br><br>Stopped driving for over 8 years (bilateral): 3 (1.4, 6.4)*<br><br>Stopped driving less than 2 years ago (bilateral): 3.6 (1.5, 5.8)
								Unilateral: 1.5 (0.7, 2.9)<br><br>Stopped driving less than 2 years ago (unilateral): 2.4 (1, 6)
							Prevalence (%)	40.6% (28/68) of all participants with

For Peer Review only

								bilateral glaucoma were not driving. 21.4% (15/70) of all with unilateral glaucoma were not driving. 15% (150/997) of all without glaucoma were not driving.
Takahashi A et al., 2018	Prospective cohort	359 (211/148)	54	Japan	Mild POAG at baseline	Participants without glaucoma	OR	No association found (data not shown)
					Moderate POAG in the better eye at the 3 year follow-up			37.7 (3.7, 383.8)
					Severe POAG in the better eye at baseline			11.52 (2.87, 46.35)
					Severe POAG in the better eye at 3-year follow-up			52.8 (3.5, 797)
							Prevalence (%)	5.3% (8/152) of those with mild glaucoma were no longer driving. 21% (7/33) of those with moderate/severe glaucoma were no longer driving. A total of 8.1% (15/185) of all participants with glaucoma were not driving compared to 1/3% (1/80) of drivers without glaucoma who

For peer review only

								were also no longer driving.
Tam A et al., 2018	Cross-sectional	99 (99/0)	71.5	Canada	Glaucoma severity was defined by the visual field mean deviation (MD) in the better eye and classified into 2 groups: mild (MD >−6 dB) and moderate/severe (MD ≤−6 dB), corrected visual acuity in the better eye ≥20/50	Mild/moderate glaucoma patients	Prevalence (%)	33% (15/46) of mild/moderate glaucoma reported driving cessation compared to 8% (4/53) of mild glaucoma patients; p= 0.002
vanLandingham et al., 2013	Cross-sectional	139 (81/58)	70.1	USA	Physician diagnosed	Glaucoma suspect controls	OR	4 (1.1, 4.7); p=0.03
							Prevalence (%)	22.5% (18/81) of participants with glaucoma were no longer driving.
**Included in Narrative Summaries Only – Low Middle Income Countries**								
Deshmukh AV et al., 2019	Case Control	150 (100/50)	64.5	India	Anderson criterion	Drivers without glaucoma	Prevalence (%)	16% (16/100) of those with glaucoma has stopped driving.

*unadjusted results

For peer review only

Table 4b(ii) All studies (n=4) on cataract and driving cessation, all suitable to be summarised narratively only

Additional Narrative Summaries:
One study with sex disaggregated analysis found male drivers to be 7.01 times more likely to stop driving compared to female drivers who only had a 3.67 odds of driving cessation. Only one study examined the impact of a diagnosis of wet AMD but did not find any significant associations.

Author and Year	Study Design	Total Participants (exposure/control)	Mean Age	Country	VI Definition	Comparator	Outcome Measure (OR, RR, HR?)	Effect Measure (with 95% CI)
**Included in Narrative Summaries Only – High Income Countries**								
MacLeod K et al., 2014	Cross-sectional	1279 (278/1001)	age, no.: 55-64 years = 233, 65-74 years = 499, 75+ years = 547	USA	Self-reported physician diagnosed	Ex-drivers without cataract.	RR (risk ratio)	1.5
							Attributable risk	10.5, p<0.1
							Prevalence (%)	8% (6/79) of participants with cataracts no longer drove compared to 5.2% (4/79) with no cataracts.
Marottoli RA et al., 1993	Cross-sectional	1331 (105/1226)	age, no.: 65-74 years = 484, 75-84 years = 105, 85+ years = 6	USA	Self-reported physician diagnosed	Current Drivers	OR	2.29 (1.28, 4.1)
							Prevalence (%)	45.7% (48/105) of participant with cataracts were no longer driving compared to 18.4% (90/488) of those who were no longer driving and did not have cataracts.
Naredo Turrado J et al., 2020	Prospective cohort	11670 (291/11379)	62.4	France	Self-reported physician diagnosed	Current drivers	HR	1.79, p>0.05
Sengupta S et al., 2014	Cross-sectional	122 (N/A)	72.4	USA	Physician diagnosed	Participants without cataract/PCSO in better eye.	PR (Prevalence Ratio)	Presence of cataract/PCO in the better seeing eye did not show any significant association

For peer review only

								with driving cessation; p>0.5

*unadjusted results

Table 4b(iii) All studies (n=5) on AMD and driving cessation with meta-analysis suitable for 3 studies

**Author and Year**	**Study Design**	**Total Participants (exposure/control)**	**Mean Age**	**Country**	**VI Definition**	**Comparator**	**Outcome Measure (OR, RR, HR?)**	**Effect Measure (with 95% CI)**
**Included in Meta-analysis**								
Campbell MK et al., 1993	Case Control	1656 (276/1380)	N/A	USA	Self-reported physician diagnosed	Current drivers	OR	4.25 (2.6, 7); p<0.001
Edwards J et al., 2008	Cross-sectional	1656 (89/1567)	72.95	USA	Self-reported physician diagnosed	Current drivers	HR	1.46 (0.91, 2.36); p=0.12
Stewart RB et al., 1993	Cross-sectional	1470 (N/A)	78.1	USA	Self-reported physician diagnosed	Current drivers	OR	3.32 (1.91, 5.77); p=0.0001
**Included in Narrative Summaries Only – High Income Countries**								
Campbell MK et al., 1993	Case Control	1656 (276/1380)	N/A	USA	Self-reported physician diagnosed	Current drivers	OR	**Male**: 7.01 (3.1, 15.9); p<0.001)*
								**Female**: 3.67 (2.0, 6.8), p<0.001*
							Prevalence (%)	5.06% (70/1379) of participants still driving had AMD compared to 17.88% (50/277) of non-drivers with AMD.
Edwards J et al., 2008	Cross-sectional	1656 (89/1567)	72.95	USA	Self-reported physician diagnosed	Current drivers	Prevalence (%)	4.9% (71/1457) of participant still driving had AMD compared to 9.5% (19/198) of non-driving participant with AMD.

For peer review only

MacLeod K et al., 2014	Cross-sectional	1279 (48/1231)	age, no.: 55-64 years = 233, 65-74 years = 499, 75+ years = 547	USA	Self-reported physician diagnosed	Ex-drivers without AMD.	RR (risk ratio)	2.3
							Attributable risk	4.5, p<0.01
							Prevalence (%)	12.7% (10/79) of ex-drivers had AMD compared to 5.6% (4/79) of ex-drivers without AMD.
Sengupta S et al., 2014	Cross-sectional	122 (64/58)	72.4	USA	Physician reported wet AMD	Participants without AMD	OR	**Any eye:** 1.9 (0.5, 7.3)
								**Worse eye:** 0.6 (0.1, 3.3)
								**Better eye:** 2.7 (0.6, 11.5)
							Prevalence (%)	74.6% (48/64) of participant with AMD were still driving compared.
								More participants in the AMD group (25.4%) had stopped driving compared to those without AMD (6.9%); p= 0.006
Stewart RB et al., 1993	Cross-sectional	1470 (N/A)	78.1	USA	Self-reported physician diagnosed	Current drivers	Prevalence (%)	59.8% (35/58) of participant with AMD were still driving.

*unadjusted results

For peer review only

Table 4b(iv) All studies (n=18) on visual acuity (VA) impairment and driving cessation, all suitable to only be summarised narratively due to their different VA cut-off points and comparators

Author and Year	Study Design	Total Participants (exposure/control)	Mean Age	Country	VI Definition	Comparator	Outcome Measure (OR, RR, HR?)	Effect Measure (with 95% CI)
**Included in Narrative Summaries Only – High Income Countries**								
Anstey K et al., 2006	Prospective Cohort	1466 (446/1020)	age no.: 70-74 years = 378, 75-79 years = 353, 80-84 years = 339, 85+ years = 396	Australia	Corrected distance VA at 3 metres in best eye at 6/12 or worse	Participants with VA better than 6/12 (i.e. better than 20/40)	OR	Visit 2: 1.91 (0.51, 7.13)<br>Visit 3: 1.84 (0.68, 4.99)<br>Visit 4: 1.15 (0.55, 2.41)
DeCarlo D et al., 2003	Cross-sectional	126 (N/A)	79	USA	Better eye	Current drivers	Mean (SD)	VA in the better eye was worse in non-drivers (1.03 +/- 0.39) than drivers (0.74 +/- 0.34).
					Worse eye			VA in the worse eye was worse in non-drivers (1.58 +/- 0.43) than drivers (1.18 +/- 0.42).

For peer review only

Edwards J et al., 2008	Cross-sectional	1656 (N/A)	72.95	USA	ETDRS chart with scores assigned from 0 to 90 (e.g. score of 0 = Snellen score of 20/125, score of 90 = Snellen score of 20/16)	Current drivers	HR (multivariate model)	0.91 (0.791, 1.046); p=0.184
							HR (cox regression)	0.69 (0.61, 0.78); p<0.001
Freeman E et al., 2005	Prospective cohort	1824 (263/1561)	73.4	USA	≥ 0.1 and <0.3 logMAR at baseline	<0.1 logMAR as baseline	HR	1.27 (0.96, 1.69)
		1824 (63/1498)			≥0.3 logMAR VA at baseline	<0.1 logMAR as baseline		1.23 (0.69, 2.18)
		1824 (329/1495)			1-2 lines VA loss	<1 line loss in VA		1.25 (0.96, 1.65)
		1824 (134/1690)			>2 lines VA loss	<1 line loss in VA		1.26 (0.87, 1.84)
Garre-Olmo J et al., 2009	Cross-sectional	875 (N/A)	81.7	Spain	Self-reported	Drivers without impaired VA	OR	0.379 (0.201, 0.714); p=0.003*
Gilhotra JS et al., 2001	Cross-sectional	3654 (80/3574)	65.9	Australia	BCVA worse than 6/12 in the better eye	Current drivers	OR	4 (2.5, 3.9)
		3654 (283/3371)			Presenting VA worse than 6/12 in the better eye			2.5 (1.9, 3.4)
							Prevalence (%)	11% (49/452) of participants have stopped driving have VA >20/40 compared to the 1% (21/2379) who are still driving with VA >20/40.
Huisingh C et al., 2016	Prospective Cohort	1995 (161/1834)	77.2	USA	logMar <0.3	Drivers without VA impairment	HR	0.83 (0.49, 1.42)
							Mean (SD)	VA of those who stopped driving (0.097 [0.15]) compared to those still driving (0.051 [0.13]).

							Prevalence (%)	90.9% (149/164) of those not driving had a VA of ≤20/40 compared to 9.2% (15/164) who stopped driving with a VA of >20/40.
Janz N et al., 2009	Prospective Cohort	607 (N/A)	age, no.: 25-49 years = 131, 50-64 years = 240, 65-74 years = 177	USA	Better eye at 6 months	Driving vs. non-drivers	2-sample t-test	Mean (SD) of VA in drivers (87.7 [4.9]) vs. non-drivers (85.1 [5.4]); p<0.001
							Linear regression	Mean (SD) of VA in drivers (87.7 [4.9]) vs. non-drivers (85.1 [5.4]); p=0.012
					Better eye at 54 months		2-sample t-test	Mean (SD) of VA in drivers (86.9 [5.7]) vs. Non-drivers (83.2 [6.9]); p= 0.025
							Linear regression	Mean (SD) of VA in drivers (86.9 [5.7]) vs. Non-drivers (83.2 [6.9]); p=0.458
					Mean (SD) difference in VA in better eye from 6 months to 54 months	Remained drivers vs. became non-drivers	Linear regression	Changes in Mean (SD) in VA of drivers (-0.4[0.6]) vs. became non-drivers (3.9[0.7]); p=0.001
					Worse eye at 6 months	Driving vs. non-drivers	2-sample t-test	Mean (SD) of VA in drivers (83.2 [7.5]) vs. non-drivers (79.7 [11.0]); p= 0.007
							Linear regression	Mean (SD) of VA in drivers (83.2 [7.5]) vs. non-drivers (79.7 [11.0]);

For peer review only

								p= 0.095
					Worse eye at 54 months		2-sample t-test	Mean (SD) of VA in drivers (81.5 [10.6]) vs. non-drivers (75.3 [14.4]); p=0.001
							Linear regression	Mean (SD) of VA in driver (81.5 [10.6]) vs. non-driver: 75.3 (14.4); p=0.003
					Mean (SD) difference in VA in worse eye from 6 months to 54 months	Remained drivers vs. became non-drivers	Linear regression	Mean (SD) of VA in drivers (1.4[1.3]) vs. became non-drivers: -5.5(2.1); p=0.054
Keay L et al., 2009	Prospective Study	1425 (N/A)	75	USA	LogMAR scale	Whole population	Mean (SD)	mean(SD) of VA statistically significant different between those who stopped driving 0.08 (0.014) and those who continued driving -0.01(0.11); p=0.0006
Keay et al., 2016	Cross-sectional	442 (N/A)	73	Australia	High contrast vision	Current drivers with cataracts NOTE: all participants had cataracts	OR	1.21 (1.07, 1.37)
					Binocular		X^2 (Chi Square)	p<0.001
					Better eye			p<0.001
					Worse Eye			p<0.001
Levecq L et al., 2013	Cross-sectional	1000 (N/A)	71.3	Belgium	Physician diagnosed binocular VA worse than 20/40	Current drivers	X^2 (Chi Square)	Right eye: Mean VA of current drivers (0.31) was significantly better than those who gave up driving due to vision (0.25); p=0.016
								Left eye: Mean VA in current drivers (0.31) was significantly

								better than those who gave up driving due to vision (0.24); p=0.004
								Both eyes:<br>Mean VA in current drivers (0.36)<br>Was significantly better than those who gave up driving due to vision (0.31); p=0.031
Ramulu P et al., 2009	Cross-sectional	1135 (N/A)	79.7	USA	Binocular acuity 0.1 logMAR or worse in better eye.	Drivers without 0.1 logMAR binocular.	OR	1.5, p<0.001
Ross L et al., 2009	Cross-sectional	5206 (1062/4144)	76.3	Australia	Physician diagnosed with participants categorised into having a VA LogMAR 0.3 or better, or worse than LogMar 0.3.	Participants with normal vision (logMAR of or better than 0.3).	OR	2.08 (2.56, 1.69)*
Rubin G et al., 2007	Prospective Cohort	2520 (N/A)	age, no.: 65-69 years = 780, 70-74 years = 829, 77-79 years = 553, 80-85 years = 350	USA	15 letter loss (logMAR 0.3)	Current Drivers	Prevalence (%)	Of those no longer driving: 84% (604/719) had VA ≤ 20/40 whilst 16% (115/719) had VA > 20/40.

For peer review only

Segal-Gidan F et al., 2010	Cross-sectional	421 (44/377)	72	USA	Mild vision impairment was defined at the BCVA in the better eye (20/40-20/63)	Current drivers	OR	5.53 (1.45, 20.98)
		421 (23/377)			Moderate/severe vision impairment was defined as BCVA in the better eye (20/80 or worse)			13.23 (1.45, 120.3)
Sengupta S et al., 2014	Cross-sectional	122 (N/A)	72.4	USA	Worse VA in the better eye (1 line loss of vision)	1 line worse in better eye acuity (logMAR) in all participants	OR	Low VA in either eye: 1.4 (1.1, 1.9); p<0.001 Low VA in better eye: 1.5 (1.2, 1.9); p<0.001
							Mean (SD)	Participants who had stopped driving (logMAR VA 0.77) had significantly worse vision in the better seeing eye than those still driving (LogMAR VA 0.08); p=0.001
Tam A et al., 2018	Cross-sectional	99 (N/A)	71.5	Canada	Physician diagnosed	N/A – looked at VA in whole population (all glaucoma patients)	X^2 (Chi Square)	Best corrected VA not associated with cessation; p=0.18
								Declines in central vision was significantly associated with driving cessation; p= 0.001
								Declines in near vision was significantly associated with driving cessation; p= 0.001
								Declines in peripheral vision was significantly associated

For peer review only

								with driving cessation; p= 0.001
vanLandingham S et al., 2013	Cross-sectional	139 (N/A)	70.1	USA	1 line worse in the better eye	Glaucoma suspect controls	OR	1.3 (1, 1.8); p<0.05
						Moderate VA loss in glaucoma cases		Severe VA loss: 1.5 (1.2, 1.8); p< 0.05

*unadjusted results

For peer review only

Table 4b(v) All studies (n=8) on contrast sensitivity (CS) impairment and driving cessation with 3 studies suitable for meta-analysis

Additional Narrative Summaries:
CS was measured either as a continuous measure, or categorised as "poor" according to normative cut-points, with one study using both measures.

**Author and Year**	**Study Design**	**Total Participants (exposure/control)**	**Mean Age**	**Country**	**VI Definition**	**Comparator**	**Outcome Measure (OR, RR, HR?)**	**Effect Measure (with 95% Cl)**
**Included in Meta-analysis**								
Huisingh C et al., 2016	Prospective cohort	1995 (130/1865)	77.2	USA	<1.5 score on Pelli-Robson chart	Drivers with no bilateral CS impairment	HR	1.73 (1.1, 2.72)
							Mean (SD)	The mean log CS of current drivers was 1.68 (0.13) compared to 1.61 (0.16) in non-drivers.
							Prevalence (%)	5.8% (106/1831) of current drivers had a log CS <1.5, compared to 14.6% (24/164) who stopped driving.
Keay L et al., 2009	Prospective cohort	1425 (N/A)	75	USA	Per letter lost	Drivers with no bilateral CS impairment	OR	1.15 (1.03, 1.28)*
					Better eye CS		Mean (SD)	CS in better eye of those who stopped driving 32.4(4.1) significantly different between those who continued driving 35.3(2.2); p<0.001
		122 (N/A)	72.4	USA			OR	1.36 (1.1, 1.7); p<0.05

Sengupta S et al., 2014	Cross-sectional				Binocular CS 1 letter worse	Drivers with no bilateral CS impairment	X^2 (Chi Square)	Those who stopped driving had significantly worse CS (log CS 1.8) compared to those still driving (log CS 1.2); p=0.03
**Included in Narrative Summaries Only – High Income Countries**								
Freeman E et al., 2005	Prospective cohort	1824 (725/1099)	73.4	USA	>=32 and <36 letters CS at baseline	Baseline CS equal to or more than 36 letters.	HR	1.26 (0.97, 1.63)
		1824 (158/1666)			<32 letters at baseline			1.46 (0.98, 2.17)
		1824 (79/1725)			5 letter CS loss in 2 years	Less than 5 letter CS loss		1.33 (0.8, 2.22)
		1824 (86/1738)			>= 6 letter CS loss in 2 years			1.71 (1.01, 2.9)
Keay L et al., 2016	Cross-sectional	442 (N/A)	73	Australia	0.12 log units drop in CS.	Cataract patients who are still driving	OR	1.29 (1.11, 1.49)
							Prevalence (%)	17% (45/263) of current drivers and 35% (37/110) of former drivers had a CS <1 log decrease by at follow-up; p< 0.001
							Mean (SD)	The worse eye CS in current drivers was 1.27 (+/- 0.36) compared to 1.11 (+/- 0.41) in former drivers; p< 0.001
Ramulu P et al., 2009	Cross-sectional	1135 (N/A)	79.7	USA	5 letters worse in better eye	Current drivers without 5 letters worse in better eye CS.	OR	3, p<0.001
Rubin G et al., 2007	Prospective Cohort	2520 (N/A)	Age, no,: 65-69 years =	USA	Log CS ≥ 1.65	Current drivers	Prevalence (%)	49.1% (884/1801) participants had stopped driving.

For peer review only

			780, 70-74 years = 829, 77-79 years = 553, 80-85 years = 350		Log CS 1.35-1.65			54% (973/1801) had stopped driving.
					Log CS <1.35			96.9% (1745/1801) had stopped driving.
vanLandingham S et al., 2013	Cross-sectional	139 (N/A)	70.1	USA	Binocular CS 1 letter worse	Glaucoma suspect controls	OR	1.3 (1.2, 1.4); p<0.05

*unadjusted results

Table 4b(vi) All studies (n=8) on visual field (VF) impairment and driving cessation, all suitable to only be summarised narratively due to their different VF cut-off points and comparators

**Author and Year**	**Study Design**	**Total Participants (exposure/control)**	**Mean Age**	**Country**	**VI Definition**	**Comparator**	**Outcome Measure (OR, RR, HR?)**	**Effect Measure (with 95% CI)**
**Included in Narrative Summaries Only – High Income Countries**								
Freeman E et al., 2005	Prospective Cohort	1824 (659/1165)	73.4	USA	>1 and <= 8 points of central visual field at baseline	Equal to or greater than 1 points missed at baseline central VF	HR	1.34 (1.02, 1.76)
		1824 (174/1650)			>9 points of central visual field at baseline	Equal to or greater than 1 points missed at baseline central VF		1.81 (1.23, 2.66)
		1824 (65/1759)			5-7 points of central visual field loss in 2 years	<5 central VF loss		1.01 (0.6, 1.72)

For peer review only

		1824 (92/1732)			>=8 points of central visual field loss in 2 years	<5 central VF loss		0.83 (0.53, 1.29)
		1824 (632/1192)			>9 and <=18 points of peripheral visual field at baseline	Less than or equal to 9 points missed at baseline peripheral VF		1.51 (1.14, 1.98)
		1824 (180/1644)			>18 points of peripheral visual field at baseline	Less than or equal to 9 points missed at baseline peripheral VF		1.73 (1.14, 1.98)
		1824 (106/1718)			6-7 points of peripheral visual field loss in 2 years	<6 points loss of peripheral VF		1.04 (0.65, 1.65)
		1824 (88/1736)			>= 8 points of peripheral visual field loss in 2 years	<6 points loss of peripheral VF		1.91 (1.23, 2.96)
Huisingh C et al., 2016	Prospective cohort	1995 (493/1502)	77.2	USA	sensitivity <=22.5 dB	Participants without VF impairment	HR	1.78 (1.29, 2.46)
Janz N et al., 2009	Prospective cohort	607 (N/A)	age, no.: 25-49 years = 131, 50-64 years = 240, 65-74 years = 187	USA	Better eye at 6 months	Drivers vs. non-drivers	2-sample t-test	Mean (SD) MD of drivers (-2.1 [2.7]), vs. non-drivers (-2.9 [3.0]); p=0.014
							Liner regression	Mean (SD) MD of drivers (-2.1 [2.7]) vs. non-drivers (-2.9 [3.0]); p=0.966
					Better eye at 54 months		2-sample t-test	Mean (SD) MD of drivers (-1.9[3.1]) vs. non-drivers (-3.5 [3.7]); p<0.001

For peer review only

							Linear regression	Mean (SD) MD of drivers (-1.9[3.1]) vs. non-driver: -(3.5[3.7]);<br>p= 0.007
					Mean (SD) difference in VA in better eye from 6 months to 54 months	Remain drivers vs. became non-drivers	Linear regression	Difference in mean (SD) MD of drivers (0.2 [2.1]) vs. became non-drivers (-0.7 [2.7]);<br>p=0.008
					Worse eye at 6 months	Drivers vs. non-drivers	2-sample t-test	Mean (SD) MD of drivers (-5.7 [4.9]) vs. non-drivers (-5.9 [4.0]);<br>p=0.014
							Liner regression	Mean (SD) MD of drivers (-5.7 [4.9]) vs. non-drivers (-5.9 [4.0]);<br>p=0.429
					Worse eye at 54 months		2-sample t-test	Mean (SD) MD of drivers (-5.4 [5.2]) vs. non-drivers (-7.0 [4.9]);<br>p=0.012
							Linear regression	Mean (SD) MD of drivers (-5.4 [5.2]) vs. non-drivers (-7.0 [4.9]);<br>p=0.080
					Mean (SD) difference in VA in worse eye from 6 months to 54 months	Remain drivers vs. became non-drivers	Linear regression	Difference in mean (SD) MD of drivers (0.3 [0.4]), vs. became non-drivers (-1.3 [0.7]);<br>p= 0.013
Keay L et al., 2009	Prospective Cohort	1425 (N/A)	75	USA	Bilateral VF points missing	Whole population	Mean (SD)	Mean(SD) of bilateral VF points<br>missing was statistically

For peer review only

								significant different between those who stopped driving 9.8(17.1) and those who continued driving 1.98(5.1); p=0.001
Keay L et al., 2016	Cross-sectional	442 (N/A)	73	Australia	Points missed on bilateral VF.	Current drivers with cataracts NOTE: all participants in this study had cataracts.	X^2 (Chi Square)	Median (IQR) of current drivers: 3 (0-10) vs. Median (IQR) of former drivers: 8 (1-19); p= 0.02
Ramulu P et al., 2009	Cross-sectional	1135 (N/A)	79.7	USA	Bilateral VF damage in glaucoma participants	Participants without glaucoma	OR	2 (1.6, 2.5)
							Prevalence (%)	21% (14/68) of participants with bilateral VF loss in the lowest tertile (less than 3 dB of VF loss in better-eye) had stopped driving.<br><br>36% (24/68) of participants with VF loss in the middle tertile (better-eye VF mean deviation between −3 and −9 dB) had stopped driving.<br><br>52% (35/68) of participants with VF loss in the highest tertile (better eye VF mean deviation <-9 dB) had stopped driving.
Segal-Gidan F et al., 2010	Cross-sectional	421 (30/391)	72	USA	Unilateral	Current drivers	OR	1.91 (0.63, 5.76)
		421 (108/318)			Bilateral, mild			2.05 (0.74, 5.66)

		421 (93/328)			Bilateral, moderate/severe			2.84 (0.92, 8.78)
vanLandingham S et al., 2013	Cross-sectional	139 (N/A)	70.1	USA	5 dB worse in the better eye	Glaucoma suspect controls	OR	1.7 (1.1, 2.5); p= 0.008
**NOTE:** There are a range of different study designs as well as cut-off points and areas of VF investigated in the identified studies. Due to methodological differences between each study, meta-analysis was limited and narrative reviews have been used instead to synthesise data.								

*unadjusted results

Table 4b(vii) All studies (n=3) on glare sensitivity (GS) impairment and driving cessation, all suitable to only be summarised narratively due to their different GS cut-off points and comparators

Author and Year	Study Design	Total Participants (exposure/control)	Mean Age	Country	VI Definition	Comparator	Outcome Measure (OR, RR, HR?)	Effect Measure (with 95% CI)
**Included in Narrative Summaries Only – High Income Countries**								
Freeman E et al., 2005	Prospective Cohort	1824 (702/1122)	73.4	USA	3-4 points of glare sensitivity at baseline	≤2 points difference with baseline glare GS	HR	0.78 (0.61, 0.99)
		1824 (206/1618)			≥5 points of glare sensitivity at baseline			0.9 (0.63, 1.28)
		1824 (71/1753)			4 points loss of glares sensitivity in 2 years	<4 points GS loss		1.18 (0.7, 1.99)
		1824 (52/1772)			≥5 points loss of glare sensitivity in 2 years	<4 points GS loss		1.3 (0.72, 2.37)
Gilhotra JS et al., 2001	Cross-sectional	3654 (969/2685)	65.9	Australia	Physician diagnosed	Participants still driving	OR (logistic regression)	1.5 (1.2, 1.8)
Tam A et al., 2018	Cross-sectional	99 (15/84)	71.5	Canada	Physician diagnosed	Still driving participants with cataracts	Prevalence ratio (PR)	4.79; p<0.013

For peer review only

*unadjusted results

Table 4b(viii) All studies (n=11) on other types of vision impairment and driving cessation, all suitable to be summarised narratively only

Author and Year	Study Design	Total Participants (exposure/control)	Mean Age	Country	Type of VI	VI Definition	Comparator	Outcome Measure (OR, RR, HR?)	Effect Measure (with 95% CI)
**Included in Narrative Summaries Only – High Income Countries**									
Campbell MK et al., 1993	Case control	1656 (28/1628)	N/A	USA	Retinal detachment	Self-reported physician diagnosed	Current drivers	Prevalence (%)	Still driving = 14.25%. Not driving = 40.95% Those not driving have a higher percentage of detached retina than those still driving (p<0.05)
					Retinal haemorrhage	Self-reported physician diagnosed		OR	Both genders = 3.86 (1.4, 10.4)*
									Females: 4.70 (1.2, 17.8); p<0.5
					Vision impairment	Self-reported		Prevalence (%)	Still driving = 13.65%. Not driving = 25.34% Those not driving have a higher percentage of other visual loss than those still driving (p<0.01)
DeCarlo D et al., 2003	Cross-sectional	126 (126/0)	79	USA	Maculopathy	exudative or non-exudative	Current drivers	Prevalence (%)	The type of AMD (exudative vs nonexudative) was not significant between the non-drivers and drivers (p=0.474). Nonexudative non-drivers: 50% (48/96), nonexudative drivers : 47% (14/30), exudative non-

For peer review only

									drivers: 50% (48/96), exudative drivers 53% (16/30).
Hajek A et al., 2019	Cross-sectional	549 (192/357)	90.3	Germany	Vision impairment	Severe impairment	Current drivers	OR	0.06 (0.01, 0.59)*
						Mild impairment			0.56 (0.24, 1.35)*
Gallo JJ et al., 1999	Case Control	1920 (N/A)	N/A	USA	Vision impairment	Self-reported	Current drivers	OR	1.86 (0.7, 4.9)
Keay et al., 2016	Cross-sectional	442 (148/294)	73	Australia	URE	Measured with autorefraction and lensometry	Cataract patients who are still driving	X^2 (Chi Square)	No significant differences between current drivers with URE (40% [99/263]) and former drivers with URE (51% [49/110]); p= 0.07
Levecq L et al., 2013	Cross-sectional	1000 (346/654)	71.3	Belgium	Vision impairment	Physician diagnosed	N/A	Prevalence (%)	Among the 190 non-drivers, 47 (24.7%) stopped driving because of their impaired vision.
Marottoli RA et al., 1993	Cross-sectional	1331 (17/1314)	age, no.: 65-74 years = 484, 75-84 years = 105, 85+ years = 6	USA	Vision impairment	Self-reported	Current drivers	Prevalence (%)	Out of the 17 drivers who reported poor vision at baseline (1983), 58.8% (9/17) of drivers who stopped driving by 1989.
Moon SH et a., 2020	Cross-sectional	2970 (1023/1947)	71	South Korea	Vision impairment	Self-reported	Current drivers	OR	0.97 (0.83, 1.14)*
Robinson JL et a., 2021	Cross-sectional	335 (N/A)	67.4	USA	Vision impairment	Self-reported	Current drivers	X^2 (Chi Square)	Participants were less likely to be driving if they had noted vision-related concerns (p<0.001).

For peer review only

Tam A et al., 2018	Cross-sectional	99 (19/80)	71.5	Canada	Dark adaptation in glaucoma patients	Self-reported	Among patients with glaucoma	X^2 (Chi Square)	Dark adaptation significantly associated with driving cessation (p<0.001)
								PR (Prevalence Ratio)	1.47; p= 0.39 Individuals with self-perceived dark adaptation difficulties were not more likely to quit driving.
Zebardast N et al., 2015	Cross-sectional	2469 (132/2337)	73.5	USA	URE	Binocular presenting visual acuity of 20/30 or worse, improving to better than 20/30 with subjective refraction	Participants with normal vision.	OR	2.1 (1.3, 3.6)
					Non-refractive visual impairment	Post-refraction binocular BCVA of 20/30 or worse			3.7 (2.4, 5.7)

*unadjusted results

Table 4b(ix) All studies (n=2; reporting on 4 RCTs in total) evaluating anti-VEGF therapy and driving cessation, suitable for narrative summaries only

**Author and Year**	**Study Design**	**Intervention (n)**	**Control (n)**	**Mean Age**	**Country**	**Vision Impairment**	**VI Definition**	**Comparator(s)**	**Outcome Measure**	**Effect measure (with 95% where appropriate)**
Bressler N et al., 2013	RCT	478	238	77.7	USA	AMD	MARINA trial: minimally	Sham injections or 0.3 mg of Ranibizumab or 0.5 mg of	Prevalence (%) + 95% CI	Among patients who had reported driving at

For peer review only

							classic or occult AMD	Ranibizuman for 24 months		baseline, 74% (146/197) sham patients and 87.8% (156/178) 0.5mg patients reported still driving at 12 months. Among patients who had reported driving at baseline, 67.2% (131/195) (95% CI 59.2-75.2) of sham patients and 78.4% (148/189) (95% CI 71.8-85.0) of 0.5mg ranibizumab patients reported still driving 24 months later.

For peer review only

Bressler N et al., 2013	RCT	280	143	77.7	USA	AMD	ANCHOR: classic neovascular AMD	Verteporfin photodynamic therapy (PDT) or 0.3 mg ranibizumab injections or 0.5 mg ranibizumab injections for 24 months	Prevalence (%) + 95% CI	Among patients who reported driving at baseline, 80.5% (77/96) PDT patients and 94.2% (86/91) 0.5 mg patients reported still driving at 12 months. Among patients who reported driving at baseline, 71.6% (67/94) (95% CI 60.8-82.4) of PDT patients and 91.4% (81/89) (95% CI 85.3-97.5) of 0.5 mg ranibizumab patients reported still driving 24 months later.

For peer review only

Bressler N et al., 2016	RCT	502	257	62.3	USA	DME	RIDE/RISE: any DME	Sham injections or 0.3 mg ranibizumab or 0.5 mg ranibizumab	Prevalence (%) + 95% CI	For 0.3 mg ranibizumab compared to those treated with sham only, there was a 7% (-5.0 to 19) difference in the number of participants now driving (who were not driving at baseline) at 12 months. For 0.5 mg ranibizumab compared to those treated with sham only, there was a 14.4% (1.1, 27.7) difference in the number of participants now driving at 12 months. For 0.3 mg ranibizumab

For peer review only

										compared to those treated with sham only, there was a 12.5% (-0.9, 25.9) difference in the number of participants now driving at 24 months. For 0.5 mg ranibizumab compared to those treated with sham only, there was a 14.3% (0.7, 27.9) difference in the number of participants now driving at 24 months.

For peer review only

Bressler N et al., 2016	RCT	234	111	62.3	USA	DME	RESTORE: DME in a least 1 eye eligible for laser treatment and a VA letter score between 78 and 39	PDT laser only or 0.5 mg + laser or 0.5 mg only	Prevalence (%) with 95% CI	After 12 months, 12.2% (6/49) of those who were not driving at baseline and were treated with 0.5 mg ranibizumab + laser have started driving. Compared to those treated with laser only, there was a 4.2% (-7.7, 16.1) difference in the number of participants now driving at 12 months. After 12 months, 8.9% (4/45) of those who were not driving at baseline and were treated

For peer review only

										with 0.5 mg ranibizumab only have started driving. Compared to those treated with laser only, there was a 0.9% (-10.3, 12.1) difference in the number of participants now driving at 12 months.

For peer review only

Table 4b(x) All studies (n=2) evaluating cataract surgery and driving cessation, suitable for narrative summaries only

Author and Year	Study Design	Total Participants (exposure/control)	Mean Age	Country	VI Definition	Comparator	Outcome Measure (OR, RR, HR?)	Effect Measure (with 95% CI)
Monestam E et al., 2005	Prospective Cohort	810 (N/A)	74.7	Sweden	Physician diagnosed cataracts	All cataract surgery patients, comparing pre and post cataract surgery outcomes.	Prevalence (%)	Before cataract surgery, 55% (224/407) were drivers while after surgery 70% (285/407) were drivers. 5 years after surgery 63% (189/300) of patients with a driving licence were still active drivers. 37% (67/183) of patients who did not drive before surgery started to drive after. 46% (31/67) of patients who did not fulfil the visual requirements for presenting VA and the 35% (24/67) who did not fulfil the requirements for BCVA for a

For peer review only

								legal licence could now legally drive. 82% (40/50) of patents who began to drive after the surgery were still driving 5 years later.
Monestam E et al., 1997	Prospective cohort	211 (N/A)	41	Sweden	Physician diagnosed cataracts	Driving status from all participants pre- and post-surgery.	Ratio (%)	The number of patients driving after surgery increased to 65% (137/211) (from 56%), but this was not significant.

Table 4b(xi) All studies (n=1) evaluating anti-glaucoma therapy and driving cessation

**Author and Year**	**Study Design**	**Total Participants (exposure/control)**	**Mean Age**	**Country**	**Vision impairment**	**VI Definition**	**Comparator**	**Outcome Measure (OR, RR, HR?)**	**Effect Measure (with 95% CI)**
Stafford WR, 1981	Cross-sectional	240 (N/A)	age, no.: 35-49 years = 11, 50-65 years = 77, >65 years = 139	USA	Glaucoma	Chronic open-angle glaucoma or ocular hypertension that has been adequately controlled for at least	Post- anti-glaucoma therapy outcomes in all participants.	Prevalence (%)	From the 229 patients who stated that the anti-glaucoma therapy side effects affected their

For peer review only

						the previous 6 months			normal activity, 12% (28/229) said that they had to give up some normal activity. Out of the 28 patients, 16 mentioned giving up driving, particularly at night.

For peer review only

Appendix 5a Associations between MVC involvement and vision impairment and vision-related intervention

Vision impairment	Outcome	Association
**Glaucoma**	**Any MVC**	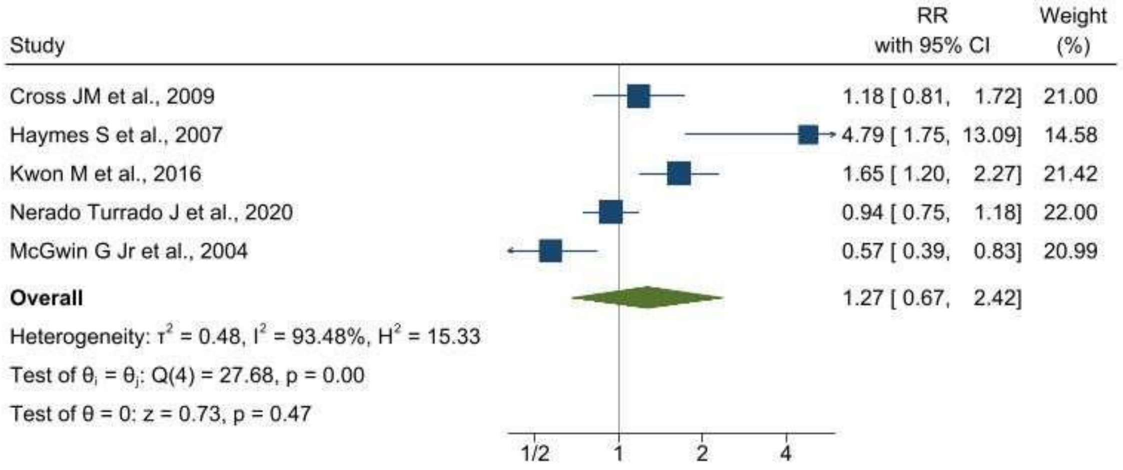
	**At-fault MVC**	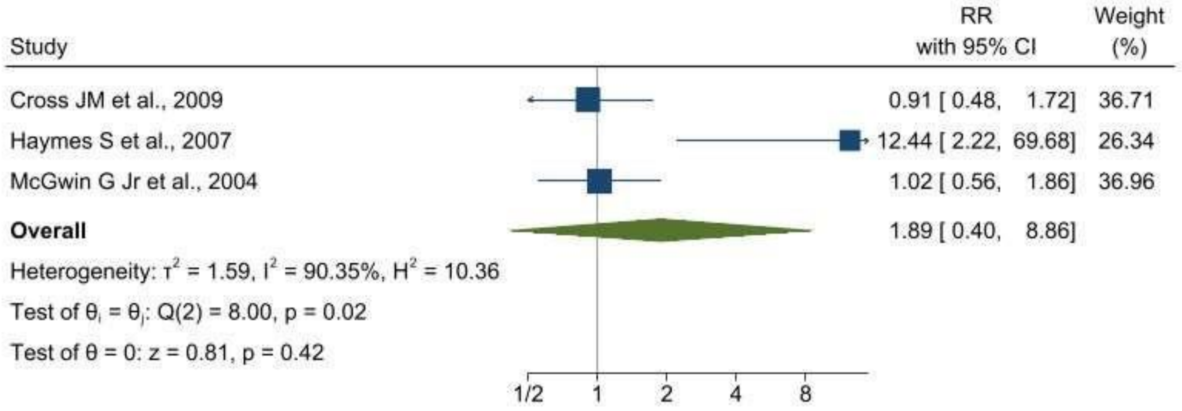

<table>
<tr><td>Cataract</td><td>Any MVC</td><td>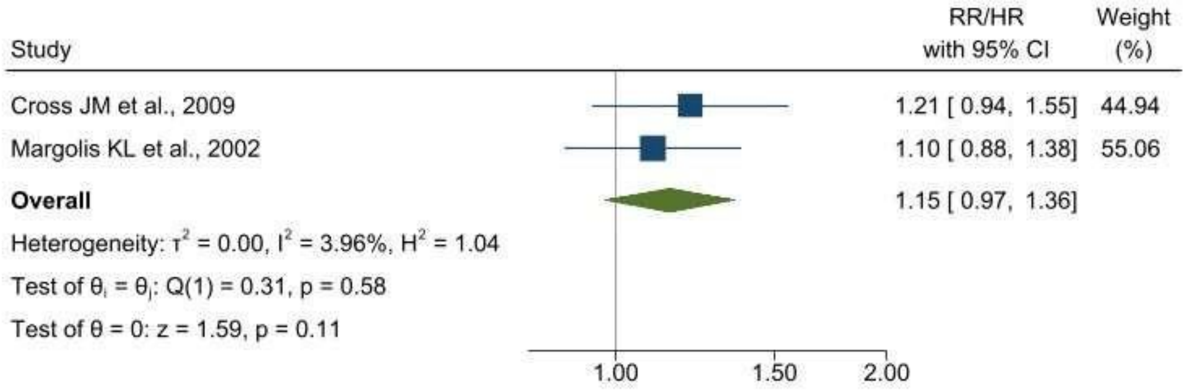

NOTE: POOLED ONE STUDY WITH RR AND ANOTHER WITH HR</td></tr>
<tr><td>Stereopsis</td><td>Any MVC</td><td>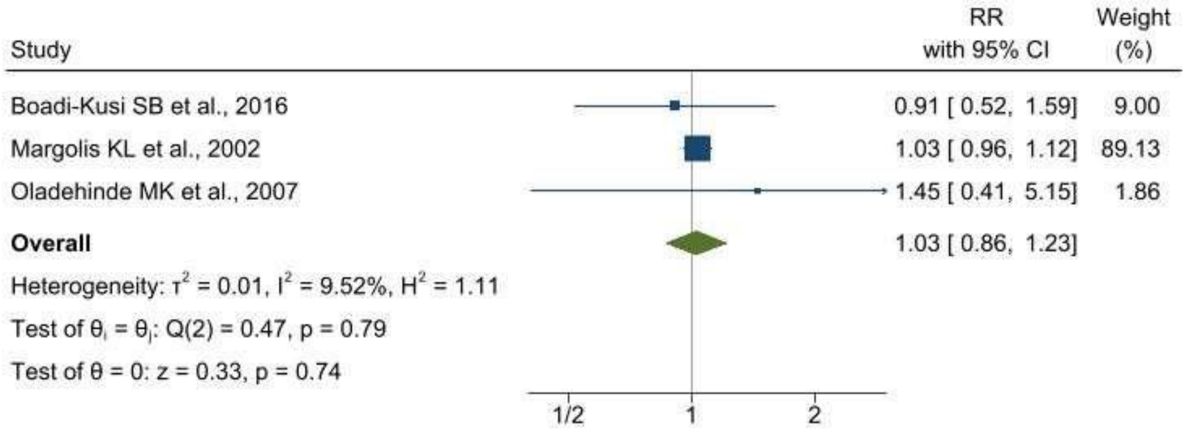
</td></tr>
</table>

<table>
<tr><th>Myopia</th><th>Any MVC</th><th>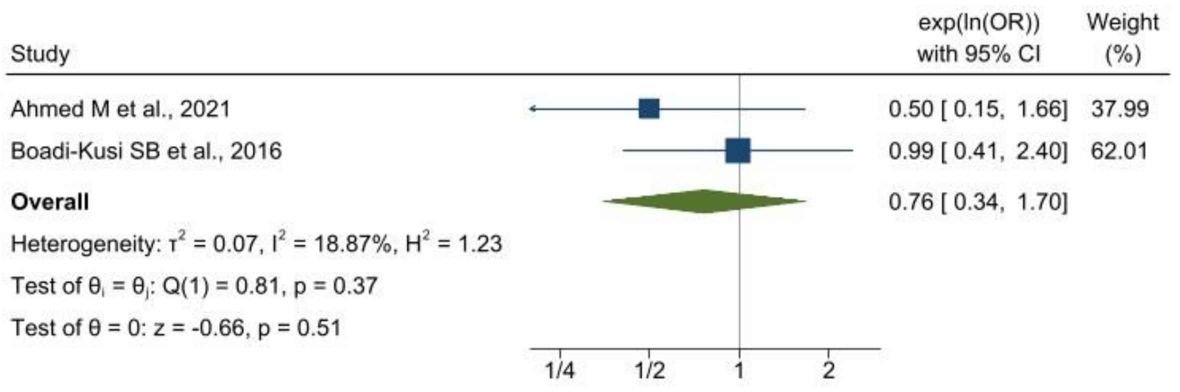
</th></tr>
</table>

For peer review only

VA ≤ 20/40	Any MVC	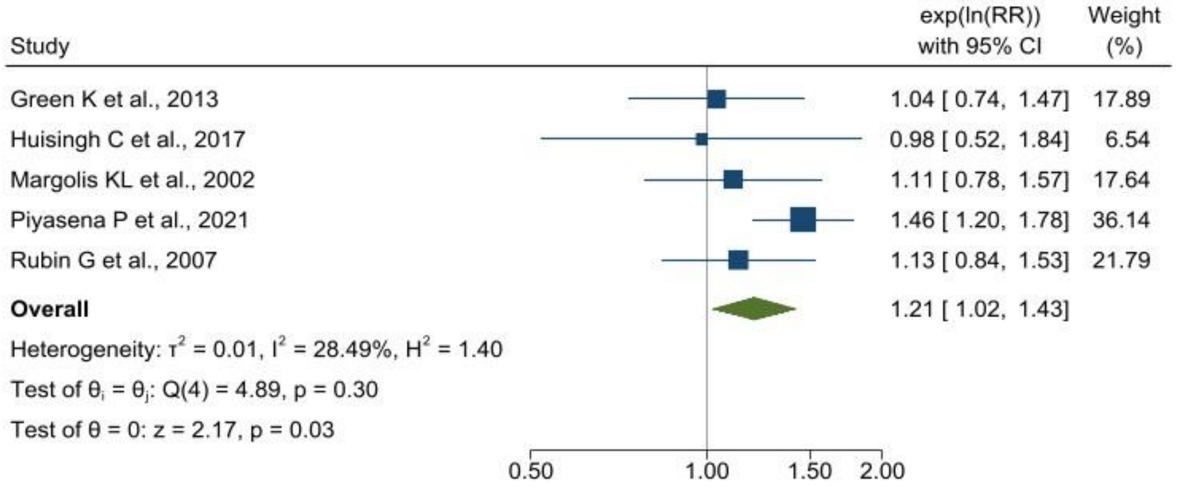
	At-fault MVC	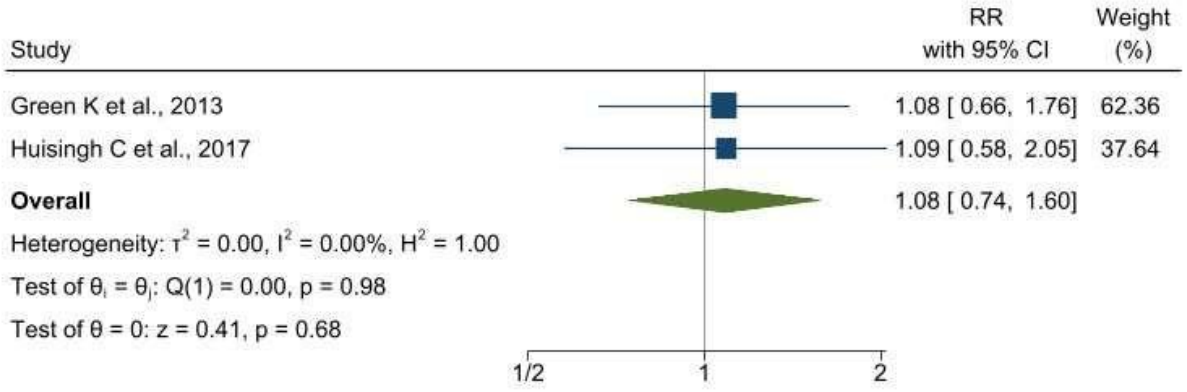

<table>
<tr><td>Contrast Sensitivity Loss</td><td>Any MVC</td><td>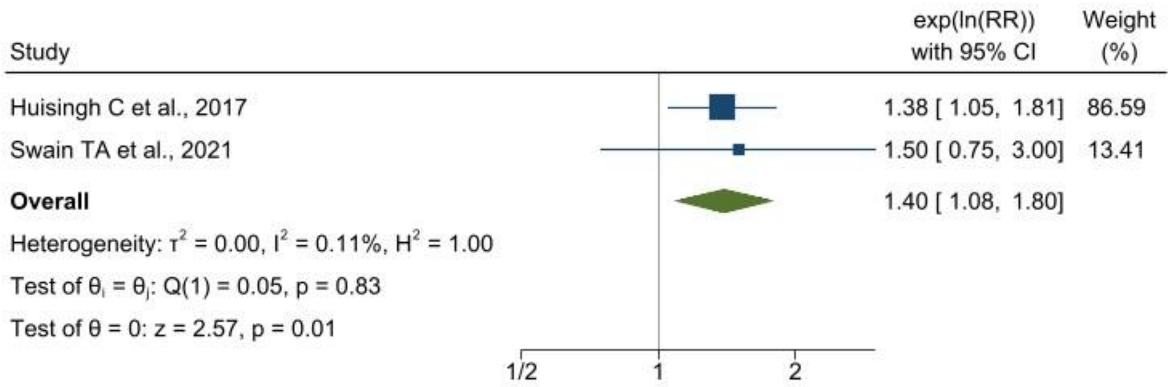
</td></tr>
<tr><td>Visual Field Loss</td><td>Any MVC</td><td>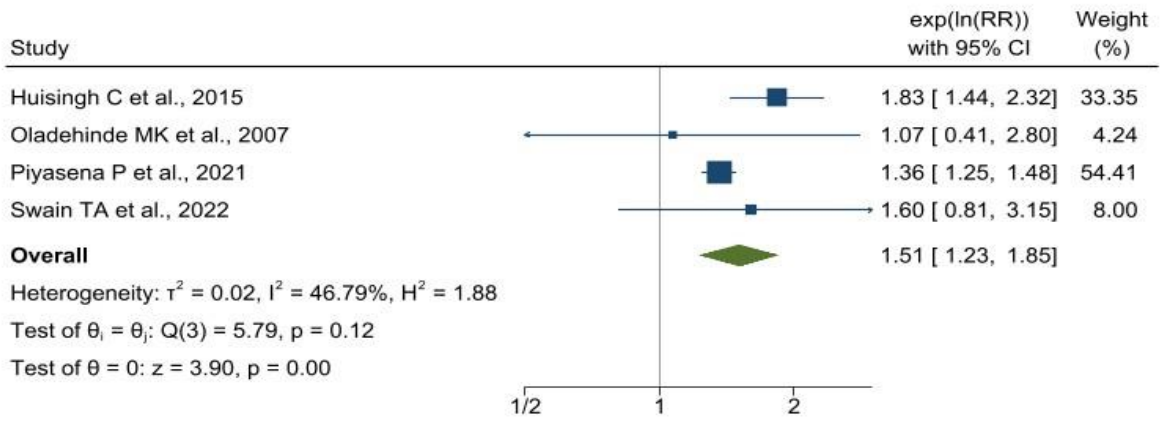
</td></tr>
<tr><td>Vision-related Intervention</td><td>Outcome</td><td>Association</td></tr>
</table>

**Cataract Surgery**	**Any MVC**	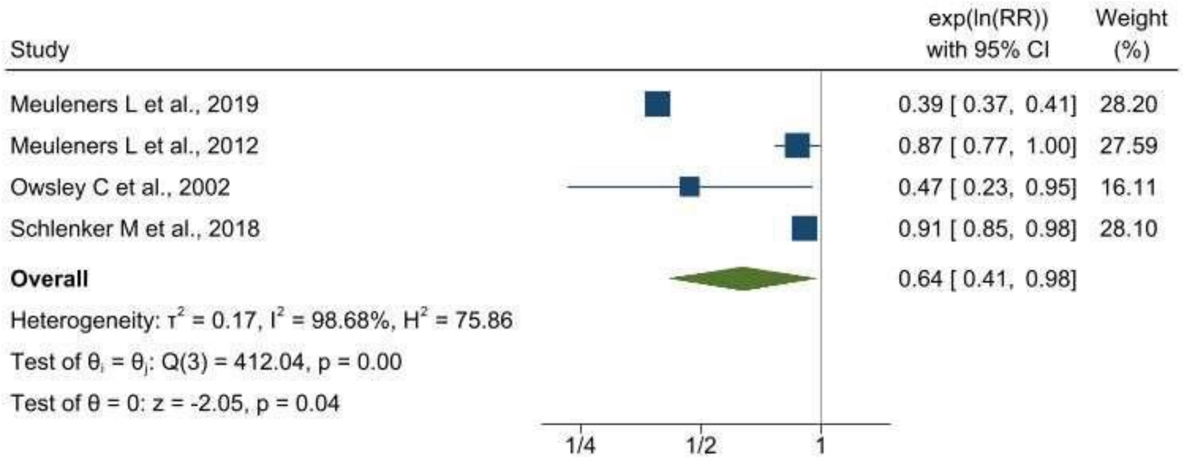<br>HOWEVER WHEN DROPPING SCHLENKER M ET AL., 2018 (UNADJUSTED EFFECT MEASURE):<br><br>**Overall** RR 0.55 [0.35, 0.92]<br>Heterogeneity: T^2 = 0.17, I^2 = 97.10%%, H^2 = 34.52<br>Test of $\theta_i = \theta_j$: Q(2) = 125.66, p = 0.00<br>Test of θ = 0: z = -2.30, p = 0.02

Appendix 5b Associations between driving cessation and vision impairments

<table>
<tr><th>Vision impairment</th><th>Association</th></tr>
<tr><td>Glaucoma</td><td>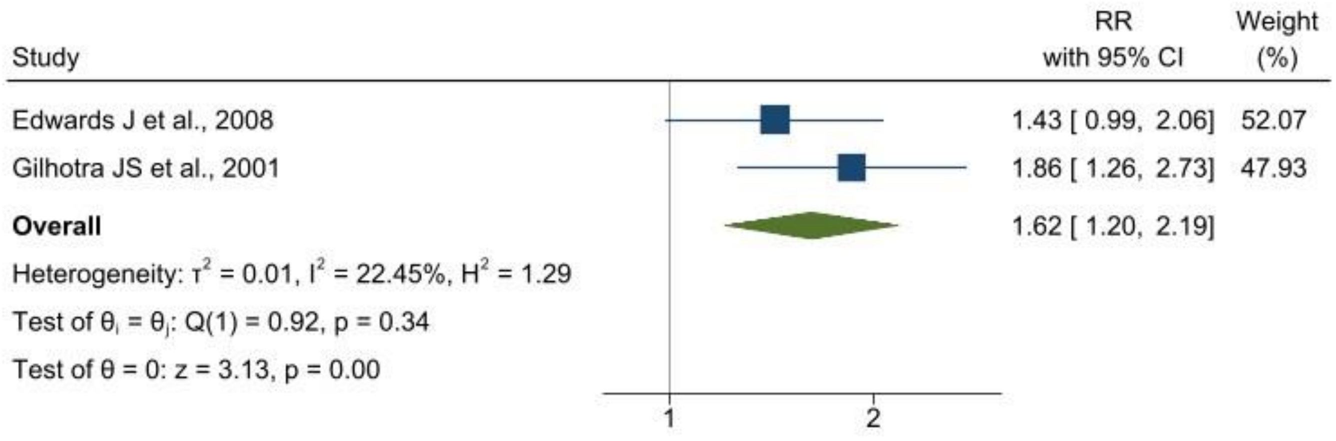
</td></tr>
<tr><td>AMD</td><td>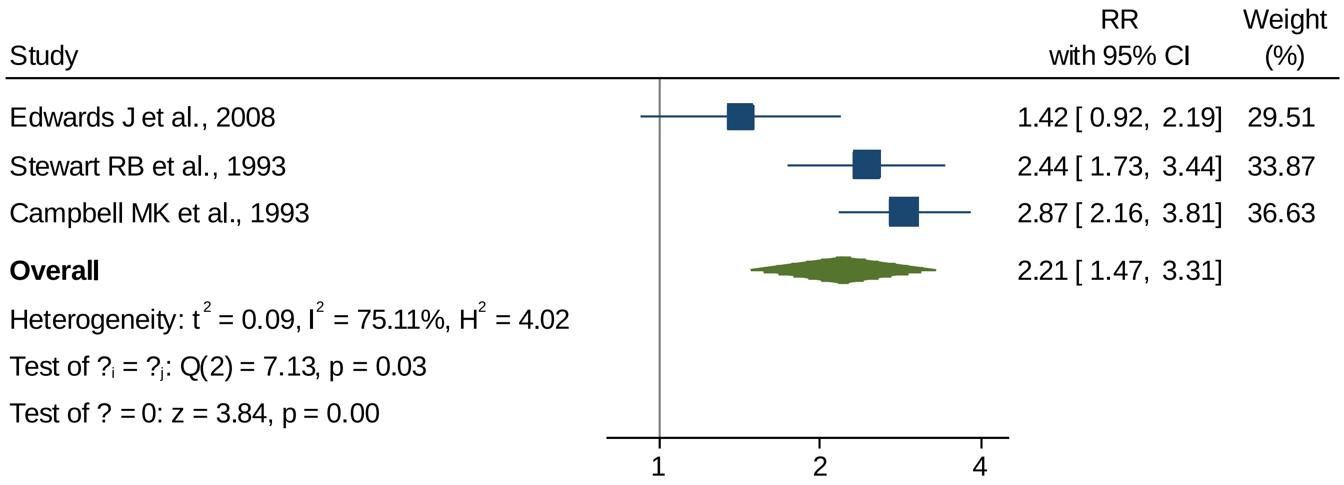
</td></tr>
<tr><td>Contrast Sensitivity</td><td></td></tr>
</table>

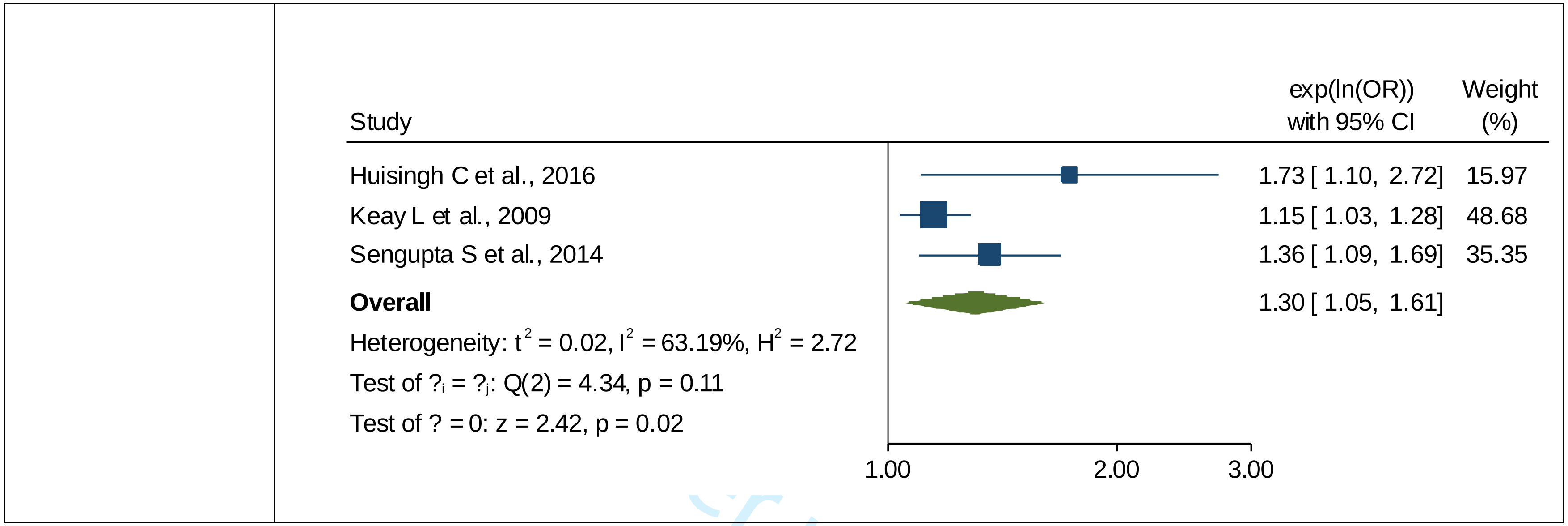
Study
exp(ln(OR)) with 95% CI
Weight (%)
Huisingh C et al., 2016
1.73 [1.10, 2.72]
15.97
Keay L et al., 2009
1.15 [1.03, 1.28]
48.68
Sengupta S et al., 2014
1.36 [1.09, 1.69]
35.35
Overall
1.30 [1.05, 1.61]
Heterogeneity: t² = 0.02, I² = 63.19%, H² = 2.72
Test of ?ᵢ = ?ⱼ: Q(2) = 4.34, p = 0.11
Test of ? = 0: z = 2.42, p = 0.02
1.00
2.00
3.00

For peer review only
